# Identification of Diabetic Retinopathy Genes through a Genome-Wide Association Study among Mexican-Americans from Starr County, Texas

**DOI:** 10.1155/2010/861291

**Published:** 2010-09-02

**Authors:** Yi-Ping Fu, D. Michael Hallman, Victor H. Gonzalez, Barbara E. K. Klein, Ronald Klein, M. Geoffrey Hayes, Nancy J. Cox, Graeme I. Bell, Craig L. Hanis

**Affiliations:** ^1^Human Genetics Center, School of Public Health, The University of Texas Health Science Center at Houston, P.O. Box 20186, Houston, TX 77225, USA; ^2^Valley Retina Institute, McAllen, TX 78503, USA; ^3^Department of Ophthalmology and Visual Sciences, University of Wisconsin School of Medicine and Public Health, Madison, WI 53705, USA; ^4^Division of Endocrinology, Feinberg School of Medicine, Northwestern University, Chicago, IL 60611, USA; ^5^Department of Human Genetics, The University of Chicago, Chicago, IL 60637, USA

## Abstract

To identify genetic loci for severe diabetic retinopathy, 286 Mexican-Americans with type 2 diabetes from Starr County, Texas, completed physical examinations including fundus photography for diabetic retinopathy grading. Individuals with moderate-to-severe non-proliferative and proliferative diabetic retinopathy were defined as cases. Direct genotyping was performed using the Affymetrix GeneChip Human Mapping 100 K Set, and SNPs passing quality control criteria were used to impute markers available in HapMap Phase III Mexican population (MXL) in Los Angeles, California. Two directly genotyped markers were associated with severe diabetic retinopathy at a *P*-value less than .0001: SNP rs2300782 (*P* = 6.04 × 10^−5^) mapped to an intron region of CAMK4 (calcium/calmodulin-dependent protein kinase IV) on chromosome 5, and SNP rs10519765 (*P* = 6.21 × 10^−5^) on chromosomal 15q13 in the FMN1 (formin 1) gene. Using well-imputed markers based on the HapMap III Mexican population, we identified an additional 32 SNPs located in 11 chromosomal regions with nominal association with severe diabetic retinopathy at *P*-value less than .0001. None of these markers were located in traditional candidate genes for diabetic retinopathy or diabetes itself. However, these signals implicate genes involved in inflammation, oxidative stress and cell adhesion for the development and progression of diabetic retinopathy.

## 1. Introduction

Diabetic retinopathy is a common microvascular complication of diabetes and remains one of the leading causes of blindness throughout the world [1]. It is estimated that 4.1 million Americans have diabetic retinopathy, which causes 12,000 to 24,000 new cases of blindness every year [2]. National Health Interview Survey and US census data lead to projections that the number of Americans 40 years or older having diabetic retinopathy will triple from 5.5 millions in 2005 to 16 millions in 2050 [3]. Although the underlying mechanisms leading to diabetic retinopathy have not been clarified, many risk factors have been reported, including poor glycemic control, longer diabetes duration, hypertension, hyperlipidemia, and albuminuria [4–7]. Evidence from ethnic and family studies has implicated genetic susceptibility for diabetic retinopathy. Mexican-Americans and African-Americans have been reported to have higher prevalence and worse severity of diabetic retinopathy in the US population when compared to non-Hispanic Whites [8, 9]. The Diabetes Control and Complications Trial (DCCT) showed a 3.1 times increased risk of severe retinopathy for individuals with retinopathy-positive relatives, and a correlation of retinopathy severity of 0.187 for all family members [10]. Similar familial clustering of diabetic retinopathy was also found among South Indians [11] and Mexican-Americans [12] with type 2 diabetes. The FIND-Eye study recently reported the broad sense heritability for diabetic retinopathy as 27% overall and 24% in Mexican-American families [13].

The suggestive genetic contribution to diabetic retinopathy has lead to the search for candidate genes and for genome-wide linkage between genetic markers and this complex disease, but no conclusive loci have been identified or replicated [14, 15]. Affected sibpair analysis in Pima Indians showed some evidence for linkage to diabetic retinopathy on chromosome 3 and 9 [16], and another signal on chromosome 1 was found in a subsequent study of the same population [17]. Our group also performed a linkage scan in Mexican-Americans from Starr County, Texas, and proposed 25 potential candidate genes for diabetic retinopathy under the linkage peaks on chromosome 3, 6, 12, 15, 19, and 20 [18].

As linkage studies lack power to identify alleles with modest effects or those that interact with other genetic or environmental factors on disease risk, genome-wide association studies (GWASs) using dense sets of SNPs across the genome have rapidly advanced our understanding of the genetic background of complex diseases [19]. The Starr County Health Studies has also reported GWAS results for type 2 diabetes among Mexican-Americans [20] with supportive replications from other studies [21–23]. Utilizing stereoscopic fundus photography within this population, we present here the first genome-wide association analysis of diabetic retinopathy among Mexican-Americans with type 2 diabetes from Starr County, Texas.

## 2. Materials and Methods

### 2.1. Study Subjects

This study includes 286 individuals with type 2 diabetes representing the earliest onset sibling having fundus photography from sibships with two or more type 2 diabetes-affected siblings from an ongoing genetic study of diabetic retinopathy among Mexican-Americans in Starr County, Texas [18, 20]. Baseline characteristics were obtained from personal interviews and physical examinations. Fasting blood samples and urine specimens were collected for glycemic, lipid, and microalbuminuria measurements, as previously described [24].

### 2.2. Genotyping

DNA samples extracted from participants were genotyped using the Affymetrix GeneChip Human Mapping 100 K Set [20, 25]. Genotypes were called using the GEL algorithm [26] given its improved call rate and consistent results with other calling methods [20]. According to the annotation file released in March, 2008 from the Affymetrix NetAffx Analysis Center (http://www.affymetrix.com/analysis/index.affx) and the National Center of Biotechnology Information (NCBI) Human Genome Build 36 data, we analyzed 112,666 autosomal SNPs that can be mapped to the NCBI Entrez SNP database and also defined their associated genes in this mapping 100 K set.

### 2.3. Diabetic Retinopathy Grading

All participants completed detailed ophthalmologic examinations including stereoscopic color fundus photography of the seven standard fields from the Diabetic Retinopathy Study (DRS) for each eye [27]. Photographs were sent to the University of Wisconsin Reading Center for examination and grading [28] using the Early Treatment Diabetic Retinopathy Study (ETDRS) adaptation of the modified Airlie House classification system [29]. The score for the more severely affected eye of an individual was used to classify retinopathy status. Since it has been shown that familial factors seem to especially influence the severity of diabetic retinopathy [10, 12], we focused this analysis on severe diabetic retinopathy and defined our cases and controls as follows. ETDRS grade 10–37: normal to early nonproliferative diabetic retinopathy (NPDR-E) as controls, and 43–85: moderate-to-severe nonproliferative diabetic retinopathy (NPDR-S) and proliferative diabetic retinopathy (PDR) as cases.

### 2.4. Quality Control and Population Stratification

Fisher's Exact tests of Hardy-Weinberg Equilibrium for controls and for all samples, as well as *χ* ^2^ tests for the distribution of missing genotypes between cases and controls, were conducted for all 112,666 autosomal SNPs. SNP quality was also assessed based on genotype call rate and minor allele frequency (MAF). A complete-linkage hierarchical clustering method implemented in PLINK [30] was conducted to explore any possible substructure among study subjects using pairwise identity-by-state (IBS) distance across the genome-wide SNP data. SNPs with a genotyping rate <95%, minor allele frequencies <1% in all study subjects, and *P*-value of exact Hardy-Weinberg Equilibrium test <.001 both in the entire study subjects and control group were tagged for potential quality control issues. A genomic inflation factor (*λ*) [31] and mean *χ* ^2^ statistics generated from all tested SNPs were also calculated to evaluate the effect of population stratification. The quantile-quantile plot of observed and expected distributions of *P*-values was used to assess any distortion of observed distribution from the null.

### 2.5. Imputation of Untyped SNPs

To expand the genomic coverage, we applied a computationally efficient hidden Markov Chain model [32] programmed in MACH (http://www.sph.umich.edu/csg/abecasis/MACH/) [33] to impute autosomal genotypes that were present in HapMap Phase III data in the Mexican population from Los Angeles, California, but not genotyped in the Affymetrix 100 K SNP set. An average allele dosage was estimated with 100 iterations of the imputation algorithm conditional on a set of known haplotypes while simultaneously estimating the recombination map. The squared correlation (*r* ^2^) between imputed and true genotypes was estimated for each SNP to evaluate imputation performance [33]. The mask option within MACH was also used to hide 2% of genotypes from the haplotyping, and imputed genotypes at these locations were compared with the actual genotypes to estimate the imputation error rate.

### 2.6. Statistical Analysis

*χ* ^2^ tests, Student *T*-tests, and logistic regressions were used to compare the basic characteristics between cases and controls using SAS/STAT system (SAS Institute Inc., Cary, NC). For single-marker case-control analyses, we did not initially filter any SNPs for quality-control reasons, but we labeled them with quality-control indicators for data interpretation. Logistic regression under an additive genetic model was performed for each directly genotyped SNP adjusting for the effects of age, gender, diabetes duration, and serum glycosylated hemoglobin level using PLINK [30]. Imputed markers with poor performance were filtered to obtain more reliable association results based on the per marker quality measures generated from MACH. A cutoff of 0.5 for *r* ^2^ between imputed and true genotypes was applied to remove about 90% of poorly imputed SNPs at a cost of 5% of good ones. To account for the probability distribution and the uncertainty of genotype imputation, logistic regression modeling adjusted for the same covariates was performed on these well-imputed markers using ProbABEL () from the ABEL set of programs [34, 35], where the SNP effect was assessed by its average imputed allele dosage.

## 3. Results

Among 286 Mexican-Americans with type 2 diabetes from Starr County, Texas, 103 (36%) with severe nonproliferative or proliferative diabetic retinopathy were defined as cases. Compared to those without retinopathy or only with modest nonproliferative retinopathy, individuals with severe diabetic retinopathy had significantly longer duration of diabetes, higher glycohemoglobin levels, and higher systolic blood pressure measurements (all *P*-values of Student *t*-test <.005, Table 1) [36]. 

The average genotype call rate among study subjects was 93%, with 14% of the 112,666 autosomal SNPs having a call rate less than 90%, and 4% having *P*-values for *χ* ^2^ tests for genotype missingness between cases and controls less than  .01. All polymorphic SNPs in this study (*n* = 111,528) remained in the single-marker analysis, and 5641 (5%) of them had minor allele frequency (MAF) less than 1%. The mean *χ* ^2^ statistics and the genomic inflation factor (*λ*) were 1.02 and 1.06, respectively, suggesting that any observed associations will unlikely be due to population stratification. However, there might be a slightly increased false positive rate since both values are greater than 1. There was no subpopulation identified from our study subjects using the IBS clustering analysis, and none of the permutation tests of IBS differences between cases and controls were significant (data not shown). The Q-Q plot in Supplementary Figure 1 also shows no strong evidence of population stratification, but with a deviation toward null possibly due to decreased power. 

**Figure 1:**
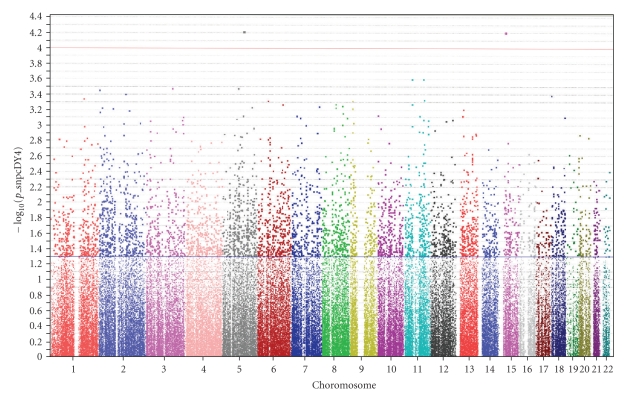
Summary of genome-wide associations between 111,528 SNPs and severe diabetic retinopathy under additive genetic model adjusted for age, gender, diabetes duration, and HbA1c level.

Figure 1 shows the *P*-value distribution for single-marker associations with severe retinopathy under an additive genetic model adjusted for age, gender, diabetes duration, and glycosylated hemoglobin level across the whole genome using the Affymetrix 100 K SNP set. All the top signals met all quality control criteria. Detailed information for the 10 strongest single marker associations is presented in Table 2. (Results for all SNPs are available as Supplementary Table 1) The best signals found in this study were rs2300782 (*P* = 6.04 × 10^−5^) in 5q21 and rs10519765 (*P* = 6.21 × 10^−5^) in 15q13, that locate in the intron of the calcium/calmodulin-dependent protein kinase IV (CAMK4) gene and formin 1 (FMN1) gene, respectively. 

The 100 K SNP set alone is insufficient to cover most variants currently available, therefore, we extended the analysis to an imputation-based GWAS using HapMap data [37]. To improve imputation performance, one subject with an overall genotyping rate less than 90% and 8,094 SNPs not meeting quality control criteria were excluded from imputation. A total of 1,326,990 SNPs from the HapMap Phase III Mexican population in Los Angeles, California, were imputed on 102 cases and 183 controls of severe diabetic retinopathy based on 104,572 directly genotyped SNPs. Supplementary Table 2 summarizes the imputation performance of MACH [33] across the whole genome. More than 88% of the imputed SNPs had a minor allele frequency greater than 1%, and the MACH estimated per allele error rate after masking 2% of direct genotypes from imputation was 8.7%. For all imputed markers, the overall *r* ^2^ between imputed and true genotypes was 0.387, and the average posterior probability for the most likely genotypes was 0.783. The 421,010 (31.7%) markers considered well imputed with an *r* ^2^ ≥ 0.5 were used for subsequent association analysis.

**Figure 2:**
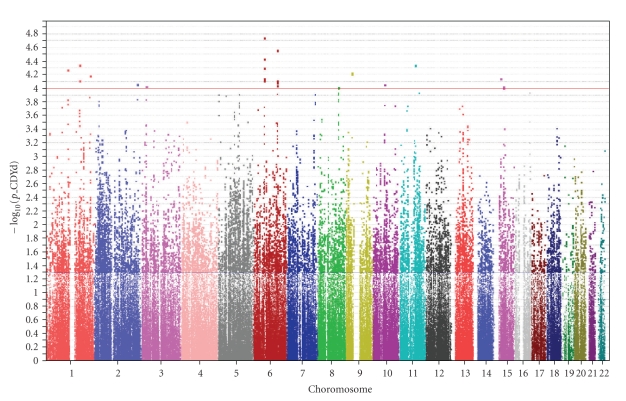
Summary of genome-wide associations between 421,010 well-imputed SNPs and severe diabetic retinopathy under additive genetic model adjusted for age, gender, diabetes duration, and HbA1c level.

Figure 2 shows the genome-wide *P*-value distribution from the logistic regression models using the well-imputed SNPs. The genomic inflation factor (*λ*) and the mean *χ* ^2^ statistics were 1.03 and 0.47, respectively, implying that any observed associations are unlikely due to population stratification. Table 3 summarizes the genes nearest the top signals (*P*-value less than 10^−4^) under logistic regression models adjusted for the effects of age, gender, diabetes duration, and glycosylated hemoglobin level. There were 32 SNPs associated with severe diabetic retinopathy at *P*-value less than 10^−4^, with 7 of these top signals in chromosomal region 6p11-12, where tubulointerstitial nephritis antigen (TINAG) is encoded. 

## 4. Discussion

Genome-wide association studies provide an additional tool, in conjunction with candidate gene and linkage studies to better understand the genetics of diabetic retinopathy. Our strongest signals from single-marker analysis implicated several genes as associated with severe diabetic retinopathy. SNP rs2300782 is located in an intronic region of CAMK4; its product has been reported within the Ca(2+)/calmodulin-dependent protein kinase subfamily and has been shown to increase transcriptional activity required for ATF-2 induced insulin gene expression [38]. The second best single-marker signal rs10519765 is located in FMN1, encoding a protein involved in cell adhesion and morphogenesis by assembling radial action cables in epithelial cells [39, 40]. However, there is very limited evidence about genetic variation on CAMK4 or FMN1 and diabetes or its complications. 

CNTN5 (contactin 5) at 11q22 is also among the nominally associated genes for severe diabetic retinopathy. The protein encoded by CNTN5 belongs to an immunoglobulin superfamily and may participate in the developing nervous system [41]. SNP variants in CNTN5 have been reported to be associated with atrial fibrillation and heart failure [42]. Another top single-marker signal was in COLEC12 (collectin subfamily member 12), encoding a protein of the C-lectin family, that possesses collagen-like sequences and carbohydrate recognition domains. This protein is a scavenger receptor recognizing oxidized phospholipids, so it may participate in removing oxidative damage [43], which is a potential etiological factor for diabetic retinopathy [44]. API5, EDIL3, BFSP2, HNMT, and SCYL1BP1 were also among the top signals from the single-marker analysis within their associated region, but there is no clear connection currently between these genes and diabetes or its complications.

Imputation of untyped SNP markers is as a useful tool to improve the coverage and power for genome-wide association studies without additional genotyping costs [45, 46]. A number of programs have been developed and routinely used to impute genotypes based on the observed haplotype structure from millions of SNPs in the HapMap project [37] or 1000 Genomes Project [47]. Given the uneven coverage of the genome by the Affymertrix 100 K SNP set, a more detailed SNP map was obtained by imputation conducted in MACH [32], since it consistently outperformed other algorithms with better accuracy and efficiency [48, 49]. The Mexican-American population in Starr County is relatively homogenous with 97.5% self-reporting as Hispanic. Genetically, this population is admixed with 68% European, 27% Asian, and 6% African ancestry [20]. Considering the importance of linkage disequilibrium pattern between the reference panel and study subjects, we used the HapMap III population with Mexican ancestry from Los Angles, California, who identified themselves as having at least three grandparents born in Mexico, as the reference set for imputation. The overall 8.7% estimate from MACH of overall allele error rate was comparable with the original MACH evaluation data (7.5%) [32]. Compared to all the autosomal SNP variants (*n* = 1,387,466) available in the HapMap III Mexican population, the genomic coverage in the imputation analysis increases extensively with 30.3% (*n* = 421,010) of the variants tested here, whereas only 4.3% (*n* = 60,283) of them are directly covered in the Affymetrix 100 K SNP set. 

Among all imputed markers associated with severe diabetic retinopathy at a *P*-value less than 10^−4^, there are 7 SNPs located in 6p11-12 where TINAG (tubulointerstitial nephritis antigen) is encoded. TINAG is a glycoprotein originally identified as a target antigen involved in human antitubular basement membrane disease [50]. It has been recognized as an extracellular matrix protein and shows increased expression in the kidney of streptozotocin-diabetic rats [51], but there is yet no evidence how TINAG might be involved in human diabetes or its complications. Chromosomal region 15q13, where FMN1 (formin 1) and GREM1 (gremlin 1) map, also has 4 of the strongest imputation-based signals. FMN1 has one of the strongest associations from direct genotyping (rs10519765). Gremlin is located ~40 kb downstream of FMN1, a highly conserved protein involved in various disorders related to fibrotic changes in the kidney, lung, and liver [52]. This protein has been shown to have a potential role in diabetic nephropathy because of enhanced expression and interaction with TGF-*β* signaling pathways [53, 54]. Recent evidence of increased gremlin mRNA in bovine retinal perictyes [55] and immunohistochemistry data on the mouse retina [54] coupled with results reported here elevate interest in gremlin. 

In addition to identifying novel candidate genes, we were interested in SNPs among genes that appear to be traditional candidates for diabetic retinopathy. A list of 208 retinopathy candidate genes has been generated [18, 56] (see Supplementary Table 3) to include genes involved in metabolic processes or clinical risk factors leading to retinopathy and those that have been previously reported to be associated with diabetic retinopathy. Among a total of 667 SNPs that are located in the coding region of these 208 candidate genes and also genotyped in the Affymetrix 100 K SNP set, 54 of them had nominal allelic association with diabetic retinopathy with *P*-values ranging from  .001 to  .05 (Supplementary Figure 2). The significance pattern found here is more than expected by chance alone. 

Although we cannot completely exclude the possibility of undetected subpopulations affecting the results, we found no evidence of population stratification. Hayes et al. also used the genome-wide SNP data to estimate proportions of ancestry on the same population. Ancestry is unlikely to be a source of spurious association in this sample since the amount of African, Asian, and European ancestries were indistinguishable between cases and controls [20].

The primary objective of this study is searching for diabetic retinopathy genes without any assumption about the location of disease-associating variants. This initial genome-wide association analysis of severe diabetic retinopathy identified several unexpected loci that may contribute to the genetic susceptibility of diabetic retinopathy. The study was limited, however, in its ability to detect small effect sizes. A sample of 103 cases and 183 controls was only powerful enough to identify variants with odds ratios greater than 2.2 (assuming disease allele frequency of 0.5 and disease prevalence of 0.5 in an additive model, calculated by CaTS [57]) with a genome-wide significance level (*P*-value less than 5 × 10^−8^). On the other hand, genetic association studies usually require replication in appropriate independent samples to validate any findings. No such comparable data among Mexican-Americans are available though we are expanding the sample and SNP coverage, even so, these results set some limits as to the magnitude of genetic effects for diabetic retinopathy. 

In summary, we observed several SNPs and genes associated with severe diabetic retinopathy in this initial genome-wide analysis. None of these loci have been previously linked to diabetic retinopathy or diabetes itself. While the underlying mechanisms leading to diabetic retinopathy remain unresolved, these results implicate genetic regulation of oxidative stress and cell adhesion as possible players in the development of diabetic retinopathy.

## Supplementary Material

Supplementary materials contains:
Supplementary Figure 1. Q-Q plot of logistic regression models adjusted for age, gender, diabetes duration and HbA1C level.
Supplementary Figure 2. Q-Q plot of SNPs located in coding region of 208 diabetic retinopathy candidate genes.
Supplementary Table 1. Detail information of SNPs with pvalues of Fisher's Exact allelic test less than 0.05.
Supplementary Table 2. Imputation quality of SNP markers used in MACH.
Supplementary Table 3. A list of 208 retinopathy candidate genes has been generated.

## Figures and Tables

**Table 1:** Basic characteristics of Mexican-Americans with type 2 diabetes from Starr County, Texas.

Variables		No DR + NPDR-E^#^	NPDR-S + PDR^#^	*P*-value^§^	OR	(95%CI)
		(controls *n* = 183)	(cases *n* = 103)			
Age	Mean ± SD	57.06 ± 11.54	58.67 ± 9.27	.1988	1.01	(0.99–1.04)
Gender	Male	66 (36.07%)	43 (41.75%)	.3422	1.00	—
	Female	117 (63.93%)	60 (58.25%)		0.79	(0.48–1.29)
Albumin	Negative	132 (74.58%)	57 (60.64%)	.0802	1.00	—
	20 mg/L	22 (12.43%)	14 (14.89%)		1.47	(0.70–3.08)
	50 mg/L	17 (9.60%)	17 (18.09%)		2.32	(1.10–4.86)*
	100 mg/L	6 (3.39%)	6 (6.38%)		2.32	(0.72–7.49)
Hypertension history	No	96 (54.24%)	59 (59.60%)	.3895	1.00	—
	Yes	81 (45.76%)	40 (40.40%)		0.80	(0.49–1.32)
Diabetes duration (yrs)	mean ± SD	12.02 ± 9.09	18.10 ± 8.22	<.0001	1.08	(1.05–1.11)***
HbA1C (%)	mean ± SD	10.87 ± 3.72	12.29 ± 3.90	.0028	1.10	(1.03–1.18)***
Fasting glucose (mg/dL)	mean ± SD	185.54 ± 68.71	201.63 ± 84.49	.0883	1.03	(0.99–1.06)
BMI (kg/m^2^)	mean ± SD	31.82 ± 6.49	30.85 ± 5.93	.2175	0.98	(0.94–1.02)
Systolic BP (mmHg)	mean ± SD	126.79 ± 17.86	134.73 ± 23.26	.0037	1.21	(1.07–1.38)***
						(every 10 unit)
Diastolic BP (mmHg)	mean ± SD	72.69 ± 10.03	73.32 ± 10.78	.6290	1.06	(0.84–1.35)
						(every 10 unit)
Total cholesterol (mg/dL)	mean ± SD	191.21 ± 41.81	199.06 ± 42.13	.1381	1.05	(0.99–1.11)
						(every 10 unit)
Triglycerides (mg/dL)	mean ± SD	184.79 ± 120.92	210.02 ± 123.59	.1031	1.02	(0.99–1.04)
						(every 10 unit)
HDL cholesterol (mg/dL)	mean ± SD	42.86 ± 11.73	40.62 ± 10.32	.1153	0.83	(0.66–1.05)
						(every 10 unit)
LDL cholesterol (mg/dL)	mean ± SD	111.49 ± 30.71	118.94 ± 34.84	.0790	1.08	(0.99–1.16)

^#^No DR: normal or nondiabetic retinopathy; NPDR-E: early nonproliferative diabetic retinopathy

^#^NPDR-S: moderate-to-severe nonproliferative diabetic retinopathy; PDR: proliferative diabetic retinopathy

^§^ *P*-value of *χ* ^2^ test or Student *t*-test

*P-value < .05, **P-value < .01, ***P-value < .005.

**Table 2:** Top 10 single-marker associations with severe diabetic retinopathy among Mexican-Americans from Starr County, Texas.

dbSNP rs#	Chr	Position*	Minor/major allele	MAF in Cases	MAF in Controls	*P*-value^§^	OR^+^ (95%CI)	Nearest gene*	Distance/ relationship to gene
rs2300782	5	110816684	A/G	0.512	0.322	6.04*E* − 05	2.64 (1.64–4.25)	CAMK4	intron/0
rs10519765	15	30992716	A/G	0.136	0.294	6.21*E* − 05	0.30 (0.16–0.54)	FMN1	intron/0
rs899036	11	41639486	C/A	0.125	0.210	2.52*E* − 04	0.32 (0.17–0.59)	API5	upstream/ 1650623
rs10501943	11	99452209	C/T	0.195	0.086	2.53*E* − 04	3.04 (1.68–5.52)	CNTN5	intron/0
rs1445754	5	83611387	A/T	0.143	0.273	3.35*E* − 04	0.37 (0.22–0.64)	EDIL3	intron/0
rs1197310	3	134610914	T/A	0.540	0.435	3.35*E* − 04	2.25 (1.45–3.51)	BFSP2	intron/0
rs699549	2	4683138	T/C	0.115	0.043	3.49*E* − 04	4.27 (1.93–9.47)	—	—
rs763970	2	138352603	A/C	0.330	0.206	4.00*E* − 04	2.25 (1.44–3.52)	HNMT	upstream/ 85675
rs599019	18	284495	G/T	0.030	0.131	4.06*E* − 04	0.15 (0.05–0.43)	COLEC12	downstream/ 24861
rs6427247	1	168647104	G/A	0.360	0.221	4.56*E* − 04	2.17 (1.41–3.35)	SCYL1BP1	upstream/ 120790

*Affymetrix NetAffx annotation build 25, NCBI genome build 36.1.

^§^ *P*-value of logistic regression under additive genetic model, adjusted for age, gender, diabetes duration, and HbA1c level.

^+^odds ratios of the minor allele.

**Table 3:** Regions with strongest imputation-inferred associations (*P* < .0001) using estimated allelic dosage under an adjusted additive genetic model.

Location	SNP with best *P*-value within this region	Position*	Best *P*-value^§^	# of top SNPs within this region	Nearest gene*
6p11-12	rs6909083	54290262	1.80*E* − 05	7	TINAG
6q22	rs17083119	121443809	2.76*E* − 05	4	C6orf170
1q23	rs1033465	171254353	4.50*E* − 05	2	TNFSF18
1p13	rs11583330	109925036	5.35*E* − 05	1	GNAI3
1q42	rs3014267	225619540	6.58*E* − 05	1	CDC42BPA
15q13	rs11635920	30999949	7.18*E* − 05	4	FMN1, GREM1
2q35-36	rs6726798	219009099	8.66*E* − 05	1	VIL1
10q21	rs11812882	59712631	8.85*E* − 05	1	ZCD1
2q35	rs1106412	219023301	8.91*E* − 05	1	USP37
3p24	rs11927173	23200198	9.39*E* − 05	2	UBE2E2
8q22	rs3098241	104494480	9.72*E* − 05	2	SLC25A32

*Affymetrix NetAffx annotation build 25, NCBI genome build 36.1

^§^ *P*-value of logistic regression under additive genetic model, adjusted for age, gender, diabetes duration, and HbA1C level.
