# Supplementary material for: Identification of Diabetic Retinopathy Genes through a Genome-Wide Association Study among Mexican-Americans from Starr County, Texas
Source: J Ophthalmol. 2010 Sep 2;2010:861291. doi: 10.1155/2010/861291 (PMC2939442; doi:10.1155/2010/861291)

Supplementary Figure 1. Q-Q plot of logistic regression models adjusted for age, gender, diabetes duration and HbA1C level.

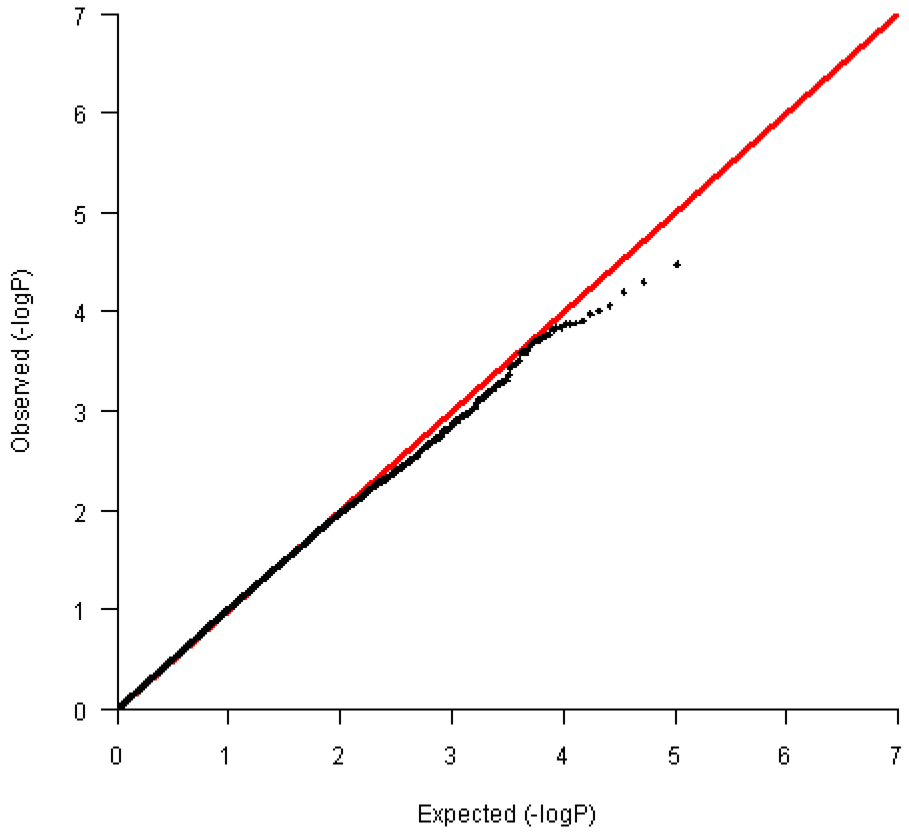

Supplement: Supplementary file 1 — Supplementary materials contains: Supplementary Figure 1. Q-Q plot of logistic regression models adjusted for age, gender, diabetes duration and HbA1C level. Supplementary Figure 2. Q-Q plot of SNPs located in coding region of 208 diabetic retinopathy candidate genes. Supplementary Table 1. Detail information of SNPs with pvalues of Fisher's Exact allelic test less than 0.05. Supplementary Table 2. Imputation quality of SNP markers used in MACH. Supplementary Table 3. A list of 208 retinopathy candidate genes has been generated. [file 861291.f1.pdf]
