# Supplementary material for: Identification of Diabetic Retinopathy Genes through a Genome-Wide Association Study among Mexican-Americans from Starr County, Texas
Source: J Ophthalmol. 2010 Sep 2;2010:861291. doi: 10.1155/2010/861291 (PMC2939442; doi:10.1155/2010/861291)

Supplementary Figure 2. Q-Q plot of SNPs located in coding region of 208 diabetic retinopathy candidate genes.

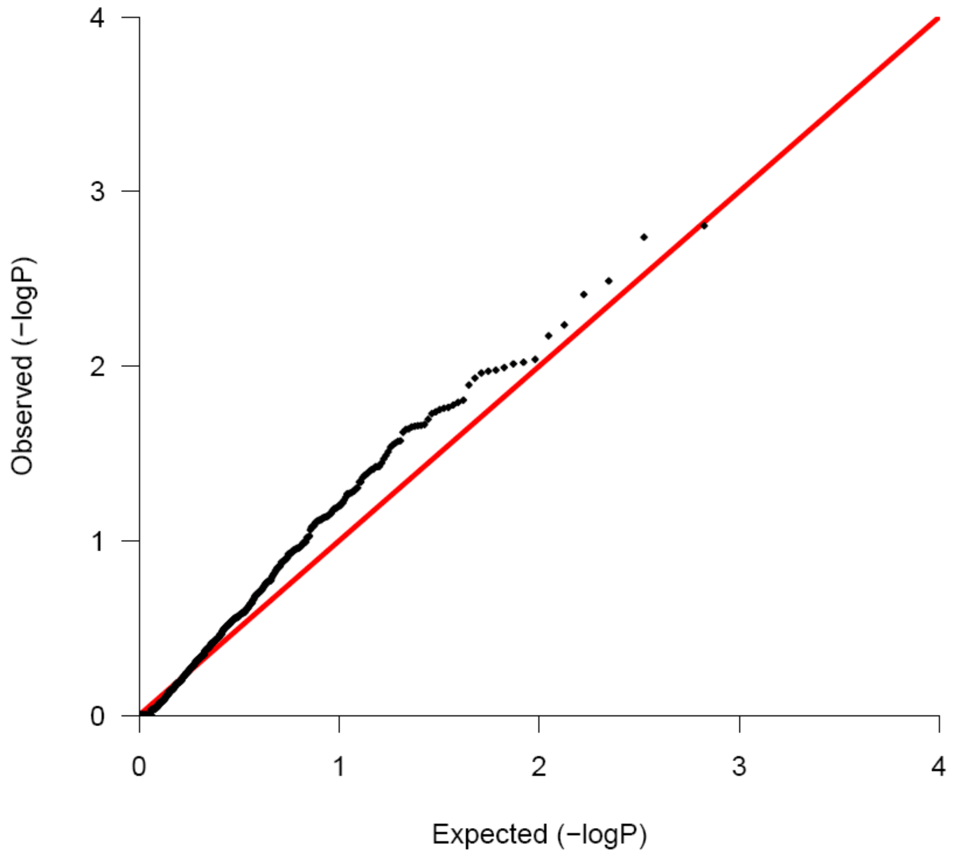

Supplement: Supplementary file 2 [file 861291.f2.pdf]
