# Supplementary material for: Identification of Diabetic Retinopathy Genes through a Genome-Wide Association Study among Mexican-Americans from Starr County, Texas
Source: J Ophthalmol. 2010 Sep 2;2010:861291. doi: 10.1155/2010/861291 (PMC2939442; doi:10.1155/2010/861291)
Supplement: Supplementary file 3 [file 861291.f3.pdf]

Supplementary Table 1. Detail information of SNPs with p-values of Fisher's Exact allelic test less than 0.05

SNP rs ID	Chr.	Physical position	Minor allele frequency	Genotype call rate	p-values for Exact tests of Hardy Weinberg Equilibrium		p-values of association tests		Transcript accession	SNP-gene relationship	Distance to the nearest gene	Unigene clutser ID	Nearest gene symbol
					All Samples	Controls Only	Fisher's Exact allelic test	Additive model adjusted for age, gender, diabetes duration, and HbA1C					
rs1889031	10	36887259	0.4594	95.09%	0.002154	0.067340	0.00003639	0.00428400	ENST00000279434	upstream	34091	---	---
rs599019	18	284495	0.0950	97.89%	0.289100	0.512600	0.00003639	0.00040560	NM_030781	downstream	24861	---	COLEC12
rs2505585	10	36888999	0.4896	84.56%	0.001016	0.015590	0.00005081	0.00373900	ENST00000279434	upstream	35831	---	---
rs3761296	20	16657662	0.3182	92.63%	0.888000	1.000000	0.00005412	0.00257500	NM_198220	upstream	980	Hs.280378	SNRPB2
rs2300782	5	110816684	0.3873	85.61%	0.787200	1.000000	0.00005741	0.00006040	NM_001744	intron	0	Hs.591269	CAMK4
rs10519765	15	30992716	0.2378	86.32%	0.725000	1.000000	0.00006387	0.00006208	ENST00000334528	intron	0	---	---
rs1537328	10	36889598	0.4640	97.54%	0.003751	0.131900	0.00006393	0.00622500	ENST00000279434	upstream	36430	---	---
rs2483021	10	36887138	0.4640	97.54%	0.003751	0.131900	0.00006393	0.00622500	ENST00000279434	upstream	33970	---	---
rs10495528	2	5975298	0.3638	90.18%	1.000000	0.875000	0.00007279	0.00068320	NM_003108	downstream	216330	Hs.432638	SOX11
rs1558068	7	30469888	0.4325	96.14%	0.175500	0.197700	0.00007412	0.00075640	NM_006092	intron	0	Hs.405153	NOD1
rs1317318	15	50920355	0.0484	97.89%	0.483500	1.000000	0.00011370	0.01634000	NM_004498	upstream	50854	Hs.658573	ONECUT1
rs2023139	8	118469892	0.3040	97.54%	0.394400	0.517300	0.00012170	0.00137900	NM_080651	upstream	132319	Hs.492612	MED30
rs993871	7	48964483	0.3459	93.33%	0.278200	0.462800	0.00012380	0.00080400	NM_198570	upstream	819315	Hs.677488	VWC2
rs903489	8	108718248	0.2113	83.86%	0.241700	1.000000	0.00012410	0.00069260	NM_001146	upstream	138789	Hs.369675	ANGPT1
rs993872	7	48964240	0.3370	94.74%	0.275200	0.708900	0.00012460	0.00146600	NM_198570	upstream	819558	Hs.677488	VWC2
rs724333	3	55933099	0.2273	92.63%	0.725600	0.417200	0.00015510	0.00125400	NM_015576	intron	0	Hs.476389	ERC2
rs1424548	7	136360300	0.3333	91.58%	0.889300	1.000000	0.00016930	0.00124800	NM_001006629	downstream	4761	Hs.535891	CHRM2
rs7921283	10	58219893	0.4415	92.98%	0.454500	0.442900	0.00019310	0.00170600	NM_001005414	upstream	428860	---	ZWINT
rs2226859	18	69195178	0.4926	94.74%	0.182000	0.048160	0.00021100	0.00078680	NM_138966	upstream	511264	Hs.465407	NETO1
rs10496192	2	73533374	0.1550	95.09%	0.643500	0.465200	0.00025300	0.00060690	NM_015120	CDS	0	Hs.184720	ALMS1
rs9918728	7	48896160	0.2760	97.89%	0.176500	0.389100	0.00025450	0.00347000	ENST00000381012	downstream	34393	---	---
rs10504455	8	70633156	0.1736	92.98%	1.000000	1.000000	0.00027780	0.00059110	NM_015170	intron	0	Hs.409602	SULF1
rs6544021	2	22606730	0.4923	90.88%	0.081510	0.536200	0.00029340	0.00105500	ENST00000384155	upstream	191350	---	---
rs10485100	6	92434626	0.0343	97.19%	1.000000	1.000000	0.00030470	0.99700000	NM_145333	upstream	1080998	Hs.644143	MAP3K7
rs10504454	8	70632908	0.1741	94.74%	1.000000	1.000000	0.00030660	0.00052840	NM_015170	intron	0	Hs.409602	SULF1
rs957940	13	53199641	0.0572	95.09%	0.045920	0.092540	0.00032750	0.00743700	ENST00000387037	downstream	469133	---	---
rs764637	2	211349248	0.0608	89.47%	1.000000	1.000000	0.00033000	0.00454900	NM_001875	downstream	97174	Hs.149252	CPS1
rs10502259	11	121089293	0.0278	94.74%	1.000000	1.000000	0.00033690	0.00101500	NM_003105	downstream	83696	Hs.368592	SORL1
rs2197713	3	74252038	0.1956	87.02%	0.224300	0.532800	0.00034830	0.00302200	ENST00000334941	downstream	16742	---	---
rs2226024	10	16238986	0.2278	94.74%	0.224600	0.131700	0.00035110	0.00110100	ENST00000364390	downstream	50840	---	---
rs10492603	13	57769822	0.2768	95.09%	0.543300	0.495600	0.00038690	0.00403900	ENST00000365514	downstream	229970	---	---
rs1428445	5	157928381	0.2078	85.26%	0.017680	0.294900	0.00039350	0.00966000	NM_024007	downstream	130316	Hs.657753	EBF1
rs1836216	2	133682647	0.4011	94.04%	1.000000	1.000000	0.00042340	0.00402100	NM_207481	intron	0	Hs.537329	NAP5
rs10508752	10	30293978	0.0186	94.39%	0.081630	1.000000	0.00043330	0.01260000	NM_021738	upstream	330071	Hs.499209	SVIL
rs10520201	4	172341431	0.1852	94.74%	0.008550	0.053700	0.00043460	0.00299800	NM_001034845	upstream	630876	Hs.386236	GALNT17
rs1445754	5	83611387	0.2264	96.84%	0.495600	0.569700	0.00043840	0.00033490	NM_005711	intron	0	Hs.482730	EDIL3
rs4860425	4	62225552	0.2379	94.39%	0.063000	0.352800	0.00044280	0.00562600	NM_015236	intron	0	Hs.694758	LPHN3
rs10509060	10	58211442	0.4364	96.49%	1.000000	0.426600	0.00048350	0.01132000	NM_001005414	upstream	420409	---	ZWINT
rs10485129	6	79427902	0.1325	94.04%	0.103700	0.641300	0.00048920	0.00389100	NM_001010844	upstream	206006	Hs.656212	IRAK1BP1
rs6427247	1	168647104	0.2706	97.89%	0.172600	1.000000	0.00049230	0.00045640	NM_152281	upstream	120790	Hs.183702	SCYL1BP1
rs10509061	10	58211893	0.4355	97.89%	0.715100	0.751200	0.00050350	0.00970500	NM_001005414	upstream	420851	---	ZWINT
rs2296041	13	42884771	0.1408	97.19%	0.803100	1.000000	0.00051570	0.00240900	NM_017993	intron	0	Hs.128258	ENOX1
rs3790568	1	67608648	0.1165	97.89%	0.775700	1.000000	0.00052690	0.00192400	NM_001559	intron	0	Hs.479347	IL12RB2
rs10517897	4	167504234	0.0466	86.67%	0.086690	0.162800	0.00053400	0.01547000	NM_016950	downstream	386933	Hs.481133	SPOCK3

rs10495679	2	18215886	0.0888	96.84%	0.706600	1.000000	0.00053630	0.00318200	NM_020905	downstream	383584	Hs.288880	RDH14
rs1463075	11	41588438	0.1315	94.74%	0.190600	0.273000	0.00053890	0.00052960	NM_006595	upstream	1701671	Hs.435771	API5
rs10501943	11	99452209	0.1245	94.39%	0.153200	0.363300	0.00054470	0.00025300	NM_175566	intron	0	Hs.656783	CNTN5
rs6500986	16	7553178	0.4259	94.74%	0.456600	0.745800	0.00054790	0.00519100	NM_018723	intron	0	Hs.459842	A2BP1
rs7184426	16	81379409	0.0604	95.79%	0.000105	0.000374	0.00055010	0.00603500	NM_001257	upstream	70051	Hs.654386	CDH13
rs10501855	11	95882837	0.1403	97.54%	0.453500	0.083310	0.00055030	0.00122600	ENST00000363541	upstream	35334	---	---
rs10506825	12	79213548	0.1734	96.14%	0.136500	0.261800	0.00056450	0.00087170	ENST00000378620	upstream	11366	---	---
rs7537126	1	171287972	0.0591	97.89%	0.059700	0.108500	0.00056620	0.00324100	NM_005092	upstream	1293	Hs.248197	TNFSF18
rs7923214	10	2283989	0.2401	97.89%	0.103400	0.477000	0.00062170	0.00074550	NM_002627	upstream	815723	Hs.26010	PFKP
rs10498405	14	48126417	0.0460	87.72%	0.084860	0.152900	0.00062680	0.03796000	ENST00000384636	upstream	246132	---	---
rs2320761	13	32381294	0.2168	68.77%	0.408200	0.300500	0.00062850	0.00075560	NM_015032	downstream	131137	Hs.699308	PDS5B
rs727845	7	67244644	0.2281	96.14%	0.863500	0.454300	0.00066990	0.00277100	ENST00000388515	upstream	43896	---	---
rs10483431	14	32751215	0.1022	96.14%	0.176900	0.203700	0.00068010	0.00969700	NM_022123	intron	0	Hs.659456	NPAS3
rs6929672	6	18208225	0.4945	95.79%	0.904000	0.283600	0.00070440	0.01092000	NM_198586	downstream	21404	Hs.348351	NHLRC1
rs10515652	5	151541471	0.1241	97.54%	0.780600	1.000000	0.00070680	0.00058190	NM_020167	downstream	209832	Hs.283093	NMUR2
rs952245	18	59218939	0.3090	74.39%	0.000052	0.037760	0.00071690	0.01239000	NM_004869	intron	0	Hs.126550	VPS4B
rs10495633	2	14230313	0.4675	97.19%	0.040850	0.164200	0.00071740	0.00266100	ENST00000388013	downstream	101884	---	---
rs2312215	2	69693329	0.3813	97.54%	0.162500	1.000000	0.00075900	0.04932000	NM_014911	intron	0	Hs.468878	AAK1
rs6425196	1	171248168	0.0484	97.89%	0.125200	0.200300	0.00081880	0.00536500	NM_005092	downstream	27555	Hs.248197	TNFSF18
rs7095137	10	84148528	0.3309	94.39%	0.492000	1.000000	0.00082750	0.01345000	NM_001010848	intron	0	Hs.125119	NRG3
rs4858389	3	22166090	0.2809	93.68%	0.649300	0.397000	0.00085270	0.00258700	NM_024697	upstream	398270	Hs.21026	ZNF385D
rs7838730	8	70637862	0.1367	93.68%	0.297700	0.297000	0.00086580	0.00557000	NM_015170	intron	0	Hs.409602	SULF1
rs1476742	5	81756346	0.1218	95.09%	0.567700	0.624300	0.00086870	0.00814600	NM_001039779	downstream	38255	---	FLJ41309
rs720763	16	9473575	0.2313	94.04%	0.605900	0.803600	0.00090170	0.01269000	ENST00000363598	downstream	92173	---	---
rs1953901	14	48134332	0.0545	93.33%	0.553700	1.000000	0.00091490	0.01018000	ENST00000384636	upstream	254047	---	---
rs1596839	11	25138024	0.1540	78.60%	0.000009	0.001251	0.00092450	0.02686000	NM_001009909	downstream	81244	Hs.144138	LUZP2
rs8092610	18	64612870	0.0215	97.89%	0.113800	1.000000	0.00093540	0.00961000	NM_024781	upstream	3480	Hs.280781	CCDC102B
rs10515167	5	73616240	0.3346	91.23%	0.166600	0.195800	0.00093570	0.00082770	ENST00000388185	upstream	33327	---	---
rs10507834	13	75274475	0.0287	97.89%	1.000000	1.000000	0.00094960	0.99740000	NM_005358	intron	0	Hs.207631	LMO7
rs1812535	8	62021574	0.0941	95.09%	1.000000	1.000000	0.00095670	0.00108900	NM_017780	downstream	79557	Hs.20395	CHD7
rs10499097	6	120580712	0.3297	97.89%	0.417600	0.853000	0.00098340	0.00193700	NM_005907	upstream	868087	Hs.102788	MAN1A1
rs2505593	10	36903981	0.4980	88.07%	0.000083	0.007186	0.00099270	0.03202000	ENST00000279434	upstream	50813	---	---
rs10508275	10	4105624	0.0609	95.09%	1.000000	1.000000	0.00103600	0.01140000	NM_001300	upstream	288157	Hs.4055	KLF6
rs1834619	2	17764966	0.3409	92.63%	0.683500	0.711500	0.00104700	0.00327200	NM_024624	intron	0	Hs.526728	SMC6
rs1323114	13	52408115	0.3436	96.49%	0.005066	0.017950	0.00104900	0.00515700	NM_032949	upstream	87543	Hs.19492	PCDH8
rs10496888	2	142087259	0.0311	90.18%	0.000000	0.000000	0.00105200	0.99810000	NM_018557	intron	0	Hs.656461	LRP1B
rs7580890	2	143039062	0.0771	93.33%	0.658600	1.000000	0.00107400	0.00313600	NM_001032998	upstream	312603	Hs.470126	KYNU
rs256817	5	123644632	0.1486	90.88%	0.468500	0.697600	0.00108800	0.02230000	NM_020747	downstream	355877	Hs.266616	ZNF608
rs6744763	2	79078334	0.0447	90.18%	0.080800	0.143800	0.00112000	0.00973700	NM_198448	upstream	28000	Hs.447084	REG3G
rs578813	11	41087368	0.0704	94.74%	0.628100	0.662700	0.00112300	0.00890700	ENST00000386948	downstream	13677	---	---
rs10489686	1	191250682	0.0431	93.68%	0.075490	0.134200	0.00113200	0.01115000	NM_015984	downstream	830	Hs.591458	UCHL5
rs2701105	12	113182071	0.3459	97.89%	0.145700	0.280600	0.00114700	0.00084950	NM_181486	downstream	94048	Hs.381715	TBX5
rs841576	1	43213063	0.4721	75.44%	0.002582	0.224200	0.00114700	0.00151700	NM_006516	upstream	15975	Hs.473721	SLC2A1
rs1892415	10	84172829	0.3582	96.49%	0.793900	0.286500	0.00115200	0.01318000	NM_001010848	intron	0	Hs.125119	NRG3
rs7357341	7	136341993	0.2847	96.14%	0.377200	0.735500	0.00116200	0.00364700	NM_001006629	intron	0	Hs.535891	CHRM2
rs10495673	2	18004709	0.2870	97.19%	0.187300	0.063440	0.00117600	0.00059540	NM_002252	downstream	27000	Hs.414489	KCNS3
rs10515380	5	107187134	0.1498	97.19%	0.233100	0.210500	0.00119500	0.00076260	NM_022824	downstream	38186	Hs.657225	FBXL17
rs10508749	10	30294794	0.0168	94.04%	1.000000	1.000000	0.00119900	0.00983900	NM_021738	upstream	330887	Hs.499209	SVIL
rs7073175	10	84148403	0.3698	84.91%	0.890200	0.457400	0.00122000	0.01927000	NM_001010848	intron	0	Hs.125119	NRG3
rs2031175	9	639750	0.3193	87.37%	0.189600	0.316800	0.00122800	0.00439500	NM_015158	intron	0	Hs.306764	ANKRD15
rs1353548	11	91409927	0.0256	95.79%	0.157100	1.000000	0.00123300	0.00828300	NM_012124	upstream	1814100	Hs.22857	CHORDC1
rs2373792	2	40203775	0.4257	87.37%	0.697200	0.636400	0.00123300	0.00736900	NM_021097	intron	0	Hs.468274	SLC8A1
rs2970925	2	88162851	0.1372	97.19%	0.616100	0.393400	0.00123400	0.00684100	NM_198274	intron	0	Hs.516176	SMYD1
rs940382	7	142424616	0.1047	90.53%	0.747200	1.000000	0.00127600	0.00413200	NM_001001658	downstream	8793	Hs.553594	OR9A2

rs1364593	3	190712898	0.3190	97.89%	0.890500	0.262300	0.00128900	0.00126200	NM_198485	downstream	188934	Hs.338851	FAM79B
rs3101644	15	25441728	0.3529	89.47%	0.013310	0.054440	0.00129900	0.00445800	NM_033223	intron	0	Hs.569475	GABRG3
rs2861215	2	77900300	0.3429	85.96%	0.001744	0.021210	0.00130500	0.01222000	ENST00000386159	downstream	151129	---	---
rs512533	4	177886641	0.0188	83.86%	0.001645	1.000000	0.00130900	0.04114000	NM_005429	intron	0	Hs.435215	VEGFC
rs10490678	2	36351175	0.1447	95.79%	0.323000	0.119200	0.00131700	0.01605000	NM_016441	upstream	85398	Hs.699247	CRIM1
rs1267622	7	140174786	0.2228	96.84%	0.485700	0.847700	0.00132100	0.01644000	NM_004333	intron	0	Hs.550061	BRAF
rs859365	1	173763324	0.0756	90.53%	0.164000	1.000000	0.00136400	0.00159100	ENST00000367674	intron	0	---	---
rs7974710	12	108158980	0.1709	82.11%	1.000000	1.000000	0.00137300	0.00255500	NM_001093	intron	0	Hs.234898	ACACB
rs1385982	8	50930576	0.0948	81.40%	0.702300	0.420600	0.00140200	0.00297200	NM_018967	upstream	538750	Hs.584914	SNTG1
rs10516104	5	173082405	0.0906	92.98%	0.011710	0.449500	0.00140600	0.05148000	NM_138369	upstream	106143	Hs.425091	FAM44B
rs10487731	7	31663909	0.0114	92.28%	1.000000	1.000000	0.00141900	0.99830000	NM_194300	downstream	4081	Hs.224269	CCDC129
rs291875	5	102866317	0.1541	97.89%	0.819300	1.000000	0.00145200	0.00463300	NM_031438	downstream	46138	Hs.434289	NUDT12
rs327866	5	125246105	0.4946	97.54%	0.811100	0.648500	0.00148100	0.00125800	NM_023927	upstream	540895	Hs.363558	GRAMD3
rs10510638	3	30751270	0.0191	82.46%	0.075050	1.000000	0.00148800	0.03647000	NM_207359	intron	0	Hs.657052	GADL1
rs10492610	13	31226328	0.2365	91.23%	0.124300	0.465500	0.00149300	0.01374000	NM_130806	intron	0	Hs.680763	RXFP2
rs9320162	6	106958006	0.2790	96.84%	0.052390	0.038730	0.00149500	0.00256100	NM_004849	upstream	77647	Hs.486063	ATG5
rs10508240	10	2719953	0.2992	89.12%	0.133600	0.308000	0.00151000	0.01022000	NM_002627	upstream	379759	Hs.26010	PFKP
rs725463	15	57930371	0.2892	94.04%	0.298600	0.401500	0.00153000	0.01172000	NM_012182	upstream	154056	Hs.160375	FOXB1
rs10504861	8	89617048	0.1444	97.19%	0.139400	0.303900	0.00154800	0.01111000	NM_003821	upstream	1222125	Hs.103755	RIPK2
rs2117784	3	6320015	0.2583	84.91%	0.063390	0.058340	0.00156300	0.00516000	NM_181874	upstream	558061	Hs.660131	GRM7
rs2376310	9	2111124	0.1861	93.33%	0.689300	0.760800	0.00156700	0.00301000	NM_003070	intron	0	Hs.298990	SMARCA2
rs10499143	6	129258873	0.0205	94.04%	1.000000	1.000000	0.00157200	0.00053260	NM_000426	intron	0	Hs.200841	LAMA2
rs229054	21	27238687	0.1900	97.89%	0.559000	0.674200	0.00157200	0.01329000	NM_007038	intron	0	Hs.58324	ADAMTS5
rs9290870	3	189339096	0.1863	95.09%	0.840900	1.000000	0.00160500	0.00097860	NM_207488	upstream	39929	---	FLJ42393
rs348561	5	40348149	0.2105	86.67%	0.022260	0.158200	0.00161100	0.00353100	ENST00000362491	downstream	42571	---	---
rs7118840	11	123605226	0.4200	96.49%	0.901900	0.519700	0.00161200	0.00086510	NM_001007249	downstream	3651	Hs.381319	OR8G2
rs10510244	3	3011671	0.0117	90.18%	1.000000	1.000000	0.00163100	0.99810000	NM_175613	intron	0	Hs.298705	CNTN4
rs1323113	13	52407476	0.1942	97.54%	0.085630	0.039850	0.00163800	0.00225700	NM_032949	upstream	86904	Hs.19492	PCDH8
rs1496103	1	185526018	0.3000	75.44%	0.005681	0.205100	0.00164100	0.00147800	NM_024420	downstream	301282	Hs.497200	PLA2G4A
rs4077557	2	22490359	0.1630	96.84%	0.045990	0.735200	0.00166300	0.00530000	ENST00000384155	upstream	74979	---	---
rs4881205	10	3743307	0.5000	95.44%	0.146800	0.444400	0.00169800	0.00567300	NM_001300	downstream	64881	Hs.4055	KLF6
rs1031159	3	125418519	0.0118	89.47%	0.000174	1.000000	0.00171000	0.99820000	NM_003947	intron	0	Hs.8004	KALRN
rs1994497	7	142527049	0.1063	94.04%	0.749500	0.746100	0.00172100	0.00740100	NM_002652	upstream	12239	Hs.99949	PIP
rs9306902	2	16913600	0.4074	94.74%	0.449600	0.743800	0.00172600	0.00978800	ENST00000386331	downstream	13844	---	---
rs2346593	4	63606400	0.0849	95.09%	0.420300	0.431600	0.00172800	0.00522000	NM_001010874	downstream	1221985	Hs.227752	SRD5A2L2
rs9309473	2	73597490	0.2097	93.68%	0.063130	0.010670	0.00174600	0.00275300	NM_015120	intron	0	Hs.184720	ALMS1
rs1578243	1	191503664	0.3759	96.14%	0.700800	0.645900	0.00175100	0.00281400	NM_024529	downstream	15985	Hs.576497	CDC73
rs3098224	8	104515872	0.1951	92.63%	0.695900	1.000000	0.00175700	0.00056130	NM_015420	intron	0	Hs.532265	WDSOF1
rs4355015	18	36966697	0.0919	95.44%	1.000000	1.000000	0.00176100	0.01470000	NM_002647	upstream	822500	Hs.464971	PIK3C3
rs1854226	13	97036243	0.1942	97.54%	0.338500	0.302400	0.00176200	0.01091000	NM_021033	downstream	118000	Hs.508480	RAP2A
rs10483927	14	79147362	0.0324	97.54%	0.247400	1.000000	0.00177500	0.00771100	NM_138970	intron	0	Hs.368307	NRXN3
rs7896207	10	19111041	0.1111	97.89%	0.759000	0.604300	0.00178400	0.00545300	NM_178815	downstream	104095	Hs.25362	ARL5B
rs10492329	12	113032591	0.2556	94.74%	0.633900	0.652600	0.00178600	0.00544900	NM_016196	upstream	144097	Hs.7482	RBM19
rs10505302	8	118479807	0.1960	97.54%	0.125200	0.210500	0.00179900	0.00729900	NM_080651	upstream	122404	Hs.492612	MED30
rs10492354	12	31248369	0.1175	94.04%	1.000000	1.000000	0.00180500	0.00838000	NM_021238	downstream	76427	Hs.505154	FAM60A
rs10495744	2	23432076	0.3403	92.28%	0.783700	0.855200	0.00181900	0.00203800	ENST00000288548	upstream	29999	---	---
rs422467	8	37251453	0.4700	81.75%	0.292700	0.192600	0.00181900	0.02266000	NM_021631	downstream	385467	Hs.651853	FKSG2
rs10484314	6	5593086	0.4277	87.37%	0.028350	0.235300	0.00182600	0.01746000	NM_006567	intron	0	Hs.484547	FARS2
rs925806	2	183704326	0.2978	95.44%	0.013180	0.239900	0.00182700	0.00803300	NM_001008544	intron	0	---	NUP35
rs10491374	5	36703336	0.4234	91.58%	0.022260	0.214600	0.00182800	0.00683100	NM_004172	intron	0	Hs.481918	SLC1A3
rs10504994	8	100694363	0.2892	94.04%	0.298600	0.067830	0.00185700	0.01876000	NM_017890	intron	0	Hs.191540	VPS13B
rs763970	2	138352603	0.2500	95.44%	0.107000	0.037480	0.00186600	0.00039980	NM_001024075	upstream	85675	Hs.42151	HNMT
rs959036	3	95660973	0.4347	94.04%	0.387300	0.873200	0.00187200	0.03847000	NM_022072	downstream	332653	Hs.656338	NSUN3
rs692419	5	18024601	0.4658	97.54%	0.717800	0.878100	0.00191000	0.00393900	ENST00000332101	upstream	336205	---	---

rs569724	11	29979751	0.1679	97.19%	1.000000	1.000000	0.00191400	0.00578300	NM_002233	downstream	8590	Hs.592002	KCNA4
rs763829	2	125916736	0.1556	94.74%	0.107000	0.458200	0.00193400	0.00747000	NM_016815	upstream	1247808	Hs.59138	GYPC
rs13183130	5	3662953	0.0278	94.74%	1.000000	1.000000	0.00193600	0.99720000	NM_024337	downstream	8436	Hs.424156	IRX1
rs4488182	11	41032137	0.2007	97.89%	0.136900	0.220900	0.00194200	0.00102200	ENST00000386948	downstream	68908	---	---
rs7792319	7	19133537	0.1679	97.19%	0.082880	0.039030	0.00194900	0.02219000	NM_000474	upstream	9717	Hs.66744	TWIST1
rs950519	20	22410490	0.0387	95.09%	0.331500	0.431600	0.00195500	0.03015000	NM_153675	downstream	99333	Hs.155651	FOXA2
rs173851	5	86041120	0.0430	97.89%	1.000000	1.000000	0.00195800	0.00345400	NM_001039781	downstream	340399	---	LOC645261
rs10484015	14	89233314	0.3209	89.12%	0.312700	0.617400	0.00196800	0.00344400	ENST00000363442	upstream	15294	---	---
rs10497961	2	212751316	0.0295	95.09%	0.017240	0.038370	0.00197000	0.99740000	NM_001042599	intron	0	Hs.390729	ERBB4
rs10506330	12	53377548	0.0295	95.09%	1.000000	1.000000	0.00197000	0.99730000	NM_053283	upstream	49135	Hs.350570	DCD
rs285406	8	87094129	0.0296	94.74%	0.204500	0.298600	0.00197100	0.99720000	NM_033126	downstream	35678	Hs.680136	PSKH2
rs7210646	17	53194185	0.0277	95.09%	1.000000	1.000000	0.00197100	0.99700000	ENST00000299415	upstream	16553	---	---
rs993873	7	48964209	0.4882	89.12%	0.260500	0.642300	0.00198600	0.00658500	NM_198570	upstream	819589	Hs.677488	VWC2
rs2932887	10	66330005	0.0611	94.74%	0.255300	1.000000	0.00201000	0.00302300	NM_013266	downstream	1019720	Hs.660362	CTNNA3
rs993870	7	48964571	0.4851	94.39%	0.902900	0.763600	0.00202900	0.00569000	NM_198570	upstream	819227	Hs.677488	VWC2
rs1451424	4	180297133	0.4777	94.39%	0.066920	1.000000	0.00204100	0.01875000	ENST00000364857	downstream	732175	---	---
rs733207	6	157835675	0.1956	95.09%	0.442600	0.674200	0.00205300	0.00318700	NM_153746	intron	0	Hs.187459	ZDHHC14
rs2151933	10	99435896	0.0675	88.42%	0.089560	0.024560	0.00208600	0.00573400	NM_021732	intron	0	Hs.23918	AVPI1
rs10488948	4	10145642	0.3007	95.09%	0.195100	0.173500	0.00216300	0.01899000	NM_052964	intron	0	Hs.678910	MIST
rs952841	5	86135888	0.1882	97.89%	0.845900	0.830500	0.00219100	0.00885300	NM_001039781	downstream	245631	---	LOC645261
rs2098067	4	11212565	0.4760	95.09%	0.543300	1.000000	0.00219800	0.00535800	NM_005114	upstream	172078	Hs.507348	HS3ST1
rs2001776	9	2110825	0.1739	96.84%	0.527900	0.748800	0.00222100	0.00435000	NM_003070	intron	0	Hs.298990	SMARCA2
rs10521030	16	50046243	0.0371	89.82%	0.038880	0.044110	0.00222300	0.00236300	NM_002968	upstream	303590	Hs.135787	SALL1
rs725779	12	1999243	0.2008	92.63%	0.052760	0.397200	0.00222600	0.00727500	NM_152640	upstream	15305	Hs.130934	DCP1B
rs845105	10	125171849	0.0450	97.54%	0.431400	0.529800	0.00223400	0.01966000	NM_153442	upstream	244012	Hs.12751	GPR26
rs10507085	12	96310831	0.0463	94.74%	0.013430	1.000000	0.00223500	0.06997000	ENST00000384854	upstream	170890	---	---
rs10513351	9	121566595	0.3646	84.21%	0.210100	0.089220	0.00225800	0.01111000	NM_014618	upstream	395073	Hs.532316	DBC1
rs963730	2	39070277	0.1412	91.93%	0.317700	0.604600	0.00227900	0.00462300	NM_005633	intron	0	Hs.654397	SOS1
rs699549	2	4683138	0.0685	94.74%	0.355200	0.270200	0.00231300	0.00034860	ENST00000387792	downstream	169328	---	---
rs612815	3	195075371	0.2870	97.19%	0.768900	0.614500	0.00231500	0.01867000	NM_005524	upstream	261257	Hs.250666	HES1
rs10504993	8	100678473	0.2782	87.02%	0.038880	0.006530	0.00231700	0.01239000	NM_017890	intron	0	Hs.191540	VPS13B
rs243030	2	60460075	0.2578	90.53%	0.005293	0.015540	0.00232100	0.01001000	ENST00000363937	upstream	5169	---	---
rs2346645	4	63565257	0.0921	93.33%	0.472900	0.505200	0.00232700	0.00959600	NM_001010874	downstream	1263128	Hs.227752	SRD5A2L2
rs254755	5	86034265	0.0394	97.89%	1.000000	1.000000	0.00232700	0.00569200	NM_001039781	downstream	347254	---	LOC645261
rs953944	7	77846067	0.1945	96.49%	0.702000	1.000000	0.00233400	0.00692300	NM_012301	intron	0	Hs.654788	MAGI2
rs496112	6	12731836	0.3750	92.63%	0.693400	0.395100	0.00235900	0.00148500	NM_030948	upstream	95127	Hs.436996	PHACTR1
rs2199297	8	113106899	0.1337	90.53%	0.001685	0.001331	0.00236700	0.03284000	ENST00000364160	downstream	123197	---	---
rs1923402	10	4150469	0.0665	97.54%	1.000000	1.000000	0.00240200	0.03424000	NM_001300	upstream	333002	Hs.4055	KLF6
rs10487391	7	117652264	0.1643	73.68%	0.079110	0.217900	0.00240900	0.00725100	NM_019644	5UTR	0	Hs.657737	ANKRD7
rs4667235	2	189228047	0.2043	82.46%	0.002037	0.001806	0.00241000	0.00317200	NM_016315	downstream	60016	Hs.470887	GULP1
rs996893	7	77844100	0.1964	96.49%	0.702000	1.000000	0.00241600	0.00611700	NM_012301	intron	0	Hs.654788	MAGI2
rs3901494	11	123740882	0.4417	93.33%	1.000000	0.634600	0.00242300	0.00128700	ENST00000359473	CDS	0	---	---
rs1415472	9	16631492	0.0701	95.09%	0.378100	1.000000	0.00243600	0.00093690	NM_017637	intron	0	Hs.656581	BNC2
rs6736638	2	64806834	0.0686	97.19%	0.029080	1.000000	0.00244700	0.01213000	NM_014755	upstream	72284	Hs.693696	SERTAD2
rs7567009	2	143039231	0.0837	90.18%	0.398600	0.423000	0.00245900	0.00395900	NM_001032998	upstream	312434	Hs.470126	KYNU
rs10495725	2	22076169	0.0867	95.09%	0.704100	1.000000	0.00246100	0.00958000	ENST00000383859	downstream	76504	---	---
rs10501940	11	99440078	0.1143	90.53%	0.348100	0.289300	0.00247500	0.00085040	NM_175566	intron	0	Hs.656783	CNTN5
rs1417694	6	63800297	0.3809	97.19%	0.160500	0.066180	0.00251600	0.00144200	ENST00000370659	upstream	179013	---	---
rs10492839	16	6685600	0.0525	96.84%	0.539700	1.000000	0.00251900	0.03456000	NM_001013705	downstream	315917	---	LOC440337
rs10490208	2	40307298	0.4904	91.58%	0.215400	0.876800	0.00253400	0.01208000	NM_021097	intron	0	Hs.468274	SLC8A1
rs851426	2	19356456	0.2158	97.54%	1.000000	0.693100	0.00254400	0.01739000	NM_145260	downstream	58271	Hs.123933	OSR1
rs10488388	7	130552209	0.0773	97.54%	1.000000	1.000000	0.00255700	0.00378900	NM_013255	upstream	110966	Hs.702060	MKLN1
rs1893290	18	19696619	0.2227	80.35%	0.000000	0.000000	0.00256200	0.06267000	NM_198129	intron	0	Hs.436367	LAMA3
rs609493	11	93708735	0.3657	94.04%	0.187800	0.279400	0.00259000	0.01850000	NM_016540	downstream	41396	Hs.272385	GPR83

rs6549162	3	69117389	0.1596	91.23%	0.252200	1.000000	0.00266600	0.02518000	NM_173654	intron	0	Hs.518059	C3orf64
rs10508567	10	19061393	0.1093	97.89%	0.755200	0.604300	0.00269900	0.00820200	NM_178815	downstream	54447	Hs.25362	ARL5B
rs1591817	10	109128388	0.3549	78.60%	0.002059	0.033490	0.00269900	0.01367000	NM_001013031	upstream	214114	Hs.591915	SORCS1
rs6733858	2	190172981	0.3727	95.09%	0.698900	0.643800	0.00270200	0.00548800	NM_014585	upstream	19123	Hs.643005	SLC40A1
rs980286	2	141447731	0.2126	86.67%	0.001869	0.001620	0.00271400	0.01221000	NM_018557	intron	0	Hs.656461	LRP1B
rs34460	12	95708104	0.1615	91.23%	0.645100	0.295200	0.00275700	0.03387000	NM_198520	downstream	24925	Hs.436197	C12orf63
rs10495743	2	23431463	0.3504	85.61%	0.092170	0.039430	0.00276100	0.01252000	ENST00000288548	upstream	30612	---	---
rs10513380	3	152126063	0.4240	87.72%	0.000752	0.016480	0.00276600	0.01134000	NM_052995	downstream	577	Hs.380222	CLRN1
rs10491242	5	81776991	0.0498	95.09%	0.494000	0.121800	0.00277400	0.01453000	NM_001039779	downstream	58900	---	FLJ41309
rs10510779	3	56086063	0.1125	95.09%	0.758200	1.000000	0.00278300	0.01135000	NM_015576	intron	0	Hs.476389	ERC2
rs956380	3	19346049	0.0556	94.74%	0.573700	1.000000	0.00279500	0.09881000	NM_144633	intron	0	Hs.475656	KCNH8
rs1395612	4	189670284	0.2754	96.84%	0.651500	0.673300	0.00281200	0.00619800	NM_178556	downstream	364641	Hs.348618	TRIML1
rs1531209	8	95306201	0.3387	97.89%	0.182800	0.589000	0.00283300	0.00225300	NM_004063	upstream	16215	Hs.591853	CDH17
rs7649544	3	353069	0.0699	97.89%	0.377500	1.000000	0.00283700	0.00206000	NM_006614	intron	0	Hs.148909	CHL1
rs2036516	9	97170923	0.3939	81.05%	0.000909	0.067860	0.00285200	0.03000000	NM_000136	upstream	51111	Hs.494529	FANCC
rs236833	2	205517999	0.4748	90.53%	0.169200	0.754600	0.00287800	0.00398700	NM_057177	intron	0	Hs.657382	PARD3B
rs10497682	2	189391807	0.0933	94.04%	0.712200	1.000000	0.00288900	0.01474000	NM_052952	upstream	28731	Hs.470892	DIRC1
rs1835705	4	133133982	0.0504	94.04%	1.000000	1.000000	0.00290500	0.00168000	NM_020815	upstream	1155938	Hs.192859	PCDH10
rs994705	4	42658294	0.1306	94.04%	0.098710	0.169500	0.00291200	0.00552500	NM_006095	upstream	304427	Hs.435052	ATP8A1
rs6990997	8	106881703	0.0794	81.75%	0.008105	0.014930	0.00291400	0.05485000	NM_012082	intron	0	Hs.431009	ZFPM2
rs1592418	7	34877868	0.3098	96.84%	0.122500	0.331000	0.00292600	0.00182300	NM_207173	intron	0	Hs.652373	NPSR1
rs1864747	14	88044620	0.1798	80.00%	0.001260	0.007693	0.00293200	0.01904000	NM_007039	intron	0	Hs.437040	PTPN21
rs3754741	2	173761465	0.2658	94.39%	0.350000	0.379400	0.00293700	0.01628000	NM_133646	intron	0	Hs.444451	ZAK
rs726789	19	14604003	0.3448	97.19%	0.790600	0.269200	0.00294700	0.00241100	NM_152939	intron	0	---	EMR3
rs724821	6	11920451	0.1208	92.98%	0.035250	0.136200	0.00297000	0.05006000	NM_032744	upstream	33399	Hs.126409	C6orf105
rs1909650	13	52689896	0.2068	67.02%	0.001405	0.000290	0.00298200	0.00392500	NM_006418	downstream	165709	Hs.559736	OLFM4
rs7296881	12	24985803	0.1781	76.84%	1.000000	1.000000	0.00298200	0.05271000	NM_005504	upstream	39711	Hs.438993	BCAT1
rs6986526	8	27325088	0.0164	85.61%	0.056610	1.000000	0.00298300	0.07008000	NM_173175	intron	0	Hs.491322	PTK2B
rs7777578	7	145307815	0.1688	81.05%	1.000000	1.000000	0.00299100	0.00057280	ENST00000385572	upstream	17539	---	---
rs7222397	17	65352438	0.1887	90.18%	0.105600	0.130300	0.00299600	0.03537000	NM_002758	downstream	301371	Hs.463978	MAP2K6
rs7144210	14	62940684	0.3393	88.42%	0.001125	0.001773	0.00299900	0.01053000	NM_006246	intron	0	Hs.334868	PPP2R5E
rs2421857	5	160352943	0.4812	83.86%	0.069990	0.261700	0.00300100	0.00108300	NM_000813	downstream	300506	Hs.303527	GABRB2
rs2320760	13	32379793	0.1523	97.89%	0.485400	0.474800	0.00302600	0.00889700	NM_015032	downstream	129636	Hs.699308	PDS5B
rs10508247	10	3171577	0.2731	87.37%	0.427400	0.265500	0.00305900	0.00550000	NM_014889	intron	0	Hs.528300	PITRM1
rs10483831	14	70130397	0.2172	93.68%	0.858000	0.793000	0.00309200	0.02216000	NM_005466	intron	0	Hs.497353	MED6
rs1920309	3	123148169	0.3628	97.19%	0.896900	0.756900	0.00309600	0.00289900	NM_021082	downstream	5022	Hs.518089	SLC15A2
rs4653643	1	223742082	0.4176	93.68%	0.614600	0.291200	0.00309900	0.00707400	NM_018212	3UTR	0	Hs.497893	ENAH
rs2593105	10	55337841	0.1816	93.68%	0.541800	0.381000	0.00311200	0.02568000	NM_033056	intron	0	Hs.672170	PCDH15
rs261004	5	169269201	0.4473	96.49%	0.626900	0.880500	0.00312600	0.01998000	NM_004946	intron	0	Hs.586174	DOCK2
rs10491249	5	126909947	0.0789	97.89%	0.682000	1.000000	0.00315600	0.01030000	NM_130809	intron	0	Hs.483259	PRRC1
rs10258159	7	9222688	0.0627	97.89%	0.081060	1.000000	0.00315900	0.01160000	NM_152745	downstream	464764	Hs.487564	NXPH1
rs10507372	13	26221607	0.0320	93.33%	0.231900	1.000000	0.00315900	0.00533500	NM_005288	downstream	8840	Hs.123034	GPR12
rs784205	13	101642950	0.1254	97.89%	0.591200	0.577400	0.00316000	0.00133300	NM_175929	intron	0	Hs.696392	FGF14
rs10497247	2	164616298	0.1981	94.74%	0.122600	0.091450	0.00316100	0.04815000	NM_018086	upstream	315539	Hs.593650	FIGN
rs10489305	1	175233447	0.2271	91.93%	0.033030	0.132600	0.00316200	0.00623400	NM_207108	intron	0	Hs.495897	ASTN1
rs1404807	7	145408598	0.1090	93.33%	0.530000	0.746900	0.00317300	0.01065000	NM_014141	upstream	2258914	Hs.655684	CNTNAP2
rs10495012	1	214165591	0.3590	93.33%	0.790700	0.598500	0.00318200	0.00385100	NM_206933	intron	0	Hs.655974	USH2A
rs7810561	7	76741153	0.0370	94.74%	0.043170	0.076550	0.00318800	0.07001000	NM_020879	intron	0	Hs.113940	CCDC146
rs12609097	19	22565844	0.1157	89.47%	0.002252	0.054170	0.00319400	0.00634700	ENST00000387067	upstream	10303	---	---
rs1986446	11	97304031	0.1703	97.89%	0.286500	0.208600	0.00319800	0.00233900	ENST00000384812	downstream	14932	---	---
rs10497542	2	180150259	0.2115	91.23%	1.000000	1.000000	0.00320400	0.00404900	NM_152520	intron	0	Hs.655005	ZNF385B
rs2710540	4	126491349	0.2751	94.39%	0.284300	0.833900	0.00320900	0.01651000	ENST00000364427	upstream	12075	---	---
rs1559990	10	79800598	0.4523	77.19%	0.000043	0.002929	0.00321800	0.00537200	ENST00000364523	downstream	3212	---	---
rs1356265	12	37689963	0.3714	96.84%	0.020850	0.085320	0.00324200	0.01146000	NM_153634	upstream	104263	Hs.40910	CPNE8

rs1370005	8	104514185	0.2019	94.74%	0.706000	0.788700	0.00324800	0.00098470	NM_015420	intron	0	Hs.532265	WDSOF1
rs10495266	1	225313639	0.1005	73.33%	0.008913	0.058520	0.00325300	0.00335500	NM_003607	intron	0	Hs.35433	CDC42BPA
rs7329659	13	97872564	0.4297	92.28%	0.531300	0.871400	0.00325400	0.00506500	NM_005766	intron	0	Hs.403917	FARP1
rs10488574	7	75761928	0.2646	96.14%	0.876500	0.517400	0.00329700	0.00099530	NM_001039212	downstream	7389	---	FLJ37078
rs10495866	2	36158207	0.0668	86.67%	1.000000	1.000000	0.00329700	0.03991000	NM_016441	upstream	278366	Hs.699247	CRIM1
rs1109786	7	66696731	0.1547	97.54%	0.256800	0.805300	0.00330000	0.00912500	ENST00000388509	upstream	194678	---	---
rs951240	1	153060387	0.1155	97.19%	0.033290	0.615500	0.00330100	0.00204500	NM_002249	intron	0	Hs.490765	KCNN3
rs3766587	1	99928777	0.0286	85.96%	0.000373	0.001297	0.00330600	0.99720000	NM_017734	intron	0	Hs.483993	PALMD
rs998225	15	70544251	0.0615	91.23%	1.000000	0.210800	0.00332500	0.00343900	NM_005744	upstream	9784	Hs.268787	ARIH1
rs10506326	12	52577033	0.1558	96.84%	0.820900	1.000000	0.00332900	0.00430500	NM_017410	upstream	41810	Hs.118608	HOXC13
rs3803261	13	52511082	0.2591	96.84%	0.639200	1.000000	0.00333000	0.01214000	NM_006418	intron	0	Hs.559736	OLFM4
rs10503328	8	5527286	0.0258	95.09%	0.158200	0.233400	0.00333300	0.99740000	ENST00000363751	downstream	228560	---	---
rs7323534	13	36948451	0.2688	88.77%	0.016010	0.262100	0.00333300	0.00136200	NM_006475	downstream	86328	Hs.136348	POSTN
rs9315495	13	36985145	0.3814	96.14%	0.124400	0.129100	0.00334200	0.02719000	NM_006475	downstream	49581	Hs.136348	POSTN
rs6790281	3	123148233	0.3656	97.89%	0.897300	0.642800	0.00334800	0.00273500	NM_021082	downstream	5086	Hs.518089	SLC15A2
rs1029819	7	77847101	0.1967	95.44%	0.701400	1.000000	0.00338000	0.00973900	NM_012301	intron	0	Hs.654788	MAGI2
rs1920241	3	190776853	0.2590	97.54%	0.754300	0.511700	0.00338500	0.00077970	NM_198485	downstream	252889	Hs.338851	FAM79B
rs2542930	2	211416575	0.2537	94.04%	0.522300	0.500500	0.00339100	0.00094320	NM_001875	downstream	164501	Hs.149252	CPS1
rs1363262	5	85471132	0.1333	94.74%	0.594900	0.788700	0.00339200	0.02370000	ENST00000380134	upstream	142886	---	---
rs4450000	1	191358607	0.3741	97.54%	1.000000	0.759100	0.00341500	0.00558100	NM_024529	intron	0	Hs.576497	CDC73
rs10512574	17	65849559	0.2846	91.23%	0.021520	0.288100	0.00345000	0.03284000	NM_000891	downstream	161804	Hs.1547	KCNJ2
rs2068338	7	77846922	0.1953	96.14%	0.701700	1.000000	0.00347600	0.01259000	NM_012301	intron	0	Hs.654788	MAGI2
rs10489313	1	173792149	0.0627	89.47%	0.253200	1.000000	0.00348500	0.00103800	ENST00000367674	intron	0	---	---
rs10506202	12	40307615	0.3662	94.39%	1.000000	0.603400	0.00349100	0.01514000	NM_013377	downstream	52964	Hs.380044	PDZRN4
rs7008914	8	25936317	0.2870	94.74%	0.052450	0.233400	0.00351800	0.05272000	ENST00000380737	intron	0	---	---
rs710768	12	68276718	0.2122	97.54%	0.371100	1.000000	0.00352000	0.02547000	NM_006431	intron	0	Hs.189772	CCT2
rs3803260	13	52511126	0.2427	96.14%	0.328100	0.644200	0.00352700	0.00860000	NM_006418	intron	0	Hs.559736	OLFM4
rs10485819	20	169018	0.0774	92.98%	1.000000	1.000000	0.00353500	0.00343300	NM_080831	downstream	10491	Hs.112087	DEFB129
rs10506203	12	40308117	0.1111	94.74%	0.754700	1.000000	0.00356200	0.01357000	NM_013377	downstream	53466	Hs.380044	PDZRN4
rs10490727	2	10434959	0.1818	96.49%	0.023740	0.065200	0.00357400	0.00718700	NM_134421	intron	0	Hs.580427	HPCAL1
rs1885234	9	118292098	0.4468	92.28%	0.318100	0.519800	0.00360100	0.01052000	NM_198188	intron	0	Hs.653266	ASTN2
rs2029206	3	151324527	0.2011	95.09%	0.449500	0.676300	0.00360200	0.00120700	ENST00000386021	upstream	46219	---	---
rs10511710	9	22874562	0.1547	92.98%	0.056400	0.046480	0.00361200	0.00392000	NM_022160	downstream	432090	Hs.371976	DMRTA1
rs7857377	9	118832071	0.4392	89.47%	0.524700	0.872300	0.00363100	0.03620000	NM_198188	intron	0	Hs.653266	ASTN2
rs1566100	4	190265102	0.4963	94.04%	0.465100	0.650400	0.00365200	0.04354000	ENST00000378771	downstream	126898	---	---
rs10502760	18	37097536	0.0778	94.74%	0.666400	0.694400	0.00368500	0.01572000	NM_002647	upstream	691661	Hs.464971	PIK3C3
rs8124344	20	168757	0.0778	94.74%	1.000000	1.000000	0.00368500	0.00285400	NM_080831	downstream	10230	Hs.112087	DEFB129
rs1864746	14	88044552	0.1996	90.53%	0.170200	0.137200	0.00369200	0.02427000	NM_007039	intron	0	Hs.437040	PTPN21
rs1073061	12	52560676	0.1601	97.54%	1.000000	1.000000	0.00372300	0.00400900	NM_017410	upstream	58167	Hs.118608	HOXC13
rs10510718	3	41344071	0.0412	97.89%	1.000000	1.000000	0.00373100	0.00250600	ENST00000301831	intron	0	---	---
rs10508750	10	30294185	0.0145	96.49%	1.000000	1.000000	0.00378600	0.02087000	NM_021738	upstream	330278	Hs.499209	SVIL
rs7553933	1	110344608	0.0305	97.89%	1.000000	1.000000	0.00378600	0.00252600	NM_006621	intron	0	Hs.705418	AHCYL1
rs1331126	1	193242564	0.2368	93.33%	0.174800	0.812600	0.00379000	0.00396600	NM_198503	downstream	1218972	Hs.657046	KCNT2
rs9316069	13	43561145	0.1044	87.37%	1.000000	0.187500	0.00380600	0.00962800	ENST00000267129	downstream	71592	---	---
rs1429143	6	53372444	0.3032	97.19%	1.000000	0.323200	0.00381300	0.00754600	ENST00000388337	downstream	15779	---	---
rs698458	9	106273931	0.3303	95.09%	0.132800	0.364300	0.00382500	0.00441200	NM_001004485	upstream	32434	Hs.553602	OR13F1
rs1531208	8	95306174	0.3405	97.89%	0.142400	0.472300	0.00383100	0.00279100	NM_004063	upstream	16188	Hs.591853	CDH17
rs7531479	1	36902174	0.2622	93.68%	0.428600	0.125900	0.00384300	0.00633800	NM_000831	downstream	137027	Hs.654524	GRIK3
rs1224635	15	45906253	0.0593	94.74%	0.232200	0.300800	0.00384600	0.01064000	NM_024966	downstream	56217	Hs.511265	SEMA6D
rs1072385	6	144192302	0.1042	92.63%	0.329000	0.316400	0.00387700	0.01552000	NM_014721	downstream	3417	Hs.654921	PHACTR2
rs1788155	8	100867916	0.2915	95.09%	0.379400	0.159200	0.00387800	0.01581000	NM_017890	intron	0	Hs.191540	VPS13B
rs1351253	11	79732182	0.1165	97.89%	1.000000	0.127600	0.00388500	0.00075630	ENST00000363276	upstream	184470	---	---
rs198045	21	23450216	0.1240	86.32%	0.004990	0.004138	0.00388600	0.05158000	ENST00000364042	downstream	125749	---	---
rs2025804	1	65718709	0.4011	97.54%	0.534400	0.761600	0.00388800	0.01725000	NM_001003680	intron	0	Hs.705413	LEPR

rs4395341	22	21036563	0.0573	97.89%	1.000000	1.000000	0.00390200	0.00820800	ENST00000334392	upstream	907	---	---
rs10486618	7	33793938	0.2606	74.74%	0.021280	0.228000	0.00390500	0.01100000	ENST00000366310	upstream	18150	---	---
rs10512304	9	104487856	0.0524	93.68%	0.003075	0.006078	0.00393200	0.12960000	NM_001340	upstream	309558	Hs.3232	CYLC2
rs16971911	16	71984877	0.0316	94.39%	0.000000	0.000128	0.00394500	0.09154000	NM_207385	upstream	249030	---	C16orf47
rs10500270	19	39302438	0.2119	94.39%	0.028460	0.024560	0.00396500	0.00373200	NM_015578	upstream	52873	Hs.407368	LSM14A
rs1546792	3	106476224	0.3502	97.19%	0.292000	0.859800	0.00398600	0.00827300	NM_001627	upstream	92179	Hs.591293	ALCAM
rs7584596	2	174158643	0.1567	94.04%	0.250500	1.000000	0.00399900	0.04450000	ENST00000306716	upstream	99307	---	---
rs10499773	7	63477213	0.0456	92.28%	0.422700	1.000000	0.00401000	0.02937000	NM_178558	downstream	140488	Hs.520886	ZNF680
rs1586601	8	118487189	0.3429	91.58%	0.891600	1.000000	0.00401800	0.00445500	NM_080651	upstream	115022	Hs.492612	MED30
rs6686648	1	171213383	0.0465	94.39%	0.442600	0.536300	0.00405600	0.00945000	NM_005092	downstream	62340	Hs.248197	TNFSF18
rs4733653	8	129913853	0.3353	88.42%	0.573100	0.850600	0.00405800	0.00911500	ENST00000384930	upstream	12548	---	---
rs4770010	13	19840037	0.3496	93.33%	0.139400	0.113400	0.00406600	0.01033000	NM_015974	downstream	35773	Hs.370703	CRYL1
rs10501549	11	83230041	0.1075	97.89%	0.538400	0.286100	0.00408000	0.02212000	NM_001364	intron	0	Hs.654862	DLG2
rs10508566	10	19060072	0.1075	97.89%	0.538400	0.604300	0.00408000	0.01128000	NM_178815	downstream	53126	Hs.25362	ARL5B
rs10510781	3	56095537	0.1075	97.89%	0.538400	1.000000	0.00408000	0.01457000	NM_015576	intron	0	Hs.476389	ERC2
rs10521151	17	6468330	0.1297	93.33%	0.059050	1.000000	0.00408700	0.05561000	NM_014804	intron	0	Hs.28070	KIAA0753
rs1482089	4	86737021	0.4173	93.33%	0.165400	0.445600	0.00412200	0.01970000	NM_001025616	intron	0	Hs.444229	ARHGAP24
rs10495790	2	33572977	0.0616	94.04%	1.000000	1.000000	0.00414500	0.00822800	NM_170672	intron	0	Hs.143674	RASGRP3
rs10494100	1	109175124	0.0693	83.51%	0.017290	0.021680	0.00415100	0.00851300	NM_152763	intron	0	Hs.514662	C1orf62
rs724946	13	29575163	0.1697	95.09%	0.192700	0.239500	0.00415400	0.04088000	NM_001014380	downstream	99604	Hs.243596	KATNAL1
rs941458	6	126395967	0.3834	67.72%	0.170900	0.280700	0.00415900	0.01177000	NM_001031712	intron	0	Hs.404186	TRMT11
rs1823634	11	4397414	0.2518	96.14%	0.422500	0.823300	0.00419700	0.01676000	NM_003141	upstream	25912	Hs.532357	TRIM21
rs2631268	4	103167753	0.4089	94.39%	1.000000	0.253700	0.00420400	0.02611000	NM_017935	intron	0	Hs.480400	BANK1
rs10491514	9	128141839	0.3132	92.98%	0.886500	0.568000	0.00421300	0.12480000	NM_001011703	intron	0	Hs.162659	FAM125B
rs294975	5	73783037	0.4373	92.28%	0.802700	1.000000	0.00421300	0.01702000	NM_003633	downstream	175953	Hs.104925	ENC1
rs10514306	5	88696674	0.0591	97.89%	0.243000	1.000000	0.00422800	0.01090000	NM_002397	upstream	481856	Hs.699175	MEF2C
rs477184	20	13249361	0.4927	95.79%	0.275700	1.000000	0.00424700	0.03332000	NM_017714	downstream	68678	Hs.369932	TASP1
rs1010392	13	80764681	0.4185	94.74%	0.381400	1.000000	0.00426800	0.00147200	ENST00000387380	downstream	26729	---	---
rs314734	5	10085216	0.4386	97.19%	0.272100	1.000000	0.00428500	0.00130500	ENST00000358163	upstream	169405	---	---
rs7161750	15	50951074	0.0957	89.82%	0.263600	0.274900	0.00431700	0.04738000	NM_004498	upstream	81573	Hs.658573	ONECUT1
rs2250237	12	89207142	0.2180	87.72%	0.004641	0.039030	0.00432800	0.02338000	ENST00000388111	downstream	562212	---	---
rs1462111	5	152658514	0.1778	94.74%	0.208900	0.027580	0.00433700	0.00360700	NM_000827	upstream	191985	Hs.519693	GRIA1
rs1560048	5	91178732	0.0270	97.54%	1.000000	1.000000	0.00435300	0.00308000	NM_020801	upstream	463855	Hs.24684	ARRDC3
rs2058063	17	65669435	0.2284	97.54%	0.306600	0.701000	0.00438100	0.02752000	NM_000891	upstream	7836	Hs.1547	KCNJ2
rs268866	2	65344163	0.3509	92.98%	0.002856	0.024420	0.00438600	0.00335100	NM_005722	intron	0	Hs.699451	ACTR2
rs1351419	4	71235977	0.0221	95.09%	1.000000	1.000000	0.00441200	0.10300000	NM_033122	CDS	0	Hs.120316	C4orf35
rs2590713	12	52549809	0.1533	96.14%	0.815200	1.000000	0.00442100	0.00576700	NM_017410	upstream	69034	Hs.118608	HOXC13
rs1319309	11	7276742	0.4583	96.84%	0.903500	0.641500	0.00442700	0.00581300	NM_175733	intron	0	Hs.177193	SYT9
rs1516606	3	96503218	0.4754	92.63%	0.621400	0.447200	0.00442900	0.09270000	ENST00000332119	upstream	363660	---	---
rs2968392	5	22358920	0.0094	93.68%	1.000000	1.000000	0.00443300	0.99840000	NM_004061	intron	0	Hs.113684	CDH12
rs511189	3	96496203	0.4549	97.19%	0.716400	0.368100	0.00444000	0.07561000	ENST00000332119	upstream	356645	---	---
rs9323099	14	44019800	0.3195	93.33%	0.779800	0.346500	0.00444200	0.01708000	NM_032135	downstream	23663	Hs.307086	FSCB
rs925140	2	17226171	0.3656	97.89%	0.897300	0.732000	0.00444400	0.01235000	NM_003385	upstream	359118	Hs.444212	VSNL1
rs2731938	3	21371100	0.3433	94.04%	0.138300	0.199400	0.00445900	0.01841000	ENST00000295509	upstream	51424	---	---
rs261009	5	169272733	0.4462	97.89%	0.116800	0.295000	0.00446400	0.06296000	NM_004946	intron	0	Hs.586174	DOCK2
rs9317034	13	58443546	0.3151	92.98%	0.394800	0.406400	0.00447500	0.00430200	ENST00000365514	upstream	443660	---	---
rs466380	5	129204748	0.0881	97.54%	0.246400	0.019240	0.00448400	0.00497200	NM_175856	upstream	63674	Hs.213137	CHSY-2
rs10505670	8	138282298	0.0461	95.09%	0.440100	1.000000	0.00449600	0.00195800	NM_015912	downstream	931410	Hs.126024	FAM135B
rs4376443	7	110178825	0.2967	95.79%	0.083110	0.317200	0.00452600	0.00603900	NM_032549	intron	0	Hs.655722	IMMP2L
rs10485128	6	79428165	0.1302	92.98%	0.173800	0.658900	0.00453600	0.01912000	NM_001010844	upstream	205743	Hs.656212	IRAK1BP1
rs2102137	5	104720450	0.1421	95.09%	0.317900	1.000000	0.00455600	0.00580100	ENST00000364920	downstream	566155	---	---
rs1326468	13	47064268	0.1212	92.63%	1.000000	0.366600	0.00456600	0.01041000	NM_003850	downstream	350524	Hs.546323	SUCLA2
rs10511695	9	21035062	0.2222	94.74%	0.596700	1.000000	0.00457200	0.00727800	NM_001010915	upstream	13422	Hs.136247	PTPLAD2
rs9314317	8	25889869	0.1162	95.09%	0.227400	0.624300	0.00457200	0.02241000	ENST00000380737	intron	0	---	---

rs10504243	8	59148054	0.0615	85.61%	0.000000	0.000000	0.00458800	0.09920000	NM_147189	intron	0	Hs.154652	FAM110B
rs10483385	14	29697941	0.0092	95.09%	1.000000	1.000000	0.00459300	0.99840000	NM_017769	upstream	400187	Hs.509008	KIAA1333
rs10518281	19	20282246	0.0093	94.04%	1.000000	1.000000	0.00459800	0.99840000	NM_021047	downstream	5835	Hs.659321	ZNF253
rs1578125	1	191885531	0.0773	97.54%	0.066870	0.083310	0.00460700	0.02722000	NM_024529	downstream	395877	Hs.576497	CDC73
rs763836	2	29758808	0.2372	96.14%	0.403900	1.000000	0.00464300	0.00675700	NM_004304	intron	0	Hs.654469	ALK
rs9317121	13	60038079	0.1045	94.04%	0.014640	0.014440	0.00465000	0.02825000	NM_030794	intron	0	Hs.525061	TDRD3
rs10496771	2	137913739	0.1906	97.54%	0.845700	0.830500	0.00465400	0.02262000	ENST00000272643	intron	0	---	---
rs10504452	8	70626236	0.1330	93.68%	0.281600	0.262000	0.00466400	0.01458000	NM_015170	intron	0	Hs.409602	SULF1
rs2153639	6	63803845	0.3817	91.93%	0.088190	0.028280	0.00467100	0.00213600	ENST00000370659	upstream	175465	---	---
rs10495671	2	17907509	0.4910	97.54%	0.071520	0.071730	0.00467800	0.01394000	NM_002252	upstream	15917	Hs.414489	KCNS3
rs9969156	7	11158039	0.0093	94.74%	1.000000	1.000000	0.00467900	0.99840000	NM_014660	intron	0	Hs.655688	PHF14
rs10514492	16	78917727	0.2413	90.88%	0.311500	0.849800	0.00469100	0.02692000	NM_130897	upstream	214628	Hs.98849	DYNLRB2
rs10517457	4	37631234	0.1320	94.39%	0.191200	0.260800	0.00470700	0.00438100	NM_015173	intron	0	Hs.176503	TBC1D1
rs1412859	13	60295951	0.1888	97.54%	0.169300	0.281400	0.00471300	0.00540300	NM_022843	downstream	585868	Hs.391781	PCDH20
rs1016425	18	62675708	0.3717	92.98%	0.896100	1.000000	0.00471700	0.00351900	NM_021153	upstream	253512	Hs.42771	CDH19
rs10491541	9	84709128	0.3050	76.49%	0.002302	0.016130	0.00472500	0.02795000	NM_152573	downstream	75192	Hs.657750	RASEF
rs10497167	2	156745232	0.2879	92.63%	0.016250	0.534900	0.00475500	0.01197000	NM_173173	downstream	143962	Hs.563344	NR4A2
rs645462	1	107758386	0.0093	94.39%	1.000000	1.000000	0.00476700	0.99840000	NM_014917	intron	0	Hs.657434	NTNG1
rs2288627	2	222028671	0.2644	97.54%	0.644000	1.000000	0.00481300	0.00227400	NM_004438	intron	0	Hs.371218	EPHA4
rs2208363	1	116519546	0.3185	94.74%	0.068410	0.007812	0.00482000	0.02042000	NM_152367	downstream	40162	Hs.376194	C1orf161
rs10490569	2	25854931	0.2391	96.14%	0.410600	0.573700	0.00484400	0.00237600	NM_018263	intron	0	Hs.700650	ASXL2
rs1144700	6	16852666	0.2838	80.35%	0.000000	0.000045	0.00484500	0.01023000	NM_000332	intron	0	Hs.434961	ATXN1
rs1418245	9	103800425	0.2968	97.54%	0.565600	0.316300	0.00485300	0.00492600	ENST00000386444	upstream	204498	---	---
rs2370596	12	2536609	0.3222	94.74%	0.405100	0.578000	0.00486000	0.01263000	NM_000719	intron	0	Hs.118262	CACNA1C
rs1510228	6	160727393	0.2075	92.98%	0.851700	1.000000	0.00486500	0.01454000	NM_021977	intron	0	Hs.567337	SLC22A3
rs684431	1	206232040	0.4833	94.39%	0.328900	0.879700	0.00487000	0.01062000	NM_025179	downstream	30170	Hs.497626	PLXNA2
rs1788153	8	100749604	0.2873	94.04%	0.138900	0.039190	0.00488400	0.02248000	NM_017890	intron	0	Hs.191540	VPS13B
rs10497284	2	166915599	0.1827	95.09%	1.000000	0.662400	0.00490000	0.01818000	ENST00000385475	upstream	7895	---	---
rs2893603	8	128149139	0.3825	94.04%	0.517600	0.877600	0.00491200	0.00080360	ENST00000385818	downstream	396058	---	---
rs1541836	18	19749481	0.3480	95.79%	0.893900	1.000000	0.00491900	0.00988200	NM_000227	intron	0	Hs.436367	LAMA3
rs530701	12	68268129	0.1981	94.74%	0.341400	0.832500	0.00492200	0.03020000	NM_006431	intron	0	Hs.189772	CCT2
rs1370107	10	128272839	0.1297	93.33%	0.589400	0.256200	0.00492300	0.01079000	NM_001004298	upstream	72839	Hs.587663	C10orf90
rs336604	3	18595288	0.2712	95.09%	0.539200	0.668800	0.00493400	0.00239100	NM_002971	upstream	140080	Hs.517717	SATB1
rs560104	9	109774726	0.3315	93.68%	0.333700	0.063880	0.00494300	0.00748100	ENST00000297812	upstream	137795	---	---
rs10492534	13	96286031	0.0094	93.68%	1.000000	1.000000	0.00494900	0.99840000	NM_153456	downstream	2359	Hs.171001	HS6ST3
rs10509334	10	73110058	0.3035	80.35%	0.000280	0.000037	0.00495800	0.02532000	NM_052836	intron	0	Hs.656032	CDH23
rs1018462	6	162061886	0.2565	94.39%	0.053880	0.365300	0.00496700	0.01824000	NM_013988	intron	0	Hs.132954	PARK2
rs283625	5	73235295	0.3381	86.67%	0.001008	0.013360	0.00496700	0.01616000	ENST00000287898	intron	0	---	---
rs797124	6	5305431	0.3971	97.19%	0.104300	0.127600	0.00498400	0.01073000	NM_006567	intron	0	Hs.484547	FARS2
rs10489592	1	118241518	0.0944	94.74%	1.000000	0.178000	0.00498700	0.00811500	NM_017686	intron	0	Hs.705484	GDAP2
rs10487394	7	117672274	0.0644	92.63%	0.288500	1.000000	0.00501200	0.03244000	NM_019644	downstream	2297	Hs.657737	ANKRD7
rs10493367	1	64978736	0.1172	95.79%	0.232600	0.625100	0.00502300	0.00865400	NM_018211	upstream	4630	Hs.591443	RAVER2
rs6904988	6	149134151	0.2690	97.19%	0.033380	0.035990	0.00502400	0.01013000	NM_005715	intron	0	Hs.657370	UST
rs6962865	7	133249973	0.3014	97.19%	0.198100	0.506500	0.00503000	0.02385000	NM_001037126	intron	0	Hs.321273	EXOC4
rs6963032	7	133250067	0.3014	97.19%	0.198100	0.506500	0.00503000	0.02385000	NM_001037126	intron	0	Hs.321273	EXOC4
rs10509453	10	84043171	0.3957	97.54%	0.900600	0.743000	0.00504800	0.03098000	NM_001010848	intron	0	Hs.125119	NRG3
rs2127125	13	80754418	0.3957	97.54%	0.532200	1.000000	0.00504800	0.00137800	ENST00000387380	downstream	16466	---	---
rs10504607	8	77124396	0.0888	90.88%	1.000000	1.000000	0.00504900	0.00364100	ENST00000388687	downstream	86681	---	---
rs10508689	10	25562286	0.1476	95.09%	0.330900	1.000000	0.00507000	0.02765000	NM_020752	intron	0	Hs.499108	GPR158
rs9294066	6	63801937	0.3900	90.88%	0.013030	0.002401	0.00507500	0.00334000	ENST00000370659	upstream	177373	---	---
rs851982	6	152066678	0.4106	96.14%	0.707900	0.648900	0.00508200	0.00284900	NM_025059	downstream	82658	Hs.660044	C6orf97
rs10484538	6	23576145	0.0333	94.74%	0.253900	1.000000	0.00508800	0.00772600	ENST00000384664	upstream	342851	---	---
rs2413163	22	31777946	0.3059	95.79%	0.887100	0.241200	0.00509400	0.00790100	NM_133633	upstream	45299	Hs.658998	SYN3
rs1927626	9	16577758	0.0618	96.49%	1.000000	0.234600	0.00509900	0.00586600	NM_017637	intron	0	Hs.656581	BNC2

rs10500734	11	10898494	0.1310	95.09%	0.790700	1.000000	0.00511600	0.00982800	NM_021211	upstream	62304	---	ZBED5
rs1422004	5	108680199	0.4058	96.84%	0.061530	0.141700	0.00513200	0.00975500	NM_014819	downstream	18110	Hs.483036	PJA2
rs576861	1	223810960	0.4164	96.49%	1.000000	0.762100	0.00514100	0.01080000	NM_018212	intron	0	Hs.497893	ENAH
rs3788961	2	17962686	0.3046	91.58%	0.561900	0.326500	0.00514400	0.03106000	NM_002252	intron	0	Hs.414489	KCNS3
rs4655769	1	68249566	0.1594	96.84%	0.653100	0.809200	0.00516300	0.07540000	NM_004675	downstream	34667	Hs.194695	DIRAS3
rs10504600	8	77043891	0.1066	85.61%	0.086300	0.601000	0.00517900	0.00515800	ENST00000388682	upstream	76197	---	---
rs458490	5	129198311	0.0849	95.09%	0.110100	0.014690	0.00519800	0.00755700	NM_175856	upstream	70111	Hs.213137	CHSY-2
rs9294040	6	77653677	0.2097	87.02%	0.442800	1.000000	0.00519900	0.00547400	NM_000863	downstream	574990	Hs.123016	HTR1B
rs4074672	3	185212989	0.3321	92.98%	0.582000	0.348600	0.00522300	0.00086480	NM_001023587	intron	0	Hs.368563	ABCC5
rs1527894	2	49501529	0.1953	97.89%	0.187300	0.780200	0.00524200	0.00283400	ENST00000384188	upstream	186987	---	---
rs10487563	7	48905713	0.1899	93.33%	0.842700	1.000000	0.00528700	0.01365000	ENST00000324993	upstream	28990	---	---
rs10504463	8	71109147	0.1272	97.89%	1.000000	1.000000	0.00531700	0.01245000	NM_024504	downstream	17427	Hs.287532	PRDM14
rs2206416	20	39994257	0.3978	94.39%	0.704000	1.000000	0.00532100	0.02191000	NM_007050	downstream	140549	Hs.526879	PTPRT
rs2254476	21	22157475	0.4205	92.63%	0.613200	0.145400	0.00532100	0.02564000	ENST00000358903	downstream	219002	---	---
rs10484203	14	48649112	0.0102	86.32%	0.020300	1.000000	0.00532800	0.99830000	ENST00000386332	upstream	439989	---	---
rs1402111	15	52599143	0.1774	97.89%	0.680400	0.761300	0.00535800	0.03704000	ENST00000260323	intron	0	---	---
rs1517044	6	70950780	0.4206	97.19%	1.000000	1.000000	0.00537900	0.00484900	NM_001858	intron	0	Hs.444842	COL19A1
rs291870	5	102869587	0.1704	94.74%	1.000000	1.000000	0.00538800	0.01274000	NM_031438	downstream	42868	Hs.434289	NUDT12
rs10510207	3	1309530	0.0415	92.98%	1.000000	1.000000	0.00539300	0.01141000	NM_014461	intron	0	Hs.387300	CNTN6
rs2549513	16	78108228	0.1063	94.04%	0.095520	0.191700	0.00539300	0.00296600	NM_001031804	downstream	77508	Hs.134859	MAF
rs10517967	4	66752247	0.3667	94.74%	0.149200	0.486100	0.00540500	0.02133000	ENST00000339867	downstream	109410	---	---
rs2199133	13	85821511	0.0425	90.88%	1.000000	1.000000	0.00540700	0.01777000	NM_032229	upstream	552867	Hs.525105	SLITRK6
rs4941701	13	32383476	0.1357	94.39%	0.797000	0.697500	0.00540800	0.01510000	NM_015032	downstream	133319	Hs.699308	PDS5B
rs7578684	2	56485672	0.4291	91.58%	0.208600	0.360900	0.00541000	0.00628200	NM_006296	upstream	1641552	Hs.631890	VRK2
rs2380684	2	15915808	0.3668	96.14%	1.000000	0.877500	0.00541100	0.01626000	NM_004939	downstream	227132	Hs.440599	DDX1
rs9308849	2	101891819	0.2849	90.53%	0.091960	0.531800	0.00541300	0.00531300	NM_145687	downstream	14236	Hs.431550	MAP4K4
rs9293473	5	86003832	0.0430	89.82%	0.375700	1.000000	0.00541400	0.01514000	NM_001039781	downstream	377687	---	LOC645261
rs10497168	2	156743961	0.3948	88.42%	0.692500	0.752300	0.00541700	0.00065260	NM_173173	downstream	145233	Hs.563344	NR4A2
rs2126270	4	179740134	0.1094	92.98%	0.216300	0.528800	0.00541800	0.04063000	ENST00000364857	downstream	175176	---	---
rs10488500	7	76679195	0.0512	89.12%	1.000000	1.000000	0.00543400	0.02862000	NM_020879	intron	0	Hs.113940	CCDC146
rs10488164	7	132756739	0.1896	94.39%	0.326600	0.191200	0.00544800	0.00394500	NM_001037126	intron	0	Hs.321273	EXOC4
rs10497330	2	168508565	0.0321	87.37%	0.220200	1.000000	0.00547600	0.05666000	NM_013233	downstream	10212	Hs.276271	STK39
rs10484612	6	99516963	0.0717	97.89%	0.007799	0.328600	0.00548700	0.01328000	NM_012160	upstream	14393	Hs.536850	FBXL4
rs2291103	10	3180797	0.2135	93.68%	1.000000	0.795300	0.00549800	0.04133000	NM_014889	intron	0	Hs.528300	PITRM1
rs6736527	2	64806767	0.0653	94.04%	0.017830	1.000000	0.00549900	0.02446000	NM_014755	upstream	72217	Hs.693696	SERTAD2
rs1414185	6	77626327	0.2389	86.67%	0.000789	0.010850	0.00551100	0.00662900	NM_000863	downstream	602314	Hs.123016	HTR1B
rs10502313	18	3310158	0.0207	84.56%	0.002677	1.000000	0.00555300	0.03962000	NM_033546	downstream	41911	Hs.464472	MRLC2
rs2157988	17	65225865	0.3974	80.35%	0.000000	0.000000	0.00555400	0.07882000	NM_002758	downstream	174798	Hs.463978	MAP2K6
rs2807238	10	25548043	0.1537	94.74%	1.000000	0.476700	0.00556200	0.01341000	NM_020752	intron	0	Hs.499108	GPR158
rs10518026	4	67747953	0.0343	97.19%	0.273400	1.000000	0.00558100	0.00217800	ENST00000383902	downstream	229952	---	---
rs7126337	11	7233719	0.4353	97.54%	0.903500	0.209800	0.00559000	0.00489600	NM_175733	intron	0	Hs.177193	SYT9
rs10502019	11	103218742	0.1222	94.74%	0.255400	1.000000	0.00564200	0.00047050	NM_033135	downstream	64389	Hs.352298	PDGFD
rs152581	5	106822183	0.4229	97.89%	0.327100	0.231100	0.00564900	0.00815200	NM_001962	intron	0	Hs.658451	EFNA5
rs10513373	3	151604811	0.2015	95.79%	0.708000	1.000000	0.00565700	0.00761600	NM_014779	upstream	4001	Hs.644065	TSC22D2
rs2457570	6	160749508	0.1896	94.39%	1.000000	0.769800	0.00565900	0.00835500	NM_021977	intron	0	Hs.567337	SLC22A3
rs266638	5	125704767	0.0233	97.89%	0.133200	0.202100	0.00566400	0.99770000	NM_023927	upstream	82233	Hs.363558	GRAMD3
rs1386761	8	89617544	0.1444	97.19%	0.139400	0.191600	0.00567400	0.02484000	NM_003821	upstream	1221629	Hs.103755	RIPK2
rs10511151	3	96496507	0.4406	97.54%	1.000000	0.536200	0.00568000	0.06433000	ENST00000332119	upstream	356949	---	---
rs9288489	2	214758120	0.0243	93.68%	1.000000	1.000000	0.00568100	0.99730000	NM_001025436	intron	0	Hs.705527	SPAG16
rs4128555	7	152967375	0.4473	96.49%	0.902900	0.546200	0.00568300	0.00848600	NM_207367	upstream	225394	---	FLJ42291
rs495862	11	93706705	0.3730	87.02%	0.021330	0.054430	0.00569100	0.03666000	NM_016540	downstream	43426	Hs.272385	GPR83
rs7692183	4	85558774	0.0989	94.04%	0.304700	0.602500	0.00572500	0.01590000	NM_006168	downstream	74686	Hs.546270	NKX6-1
rs870029	4	189633069	0.1101	94.04%	0.340000	0.095120	0.00573300	0.04609000	NM_178556	downstream	327426	Hs.348618	TRIML1
rs260454	19	63458980	0.3707	92.28%	0.894800	0.530200	0.00574600	0.04617000	NM_014480	intron	0	Hs.438994	ZNF544

rs10512610	17	72085727	0.1141	92.28%	0.219300	0.205800	0.00574800	0.01957000	NM_006456	intron	0	Hs.592105	ST6GALNAC
rs1480147	8	16170358	0.3159	97.19%	0.334100	0.708300	0.00575400	0.04397000	NM_138716	upstream	75763	Hs.147635	MSR1
rs10508058	13	100659913	0.2883	96.14%	0.007836	0.222100	0.00577300	0.01706000	NM_052867	intron	0	Hs.525146	NALCN
rs7724762	5	40090135	0.4391	97.89%	1.000000	0.296400	0.00578100	0.00790800	NM_001343	upstream	629432	Hs.481980	DAB2
rs10519102	15	45194506	0.4585	97.19%	0.039360	0.036460	0.00578300	0.00306800	ENST00000381028	downstream	18636	---	---
rs963731	2	39070377	0.1117	92.63%	0.546100	0.747400	0.00578400	0.00902100	NM_005633	intron	0	Hs.654397	SOS1
rs2310160	4	186427288	0.4319	97.89%	0.627100	0.636900	0.00579800	0.00163900	NM_031953	intron	0	Hs.369091	SNX25
rs4135304	3	12369601	0.0240	95.09%	1.000000	1.000000	0.00581000	0.99760000	NM_005037	intron	0	Hs.162646	PPARG
rs10498243	2	230708466	0.3039	81.40%	0.163000	0.047560	0.00581600	0.00455000	NM_080424	downstream	33618	Hs.145150	SP110
rs7615571	3	123148640	0.3664	97.19%	0.897000	0.755700	0.00582800	0.00492400	NM_021082	downstream	5493	Hs.518089	SLC15A2
rs994580	8	113029469	0.1245	94.39%	0.011090	0.007615	0.00583500	0.02873000	ENST00000364160	downstream	200627	---	---
rs1423399	5	41160829	0.3773	95.79%	0.606700	0.497000	0.00584700	0.02673000	NM_000065	downstream	17264	Hs.481992	C6
rs10487052	7	86248625	0.1679	96.14%	0.384000	0.518200	0.00585700	0.02052000	NM_000840	intron	0	Hs.590575	GRM3
rs10484875	6	52377895	0.1093	94.74%	0.338700	0.532800	0.00587100	0.03335000	NM_133367	3UTR	0	Hs.239388	PAQR8
rs726646	1	101711251	0.5000	96.84%	0.230100	0.452900	0.00587400	0.01205000	NM_001400	downstream	231589	Hs.154210	EDG1
rs1458	19	39351986	0.4946	96.84%	0.148200	0.068880	0.00587500	0.03273000	NM_015578	upstream	3325	Hs.407368	LSM14A
rs1601467	8	25977183	0.2842	97.54%	0.027280	0.305400	0.00587500	0.06109000	ENST00000380737	intron	0	---	---
rs3849443	3	96354919	0.4565	96.84%	0.903800	0.641500	0.00587500	0.07639000	ENST00000332119	upstream	215361	---	---
rs10508060	13	100756272	0.2667	89.47%	0.076740	0.127700	0.00589000	0.02001000	NM_052867	intron	0	Hs.525146	NALCN
rs7791118	7	13274540	0.2574	95.44%	1.000000	0.719000	0.00589500	0.02725000	NM_004956	downstream	622839	Hs.22634	ETV1
rs7009407	8	107985237	0.0396	97.54%	0.351000	0.431600	0.00590300	0.01844000	NM_139166	upstream	133589	Hs.374668	ABRA
rs1419294	10	8347160	0.4728	96.84%	1.000000	0.368200	0.00590600	0.01650000	NM_002051	downstream	189990	Hs.524134	GATA3
rs10485712	20	7044163	0.0746	94.04%	0.046370	0.343300	0.00591700	0.02312000	NM_001200	downstream	335253	Hs.73853	BMP2
rs828721	5	162240179	0.1598	93.33%	0.645900	0.739300	0.00593000	0.00238100	NM_199246	upstream	556976	Hs.79101	CCNG1
rs2011341	4	10218108	0.0256	89.12%	0.145700	0.214300	0.00593900	0.99750000	NM_052964	intron	0	Hs.678910	MIST
rs2660589	4	63555481	0.1000	94.74%	0.736900	1.000000	0.00595400	0.01371000	NM_001010874	downstream	1272904	Hs.227752	SRD5A2L2
rs10505197	8	114044747	0.0394	97.89%	1.000000	1.000000	0.00597900	0.02019000	NM_052900	intron	0	Hs.91381	CSMD3
rs1848864	12	113984486	0.4606	97.89%	0.717600	0.877700	0.00599300	0.03752000	ENST00000386518	downstream	324483	---	---
rs1079654	6	132648471	0.0932	97.89%	0.275600	0.562100	0.00599400	0.00764800	NM_001031699	downstream	10416	---	MOXD1
rs4669060	2	6395879	0.0932	97.89%	0.146300	1.000000	0.00599400	0.00196100	NM_001001695	downstream	390904	---	FLJ42418
rs8044486	16	81044043	0.1734	95.09%	0.674200	0.535700	0.00599700	0.03272000	NM_005792	upstream	282730	Hs.344400	MPHOSPH6
rs890681	2	25831991	0.2025	97.89%	0.852900	0.682300	0.00601100	0.00291500	NM_018263	intron	0	Hs.700650	ASXL2
rs1364637	2	218416677	0.3195	93.33%	0.322900	0.742500	0.00602600	0.08833000	NM_022648	intron	0	Hs.471381	TNS1
rs1374031	5	63639566	0.2201	94.04%	0.155600	0.324200	0.00603500	0.00803500	ENST00000389100	intron	0	---	---
rs275754	15	37904019	0.5000	75.44%	0.055790	0.129700	0.00604100	0.03559000	NM_007223	intron	0	Hs.37196	GPR176
rs953073	5	85051865	0.3208	97.89%	1.000000	0.459100	0.00605100	0.00482800	ENST00000333189	downstream	170341	---	---
rs6816174	4	11218802	0.4765	97.19%	0.278600	0.167300	0.00606000	0.01723000	NM_005114	upstream	178315	Hs.507348	HS3ST1
rs10521130	17	4056773	0.4819	96.84%	0.546400	0.447400	0.00606800	0.00990000	NM_020740	intron	0	Hs.696087	ANKFY1
rs1396798	2	69741341	0.2518	97.54%	0.635400	0.658400	0.00607400	0.06225000	ENST00000386230	upstream	6654	---	---
rs10507918	13	80774365	0.3773	97.19%	0.523300	1.000000	0.00608900	0.00273000	ENST00000387380	downstream	36413	---	---
rs594516	15	43722685	0.2068	97.54%	1.000000	1.000000	0.00609000	0.01205000	NM_021199	intron	0	Hs.511251	SQRDL
rs3847896	12	23051233	0.4892	97.54%	0.471300	0.178000	0.00609400	0.00116400	NM_178010	downstream	525266	Hs.657542	SOX5
rs6684	6	97445799	0.4114	89.12%	0.697500	1.000000	0.00610500	0.05572000	NM_014165	CDS	0	Hs.591333	C6orf66
rs10489869	1	190782401	0.4850	93.68%	0.806500	0.649700	0.00610600	0.02074000	NM_002922	upstream	29079	Hs.75256	RGS1
rs575309	4	63963215	0.1531	95.09%	0.098520	0.718600	0.00612100	0.01313000	NM_001010874	downstream	865170	Hs.227752	SRD5A2L2
rs1564032	2	6446955	0.1149	91.58%	0.032070	0.613000	0.00614400	0.00193900	NM_001001695	downstream	339828	---	FLJ42418
rs8027192	15	69793594	0.0695	90.88%	0.111500	0.143700	0.00615300	0.07034000	NM_024817	intron	0	Hs.387057	THSD4
rs10519703	5	121867356	0.1057	97.89%	0.021520	0.286100	0.00615400	0.02663000	NM_005460	downstream	39663	Hs.426463	SNCAIP
rs1382061	15	25430180	0.3759	97.54%	0.159300	0.440800	0.00618500	0.01183000	NM_033223	intron	0	Hs.569475	GABRG3
rs1660797	10	53916144	0.0415	97.19%	1.000000	1.000000	0.00619900	0.02651000	NM_012242	downstream	168721	Hs.40499	DKK1
rs998125	1	64984109	0.1332	80.35%	0.253400	0.642500	0.00621700	0.01674000	NM_018211	intron	0	Hs.591443	RAVER2
rs9312212	4	178906882	0.3619	94.04%	0.046660	0.165300	0.00622700	0.05214000	ENST00000365495	downstream	167814	---	---
rs10507917	13	80741431	0.4299	95.09%	0.805300	1.000000	0.00624900	0.00236700	ENST00000387380	downstream	3479	---	---
rs10506975	12	88510654	0.0412	97.89%	1.000000	1.000000	0.00625400	0.02554000	NM_001682	intron	0	Hs.506276	ATP2B1

rs1733732	10	53932187	0.0412	97.89%	1.000000	1.000000	0.00625400	0.02298000	NM_012242	downstream	184764	Hs.40499	DKK1
rs10482860	21	15116371	0.0473	96.49%	0.462000	1.000000	0.00625800	0.00521900	ENST00000389440	intron	0	---	---
rs994214	2	77798750	0.3807	92.63%	0.603100	0.236700	0.00626400	0.05168000	ENST00000386159	downstream	49579	---	---
rs10502250	11	121908673	0.0851	96.84%	0.118800	0.467500	0.00626800	0.01377000	ENST00000364077	downstream	46450	---	---
rs2175573	15	45194356	0.3407	95.79%	0.014910	0.054030	0.00627100	0.00512200	ENST00000381028	downstream	18786	---	---
rs1241078	18	31387808	0.0695	93.33%	0.362400	0.638000	0.00627300	0.01593000	NM_194281	upstream	55855	Hs.464903	C18orf37
rs759324	16	9499204	0.1880	96.14%	0.016440	0.138400	0.00627600	0.06363000	ENST00000363598	downstream	66544	---	---
rs1479640	1	162214997	0.4554	94.39%	0.085010	0.756000	0.00629300	0.06535000	ENST00000385701	downstream	55263	---	---
rs10509499	10	86452994	0.0817	88.07%	0.015370	0.019240	0.00631200	0.01608000	NM_018999	downstream	184738	Hs.461988	KIAA1128
rs9284423	18	36256475	0.2500	94.04%	0.513900	0.854500	0.00632300	0.01185000	ENST00000364090	upstream	50309	---	---
rs6988978	8	13261437	0.0885	85.26%	0.411000	0.466600	0.00632700	0.00942800	NM_024767	intron	0	Hs.134296	DLC1
rs851983	6	152066108	0.4121	95.79%	0.708100	0.652500	0.00632800	0.00345100	NM_025059	downstream	82088	Hs.660044	C6orf97
rs10516484	4	103011108	0.1296	66.32%	0.004783	0.020620	0.00633900	0.00421400	NM_017935	intron	0	Hs.480400	BANK1
rs1959573	14	48204884	0.0493	96.14%	0.490000	0.570900	0.00637400	0.02284000	ENST00000384636	upstream	324599	---	---
rs883398	1	63261928	0.1426	94.74%	0.453600	0.607200	0.00637700	0.05601000	NM_178221	downstream	158156	Hs.7353	ATG4C
rs4725680	7	145064072	0.4667	94.74%	0.714500	0.282300	0.00638500	0.00488500	ENST00000385572	upstream	261282	---	---
rs10514457	16	78063502	0.0466	97.89%	0.457000	1.000000	0.00639000	0.02599000	NM_001031804	downstream	122234	Hs.134859	MAF
rs7017825	8	108819531	0.3309	97.54%	0.223600	0.521500	0.00640200	0.00523800	NM_001146	upstream	240072	Hs.369675	ANGPT1
rs10517999	4	169665396	0.0698	92.98%	1.000000	0.304600	0.00640300	0.02526000	NM_016081	intron	0	Hs.151220	PALLD
rs9317845	13	69240293	0.1563	95.44%	0.644400	0.333200	0.00640600	0.03389000	NM_020866	intron	0	Hs.508201	KLHL1
rs1417696	6	63795895	0.3903	97.54%	0.377900	0.096070	0.00640800	0.00278400	ENST00000356170	upstream	183526	---	---
rs4128784	4	30481748	0.0848	97.19%	0.427000	0.111300	0.00640800	0.02596000	NM_032457	intron	0	Hs.570785	PCDH7
rs7560607	2	64807513	0.0578	94.04%	0.006973	1.000000	0.00641200	0.03096000	NM_014755	upstream	72963	Hs.693696	SERTAD2
rs9327287	5	122547512	0.3950	91.93%	0.518600	0.641800	0.00641400	0.03555000	ENST00000261364	intron	0	---	---
rs10485510	20	33908578	0.1004	94.39%	0.737700	1.000000	0.00641800	0.01250000	NM_016436	intron	0	Hs.517044	PHF20
rs10501129	11	33376740	0.4611	94.74%	0.624500	0.440100	0.00642300	0.01158000	NM_005734	downstream	45050	Hs.201918	HIPK3
rs6531074	2	16408444	0.2814	81.05%	0.142200	0.824300	0.00642800	0.02846000	ENST00000388035	downstream	164399	---	---
rs10519389	2	230151820	0.0605	89.82%	1.000000	1.000000	0.00643400	0.00574700	NM_139072	intron	0	Hs.234074	DNER
rs1155642	15	95303615	0.3967	96.84%	0.615100	0.869200	0.00643700	0.00605400	ENST00000364271	downstream	116985	---	---
rs1343870	14	51430563	0.1855	89.82%	0.678000	1.000000	0.00644400	0.00203500	NM_053064	intron	0	Hs.705393	GNG2
rs10483430	14	32711870	0.3794	88.77%	1.000000	0.388800	0.00645300	0.01460000	NM_022123	intron	0	Hs.659456	NPAS3
rs10514276	5	85449460	0.1685	97.89%	0.391000	0.648800	0.00646500	0.06344000	ENST00000380134	upstream	164558	---	---
rs2504939	6	160752140	0.1934	96.14%	0.846000	0.576400	0.00646700	0.01200000	NM_021977	intron	0	Hs.567337	SLC22A3
rs10493147	4	128956949	0.1493	97.54%	1.000000	1.000000	0.00648600	0.09988000	NM_014278	intron	0	Hs.135554	HSPA4L
rs10516016	5	165911472	0.0424	95.09%	1.000000	1.000000	0.00648600	0.01228000	ENST00000320147	downstream	169285	---	---
rs10511468	9	7377977	0.1705	90.53%	0.124600	0.059890	0.00650600	0.05293000	NM_015061	downstream	212329	Hs.157106	JMJD2C
rs3091599	20	39830225	0.1709	97.54%	0.673700	0.489500	0.00655600	0.03130000	NM_007050	downstream	304581	Hs.526879	PTPRT
rs39745	7	117492493	0.0964	96.49%	0.089330	0.724800	0.00657200	0.06776000	NM_016200	upstream	118962	Hs.657737	LSM8
rs1508250	6	87171740	0.1531	90.53%	0.000428	0.010450	0.00657700	0.09215000	ENST00000388670	upstream	30546	---	---
rs10488453	7	134056142	0.3878	85.96%	0.007008	0.052400	0.00658300	0.00616400	NM_199186	downstream	41037	Hs.198365	BPGM
rs268870	2	65337053	0.2356	97.54%	0.506200	0.480000	0.00658700	0.00833000	NM_005722	intron	0	Hs.699451	ACTR2
rs2652072	5	134767194	0.1316	93.33%	1.000000	1.000000	0.00658900	0.05088000	NM_001040158	upstream	3718	Hs.696013	H2AFY
rs702844	5	89410835	0.3400	96.49%	0.893600	0.718300	0.00658900	0.00178100	NM_004365	downstream	314452	Hs.591767	CETN3
rs1531575	13	80759483	0.3935	97.19%	0.615000	1.000000	0.00659100	0.00264100	ENST00000387380	downstream	21531	---	---
rs17581017	13	100452590	0.0971	97.54%	0.305800	0.310700	0.00660100	0.00972000	NM_052867	downstream	51539	Hs.525146	NALCN
rs3134171	8	100593748	0.3516	95.79%	0.287900	0.034210	0.00660100	0.03236000	NM_017890	intron	0	Hs.191540	VPS13B
rs460136	5	129204786	0.0873	96.49%	0.135700	0.019540	0.00661300	0.00927000	NM_175856	upstream	63636	Hs.213137	CHSY-2
rs10483167	22	31655803	0.2876	67.72%	0.112700	0.159300	0.00662400	0.00524400	NM_133633	intron	0	Hs.658998	SYN3
rs1829684	1	166125505	0.1423	93.68%	0.448700	1.000000	0.00662700	0.00719700	NM_018417	intron	0	Hs.320892	SAC
rs667419	5	131309963	0.3170	92.98%	0.010250	0.020030	0.00662900	0.00824700	ENST00000296869	intron	0	---	---
rs4525555	17	42692948	0.2394	90.88%	0.087170	0.445200	0.00664100	0.03366000	NM_000212	intron	0	Hs.218040	ITGB3
rs769039	5	121872093	0.2815	94.74%	0.175700	0.153200	0.00665100	0.09607000	NM_005460	downstream	44400	Hs.426463	SNCAIP
rs31319	5	123642159	0.1455	94.04%	1.000000	1.000000	0.00665400	0.02454000	NM_020747	downstream	358350	Hs.266616	ZNF608
rs648097	1	18542037	0.2852	94.74%	0.136200	1.000000	0.00666200	0.02613000	NM_032880	intron	0	Hs.212511	IGSF21

rs10504509	8	72634113	0.0684	82.11%	0.014510	0.237700	0.00666700	0.03310000	NM_172059	upstream	197092	Hs.491997	EYA1
rs233598	21	27252709	0.1624	95.09%	0.657400	0.479300	0.00670600	0.04186000	NM_007038	intron	0	Hs.58324	ADAMTS5
rs3935969	17	63312421	0.3004	97.54%	0.886500	0.845800	0.00672100	0.03394000	NM_004459	intron	0	Hs.444200	BPTF
rs1836541	15	60649430	0.2978	97.19%	0.116000	0.064080	0.00673200	0.04330000	NM_015059	upstream	77372	Hs.569438	TLN2
rs3898762	18	9565419	0.1868	92.98%	1.000000	1.000000	0.00673300	0.00352300	NM_005134	intron	0	Hs.464595	PPP4R1
rs4799697	18	29263749	0.2914	93.33%	0.104500	0.172600	0.00673300	0.02957000	NM_198995	intron	0	Hs.115461	C18orf34
rs1604355	1	188725630	0.1097	97.54%	1.000000	0.608900	0.00679400	0.02761000	NM_199051	upstream	12248	Hs.65765	FAM5C
rs1339658	10	20737858	0.3392	79.65%	0.102400	0.695300	0.00681700	0.01176000	NM_032812	downstream	128566	Hs.658134	PLXDC2
rs2616231	8	20613527	0.3491	96.49%	0.508500	0.860400	0.00682400	0.00658600	ENST00000387608	upstream	96810	---	---
rs2389530	13	95348755	0.4572	94.39%	0.462700	0.347100	0.00683200	0.01857000	NM_020121	intron	0	Hs.193226	UGCGL2
rs261861	1	239162199	0.4805	89.82%	0.213300	0.280800	0.00683400	0.00173500	NM_002924	intron	0	Hs.655739	RGS7
rs1400131	2	183709600	0.3000	96.49%	0.084110	0.612900	0.00683600	0.01862000	NM_001008544	intron	0	---	NUP35
rs1428504	5	164770559	0.2198	95.79%	0.214600	0.554200	0.00684300	0.00306800	ENST00000385732	upstream	198465	---	---
rs234443	14	96980650	0.2357	92.28%	0.395700	1.000000	0.00687900	0.00278500	NM_003384	downstream	562947	Hs.422662	VRK1
rs7922744	10	107572127	0.0500	94.74%	1.000000	1.000000	0.00688300	0.00944900	NM_001013031	downstream	754842	Hs.591915	SORCS1
rs9304435	18	48670227	0.0500	94.74%	1.000000	1.000000	0.00688300	0.01522000	NM_005215	intron	0	Hs.579550	DCC
rs2575710	8	97653152	0.4218	96.49%	0.901700	1.000000	0.00689600	0.02297000	NM_002998	intron	0	Hs.1501	SDC2
rs10508616	10	20502090	0.1458	95.09%	0.137900	0.707700	0.00689800	0.00681300	NM_032812	intron	0	Hs.658134	PLXDC2
rs2367979	10	26020563	0.2305	94.39%	0.605400	0.810000	0.00694800	0.04039000	NM_020752	downstream	89402	Hs.499108	GPR158
rs10496139	2	67116647	0.1913	97.19%	0.699900	1.000000	0.00695600	0.01903000	NM_019002	upstream	361299	Hs.353022	ETAA1
rs1445442	12	63577561	0.3040	97.54%	0.046420	0.134600	0.00696600	0.00437300	ENST00000229088	downstream	19211	---	---
rs958404	7	133247726	0.3004	97.54%	0.256200	0.619000	0.00696900	0.02887000	NM_001037126	intron	0	Hs.321273	EXOC4
rs4603704	2	143033654	0.0758	97.19%	0.660700	0.676500	0.00697200	0.00586300	NM_001032998	upstream	318011	Hs.470126	KYNU
rs1972326	5	90461165	0.0141	87.37%	1.000000	1.000000	0.00697400	0.06541000	NM_032119	intron	0	Hs.591777	GPR98
rs2181302	13	105842361	0.4004	93.33%	0.609200	0.760500	0.00697800	0.03170000	ENST00000310792	upstream	33761	---	---
rs577953	1	109897420	0.0850	88.77%	0.401700	0.414100	0.00697900	0.00502800	NM_006496	intron	0	Hs.73799	GNAI3
rs9308871	2	104033366	0.2907	94.74%	0.554800	1.000000	0.00699700	0.00353700	NM_144632	downstream	1233056	Hs.436203	TMEM182
rs6438689	3	123148392	0.2384	97.89%	0.068790	1.000000	0.00700100	0.00198500	NM_021082	downstream	5245	Hs.518089	SLC15A2
rs890945	5	157924801	0.2882	71.23%	0.232000	0.081610	0.00700200	0.01409000	NM_024007	downstream	133896	Hs.657753	EBF1
rs3923935	1	219509198	0.3447	92.63%	0.220300	0.519700	0.00702900	0.06145000	NM_001010913	downstream	60847	---	---
rs10491511	9	103219769	0.0769	95.79%	0.663900	0.679600	0.00706300	0.03064000	NM_000035	downstream	2912	Hs.530274	ALDOB
rs334357	9	100949574	0.1808	95.09%	0.216400	0.180800	0.00706800	0.00649900	NM_004612	intron	0	Hs.494622	TGFBR1
rs1409298	10	92088003	0.0309	90.88%	1.000000	1.000000	0.00707000	0.03948000	ENST00000387911	upstream	174306	---	---
rs10489284	1	170633685	0.1757	96.84%	1.000000	0.823700	0.00708200	0.04464000	NM_015569	intron	0	Hs.654775	DNM3
rs285047	13	97555091	0.0706	94.39%	0.032060	0.331800	0.00708300	0.14730000	NM_001001715	upstream	38344	Hs.403917	FARP1
rs724201	12	70242280	0.4173	97.54%	0.805400	0.293000	0.00710700	0.00492300	NM_003667	intron	0	Hs.658889	LGR5
rs2247166	12	89185397	0.2410	97.54%	0.138300	0.261300	0.00711600	0.01965000	ENST00000388111	downstream	540467	---	---
rs968432	20	4690282	0.0136	90.18%	1.000000	1.000000	0.00712400	0.02608000	NM_170774	downstream	18387	Hs.631504	RASSF2
rs10519856	5	125201023	0.2315	94.74%	0.608300	1.000000	0.00713200	0.01681000	NM_023927	upstream	585977	Hs.363558	GRAMD3
rs10518144	4	77256055	0.2281	80.00%	0.258000	0.531700	0.00718300	0.00509200	NM_017426	intron	0	Hs.430435	NUP54
rs4129221	3	29367167	0.2419	97.89%	0.329300	0.165000	0.00720300	0.03768000	NM_001003792	intron	0	Hs.696468	RBMS3
rs10484613	6	99587626	0.0709	94.04%	0.032460	0.333400	0.00720700	0.01522000	NM_012160	upstream	85056	Hs.536850	FBXL4
rs7319671	13	36923224	0.4135	93.33%	0.800400	1.000000	0.00720700	0.00062650	NM_006475	downstream	111502	Hs.136348	POSTN
rs1445324	11	26991184	0.3989	93.68%	0.702700	0.740300	0.00720800	0.03391000	NM_203371	downstream	15978	Hs.705612	FIBIN
rs1882375	2	170447886	0.3421	80.00%	0.306700	0.116800	0.00721000	0.04024000	NM_172070	upstream	45628	---	UBR3
rs4252894	4	78196595	0.1213	82.46%	1.000000	0.614100	0.00721300	0.00204600	NM_006835	intron	0	Hs.518827	CCNI
rs452582	9	10089873	0.4722	94.74%	0.465800	0.215100	0.00722000	0.00124500	ENST00000363183	downstream	657521	---	---
rs1114967	14	20257888	0.3076	97.54%	0.888000	0.850200	0.00723600	0.02763000	NM_194430	downstream	19291	---	RNASE4
rs10275018	7	27475922	0.4048	88.42%	0.513300	1.000000	0.00724000	0.02531000	NM_152740	downstream	55666	Hs.406758	HIBADH
rs1667638	8	100749076	0.2805	86.32%	0.017650	0.009842	0.00724100	0.04040000	NM_017890	intron	0	Hs.191540	VPS13B
rs184461	18	36538715	0.1481	94.74%	0.332000	0.798800	0.00725500	0.00824800	NM_002647	upstream	1250482	Hs.464971	PIK3C3
rs1822202	15	60599740	0.4826	90.53%	0.620000	0.443800	0.00729000	0.02530000	NM_015059	upstream	127062	Hs.569438	TLN2
rs9294584	6	94742375	0.4384	96.84%	0.902700	1.000000	0.00732600	0.02095000	ENST00000364082	downstream	96709	---	---
rs1825754	8	100574413	0.3513	97.89%	0.358500	0.053480	0.00732800	0.04643000	NM_017890	intron	0	Hs.191540	VPS13B

rs10516485	4	102971488	0.0260	94.39%	0.159300	1.000000	0.00733200	0.01176000	NM_017935	intron	0	Hs.480400	BANK1
rs9308363	16	84481566	0.4094	92.98%	0.446200	0.249600	0.00733500	0.03355000	NM_002163	upstream	8709	Hs.137427	IRF8
rs7948646	11	84463639	0.2754	96.84%	0.880400	0.401100	0.00734100	0.00909200	ENST00000376104	intron	0	---	---
rs4131826	1	159549008	0.3082	97.89%	0.888700	0.849900	0.00737800	0.05682000	NM_000530	upstream	2646	Hs.591486	MPZ
rs1899727	15	50927337	0.0935	91.93%	0.000510	0.000439	0.00738000	0.04198000	NM_004498	upstream	57836	Hs.658573	ONECUT1
rs1013997	11	91957013	0.1534	92.63%	1.000000	0.299000	0.00739500	0.01629000	ENST00000298047	intron	0	---	---
rs10517685	4	159252777	0.0372	94.39%	0.043470	1.000000	0.00739600	0.01461000	NM_016613	downstream	15232	Hs.567498	C4orf18
rs10508850	10	36950870	0.2757	92.28%	0.440100	1.000000	0.00741100	0.01022000	ENST00000384541	downstream	4081	---	---
rs2073515	6	16767140	0.0130	94.74%	1.000000	1.000000	0.00741100	0.00286600	NM_000332	intron	0	Hs.434961	ATXN1
rs729453	9	22681708	0.4938	84.56%	0.001915	0.053940	0.00741700	0.03282000	NM_022160	downstream	239236	Hs.371976	DMRTA1
rs10488172	7	132985716	0.3858	89.12%	0.146000	0.301100	0.00743900	0.01133000	NM_001037126	intron	0	Hs.321273	EXOC4
rs2333467	4	177593682	0.1953	97.89%	0.570900	1.000000	0.00746100	0.12210000	NM_021928	downstream	106344	Hs.42194	SPCS3
rs10490607	2	21372683	0.1264	97.19%	0.169600	0.222900	0.00746600	0.04159000	ENST00000317039	upstream	152662	---	---
rs9307199	4	77970671	0.3272	95.44%	0.411900	0.199100	0.00748500	0.00184800	NM_020859	downstream	50705	Hs.702168	SHROOM3
rs505974	3	191389467	0.2108	94.04%	0.141500	0.434800	0.00748700	0.01268000	NM_021101	downstream	116730	Hs.439060	CLDN1
rs1150078	6	111666994	0.4728	96.84%	0.053000	0.359600	0.00749000	0.01580000	NM_018593	downstream	15156	Hs.591327	SLC16A10
rs9320930	6	123724727	0.3700	95.79%	0.363700	0.730400	0.00750700	0.00295700	NM_006073	intron	0	Hs.654601	TRDN
rs39061	7	29222789	0.4093	94.74%	0.256900	0.332300	0.00750800	0.00885000	NM_004067	intron	0	Hs.654611	CHN2
rs3922878	16	85412205	0.2303	80.00%	0.851700	0.599500	0.00757800	0.00956400	NM_005250	downstream	239409	Hs.533830	FOXL1
rs477086	5	131312509	0.3212	96.14%	0.211800	0.259000	0.00758500	0.00637900	ENST00000296869	intron	0	---	---
rs1459320	8	21181308	0.2849	90.53%	0.067050	0.414300	0.00761300	0.02489000	ENST00000387614	upstream	318944	---	---
rs1344870	3	21282405	0.3566	85.61%	0.026110	0.043130	0.00763400	0.00119700	ENST00000295509	upstream	140119	---	---
rs4924292	15	36793190	0.4345	93.68%	1.000000	1.000000	0.00764100	0.02822000	NM_207444	downstream	13659	Hs.448785	C15orf53
rs4710239	6	63789613	0.3697	91.58%	0.594900	0.205200	0.00765200	0.00174800	ENST00000356170	upstream	189808	---	---
rs7841308	8	59092187	0.0865	91.23%	1.000000	1.000000	0.00765200	0.02296000	NM_147189	intron	0	Hs.154652	FAM110B
rs1331905	9	28390284	0.4307	93.68%	0.709300	0.874000	0.00765800	0.01982000	NM_152570	intron	0	Hs.699432	LINGO2
rs7947527	11	5975218	0.4964	96.49%	0.809400	0.762600	0.00766700	0.05513000	NM_001005179	downstream	4639	Hs.553571	OR56A4
rs1820677	5	134079303	0.0663	97.89%	0.618300	1.000000	0.00767000	0.05152000	ENST00000265341	intron	0	---	---
rs251170	5	62062829	0.1614	88.07%	0.244700	0.137100	0.00767500	0.00133600	NM_016338	downstream	103928	Hs.482269	IPO11
rs2199862	4	58437185	0.1343	94.04%	0.287600	0.174100	0.00767900	0.03654000	ENST00000387903	upstream	383889	---	---
rs2135046	5	170034318	0.3615	97.54%	0.242200	0.120200	0.00768700	0.05982000	NM_001034838	intron	0	Hs.484111	KCNIP1
rs2355723	2	170890612	0.1804	89.47%	0.523200	0.129200	0.00769200	0.02532000	NM_138995	intron	0	Hs.671900	MYO3B
rs1387434	8	118472448	0.3582	94.04%	0.144100	0.213300	0.00769600	0.00769400	NM_080651	upstream	129763	Hs.492612	MED30
rs10489058	4	15967050	0.0940	93.33%	0.000634	0.003573	0.00770300	0.00500600	NM_152684	downstream	127667	---	FLJ39653
rs10501715	11	89599131	0.1367	93.68%	0.437000	0.173100	0.00771700	0.01180000	NM_012124	upstream	3304	Hs.22857	CHORDC1
rs7579207	2	115598322	0.3075	92.98%	0.885300	0.440100	0.00772300	0.26990000	NM_001004360	upstream	37949	Hs.591555	DPP10
rs9295647	6	25201412	0.1624	95.09%	0.500800	1.000000	0.00773600	0.01847000	ENST00000377993	intron	0	---	---
rs10504744	8	82952758	0.3563	94.04%	1.000000	0.727100	0.00774000	0.01931000	NM_152837	upstream	35768	Hs.492121	SNX16
rs1991488	5	125189097	0.4745	96.49%	0.809300	0.878400	0.00775600	0.00537100	NM_023927	upstream	597903	Hs.363558	GRAMD3
rs10494898	1	206664604	0.1129	97.89%	0.367900	0.754300	0.00775900	0.06269000	NM_025179	upstream	180316	Hs.497626	PLXNA2
rs10498235	2	229824279	0.1352	94.74%	0.294100	0.266300	0.00777200	0.01185000	NM_017933	intron	0	Hs.409352	PID1
rs1455158	2	138490108	0.3071	93.68%	0.313300	0.333700	0.00777700	0.00979800	NM_006895	3UTR	0	Hs.42151	HNMT
rs2332283	14	69827532	0.1601	97.54%	1.000000	0.177200	0.00778300	0.02256000	NM_016468	downstream	34323	Hs.443661	C14orf112
rs731700	14	58334879	0.4600	96.49%	0.715600	1.000000	0.00778600	0.01991000	NM_014992	upstream	390273	Hs.654934	DAAM1
rs2868134	4	82235245	0.1748	79.30%	0.103300	0.045300	0.00778900	0.07371000	NM_006259	intron	0	Hs.570833	PRKG2
rs1374122	2	4776973	0.0288	97.54%	1.000000	1.000000	0.00779200	0.00759700	ENST00000387792	downstream	75493	---	---
rs10520436	4	180956420	0.2294	89.47%	0.004013	0.005309	0.00780100	0.12870000	ENST00000385763	downstream	686534	---	---
rs1974307	13	103517148	0.4257	94.39%	0.384600	0.750300	0.00780500	0.00622500	NM_000452	upstream	999951	Hs.194783	SLC10A2
rs3925061	4	27819126	0.1888	84.56%	0.000563	0.025580	0.00781000	0.00883100	ENST00000387335	upstream	502793	---	---
rs7118119	11	84465202	0.2798	97.19%	0.765300	0.539200	0.00781300	0.00780700	ENST00000376104	intron	0	---	---
rs6956693	7	145069659	0.1807	96.14%	0.839200	1.000000	0.00782400	0.00746300	ENST00000385572	upstream	255695	---	---
rs10491773	9	14462090	0.0535	95.09%	0.546600	1.000000	0.00782600	0.00877100	NM_178566	downstream	143440	Hs.649522	ZDHHC21
rs10497355	2	170582455	0.3682	97.19%	1.000000	0.440700	0.00783200	0.02012000	NM_172070	intron	0	---	UBR3
rs4471302	1	170633760	0.1792	97.89%	0.839500	1.000000	0.00786300	0.04698000	NM_015569	intron	0	Hs.654775	DNM3

rs10508753	10	30293881	0.0130	94.74%	1.000000	1.000000	0.00787900	0.02373000	NM_021738	upstream	329974	Hs.499209	SVIL
rs1980892	1	193060259	0.4568	93.33%	0.009576	0.060340	0.00788900	0.08949000	NM_198503	downstream	1401277	Hs.657046	KCNT2
rs243027	2	60460511	0.4766	97.54%	0.399700	0.551200	0.00790600	0.00160200	ENST00000363937	upstream	4733	---	---
rs9313729	5	175195244	0.1807	87.37%	0.204200	0.095530	0.00790800	0.03731000	NM_001008220	intron	0	Hs.193235	CPLX2
rs1455157	2	138490222	0.3104	94.39%	0.393400	0.449200	0.00792100	0.01098000	NM_006895	3UTR	0	Hs.42151	HNMT
rs1380703	2	57794791	0.3225	96.84%	0.782700	0.852700	0.00794000	0.01593000	NM_006296	upstream	332433	Hs.631890	VRK2
rs10496491	2	115836547	0.2883	96.14%	0.555600	0.417100	0.00794400	0.04808000	NM_001004360	intron	0	Hs.591555	DPP10
rs10494572	1	182250203	0.2518	97.54%	0.749900	0.585800	0.00796900	0.04427000	NM_015101	intron	0	Hs.387995	GLT25D2
rs9327369	5	125247640	0.1989	97.89%	0.261400	0.273000	0.00798400	0.04165000	NM_023927	upstream	539360	Hs.363558	GRAMD3
rs1500737	10	57503439	0.4720	94.04%	0.541800	0.294100	0.00798600	0.01213000	NM_001005413	downstream	284471	Hs.591363	ZWINT
rs759572	12	96007166	0.4352	94.74%	0.264700	0.097470	0.00799500	0.01622000	ENST00000365498	upstream	109628	---	---
rs1332966	1	168553373	0.2527	97.19%	0.055020	0.660000	0.00800200	0.02074000	NM_152281	upstream	214521	Hs.183702	SCYL1BP1
rs323744	5	86861304	0.3760	86.32%	0.343200	0.595900	0.00800400	0.02545000	NM_153354	downstream	665477	Hs.379972	TMEM161B
rs10506132	12	37690866	0.3297	96.84%	0.415400	0.715800	0.00800600	0.01426000	NM_153634	upstream	105166	Hs.40910	CPNE8
rs325815	5	41157377	0.3800	96.49%	1.000000	0.236900	0.00800700	0.02167000	NM_000065	downstream	20716	Hs.481992	C6
rs334958	3	18585681	0.2248	97.54%	0.226800	0.329700	0.00801600	0.00500600	NM_002971	upstream	130473	Hs.517717	SATB1
rs601441	5	131312332	0.3231	97.19%	0.170000	0.142700	0.00802100	0.00525600	ENST00000296869	intron	0	---	---
rs952506	4	102201934	0.2222	97.89%	0.728500	0.846300	0.00804100	0.00659900	NM_000944	intron	0	Hs.435512	PPP3CA
rs1562489	3	135888563	0.2390	88.07%	0.117500	0.175700	0.00805700	0.00110700	NM_178554	upstream	36009	Hs.146730	KY
rs1952863	14	97967839	0.1092	91.58%	0.099160	0.074530	0.00806700	0.01015000	NM_182560	upstream	279864	Hs.448754	C14orf177
rs2860940	2	77154619	0.3556	94.74%	0.506300	0.435900	0.00807900	0.00488100	NM_024993	intron	0	Hs.285782	LRRTM4
rs1595423	2	78182256	0.0771	88.77%	0.009362	0.012060	0.00808200	0.01427000	ENST00000325094	downstream	311166	---	---
rs340686	5	8440200	0.2183	94.04%	0.281100	0.548400	0.00808200	0.04058000	ENST00000387188	downstream	232017	---	---
rs10515849	5	162538010	0.3746	97.89%	0.007060	0.014150	0.00808300	0.03881000	NM_199246	upstream	259145	Hs.79101	CCNG1
rs2044673	9	22697790	0.4748	97.54%	0.228700	0.878700	0.00808800	0.01933000	NM_022160	downstream	255318	Hs.371976	DMRTA1
rs7724015	5	160137590	0.1029	97.19%	0.750300	1.000000	0.00808900	0.01072000	ENST00000327245	intron	0	---	---
rs2392727	8	128148778	0.3614	87.37%	0.040210	0.420600	0.00809600	0.00274300	ENST00000385818	downstream	395697	---	---
rs2689138	1	237079798	0.1370	94.74%	0.126100	0.426400	0.00809700	0.08397000	NM_000740	upstream	779214	Hs.7138	CHRM3
rs10500376	16	10591252	0.0744	91.93%	0.158500	0.657500	0.00811600	0.05851000	NM_001424	upstream	9212	Hs.655130	EMP2
rs1468322	17	65314774	0.1900	95.09%	0.845800	1.000000	0.00813300	0.05391000	NM_002758	downstream	263707	Hs.463978	MAP2K6
rs311365	19	52162376	0.4424	94.39%	0.620800	0.271300	0.00814600	0.01219000	NM_004491	intron	0	Hs.509447	GRLF1
rs810123	13	104121974	0.0302	92.98%	1.000000	1.000000	0.00816300	0.03131000	NM_172370	upstream	794619	Hs.381382	DAOA
rs7077278	10	901291	0.1973	91.58%	0.246500	0.157700	0.00817400	0.09677000	NM_015155	intron	0	Hs.705392	LARP5
rs6975590	7	54443549	0.2185	94.74%	0.032480	0.164300	0.00820400	0.01424000	NM_182546	upstream	134086	Hs.335933	VSTM2A
rs10494643	1	189036417	0.2148	94.74%	0.010450	0.107900	0.00820600	0.03774000	NM_199051	upstream	323035	Hs.65765	FAM5C
rs1327990	6	4259879	0.2019	91.23%	0.120800	0.523900	0.00822500	0.04344000	ENST00000363028	upstream	86118	---	---
rs10501714	11	89593029	0.1643	97.19%	0.025600	0.003822	0.00823100	0.03719000	NM_012124	intron	0	Hs.22857	CHORDC1
rs10503122	18	64600521	0.0252	97.54%	0.154400	1.000000	0.00825800	0.06613000	NM_024781	upstream	15829	Hs.280781	CCDC102B
rs2253624	17	67243676	0.0135	90.88%	0.040230	1.000000	0.00826600	0.07774000	ENST00000334565	downstream	114504	---	---
rs8060446	16	81043837	0.1729	94.39%	0.670300	0.535700	0.00827100	0.04382000	NM_005792	upstream	282524	Hs.344400	MPHOSPH6
rs1393516	3	59418742	0.2077	95.44%	0.585700	1.000000	0.00828400	0.00545700	NM_002012	downstream	294250	Hs.655995	FHIT
rs4128166	9	120331586	0.4635	91.23%	0.900900	0.349500	0.00829800	0.00682700	NM_014618	downstream	637143	Hs.532316	DBC1
rs1368086	2	43818511	0.2967	95.79%	0.020930	0.326500	0.00830800	0.01322000	NM_172069	intron	0	Hs.164162	PLEKHH2
rs810134	13	31658874	0.2656	89.82%	0.056190	0.203300	0.00831100	0.03033000	NM_023037	intron	0	Hs.591225	FRY
rs10497117	2	153431463	0.4428	95.09%	0.141900	0.278200	0.00833500	0.00962400	NM_152522	downstream	105526	Hs.516468	ARL6IP6
rs684080	4	74978908	0.3856	82.81%	0.274800	0.490600	0.00836300	0.02631000	NM_001511	downstream	10659	Hs.789	CXCL1
rs10503847	8	29450387	0.2036	88.77%	0.243400	0.199200	0.00839000	0.02633000	ENST00000385149	downstream	114596	---	---
rs1423417	5	41175549	0.3788	92.63%	1.000000	0.236700	0.00839400	0.06758000	NM_000065	downstream	2544	Hs.481992	C6
rs2216081	2	64805405	0.0701	97.54%	0.006223	0.328600	0.00842000	0.02977000	NM_014755	upstream	70855	Hs.693696	SERTAD2
rs6776964	3	190787296	0.4225	95.09%	0.171200	0.222300	0.00844600	0.01486000	NM_003722	upstream	44614	Hs.137569	TP63
rs10508570	10	19228576	0.0963	94.74%	0.286400	1.000000	0.00845000	0.03487000	NM_178815	downstream	221630	Hs.25362	ARL5B
rs10491833	9	24710232	0.1896	94.39%	0.169400	0.376700	0.00845500	0.00625200	ENST00000380098	upstream	175997	---	---
rs1224703	6	58511977	0.4373	89.47%	0.202900	0.283100	0.00846900	0.00572700	NM_206908	upstream	116322	---	GUSBL2
rs10502714	18	34744797	0.0921	97.19%	0.713400	0.562100	0.00848100	0.01020000	NM_020180	upstream	1344856	Hs.435976	BRUNOL4

rs1793696	8	118393066	0.3083	88.77%	0.142400	0.173100	0.00849200	0.08381000	NM_173851	downstream	134935	Hs.532270	SLC30A8
rs1865235	2	173975594	0.1313	97.54%	0.440200	0.576700	0.00850100	0.02619000	NM_145810	downstream	33629	Hs.470654	CDCA7
rs794143	4	110172663	0.4686	95.09%	0.010570	0.169300	0.00851400	0.00785700	NM_032518	intron	0	Hs.658842	COL25A1
rs1820008	5	165267267	0.0251	97.89%	1.000000	1.000000	0.00851500	0.01358000	ENST00000385726	downstream	297596	---	---
rs10493858	1	92060525	0.1318	97.19%	0.797500	0.781000	0.00853100	0.02461000	NM_003243	intron	0	Hs.482390	TGFBR3
rs3887137	9	106738433	0.1352	94.74%	0.294100	0.413900	0.00853300	0.02735000	NM_005502	upstream	8094	Hs.429294	ABCA1
rs923887	11	96543926	0.2292	97.19%	0.397300	0.813300	0.00854300	0.01455000	ENST00000362445	upstream	489748	---	---
rs7725707	5	81866327	0.1158	90.88%	0.357500	0.620000	0.00854900	0.04903000	NM_001039779	downstream	148236	---	FLJ41309
rs1014313	8	90096817	0.3327	97.54%	0.501300	0.585900	0.00855100	0.01269000	NM_003821	upstream	742356	Hs.103755	RIPK2
rs10490881	3	190178441	0.0132	93.33%	0.039170	1.000000	0.00856700	0.06022000	NM_005578	downstream	103480	Hs.444362	LPP
rs10493158	1	51787780	0.0746	94.04%	0.169100	0.203600	0.00856900	0.12090000	NM_148909	upstream	67346	Hs.21938	OSBPL9
rs717827	2	40351005	0.3989	97.19%	0.706800	0.628300	0.00857000	0.01756000	NM_021097	intron	0	Hs.468274	SLC8A1
rs4783222	16	81140611	0.3927	91.58%	0.605200	0.158600	0.00857500	0.01103000	NM_001257	upstream	308849	Hs.654386	CDH13
rs1601468	8	25977411	0.2151	95.44%	0.371800	1.000000	0.00858200	0.12280000	ENST00000380737	intron	0	---	---
rs275737	15	37966305	0.1980	85.96%	0.014380	0.145800	0.00859000	0.03199000	NM_007223	intron	0	Hs.37196	GPR176
rs10494506	1	176266337	0.1720	87.72%	0.181600	0.743200	0.00859100	0.00280100	ENST00000308284	intron	0	---	---
rs10484611	6	99451405	0.0699	97.89%	0.034130	0.328600	0.00860100	0.01696000	NM_012160	intron	0	Hs.536850	FBXL4
rs6807625	3	20808822	0.1846	97.89%	0.691300	0.770500	0.00861800	0.00894800	ENST00000384080	upstream	284570	---	---
rs1454796	8	115768911	0.1985	93.68%	0.847800	0.831500	0.00861900	0.10390000	NM_014112	downstream	725060	Hs.657018	TRPS1
rs7069064	10	58104365	0.2068	97.54%	0.042460	0.038910	0.00862100	0.09252000	NM_001005414	upstream	313325	---	ZWINT
rs10506868	10	114309370	0.0252	97.54%	0.154400	1.000000	0.00866300	0.03300000	NM_145206	intron	0	Hs.194554	VTI1A
rs6758008	2	56564993	0.4188	95.09%	0.901100	1.000000	0.00866400	0.00457900	NM_006296	upstream	1562231	Hs.631890	VRK2
rs3764131	13	48721288	0.2898	92.63%	0.374100	0.686700	0.00868300	0.04240000	NM_030911	intron	0	Hs.388220	CDADC1
rs10508087	13	101918278	0.1853	97.54%	0.321900	0.248600	0.00869200	0.00785600	NM_175929	upstream	66122	Hs.696392	FGF14
rs10512976	5	8722512	0.0401	91.93%	0.341100	1.000000	0.00870200	0.01649000	NM_003966	downstream	373497	Hs.27621	SEMA5A
rs9309078	2	42103394	0.1435	83.16%	0.013450	0.087680	0.00871400	0.00937100	NM_138370	upstream	25863	Hs.408542	LOC91461
rs10483671	14	56135072	0.0787	89.12%	0.055040	0.067090	0.00872600	0.04941000	NM_017799	intron	0	Hs.497253	C14orf101
rs10492461	13	29430661	0.0746	94.04%	0.169100	1.000000	0.00872600	0.01322000	NM_001010918	downstream	8036	---	LOC440131
rs10493371	1	65018226	0.1241	94.74%	0.577800	1.000000	0.00873200	0.01448000	NM_018211	intron	0	Hs.591443	RAVER2
rs2244882	13	34774714	0.1697	96.14%	1.000000	0.814400	0.00873700	0.03954000	NM_015678	intron	0	Hs.491172	NBEA
rs10491478	5	179301894	0.4066	95.79%	1.000000	0.443500	0.00873800	0.14720000	NM_018434	downstream	13217	Hs.484363	RNF130
rs1934511	1	161733144	0.1974	95.09%	1.000000	0.673700	0.00874100	0.08994000	ENST00000385703	upstream	12754	---	---
rs10493028	1	26425508	0.0630	94.74%	0.609300	1.000000	0.00875300	0.00689500	NM_022778	upstream	7772	Hs.63795	CCDC21
rs4799431	18	32558105	0.4757	93.68%	0.623900	1.000000	0.00875700	0.00779900	NM_025135	intron	0	Hs.436636	FHOD3
rs10508059	13	100730832	0.2681	82.46%	0.045970	0.020280	0.00876400	0.04411000	NM_052867	intron	0	Hs.525146	NALCN
rs2371231	5	65725859	0.1190	95.79%	0.778500	0.539800	0.00877300	0.06933000	NM_001001703	downstream	75850	---	FLJ46010
rs10497760	2	196017708	0.2005	65.61%	0.005148	0.086330	0.00877800	0.01121000	ENST00000365336	upstream	69299	---	---
rs17797113	4	56475111	0.0351	84.91%	0.252500	0.329400	0.00878300	0.02352000	NM_001024924	downstream	9110	Hs.269665	EXOC1
rs131	7	24905412	0.3874	91.93%	0.435900	0.637200	0.00878600	0.05288000	NM_145321	intron	0	Hs.520259	OSBPL3
rs6748458	2	98132982	0.1496	96.14%	0.091180	0.716700	0.00880300	0.08970000	NM_144992	intron	0	Hs.269977	VWA3B
rs1354757	4	135996282	0.4795	94.04%	0.273700	1.000000	0.00885200	0.03006000	ENST00000384459	downstream	459743	---	---
rs4672212	2	57546267	0.1448	90.88%	0.078460	0.233800	0.00886500	0.00233200	NM_006296	upstream	580957	Hs.631890	VRK2
rs10490072	2	60523435	0.1649	97.89%	0.131500	0.055910	0.00888000	0.05713000	NM_138553	downstream	8371	---	BCL11A
rs10512281	9	103207574	0.0809	97.54%	0.695400	1.000000	0.00888100	0.02732000	NM_197977	intron	0	Hs.50123	ZNF189
rs2898455	8	18031292	0.3271	94.39%	0.026340	0.141000	0.00888100	0.03600000	NM_004315	upstream	44535	Hs.527412	ASAH1
rs728366	11	123539663	0.2874	91.58%	0.033390	0.034040	0.00888300	0.00779000	ENST00000355343	downstream	4651	---	---
rs1983453	4	57903149	0.3345	97.54%	0.224600	0.029340	0.00888500	0.04412000	NM_001553	upstream	231841	Hs.479808	IGFBP7
rs10493369	1	64994244	0.1245	94.39%	0.270300	0.664100	0.00889500	0.01619000	NM_018211	intron	0	Hs.591443	RAVER2
rs4944558	11	85507515	0.2345	96.49%	0.738800	1.000000	0.00890700	0.02717000	ENST00000387875	downstream	24532	---	---
rs10515813	5	159960260	0.0742	82.81%	1.000000	1.000000	0.00892100	0.01846000	ENST00000327245	intron	0	---	---
rs10519101	15	45194109	0.3467	96.14%	0.062200	0.151900	0.00892100	0.00387000	ENST00000381028	downstream	19033	---	---
rs1975974	17	21631187	0.1791	94.04%	1.000000	0.501600	0.00895000	0.03284000	ENST00000344561	upstream	22813	---	---
rs10496383	2	104334920	0.0897	91.93%	0.445200	0.156800	0.00895900	0.02140000	NM_144632	downstream	1534610	Hs.436203	TMEM182
rs10507416	13	33279750	0.3806	94.04%	0.092450	0.000710	0.00896400	0.02669000	NM_181558	upstream	10484	Hs.115474	RFC3

rs1337788	13	97318472	0.2293	84.91%	0.000972	0.203000	0.00897900	0.03271000	NM_021033	downstream	400229	Hs.508480	RAP2A
rs6097963	20	52452442	0.0921	97.19%	0.265700	1.000000	0.00897900	0.01554000	NM_018431	upstream	73146	Hs.656582	DOK5
rs2213980	7	90227582	0.1470	97.89%	1.000000	0.299600	0.00898000	0.01376000	NM_012395	intron	0	Hs.430742	PFTK1
rs1113478	13	88908389	0.4212	91.23%	0.097630	0.022040	0.00899600	0.00838800	ENST00000298437	upstream	96599	---	---
rs360384	2	126837248	0.0809	97.54%	0.695400	1.000000	0.00900600	0.05764000	NM_016815	upstream	327296	Hs.59138	GYPC
rs1553251	15	67236901	0.3007	96.84%	0.045490	0.240200	0.00904400	0.01752000	NM_015554	upstream	3126	Hs.183006	GLCE
rs10517628	4	157113913	0.1511	97.54%	1.000000	0.476200	0.00906600	0.00998800	NM_001334	upstream	19415	Hs.75262	CTSO
rs10492609	13	31228179	0.2537	94.04%	0.035180	0.043380	0.00909100	0.04202000	NM_130806	intron	0	Hs.680763	RXFP2
rs10508272	10	4046871	0.0315	94.74%	0.228800	0.298600	0.00912100	0.05554000	NM_001300	upstream	229404	Hs.4055	KLF6
rs659793	11	109191077	0.0315	94.74%	0.228800	0.298600	0.00912100	0.08205000	NM_207645	downstream	389459	Hs.172982	LOC399947
rs965158	8	107564094	0.0315	94.74%	1.000000	1.000000	0.00912100	0.07557000	NM_181354	upstream	175176	Hs.148778	OXR1
rs9300636	13	100362458	0.4610	94.39%	0.087400	0.120600	0.00914000	0.00128000	NM_052867	downstream	141671	Hs.525146	NALCN
rs1283694	8	108481118	0.1864	97.89%	0.692100	0.772100	0.00914500	0.00271500	NM_001146	intron	0	Hs.369675	ANGPT1
rs10497941	2	211667103	0.2243	92.28%	0.859100	1.000000	0.00915000	0.11090000	NM_001042599	downstream	289482	Hs.390729	ERBB4
rs10499149	6	129425863	0.1149	91.58%	0.058010	0.120900	0.00917400	0.00856200	NM_000426	intron	0	Hs.200841	LAMA2
rs6060578	20	33768859	0.0914	97.89%	0.487900	0.562100	0.00918300	0.01390000	NM_184241	intron	0	---	RBM39
rs2056698	12	111053486	0.2841	95.09%	0.456400	0.548100	0.00922000	0.00510400	NM_006700	intron	0	Hs.5148	TRAFD1
rs6570896	6	149134450	0.2717	96.84%	0.022470	0.014450	0.00922000	0.01941000	NM_005715	intron	0	Hs.657370	UST
rs10507340	13	24185174	0.0725	96.84%	0.158500	1.000000	0.00922300	0.30750000	NM_001676	downstream	1265	Hs.147111	ATP12A
rs1710506	2	2497383	0.4170	97.19%	0.536300	0.650800	0.00922900	0.03113000	NM_015025	upstream	183331	Hs.434418	MYT1L
rs10497464	2	177644761	0.1039	97.89%	1.000000	0.286100	0.00923300	0.05446000	ENST00000385460	upstream	66927	---	---
rs10512498	17	58507111	0.0314	95.09%	1.000000	1.000000	0.00924300	0.02413000	ENST00000384821	downstream	131706	---	---
rs300330	5	152702916	0.1426	94.74%	0.317900	0.133900	0.00925800	0.01053000	NM_000827	upstream	147583	Hs.519693	GRIA1
rs10483305	14	25626745	0.3506	91.58%	0.417300	0.522000	0.00926500	0.00591100	ENST00000384946	downstream	84467	---	---
rs10500773	11	13128803	0.3996	94.39%	0.799900	0.743800	0.00929000	0.01071000	ENST00000386734	upstream	113244	---	---
rs10484386	6	22223919	0.0387	95.09%	1.000000	1.000000	0.00933100	0.00895400	ENST00000378559	upstream	5430	---	---
rs4879613	9	32727092	0.0387	95.09%	0.051910	1.000000	0.00933100	0.01545000	NM_212558	upstream	46904	Hs.522063	LOC401498
rs6890756	5	62147584	0.1209	97.19%	0.149200	0.368600	0.00933700	0.05169000	ENST00000381087	upstream	2785	---	---
rs6844637	4	113967887	0.3459	97.89%	0.233900	0.434300	0.00934500	0.02202000	ENST00000341244	CDS	0	---	---
rs1410093	13	72736628	0.4783	88.77%	0.077850	0.272100	0.00936100	0.01002000	NM_007249	downstream	421522	Hs.373857	KLF12
rs6938452	6	107071417	0.3992	88.77%	0.012610	0.162500	0.00937000	0.01354000	NM_001624	intron	0	Hs.643590	AIM1
rs10485714	20	7085322	0.0335	94.39%	0.254700	0.333400	0.00937300	0.05868000	NM_001200	downstream	376395	Hs.73853	BMP2
rs7127817	11	112131896	0.3030	92.63%	0.188900	0.128500	0.00938400	0.01033000	ENST00000388431	upstream	77972	---	---
rs10497624	2	183777265	0.0963	94.74%	0.024400	0.079880	0.00939000	0.04231000	NM_001008544	downstream	42615	---	NUP35
rs1440564	4	67883036	0.0858	94.04%	1.000000	1.000000	0.00939100	0.06252000	ENST00000383902	downstream	94869	---	---
rs10496133	2	66454940	0.0429	85.96%	0.360700	0.439600	0.00939600	0.01895000	NM_002398	upstream	61096	Hs.526754	MEIS1
rs3892760	5	168946565	0.0199	96.84%	0.096590	0.148000	0.00939700	0.99760000	NM_017785	intron	0	Hs.368710	CCDC99
rs1417693	6	63800373	0.3727	95.09%	0.037120	0.004745	0.00940100	0.00573700	ENST00000356170	upstream	179048	---	---
rs10503664	8	19646975	0.0646	95.09%	0.611700	0.615500	0.00940500	0.00403100	NM_018371	upstream	62423	Hs.655166	ChGn
rs1114626	5	21269294	0.0197	97.89%	1.000000	1.000000	0.00942400	0.99770000	NM_004934	upstream	1245198	Hs.317632	CDH18
rs10519705	5	121884370	0.0174	90.88%	1.000000	1.000000	0.00943400	0.09500000	NM_005460	downstream	56677	Hs.426463	SNCAIP
rs10496141	2	67116762	0.1888	97.54%	1.000000	1.000000	0.00944300	0.02531000	NM_019002	upstream	361184	Hs.353022	ETAA1
rs1395867	2	189236054	0.0883	93.33%	0.131900	0.452700	0.00945600	0.01599000	NM_016315	downstream	68023	Hs.470887	GULP1
rs661592	7	42919011	0.2490	89.47%	1.000000	0.821100	0.00945800	0.01801000	NM_024054	upstream	829	Hs.214247	C7orf25
rs4485440	18	34728093	0.0949	96.14%	0.720000	0.573100	0.00946600	0.01098000	NM_020180	upstream	1328152	Hs.435976	BRUNOL4
rs2077683	5	65683206	0.4122	97.89%	0.537300	0.748600	0.00946700	0.09019000	NM_001001703	downstream	33197	---	FLJ46010
rs2804284	9	582757	0.3302	94.04%	0.128000	0.190900	0.00946700	0.01019000	NM_015158	intron	0	Hs.306764	ANKRD15
rs241473	1	49545039	0.3390	93.68%	0.784600	0.857400	0.00947500	0.05091000	ENST00000334103	intron	0	---	---
rs4672995	2	221727827	0.0877	94.04%	0.129100	0.235100	0.00948000	0.03728000	NM_004438	downstream	263166	Hs.371218	EPHA4
rs200144	6	143385348	0.1111	94.74%	0.219800	0.206100	0.00949300	0.02704000	NM_016108	upstream	38358	Hs.567501	AIG1
rs714256	6	99558442	0.0871	92.63%	0.118700	0.127400	0.00950800	0.00510600	NM_012160	upstream	55872	Hs.536850	FBXL4
rs2035831	3	175166485	0.2048	79.65%	0.038990	0.039900	0.00951100	0.09133000	NM_014932	intron	0	Hs.478289	NLGN1
rs7561527	2	59956272	0.1076	88.07%	1.000000	1.000000	0.00951300	0.01358000	ENST00000386566	downstream	180782	---	---
rs2141859	12	103424111	0.4455	96.49%	0.625200	0.438500	0.00952000	0.00526400	NM_018413	intron	0	Hs.17569	CHST11

rs544283	5	39913976	0.3811	92.98%	0.516400	0.493100	0.00952100	0.01568000	NM_001343	upstream	453273	Hs.481980	DAB2
rs10508748	10	30297356	0.0126	97.54%	1.000000	1.000000	0.00952300	0.02449000	NM_021738	upstream	333449	Hs.499209	SVIL
rs10491156	17	50544682	0.0389	94.74%	1.000000	1.000000	0.00955900	0.02886000	NM_178509	intron	0	Hs.35199	STXBP4
rs10495496	2	4242708	0.0389	94.74%	1.000000	1.000000	0.00955900	0.03208000	ENST00000382050	downstream	513207	---	---
rs10493724	1	82945162	0.1654	93.33%	0.662900	0.520200	0.00958700	0.01987000	NM_012302	downstream	714467	Hs.24212	LPHN2
rs727989	7	86251533	0.1397	95.44%	0.038850	0.228600	0.00959000	0.01527000	NM_000840	intron	0	Hs.590575	GRM3
rs7781966	7	76385939	0.4176	97.89%	0.902700	0.227800	0.00959200	0.03839000	NM_152992	upstream	291440	Hs.488877	POMZP3
rs7707528	5	83572343	0.2169	95.44%	0.720500	0.419800	0.00960100	0.00510100	NM_005711	intron	0	Hs.482730	EDIL3
rs993736	2	83617449	0.2708	97.19%	0.446300	0.480000	0.00960100	0.00972000	ENST00000362425	upstream	120921	---	---
rs10483814	14	68125552	0.0870	94.74%	1.000000	1.000000	0.00960700	0.04015000	NM_133509	intron	0	Hs.172587	RAD51L1
rs442553	5	89355622	0.3402	93.33%	0.891800	0.468600	0.00961000	0.00577500	NM_004365	downstream	369665	Hs.591767	CETN3
rs2278913	2	39053384	0.0852	94.74%	0.704100	0.697300	0.00961100	0.00197400	NM_005633	downstream	13085	Hs.654397	SOS1
rs7931910	11	109749455	0.0852	94.74%	1.000000	1.000000	0.00961100	0.01186000	NM_004109	upstream	56349	Hs.744	FDX1
rs866096	1	221654438	0.1551	96.14%	0.818200	1.000000	0.00961700	0.00831000	NM_152610	downstream	19003	Hs.192090	C1orf65
rs10496699	2	133693475	0.4278	97.19%	0.623300	0.547200	0.00963900	0.02347000	NM_207481	intron	0	Hs.537329	NAP5
rs9311594	3	56107690	0.1079	97.54%	0.539700	1.000000	0.00964300	0.02712000	NM_015576	intron	0	Hs.476389	ERC2
rs150948	14	69605873	0.0545	93.33%	0.173100	0.215400	0.00965200	0.01459000	NM_182936	intron	0	Hs.337696	SLC8A3
rs2466025	8	128258556	0.3370	94.74%	1.000000	1.000000	0.00965500	0.00237400	ENST00000377972	downstream	453154	---	---
rs1877771	6	163238470	0.4767	90.53%	0.453700	0.345700	0.00965800	0.03807000	NM_152410	intron	0	Hs.25791	PACRG
rs1556541	13	37203414	0.4670	95.79%	0.330400	0.034770	0.00966500	0.07678000	NM_016179	intron	0	Hs.262960	TRPC4
rs8042584	15	21674230	0.0556	94.74%	0.188900	0.239800	0.00966500	0.04124000	NM_018958	upstream	797878	Hs.649663	C15orf2
rs1454836	15	45338346	0.4139	93.68%	0.530000	0.647100	0.00966600	0.00167600	ENST00000364974	upstream	13740	---	---
rs10496042	2	55453314	0.2603	93.68%	0.527400	0.275900	0.00967100	0.01584000	NM_018084	intron	0	Hs.292925	CCDC88A
rs10504464	8	71109183	0.1362	97.89%	0.798200	0.790100	0.00969400	0.02351000	NM_024504	downstream	17391	Hs.287532	PRDM14
rs1949532	3	168225976	0.3352	93.68%	0.680400	1.000000	0.00970000	0.05894000	ENST00000324656	upstream	40229	---	---
rs1859275	7	8552281	0.2917	88.42%	0.542500	0.409900	0.00970600	0.02473000	NM_152745	upstream	204882	Hs.487564	NXPH1
rs10495649	2	16283870	0.0542	97.19%	0.563900	1.000000	0.00972300	0.03593000	ENST00000388035	downstream	39825	---	---
rs2778	6	117716222	0.4355	97.89%	0.807900	0.436200	0.00976000	0.02200000	NM_002944	3UTR	0	Hs.1041	ROS1
rs2417232	12	12816227	0.4371	97.54%	0.145700	0.134200	0.00976200	0.01107000	NM_201224	intron	0	Hs.504828	DDX47
rs1195963	11	64698446	0.1945	96.49%	0.000004	0.008892	0.00976400	0.00999700	NM_001008778	downstream	1182	Hs.450642	SPDYC
rs10514960	5	63669697	0.1860	90.53%	0.216600	0.361200	0.00978200	0.00928400	ENST00000389100	intron	0	---	---
rs1071883	5	178068474	0.2726	97.19%	0.544100	0.598600	0.00978200	0.05287000	NM_005649	downstream	2663	Hs.484324	ZNF354A
rs10490070	2	60509183	0.1847	94.04%	0.152200	0.043380	0.00981400	0.04509000	NM_138553	downstream	22623	---	BCL11A
rs9314189	5	96547425	0.4638	96.84%	1.000000	0.764800	0.00981400	0.01259000	NM_018343	upstream	2705	Hs.27021	RIOK2
rs2209810	6	85421495	0.4528	81.75%	0.003526	0.007813	0.00982400	0.01107000	NM_014895	upstream	427462	Hs.485865	KIAA1009
rs515212	13	88955133	0.4022	95.09%	0.377400	0.104800	0.00984000	0.02166000	ENST00000298437	upstream	143343	---	---
rs10490442	2	34283829	0.1000	94.74%	0.736900	1.000000	0.00985300	0.01200000	ENST00000322472	upstream	477483	---	---
rs2050473	1	26426683	0.0679	92.98%	1.000000	1.000000	0.00985900	0.00408900	NM_022778	upstream	6597	Hs.63795	CCDC21
rs7559216	2	228888754	0.2632	93.33%	0.429500	0.717400	0.00986200	0.03463000	NM_030623	upstream	134168	Hs.436306	SPHKAP
rs1823777	11	84463859	0.2790	96.84%	0.764900	0.539200	0.00987600	0.00933000	ENST00000376104	intron	0	---	---
rs10512673	5	38030948	0.0125	97.89%	1.000000	1.000000	0.00987700	0.08925000	NM_199234	upstream	159598	Hs.248114	GDNF
rs2675045	2	183708388	0.3233	75.44%	0.008199	0.204500	0.00991200	0.03993000	NM_001008544	intron	0	---	NUP35
rs4236316	7	24949054	0.2981	94.74%	0.562000	0.435500	0.00991300	0.04796000	NM_145321	intron	0	Hs.520259	OSBPL3
rs726236	2	79428695	0.4549	97.19%	0.146600	0.178000	0.00991400	0.04152000	NM_138937	upstream	188308	Hs.567312	REG3A
rs10490203	2	102425669	0.1764	96.49%	0.678000	0.763400	0.00994800	0.05847000	NM_003853	intron	0	Hs.158315	IL18RAP
rs9299988	11	25250805	0.0181	87.02%	1.000000	1.000000	0.00996200	0.04600000	NM_001009909	downstream	194025	Hs.144138	LUZP2
rs6507838	18	44089985	0.2315	94.74%	0.393000	0.847700	0.00996500	0.05590000	ENST00000324663	downstream	56779	---	---
rs7570682	2	104349699	0.2315	94.74%	0.170000	0.052130	0.00996500	0.02206000	NM_144632	downstream	1549389	Hs.436203	TMEM182
rs10483892	14	77692887	0.1161	93.68%	0.064060	0.134400	0.00996700	0.00643700	NM_020421	downstream	222840	Hs.413208	ADCK1
rs10520767	15	93180541	0.1996	88.77%	0.073670	0.401500	0.00996800	0.02565000	NM_018349	downstream	355905	Hs.592017	MCTP2
rs726788	19	14604245	0.4981	91.23%	0.710800	0.437600	0.00997700	0.00307700	NM_152939	intron	0	---	EMR3
rs10485987	7	21875489	0.4474	93.33%	0.001865	0.015230	0.00998400	0.09924000	NM_003777	intron	0	Hs.655326	DNAH11
rs7591631	2	175000572	0.2192	96.84%	0.051800	0.320700	0.00998800	0.00867400	NM_024583	intron	0	Hs.470679	SCRN3
rs950591	12	126238381	0.4837	96.84%	0.118800	0.366400	0.00999900	0.03230000	ENST00000386224	upstream	21441	---	---

rs10504091	8	50923329	0.0762	94.39%	1.000000	0.401300	0.01001000	0.01733000	NM_018967	upstream	545997	Hs.584914	SNTG1
rs9320599	6	117847159	0.0762	94.39%	0.187200	0.077330	0.01001000	0.01219000	NM_002944	intron	0	Hs.1041	ROS1
rs10495660	2	17260029	0.4909	96.84%	0.228100	0.096610	0.01002000	0.06150000	NM_003385	upstream	325260	Hs.444212	VSNL1
rs10514949	5	62068672	0.4462	97.89%	0.184600	0.757000	0.01002000	0.01145000	NM_016338	downstream	109771	Hs.482269	IPO11
rs10496360	2	101786324	0.2748	91.93%	0.756300	1.000000	0.01004000	0.02291000	NM_145687	intron	0	Hs.431550	MAP4K4
rs226984	14	44721431	0.1852	94.74%	0.543500	0.251000	0.01004000	0.01965000	NM_020937	intron	0	Hs.509229	FANCM
rs7825740	8	33108634	0.0131	93.68%	1.000000	1.000000	0.01004000	0.03375000	ENST00000387861	upstream	114317	---	---
rs10494486	1	168565078	0.2388	94.04%	0.063500	0.815900	0.01005000	0.02282000	NM_152281	upstream	202816	Hs.183702	SCYL1BP1
rs763650	16	7669222	0.4477	97.19%	0.716500	1.000000	0.01005000	0.00892900	NM_018723	intron	0	Hs.459842	A2BP1
rs10499290	6	156474099	0.0916	91.93%	1.000000	1.000000	0.01006000	0.04119000	ENST00000384945	downstream	32150	---	---
rs3797027	4	186531278	0.0829	74.04%	0.638800	0.653300	0.01006000	0.07692000	NM_018409	intron	0	Hs.558513	LRP2BP
rs491544	18	63676351	0.2910	94.04%	0.235300	0.687800	0.01008000	0.15840000	NM_032160	upstream	341404	Hs.124673	DSEL
rs682913	5	39932960	0.3801	86.32%	0.021920	0.280700	0.01009000	0.02059000	NM_001343	upstream	472257	Hs.481980	DAB2
rs10496540	2	117922404	0.0629	97.54%	1.000000	0.264800	0.01010000	0.03889000	NM_006773	upstream	366321	Hs.363492	DDX18
rs7100162	10	1972527	0.1859	94.39%	0.691200	0.260600	0.01010000	0.02696000	NM_018702	upstream	202857	Hs.657984	ADARB2
rs1125607	7	19260655	0.4427	97.89%	0.904000	1.000000	0.01011000	0.00400000	NM_152898	upstream	109145	Hs.592168	FERD3L
rs9316695	13	53513774	0.2116	93.68%	0.271300	1.000000	0.01011000	0.01990000	ENST00000258651	downstream	399066	---	---
rs1875403	15	52909283	0.1461	93.68%	0.045860	0.063140	0.01012000	0.00853500	NM_016304	downstream	351530	Hs.274772	C15orf15
rs4128767	15	79330462	0.3755	97.19%	0.444100	0.866700	0.01012000	0.01330000	NM_172217	intron	0	Hs.459095	IL16
rs10508261	10	3723731	0.3626	91.93%	0.689100	0.749600	0.01013000	0.04354000	NM_001300	downstream	84457	Hs.4055	KLF6
rs10492087	12	100769604	0.2808	96.84%	0.882400	0.540400	0.01014000	0.10540000	ENST00000386360	downstream	14787	---	---
rs10514652	3	3533527	0.1648	94.74%	0.185100	0.094320	0.01014000	0.04343000	NM_020873	upstream	283023	Hs.163244	LRRN1
rs9304121	18	29297256	0.3136	97.89%	0.126600	0.411900	0.01014000	0.03483000	NM_198995	upstream	22573	Hs.115461	C18orf34
rs10517057	4	42595319	0.4658	97.54%	0.547000	0.222200	0.01016000	0.01988000	NM_006095	upstream	241452	Hs.435052	ATP8A1
rs639441	5	39889373	0.2780	97.19%	0.653600	0.605800	0.01016000	0.03750000	NM_001343	upstream	428670	Hs.481980	DAB2
rs2400502	5	147615238	0.0178	88.77%	1.000000	1.000000	0.01017000	0.03364000	NM_205841	downstream	40346	Hs.334274	SPINK6
rs10500692	11	7285272	0.2742	97.89%	0.652100	0.682300	0.01019000	0.02574000	NM_175733	intron	0	Hs.177193	SYT9
rs1873039	3	174993986	0.2770	97.54%	0.880900	0.150300	0.01019000	0.01255000	NM_014932	intron	0	Hs.478289	NLGN1
rs10492161	12	106442173	0.1479	93.68%	0.226100	0.298100	0.01020000	0.05256000	NM_152322	intron	0	---	BTBD11
rs10501111	11	29101598	0.0722	97.19%	0.640700	0.363800	0.01020000	0.01157000	NM_152636	downstream	790079	Hs.243326	METT5D1
rs1535563	13	25166265	0.3397	82.11%	0.002045	0.020140	0.01024000	0.02245000	NM_016529	intron	0	Hs.444957	ATP8A2
rs2137520	13	103468076	0.4315	87.02%	0.517200	0.134700	0.01024000	0.00672200	NM_000452	upstream	950879	Hs.194783	SLC10A2
rs1340184	1	176342013	0.0899	93.68%	0.454300	0.538400	0.01025000	0.00621500	NM_170692	intron	0	Hs.656823	RASAL2
rs7407762	18	36255946	0.2424	92.63%	0.242400	0.569000	0.01028000	0.02273000	ENST00000364090	upstream	50838	---	---
rs878196	5	86830251	0.4749	97.89%	0.904500	1.000000	0.01028000	0.06856000	ENST00000388468	upstream	8519	---	---
rs10501929	11	99278381	0.0756	95.09%	0.378900	0.376000	0.01029000	0.00583900	NM_175566	intron	0	Hs.656783	CNTN5
rs712374	14	36406369	0.1769	97.19%	1.000000	1.000000	0.01029000	0.01171000	NM_030631	intron	0	Hs.693866	SLC25A21
rs7988230	13	19526985	0.0414	93.33%	0.064240	0.095790	0.01029000	0.05672000	NM_003453	intron	0	Hs.644041	ZMYM2
rs1392885	4	101712792	0.1489	91.93%	0.024280	0.065020	0.01031000	0.01021000	NM_016242	upstream	54590	Hs.152913	EMCN
rs7859334	9	20660966	0.3041	94.04%	0.148300	0.132200	0.01032000	0.04096000	NM_017794	intron	0	Hs.408652	KIAA1797
rs10500130	7	138466453	0.0743	96.84%	0.378100	0.374300	0.01034000	0.02707000	NM_024926	upstream	2611	Hs.659165	TTC26
rs1383837	8	79220905	0.3687	97.54%	0.897800	1.000000	0.01034000	0.07960000	NM_000318	upstream	1145998	Hs.437966	PXMP3
rs2737244	8	116727405	0.4784	97.54%	0.092000	0.135900	0.01034000	0.16360000	NM_014112	intron	0	Hs.657018	TRPS1
rs1901696	8	114860437	0.2639	81.75%	0.612300	1.000000	0.01035000	0.06971000	NM_052900	upstream	342019	Hs.91381	CSMD3
rs1032047	3	128324474	0.0491	92.98%	0.475100	0.542800	0.01037000	0.01327000	NM_032242	downstream	85556	Hs.432329	PLXNA1
rs6697656	1	156787361	0.4857	97.89%	0.188200	0.546300	0.01037000	0.08663000	NM_001005189	upstream	2842	Hs.553780	OR6Y1
rs2432	1	114433503	0.4052	94.39%	0.526800	1.000000	0.01038000	0.01294000	NM_205848	3UTR	0	Hs.370963	SYT6
rs9316770	13	54689401	0.3723	97.54%	0.898300	0.735000	0.01038000	0.01302000	ENST00000258651	upstream	775950	---	---
rs10489056	4	26575801	0.0176	89.47%	1.000000	1.000000	0.01039000	0.01782000	NM_020860	intron	0	Hs.135763	STIM2
rs1730462	10	53933391	0.0406	95.09%	1.000000	1.000000	0.01041000	0.04021000	NM_012242	downstream	185968	Hs.40499	DKK1
rs607875	11	93705335	0.3563	94.04%	0.111400	0.156800	0.01041000	0.04830000	NM_016540	downstream	44796	Hs.272385	GPR83
rs798966	13	31702129	0.2769	91.23%	0.536200	0.202600	0.01042000	0.04519000	NM_023037	intron	0	Hs.591225	FRY
rs10504742	8	82950922	0.3550	94.39%	1.000000	0.727100	0.01043000	0.01769000	NM_152837	upstream	33932	Hs.492121	SNX16
rs6491830	13	103610962	0.0224	94.04%	1.000000	1.000000	0.01043000	0.99720000	NM_000452	upstream	1093765	Hs.194783	SLC10A2

rs10489971	2	101894259	0.3201	97.54%	0.492100	0.268800	0.01044000	0.03187000	NM_145687	downstream	16676	Hs.431550	MAP4K4
rs3856123	1	181022917	0.0225	93.68%	0.118700	0.178100	0.01044000	0.99740000	NM_030769	upstream	5100	Hs.496969	NPL
rs10495779	2	30861638	0.1863	95.09%	0.229000	0.260600	0.01046000	0.00135900	NM_144575	intron	0	Hs.660911	CAPN13
rs7534319	1	156798919	0.4387	94.39%	0.387700	0.432800	0.01046000	0.05443000	NM_001005189	upstream	14400	Hs.553780	OR6Y1
rs8072462	17	65314625	0.1863	95.09%	0.840900	1.000000	0.01046000	0.05171000	NM_002758	downstream	263558	Hs.463978	MAP2K6
rs4128340	4	89127160	0.2293	93.33%	0.298900	1.000000	0.01047000	0.05665000	NM_001040060	downstream	3658	Hs.313	SPP1
rs757165	16	2903318	0.4678	81.75%	0.003829	0.016660	0.01047000	0.00318500	NM_020912	intron	0	Hs.655321	FLYWCH1
rs10495657	2	17162350	0.3710	97.89%	0.123700	0.031490	0.01049000	0.06246000	ENST00000388051	downstream	5329	---	---
rs10519134	15	74366145	0.0804	89.47%	1.000000	1.000000	0.01049000	0.06644000	NM_000126	intron	0	Hs.39925	ETFA
rs3857428	5	151509510	0.2880	96.84%	0.463900	1.000000	0.01052000	0.01787000	NM_000171	upstream	224914	Hs.121490	GLRA1
rs2823319	21	15797005	0.0128	95.79%	1.000000	1.000000	0.01054000	0.99840000	ENST00000386941	upstream	111469	---	---
rs10500963	11	23506567	0.1756	97.89%	0.537300	1.000000	0.01055000	0.00323400	ENST00000386849	upstream	321330	---	---
rs10510420	3	12408298	0.0223	94.39%	1.000000	1.000000	0.01055000	0.99770000	NM_005037	intron	0	Hs.162646	PPARG
rs1149350	6	161289796	0.1756	97.89%	0.678700	0.647400	0.01055000	0.01882000	NM_006724	upstream	42953	Hs.390428	MAP3K4
rs30669	5	129024772	0.3424	90.18%	1.000000	0.274500	0.01055000	0.02205000	NM_133638	intron	0	Hs.23751	ADAMTS19
rs1507979	2	17211253	0.2082	94.39%	0.582400	0.789700	0.01056000	0.26270000	NM_003385	upstream	374036	Hs.444212	VSNL1
rs481854	4	112073880	0.1306	94.04%	0.276800	0.222100	0.01056000	0.05677000	NM_000325	upstream	310177	Hs.643588	PITX2
rs727955	1	193278389	0.2341	93.68%	0.039060	0.810100	0.01056000	0.00733100	NM_198503	downstream	1183147	Hs.657046	KCNT2
rs1233255	2	190414337	0.2194	97.54%	0.380700	0.142900	0.01058000	0.09053000	NM_000534	intron	0	Hs.111749	PMS1
rs1447429	3	63028137	0.1975	96.84%	0.021860	0.054280	0.01059000	0.16950000	NM_183393	upstream	192043	Hs.654933	CADPS
rs2205779	20	8023023	0.3257	91.58%	0.777800	0.741400	0.01061000	0.09558000	NM_182734	upstream	37885	Hs.431173	PLCB1
rs10500962	11	23505641	0.1745	97.54%	0.531600	1.000000	0.01064000	0.00360500	ENST00000386849	upstream	322256	---	---
rs10494702	1	192503271	0.0535	85.26%	0.138500	0.181800	0.01065000	0.09926000	ENST00000385495	downstream	220585	---	---
rs10505181	8	113368451	0.2000	80.70%	0.300100	0.492200	0.01065000	0.05737000	NM_052900	intron	0	Hs.91381	CSMD3
rs6573960	14	69926775	0.1487	94.39%	0.226500	0.716700	0.01065000	0.01429000	NM_018373	intron	0	Hs.443661	SYNJ2BP
rs1017833	4	71206730	0.0216	97.54%	1.000000	1.000000	0.01066000	0.11700000	NM_033122	upstream	28557	Hs.120316	C4orf35
rs1480741	4	71221349	0.0216	97.54%	1.000000	1.000000	0.01066000	0.11880000	NM_033122	upstream	13938	Hs.120316	C4orf35
rs2010521	15	77274773	0.2401	79.65%	0.071390	0.198800	0.01068000	0.06594000	NM_153815	upstream	104612	Hs.591111	RASGRF1
rs10501766	11	91297444	0.4358	92.98%	0.708000	0.338200	0.01069000	0.19960000	NM_012124	upstream	1701617	Hs.22857	CHORDC1
rs3212539	5	52392458	0.0759	94.74%	1.000000	1.000000	0.01069000	0.01577000	NM_002203	intron	0	Hs.482077	ITGA2
rs951196	6	162581980	0.3863	97.19%	1.000000	0.618700	0.01070000	0.02428000	NM_013988	intron	0	Hs.132954	PARK2
rs10486383	7	20154491	0.0480	95.09%	1.000000	1.000000	0.01071000	0.06617000	NM_182762	intron	0	Hs.598388	7A5
rs10505202	8	114295875	0.0500	91.23%	1.000000	1.000000	0.01071000	0.02409000	NM_052900	intron	0	Hs.91381	CSMD3
rs2052943	2	222019310	0.1333	84.21%	0.395900	0.562300	0.01071000	0.00670900	NM_004438	intron	0	Hs.371218	EPHA4
rs336081	5	54469150	0.2481	91.93%	0.012260	0.007999	0.01071000	0.03134000	NM_152623	intron	0	Hs.669184	CDC20B
rs1021479	16	84514915	0.2043	90.18%	0.570200	0.788400	0.01075000	0.00617900	NM_002163	downstream	1205	Hs.137427	IRF8
rs3920498	1	22365474	0.2536	96.84%	0.636300	0.661600	0.01075000	0.03710000	NM_030761	upstream	23436	Hs.25766	WNT4
rs4881113	10	3180853	0.2178	92.63%	1.000000	0.803800	0.01075000	0.05297000	NM_014889	intron	0	Hs.528300	PITRM1
rs10510971	3	68764105	0.0400	96.49%	0.060480	0.078070	0.01076000	0.01443000	NM_213609	downstream	74945	Hs.655061	FAM19A1
rs1480744	4	71225621	0.0215	97.89%	1.000000	1.000000	0.01076000	0.12150000	NM_033122	upstream	9666	Hs.120316	C4orf35
rs1977504	9	102872354	0.0353	89.47%	0.267100	1.000000	0.01079000	0.00213600	NM_207299	intron	0	Hs.382683	RP11-35N6
rs3100717	2	138491021	0.3130	94.74%	0.480000	0.574600	0.01080000	0.00659800	NM_001024075	downstream	35303	Hs.42151	HNMT
rs10515818	5	160392731	0.3308	91.23%	0.484600	0.360800	0.01081000	0.01592000	NM_000813	downstream	260718	Hs.303527	GABRB2
rs7995365	13	72713928	0.3450	95.09%	0.422000	0.425500	0.01082000	0.01245000	NM_007249	downstream	444222	Hs.373857	KLF12
rs10513175	9	114786691	0.3364	96.49%	0.591400	0.338500	0.01083000	0.00347400	NM_003408	downstream	57304	Hs.150406	ZFP37
rs2041764	2	40424390	0.2996	90.18%	0.881800	0.495100	0.01083000	0.00572900	NM_021097	intron	0	Hs.468274	SLC8A1
rs10489941	1	191950366	0.0781	94.39%	0.063520	0.071690	0.01086000	0.04549000	NM_024529	downstream	460712	Hs.576497	CDC73
rs10508349	10	8338970	0.2704	94.74%	0.646400	1.000000	0.01086000	0.08566000	NM_002051	downstream	181800	Hs.524134	GATA3
rs10519562	4	141629187	0.1750	91.23%	0.389600	0.358000	0.01086000	0.00414800	NM_001034840	intron	0	---	LOC152586
rs708881	12	118432415	0.3382	95.44%	0.787900	0.855700	0.01088000	0.01805000	NM_178499	intron	0	Hs.98188	CCDC60
rs1871710	4	71225004	0.0217	97.19%	1.000000	1.000000	0.01089000	0.12740000	NM_033122	upstream	10283	Hs.120316	C4orf35
rs2001778	11	5575584	0.4556	94.74%	0.110300	0.029690	0.01089000	0.01050000	NM_001003819	intron	0	Hs.125300	TRIM6-TRI
rs4128218	4	36133208	0.4432	92.63%	0.012530	0.645400	0.01089000	0.02803000	ENST00000357504	downstream	111397	---	---
rs2541422	8	37400936	0.1882	95.09%	0.108400	0.128200	0.01090000	0.06339000	NM_021631	downstream	534950	Hs.651853	FKSG2

rs1959915	14	40613479	0.3927	96.49%	0.899600	0.761200	0.01091000	0.04635000	NM_152447	upstream	533044	Hs.136893	LRFN5
rs1990745	5	103409821	0.3100	95.09%	0.117400	0.022850	0.01091000	0.00341200	NM_031438	upstream	483428	Hs.434289	NUDT12
rs1518081	2	199342013	0.2073	96.49%	1.000000	0.837500	0.01092000	0.00827200	NM_006226	downstream	620720	Hs.153322	PLCL1
rs7729828	5	146036640	0.0659	95.79%	0.324300	0.038780	0.01092000	0.01365000	NM_181678	intron	0	Hs.655213	PPP2R2B
rs1537394	1	168673730	0.3863	97.19%	0.163300	0.281500	0.01093000	0.04720000	NM_152281	upstream	94164	Hs.183702	SCYL1BP1
rs2058404	7	10133347	0.0128	96.14%	1.000000	1.000000	0.01093000	0.02831000	ENST00000387411	upstream	95602	---	---
rs496916	13	109649015	0.2559	89.82%	0.140900	0.137100	0.01094000	0.01079000	NM_001845	intron	0	Hs.17441	COL4A1
rs2899632	15	56599161	0.1398	97.89%	0.452500	1.000000	0.01095000	0.03153000	NM_000236	intron	0	Hs.654472	LIPC
rs7124287	11	96375443	0.4758	94.39%	0.328900	0.442900	0.01095000	0.01064000	ENST00000362445	upstream	658231	---	---
rs9304243	18	36966235	0.1398	97.89%	0.452500	0.285100	0.01095000	0.06681000	NM_002647	upstream	822962	Hs.464971	PIK3C3
rs711865	4	110173221	0.4720	94.04%	0.014020	0.168700	0.01096000	0.00826400	NM_032518	intron	0	Hs.658842	COL25A1
rs7870469	9	101899623	0.1111	97.89%	0.126000	0.334800	0.01098000	0.02309000	NM_015051	intron	0	Hs.591899	TXNDC4
rs1478876	13	80741069	0.4837	86.32%	0.073440	0.195700	0.01099000	0.00389700	ENST00000387380	downstream	3117	---	---
rs10490899	10	24635726	0.0817	90.18%	0.073140	0.081350	0.01102000	0.01422000	NM_019590	intron	0	Hs.445885	KIAA1217
rs2277401	12	5712102	0.3718	68.42%	0.000744	0.000517	0.01104000	0.03446000	NM_020373	intron	0	Hs.148970	TMEM16B
rs7716399	5	158083224	0.0932	82.81%	1.000000	1.000000	0.01104000	0.01877000	NM_024007	intron	0	Hs.657753	EBF1
rs10512687	5	38617019	0.3095	88.42%	0.183600	0.324400	0.01107000	0.00871000	NM_002310	intron	0	Hs.133421	LIFR
rs2442359	13	38202814	0.3871	97.89%	0.613800	0.358900	0.01107000	0.00373000	NM_207361	intron	0	Hs.253994	FREM2
rs1361472	13	58406463	0.2491	93.68%	0.743400	1.000000	0.01110000	0.00653100	ENST00000365514	upstream	406577	---	---
rs10514961	5	63123359	0.0775	95.09%	1.000000	1.000000	0.01111000	0.00837100	NM_000524	downstream	168675	Hs.247940	HTR1A
rs1389319	5	64662209	0.0860	97.89%	0.438700	1.000000	0.01111000	0.04899000	NM_197941	intron	0	Hs.482291	ADAMTS6
rs2131592	5	64671924	0.0866	97.19%	0.441100	1.000000	0.01111000	0.05501000	NM_197941	intron	0	Hs.482291	ADAMTS6
rs718652	13	25878590	0.3933	83.86%	0.014840	0.157900	0.01112000	0.04038000	NM_001260	downstream	2021	Hs.382306	CDK8
rs10502759	18	37083253	0.0799	94.39%	0.679200	0.694400	0.01113000	0.03256000	NM_002647	upstream	705944	Hs.464971	PIK3C3
rs7093376	10	84018388	0.3971	97.19%	0.900700	0.871800	0.01113000	0.06681000	NM_001010848	intron	0	Hs.125119	NRG3
rs1035496	5	41174006	0.2917	96.84%	0.467400	0.692700	0.01115000	0.08059000	NM_000065	downstream	4087	Hs.481992	C6
rs3885379	4	190714909	0.1214	73.68%	0.049710	0.601600	0.01117000	0.01541000	ENST00000378770	downstream	81571	---	---
rs956129	15	65416229	0.2554	97.54%	0.081320	0.128200	0.01117000	0.01569000	NM_022784	intron	0	Hs.657894	IQCH
rs10514151	5	78339243	0.0796	94.74%	0.227800	0.394600	0.01120000	0.05065000	NM_013391	intron	0	Hs.655653	DMGDH
rs10518007	4	67385120	0.1011	95.44%	0.168600	0.737900	0.01120000	0.02547000	ENST00000383902	downstream	592785	---	---
rs249732	5	141867735	0.2741	80.00%	0.000000	0.000000	0.01121000	0.02413000	NM_033137	downstream	85572	Hs.483635	FGF1
rs1370360	2	55416992	0.2609	96.84%	0.754600	0.390800	0.01123000	0.02882000	NM_018084	intron	0	Hs.292925	CCDC88A
rs2061638	5	152618325	0.1231	91.23%	1.000000	1.000000	0.01123000	0.00448900	NM_000827	upstream	232174	Hs.519693	GRIA1
rs4865582	5	54029405	0.2435	95.09%	0.030790	0.261300	0.01123000	0.03959000	NM_052870	downstream	177979	Hs.432755	SNAG1
rs2593122	10	55327026	0.1890	86.32%	1.000000	0.820700	0.01124000	0.03028000	NM_033056	intron	0	Hs.672170	PCDH15
rs7809986	7	78525055	0.1439	97.54%	0.048380	1.000000	0.01124000	0.02551000	NM_012301	intron	0	Hs.654788	MAGI2
rs2211404	6	112852722	0.3887	92.98%	0.039280	0.216300	0.01125000	0.01272000	ENST00000386079	upstream	51899	---	---
rs10496795	2	138521143	0.1519	94.74%	0.353000	0.609500	0.01126000	0.00676300	NM_001024075	downstream	65425	Hs.42151	HNMT
rs7135276	12	40329444	0.3187	91.93%	0.392700	0.336900	0.01126000	0.01816000	NM_013377	downstream	74793	Hs.380044	PDZRN4
rs10495301	1	229101263	0.2070	95.79%	0.362300	0.600700	0.01127000	0.03032000	NM_024525	downstream	7349	Hs.424788	TTC13
rs1496786	7	78128273	0.4741	81.40%	0.147000	0.098350	0.01128000	0.18890000	NM_012301	intron	0	Hs.654788	MAGI2
rs1910647	21	24342702	0.3160	94.39%	0.397500	0.194900	0.01128000	0.08652000	ENST00000355181	upstream	440439	---	---
rs717336	22	48069777	0.3412	97.19%	0.893600	0.525900	0.01128000	0.00398800	NM_207478	upstream	258556	---	FLJ44385
rs913098	6	51750772	0.3155	95.09%	0.121200	0.039620	0.01132000	0.00842600	NM_170724	intron	0	Hs.662050	PKHD1
rs10506980	12	88669315	0.3820	93.68%	0.796000	1.000000	0.01134000	0.02866000	ENST00000388111	downstream	24385	---	---
rs10514724	3	69839673	0.2956	96.14%	0.147500	0.127300	0.01134000	0.08645000	NM_198178	upstream	55979	Hs.166017	MITF
rs4239056	17	5265130	0.3907	97.89%	0.381300	0.365000	0.01135000	0.05494000	NM_032308	intron	0	---	RPAIN
rs9303192	17	5265560	0.3907	97.89%	0.381300	0.365000	0.01135000	0.05494000	NM_032308	intron	0	---	RPAIN
rs1571687	21	31018364	0.0441	91.58%	1.000000	1.000000	0.01137000	0.00964300	NM_181617	downstream	22776	Hs.553699	KRTAP21-2
rs10518645	1	80639912	0.0604	92.98%	0.238900	0.238200	0.01138000	0.17250000	ENST00000388387	upstream	850150	---	---
rs10504859	8	89628714	0.1500	94.74%	0.155300	0.204700	0.01139000	0.02944000	NM_003821	upstream	1210459	Hs.103755	RIPK2
rs6989533	8	89617688	0.1500	94.74%	0.155300	0.204700	0.01139000	0.04201000	NM_003821	upstream	1221485	Hs.103755	RIPK2
rs1401705	2	17421443	0.3424	90.18%	0.001342	0.005022	0.01140000	0.04144000	NM_003385	upstream	163846	Hs.444212	VSNL1
rs768804	12	90518229	0.0916	91.93%	1.000000	0.549600	0.01140000	0.03844000	NM_133507	upstream	417292	Hs.156316	DCN

rs10502712	18	34729747	0.0911	94.39%	1.000000	1.000000	0.01143000	0.01416000	NM_020180	upstream	1329806	Hs.435976	BRUNOL4
rs10493689	1	81567840	0.1208	92.98%	0.557900	0.766900	0.01145000	0.00160800	ENST00000370721	intron	0	---	---
rs10496140	2	67116731	0.1848	96.84%	0.691100	0.662400	0.01148000	0.01988000	NM_019002	upstream	361215	Hs.353022	ETAA1
rs10496490	2	115826981	0.2960	97.19%	0.470600	0.434000	0.01148000	0.07137000	NM_001004360	intron	0	Hs.591555	DPP10
rs9313447	5	168544310	0.1212	92.63%	1.000000	1.000000	0.01148000	0.02852000	NM_003062	intron	0	Hs.604116	SLIT3
rs774788	18	48093325	0.1109	90.18%	0.000203	0.004067	0.01150000	0.01341000	NM_005215	upstream	27831	Hs.579550	DCC
rs10496165	2	68473593	0.2199	93.33%	0.594000	1.000000	0.01151000	0.00886700	NM_002664	intron	0	Hs.468840	PLEK
rs6467491	7	132789301	0.1568	82.81%	0.004911	0.006168	0.01153000	0.00724300	NM_001037126	intron	0	Hs.321273	EXOC4
rs7241727	18	28623586	0.3454	91.93%	0.785700	1.000000	0.01154000	0.10240000	NM_020805	upstream	16614	Hs.446164	KLHL14
rs259174	5	119121597	0.3773	94.39%	0.091320	0.064680	0.01157000	0.04246000	NM_182761	downstream	122182	Hs.406861	LOC340069
rs669644	5	39912612	0.4079	97.19%	0.216100	0.871900	0.01158000	0.01381000	NM_001343	upstream	451909	Hs.481980	DAB2
rs10491157	17	50574958	0.0429	94.04%	1.000000	1.000000	0.01160000	0.01572000	NM_178509	intron	0	Hs.35199	STXBP4
rs10507510	13	41971157	0.2025	97.89%	0.194300	0.676800	0.01160000	0.02267000	NM_033012	upstream	75283	Hs.333791	TNFSF11
rs794255	6	119392448	0.0657	96.14%	0.614900	0.617300	0.01160000	0.06245000	NM_024581	intron	0	Hs.443789	C6orf60
rs9299457	10	52509700	0.2833	92.28%	0.447000	0.011820	0.01161000	0.04930000	NM_006258	intron	0	Hs.654556	PRKG1
rs1930386	9	80756308	0.1014	96.84%	0.500500	0.602500	0.01162000	0.16370000	ENST00000386386	upstream	208060	---	---
rs7114092	11	33602034	0.3398	90.88%	0.580000	0.717700	0.01163000	0.00603000	ENST00000389726	intron	0	---	---
rs132692	22	34909112	0.2581	97.89%	0.087580	0.104700	0.01165000	0.15660000	NM_145660	downstream	7957	Hs.115099	APOL4
rs2172829	4	85331893	0.0357	93.33%	1.000000	1.000000	0.01165000	0.04217000	ENST00000363238	upstream	41941	---	---
rs10503603	8	17607342	0.1122	89.12%	0.006180	0.021750	0.01166000	0.00252500	NM_001001927	intron	0	---	MTUS1
rs10515858	5	162772506	0.0281	93.68%	0.183000	1.000000	0.01166000	0.03352000	NM_199246	upstream	24649	Hs.79101	CCNG1
rs1921314	12	11473914	0.0583	93.33%	0.215000	0.212000	0.01166000	0.07867000	NM_199354	upstream	34161	Hs.631726	PRB1
rs359470	5	173233293	0.4644	93.68%	0.460100	0.124400	0.01166000	0.00877600	NM_030627	upstream	15502	Hs.127126	CPEB4
rs613188	1	238011978	0.2292	97.19%	0.128100	0.334400	0.01169000	0.04448000	NM_000740	intron	0	Hs.7138	CHRM3
rs198278	7	24214718	0.4757	94.04%	0.006894	0.220400	0.01171000	0.05946000	ENST00000387588	downstream	53037	---	---
rs2255565	2	118114972	0.0940	93.33%	0.713000	0.710500	0.01171000	0.01901000	NM_006773	upstream	173753	Hs.363492	DDX18
rs544166	11	131320554	0.3025	96.84%	0.255800	0.180300	0.01171000	0.03537000	NM_016522	intron	0	Hs.504352	HNT
rs6495809	15	34038746	0.1970	94.39%	0.562900	0.830900	0.01171000	0.01363000	NM_080650	upstream	413090	Hs.107196	ATPBD4
rs7827393	8	56669762	0.0431	93.68%	0.390300	1.000000	0.01172000	0.04267000	NM_052898	downstream	70422	Hs.130197	XKR4
rs10483670	14	56079771	0.0766	96.14%	0.200000	0.078130	0.01173000	0.02232000	NM_017799	upstream	36493	Hs.497253	C14orf101
rs10510780	3	56086412	0.1007	97.54%	0.331100	0.602600	0.01173000	0.02037000	NM_015576	intron	0	Hs.476389	ERC2
rs2048541	11	114884183	0.1063	94.04%	0.196600	0.185100	0.01173000	0.04899000	NM_014333	upstream	3861	Hs.370510	CADM1
rs1341198	6	63794816	0.3783	92.28%	0.115000	0.016930	0.01174000	0.00696700	ENST00000370659	upstream	184494	---	---
rs10502763	18	37178557	0.1537	94.74%	0.099430	0.032650	0.01175000	0.02651000	NM_002647	upstream	610640	Hs.464971	PIK3C3
rs627894	11	81585586	0.4669	95.44%	0.038190	0.065080	0.01175000	0.01759000	ENST00000357859	upstream	492663	---	---
rs9322419	6	153669526	0.2074	80.35%	0.007720	0.006350	0.01175000	0.15930000	ENST00000312401	upstream	23965	---	---
rs802296	6	124542788	0.3715	88.77%	0.590900	0.602100	0.01176000	0.00565100	NM_001040214	intron	0	Hs.656604	NKAIN2
rs2030712	7	138945471	0.3258	93.68%	0.265900	0.623600	0.01177000	0.07682000	NM_022740	intron	0	Hs.397465	HIPK2
rs2139090	11	93363251	0.3826	92.63%	1.000000	0.874700	0.01178000	0.00328900	ENST00000389578	upstream	30941	---	---
rs687582	13	43405689	0.2939	97.89%	0.566300	0.695000	0.01180000	0.02946000	NM_153218	downstream	41322	Hs.210586	C13orf31
rs10516838	4	90677255	0.1956	95.09%	0.175100	1.000000	0.01181000	0.01876000	NM_000345	downstream	189224	Hs.271771	SNCA
rs1331649	13	88636728	0.1956	95.09%	0.332900	0.787600	0.01181000	0.01538000	NM_015567	downstream	1506856	Hs.591208	SLITRK5
rs7041758	9	109320317	0.2372	96.14%	0.066410	0.058820	0.01181000	0.02464000	NM_004235	upstream	28569	Hs.376206	KLF4
rs10507396	13	30611542	0.1022	96.14%	1.000000	0.602500	0.01182000	0.04602000	NM_006644	intron	0	Hs.36927	HSPH1
rs1426247	11	59805749	0.0351	95.09%	1.000000	1.000000	0.01183000	0.00341600	NM_148975	intron	0	Hs.325960	MS4A4A
rs10485739	20	10108092	0.4724	95.44%	0.716400	0.758800	0.01184000	0.09360000	NM_130811	upstream	39385	Hs.167317	SNAP25
rs10517003	4	40963950	0.1319	95.79%	0.283600	0.157500	0.01184000	0.03691000	NM_004181	intron	0	Hs.518731	UCHL1
rs2378665	9	85924039	0.3369	97.89%	0.420900	0.477500	0.01184000	0.02079000	NM_024945	downstream	115234	Hs.284137	RMI1
rs587703	11	116332775	0.0082	85.61%	1.000000	1.000000	0.01184000	0.99870000	NM_025164	intron	0	Hs.167451	KIAA0999
rs10510364	3	7356271	0.1625	97.19%	0.505200	0.228200	0.01185000	0.02686000	NM_181875	intron	0	---	GRM7
rs1872325	2	22775527	0.4341	95.79%	0.805500	0.545100	0.01186000	0.02637000	ENST00000388128	upstream	162404	---	---
rs2345475	4	42443300	0.4206	97.19%	0.623100	0.880700	0.01188000	0.05754000	NM_006095	upstream	89641	Hs.435052	ATP8A1
rs2378303	9	80734665	0.4355	89.82%	0.527200	1.000000	0.01188000	0.06180000	ENST00000386386	upstream	186417	---	---
rs1599238	18	53342611	0.2854	94.04%	0.230500	0.305100	0.01191000	0.15140000	NM_000140	downstream	23924	Hs.465221	FECH

rs10509346	10	75266020	0.1673	95.44%	0.077570	0.053980	0.01192000	0.01340000	NM_172172	intron	0	---	CAMK2G
rs182713	12	52560891	0.1449	96.84%	0.002385	0.002782	0.01192000	0.00592600	NM_017410	upstream	57952	Hs.118608	HOXC13
rs984440	8	139146910	0.1272	97.89%	0.277100	0.261500	0.01193000	0.00739400	NM_015912	downstream	66798	Hs.126024	FAM135B
rs718714	18	1686271	0.1012	88.42%	0.151300	0.225600	0.01195000	0.05401000	NM_022840	downstream	841259	Hs.126888	METTL4
rs10518819	2	57568221	0.3496	96.84%	0.692800	0.598100	0.01198000	0.00617400	NM_006296	upstream	559003	Hs.631890	VRK2
rs32249	5	12519975	0.0489	82.46%	0.000907	0.004000	0.01199000	0.04040000	NM_001369	downstream	1225064	Hs.212360	DNAH5
rs9332418	1	182268515	0.2841	95.09%	0.655100	0.494600	0.01199000	0.05491000	NM_015101	intron	0	Hs.387995	GLT25D2
rs1999571	1	98018329	0.0352	94.74%	0.279600	1.000000	0.01201000	0.00840300	NM_000110	intron	0	Hs.335034	DPYD
rs4941700	13	32383381	0.2852	94.74%	0.765700	0.314600	0.01202000	0.01109000	NM_015032	downstream	133224	Hs.699308	PDS5B
rs10496065	2	57567944	0.3489	97.54%	0.692800	0.489400	0.01203000	0.00537800	NM_006296	upstream	559280	Hs.631890	VRK2
rs2271700	10	71519942	0.2986	97.54%	0.886800	0.701300	0.01203000	0.01524000	NM_018649	intron	0	Hs.499953	H2AFY2
rs3733727	5	134717199	0.1236	93.68%	0.393100	0.374800	0.01204000	0.05153000	NM_001040158	intron	0	Hs.696013	H2AFY
rs391449	8	37251704	0.3966	93.33%	0.608600	0.758100	0.01205000	0.12810000	NM_021631	downstream	385718	Hs.651853	FKSG2
rs10498359	14	40607147	0.3856	95.09%	0.521500	0.541800	0.01206000	0.06086000	NM_152447	upstream	539376	Hs.136893	LRFN5
rs7943194	11	84347646	0.4041	93.33%	0.610700	0.066440	0.01207000	0.05460000	ENST00000376104	intron	0	---	---
rs10493370	1	65017928	0.1222	94.74%	0.568600	1.000000	0.01209000	0.01338000	NM_018211	intron	0	Hs.591443	RAVER2
rs10500560	16	70238654	0.2519	93.33%	0.744800	0.376300	0.01210000	0.08608000	NM_015020	3UTR	0	Hs.531564	PHLPPL
rs10508343	10	8190719	0.1895	89.82%	0.228100	0.082220	0.01210000	0.03967000	NM_002051	downstream	33549	Hs.524134	GATA3
rs1917450	11	103062875	0.1502	95.79%	0.029890	0.278900	0.01212000	0.00174900	NM_033135	downstream	220256	Hs.352298	PDGFD
rs1867324	5	112567854	0.4140	97.89%	0.459200	0.113300	0.01214000	0.02762000	NM_002387	intron	0	Hs.593171	MCC
rs747498	8	131099762	0.3266	95.09%	0.581100	1.000000	0.01214000	0.20620000	NM_018482	downstream	33773	Hs.655552	DDEF1
rs10509956	10	113984142	0.0891	96.49%	0.048960	0.155300	0.01215000	0.02342000	NM_058222	upstream	49341	Hs.348615	TECTB
rs1941375	11	85434781	0.2616	97.89%	0.007931	0.004976	0.01215000	0.00609600	NM_001008660	intron	0	Hs.163893	PICALM
rs10483453	14	34965979	0.0996	89.82%	1.000000	1.000000	0.01216000	0.01567000	NM_020529	upstream	22276	Hs.81328	NFKBIA
rs10502437	18	19224704	0.2409	96.14%	0.409900	0.646300	0.01216000	0.01224000	NM_032933	intron	0	Hs.137562	C18orf45
rs10506657	12	71254449	0.2129	89.82%	0.708800	0.037630	0.01217000	0.02056000	NM_013381	intron	0	Hs.199814	TRHDE
rs10490887	3	62693701	0.1637	97.54%	0.272000	0.477000	0.01218000	0.08225000	NM_183393	intron	0	Hs.654933	CADPS
rs1560326	5	97053242	0.0473	85.26%	0.089220	1.000000	0.01218000	0.00193100	ENST00000388601	downstream	13029	---	---
rs9316434	13	48722357	0.2862	94.39%	0.766300	1.000000	0.01218000	0.04791000	NM_030911	intron	0	Hs.388220	CDADC1
rs2227198	1	170626787	0.4158	97.89%	0.006750	0.004006	0.01219000	0.11440000	NM_015569	intron	0	Hs.654775	DNM3
rs10491253	5	127248472	0.0293	89.82%	0.190300	1.000000	0.01224000	0.06738000	ENST00000359805	upstream	160489	---	---
rs10487607	7	24346300	0.2159	95.09%	0.373400	0.841400	0.01225000	0.01024000	NM_000905	downstream	48298	Hs.1832	NPY
rs10498616	14	87625008	0.2241	94.74%	0.117400	0.226200	0.01225000	0.00607100	NM_003608	downstream	76844	Hs.513440	GPR65
rs724806	2	171110979	0.4963	95.79%	0.115200	0.129000	0.01225000	0.05539000	NM_138995	intron	0	Hs.671900	MYO3B
rs2333990	17	76399520	0.1150	96.14%	0.374800	0.752000	0.01226000	0.02711000	NM_020761	intron	0	Hs.133044	KIAA1303
rs4418789	11	12399799	0.4816	95.44%	0.003671	0.049870	0.01226000	0.02811000	NM_018222	intron	0	Hs.607144	PARVA
rs2227199	1	170626403	0.4131	90.88%	0.202100	0.083370	0.01227000	0.16110000	NM_015569	intron	0	Hs.654775	DNM3
rs4589149	1	56088549	0.4398	96.14%	0.221500	0.048760	0.01227000	0.03229000	NM_207465	downstream	118169	---	---
rs1074946	13	58604144	0.2156	94.39%	0.857200	0.544200	0.01228000	0.01799000	ENST00000364727	upstream	348060	---	---
rs748549	12	124387283	0.4211	97.89%	0.177900	1.000000	0.01229000	0.00315400	NM_023928	downstream	193469	Hs.656073	AACS
rs2963050	5	108032333	0.4873	82.81%	0.000000	0.000001	0.01231000	0.03769000	NM_005246	upstream	79089	Hs.221472	FER
rs4880983	10	267232	0.1853	97.54%	0.044170	0.266800	0.01233000	0.02267000	NM_212479	intron	0	Hs.292265	ZMYND11
rs10501128	11	33669411	0.1707	72.98%	0.461200	0.468300	0.01235000	0.02866000	NM_152314	downstream	17191	---	C11orf41
rs2315078	9	133001801	0.2109	96.49%	0.017760	0.195300	0.01236000	0.02819000	NM_005085	intron	0	Hs.654530	NUP214
rs10519357	4	136539097	0.1168	96.14%	0.231900	0.355900	0.01238000	0.09189000	ENST00000385114	upstream	26812	---	---
rs9285581	14	54156583	0.4599	96.14%	0.903200	0.651000	0.01238000	0.04506000	NM_015589	intron	0	Hs.98259	SAMD4A
rs10485030	6	37899874	0.1384	95.09%	0.797900	0.420500	0.01239000	0.00663400	NM_021943	intron	0	Hs.36959	ZFAND3
rs10494687	1	191726077	0.2045	92.63%	0.060630	0.014870	0.01240000	0.08329000	NM_024529	downstream	238398	Hs.576497	CDC73
rs677794	11	81577570	0.3091	96.49%	0.574200	1.000000	0.01240000	0.00853100	ENST00000357859	upstream	500679	---	---
rs10489835	1	156788016	0.4335	97.54%	0.144100	0.275500	0.01241000	0.07200000	NM_001005189	upstream	3497	Hs.553780	OR6Y1
rs1562028	7	135204272	0.4908	95.44%	1.000000	0.762700	0.01241000	0.02164000	NM_145808	downstream	57779	Hs.654894	MTPN
rs1361668	9	101896127	0.2973	92.63%	0.302300	0.606800	0.01242000	0.02634000	NM_015051	intron	0	Hs.591899	TXNDC4
rs2247977	10	56642818	0.0354	94.04%	0.281400	1.000000	0.01242000	0.02232000	NM_033056	upstream	411761	Hs.672170	PCDH15
rs7630783	3	134343284	0.3893	95.09%	0.522700	0.411100	0.01243000	0.05656000	NM_023943	intron	0	Hs.191616	TMEM108

rs9315511	13	37249874	0.3180	95.44%	0.161500	0.039180	0.01243000	0.06747000	NM_016179	intron	0	Hs.262960	TRPC4
rs10493348	1	63918978	0.0323	81.40%	0.208300	1.000000	0.01244000	0.01114000	NM_002633	downstream	20474	Hs.1869	PGM1
rs10512279	9	103106701	0.3491	92.98%	0.685000	1.000000	0.01247000	0.12180000	NM_017753	intron	0	Hs.382683	RP11-35N6
rs1149000	12	70280349	0.3967	95.09%	0.899700	0.649400	0.01247000	0.01515000	NM_144982	downstream	9302	Hs.527874	CCDC131
rs10503450	8	13261245	0.1152	89.82%	0.350200	0.124300	0.01249000	0.03367000	NM_024767	intron	0	Hs.134296	DLC1
rs1368119	4	70438785	0.2717	96.84%	0.447000	0.480000	0.01249000	0.14590000	NM_021139	upstream	42573	Hs.285887	UGT2B4
rs1014772	5	102816580	0.1771	95.09%	0.297800	0.221400	0.01250000	0.10100000	NM_031438	downstream	95875	Hs.434289	NUDT12
rs7196791	16	25664089	0.0434	92.98%	1.000000	1.000000	0.01253000	0.01825000	NM_006040	intron	0	Hs.655275	HS3ST4
rs10501586	11	84433725	0.2566	93.68%	0.151300	0.049780	0.01254000	0.02582000	ENST00000376104	intron	0	---	---
rs10493631	1	80250468	0.3645	95.79%	0.434900	0.490700	0.01256000	0.08139000	ENST00000370739	upstream	1005518	---	---
rs7981837	13	70842044	0.4640	82.81%	0.036050	0.738200	0.01257000	0.10360000	NM_004392	downstream	68055	Hs.129452	DACH1
rs1123391	5	2029478	0.3473	91.93%	0.223000	0.856800	0.01258000	0.06091000	NM_016358	upstream	93598	Hs.196927	IRX4
rs951265	15	59077153	0.4477	97.19%	0.716500	0.756600	0.01266000	0.01266000	NM_134261	intron	0	Hs.695914	RORA
rs4979802	10	79566202	0.4072	92.63%	0.125700	0.536800	0.01267000	0.02187000	NM_001026	downstream	95725	Hs.356794	RPS24
rs724334	3	55933122	0.4057	92.98%	0.798800	1.000000	0.01267000	0.00664100	NM_015576	intron	0	Hs.476389	ERC2
rs7996782	13	38937224	0.4927	96.14%	0.070660	0.066310	0.01268000	0.07819000	NM_005780	intron	0	Hs.507798	LHFP
rs10520144	15	37730935	0.0282	93.33%	1.000000	1.000000	0.01272000	0.01378000	NM_152597	intron	0	Hs.129598	FSIP1
rs10509948	10	113479039	0.1169	97.54%	0.034680	0.150500	0.01275000	0.02138000	NM_020918	downstream	420575	Hs.42586	GPAM
rs1995358	5	134736955	0.1245	94.39%	0.395900	0.382100	0.01275000	0.04991000	NM_001040158	intron	0	Hs.696013	H2AFY
rs9309416	2	68459224	0.4458	97.19%	0.007645	0.006588	0.01276000	0.06037000	NM_002664	intron	0	Hs.468840	PLEK
rs1355296	15	34027338	0.0484	97.89%	1.000000	1.000000	0.01279000	0.13310000	NM_080650	upstream	401682	Hs.107196	ATPBD4
rs6098304	20	52945008	0.2603	93.68%	0.114600	0.381000	0.01279000	0.01354000	ENST00000388080	upstream	35250	---	---
rs1982937	12	129434048	0.1036	88.07%	0.735600	1.000000	0.01280000	0.03228000	NM_004764	downstream	11223	Hs.405659	PIWIL1
rs2190547	17	66441030	0.2384	97.89%	0.741300	0.647000	0.01281000	0.05943000	NM_000891	downstream	753275	Hs.1547	KCNJ2
rs2304510	3	14495564	0.2019	94.74%	0.850500	0.420600	0.01283000	0.01511000	NM_003043	intron	0	Hs.529488	SLC6A6
rs2891097	2	1892286	0.1256	76.84%	0.030160	0.362400	0.01287000	0.02319000	NM_015025	intron	0	Hs.434418	MYT1L
rs2641998	9	584262	0.3152	96.84%	0.486300	0.513800	0.01288000	0.01027000	NM_015158	intron	0	Hs.306764	ANKRD15
rs6989402	8	89460062	0.1381	94.04%	0.066140	0.092910	0.01288000	0.01087000	NM_022564	upstream	51170	Hs.546267	MMP16
rs1347637	3	21283719	0.4462	97.89%	0.627800	0.765700	0.01289000	0.00087220	ENST00000295509	upstream	138805	---	---
rs10496769	2	137797535	0.0506	93.68%	0.499400	1.000000	0.01291000	0.00909300	ENST00000272643	intron	0	---	---
rs10500988	11	24706400	0.3615	97.54%	0.242200	0.439400	0.01292000	0.06527000	NM_001009909	intron	0	Hs.144138	LUZP2
rs291296	9	10103900	0.4130	88.77%	0.153200	0.876500	0.01293000	0.00048190	ENST00000363183	downstream	671548	---	---
rs436563	9	10090851	0.4534	97.89%	0.091030	0.122400	0.01300000	0.00060520	ENST00000363183	downstream	658499	---	---
rs210646	6	117948723	0.3224	90.88%	0.776400	1.000000	0.01301000	0.00752900	NM_173674	intron	0	Hs.658304	DCBLD1
rs10517774	4	164011156	0.3561	92.63%	0.140700	0.007897	0.01302000	0.00397300	NM_138386	downstream	258119	Hs.655698	LOC92345
rs2056933	20	8023613	0.3140	90.53%	0.665100	0.497700	0.01302000	0.05983000	NM_182734	upstream	37295	Hs.431173	PLCB1
rs1920267	3	190853682	0.0266	85.61%	1.000000	1.000000	0.01304000	0.02732000	NM_003722	intron	0	Hs.137569	TP63
rs3762982	5	76359244	0.2426	82.46%	0.154400	0.420700	0.01306000	0.03911000	NM_018046	upstream	2743	Hs.634849	AGGF1
rs10821851	10	62330917	0.2963	94.74%	0.558900	0.866700	0.01307000	0.02108000	NM_001032380	intron	0	Hs.148670	RHOBTB1
rs334960	3	18585881	0.2457	82.11%	0.376600	0.237200	0.01308000	0.00686900	NM_002971	upstream	130673	Hs.517717	SATB1
rs10501446	11	78920460	0.0739	92.63%	0.639800	0.649800	0.01309000	0.06195000	ENST00000385066	upstream	129643	---	---
rs1186892	2	61813727	0.2762	97.19%	0.232800	0.600100	0.01313000	0.12230000	NM_032180	downstream	91767	Hs.440466	FLJ13305
rs1476322	3	162928749	0.4213	82.46%	0.001255	0.000003	0.01313000	0.02085000	ENST00000327928	downstream	224325	---	---
rs9301388	13	108934565	0.4801	97.19%	0.547900	0.880600	0.01314000	0.01859000	NM_003749	downstream	269620	Hs.442344	IRS2
rs2339575	10	66307284	0.0582	96.49%	1.000000	1.000000	0.01315000	0.00862500	NM_013266	downstream	1042441	Hs.660362	CTNNA3
rs10497423	2	174555939	0.3448	87.02%	0.000224	0.001441	0.01316000	0.07414000	NM_001017371	upstream	17263	Hs.531587	SP3
rs10494831	1	200165397	0.3599	90.18%	0.222700	0.472700	0.01318000	0.11040000	NM_012134	intron	0	Hs.519075	LMOD1
rs4935578	10	56567867	0.1931	97.19%	0.702700	0.830100	0.01327000	0.00903300	NM_033056	upstream	336810	Hs.672170	PCDH15
rs532188	6	100251871	0.1794	91.93%	0.528100	0.204700	0.01331000	0.07740000	NM_032503	downstream	222636	Hs.591342	MCHR2
rs6107905	20	6927153	0.2030	95.09%	0.709800	0.605200	0.01333000	0.01854000	NM_001200	downstream	218226	Hs.73853	BMP2
rs1370105	10	128272934	0.1326	97.89%	1.000000	0.576700	0.01335000	0.02892000	NM_001004298	upstream	72934	Hs.587663	C10orf90
rs725550	6	18444756	0.1326	97.89%	1.000000	1.000000	0.01335000	0.01217000	NM_182757	upstream	50817	Hs.148741	RNF144B
rs7151164	14	88045426	0.2110	92.28%	0.264300	0.298900	0.01336000	0.05406000	NM_007039	intron	0	Hs.437040	PTPN21
rs10520301	4	176797085	0.4910	97.54%	0.632600	0.451300	0.01337000	0.05086000	NM_201592	intron	0	Hs.75819	GPM6A

rs7673719	4	172319664	0.2437	97.19%	0.142800	0.066130	0.01339000	0.06832000	NM_001034845	upstream	652643	Hs.386236	GALNT17
rs10519383	4	137334697	0.1492	90.53%	0.013690	0.017540	0.01341000	0.01766000	ENST00000311003	downstream	161021	---	---
rs10516162	4	57631677	0.1516	97.19%	0.159600	0.209000	0.01343000	0.00729500	NM_001553	intron	0	Hs.479808	IGFBP7
rs10505484	8	128170494	0.2556	94.74%	0.266800	0.268000	0.01344000	0.01378000	ENST00000385818	downstream	417413	---	---
rs10511305	3	113296986	0.0681	97.89%	0.028390	0.328600	0.01344000	0.27180000	NM_024616	intron	0	Hs.434247	C3orf52
rs10509309	10	70497287	0.2727	84.91%	0.196300	0.077150	0.01346000	0.03559000	NM_002727	upstream	20547	Hs.1908	SRGN
rs511217	11	29986524	0.1833	94.74%	0.418300	0.658400	0.01347000	0.02531000	NM_002233	downstream	1817	Hs.592002	KCNA4
rs10501247	11	41032784	0.0606	92.63%	1.000000	1.000000	0.01349000	0.02785000	ENST00000386948	downstream	68261	---	---
rs1463073	11	41574517	0.1796	94.74%	0.095320	0.066130	0.01351000	0.03918000	NM_006595	upstream	1715592	Hs.435771	API5
rs2639277	3	81175378	0.3090	93.68%	0.151100	0.002095	0.01351000	0.00506000	NM_000158	downstream	446164	Hs.436062	GBE1
rs2683408	4	63554481	0.1224	85.96%	0.000000	0.000075	0.01351000	0.10130000	NM_001010874	downstream	1273904	Hs.227752	SRD5A2L2
rs6713940	2	50037876	0.3489	94.04%	0.893800	0.860300	0.01351000	0.01705000	NM_138735	intron	0	Hs.637685	NRXN1
rs836019	4	172912358	0.0914	97.89%	0.063520	0.255200	0.01352000	0.00625500	NM_001034845	upstream	59949	Hs.386236	GALNT17
rs1073893	2	108855797	0.1709	97.54%	0.673700	0.755100	0.01353000	0.09846000	NM_144978	intron	0	Hs.362702	CCDC138
rs7356548	5	129751451	0.4272	94.04%	0.803200	0.874000	0.01354000	0.03856000	NM_005340	downstream	771432	Hs.483305	HINT1
rs10493722	1	82944582	0.1516	89.12%	0.324900	0.277100	0.01355000	0.03019000	NM_012302	downstream	713887	Hs.24212	LPHN2
rs7320767	13	88932399	0.3755	97.19%	0.369400	0.095080	0.01355000	0.01617000	ENST00000298437	upstream	120609	---	---
rs10502906	18	45928041	0.0896	97.89%	0.470300	0.462800	0.01356000	0.13650000	ENST00000285039	intron	0	---	---
rs1033147	13	103460337	0.2981	94.74%	0.662700	1.000000	0.01358000	0.00474700	NM_000452	upstream	943140	Hs.194783	SLC10A2
rs10504421	8	69270284	0.0598	96.84%	0.608700	0.604500	0.01358000	0.03961000	NM_024870	intron	0	Hs.591867	DEPDC2
rs10512260	9	100544496	0.0074	95.09%	1.000000	1.000000	0.01358000	0.99870000	NM_173551	intron	0	Hs.406890	ANKS6
rs10516992	4	97722954	0.0074	95.09%	1.000000	1.000000	0.01358000	0.99860000	ENST00000364967	downstream	116548	---	---
rs1240584	6	16841472	0.0074	95.09%	1.000000	1.000000	0.01358000	0.99840000	NM_000332	intron	0	Hs.434961	ATXN1
rs176713	6	90727835	0.1183	97.89%	0.561300	0.646300	0.01358000	0.06011000	NM_021813	intron	0	Hs.269764	BACH2
rs929710	12	4392161	0.0074	95.09%	0.011080	1.000000	0.01358000	0.99840000	NM_020996	downstream	21408	Hs.166015	FGF6
rs10507549	13	46512093	0.1008	90.53%	0.304000	0.726400	0.01359000	0.02245000	ENST00000386918	upstream	82655	---	---
rs1222410	7	133489096	0.0075	94.04%	1.000000	1.000000	0.01359000	0.99840000	NM_144648	intron	0	Hs.149774	LRGUK
rs748138	5	84977466	0.3235	83.51%	0.374900	0.076850	0.01359000	0.00798400	ENST00000333189	downstream	95942	---	---
rs9299191	9	112904366	0.3614	93.68%	0.691300	0.732000	0.01360000	0.14180000	NM_205859	downstream	225218	Hs.381312	OR2K2
rs2570024	3	483467	0.4513	93.68%	0.216900	0.448000	0.01363000	0.00421000	NM_006614	downstream	57369	Hs.148909	CHL1
rs10507629	13	58612935	0.0683	97.54%	1.000000	1.000000	0.01366000	0.05896000	ENST00000364727	upstream	339269	---	---
rs10511749	9	24209684	0.0281	93.68%	0.183000	0.243300	0.01367000	0.06142000	NM_004432	upstream	393349	Hs.166109	ELAVL2
rs1359380	10	91633288	0.4850	93.68%	0.902700	0.878700	0.01367000	0.03539000	NM_016195	downstream	108608	Hs.240	MPHOSPH1
rs2703813	17	20055907	0.2860	82.81%	0.751400	0.274300	0.01369000	0.01755000	NM_001033553	intron	0	Hs.431045	SPECC1
rs719614	13	48743486	0.2808	96.84%	0.765400	0.838900	0.01369000	0.04986000	NM_030911	intron	0	Hs.388220	CDADC1
rs7715468	5	162484076	0.4139	93.68%	0.129600	0.192900	0.01370000	0.10760000	NM_199246	upstream	313079	Hs.79101	CCNG1
rs9326835	5	110802124	0.2855	96.49%	0.657700	0.236100	0.01370000	0.03521000	NM_001744	intron	0	Hs.591269	CAMK4
rs10493029	1	26425552	0.0730	93.68%	1.000000	1.000000	0.01374000	0.00538800	NM_022778	upstream	7728	Hs.63795	CCDC21
rs471095	9	22730332	0.3123	91.58%	0.773400	0.497700	0.01374000	0.01723000	NM_022160	downstream	287860	Hs.371976	DMRTA1
rs10498403	14	47966988	0.1093	94.74%	0.025540	0.354900	0.01375000	0.09387000	ENST00000384636	upstream	86703	---	---
rs10490433	2	16934254	0.2325	95.09%	0.398800	0.641100	0.01376000	0.04157000	ENST00000386331	downstream	34498	---	---
rs10520627	15	84779592	0.0074	94.74%	1.000000	1.000000	0.01378000	0.99860000	ENST00000389298	intron	0	---	---
rs4947673	7	52022186	0.3325	67.02%	0.033130	0.092700	0.01378000	0.02782000	ENST00000388001	downstream	270066	---	---
rs9307757	4	138096276	0.0074	94.74%	1.000000	1.000000	0.01378000	0.99860000	ENST00000364398	downstream	537503	---	---
rs10520694	15	89701629	0.1889	91.93%	0.839800	0.822900	0.01381000	0.04003000	NM_013272	upstream	496321	Hs.311187	SLCO3A1
rs16928254	9	11942979	0.0076	92.63%	1.000000	1.000000	0.01381000	0.99850000	ENST00000364783	downstream	347291	---	---
rs2063345	6	160780144	0.2689	83.51%	0.000000	0.000013	0.01381000	0.01575000	NM_021977	intron	0	Hs.567337	SLC22A3
rs10508091	13	102040254	0.1119	94.04%	1.000000	0.746100	0.01384000	0.05338000	NM_003291	upstream	7033	Hs.432424	TPP2
rs1361251	9	102682223	0.3873	96.49%	0.702900	0.877600	0.01384000	0.01267000	NM_001018116	downstream	292231	Hs.99004	LOC347273
rs2742347	2	179308893	0.1461	93.68%	0.808200	0.709800	0.01384000	0.08988000	NM_133437	intron	0	Hs.654592	TTN
rs4918563	10	112448484	0.1692	92.28%	0.075200	0.025590	0.01385000	0.00810600	ENST00000369521	intron	0	---	---
rs964248	5	94389593	0.0265	85.96%	1.000000	1.000000	0.01385000	0.00139000	NM_001002796	intron	0	Hs.655087	MCTP1
rs1561288	2	25222506	0.3466	88.07%	0.209000	0.580500	0.01387000	0.02505000	ENST00000264719	intron	0	---	---
rs7829812	8	89791390	0.2176	97.54%	0.727000	1.000000	0.01387000	0.05318000	NM_003821	upstream	1047783	Hs.103755	RIPK2

rs10510927	3	64725784	0.4059	89.47%	0.004348	0.211000	0.01388000	0.15300000	NM_182920	upstream	77379	Hs.656071	ADAMTS9
rs1012566	2	11900734	0.3877	96.84%	0.255100	0.441900	0.01389000	0.18420000	NM_145693	downstream	15752	Hs.467740	LPIN1
rs1486988	4	135508554	0.2948	88.07%	1.000000	0.541300	0.01389000	0.02008000	ENST00000386270	downstream	578648	---	---
rs2890982	9	14171653	0.1935	97.89%	0.702400	0.175800	0.01389000	0.00625400	NM_005596	intron	0	Hs.644095	NFIB
rs2572116	6	99589485	0.0970	94.04%	0.723400	0.717600	0.01390000	0.00564800	NM_012160	upstream	86915	Hs.536850	FBXL4
rs9291500	4	27145506	0.3273	96.49%	0.338700	0.463100	0.01391000	0.02143000	ENST00000363691	upstream	311359	---	---
rs984441	8	139146659	0.2212	97.54%	1.000000	0.693200	0.01391000	0.01007000	NM_015912	downstream	67049	Hs.126024	FAM135B
rs10497454	2	176971096	0.1107	95.09%	0.754400	0.745400	0.01392000	0.01479000	NM_001006635	downstream	60099	Hs.470728	MTX2
rs360388	2	126837503	0.0830	95.09%	0.702700	1.000000	0.01392000	0.08301000	NM_016815	upstream	327041	Hs.59138	GYPC
rs720651	13	25129946	0.3763	97.89%	0.525200	0.617400	0.01392000	0.01498000	NM_016529	intron	0	Hs.444957	ATP8A2
rs1841579	6	58594201	0.2678	93.68%	0.353200	0.832000	0.01393000	0.00769600	NM_206908	upstream	198546	---	GUSBL2
rs10491441	5	58924994	0.1605	95.09%	0.651500	0.514400	0.01394000	0.09130000	ENST00000340635	intron	0	---	---
rs2290445	2	39068325	0.0642	90.18%	1.000000	1.000000	0.01394000	0.00272300	NM_005633	intron	0	Hs.654397	SOS1
rs10515778	5	158590590	0.1015	91.58%	0.735800	0.732100	0.01400000	0.02756000	NM_145049	upstream	32267	Hs.591733	UBLCP1
rs1462395	14	46195273	0.0240	95.09%	1.000000	1.000000	0.01401000	0.04569000	NM_080746	upstream	4584	Hs.308332	RPL10L
rs2305516	2	39052470	0.0849	95.09%	0.704200	0.697400	0.01401000	0.00224500	NM_005633	downstream	13999	Hs.654397	SOS1
rs3922595	12	24250039	0.0240	95.09%	0.137000	0.016880	0.01401000	0.09329000	NM_144667	downstream	377781	Hs.350668	FLJ32894
rs10500961	11	23505379	0.1721	96.84%	0.403300	1.000000	0.01405000	0.00493000	ENST00000386849	upstream	322518	---	---
rs723597	8	51907361	0.3781	97.89%	0.799300	0.644400	0.01405000	0.04165000	NM_018967	downstream	39383	Hs.584914	SNTG1
rs10488670	13	29772175	0.0412	97.89%	1.000000	1.000000	0.01406000	0.02537000	NM_001014380	intron	0	Hs.243596	KATNAL1
rs10518080	4	72103624	0.0412	97.89%	0.376600	1.000000	0.01406000	0.23950000	NM_000788	intron	0	Hs.709	DCK
rs1446821	2	176342447	0.0412	97.89%	0.376600	0.097890	0.01406000	0.00825700	NM_030650	downstream	154449	Hs.209561	KIAA1715
rs9317195	13	61121561	0.0412	97.89%	0.376600	0.097890	0.01406000	0.00535200	NM_022843	upstream	234164	Hs.391781	PCDH20
rs3750300	1	17458721	0.1016	89.82%	0.007717	0.143900	0.01408000	0.00275600	NM_016233	CDS	0	Hs.149195	PADI3
rs721231	1	156017335	0.1985	95.44%	0.850700	0.789800	0.01409000	0.05806000	NM_030764	upstream	3799	Hs.437393	FCRL2
rs9321615	6	137880908	0.1387	96.14%	0.443600	1.000000	0.01409000	0.00876300	NM_175747	upstream	23828	Hs.195398	OLIG3
rs936868	4	72110999	0.0417	96.84%	0.379900	1.000000	0.01410000	0.24700000	NM_000788	intron	0	Hs.709	DCK
rs2071114	11	2937407	0.1849	92.98%	0.105700	0.245900	0.01412000	0.09314000	NM_005969	intron	0	Hs.501684	NAP1L4
rs2399669	10	11303519	0.1264	92.98%	1.000000	1.000000	0.01412000	0.06372000	NM_001025077	intron	0	Hs.309288	CUGBP2
rs10488086	7	30083942	0.2204	97.89%	0.294600	0.046740	0.01413000	0.01487000	NM_015899	intron	0	Hs.558495	PLEKHA9
rs2251775	2	183696844	0.0637	93.68%	0.014520	0.018860	0.01417000	0.06279000	NM_001008544	upstream	484	---	NUP35
rs1004704	16	47094922	0.3327	91.23%	0.263300	0.463100	0.01419000	0.03184000	NM_153029	downstream	35218	Hs.511839	N4BP1
rs1354038	10	57503697	0.4777	94.39%	0.807200	0.450000	0.01421000	0.01458000	NM_001005414	downstream	283508	---	ZWINT
rs1564269	5	40338324	0.4646	94.04%	0.623500	0.536000	0.01421000	0.02015000	ENST00000362491	downstream	32746	---	---
rs798285	7	77777932	0.4777	94.39%	0.087220	0.049170	0.01421000	0.00268300	NM_012301	intron	0	Hs.654788	MAGI2
rs495236	18	64998152	0.4631	95.09%	0.713800	0.645100	0.01423000	0.00722900	NM_152721	upstream	221119	Hs.569915	DOK6
rs2028165	5	106100920	0.1141	84.56%	0.000000	0.000000	0.01425000	0.08062000	NM_001962	downstream	639569	Hs.658451	EFNA5
rs3024490	1	205011934	0.3412	89.47%	0.890100	0.249000	0.01425000	0.00475100	NM_000572	intron	0	Hs.193717	IL10
rs3734369	6	71625850	0.0704	94.74%	1.000000	1.000000	0.01426000	0.03840000	NM_021940	intron	0	Hs.485717	SMAP1
rs6495808	15	34038553	0.2135	93.68%	0.199700	0.543700	0.01426000	0.02344000	NM_080650	upstream	412897	Hs.107196	ATPBD4
rs1941891	18	24850496	0.2306	95.09%	0.391900	0.442000	0.01427000	0.02570000	NM_001792	upstream	839307	Hs.464829	CDH2
rs2181246	6	76600038	0.5000	94.39%	0.179500	0.288000	0.01428000	0.07261000	NM_004999	intron	0	Hs.149387	MYO6
rs1048821	2	138151484	0.2832	97.89%	0.556400	0.087600	0.01431000	0.02038000	ENST00000272643	3UTR	0	---	---
rs10508344	10	8262218	0.1125	95.09%	0.758200	0.624300	0.01431000	0.03113000	NM_002051	downstream	105048	Hs.524134	GATA3
rs4749562	10	30858830	0.1891	93.68%	0.842300	0.825900	0.01432000	0.02926000	NM_005204	downstream	68063	Hs.432453	MAP3K8
rs4767212	12	113012888	0.2518	96.84%	1.000000	0.824900	0.01432000	0.03733000	NM_016196	upstream	124394	Hs.7482	RBM19
rs10485352	6	106854904	0.2753	93.68%	0.045290	0.101100	0.01433000	0.04044000	NM_004849	intron	0	Hs.486063	ATG5
rs2124432	2	128900636	0.3170	92.98%	0.672600	1.000000	0.01435000	0.00291400	ENST00000362822	downstream	18489	---	---
rs1418259	20	167011	0.5000	94.74%	0.544200	0.652000	0.01437000	0.00263000	NM_080831	downstream	8484	Hs.112087	DEFB129
rs947808	11	62984635	0.0293	95.79%	0.017000	0.030360	0.01437000	0.05180000	NM_054108	downstream	2751	Hs.410316	HRASLS5
rs2408412	5	55900767	0.0290	96.84%	1.000000	1.000000	0.01438000	0.05545000	ENST00000381279	intron	0	---	---
rs2059743	3	16604325	0.2708	92.63%	0.876200	0.522500	0.01439000	0.03477000	NM_001351	3UTR	0	Hs.131179	DAZL
rs10485732	20	9467529	0.0288	97.54%	1.000000	1.000000	0.01441000	0.06175000	NM_177990	3UTR	0	Hs.32539	PAK7
rs10504709	8	80781919	0.2795	92.28%	0.219600	0.414500	0.01442000	0.11170000	NM_007029	downstream	40988	Hs.521651	STMN2

rs461721	5	100835207	0.4259	94.74%	0.804000	0.760000	0.01442000	0.00288600	ENST00000388691	upstream	110226	---	---
rs4678221	3	138252492	0.3090	93.68%	0.668200	0.403600	0.01442000	0.00305800	NM_144717	downstream	39882	Hs.61232	IL20RB
rs2843780	8	37401206	0.1870	94.74%	0.071990	0.121000	0.01444000	0.07268000	NM_021631	downstream	535220	Hs.651853	FKSG2
rs10516343	4	19583960	0.2554	96.84%	0.343800	0.278800	0.01445000	0.02028000	NM_004787	upstream	280373	Hs.699467	SLIT2
rs268086	5	39515041	0.2964	88.77%	1.000000	0.541800	0.01447000	0.06098000	NM_001343	upstream	54338	Hs.481980	DAB2
rs1438524	1	189066548	0.4648	94.74%	0.066430	0.090280	0.01448000	0.03097000	NM_199051	upstream	353166	Hs.65765	FAM5C
rs2385861	3	46904357	0.0271	97.19%	1.000000	1.000000	0.01448000	0.08795000	NM_000316	intron	0	Hs.1019	PTHR1
rs347163	3	32435579	0.4594	95.09%	0.465200	0.758300	0.01449000	0.07714000	NM_181472	intron	0	Hs.440494	CMTM7
rs2466605	11	123605456	0.4079	97.19%	0.901000	0.880500	0.01454000	0.02719000	NM_001007249	downstream	3881	Hs.381319	OR8G2
rs10506322	12	52541474	0.1852	94.74%	0.839600	0.823100	0.01458000	0.00834600	NM_017410	upstream	77369	Hs.118608	HOXC13
rs4503638	12	69534032	0.4562	88.07%	1.000000	0.349800	0.01458000	0.02046000	NM_002849	intron	0	Hs.506076	PTPRR
rs2384351	12	113008899	0.2527	97.89%	0.874900	0.826900	0.01459000	0.03261000	NM_016196	upstream	120405	Hs.7482	RBM19
rs2761272	20	10697074	0.2938	96.14%	0.770700	0.692000	0.01460000	0.11240000	NM_000214	upstream	94438	Hs.224012	JAG1
rs10497357	2	170578841	0.3764	92.28%	0.357600	0.874400	0.01462000	0.05688000	NM_172070	intron	0	---	UBR3
rs10506658	12	71254956	0.1941	83.16%	0.836500	0.105500	0.01465000	0.01908000	NM_013381	intron	0	Hs.199814	TRHDE
rs7711884	5	146075685	0.1913	92.63%	0.108300	0.046630	0.01465000	0.00916200	NM_181678	intron	0	Hs.655213	PPP2R2B
rs10509463	10	84689837	0.3396	84.21%	0.886600	1.000000	0.01469000	0.02611000	NM_001010848	intron	0	Hs.125119	NRG3
rs990909	13	38188569	0.4760	95.09%	1.000000	0.765600	0.01469000	0.00525300	NM_207361	intron	0	Hs.253994	FREM2
rs1450906	4	42600857	0.2896	97.54%	0.108200	0.172700	0.01470000	0.07439000	NM_006095	upstream	246990	Hs.435052	ATP8A1
rs2001208	10	84936406	0.3500	94.74%	0.348600	0.377700	0.01471000	0.11210000	ENST00000363904	upstream	118599	---	---
rs2512185	11	123634716	0.4073	96.49%	1.000000	1.000000	0.01472000	0.03211000	NM_001005198	intron	0	Hs.626840	OR8G5
rs10506918	12	84752044	0.2266	97.54%	0.497400	0.845800	0.01474000	0.07856000	NM_005447	upstream	28171	Hs.527881	PAMCI
rs708087	1	18551345	0.2159	92.63%	0.006359	0.155500	0.01475000	0.15100000	NM_032880	intron	0	Hs.212511	IGSF21
rs9311951	3	65663422	0.2176	91.93%	0.003144	0.000338	0.01477000	0.03805000	NM_001033057	intron	0	Hs.651939	MAGI1
rs1676853	18	64600350	0.0358	92.98%	0.036450	0.061030	0.01486000	0.08727000	NM_024781	upstream	16000	Hs.280781	CCDC102B
rs2115448	5	85010848	0.3102	93.33%	0.199900	0.082550	0.01486000	0.02983000	ENST00000333189	downstream	129324	---	---
rs4816083	20	8702768	0.0296	94.74%	1.000000	1.000000	0.01489000	0.01946000	NM_182734	intron	0	Hs.431173	PLCB1
rs728997	6	51750794	0.3221	93.68%	0.069360	0.025600	0.01489000	0.01056000	NM_170724	intron	0	Hs.662050	PKHD1
rs389075	5	54445344	0.1565	97.54%	0.168800	0.140700	0.01490000	0.01402000	NM_152623	3UTR	0	Hs.669184	CDC20B
rs1876092	3	63565854	0.3155	95.09%	0.049460	0.100800	0.01491000	0.05755000	NM_144642	intron	0	Hs.648668	SYNPR
rs9303982	18	6081294	0.0248	70.88%	1.000000	1.000000	0.01491000	0.07194000	NM_173464	intron	0	Hs.128279	L3MBTL4
rs9697	1	238719570	0.1901	92.28%	0.072980	0.567800	0.01493000	0.02569000	NM_022469	3UTR	0	Hs.98206	GREM2
rs4782644	16	81046774	0.1580	81.05%	0.618500	0.469800	0.01494000	0.04396000	NM_001257	upstream	402686	Hs.654386	CDH13
rs4846826	1	215484741	0.3027	89.82%	0.882600	0.303400	0.01495000	0.02600000	NM_206595	upstream	155171	Hs.444225	ESRRG
rs10503161	8	1057421	0.4283	90.53%	0.373600	0.537900	0.01496000	0.01896000	NM_004745	upstream	379520	Hs.113287	DLGAP2
rs10490615	2	65792593	0.0821	96.14%	0.405800	0.113500	0.01497000	0.06701000	NM_002398	upstream	723443	Hs.526754	MEIS1
rs10501712	11	89587770	0.1296	94.74%	0.589900	0.259700	0.01497000	0.02337000	NM_012124	intron	0	Hs.22857	CHORDC1
rs2833052	21	31026162	0.0417	96.84%	1.000000	1.000000	0.01500000	0.02237000	NM_181617	downstream	14978	Hs.553699	KRTAP21-2
rs9283770	5	22613182	0.3257	91.58%	0.089940	0.022760	0.01500000	0.06548000	NM_004061	intron	0	Hs.113684	CDH12
rs644425	11	109192230	0.0295	95.09%	1.000000	1.000000	0.01501000	0.07823000	NM_207645	downstream	390612	Hs.172982	LOC399947
rs6501347	17	65322249	0.1820	95.44%	0.415700	0.548000	0.01501000	0.14960000	NM_002758	downstream	271182	Hs.463978	MAP2K6
rs777230	3	127786728	0.2510	91.58%	0.000452	0.001060	0.01501000	0.00245600	NM_001039783	upstream	12652	Hs.477475	TR2IT1
rs1071895	10	120637136	0.3128	74.04%	0.000375	0.000368	0.01503000	0.06751000	NM_001009553	upstream	142082	---	NANOS1
rs1239067	2	78719000	0.4286	90.88%	0.309700	0.760000	0.01503000	0.01790000	ENST00000325094	upstream	225459	---	---
rs899036	11	41639486	0.1793	96.84%	0.063050	0.010700	0.01503000	0.00025180	NM_006595	upstream	1650623	Hs.435771	API5
rs1444581	12	105838381	0.2768	95.09%	0.651400	0.415200	0.01504000	0.01014000	NM_018157	downstream	33191	Hs.131306	RIC8B
rs7903492	10	118340939	0.0443	95.09%	0.086140	0.121800	0.01506000	0.02506000	NM_006229	intron	0	Hs.73923	PNLIPRP1
rs9307358	4	112143490	0.0443	95.09%	0.412900	1.000000	0.01506000	0.02017000	NM_000325	upstream	379787	Hs.643588	PITX2
rs6097934	20	52411601	0.1468	94.39%	0.139500	0.276800	0.01508000	0.05172000	NM_018431	upstream	113987	Hs.656582	DOK5
rs1515733	3	162919687	0.4273	96.49%	0.174800	0.001503	0.01509000	0.01252000	ENST00000327928	downstream	215263	---	---
rs353721	8	57855203	0.3104	94.39%	0.569400	0.502000	0.01511000	0.03055000	NM_006211	upstream	334060	Hs.339831	PENK
rs473698	2	70529089	0.3365	91.23%	0.212200	1.000000	0.01511000	0.02130000	NM_003236	3UTR	0	Hs.170009	TGFA
rs1627431	12	74249179	0.3208	92.98%	0.120300	0.706200	0.01512000	0.02809000	NM_007043	upstream	57520	Hs.645517	KRR1
rs9291499	4	27145486	0.3345	97.54%	0.180700	0.281200	0.01512000	0.03391000	ENST00000363691	upstream	311339	---	---

rs10516483	4	103010928	0.4253	91.58%	0.099240	0.519700	0.01515000	0.01835000	NM_017935	intron	0	Hs.480400	BANK1
rs10516748	4	86585836	0.1498	93.68%	0.335500	0.796000	0.01515000	0.02096000	NM_001042669	upstream	484614	Hs.444229	ARHGAP24
rs2404571	3	162875663	0.1141	92.28%	1.000000	1.000000	0.01517000	0.03048000	ENST00000327928	downstream	171239	---	---
rs10512312	9	105018860	0.4288	96.14%	0.267500	0.449200	0.01518000	0.01117000	NM_001340	downstream	198269	Hs.3232	CYLC2
rs1493931	8	119825834	0.1815	87.02%	0.672100	0.476200	0.01518000	0.01474000	NM_207506	upstream	122533	Hs.492653	SAMD12
rs1481253	4	67441849	0.1099	95.79%	0.007960	0.018750	0.01519000	0.01192000	ENST00000383902	downstream	536056	---	---
rs10492385	12	29248257	0.3442	96.84%	1.000000	1.000000	0.01520000	0.03686000	NM_018099	intron	0	Hs.298851	MLSTD1
rs10495987	2	50006784	0.4094	92.98%	0.030910	0.146800	0.01520000	0.07475000	NM_138735	intron	0	Hs.637685	NRXN1
rs10502626	18	30492864	0.0525	96.84%	0.539700	0.595800	0.01520000	0.05664000	NM_032981	intron	0	Hs.643454	DTNA
rs1014106	7	41672091	0.1600	96.49%	0.653900	0.183000	0.01521000	0.07191000	NM_002192	downstream	23035	Hs.583348	INHBA
rs6753156	2	202873779	0.2435	94.39%	0.508400	0.361200	0.01521000	0.04560000	NM_015934	intron	0	Hs.471104	NOP5/NOP5
rs1458438	6	78325600	0.2830	92.98%	0.649300	0.307600	0.01522000	0.02721000	NM_000863	upstream	95700	Hs.123016	HTR1B
rs2175953	13	60608503	0.2040	97.19%	0.008626	0.037760	0.01523000	0.07754000	NM_022843	downstream	273316	Hs.391781	PCDH20
rs2399460	3	114254195	0.2164	94.04%	0.209600	0.047570	0.01523000	0.03588000	NM_033254	upstream	159870	Hs.591318	BOC
rs1356441	12	82120457	0.4539	95.09%	0.327100	0.446800	0.01524000	0.01670000	NM_152588	downstream	68263	Hs.577775	TMTC2
rs10494508	1	176267144	0.1310	95.09%	0.591300	0.698000	0.01525000	0.00608400	ENST00000308284	intron	0	---	---
rs10509800	10	107510015	0.0296	94.74%	1.000000	1.000000	0.01525000	0.05218000	NM_001013031	downstream	816954	Hs.591915	SORCS1
rs232885	1	58884645	0.2032	97.54%	0.195500	0.404200	0.01526000	0.01379000	ENST00000294610	downstream	8354	---	---
rs3761919	1	203961343	0.2950	97.54%	0.885200	0.845900	0.01526000	0.03473000	NM_022731	intron	0	Hs.632458	NUCKS1
rs10505840	12	19294996	0.0699	97.89%	1.000000	1.000000	0.01527000	0.00990700	NM_019012	intron	0	Hs.188614	PLEKHA5
rs6716767	2	42412760	0.0771	88.77%	1.000000	1.000000	0.01530000	0.02125000	NM_019063	3UTR	0	Hs.593614	EML4
rs546608	4	112045354	0.1030	93.68%	1.000000	1.000000	0.01533000	0.04498000	NM_000325	upstream	281651	Hs.643588	PITX2
rs10513826	3	117647582	0.1685	94.74%	0.382100	0.094040	0.01534000	0.06936000	NM_002338	upstream	1014	Hs.657246	LSAMP
rs2748219	22	24561951	0.4770	83.86%	0.697700	0.421200	0.01535000	0.09114000	NM_032608	intron	0	Hs.417959	MYO18B
rs1392908	4	14173600	0.1661	95.09%	0.509800	0.749400	0.01536000	0.02946000	ENST00000357803	upstream	89482	---	---
rs1989627	1	171236745	0.0349	95.44%	1.000000	1.000000	0.01537000	0.01207000	NM_005092	downstream	38978	Hs.248197	TNFSF18
rs423246	6	94982708	0.4206	97.19%	0.460900	0.342800	0.01537000	0.05548000	ENST00000386414	downstream	230854	---	---
rs10484900	6	108657232	0.1075	97.89%	0.538400	0.512600	0.01539000	0.11580000	NM_003795	intron	0	Hs.12102	SNX3
rs4783035	16	81046985	0.1654	94.39%	0.266700	1.000000	0.01542000	0.05969000	NM_001257	upstream	402475	Hs.654386	CDH13
rs1910650	21	24343089	0.3229	95.09%	0.070270	0.026540	0.01544000	0.10630000	ENST00000355181	upstream	440052	---	---
rs1915602	10	66125009	0.1728	67.02%	0.213500	0.279400	0.01544000	0.01352000	NM_001001330	downstream	1070122	Hs.499833	REEP3
rs2691390	4	57905571	0.3423	97.89%	0.145500	0.022450	0.01544000	0.04489000	NM_001553	upstream	234263	Hs.479808	IGFBP7
rs10512374	9	109775385	0.1147	93.33%	0.130200	0.138100	0.01545000	0.08785000	ENST00000297812	upstream	138454	---	---
rs2631267	4	103167495	0.1358	92.98%	0.014530	0.000126	0.01545000	0.11900000	NM_017935	intron	0	Hs.480400	BANK1
rs6940582	6	124724287	0.1105	96.84%	0.349800	0.326100	0.01546000	0.04142000	NM_001040214	intron	0	Hs.656604	NKAIN2
rs10497035	2	149022511	0.4259	92.28%	0.077010	0.037240	0.01547000	0.00456200	NM_018328	downstream	34999	Hs.458312	MBD5
rs3808747	9	17616616	0.1320	94.39%	1.000000	0.697500	0.01547000	0.00347600	NM_003026	intron	0	Hs.75149	SH3GL2
rs261212	5	95967121	0.3947	93.33%	0.898100	0.756800	0.01548000	0.00928500	NM_001042443	upstream	97140	Hs.440961	CAST
rs9293020	5	22556305	0.3321	92.98%	1.000000	0.855500	0.01548000	0.02851000	NM_004061	intron	0	Hs.113684	CDH12
rs1392409	5	85119970	0.2912	91.58%	0.652400	0.600300	0.01549000	0.11780000	ENST00000333189	downstream	238446	---	---
rs9301795	13	91765949	0.0430	97.89%	1.000000	1.000000	0.01550000	0.01080000	NM_004466	intron	0	Hs.655675	GPC5
rs2312933	6	76634385	0.0500	84.21%	0.106500	0.139800	0.01551000	0.06571000	NM_004999	intron	0	Hs.149387	MYO6
rs12455845	18	1427602	0.0400	87.72%	1.000000	1.000000	0.01553000	0.02735000	NM_022840	downstream	1099928	Hs.126888	METTL4
rs10487393	7	117664649	0.0646	95.09%	0.302400	1.000000	0.01554000	0.08257000	NM_019644	intron	0	Hs.657737	ANKRD7
rs2374334	7	90123410	0.2292	97.19%	0.865500	1.000000	0.01554000	0.02283000	ENST00000306129	intron	0	---	---
rs3757797	7	117659274	0.0646	95.09%	0.302400	1.000000	0.01554000	0.08257000	NM_019644	intron	0	Hs.657737	ANKRD7
rs3913016	12	57002855	0.1476	95.09%	0.627200	1.000000	0.01556000	0.02504000	ENST00000354328	downstream	5576	---	---
rs10499999	7	110196597	0.2966	92.28%	1.000000	0.734500	0.01557000	0.01758000	NM_032549	intron	0	Hs.655722	IMMP2L
rs9312227	4	169008236	0.0368	95.44%	0.042590	0.064750	0.01557000	0.05735000	NM_007193	upstream	242074	Hs.188401	ANXA10
rs2373478	11	79727297	0.3011	96.14%	0.316200	0.557700	0.01559000	0.00947300	ENST00000363276	upstream	189355	---	---
rs733046	4	32076883	0.4524	95.79%	0.902900	0.763500	0.01559000	0.00160100	NM_032457	downstream	1323314	Hs.570785	PCDH7
rs2032092	21	38704841	0.2306	90.53%	0.290800	0.046410	0.01561000	0.01419000	NM_004449	intron	0	Hs.473819	ERG
rs2465764	11	106419130	0.1933	94.39%	1.000000	0.782800	0.01561000	0.00205100	NM_000855	upstream	24749	Hs.654555	GUCY1A2
rs3749214	3	158465967	0.3926	94.74%	0.798400	0.878700	0.01561000	0.02133000	NM_024621	intron	0	Hs.658046	VEPH1

rs1389563	9	586826	0.2809	93.68%	0.094380	0.078840	0.01562000	0.00649000	NM_015158	intron	0	Hs.306764	ANKRD15
rs728695	9	84651341	0.3448	91.58%	0.681000	0.418100	0.01564000	0.01815000	NM_152573	downstream	132979	Hs.657750	RASEF
rs9302112	15	40607743	0.4281	97.54%	0.902600	0.549500	0.01565000	0.06676000	NM_130798	intron	0	Hs.511149	SNAP23
rs231948	6	155783805	0.4332	97.19%	0.807300	0.547100	0.01568000	0.00796200	NM_015718	intron	0	Hs.247776	NOX3
rs10509278	10	68476319	0.0535	95.09%	0.546600	0.595800	0.01570000	0.09583000	NM_013266	intron	0	Hs.660362	CTNNA3
rs10516731	4	86217807	0.0535	95.09%	0.546600	0.595800	0.01570000	0.06595000	ENST00000365031	upstream	22866	---	---
rs1718794	4	171447101	0.4725	89.47%	0.452000	0.440300	0.01570000	0.09407000	NM_182662	upstream	199154	Hs.529735	AADAT
rs9309658	2	28110335	0.3560	90.18%	0.021410	0.077480	0.01570000	0.09788000	NM_199192	intron	0	Hs.258314	BRE
rs154152	5	14433324	0.1601	71.23%	0.793000	1.000000	0.01572000	0.00901500	NM_007118	intron	0	Hs.130031	TRIO
rs10923960	1	116378633	0.0648	94.74%	0.304000	0.298600	0.01573000	0.03456000	NM_018420	intron	0	Hs.125482	SLC22A15
rs2945075	11	28833042	0.4218	91.93%	0.799900	0.417900	0.01574000	0.07091000	NM_152636	downstream	521523	Hs.243326	METT5D1
rs10495175	1	219889769	0.3616	95.09%	0.237200	0.731300	0.01575000	0.06062000	NM_144729	downstream	51620	Hs.497822	DUSP10
rs10490863	3	133613652	0.1803	81.75%	0.825200	0.811300	0.01576000	0.09185000	NM_015268	upstream	22433	Hs.12707	DNAJC13
rs10491803	9	94652670	0.0072	97.54%	1.000000	1.000000	0.01577000	0.99870000	NM_001010925	intron	0	---	ANKRD19
rs7909489	10	20627497	0.3258	93.68%	0.404400	1.000000	0.01577000	0.01507000	NM_032812	downstream	18205	Hs.658134	PLXDC2
rs1732351	12	40756788	0.3520	97.19%	1.000000	1.000000	0.01578000	0.03004000	ENST00000280876	downstream	10525	---	---
rs961090	15	38404706	0.4708	96.14%	0.089260	0.361700	0.01578000	0.13940000	NM_207380	downstream	6301	Hs.32433	C15orf52
rs10494964	1	212033510	0.4981	90.88%	0.709200	0.759700	0.01579000	0.07096000	ENST00000362373	downstream	148166	---	---
rs6509546	19	56710286	0.1850	89.12%	0.837100	0.822700	0.01581000	0.00618400	NM_198846	downstream	4510	Hs.397255	SIGLEC6
rs6581827	12	67170158	0.3675	82.11%	0.259900	0.738400	0.01581000	0.00499300	NM_017440	upstream	157779	Hs.655702	MDM1
rs953323	6	4381298	0.4653	96.14%	0.274600	1.000000	0.01583000	0.04791000	NM_001011540	upstream	174547	---	KU-MEL-3
rs2863171	11	45207308	0.1395	81.75%	0.585500	0.696300	0.01584000	0.07116000	NM_020229	downstream	4094	Hs.178715	PRDM11
rs4085170	11	102307932	0.1726	78.25%	0.017170	0.021220	0.01589000	0.06951000	NM_002427	downstream	11005	Hs.2936	MMP13
rs7140637	14	49892268	0.3959	94.39%	0.310800	0.188600	0.01589000	0.10860000	NM_004196	intron	0	Hs.705385	CDKL1
rs2077713	20	166783	0.1165	93.33%	0.765800	0.752000	0.01594000	0.00669700	NM_080831	downstream	8256	Hs.112087	DEFB129
rs10517742	4	162421722	0.2884	93.68%	1.000000	0.862600	0.01596000	0.03657000	NM_020116	downstream	102779	Hs.591707	FSTL5
rs660274	11	84482949	0.3444	84.56%	0.478500	0.706400	0.01596000	0.06689000	ENST00000376104	intron	0	---	---
rs1025226	15	40001517	0.1148	94.74%	0.763400	1.000000	0.01598000	0.02001000	NM_139265	intron	0	Hs.143703	EHD4
rs1384370	18	64079465	0.0833	96.84%	0.415400	0.499000	0.01598000	0.01248000	NM_019022	downstream	412442	Hs.440534	TXNDC10
rs2859443	22	42062368	0.2928	92.28%	0.458600	0.544700	0.01598000	0.02052000	NM_173050	intron	0	Hs.133995	SCUBE1
rs9285582	14	54156618	0.4442	84.91%	0.363900	0.342800	0.01599000	0.03623000	NM_015589	intron	0	Hs.98259	SAMD4A
rs1157819	1	207671357	0.3634	83.51%	0.000153	0.017170	0.01600000	0.00865700	ENST00000367032	5UTR	0	---	---
rs10485681	20	39926486	0.0759	94.74%	1.000000	1.000000	0.01601000	0.07055000	NM_007050	downstream	208320	Hs.526879	PTPRT
rs10502605	18	28542164	0.2975	97.89%	0.566200	0.130900	0.01601000	0.17600000	NM_020805	intron	0	Hs.446164	KLHL14
rs10520944	5	28874857	0.1625	97.19%	0.505200	0.467300	0.01602000	0.03563000	ENST00000333399	upstream	86607	---	---
rs10509043	10	57304421	0.0824	97.89%	0.705400	0.698300	0.01603000	0.06229000	NM_001005414	downstream	482784	---	ZWINT
rs360380	2	126836951	0.0824	97.89%	1.000000	1.000000	0.01603000	0.08453000	NM_016815	upstream	327593	Hs.59138	GYPC
rs4593265	5	125188437	0.4800	96.49%	0.717300	1.000000	0.01604000	0.01028000	NM_023927	upstream	598563	Hs.363558	GRAMD3
rs10503648	8	19062324	0.0839	96.14%	1.000000	1.000000	0.01605000	0.01294000	NM_022071	upstream	153084	Hs.303208	SH2D4A
rs10507313	13	22170145	0.0377	92.98%	0.310800	1.000000	0.01605000	0.03527000	ENST00000362980	upstream	105139	---	---
rs2880538	1	240840987	0.0562	93.68%	0.578000	1.000000	0.01605000	0.07800000	NM_152666	upstream	86366	Hs.672452	PLD5
rs6724513	2	20755964	0.1478	86.67%	0.038210	0.011750	0.01608000	0.10910000	NM_021925	intron	0	Hs.187823	C2orf43
rs10495585	2	11909579	0.0326	91.58%	0.001225	0.002736	0.01610000	0.18560000	NM_145693	downstream	24597	Hs.467740	LPIN1
rs2526632	7	18999334	0.4903	90.88%	0.047100	0.285700	0.01610000	0.02576000	NM_178425	intron	0	Hs.196054	HDAC9
rs10508146	13	103854808	0.0756	95.09%	0.184300	0.198700	0.01611000	0.11270000	NM_172370	upstream	1061785	Hs.381382	DAOA
rs9319012	13	83522733	0.3209	94.04%	1.000000	0.576500	0.01611000	0.03381000	NM_052910	upstream	168204	Hs.415478	SLITRK1
rs10495130	1	217872013	0.0318	93.68%	0.231100	0.300000	0.01613000	0.03843000	NM_138794	downstream	419183	Hs.657617	LYPLAL1
rs489565	1	225376830	0.3180	87.72%	0.561700	0.493300	0.01614000	0.04356000	NM_003607	intron	0	Hs.35433	CDC42BPA
rs1386286	3	151326543	0.2066	90.88%	1.000000	1.000000	0.01615000	0.00953800	ENST00000386021	upstream	48235	---	---
rs1496939	4	190243275	0.4049	94.04%	0.128700	0.646000	0.01618000	0.11100000	ENST00000378771	downstream	148725	---	---
rs1073731	9	140010194	0.0072	97.89%	1.000000	1.000000	0.01619000	0.99850000	NM_000718	intron	0	Hs.495522	CACNA1B
rs1479237	2	4619354	0.1513	95.09%	0.351200	0.313400	0.01619000	0.01419000	ENST00000387792	downstream	233112	---	---
rs1869941	4	190586616	0.4768	90.88%	0.803600	0.532700	0.01619000	0.02012000	ENST00000378770	upstream	44679	---	---
rs2327477	6	134834477	0.0953	97.54%	0.290400	0.227400	0.01619000	0.00998100	NM_005627	upstream	296797	Hs.510078	SGK1

rs9293311	5	82063886	0.3333	94.74%	0.412800	1.000000	0.01619000	0.07002000	NM_174909	downstream	320537	Hs.355606	TMEM167
rs953497	6	130500818	0.0072	97.89%	0.010760	1.000000	0.01619000	0.99850000	NM_001007102	intron	0	Hs.658051	L3MBTL3
rs10519764	15	30986441	0.2050	97.54%	1.000000	1.000000	0.01620000	0.01245000	ENST00000334528	intron	0	---	---
rs348566	5	40343736	0.2135	96.14%	0.369200	1.000000	0.01620000	0.02396000	ENST00000362491	downstream	38158	---	---
rs6501346	17	65321736	0.1836	96.49%	0.840000	1.000000	0.01620000	0.15270000	NM_002758	downstream	270669	Hs.463978	MAP2K6
rs10492184	12	5569309	0.0964	96.49%	0.294400	1.000000	0.01621000	0.04945000	NM_020373	intron	0	Hs.148970	TMEM16B
rs1501045	18	4941575	0.2860	95.09%	1.000000	0.388600	0.01621000	0.01087000	ENST00000320108	downstream	285788	---	---
rs10517410	4	37045599	0.0842	97.89%	0.704400	0.697500	0.01622000	0.00571900	ENST00000389006	intron	0	---	---
rs959170	1	21236770	0.0877	94.04%	1.000000	1.000000	0.01623000	0.04642000	NM_003760	intron	0	Hs.467084	EIF4G3
rs1412938	13	93661657	0.4120	93.68%	0.016240	0.015910	0.01624000	0.01862000	NM_005708	intron	0	Hs.444329	GPC6
rs894246	4	77298526	0.1841	97.19%	0.315800	0.379600	0.01624000	0.00603400	NM_005506	downstream	392	Hs.349656	SCARB2
rs2826766	21	21582505	0.1371	90.88%	0.188000	0.256200	0.01626000	0.01444000	NM_004540	intron	0	Hs.473450	NCAM2
rs634283	11	81529120	0.3143	95.44%	1.000000	0.868000	0.01626000	0.01724000	ENST00000357859	upstream	549129	---	---
rs6600813	4	71252643	0.1328	95.09%	0.796000	0.424600	0.01626000	0.12060000	NM_012390	upstream	8439	Hs.701334	SMR3A
rs10491106	17	16395636	0.0317	94.04%	0.022680	1.000000	0.01629000	0.07125000	NM_020653	3UTR	0	Hs.99724	ZNF287
rs10505996	12	26393672	0.0314	95.09%	1.000000	1.000000	0.01630000	0.05181000	NM_002223	intron	0	Hs.512235	ITPR2
rs10507127	12	99914189	0.0975	97.19%	0.731700	0.602900	0.01631000	0.02342000	NM_178826	intron	0	Hs.58785	TMEM16D
rs1322100	6	77796828	0.2509	95.09%	0.014500	0.016160	0.01632000	0.02636000	NM_000863	downstream	431839	Hs.123016	HTR1B
rs1445420	3	145323434	0.1729	94.39%	0.670300	0.644200	0.01632000	0.04652000	NM_173552	downstream	129541	Hs.288954	C3orf58
rs10485313	6	65977375	0.2230	94.39%	0.292100	0.628800	0.01634000	0.01039000	ENST00000370615	downstream	85103	---	---
rs336030	2	38120500	0.4649	84.91%	0.013920	0.193200	0.01634000	0.05894000	NM_144713	intron	0	Hs.591566	FAM82A
rs10502665	18	32530580	0.1100	87.72%	0.514100	0.299900	0.01636000	0.02309000	NM_025135	intron	0	Hs.436636	FHOD3
rs2187753	22	48070584	0.3587	96.84%	0.600700	1.000000	0.01638000	0.00853000	NM_207478	upstream	257749	---	FLJ44385
rs723105	3	119284498	0.2672	81.40%	0.067210	0.436600	0.01638000	0.15970000	NM_001015887	downstream	819193	Hs.112873	IGSF11
rs10501062	11	26642651	0.1787	92.28%	0.402800	1.000000	0.01639000	0.03166000	NM_031418	downstream	1240	Hs.91791	TMEM16C
rs6067805	20	49568498	0.1208	92.98%	0.078770	0.652300	0.01640000	0.02188000	NM_173091	intron	0	Hs.699441	NFATC2
rs7560892	2	23782141	0.3451	79.30%	0.039530	0.005985	0.01640000	0.01264000	ENST00000288548	intron	0	---	---
rs7895806	10	29278534	0.0894	92.28%	0.136100	0.131700	0.01640000	0.03359000	NM_032517	upstream	339462	Hs.558572	LYZL1
rs10519620	2	4242738	0.0370	94.74%	1.000000	1.000000	0.01641000	0.04771000	ENST00000382050	downstream	513237	---	---
rs10506611	12	69537397	0.4636	96.49%	0.544100	0.132200	0.01642000	0.02553000	NM_002849	intron	0	Hs.506076	PTPRR
rs2057664	7	25378628	0.3087	97.19%	0.778800	1.000000	0.01643000	0.06549000	ENST00000365363	downstream	107603	---	---
rs10515345	5	102814058	0.1867	87.37%	0.303400	0.215500	0.01644000	0.07363000	NM_031438	downstream	98397	Hs.434289	NUDT12
rs6844026	4	70418580	0.2698	97.54%	0.445800	0.381900	0.01644000	0.17220000	NM_021139	upstream	22368	Hs.285887	UGT2B4
rs1882256	3	172933521	0.3579	97.54%	1.000000	0.494600	0.01645000	0.09730000	NM_002662	intron	0	Hs.382865	PLD1
rs4852483	2	79454929	0.1194	94.04%	0.556100	0.649800	0.01647000	0.11610000	NM_138937	upstream	214542	Hs.567312	REG3A
rs10515062	5	67343968	0.3745	88.07%	0.180000	0.375800	0.01648000	0.08447000	NM_181524	upstream	276040	Hs.132225	PIK3R1
rs955638	4	183491655	0.2079	97.89%	0.855800	1.000000	0.01648000	0.02239000	ENST00000389043	intron	0	---	---
rs562125	9	109774984	0.3829	88.42%	0.350800	0.862000	0.01649000	0.00681600	ENST00000297812	upstream	138053	---	---
rs10503430	8	12902127	0.1286	96.84%	1.000000	1.000000	0.01651000	0.02932000	NM_001039462	intron	0	---	C8orf79
rs10505338	8	119824671	0.1734	95.09%	0.401100	0.242100	0.01654000	0.00980400	NM_207506	upstream	121370	Hs.492653	SAMD12
rs1956455	14	93711620	0.1415	90.53%	0.000052	0.001412	0.01654000	0.04000000	NM_020958	intron	0	Hs.259599	KIAA1622
rs2193135	14	79517999	0.1846	97.89%	0.426000	0.261500	0.01654000	0.02958000	NM_138970	downstream	117488	Hs.368307	NRXN3
rs7634356	3	181630586	0.4583	84.21%	0.027170	0.024080	0.01654000	0.08327000	ENST00000364940	upstream	112597	---	---
rs2063428	6	165167856	0.4856	97.19%	0.718300	1.000000	0.01657000	0.01666000	NM_144980	downstream	445300	Hs.144734	C6orf118
rs1112762	10	8347412	0.4982	96.49%	1.000000	0.451100	0.01658000	0.05593000	NM_002051	downstream	190242	Hs.524134	GATA3
rs1578792	10	37183267	0.1333	94.74%	0.594900	1.000000	0.01658000	0.16970000	NM_052997	upstream	271524	Hs.373787	ANKRD30A
rs7097123	10	107583441	0.0461	95.09%	1.000000	1.000000	0.01658000	0.01412000	NM_001013031	downstream	743528	Hs.591915	SORCS1
rs710765	12	68277894	0.1970	94.39%	0.334900	0.670400	0.01658000	0.02874000	NM_006431	intron	0	Hs.189772	CCT2
rs1993059	4	42551651	0.4585	92.98%	0.902000	0.756700	0.01659000	0.02105000	NM_006095	upstream	197992	Hs.435052	ATP8A1
rs2174623	4	125925394	0.3389	94.74%	0.134500	0.592200	0.01663000	0.06871000	NM_020337	upstream	74012	Hs.480694	ANKRD50
rs9283491	2	184877562	0.2509	94.39%	0.516900	0.711900	0.01665000	0.03445000	NM_194250	upstream	294370	Hs.159528	ZNF804A
rs782945	15	59125923	0.4712	97.54%	0.809700	1.000000	0.01667000	0.12190000	NM_134261	intron	0	Hs.695914	RORA
rs952840	5	86135661	0.4427	97.89%	0.904000	0.881100	0.01667000	0.00814100	NM_001039781	downstream	245858	---	LOC645261
rs10485818	20	168303	0.1059	82.81%	1.000000	1.000000	0.01668000	0.00416800	NM_080831	downstream	9776	Hs.112087	DEFB129

rs10484097	14	85835026	0.4389	91.93%	0.381300	0.749500	0.01670000	0.13100000	NM_013231	downstream	671004	Hs.533710	FLRT2
rs167527	5	3189229	0.1030	93.68%	0.499200	0.483700	0.01670000	0.12360000	NM_178569	downstream	380718	Hs.668017	C5orf38
rs10510019	10	118183010	0.3651	97.54%	1.000000	0.756800	0.01671000	0.03348000	NM_001011709	intron	0	Hs.276724	PNLIPRP3
rs3755806	3	52618725	0.5000	96.84%	0.337000	0.649000	0.01671000	0.09870000	NM_181042	CDS	0	Hs.189920	PBRM1
rs1400549	11	103245119	0.4606	97.89%	0.040170	0.125400	0.01672000	0.00823600	NM_033135	downstream	38012	Hs.352298	PDGFD
rs1892917	9	18100746	0.4089	94.39%	0.528300	0.285200	0.01672000	0.11040000	NM_003026	downstream	313619	Hs.75149	SH3GL2
rs10507243	12	113193181	0.4419	93.68%	0.459400	0.226400	0.01673000	0.01308000	NM_181486	downstream	82938	Hs.381715	TBX5
rs4074785	9	21971583	0.2690	97.19%	0.091520	0.536800	0.01673000	0.01103000	NM_058195	intron	0	Hs.512599	CDKN2A
rs10498990	6	95943353	0.2440	88.42%	0.608600	0.475100	0.01674000	0.01986000	NM_024641	upstream	188781	Hs.533323	MANEA
rs10494648	1	189080190	0.2441	89.82%	0.234400	0.702600	0.01679000	0.02132000	NM_199051	upstream	366808	Hs.65765	FAM5C
rs10498556	14	82446133	0.1963	94.74%	1.000000	0.032720	0.01679000	0.01098000	ENST00000362427	upstream	138542	---	---
rs6879726	5	81990043	0.3127	90.88%	0.000293	0.000162	0.01679000	0.17910000	NM_001039779	downstream	271952	---	FLJ41309
rs801760	7	18559723	0.0180	97.54%	0.079050	0.121100	0.01679000	0.99780000	NM_178425	intron	0	Hs.196054	HDAC9
rs2953617	7	133324640	0.3000	92.98%	0.558500	0.865000	0.01682000	0.08069000	NM_001037126	intron	0	Hs.321273	EXOC4
rs885685	7	48898843	0.4130	94.74%	0.133600	0.546100	0.01683000	0.05486000	ENST00000324993	upstream	35860	---	---
rs1152344	14	82253091	0.3810	66.32%	0.644500	0.720500	0.01684000	0.06620000	ENST00000387641	downstream	254807	---	---
rs1915761	2	79079645	0.0375	93.68%	0.308800	0.368900	0.01685000	0.01085000	NM_198448	upstream	26689	Hs.447084	REG3G
rs533480	10	97222195	0.2007	94.39%	0.703200	1.000000	0.01687000	0.00521900	NM_001034957	intron	0	Hs.696027	SORBS1
rs7790890	7	108037207	0.4310	94.04%	0.709600	0.652500	0.01687000	0.01838000	NM_012328	downstream	34677	Hs.6790	DNAJB9
rs10493753	1	84764257	0.0195	90.18%	0.002356	0.005609	0.01689000	0.99740000	NM_022354	CDS	0	---	SPATA1
rs10503705	8	21325431	0.0072	96.84%	1.000000	1.000000	0.01690000	0.99850000	ENST00000387614	upstream	174821	---	---
rs7834726	8	106866555	0.0370	94.74%	0.003297	0.005568	0.01690000	0.08650000	NM_012082	intron	0	Hs.431009	ZFPM2
rs6491544	13	99549209	0.4856	97.54%	0.402900	0.290900	0.01691000	0.02188000	NM_000282	intron	0	Hs.80741	PCCA
rs2292225	2	25508950	0.5000	97.54%	0.230100	0.293000	0.01693000	0.03518000	NM_183361	intron	0	Hs.307720	DTNB
rs2834448	21	34622354	0.0387	95.09%	1.000000	1.000000	0.01693000	0.05103000	NM_172201	upstream	35839	Hs.551521	KCNE2
rs2272504	12	71255465	0.2174	80.70%	0.334400	0.017590	0.01696000	0.04893000	NM_013381	intron	0	Hs.199814	TRHDE
rs333047	8	19094091	0.4838	97.54%	0.151500	0.655100	0.01697000	0.03265000	NM_022071	upstream	121317	Hs.303208	SH2D4A
rs10515811	5	159913158	0.1347	95.09%	0.599400	0.781000	0.01698000	0.02473000	ENST00000385201	downstream	68123	---	---
rs3732136	2	58240504	0.2112	97.19%	0.473200	0.456300	0.01699000	0.01259000	NM_006296	3UTR	0	Hs.631890	VRK2
rs224939	5	81752858	0.3333	94.74%	0.101400	0.856800	0.01700000	0.20210000	NM_001039779	downstream	34767	---	FLJ41309
rs6437541	3	105996424	0.1655	96.49%	0.827200	0.628200	0.01700000	0.09061000	ENST00000388649	downstream	16028	---	---
rs2032203	21	20598845	0.4342	93.33%	0.382000	0.267900	0.01701000	0.01956000	ENST00000387009	downstream	51086	---	---
rs4344605	13	54680266	0.3718	97.19%	0.897700	0.866700	0.01703000	0.02714000	ENST00000258651	upstream	766815	---	---
rs1371088	12	89184864	0.2419	97.19%	0.032170	0.092260	0.01704000	0.02992000	ENST00000388111	downstream	539934	---	---
rs1887129	13	57483769	0.3967	95.09%	0.899700	0.877700	0.01706000	0.02708000	ENST00000365552	downstream	20171	---	---
rs10490206	2	40204350	0.4676	97.54%	0.029940	0.052180	0.01707000	0.10680000	NM_021097	intron	0	Hs.468274	SLC8A1
rs9311935	3	65128739	0.2623	92.98%	0.038130	0.049300	0.01708000	0.02663000	NM_001033057	downstream	188354	Hs.651939	MAGI1
rs10519718	5	122481458	0.2191	88.07%	0.853700	0.206800	0.01709000	0.07690000	ENST00000261364	intron	0	---	---
rs10494789	1	197532204	0.3265	94.04%	0.038220	0.868200	0.01710000	0.05956000	ENST00000385491	upstream	284666	---	---
rs2219610	3	30737665	0.2126	89.12%	1.000000	0.792600	0.01710000	0.10030000	NM_207359	downstream	5031	Hs.657052	GADL1
rs10488874	4	108640002	0.0369	95.09%	0.304900	0.363800	0.01711000	0.07891000	NM_005443	downstream	114270	Hs.368610	PAPSS1
rs10515812	5	159927389	0.3563	91.58%	0.078650	0.157800	0.01711000	0.09190000	ENST00000327245	intron	0	---	---
rs2849380	18	59130340	0.1556	94.74%	0.816800	1.000000	0.01712000	0.04608000	NM_000657	intron	0	Hs.150749	BCL2
rs413853	1	75064286	0.4731	97.89%	0.719300	0.881700	0.01712000	0.02758000	NM_138467	downstream	60390	Hs.348411	TYW3
rs1368120	4	70438719	0.2706	97.89%	0.448700	0.381900	0.01713000	0.19220000	NM_021139	upstream	42507	Hs.285887	UGT2B4
rs2591205	5	13687084	0.1641	89.82%	0.255000	0.741400	0.01714000	0.07453000	NM_001369	downstream	56353	Hs.212360	DNAH5
rs440834	9	10091415	0.4101	93.68%	0.527500	0.073020	0.01714000	0.00273400	ENST00000363183	downstream	659063	---	---
rs6699473	1	156761833	0.4755	92.98%	0.267900	0.759800	0.01714000	0.13910000	NM_001005189	downstream	21709	Hs.553780	OR6Y1
rs920956	13	92901302	0.2104	90.88%	0.350100	1.000000	0.01714000	0.02537000	NM_005708	intron	0	Hs.444329	GPC6
rs10514305	5	88669444	0.0558	94.39%	0.575100	1.000000	0.01715000	0.01576000	NM_002397	upstream	454626	Hs.699175	MEF2C
rs10518943	15	55942641	0.4147	88.42%	0.069450	0.030050	0.01715000	0.01641000	NM_170697	downstream	90279	Hs.699620	ALDH1A2
rs10487719	7	31309586	0.0236	96.49%	0.135100	1.000000	0.01716000	0.02110000	NM_022728	downstream	34021	Hs.45152	NEUROD6
rs10501722	11	89921778	0.2312	93.33%	1.000000	1.000000	0.01716000	0.01259000	NM_012124	upstream	325951	Hs.22857	CHORDC1
rs1392909	4	14176869	0.1898	96.14%	0.236800	0.387800	0.01717000	0.01076000	ENST00000357803	upstream	92751	---	---

rs17626707	13	27870743	0.2330	92.63%	0.057400	0.442000	0.01724000	0.06120000	NM_002019	intron	0	Hs.654360	FLT1
rs4442876	17	36446323	0.1087	88.77%	1.000000	1.000000	0.01725000	0.21420000	NM_030966	downstream	4319	Hs.534495	KRTAP1-3
rs8102512	19	36360499	0.4387	94.39%	0.620400	0.754400	0.01725000	0.20410000	NM_020856	downstream	98341	Hs.278436	TSHZ3
rs1948270	21	19679846	0.3340	87.72%	0.117700	0.262900	0.01726000	0.02514000	ENST00000387002	downstream	40346	---	---
rs3908410	11	35546863	0.0242	94.39%	1.000000	1.000000	0.01727000	0.01369000	NM_001001991	upstream	42708	Hs.55044	DKFZP586H
rs4575607	18	34747458	0.0993	97.19%	0.739600	1.000000	0.01727000	0.02417000	NM_020180	upstream	1347517	Hs.435976	BRUNOL4
rs542873	1	57487117	0.3656	97.89%	0.155400	0.352800	0.01727000	0.04592000	NM_021080	intron	0	Hs.477370	DAB1
rs10500833	11	18595429	0.1480	87.72%	1.000000	1.000000	0.01728000	0.00248000	NM_194285	intron	0	Hs.268668	SPTY2D1
rs2551397	18	59105557	0.3457	94.39%	0.592800	0.481000	0.01729000	0.02347000	NM_000657	intron	0	Hs.150749	BCL2
rs10504450	8	70626079	0.1221	91.93%	0.144300	0.131300	0.01730000	0.05355000	NM_015170	intron	0	Hs.409602	SULF1
rs4728928	7	90161222	0.3577	91.23%	0.788200	0.211300	0.01731000	0.03267000	ENST00000306129	intron	0	---	---
rs10493113	1	42931097	0.1864	97.89%	0.329700	0.577400	0.01732000	0.01540000	NM_004559	intron	0	Hs.473583	YBX1
rs1010390	13	80764540	0.4030	83.16%	0.080040	0.489400	0.01733000	0.00315400	ENST00000387380	downstream	26588	---	---
rs1506065	4	114151779	0.3183	97.54%	0.272200	0.141900	0.01733000	0.08129000	NM_020977	upstream	38540	Hs.620557	ANK2
rs1598978	12	113008816	0.3963	94.74%	0.525400	0.321600	0.01733000	0.08420000	NM_016196	upstream	120322	Hs.7482	RBM19
rs10490110	2	59251013	0.2660	92.98%	0.752700	0.674300	0.01734000	0.01262000	ENST00000365640	upstream	151806	---	---
rs2837758	21	40940879	0.3723	97.54%	0.522800	0.122200	0.01735000	0.00687100	NM_206887	intron	0	---	DSCAM
rs3012068	10	126707120	0.0198	88.42%	1.000000	1.000000	0.01736000	0.99790000	NM_001329	intron	0	Hs.501345	CTBP2
rs10485499	20	30677558	0.1774	92.98%	0.289900	0.483100	0.01737000	0.05777000	NM_001010976	downstream	17202	---	RP11-410N
rs1442426	1	192267576	0.2296	94.74%	0.119800	0.250300	0.01737000	0.01222000	ENST00000385496	upstream	299447	---	---
rs10490803	3	171691928	0.1691	96.49%	0.084440	0.058140	0.01738000	0.05132000	NM_020949	intron	0	Hs.674783	SLC7A14
rs10498561	14	83013970	0.0735	97.89%	0.648800	0.395800	0.01738000	0.02143000	ENST00000387648	downstream	110158	---	---
rs10505136	8	111210397	0.0735	97.89%	0.648800	1.000000	0.01738000	0.05149000	NM_014379	upstream	154262	Hs.13285	KCNV1
rs721569	2	185553368	0.0656	77.54%	0.232000	0.266700	0.01738000	0.02116000	NM_194250	downstream	41370	Hs.159528	ZNF804A
rs1285405	7	9234454	0.2509	95.09%	0.003374	0.026270	0.01740000	0.04686000	NM_152745	downstream	476530	Hs.487564	NXPH1
rs2317212	3	98810974	0.3804	96.84%	0.004981	0.013510	0.01740000	0.03703000	ENST00000389672	intron	0	---	---
rs304861	5	152699455	0.0743	96.84%	0.650900	1.000000	0.01740000	0.02773000	NM_000827	upstream	151044	Hs.519693	GRIA1
rs955612	1	232783216	0.1679	97.19%	0.386700	0.340700	0.01740000	0.06767000	NM_005646	upstream	101744	Hs.498115	TARBP1
rs1038832	3	175168617	0.2251	95.09%	0.484900	0.333700	0.01741000	0.10820000	NM_014932	intron	0	Hs.478289	NLGN1
rs1903905	10	109261677	0.3284	95.09%	0.890400	0.854200	0.01744000	0.02618000	ENST00000363209	downstream	50260	---	---
rs6753044	2	202873678	0.1399	94.04%	0.617700	0.467700	0.01744000	0.03513000	NM_015934	intron	0	Hs.471104	NOP5/NOP5
rs2295135	14	87963963	0.1667	97.89%	0.524700	1.000000	0.01747000	0.04433000	NM_001040428	intron	0	Hs.525518	SPATA7
rs1069977	3	64085175	0.1429	90.88%	0.798300	0.701500	0.01748000	0.26790000	NM_198859	intron	0	Hs.699317	PRICKLE2
rs168718	5	80477429	0.2441	89.82%	0.313000	0.565200	0.01748000	0.02453000	NM_006909	intron	0	Hs.162129	RASGRF2
rs215749	7	32424097	0.2886	86.32%	0.534600	1.000000	0.01749000	0.05737000	ENST00000381651	upstream	30827	---	---
rs3808227	7	90242612	0.1578	92.28%	0.487000	0.451300	0.01751000	0.01666000	NM_012395	intron	0	Hs.430742	PFTK1
rs1853326	13	36896714	0.2247	93.68%	0.219700	0.626700	0.01753000	0.01492000	NM_006475	downstream	138065	Hs.136348	POSTN
rs9295481	6	20846555	0.3038	92.98%	0.561600	0.051220	0.01753000	0.01210000	NM_017774	intron	0	Hs.657604	CDKAL1
rs2170260	8	136149422	0.0996	96.84%	0.326100	0.260500	0.01755000	0.05675000	NM_006558	upstream	389462	Hs.444558	KHDRBS3
rs16920830	8	55921493	0.0310	96.14%	1.000000	1.000000	0.01757000	0.05928000	NM_006269	downstream	215546	Hs.458845	RP1
rs1888057	13	32520695	0.1649	97.89%	0.077820	0.050860	0.01757000	0.01693000	NM_004795	intron	0	Hs.524953	KL
rs2166362	5	3695021	0.2952	95.09%	0.143200	0.124300	0.01759000	0.00880700	NM_024337	downstream	40504	Hs.424156	IRX1
rs163234	16	20559524	0.3909	96.49%	0.613900	0.744100	0.01761000	0.00776100	NM_052956	intron	0	Hs.306812	ACSM1
rs334356	9	100949193	0.1882	97.89%	0.431800	0.274300	0.01761000	0.01370000	NM_004612	intron	0	Hs.494622	TGFBR1
rs2010269	7	51090457	0.4679	92.98%	0.174400	0.363900	0.01762000	0.04157000	NM_015198	intron	0	Hs.99141	COBL
rs2333634	18	62664021	0.4591	94.39%	0.714000	0.653700	0.01763000	0.01708000	NM_021153	upstream	241825	Hs.42771	CDH19
rs4901596	14	37659956	0.4478	94.04%	0.621100	0.449300	0.01764000	0.02005000	NM_001049	upstream	86999	Hs.248160	SSTR1
rs9310824	3	26944532	0.1930	95.44%	0.845400	0.779400	0.01767000	0.13450000	NM_052953	downstream	217266	Hs.517868	LRRC3B
rs10505808	12	17679831	0.0737	97.54%	0.377900	1.000000	0.01768000	0.03324000	ENST00000383935	upstream	75304	---	---
rs198057	21	23442474	0.1319	95.79%	0.283600	0.149200	0.01771000	0.11340000	ENST00000364042	downstream	133491	---	---
rs2343606	16	24701242	0.2415	82.81%	0.860500	0.842700	0.01773000	0.06062000	NM_020847	intron	0	---	TNRC6A
rs4669244	2	8228462	0.4612	85.96%	0.002997	0.033920	0.01774000	0.11510000	NM_207358	downstream	116457	---	C2orf46
rs478455	12	165807	0.1426	92.28%	0.799500	1.000000	0.01774000	0.04387000	NM_003044	downstream	3706	Hs.437174	SLC6A12
rs1541350	17	13108765	0.2752	97.54%	0.036370	0.080520	0.01775000	0.10090000	NM_006042	downstream	230966	Hs.462270	HS3ST3A1

rs2152853	1	179791481	0.1543	94.39%	0.480900	0.730800	0.01775000	0.01951000	NM_000721	intron	0	Hs.437444	CACNA1E
rs2063944	6	58703545	0.4303	88.07%	0.094930	0.271700	0.01779000	0.01091000	NM_206908	upstream	307890	---	GUSBL2
rs7030902	9	87024911	0.3151	92.98%	0.669300	0.578800	0.01780000	0.00448200	NM_001018066	downstream	342278	Hs.494312	NTRK2
rs1603182	4	85471690	0.4111	94.74%	0.261100	0.196900	0.01781000	0.02655000	NM_006168	downstream	161770	Hs.546270	NKX6-1
rs10503076	18	58868191	0.0382	96.49%	0.050540	1.000000	0.01784000	0.02522000	NM_194449	downstream	69546	Hs.465337	PHLPP
rs10510428	3	14592929	0.2611	94.74%	0.430500	0.586300	0.01784000	0.06792000	NM_016474	upstream	75344	Hs.517820	C3orf19
rs2312244	5	128493261	0.4794	93.68%	0.624300	0.545000	0.01784000	0.07038000	NM_016048	downstream	15649	Hs.483296	ISOC1
rs4748503	10	18976085	0.4535	94.39%	0.325900	0.131000	0.01784000	0.02060000	NM_182543	intron	0	Hs.396175	NSUN6
rs9327861	5	101895776	0.2620	95.09%	0.160400	0.208900	0.01785000	0.11370000	NM_173488	upstream	33186	Hs.388874	SLCO6A1
rs953359	4	185428506	0.2756	89.12%	0.875000	1.000000	0.01786000	0.04259000	NM_153343	upstream	52493	Hs.297814	ENPP6
rs1876482	2	17226049	0.1927	96.49%	0.559600	0.576800	0.01787000	0.27870000	NM_003385	upstream	359240	Hs.444212	VSNL1
rs10498134	2	222813224	0.3736	91.58%	0.895000	0.431000	0.01788000	0.00695200	NM_181460	intron	0	Hs.42146	PAX3
rs1407043	20	52438394	0.1697	97.19%	0.669300	0.755100	0.01789000	0.11290000	NM_018431	upstream	87194	Hs.656582	DOK5
rs10502192	11	114127562	0.4831	93.33%	0.902200	0.758800	0.01790000	0.02173000	NM_182495	downstream	44067	---	FAM55B
rs10512369	9	109805180	0.2019	94.74%	1.000000	0.673300	0.01791000	0.09605000	ENST00000297812	upstream	168249	---	---
rs1411439	9	71403874	0.0480	95.09%	0.467100	0.147200	0.01791000	0.11950000	NM_001163	intron	0	Hs.695952	APBA1
rs10502535	18	25738740	0.1034	93.33%	0.174300	0.071270	0.01794000	0.02050000	ENST00000384802	downstream	708250	---	---
rs1529236	7	9211096	0.0306	97.54%	1.000000	1.000000	0.01795000	0.05499000	NM_152745	downstream	453172	Hs.487564	NXPH1
rs6804032	3	116031161	0.1791	94.04%	0.535300	1.000000	0.01795000	0.06174000	NM_015642	intron	0	Hs.655108	ZBTB20
rs9294029	6	77626834	0.3556	94.74%	0.045740	0.085680	0.01795000	0.01465000	NM_000863	downstream	601833	Hs.123016	HTR1B
rs719379	4	40537658	0.3849	97.54%	0.526200	0.220400	0.01797000	0.03285000	NM_173075	intron	0	Hs.479602	APBB2
rs385064	8	86612641	0.1854	74.74%	0.174200	1.000000	0.01799000	0.09579000	NM_000067	downstream	31696	Hs.155097	CA2
rs334961	3	18586159	0.2192	96.84%	0.078430	0.112800	0.01802000	0.00680500	NM_002971	upstream	130951	Hs.517717	SATB1
rs3771372	2	71478662	0.0926	94.74%	0.143400	1.000000	0.01802000	0.04904000	NM_001014972	intron	0	Hs.434401	ZNF638
rs2204410	7	142886781	0.1803	94.39%	0.542400	0.770100	0.01803000	0.01387000	ENST00000385583	downstream	25433	---	---
rs8055123	16	10953220	0.1260	91.93%	0.577200	1.000000	0.01803000	0.01377000	NM_015226	intron	0	Hs.35490	CLEC16A
rs1938577	1	70080009	0.1774	92.98%	0.525800	0.479900	0.01804000	0.08597000	NM_020794	intron	0	Hs.479658	LRRC7
rs3740798	11	35663356	0.0784	82.81%	0.040910	0.046770	0.01804000	0.09501000	NM_017583	intron	0	Hs.591987	TRIM44
rs719139	2	224043745	0.2296	94.74%	0.734000	0.845300	0.01805000	0.13510000	NM_003469	downstream	126161	Hs.516726	SCG2
rs10513958	18	65066396	0.0481	94.74%	1.000000	1.000000	0.01806000	0.00438600	NM_152721	upstream	152875	Hs.569915	DOK6
rs1816239	19	33307040	0.3172	97.89%	0.409400	0.853400	0.01806000	0.05203000	ENST00000379373	upstream	477982	---	---
rs1349063	12	84788368	0.2243	95.44%	1.000000	0.843700	0.01807000	0.11000000	NM_006183	upstream	3838	Hs.80962	NTS
rs1279321	14	32657928	0.1222	93.33%	0.249000	0.376800	0.01813000	0.05889000	NM_022123	intron	0	Hs.659456	NPAS3
rs9323727	14	82420103	0.1895	89.82%	0.023660	0.367900	0.01813000	0.01379000	ENST00000362427	upstream	164572	---	---
rs4131048	10	124981713	0.3795	97.54%	0.612000	0.739800	0.01814000	0.03881000	NM_153442	upstream	434148	Hs.12751	GPR26
rs10496839	2	140766339	0.2482	96.14%	0.517100	1.000000	0.01816000	0.03956000	NM_018557	intron	0	Hs.656461	LRP1B
rs10485064	6	85653291	0.0185	95.09%	1.000000	1.000000	0.01818000	0.99780000	ENST00000230608	upstream	122673	---	---
rs10496804	2	139392949	0.0307	97.19%	1.000000	1.000000	0.01818000	0.01092000	ENST00000363563	upstream	55014	---	---
rs1885373	9	35686802	0.0185	95.09%	1.000000	1.000000	0.01818000	0.99780000	NM_006289	downstream	536	Hs.471014	TLN1
rs496289	11	36484917	0.0185	95.09%	1.000000	1.000000	0.01818000	0.99780000	NM_004620	intron	0	Hs.591983	TRAF6
rs7984449	13	52641443	0.0185	95.09%	0.081040	0.121800	0.01818000	0.99770000	NM_006418	downstream	117256	Hs.559736	OLFM4
rs962353	11	36521184	0.0185	95.09%	1.000000	1.000000	0.01818000	0.99780000	NM_000448	upstream	24955	Hs.73958	RAG1
rs10487395	7	117678691	0.0645	97.89%	0.314600	1.000000	0.01819000	0.06408000	NM_019644	downstream	8714	Hs.657737	ANKRD7
rs2057684	7	117678408	0.0645	97.89%	0.314600	1.000000	0.01819000	0.06408000	NM_019644	downstream	8431	Hs.657737	ANKRD7
rs2057685	7	117678603	0.0645	97.89%	0.314600	1.000000	0.01819000	0.06408000	NM_019644	downstream	8626	Hs.657737	ANKRD7
rs4947461	7	54377737	0.3851	91.58%	0.515900	0.866200	0.01819000	0.00168300	NM_001001707	upstream	530627	---	FLJ45974
rs1407513	6	168894852	0.0310	96.14%	1.000000	1.000000	0.01821000	0.03727000	NM_022138	downstream	84253	Hs.487200	SMOC2
rs156372	5	174888400	0.1592	93.68%	0.258100	0.452500	0.01822000	0.04927000	NM_022754	downstream	110	Hs.369440	SFXN1
rs10503665	8	19664614	0.1057	92.98%	0.187200	0.281700	0.01823000	0.05698000	NM_018142	upstream	54663	Hs.512627	INTS10
rs2354327	14	61809577	0.1057	92.98%	0.000000	0.001398	0.01823000	0.00783300	NM_001039785	downstream	138923	---	FLJ43390
rs9300689	13	101100765	0.2176	97.54%	0.295600	0.022620	0.01823000	0.02645000	NM_004791	intron	0	Hs.696554	ITGBL1
rs1114531	4	183281048	0.1222	94.74%	0.392700	0.377800	0.01824000	0.03952000	ENST00000315302	downstream	18753	---	---
rs1916309	9	14995594	0.2482	97.54%	0.872200	1.000000	0.01825000	0.01044000	NM_144966	upstream	136619	Hs.50850	FREM1
rs260970	1	39323829	0.1188	84.21%	0.117200	0.528700	0.01825000	0.00579700	NM_012090	intron	0	Hs.580782	MACF1

rs285046	13	97554998	0.0796	94.74%	0.678300	0.465400	0.01827000	0.14770000	NM_001001715	upstream	38437	Hs.403917	FARP1
rs10490434	2	16897839	0.1762	85.61%	0.024840	0.103700	0.01828000	0.05249000	NM_030797	upstream	187256	Hs.467769	FAM49A
rs10507384	13	27877480	0.2315	94.74%	0.231800	1.000000	0.01828000	0.06191000	NM_002019	intron	0	Hs.654360	FLT1
rs2454018	8	104182183	0.3255	97.54%	0.585000	0.202100	0.01829000	0.02995000	NM_001024372	upstream	39914	Hs.533446	BAALC
rs529618	11	115994188	0.1350	96.14%	0.191100	0.260300	0.01829000	0.02236000	NM_032725	downstream	129908	Hs.437341	BUD13
rs10496931	2	143033741	0.0798	92.28%	0.672700	0.677600	0.01830000	0.01473000	NM_001032998	upstream	317924	Hs.470126	KYNU
rs6054326	20	6444783	0.1610	51.23%	1.000000	1.000000	0.01830000	0.03156000	NM_001200	upstream	251962	Hs.73853	BMP2
rs10504472	8	71403032	0.1504	96.84%	0.476400	0.606200	0.01831000	0.01519000	NM_006540	intron	0	Hs.446678	NCOA2
rs2965710	12	11478437	0.4357	95.44%	0.540300	0.434600	0.01831000	0.03426000	NM_199354	upstream	38684	Hs.631726	PRB1
rs10505807	12	17419266	0.4705	95.09%	0.903000	0.445300	0.01833000	0.00709400	ENST00000386797	upstream	303560	---	---
rs634050	11	84433193	0.4453	86.67%	0.020660	0.137800	0.01833000	0.06481000	ENST00000376104	intron	0	---	---
rs10493228	1	57759841	0.2611	94.74%	0.344600	0.010650	0.01835000	0.02909000	NM_021080	intron	0	Hs.477370	DAB1
rs1947942	15	99686574	0.2814	97.89%	0.053190	0.015150	0.01835000	0.00315900	NM_138321	intron	0	Hs.498494	PCSK6
rs2166480	2	135353808	0.3840	92.28%	0.896700	0.754300	0.01836000	0.10280000	NM_138326	intron	0	Hs.655728	ACMSD
rs971120	2	76815026	0.3657	94.04%	0.357700	0.608300	0.01838000	0.04293000	NM_024993	downstream	14303	Hs.285782	LRRTM4
rs10486315	7	18883166	0.0305	97.89%	1.000000	1.000000	0.01839000	0.02803000	NM_178425	intron	0	Hs.196054	HDAC9
rs10496171	2	69868685	0.2574	94.74%	0.427200	1.000000	0.01839000	0.05842000	NM_001153	intron	0	Hs.422986	ANXA4
rs333856	2	117692216	0.0305	97.89%	1.000000	1.000000	0.01839000	0.18180000	NM_006773	upstream	596509	Hs.363492	DDX18
rs532827	5	39961142	0.4018	96.49%	0.380500	0.746200	0.01839000	0.01967000	NM_001343	upstream	500439	Hs.481980	DAB2
rs10499634	7	45442863	0.0578	94.04%	1.000000	1.000000	0.01840000	0.00936900	NM_021116	upstream	137805	Hs.192215	ADCY1
rs4468424	12	94218737	0.1007	97.54%	0.747400	0.734000	0.01840000	0.03331000	NM_017599	3UTR	0	Hs.24135	VEZT
rs10484056	14	98009509	0.0308	96.84%	1.000000	1.000000	0.01843000	0.00996100	NM_182560	upstream	238194	Hs.448754	C14orf177
rs10484996	6	97341865	0.3670	93.68%	0.599300	0.638100	0.01843000	0.15840000	NM_030784	downstream	10780	Hs.632612	GPR63
rs1876091	3	63566006	0.3239	92.63%	0.090860	0.139400	0.01843000	0.07019000	NM_144642	intron	0	Hs.648668	SYNPR
rs1987295	14	40452545	0.4981	94.74%	0.466700	0.652000	0.01847000	0.01015000	NM_152447	upstream	693978	Hs.136893	LRFN5
rs2055056	8	96347246	0.1370	94.74%	0.126100	0.127400	0.01848000	0.02974000	NM_177965	intron	0	Hs.548157	C8orf37
rs922522	13	94512496	0.0769	95.79%	0.013460	0.012760	0.01848000	0.09918000	NM_005845	intron	0	Hs.508423	ABCC4
rs9301679	13	89938730	0.2797	91.58%	0.091230	0.534900	0.01849000	0.06413000	ENST00000385123	downstream	257198	---	---
rs10509874	10	110332098	0.0698	90.53%	1.000000	1.000000	0.01850000	0.07212000	ENST00000388288	downstream	358723	---	---
rs2170177	3	51324927	0.2260	87.72%	0.717100	0.682500	0.01850000	0.10450000	NM_004947	intron	0	Hs.476284	DOCK3
rs10515586	5	146747481	0.1481	91.23%	1.000000	1.000000	0.01852000	0.05178000	NM_001387	downstream	3088	Hs.519659	DPYSL3
rs10510017	10	118109971	0.1635	93.33%	1.000000	0.628200	0.01853000	0.00472800	NM_198515	intron	0	Hs.233407	C10orf96
rs1510063	5	83732185	0.4299	95.09%	0.217600	0.285900	0.01853000	0.07075000	NM_005711	upstream	15818	Hs.482730	EDIL3
rs10518875	15	34446568	0.4833	94.39%	0.903000	0.445400	0.01854000	0.03433000	NM_080650	upstream	820912	Hs.107196	ATPBD4
rs4374314	2	143033630	0.0761	96.84%	0.661400	0.664100	0.01854000	0.01219000	NM_001032998	upstream	318035	Hs.470126	KYNU
rs10519933	4	149135887	0.0808	93.33%	0.682100	1.000000	0.01855000	0.05485000	NM_024605	intron	0	Hs.368631	ARHGAP10
rs1525169	2	149752898	0.1004	97.89%	0.089290	0.079910	0.01855000	0.06562000	NM_177964	intron	0	Hs.357567	LOC130576
rs10509393	10	79596991	0.1434	92.98%	0.450600	0.283800	0.01859000	0.04325000	NM_001026	downstream	126514	Hs.356794	RPS24
rs6878331	5	38617821	0.0198	88.77%	1.000000	1.000000	0.01859000	0.99780000	NM_002310	intron	0	Hs.133421	LIFR
rs10491442	5	58925052	0.1648	92.63%	0.657700	0.524400	0.01860000	0.10170000	ENST00000340635	intron	0	---	---
rs2867321	19	57117898	0.1399	94.04%	1.000000	1.000000	0.01861000	0.02142000	NM_024840	upstream	4643	Hs.183390	ZNF613
rs10513444	3	154487391	0.0492	92.63%	0.476400	1.000000	0.01863000	0.04312000	ENST00000383956	upstream	86895	---	---
rs1390762	16	49452675	0.2724	94.04%	0.442100	0.679700	0.01863000	0.03457000	NM_001042412	downstream	62646	Hs.578973	CYLD
rs2354340	7	138289024	0.0685	94.74%	0.355200	0.625100	0.01864000	0.04715000	NM_080660	downstream	71968	Hs.659143	ZC3HAV1L
rs1566313	12	114343491	0.4880	87.37%	0.003447	0.002592	0.01865000	0.15710000	ENST00000386460	upstream	296416	---	---
rs769657	4	141097109	0.1505	97.89%	0.354200	1.000000	0.01868000	0.05719000	NM_018717	upstream	65529	---	MAML3
rs10496582	2	122835251	0.3713	94.04%	0.359800	0.238800	0.01870000	0.12650000	NM_004622	downstream	595838	Hs.75066	TSN
rs1399141	13	60238751	0.4554	94.39%	0.085010	0.093860	0.01871000	0.07341000	ENST00000384127	upstream	22944	---	---
rs1539804	18	5949258	0.2491	97.89%	0.148800	0.041260	0.01871000	0.08412000	ENST00000317931	intron	0	---	---
rs2025503	6	23701746	0.2500	97.54%	0.338300	0.380700	0.01871000	0.07768000	ENST00000330282	upstream	261115	---	---
rs2328411	20	19495213	0.2500	97.54%	0.423600	0.119700	0.01871000	0.02141000	NM_020689	intron	0	Hs.654790	SLC24A3
rs266175	2	107417762	0.1918	97.89%	0.846400	1.000000	0.01875000	0.03723000	NM_032528	upstream	548720	Hs.98265	ST6GAL2
rs266177	2	107417947	0.1918	97.89%	0.846400	1.000000	0.01875000	0.03723000	NM_032528	upstream	548905	Hs.98265	ST6GAL2
rs10504934	8	95274323	0.3382	96.49%	0.139000	0.284000	0.01876000	0.00843200	NM_004063	intron	0	Hs.591853	CDH17

rs2333537	14	28645046	0.1018	96.49%	0.750000	0.735200	0.01876000	0.04215000	ENST00000386746	upstream	123679	---	---
rs2645220	10	4061978	0.1115	91.23%	0.220000	0.318500	0.01876000	0.09853000	NM_001300	upstream	244511	Hs.4055	KLF6
rs10506230	12	42320036	0.3939	97.54%	0.900000	0.743600	0.01877000	0.04998000	NM_175851	upstream	88045	---	ADAMTS20
rs597941	1	57530112	0.4622	88.07%	0.611300	0.215200	0.01877000	0.08894000	NM_021080	intron	0	Hs.477370	DAB1
rs2548003	5	28783080	0.4228	95.44%	1.000000	0.879000	0.01879000	0.11130000	ENST00000387488	downstream	122451	---	---
rs1106185	2	10881021	0.4834	95.09%	0.273800	0.651000	0.01881000	0.00249900	NM_005742	upstream	10600	Hs.212102	PDIA6
rs4978512	9	114700189	0.2539	90.53%	0.322900	1.000000	0.01884000	0.03117000	NM_033051	upstream	7175	Hs.512668	SLC46A2
rs10500387	16	12622680	0.1236	95.09%	0.094240	0.382100	0.01886000	0.05191000	NM_018340	downstream	41758	Hs.460002	FLJ11151
rs487707	11	113955918	0.2917	96.84%	0.382000	0.864900	0.01886000	0.02198000	NM_017678	intron	0	Hs.179100	FAM55D
rs720605	14	55957690	0.2941	95.44%	0.190500	0.301200	0.01886000	0.00514900	NM_021255	downstream	119917	Hs.657926	PELI2
rs7018877	9	16600703	0.0519	91.23%	1.000000	1.000000	0.01889000	0.01939000	NM_017637	intron	0	Hs.656581	BNC2
rs2843070	6	63510470	0.4063	84.21%	0.015660	0.014990	0.01890000	0.02364000	ENST00000384941	upstream	59338	---	---
rs4145660	5	83611438	0.3989	97.19%	0.380100	0.879600	0.01893000	0.00362500	NM_005711	intron	0	Hs.482730	EDIL3
rs1961415	13	21382460	0.0957	80.70%	0.000002	0.000012	0.01894000	0.05706000	ENST00000386670	downstream	119476	---	---
rs6849746	4	112063535	0.0793	95.09%	1.000000	1.000000	0.01894000	0.08725000	NM_000325	upstream	299832	Hs.643588	PITX2
rs646983	13	29413553	0.2860	90.18%	0.444800	0.078160	0.01895000	0.06425000	NM_001010918	intron	0	---	LOC440131
rs1048829	2	203138701	0.3717	94.39%	0.604000	0.614300	0.01896000	0.07742000	NM_001204	3UTR	0	Hs.471119	BMPR2
rs10489061	4	15958047	0.0921	93.33%	0.254500	0.240100	0.01898000	0.01435000	NM_152684	downstream	118664	---	FLJ39653
rs1399664	18	25073597	0.0246	78.60%	1.000000	1.000000	0.01898000	0.99770000	ENST00000384802	downstream	43107	---	---
rs3754743	2	173710338	0.1715	97.19%	0.134800	0.163500	0.01900000	0.16670000	NM_133646	intron	0	Hs.444451	ZAK
rs10489832	1	156720980	0.1328	89.82%	0.095940	0.405700	0.01901000	0.07907000	NM_001004472	downstream	3681	Hs.704755	OR10R2
rs1105047	2	202911203	0.2416	93.68%	0.406300	0.257900	0.01901000	0.05059000	ENST00000385404	downstream	4507	---	---
rs10489069	4	9887766	0.1447	93.33%	0.217900	0.291900	0.01904000	0.01695000	NM_005112	upstream	160095	Hs.128548	WDR1
rs2327597	20	11909243	0.1241	94.74%	1.000000	0.225200	0.01904000	0.07029000	NM_181443	downstream	54000	Hs.702048	BTBD3
rs10518147	4	77541071	0.1757	96.84%	0.021650	0.378600	0.01905000	0.00187500	NM_178555	intron	0	---	FLJ25770
rs2355145	7	11898092	0.4570	89.82%	0.614500	1.000000	0.01905000	0.01979000	NM_018374	upstream	322861	Hs.396358	TMEM106B
rs2828759	21	24340799	0.3388	96.84%	0.178300	0.021050	0.01907000	0.12300000	ENST00000355181	upstream	442342	---	---
rs9554928	13	102468201	0.3708	95.09%	0.009162	0.011030	0.01907000	0.02302000	NM_000452	downstream	26150	Hs.194783	SLC10A2
rs970761	7	66763047	0.0878	97.89%	0.145500	0.232800	0.01908000	0.00352700	ENST00000388509	upstream	260994	---	---
rs1718582	7	68301997	0.3224	85.96%	0.466000	0.610700	0.01911000	0.05358000	ENST00000363319	downstream	73698	---	---
rs2593686	11	22346871	0.2194	97.54%	0.599000	1.000000	0.01911000	0.04583000	NM_020346	intron	0	Hs.242821	SLC17A6
rs6794333	3	133643347	0.1868	92.98%	0.690400	1.000000	0.01912000	0.13770000	NM_015268	intron	0	Hs.12707	DNAJC13
rs10507821	13	73492535	0.1889	76.14%	1.000000	0.528100	0.01915000	0.01927000	NM_007249	intron	0	Hs.373857	KLF12
rs181130	2	8190471	0.3315	97.89%	0.279200	0.719400	0.01918000	0.00494700	NM_207358	downstream	154448	---	C2orf46
rs617739	6	165816934	0.2316	85.61%	1.000000	1.000000	0.01918000	0.07601000	NM_006661	intron	0	Hs.584856	PDE10A
rs10501721	11	89921579	0.2361	94.39%	0.866000	1.000000	0.01921000	0.01627000	NM_012124	upstream	325752	Hs.22857	CHORDC1
rs1021428	4	11484791	0.0769	95.79%	0.663900	0.664100	0.01925000	0.02493000	NM_005114	upstream	444304	Hs.507348	HS3ST1
rs10520241	2	79525360	0.0206	93.68%	1.000000	1.000000	0.01925000	0.99770000	NM_138937	upstream	284973	Hs.567312	REG3A
rs953896	6	54639908	0.4070	90.53%	0.700100	0.637400	0.01925000	0.01065000	NM_014464	downstream	277038	Hs.127011	TINAG
rs10484717	6	128221953	0.0156	90.18%	1.000000	1.000000	0.01926000	0.02811000	NM_001010923	intron	0	Hs.661756	C6orf190
rs10516456	4	100878532	0.1090	93.33%	0.022810	0.360500	0.01926000	0.10540000	NM_014395	upstream	78481	Hs.436271	DAPP1
rs9286854	1	170633429	0.4101	97.54%	0.001924	0.000496	0.01927000	0.17120000	NM_015569	intron	0	Hs.654775	DNM3
rs10503576	8	16179617	0.1139	90.88%	0.218500	0.366300	0.01933000	0.00946600	NM_138716	upstream	85022	Hs.147635	MSR1
rs4404787	6	129422719	0.0205	94.04%	1.000000	1.000000	0.01934000	0.99800000	NM_000426	CDS	0	Hs.200841	LAMA2
rs10502797	18	38584413	0.0203	95.09%	1.000000	1.000000	0.01935000	0.99760000	NM_002930	intron	0	Hs.464985	RIT2
rs2828309	21	23887126	0.0203	95.09%	1.000000	1.000000	0.01935000	0.99760000	ENST00000362828	upstream	160653	---	---
rs10501558	11	83709460	0.1880	93.33%	0.070190	0.017590	0.01936000	0.02881000	ENST00000376104	intron	0	---	---
rs976436	12	24985125	0.1380	87.72%	0.281100	0.428900	0.01937000	0.12000000	NM_005504	upstream	39033	Hs.438993	BCAT1
rs10512035	9	1913359	0.0899	97.54%	0.709100	0.701900	0.01938000	0.01317000	NM_139045	upstream	91983	Hs.298990	SMARCA2
rs10489833	1	156757075	0.4777	94.39%	0.328700	0.759300	0.01940000	0.19230000	NM_001005189	downstream	26467	Hs.553780	OR6Y1
rs10498019	2	215197842	0.0204	94.74%	1.000000	1.000000	0.01940000	0.99770000	ENST00000312710	upstream	72862	---	---
rs2369705	4	5487491	0.1563	78.60%	0.001968	0.054410	0.01940000	0.03855000	NM_018401	intron	0	Hs.133062	STK32B
rs983459	12	16543428	0.0771	97.89%	1.000000	0.429700	0.01940000	0.00412000	NM_001001395	downstream	49146	Hs.504908	LMO3
rs10507254	12	113821356	0.3024	73.68%	0.070820	0.127800	0.01941000	0.03479000	ENST00000386518	downstream	161353	---	---

rs4774383	15	59105220	0.3277	92.63%	0.017390	0.020440	0.01943000	0.04402000	NM_134261	intron	0	Hs.695914	RORA
rs2345963	1	162008708	0.1020	85.96%	0.724400	1.000000	0.01946000	0.06748000	ENST00000385701	upstream	150823	---	---
rs958485	15	42137357	0.0897	95.79%	0.462300	1.000000	0.01946000	0.05373000	NM_032892	intron	0	Hs.578544	FRMD5
rs10501724	11	89922809	0.2362	95.09%	0.866200	1.000000	0.01952000	0.01441000	NM_012124	upstream	326982	Hs.22857	CHORDC1
rs4474302	1	56040625	0.4535	94.39%	0.109700	0.018450	0.01952000	0.03612000	NM_207465	downstream	70245	---	---
rs4131134	4	47349266	0.0896	97.89%	0.470300	0.562100	0.01953000	0.02144000	NM_006587	intron	0	Hs.518618	CORIN
rs26207	5	129063995	0.3346	91.23%	0.406600	0.357500	0.01954000	0.01642000	NM_133638	intron	0	Hs.23751	ADAMTS19
rs3751420	13	18600924	0.3399	97.54%	0.184300	0.595000	0.01955000	0.04006000	ENST00000343741	downstream	8635	---	---
rs10519706	5	121884405	0.0954	91.93%	0.714700	1.000000	0.01958000	0.02770000	NM_005460	downstream	56712	Hs.426463	SNCAIP
rs950230	6	44421400	0.2610	95.44%	0.273100	0.716300	0.01958000	0.05721000	NM_145026	intron	0	Hs.135283	SPATS1
rs10486788	7	15981866	0.3778	94.74%	0.093610	0.093640	0.01960000	0.01384000	ENST00000366332	downstream	113355	---	---
rs10502380	18	8787022	0.0843	91.58%	0.405800	0.404100	0.01960000	0.00760500	NM_015210	intron	0	Hs.650822	KIAA0802
rs10491340	5	147370294	0.0616	76.84%	0.000000	0.000563	0.01962000	0.12470000	NM_006846	upstream	53507	Hs.331555	SPINK5
rs530055	12	68258648	0.1916	91.58%	0.550700	1.000000	0.01964000	0.04148000	NM_001042555	downstream	3637	Hs.593446	FRS2
rs10483095	22	16511431	0.0238	81.05%	0.114700	0.165800	0.01966000	0.99760000	NM_015367	intron	0	Hs.699302	BCL2L13
rs10517249	4	32017986	0.2581	97.19%	0.875500	0.830100	0.01966000	0.00538100	NM_032457	downstream	1264417	Hs.570785	PCDH7
rs10510536	3	23244774	0.0955	93.68%	1.000000	1.000000	0.01967000	0.00157600	NM_152653	intron	0	Hs.475688	UBE2E2
rs1460465	13	71361542	0.0776	97.19%	1.000000	0.433500	0.01967000	0.12210000	NM_004392	upstream	22211	Hs.129452	DACH1
rs2253977	20	3887140	0.3132	92.98%	0.668500	1.000000	0.01968000	0.08555000	NM_007219	intron	0	Hs.589884	RNF24
rs2527165	8	5521071	0.4493	76.14%	0.013360	0.241100	0.01968000	0.08441000	ENST00000363751	downstream	234775	---	---
rs901558	11	11381343	0.1191	97.19%	0.246600	0.368900	0.01968000	0.05971000	NM_198516	intron	0	Hs.655152	GALNTL4
rs2288760	2	157135568	0.3546	88.07%	0.000856	0.043890	0.01971000	0.02770000	NM_000408	intron	0	Hs.512382	GPD2
rs10514631	2	19767453	0.1679	91.93%	0.266600	0.469300	0.01972000	0.05247000	NM_145260	upstream	345558	Hs.123933	OSR1
rs6735064	2	14372951	0.1611	94.74%	0.499600	1.000000	0.01972000	0.02585000	NM_145175	upstream	317310	Hs.260855	FAM84A
rs9284975	5	167313056	0.1481	91.23%	0.466900	1.000000	0.01972000	0.02324000	ENST00000388904	intron	0	---	---
rs10496244	2	80940121	0.4464	88.42%	0.310000	0.258000	0.01973000	0.01320000	NM_004389	downstream	211613	Hs.167368	CTNNA2
rs2387174	5	9882373	0.1399	94.04%	1.000000	1.000000	0.01973000	0.07651000	ENST00000362925	downstream	68255	---	---
rs6855439	4	37535285	0.1397	86.67%	0.032980	0.158200	0.01973000	0.08299000	NM_018290	intron	0	Hs.23363	PGM2
rs9294087	6	78581437	0.1777	95.79%	1.000000	0.545800	0.01973000	0.00619500	NM_001010844	upstream	1052471	Hs.656212	IRAK1BP1
rs2215232	4	11203210	0.4623	88.42%	0.447500	0.748600	0.01975000	0.06422000	NM_005114	upstream	162723	Hs.507348	HS3ST1
rs10489575	1	231300770	0.0830	92.98%	0.696000	1.000000	0.01977000	0.04762000	ENST00000366625	intron	0	---	---
rs1171576	1	154780180	0.2161	82.81%	0.443600	1.000000	0.01979000	0.07402000	NM_178229	intron	0	Hs.591495	IQGAP3
rs398	5	9667053	0.0406	95.09%	1.000000	1.000000	0.01979000	0.04435000	NM_019599	downstream	15062	Hs.567492	TAS2R1
rs10493013	1	22575622	0.1593	87.02%	1.000000	0.311700	0.01981000	0.03500000	NM_014870	upstream	75315	Hs.418966	ZBTB40
rs10494963	1	212029177	0.0111	95.09%	1.000000	1.000000	0.01982000	0.05198000	ENST00000362373	downstream	152499	---	---
rs10509693	10	97135219	0.0111	95.09%	1.000000	1.000000	0.01982000	0.04311000	NM_001034957	intron	0	Hs.696027	SORBS1
rs10518612	1	80209336	0.0111	95.09%	0.027570	1.000000	0.01982000	0.99820000	ENST00000294631	upstream	964386	---	---
rs7992455	13	45309508	0.0111	95.09%	1.000000	1.000000	0.01982000	0.03745000	NM_015070	downstream	124807	Hs.136102	ZC3H13
rs10501919	11	98987681	0.2111	94.74%	0.466200	1.000000	0.01983000	0.00561600	NM_175566	upstream	207792	Hs.656783	CNTN5
rs10493605	1	77642912	0.1191	97.19%	0.562700	0.366400	0.01985000	0.03029000	NM_012093	intron	0	Hs.559718	AK5
rs388491	6	94994319	0.4239	96.84%	0.391000	0.269900	0.01985000	0.07566000	ENST00000386414	downstream	219243	---	---
rs632480	9	102839173	0.3585	95.44%	0.792500	0.634600	0.01988000	0.00457900	NM_207299	intron	0	Hs.382683	RP11-35N6
rs2141531	7	9219938	0.2318	96.14%	0.401300	0.442200	0.01989000	0.01703000	NM_152745	downstream	462014	Hs.487564	NXPH1
rs10514979	5	64643370	0.0812	95.09%	0.398100	0.692700	0.01991000	0.07701000	NM_197941	intron	0	Hs.482291	ADAMTS6
rs202116	5	64101033	0.2012	89.82%	0.845500	1.000000	0.01991000	0.02097000	NM_005869	intron	0	Hs.371372	SDCCAG10
rs6732490	2	67714314	0.2824	91.93%	0.446900	0.594400	0.01991000	0.01230000	NM_019002	downstream	223136	Hs.353022	ETAA1
rs7216226	17	10875893	0.0812	95.09%	0.398100	0.393400	0.01991000	0.05218000	ENST00000386899	upstream	125604	---	---
rs1790821	18	70079851	0.1979	82.46%	0.003164	0.016230	0.01992000	0.20590000	NM_001914	intron	0	Hs.465413	CYB5A
rs2823538	21	16293721	0.2591	96.84%	0.639200	0.669000	0.01992000	0.13680000	NM_013396	downstream	119473	Hs.473370	USP25
rs2929660	15	36852716	0.2256	97.19%	0.863400	0.693200	0.01992000	0.10410000	NM_207444	downstream	73185	Hs.448785	C15orf53
rs10486800	7	39619950	0.0556	97.89%	0.042600	0.050850	0.01995000	0.06296000	NM_005402	upstream	9737	Hs.6906	RALA
rs1586681	9	86837254	0.0556	97.89%	0.586000	1.000000	0.01995000	0.10050000	NM_001018066	downstream	154621	Hs.494312	NTRK2
rs2032202	21	20598799	0.4346	91.23%	0.209800	0.112600	0.01995000	0.02227000	ENST00000387009	downstream	51132	---	---
rs704407	3	64220305	0.2240	97.89%	0.086170	0.014550	0.01995000	0.01374000	NM_198859	upstream	34134	Hs.699317	PRICKLE2

rs10488674	11	2956971	0.1863	95.09%	0.315300	0.392500	0.01996000	0.12410000	NM_005969	intron	0	Hs.501684	NAP1L4
rs10494032	1	106012636	0.4291	96.49%	0.713800	0.874300	0.01996000	0.12840000	ENST00000388703	downstream	1135093	---	---
rs1321465	20	52465568	0.1863	95.09%	0.840900	0.775900	0.01996000	0.09386000	NM_018431	upstream	60020	Hs.656582	DOK5
rs1375272	4	190034255	0.1863	95.09%	0.840900	0.775900	0.01996000	0.05088000	ENST00000321235	downstream	136803	---	---
rs1607452	2	4250757	0.0469	97.19%	0.015830	0.007505	0.01996000	0.02724000	ENST00000382050	downstream	521256	---	---
rs1321601	6	153630201	0.1630	94.74%	0.187000	1.000000	0.01997000	0.03987000	ENST00000312401	downstream	14914	---	---
rs7074141	10	67452748	0.1630	94.74%	0.377500	1.000000	0.01997000	0.00693600	NM_013266	intron	0	Hs.660362	CTNNA3
rs10518475	4	126770936	0.3540	96.14%	0.792200	0.387400	0.01999000	0.00306100	NM_024582	downstream	138563	Hs.702217	FAT4
rs740676	16	7652417	0.4314	89.47%	0.055890	0.271700	0.02001000	0.01397000	NM_018723	intron	0	Hs.459842	A2BP1
rs10504782	8	84319188	0.1264	94.39%	0.401300	1.000000	0.02002000	0.00688300	NM_173848	upstream	1284924	Hs.121663	RALYL
rs1387774	3	177892983	0.2647	89.47%	0.422600	0.515800	0.02002000	0.17530000	ENST00000359552	downstream	33357	---	---
rs10484322	6	6210193	0.1241	94.74%	1.000000	1.000000	0.02003000	0.01523000	NM_000129	intron	0	Hs.335513	F13A1
rs6580266	5	142415941	0.4564	96.49%	0.903300	0.651900	0.02003000	0.01022000	NM_015071	intron	0	Hs.654668	ARHGAP26
rs1039116	15	84618201	0.3007	96.84%	0.775200	0.847700	0.02005000	0.05215000	NM_152336	intron	0	Hs.679833	AGBL1
rs1191599	14	29173192	0.0523	90.53%	0.512000	1.000000	0.02005000	0.01105000	NM_002742	intron	0	Hs.508999	PRKD1
rs10496131	2	66156377	0.0951	94.04%	0.275200	0.259900	0.02006000	0.06798000	NM_002398	upstream	359659	Hs.526754	MEIS1
rs1375872	3	36214676	0.2996	97.19%	0.390400	0.614400	0.02007000	0.04964000	NM_003149	upstream	182425	Hs.56045	STAC
rs9299440	10	61740701	0.3429	91.58%	0.075280	0.328300	0.02008000	0.01217000	NM_020987	intron	0	Hs.499725	ANK3
rs10509743	10	102180396	0.1763	97.54%	0.678800	0.357300	0.02010000	0.15370000	NM_003393	upstream	32392	Hs.421281	WNT8B
rs10511605	9	15011481	0.2172	93.68%	0.470700	0.452500	0.02010000	0.00500500	NM_152574	downstream	150080	Hs.563630	C9orf52
rs1363148	5	128910813	0.3453	97.54%	1.000000	0.165400	0.02010000	0.01447000	NM_133638	intron	0	Hs.23751	ADAMTS19
rs10501725	11	89966065	0.2291	96.49%	0.864500	0.635000	0.02011000	0.02878000	NM_012124	upstream	370238	Hs.22857	CHORDC1
rs4944267	11	80255476	0.2968	97.54%	0.249900	0.498300	0.02011000	0.05388000	ENST00000363276	downstream	338718	---	---
rs10509812	10	107944383	0.1981	91.23%	1.000000	0.663700	0.02012000	0.00817300	NM_001013031	downstream	382586	Hs.591915	SORCS1
rs2715694	3	110023833	0.1788	96.14%	0.213900	0.494500	0.02012000	0.07949000	NM_016388	upstream	488	Hs.138701	TRAT1
rs673702	1	49342221	0.3488	90.53%	0.493700	0.857800	0.02012000	0.07772000	ENST00000334103	intron	0	---	---
rs10506337	12	53692096	0.1605	95.09%	0.366400	0.742200	0.02013000	0.07131000	NM_021191	upstream	7793	Hs.591024	NEUROD4
rs1271582	14	64634456	0.2481	94.04%	0.100100	0.092800	0.02013000	0.00369100	NM_197957	intron	0	Hs.285354	MAX
rs290641	15	93485231	0.4961	90.88%	0.619000	0.540900	0.02013000	0.23880000	ENST00000313753	downstream	285240	---	---
rs2206333	6	23627693	0.2590	97.54%	0.876800	0.400000	0.02014000	0.04627000	ENST00000330282	upstream	335168	---	---
rs10502429	18	18759278	0.0941	95.09%	0.271800	0.256700	0.02015000	0.01773000	NM_203292	upstream	8015	Hs.546282	RBBP8
rs2049638	2	34808299	0.3278	94.74%	0.168700	0.256600	0.02015000	0.08101000	ENST00000384525	upstream	741876	---	---
rs10493169	1	52876626	0.0111	94.74%	1.000000	1.000000	0.02016000	0.27150000	ENST00000343491	intron	0	---	---
rs10508631	10	20952980	0.0872	90.53%	0.031100	0.030560	0.02016000	0.05170000	NM_213569	downstream	159861	Hs.5025	NEBL
rs10513820	3	189015786	0.0111	94.74%	1.000000	1.000000	0.02016000	0.99840000	NM_138931	upstream	69617	Hs.478588	BCL6
rs1980078	3	125974349	0.3016	90.18%	0.001860	0.002991	0.02017000	0.02450000	NM_002213	intron	0	Hs.536663	ITGB5
rs514778	6	12701446	0.2059	89.47%	0.341700	0.788700	0.02017000	0.01633000	NM_030948	upstream	125517	Hs.436996	PHACTR1
rs2024265	7	156450380	0.1692	93.33%	0.515400	0.631300	0.02018000	0.02342000	NM_138400	intron	0	Hs.15825	NOM1
rs10485447	20	51423786	0.2435	95.09%	0.325200	0.851600	0.02020000	0.04520000	NM_173485	intron	0	Hs.473117	TSHZ2
rs10519231	15	77273165	0.2199	93.33%	0.594000	0.804000	0.02023000	0.26700000	NM_153815	upstream	103004	Hs.591111	RASGRF1
rs1595902	18	60646565	0.2119	94.39%	0.147000	0.307000	0.02025000	0.02276000	ENST00000363325	upstream	278842	---	---
rs2466030	8	128276320	0.2140	95.09%	0.587800	1.000000	0.02027000	0.01393000	ENST00000377972	downstream	435390	---	---
rs10497720	2	192622531	0.2444	93.33%	0.739800	0.848600	0.02029000	0.02141000	NM_016192	intron	0	Hs.144513	TMEFF2
rs839022	4	57986522	0.4635	96.14%	0.395700	0.643900	0.02029000	0.34480000	NM_001553	upstream	315214	Hs.479808	IGFBP7
rs10513246	3	145946023	0.0201	95.79%	1.000000	1.000000	0.02030000	0.12270000	ENST00000355888	downstream	298647	---	---
rs10511284	3	111511355	0.1925	83.86%	0.000313	0.001984	0.02032000	0.08928000	ENST00000388702	downstream	242053	---	---
rs1564870	3	68070620	0.2434	93.68%	0.045580	0.450500	0.02035000	0.33680000	NM_213609	upstream	67840	Hs.655061	FAM19A1
rs198617	13	47817359	0.0409	77.19%	0.303900	1.000000	0.02035000	0.07060000	NM_000321	intron	0	Hs.408528	RB1
rs2469583	15	46146164	0.3956	87.37%	1.000000	0.524400	0.02035000	0.01260000	NM_205850	upstream	54297	Hs.699810	SLC24A5
rs7853963	9	82908731	0.1461	93.68%	1.000000	0.421600	0.02035000	0.01314000	NM_005077	downstream	479687	Hs.197320	TLE1
rs10493475	1	70809473	0.0704	94.74%	0.005504	0.365500	0.02036000	0.01442000	NM_198719	downstream	399579	Hs.445000	PTGER3
rs10516164	4	4547912	0.1201	97.89%	1.000000	0.660000	0.02037000	0.03053000	NM_016930	intron	0	Hs.584913	STX18
rs2029395	2	179388466	0.3889	94.74%	0.010320	0.191500	0.02037000	0.03418000	NM_133379	upstream	8072	Hs.654592	TTN
rs1423297	5	28803744	0.4365	91.23%	0.706600	0.760800	0.02039000	0.08844000	ENST00000387488	downstream	143115	---	---

rs6427242	1	168554781	0.3222	94.74%	0.095860	1.000000	0.02040000	0.03217000	NM_152281	upstream	213113	Hs.183702	SCYL1BP1
rs10520958	5	30200233	0.2047	96.84%	0.098630	0.793500	0.02042000	0.04719000	ENST00000365278	downstream	215217	---	---
rs1458869	2	71364521	0.0846	93.33%	0.232600	1.000000	0.02042000	0.02736000	NM_001014972	upstream	47876	Hs.434401	ZNF638
rs10516049	5	168097247	0.0620	90.53%	0.248800	0.284200	0.02045000	0.08547000	NM_003062	intron	0	Hs.604116	SLIT3
rs10261711	7	9223227	0.0207	93.33%	0.100100	1.000000	0.02048000	0.02157000	NM_152745	downstream	465303	Hs.487564	NXPH1
rs10484093	14	85767631	0.2284	97.54%	0.234800	0.340700	0.02048000	0.38050000	NM_013231	downstream	603609	Hs.533710	FLRT2
rs2629812	1	114454559	0.4004	86.32%	0.000892	0.010310	0.02048000	0.27050000	NM_205848	intron	0	Hs.370963	SYT6
rs10521232	17	13480529	0.2122	95.09%	0.719300	0.837900	0.02049000	0.06911000	NM_006042	upstream	34560	Hs.462270	HS3ST3A1
rs359421	5	173258260	0.4615	95.79%	0.225900	0.069140	0.02049000	0.00452500	NM_030627	intron	0	Hs.127126	CPEB4
rs10494638	1	188850238	0.1218	96.49%	1.000000	0.374800	0.02053000	0.01777000	NM_199051	upstream	136856	Hs.65765	FAM5C
rs10500696	11	7332490	0.0967	94.39%	0.722800	0.233500	0.02053000	0.02451000	NM_175733	intron	0	Hs.177193	SYT9
rs1443402	5	17957829	0.4247	97.89%	0.327900	0.156200	0.02056000	0.02248000	ENST00000332101	upstream	269433	---	---
rs1462935	1	60379766	0.1712	90.18%	0.378000	0.327500	0.02056000	0.05472000	NM_152377	upstream	67736	Hs.47385	C1orf87
rs516224	4	74969011	0.4247	97.89%	0.713400	0.347200	0.02056000	0.03600000	NM_001511	downstream	762	Hs.789	CXCL1
rs1895370	16	25202852	0.2656	95.79%	0.538000	0.717400	0.02057000	0.02427000	NM_001012981	upstream	26509	Hs.513451	ZKSCAN2
rs368248	2	40340545	0.2816	85.96%	0.636200	0.529800	0.02057000	0.06873000	NM_021097	intron	0	Hs.468274	SLC8A1
rs8123670	20	22408864	0.1283	94.39%	0.783300	0.565800	0.02058000	0.03575000	NM_153675	downstream	100959	Hs.155651	FOXA2
rs11477	5	138775359	0.3014	97.19%	0.257200	0.128600	0.02059000	0.11860000	NM_152686	3UTR	0	Hs.483537	DNAJC18
rs2488264	10	29609361	0.2353	95.44%	0.502200	0.846700	0.02059000	0.04248000	NM_032517	upstream	8635	Hs.558572	LYZL1
rs2281236	16	1543577	0.4583	96.84%	0.628800	0.441200	0.02061000	0.12270000	NM_024600	intron	0	Hs.459652	TMEM204
rs4977824	9	23111911	0.1566	87.37%	0.087730	0.067650	0.02062000	0.09871000	NM_004432	downstream	570073	Hs.166109	ELAVL2
rs899361	13	80698342	0.3735	88.77%	0.031630	0.015910	0.02062000	0.00294000	ENST00000387380	upstream	39541	---	---
rs1910651	21	24343223	0.3277	93.68%	0.094720	0.028530	0.02063000	0.14630000	ENST00000355181	upstream	439918	---	---
rs1371422	2	35626263	0.3569	96.84%	0.358100	0.269700	0.02064000	0.02243000	NM_016441	upstream	810310	Hs.699247	CRIM1
rs381627	2	40341098	0.2463	94.04%	0.137200	1.000000	0.02064000	0.06458000	NM_021097	intron	0	Hs.468274	SLC8A1
rs805935	20	37690703	0.3441	92.28%	0.132100	1.000000	0.02064000	0.12350000	NM_005461	downstream	1057199	Hs.702085	MAFB
rs1914154	10	37347820	0.4982	96.84%	0.400900	0.131100	0.02068000	0.05090000	NM_052997	upstream	106971	Hs.373787	ANKRD30A
rs10509692	10	97226588	0.4045	93.68%	1.000000	0.745200	0.02069000	0.03346000	NM_001034957	intron	0	Hs.696027	SORBS1
rs2388956	1	80256594	0.0197	97.89%	0.095580	1.000000	0.02070000	0.10310000	ENST00000370739	upstream	1011644	---	---
rs4438374	18	34747509	0.0696	80.70%	0.082090	0.277100	0.02071000	0.03829000	NM_020180	upstream	1347568	Hs.435976	BRUNOL4
rs641117	7	51792039	0.1496	92.63%	0.811100	0.720700	0.02072000	0.00448000	ENST00000388001	downstream	39919	---	---
rs1430024	2	37967500	0.1673	94.39%	0.185700	0.205800	0.02073000	0.02363000	NM_144713	upstream	63610	Hs.591566	FAM82A
rs2710299	12	11960247	0.1538	91.23%	0.228500	0.797700	0.02074000	0.04821000	NM_001987	downstream	20659	Hs.504765	ETV6
rs9301833	13	92178407	0.1588	96.14%	0.363200	0.619700	0.02074000	0.00928800	NM_004466	intron	0	Hs.655675	GPC5
rs10484689	6	147967443	0.4301	97.89%	0.020940	0.134300	0.02076000	0.10440000	NM_001030060	downstream	34593	Hs.567973	SAMD5
rs10509054	10	58085702	0.0578	97.19%	0.223300	0.256100	0.02076000	0.06137000	NM_001005413	upstream	294669	Hs.591363	ZWINT
rs10502755	18	37055020	0.0881	91.58%	1.000000	1.000000	0.02077000	0.04951000	NM_002647	upstream	734177	Hs.464971	PIK3C3
rs2157998	7	91158790	0.2780	78.25%	0.616200	0.364400	0.02080000	0.02296000	NM_006980	downstream	181167	Hs.532216	MTERF
rs2367901	3	63566968	0.3303	95.09%	0.218700	0.331400	0.02080000	0.07047000	NM_144642	intron	0	Hs.648668	SYNPR
rs7669414	4	36230220	0.1259	94.74%	0.159200	0.235200	0.02080000	0.03654000	ENST00000357504	downstream	208409	---	---
rs6461667	7	22763268	0.2032	97.54%	0.578800	0.793000	0.02081000	0.17170000	ENST00000387559	downstream	8255	---	---
rs10489042	4	17490583	0.0208	92.63%	1.000000	1.000000	0.02083000	0.01752000	NM_153686	intron	0	Hs.446201	LCORL
rs304073	3	4511386	0.4673	91.23%	0.618300	0.875800	0.02083000	0.12890000	NM_002222	intron	0	Hs.699169	ITPR1
rs1502702	9	90639864	0.1380	97.89%	1.000000	0.462800	0.02084000	0.02676000	NM_001001938	upstream	155799	Hs.585118	C9orf47
rs1463074	11	41584071	0.3040	97.54%	1.000000	1.000000	0.02085000	0.01557000	NM_006595	upstream	1706038	Hs.435771	API5
rs4703156	5	100935754	0.4506	88.77%	0.703600	1.000000	0.02087000	0.00140600	ENST00000388691	upstream	9679	---	---
rs2640504	1	110975409	0.0375	56.14%	0.013830	0.028110	0.02089000	0.99770000	ENST00000369770	intron	0	---	---
rs949731	3	7327379	0.2807	85.61%	0.010810	0.028910	0.02091000	0.01190000	NM_181875	intron	0	---	GRM7
rs998038	6	157397855	0.1612	85.96%	0.154800	0.070200	0.02094000	0.05789000	NM_175863	intron	0	Hs.291587	ARID1B
rs10497527	2	179406841	0.1509	92.98%	1.000000	0.728300	0.02095000	0.19730000	ENST00000295723	3UTR	0	---	---
rs4752977	11	47257005	0.2344	95.79%	0.027000	0.080610	0.02095000	0.04253000	NM_130476	intron	0	Hs.82548	MADD
rs10504946	8	96370251	0.1483	82.81%	0.000007	0.010050	0.02097000	0.10750000	NM_177965	upstream	19657	Hs.548157	C8orf37
rs1213257	11	84898421	0.3029	97.89%	0.121100	0.703300	0.02099000	0.16440000	ENST00000383856	upstream	209	---	---
rs1480039	12	26313915	0.2500	77.89%	0.001974	0.044430	0.02099000	0.14990000	NM_005086	downstream	36781	Hs.183428	SSPN

rs1359379	10	91633312	0.4260	87.72%	0.001855	0.003280	0.02100000	0.01139000	NM_016195	downstream	108632	Hs.240	MPHOSPH1
rs1887291	14	87624362	0.1412	89.47%	1.000000	0.788100	0.02100000	0.03728000	NM_003608	downstream	76198	Hs.513440	GPR65
rs10492645	13	105818339	0.0119	88.77%	1.000000	1.000000	0.02105000	0.08382000	ENST00000310792	upstream	57783	---	---
rs774861	3	113167556	0.0913	88.42%	0.001705	0.003327	0.02105000	0.24690000	NM_145753	intron	0	Hs.477114	PHLDB2
rs2052003	17	12527057	0.4089	94.39%	0.450500	0.747700	0.02106000	0.07249000	NM_153604	intron	0	Hs.567641	MYOCD
rs9321060	6	126491345	0.2807	94.39%	0.879800	0.860000	0.02106000	0.01116000	NM_001031712	downstream	89234	Hs.404186	TRMT11
rs10490792	3	64251892	0.1043	97.54%	0.516400	0.092540	0.02107000	0.07985000	NM_198859	upstream	65721	Hs.699317	PRICKLE2
rs1115508	11	65679725	0.4982	97.19%	0.008056	0.034330	0.02109000	0.06916000	NM_018026	intron	0	Hs.644326	PACS1
rs763406	20	16952565	0.1385	97.54%	0.319400	1.000000	0.02109000	0.07095000	ENST00000387043	upstream	40887	---	---
rs10496012	2	53117375	0.0593	94.74%	1.000000	1.000000	0.02110000	0.00368800	ENST00000384021	downstream	433714	---	---
rs932877	10	17440957	0.3605	96.84%	0.067560	0.169100	0.02110000	0.07319000	NM_001004470	intron	0	Hs.677766	ST8SIA6
rs10499204	6	139258119	0.0354	89.12%	1.000000	1.000000	0.02112000	0.02313000	NM_031922	downstream	9195	Hs.334603	REPS1
rs10519734	5	123219082	0.1515	92.63%	1.000000	1.000000	0.02112000	0.03484000	NM_020747	downstream	781427	Hs.266616	ZNF608
rs2561445	2	107405193	0.2347	97.19%	0.866900	1.000000	0.02112000	0.08261000	NM_032528	upstream	536151	Hs.98265	ST6GAL2
rs712530	3	114498646	0.2302	97.54%	1.000000	0.845800	0.02112000	0.01075000	ENST00000308346	intron	0	---	---
rs1673997	5	87070011	0.4586	97.54%	0.547000	0.286200	0.02114000	0.02506000	NM_153354	downstream	456770	Hs.379972	TMEM161B
rs2086200	13	80698798	0.4182	94.39%	0.706900	0.362500	0.02114000	0.00750500	ENST00000387380	upstream	39085	---	---
rs10496201	2	76196768	0.1097	94.39%	0.026030	0.116000	0.02115000	0.06823000	NM_003203	upstream	404938	Hs.303808	C2orf3
rs10500757	11	12399173	0.4819	96.84%	0.003926	0.051080	0.02115000	0.05611000	NM_018222	intron	0	Hs.607144	PARVA
rs10518132	4	75985251	0.0599	93.68%	0.236200	1.000000	0.02115000	0.00903600	NM_001729	upstream	46540	Hs.591704	BTC
rs1284226	11	93246069	0.0573	97.89%	1.000000	1.000000	0.02117000	0.09229000	ENST00000384184	downstream	45523	---	---
rs9313473	5	169152577	0.1306	94.04%	0.276800	0.223300	0.02118000	0.00678500	NM_004946	intron	0	Hs.586174	DOCK2
rs1037254	13	38218761	0.3352	94.74%	0.339100	0.146000	0.02119000	0.00818100	NM_207361	intron	0	Hs.253994	FREM2
rs262036	5	177955387	0.2043	97.89%	0.202000	0.094880	0.02120000	0.03267000	NM_173465	upstream	5583	Hs.660026	COL23A1
rs9323852	14	90165038	0.4632	90.53%	0.103600	0.057150	0.02122000	0.03850000	NM_001010854	intron	0	Hs.655697	TTC7B
rs10511182	3	102255525	0.0665	97.54%	0.023700	0.049020	0.02124000	0.02020000	NM_015429	upstream	60586	Hs.477015	ABI3BP
rs2792695	10	113820301	0.1047	97.19%	0.750000	0.743800	0.02126000	0.01915000	NM_020918	downstream	79313	Hs.42586	GPAM
rs6850284	4	42441381	0.1047	97.19%	1.000000	1.000000	0.02126000	0.05385000	NM_006095	upstream	87722	Hs.435052	ATP8A1
rs9316935	13	56918224	0.4588	97.89%	0.548300	0.168600	0.02130000	0.02318000	NM_001040429	upstream	186432	Hs.106511	PCDH17
rs1197310	3	134610914	0.4730	97.54%	0.016420	0.048290	0.02131000	0.00033500	NM_003571	intron	0	Hs.659862	BFSP2
rs9325885	8	13210138	0.4523	91.93%	0.535400	0.638200	0.02131000	0.00939300	NM_024767	intron	0	Hs.134296	DLC1
rs10514033	18	67862234	0.0466	97.89%	0.457000	1.000000	0.02134000	0.12230000	NM_182511	downstream	492661	Hs.569851	CBLN2
rs3775567	4	185598236	0.0766	87.02%	0.007978	0.007897	0.02134000	0.17330000	NM_002199	intron	0	Hs.654566	IRF2
rs7667905	4	109506094	0.2025	97.89%	0.136400	0.060620	0.02134000	0.04176000	NM_016269	upstream	197067	Hs.555947	LEF1
rs10510981	3	69685275	0.1630	95.79%	0.006112	0.011850	0.02136000	0.13480000	NM_198178	upstream	210377	Hs.166017	MITF
rs1074463	5	22671126	0.3566	97.89%	0.241600	0.124200	0.02137000	0.05489000	NM_004061	intron	0	Hs.113684	CDH12
rs10515455	5	130906688	0.3229	95.09%	0.070270	0.194800	0.02138000	0.01181000	NM_016340	intron	0	Hs.483329	RAPGEF6
rs2207651	10	83554440	0.4315	87.02%	0.071390	0.513900	0.02138000	0.00664600	NM_001010848	upstream	70637	Hs.125119	NRG3
rs33422	5	71915951	0.2472	95.09%	0.416800	0.454300	0.02138000	0.00656700	NM_152625	upstream	76949	Hs.370303	ZNF366
rs6013989	20	52416192	0.0803	96.14%	1.000000	1.000000	0.02139000	0.13320000	NM_018431	upstream	109396	Hs.656582	DOK5
rs10506319	12	52505344	0.3058	97.54%	0.397900	0.868300	0.02140000	0.01104000	NM_020898	upstream	97858	Hs.156667	CALCOCO1
rs10492405	13	32659455	0.0632	91.58%	0.070510	0.083910	0.02142000	0.02062000	NM_052851	intron	0	Hs.507704	STARD13
rs718980	15	23325412	0.1606	96.14%	0.075710	0.046430	0.02143000	0.14540000	NM_130839	upstream	90191	Hs.654383	UBE3A
rs10499203	6	139257322	0.0332	95.09%	1.000000	1.000000	0.02144000	0.02205000	NM_031922	downstream	9992	Hs.334603	REPS1
rs10500928	11	22434893	0.2320	97.54%	0.501300	0.482900	0.02144000	0.04085000	NM_020346	downstream	77274	Hs.242821	SLC17A6
rs1491192	10	133792769	0.2320	97.54%	0.128400	0.061700	0.02144000	0.05017000	NM_194303	intron	0	---	C10orf39
rs6502302	17	13672493	0.0332	95.09%	1.000000	1.000000	0.02144000	0.03941000	NM_006042	upstream	226524	Hs.462270	HS3ST3A1
rs9302028	13	94394432	0.0336	94.04%	1.000000	1.000000	0.02145000	0.01622000	NM_005845	downstream	75658	Hs.508423	ABCC4
rs766132	15	45368231	0.4213	93.68%	0.616000	0.542000	0.02147000	0.00541300	NM_024966	upstream	471057	Hs.511265	SEMA6D
rs1276535	6	112989635	0.3333	94.74%	0.013970	0.105800	0.02148000	0.03901000	ENST00000386079	downstream	84914	---	---
rs4568840	1	220614011	0.4748	97.54%	0.809800	0.881700	0.02149000	0.02116000	ENST00000385440	downstream	24509	---	---
rs4044903	12	63635187	0.1577	97.89%	0.823100	0.803600	0.02150000	0.03385000	NM_007191	downstream	95487	Hs.284122	WIF1
rs10516683	4	84293850	0.2690	97.19%	0.361200	0.405500	0.02151000	0.11640000	NM_016619	upstream	38915	Hs.546392	PLAC8
rs10519816	15	31456613	0.1601	97.54%	0.501600	0.631100	0.02151000	0.04732000	NM_001036	upstream	88612	Hs.369250	RYR3

rs7828725	8	94208094	0.3313	88.42%	0.776000	1.000000	0.02153000	0.06689000	ENST00000378861	upstream	109017	---	---
rs649841	11	81554687	0.3175	96.14%	0.488900	0.321700	0.02154000	0.02265000	ENST00000357859	upstream	523562	---	---
rs7591597	2	138508913	0.4946	96.84%	1.000000	0.449100	0.02155000	0.07163000	NM_001024075	downstream	53195	Hs.42151	HNMT
rs10500801	11	13886095	0.2130	94.74%	1.000000	0.607400	0.02156000	0.04918000	NM_006108	upstream	54719	Hs.705394	SPON1
rs1425727	8	25642697	0.2363	89.82%	0.602800	1.000000	0.02156000	0.06063000	ENST00000380737	intron	0	---	---
rs4943136	13	33194632	0.3485	92.63%	1.000000	1.000000	0.02156000	0.04735000	NM_181558	upstream	95571	Hs.115474	RFC3
rs9290479	3	174993221	0.2699	96.84%	0.543900	0.092330	0.02158000	0.03151000	NM_014932	intron	0	Hs.478289	NLGN1
rs10491136	17	36376768	0.1077	96.14%	1.000000	1.000000	0.02160000	0.19200000	NM_213656	upstream	98	Hs.28467	KRT39
rs4099108	5	159910268	0.1429	95.79%	0.459100	0.590000	0.02160000	0.02571000	ENST00000385201	downstream	65233	---	---
rs4685356	3	16599918	0.3510	85.96%	0.001176	0.010160	0.02166000	0.08152000	NM_001351	downstream	3389	Hs.131179	DAZL
rs10510932	3	65122791	0.0150	93.33%	1.000000	1.000000	0.02167000	0.04694000	NM_001033057	downstream	194302	Hs.651939	MAGI1
rs10492886	16	55168116	0.0618	93.68%	0.608300	1.000000	0.02168000	0.03101000	NM_032935	downstream	7771	Hs.567624	MT4
rs2782327	10	19541172	0.0618	93.68%	0.066640	0.268800	0.02168000	0.02227000	ENST00000377266	downstream	2551	---	---
rs10490131	2	169759413	0.1848	96.84%	0.841400	1.000000	0.02170000	0.11250000	NM_004525	intron	0	Hs.657729	LRP2
rs4128360	13	100361380	0.4964	97.89%	0.403400	0.452900	0.02170000	0.00704100	NM_052867	downstream	142749	Hs.525146	NALCN
rs1519860	20	4871286	0.0789	97.89%	0.682000	0.463500	0.02171000	0.00132000	NM_203327	intron	0	Hs.516866	SLC23A2
rs172570	5	152699029	0.0789	97.89%	0.682000	1.000000	0.02171000	0.02783000	NM_000827	upstream	151470	Hs.519693	GRIA1
rs304863	5	152700181	0.0789	97.89%	0.682000	1.000000	0.02171000	0.02783000	NM_000827	upstream	150318	Hs.519693	GRIA1
rs9325452	10	82375021	0.0789	97.89%	0.393100	1.000000	0.02171000	0.04792000	NM_207372	intron	0	Hs.147643	SH2D4B
rs10483434	14	32759374	0.1302	84.91%	0.040310	0.225800	0.02172000	0.09708000	NM_173159	intron	0	Hs.659456	NPAS3
rs2172520	12	90994568	0.0635	91.23%	0.608300	1.000000	0.02172000	0.00779800	NM_001731	downstream	66462	Hs.255935	BTG1
rs2906388	7	10049603	0.0154	91.23%	1.000000	1.000000	0.02172000	0.06398000	ENST00000387411	upstream	179346	---	---
rs613623	1	238012069	0.2345	96.49%	0.128700	0.238600	0.02173000	0.08068000	NM_000740	intron	0	Hs.7138	CHRM3
rs9294602	6	95202609	0.0759	78.60%	0.118500	0.137000	0.02176000	0.04427000	ENST00000386414	downstream	10953	---	---
rs10491882	9	112933887	0.3700	97.19%	0.699300	0.400700	0.02177000	0.13840000	NM_205859	downstream	195697	Hs.381312	OR2K2
rs3846306	4	149350714	0.4394	92.63%	0.454800	0.031710	0.02178000	0.02177000	NM_000901	intron	0	Hs.163924	NR3C2
rs10500608	11	4628914	0.1149	91.58%	0.058010	0.021970	0.02180000	0.08269000	NM_152430	intron	0	Hs.669427	OR51E1
rs10508956	10	53304425	0.1775	91.93%	0.831800	1.000000	0.02180000	0.01707000	NM_006258	intron	0	Hs.654556	PRKG1
rs1360470	9	92476704	0.2944	87.02%	0.647500	0.685700	0.02180000	0.13860000	NM_017594	upstream	31776	Hs.165636	DIRAS2
rs1148259	10	37548456	0.4892	97.89%	0.337800	0.364800	0.02183000	0.01968000	NM_052997	CDS	0	Hs.373787	ANKRD30A
rs1864175	5	125191894	0.4946	97.54%	0.904500	0.763500	0.02183000	0.01232000	NM_023927	upstream	595106	Hs.363558	GRAMD3
rs7667916	4	76251680	0.0473	96.49%	0.462000	1.000000	0.02183000	0.04998000	NM_015393	downstream	57333	Hs.105460	DKFZP564O
rs1384737	15	91670604	0.2736	96.84%	0.652200	0.288500	0.02184000	0.02688000	ENST00000384809	upstream	40048	---	---
rs2328413	20	19495298	0.3832	96.14%	1.000000	0.742100	0.02184000	0.04614000	NM_020689	intron	0	Hs.654790	SLC24A3
rs10499943	7	97621894	0.1751	90.18%	0.030820	0.016170	0.02185000	0.00671000	NM_014916	intron	0	Hs.444179	LMTK2
rs10505301	8	118479876	0.2061	97.89%	0.713900	0.441000	0.02186000	0.12090000	NM_080651	upstream	122335	Hs.492612	MED30
rs2202802	6	54275640	0.3300	87.72%	0.022140	0.184100	0.02186000	0.00047790	NM_014464	upstream	5522	Hs.127011	TINAG
rs10488495	7	76287232	0.0498	95.09%	1.000000	1.000000	0.02187000	0.00771300	NM_152992	upstream	192733	Hs.488877	POMZP3
rs10518064	4	70455669	0.2680	97.54%	0.359900	0.288900	0.02187000	0.23630000	NM_006798	downstream	33893	Hs.225950	UGT2A1
rs2683687	2	67612167	0.2680	97.54%	0.541600	0.479500	0.02187000	0.02471000	NM_019002	downstream	120989	Hs.353022	ETAA1
rs10504211	8	58039887	0.2205	92.28%	0.071550	0.310700	0.02188000	0.09296000	NM_017813	intron	0	Hs.438689	IMPAD1
rs2305484	2	108271462	0.4734	85.61%	0.198600	0.420600	0.02189000	0.07355000	NM_176825	upstream	65	Hs.436123	SULT1C2
rs7782296	7	124240337	0.3635	91.23%	0.502900	0.201200	0.02189000	0.08458000	NM_001042594	downstream	10875	Hs.31968	POT1
rs10518062	4	70438590	0.2688	97.89%	0.365100	0.374400	0.02190000	0.22890000	NM_021139	upstream	42378	Hs.285887	UGT2B4
rs10501109	11	29017696	0.1075	97.89%	0.538400	1.000000	0.02191000	0.04202000	NM_152636	downstream	706177	Hs.243326	METT5D1
rs10519347	5	112621246	0.1848	96.84%	0.841400	0.577900	0.02193000	0.04446000	NM_002387	intron	0	Hs.593171	MCC
rs600589	18	8821217	0.4331	94.39%	0.456300	0.880200	0.02193000	0.11460000	NM_015210	intron	0	Hs.650822	KIAA0802
rs501125	11	91996379	0.0978	95.09%	1.000000	0.258300	0.02194000	0.04614000	ENST00000298047	intron	0	---	---
rs9290490	3	175243160	0.1345	92.63%	0.059760	0.228600	0.02195000	0.11760000	NM_014932	intron	0	Hs.478289	NLGN1
rs1420533	16	51121127	0.3883	95.79%	0.898500	1.000000	0.02196000	0.00544700	ENST00000388816	intron	0	---	---
rs10494574	1	182277350	0.3142	91.58%	0.062130	0.064190	0.02197000	0.08706000	NM_015101	upstream	3864	Hs.387995	GLT25D2
rs1371230	4	178378609	0.3714	96.84%	0.199500	0.275400	0.02197000	0.06536000	NM_018248	upstream	89413	Hs.405467	NEIL3
rs1812583	15	37541006	0.1134	94.39%	0.356900	0.328100	0.02199000	0.04594000	NM_003246	upstream	119566	Hs.164226	THBS1
rs2205892	1	167871378	0.4436	93.33%	0.214800	0.648400	0.02199000	0.15000000	NM_003005	upstream	5355	Hs.73800	SELP

rs9285586	14	59432923	0.4094	89.12%	0.245600	0.437300	0.02199000	0.04162000	NM_206852	upstream	265650	Hs.368626	RTN1
rs10504160	8	55175649	0.0148	95.09%	1.000000	1.000000	0.02204000	0.04087000	NM_006330	intron	0	Hs.435850	LYPLA1
rs1669539	2	105981706	0.0148	95.09%	1.000000	1.000000	0.02204000	0.00615400	NM_032411	upstream	66839	Hs.43125	C2orf40
rs2457555	6	160720302	0.1735	94.04%	0.831300	1.000000	0.02204000	0.02291000	NM_021977	intron	0	Hs.567337	SLC22A3
rs7946903	11	83607739	0.0148	95.09%	0.051040	1.000000	0.02204000	0.16270000	NM_001364	intron	0	Hs.654862	DLG2
rs10505128	8	110894494	0.0263	93.33%	1.000000	1.000000	0.02206000	0.05298000	NM_017786	upstream	164310	Hs.390738	GOLSYN
rs2125834	15	21661869	0.0688	96.84%	0.370700	0.625100	0.02206000	0.12530000	NM_018958	upstream	810239	Hs.649663	C15orf2
rs7769725	6	23673083	0.1801	91.58%	1.000000	1.000000	0.02208000	0.05514000	ENST00000330282	upstream	289778	---	---
rs7573495	2	266942	0.2698	97.54%	0.545900	1.000000	0.02210000	0.04079000	NM_004300	intron	0	Hs.558296	ACP1
rs10513009	5	52407631	0.0791	97.54%	1.000000	1.000000	0.02211000	0.06045000	NM_002203	intron	0	Hs.482077	ITGA2
rs6817452	4	87390273	0.2202	85.26%	0.000003	0.000073	0.02213000	0.24420000	NM_138981	intron	0	Hs.125503	MAPK10
rs10495674	2	18023796	0.1716	95.09%	1.000000	0.762400	0.02218000	0.02039000	NM_002252	downstream	46087	Hs.414489	KCNS3
rs4631686	1	14450061	0.1595	90.18%	0.004521	0.003925	0.02218000	0.01269000	NM_201628	upstream	848051	Hs.368823	RP1-21O18
rs10498453	14	53308392	0.1130	94.74%	0.758600	1.000000	0.02221000	0.03009000	NM_130851	downstream	177815	Hs.68879	BMP4
rs10511602	9	14918013	0.0686	97.19%	0.123800	0.129200	0.02221000	0.23620000	NM_144966	upstream	59038	Hs.50850	FREM1
rs1826349	3	95647674	0.2068	97.54%	0.714300	0.606200	0.02224000	0.16270000	NM_022072	downstream	319354	Hs.656338	NSUN3
rs10494617	1	187143468	0.0262	93.68%	0.160400	0.213100	0.02226000	0.10240000	NM_199051	downstream	1189952	Hs.65765	FAM5C
rs10501059	11	26625743	0.1835	97.54%	0.230500	0.772400	0.02227000	0.01908000	NM_031418	intron	0	Hs.91791	TMEM16C
rs1899951	3	12369840	0.1144	95.09%	0.366100	1.000000	0.02231000	0.02308000	NM_005037	intron	0	Hs.162646	PPARG
rs10520901	5	24949824	0.1264	95.79%	0.164600	0.078950	0.02233000	0.05678000	ENST00000363041	downstream	3265	---	---
rs1452237	12	83704259	0.2520	86.32%	0.002125	0.020830	0.02233000	0.01407000	NM_018057	downstream	94499	Hs.44424	SLC6A15
rs1925151	6	56208749	0.0484	97.89%	0.483500	0.529800	0.02233000	0.02781000	NM_030820	intron	0	Hs.47629	COL21A1
rs1418505	9	120108148	0.2594	93.33%	0.523300	0.855800	0.02236000	0.18430000	NM_014618	downstream	860581	Hs.532316	DBC1
rs4654438	1	4459761	0.3517	92.28%	0.138500	0.145100	0.02236000	0.06098000	NM_001042478	upstream	154891	Hs.25924	AJAP1
rs723595	8	51907585	0.3741	97.54%	1.000000	0.438800	0.02236000	0.04751000	NM_018967	downstream	39607	Hs.584914	SNTG1
rs9324486	8	139428681	0.2268	94.39%	0.295700	0.152800	0.02237000	0.00455500	NM_015912	intron	0	Hs.126024	FAM135B
rs1342501	9	120196645	0.0956	95.44%	0.721100	0.709800	0.02238000	0.01003000	NM_014618	downstream	772084	Hs.532316	DBC1
rs9309308	2	24848074	0.1138	94.04%	0.358100	0.535100	0.02238000	0.01174000	NM_147233	downstream	999	Hs.699183	NCOA1
rs1502800	12	85000386	0.3225	96.84%	0.131400	0.257900	0.02239000	0.05137000	NM_013244	upstream	32740	Hs.662231	MGAT4C
rs598218	6	92451486	0.0683	97.54%	0.028730	0.127600	0.02240000	0.04512000	NM_145333	upstream	1097858	Hs.644143	MAP3K7
rs9285217	13	58468127	0.2528	93.68%	0.747100	0.709000	0.02240000	0.03009000	ENST00000365514	upstream	468241	---	---
rs2279152	19	57791787	0.4382	88.07%	0.307400	0.149800	0.02241000	0.11230000	ENST00000333303	CDS	0	---	---
rs997737	5	90602787	0.3439	94.39%	0.006743	0.077740	0.02244000	0.03205000	ENST00000362773	downstream	348	---	---
rs1809139	2	173691122	0.3177	97.19%	0.052720	0.039850	0.02245000	0.04634000	NM_133646	intron	0	Hs.444451	ZAK
rs7995081	13	54781683	0.3125	92.63%	0.389700	0.182000	0.02245000	0.07045000	ENST00000258651	upstream	868232	---	---
rs259195	5	119128515	0.3257	84.56%	0.000380	0.007753	0.02246000	0.05292000	NM_182761	downstream	129100	Hs.406861	LOC340069
rs2619483	12	25593934	0.1089	87.02%	0.183500	0.487500	0.02246000	0.04233000	NM_152590	intron	0	Hs.44647	IFLTD1
rs4730462	7	110211723	0.2079	97.89%	0.277600	0.682300	0.02246000	0.05853000	NM_032549	intron	0	Hs.655722	IMMP2L
rs10516320	4	17740057	0.0148	94.74%	1.000000	1.000000	0.02247000	0.08338000	NM_153686	upstream	107460	Hs.446201	LCORL
rs1325197	1	191418940	0.3057	92.98%	0.885700	0.865700	0.02248000	0.02970000	NM_003783	intron	0	Hs.518834	B3GALT2
rs1394003	4	112589872	0.3674	97.89%	0.701300	0.437100	0.02249000	0.01586000	NM_152400	upstream	696255	Hs.23439	C4orf32
rs297961	5	162909730	0.0945	96.49%	1.000000	1.000000	0.02250000	0.00580700	NM_182796	downstream	30826	Hs.696057	MAT2B
rs4891315	18	63119836	0.3266	95.09%	1.000000	0.624100	0.02250000	0.01958000	NM_032160	downstream	204963	Hs.124673	DSEL
rs10510934	3	65124441	0.0152	92.63%	1.000000	1.000000	0.02254000	0.04698000	NM_001033057	downstream	192652	Hs.651939	MAGI1
rs10493729	1	82989954	0.1613	97.89%	0.663300	0.528400	0.02257000	0.04635000	NM_012302	downstream	759259	Hs.24212	LPHN2
rs2903611	19	36346051	0.3213	97.19%	0.782800	0.462400	0.02260000	0.29680000	NM_020856	downstream	112789	Hs.278436	TSHZ3
rs266508	1	180696351	0.3177	97.19%	0.126800	1.000000	0.02261000	0.00776600	NM_032267	intron	0	---	RGSL2
rs7936229	11	19204343	0.0841	81.40%	1.000000	1.000000	0.02261000	0.00846900	NM_024680	intron	0	Hs.523526	E2F8
rs3828488	4	178510024	0.2729	91.93%	0.641600	0.479500	0.02262000	0.05327000	NM_018248	intron	0	Hs.405467	NEIL3
rs715243	9	86406665	0.4387	94.39%	0.457500	0.653400	0.02262000	0.07908000	NM_022127	upstream	261245	Hs.591877	SLC28A3
rs4778189	15	25745823	0.4534	94.04%	1.000000	0.756400	0.02264000	0.02411000	NM_000275	intron	0	Hs.654411	OCA2
rs10496172	2	69893827	0.2122	97.54%	0.720000	0.803800	0.02265000	0.00951700	NM_001153	intron	0	Hs.422986	ANXA4
rs1063183	13	49387084	0.0815	96.84%	0.697400	1.000000	0.02266000	0.06340000	NM_020456	3UTR	0	Hs.44235	C13orf1
rs1384739	15	91670679	0.2742	97.89%	0.764000	0.292600	0.02268000	0.02466000	ENST00000384809	upstream	40123	---	---

rs10486507	7	32366358	0.2259	94.74%	0.863300	0.481700	0.02269000	0.08307000	NM_005020	upstream	288842	Hs.655694	PDE1C
rs10499732	7	54378030	0.3965	89.82%	0.298100	1.000000	0.02269000	0.00438500	NM_001001707	upstream	530920	---	FLJ45974
rs997435	1	19784062	0.0733	93.33%	0.153200	0.160500	0.02269000	0.02907000	NM_001032363	upstream	11992	Hs.466662	C1orf151
rs459784	5	129203740	0.0824	95.79%	0.094240	0.019840	0.02270000	0.02681000	NM_175856	upstream	64682	Hs.213137	CHSY-2
rs10499052	6	109992168	0.2077	91.23%	1.000000	0.396100	0.02272000	0.01042000	NM_001001706	upstream	13735	Hs.205144	FLJ42177
rs4425968	1	165420387	0.2293	93.33%	0.298900	0.479500	0.02272000	0.01650000	NM_002697	upstream	36372	Hs.493649	POU2F1
rs10495750	2	24847949	0.0502	94.39%	0.496700	0.176200	0.02273000	0.06257000	NM_147233	downstream	874	Hs.699183	NCOA1
rs9309549	2	79453610	0.1245	97.19%	1.000000	1.000000	0.02273000	0.15470000	NM_138937	upstream	213223	Hs.567312	REG3A
rs952851	12	85001472	0.3255	96.49%	0.101500	0.252900	0.02273000	0.05113000	NM_013244	upstream	33826	Hs.662231	MGAT4C
rs770918	1	98701525	0.0806	97.89%	0.694400	0.691100	0.02275000	0.03976000	NM_152238	upstream	198481	Hs.197015	SNX7
rs727954	1	193278268	0.2318	91.58%	0.083720	0.476100	0.02276000	0.02070000	NM_198503	downstream	1183268	Hs.657046	KCNT2
rs10483653	14	55915344	0.1530	94.04%	0.032230	0.483700	0.02277000	0.04153000	NM_021255	downstream	77571	Hs.657926	PELI2
rs1943723	11	84259364	0.4627	94.04%	1.000000	0.878000	0.02280000	0.07635000	ENST00000376104	intron	0	---	---
rs6974676	7	40974255	0.0531	89.12%	0.002729	0.186100	0.02280000	0.03341000	NM_002192	downstream	720871	Hs.583348	INHBA
rs10514994	5	65775312	0.2375	91.58%	1.000000	0.646300	0.02281000	0.01893000	NM_001001703	downstream	125303	---	FLJ46010
rs10515844	5	162175115	0.1655	96.49%	0.275700	0.094670	0.02281000	0.09907000	NM_199246	upstream	622040	Hs.79101	CCNG1
rs1540988	10	4061155	0.0891	90.53%	0.703400	0.696400	0.02282000	0.17740000	NM_001300	upstream	243688	Hs.4055	KLF6
rs871853	5	175214916	0.2299	91.58%	0.483400	0.327300	0.02283000	0.13060000	NM_001008220	intron	0	Hs.193235	CPLX2
rs10519505	15	23395922	0.1212	91.23%	0.075280	0.189700	0.02285000	0.10160000	NM_024490	downstream	77591	Hs.659258	ATP10A
rs7327037	13	37246306	0.4498	94.39%	0.712300	0.453500	0.02285000	0.04887000	NM_016179	intron	0	Hs.262960	TRPC4
rs10508250	10	3280142	0.1320	94.39%	0.591700	0.578400	0.02286000	0.00291100	NM_014889	upstream	75143	Hs.528300	PITRM1
rs10521242	16	49914711	0.1890	89.12%	0.683500	1.000000	0.02287000	0.02862000	NM_002968	upstream	172058	Hs.135787	SALL1
rs2827980	21	23524857	0.0701	97.54%	0.138800	0.362100	0.02290000	0.13270000	ENST00000364042	downstream	51108	---	---
rs10487144	7	98370725	0.0149	94.39%	0.051410	1.000000	0.02291000	0.04626000	NM_003496	intron	0	Hs.203952	TRRAP
rs10495263	1	225326507	0.0783	87.37%	0.177200	0.652400	0.02291000	0.00576800	NM_003607	intron	0	Hs.35433	CDC42BPA
rs10515518	5	142237775	0.0259	94.74%	1.000000	1.000000	0.02291000	0.07999000	NM_015071	intron	0	Hs.654668	ARHGAP26
rs10501382	11	59789067	0.1276	85.26%	0.244800	0.648800	0.02292000	0.04383000	NM_148975	upstream	15583	Hs.325960	MS4A4A
rs10499871	7	82336411	0.1500	94.74%	0.089270	0.316100	0.02293000	0.06822000	ENST00000389476	intron	0	---	---
rs10521231	17	13455247	0.1343	94.04%	0.110400	0.395600	0.02293000	0.01001000	NM_006042	upstream	9278	Hs.462270	HS3ST3A1
rs2124297	18	9651222	0.1802	90.53%	0.020750	0.033820	0.02293000	0.00820600	NM_006868	upstream	47043	Hs.99528	RAB31
rs10519250	15	48056765	0.2292	92.63%	0.162600	0.141200	0.02294000	0.03528000	NM_024837	intron	0	Hs.511311	ATP8B4
rs445945	20	15019192	0.2050	91.58%	0.058280	0.423700	0.02294000	0.14410000	NM_001033086	intron	0	---	MACROD2
rs643630	6	124839601	0.2471	89.47%	0.867000	1.000000	0.02294000	0.00404500	NM_001040214	intron	0	Hs.656604	NKAIN2
rs10515379	5	107167562	0.1930	95.44%	0.439800	0.783800	0.02296000	0.15080000	ENST00000386422	upstream	6664	---	---
rs1372393	2	154401057	0.0712	96.14%	0.633900	1.000000	0.02297000	0.04122000	NM_052917	upstream	35655	Hs.470277	GALNT13
rs6135875	20	16675859	0.2167	94.74%	1.000000	0.682200	0.02297000	0.01503000	NM_020157	upstream	1144	Hs.41119	OTOR
rs2939659	8	56549343	0.2455	96.49%	0.514500	0.112400	0.02298000	0.01059000	NM_052898	intron	0	Hs.130197	XKR4
rs717326	9	21948524	0.2800	96.49%	0.071510	0.413600	0.02298000	0.00817400	ENST00000380190	intron	0	---	---
rs939344	8	61158811	0.1619	97.54%	1.000000	1.000000	0.02298000	0.07199000	NM_004056	downstream	105166	Hs.654388	CA8
rs2349775	7	8684605	0.2401	97.19%	0.510700	0.256400	0.02301000	0.02827000	NM_152745	upstream	72558	Hs.487564	NXPH1
rs2966312	16	83164493	0.2818	96.49%	0.298200	0.294300	0.02302000	0.04726000	NM_021149	intron	0	Hs.289092	COTL1
rs10489974	2	101815260	0.0809	97.54%	0.695400	0.691100	0.02303000	0.02172000	NM_145687	intron	0	Hs.431550	MAP4K4
rs10512559	17	66544068	0.3569	94.39%	0.894300	0.753500	0.02303000	0.04890000	ENST00000388517	upstream	29251	---	---
rs10488462	7	134160338	0.0728	94.04%	0.038950	0.156400	0.02304000	0.10870000	NM_033140	intron	0	Hs.490203	CALD1
rs454686	21	17182222	0.3011	94.39%	0.385500	0.183200	0.02305000	0.02915000	ENST00000365203	downstream	168875	---	---
rs10521192	16	48354035	0.4451	86.32%	0.070360	0.057380	0.02306000	0.00675300	NM_015069	intron	0	Hs.530930	ZNF423
rs1567442	6	160777305	0.2357	92.28%	1.000000	0.440100	0.02307000	0.01567000	NM_021977	intron	0	Hs.567337	SLC22A3
rs3949926	1	87332366	0.0897	64.56%	0.007491	0.043910	0.02309000	0.08244000	NM_012262	intron	0	Hs.48823	HS2ST1
rs720863	6	76664374	0.4776	94.04%	0.177600	0.289400	0.02309000	0.09761000	NM_004999	intron	0	Hs.149387	MYO6
rs10518676	15	38037969	0.3895	96.84%	0.527500	0.877600	0.02310000	0.05720000	NM_001013703	intron	0	Hs.656673	EIF2AK4
rs10518932	15	35376766	0.1886	95.79%	0.232500	0.389100	0.02311000	0.17050000	NM_020149	upstream	196093	---	MEIS2
rs1597082	3	68066123	0.2419	97.89%	0.140900	0.851100	0.02311000	0.29720000	NM_213609	upstream	72337	Hs.655061	FAM19A1
rs220793	6	165727492	0.3231	97.19%	0.492700	0.365500	0.02311000	0.01707000	NM_006661	intron	0	Hs.584856	PDE10A
rs10491361	5	149188961	0.1434	97.89%	1.000000	0.474800	0.02312000	0.00644100	NM_133263	intron	0	Hs.591261	PPARGC1B

rs10491533	9	112904471	0.3537	94.74%	0.507400	0.862700	0.02312000	0.11510000	NM_205859	downstream	225113	Hs.381312	OR2K2
rs10513692	3	172322849	0.0157	89.12%	1.000000	1.000000	0.02312000	0.03249000	NM_015028	intron	0	Hs.34024	TNIK
rs7334629	13	60196965	0.0304	63.51%	0.007343	1.000000	0.02314000	0.10810000	ENST00000384127	upstream	64730	---	---
rs4780417	16	12270901	0.3679	92.98%	0.291000	0.308900	0.02315000	0.47650000	ENST00000306030	intron	0	---	---
rs846963	6	108075563	0.1000	92.98%	0.309500	1.000000	0.02317000	0.13830000	NM_018013	intron	0	Hs.445244	SOBP
rs10520528	4	183497595	0.3500	87.72%	0.025580	0.203000	0.02318000	0.03525000	ENST00000330336	intron	0	---	---
rs10496836	2	140665189	0.5000	78.60%	0.000000	0.000004	0.02320000	0.03180000	ENST00000385554	upstream	26102	---	---
rs10503938	8	32979287	0.0109	96.84%	0.027070	1.000000	0.02322000	0.04070000	ENST00000387834	upstream	9219	---	---
rs2807562	1	15106616	0.2164	96.49%	0.285100	0.103100	0.02322000	0.14350000	NM_201628	intron	0	Hs.368823	RP1-21O18
rs558325	1	66537540	0.0784	89.47%	1.000000	1.000000	0.02322000	0.05573000	NM_001037341	intron	0	Hs.198072	PDE4B
rs1765738	13	100181091	0.0116	90.88%	1.000000	1.000000	0.02323000	0.09069000	ENST00000376232	upstream	30870	---	---
rs10501793	11	91957395	0.1531	95.09%	1.000000	0.489600	0.02324000	0.02530000	ENST00000298047	intron	0	---	---
rs1443513	3	64399311	0.3539	93.68%	0.893800	0.630100	0.02327000	0.08321000	NM_198859	upstream	213140	Hs.699317	PRICKLE2
rs10511760	9	25556450	0.3320	88.77%	0.001802	0.011010	0.02328000	0.04578000	NM_001004125	downstream	111231	Hs.26268	TUSC1
rs10503549	8	15632795	0.0251	97.89%	1.000000	1.000000	0.02332000	0.16110000	NM_178234	intron	0	Hs.591845	TUSC3
rs4108601	2	51729670	0.0251	97.89%	0.153900	0.202100	0.02332000	0.03360000	ENST00000378257	upstream	620761	---	---
rs1914812	3	118196781	0.0701	97.54%	1.000000	1.000000	0.02333000	0.20750000	ENST00000363502	upstream	30018	---	---
rs1355806	4	69674823	0.2906	71.23%	0.500600	0.639200	0.02334000	0.01117000	NM_001075	upstream	41479	Hs.201634	UGT2B10
rs1028777	8	139146388	0.2156	96.84%	0.860000	0.684300	0.02336000	0.01776000	NM_015912	downstream	67320	Hs.126024	FAM135B
rs10509069	10	58405558	0.2130	97.19%	0.029810	0.025160	0.02336000	0.36400000	NM_001005414	upstream	614516	---	ZWINT
rs17062422	6	102284737	0.0149	94.04%	0.051600	1.000000	0.02336000	0.02101000	NM_175768	intron	0	Hs.654523	GRIK2
rs643599	7	42950715	0.1867	81.75%	0.200400	0.765300	0.02336000	0.06432000	NM_031903	downstream	6739	Hs.50252	MRPL32
rs10504995	8	101236990	0.2510	88.77%	0.003993	0.012540	0.02338000	0.03433000	NM_005034	downstream	1584	Hs.351475	POLR2K
rs1424118	16	6051074	0.1649	97.89%	0.828400	1.000000	0.02338000	0.01977000	NM_001013705	upstream	316691	---	LOC440337
rs4887987	16	77371955	0.1884	96.84%	0.692800	0.157500	0.02339000	0.30840000	NM_130844	intron	0	Hs.461453	WWOX
rs2042741	15	42177578	0.1007	94.04%	1.000000	1.000000	0.02340000	0.13550000	NM_032892	intron	0	Hs.578544	FRMD5
rs431903	5	95003757	0.0746	94.04%	0.046370	0.047940	0.02340000	0.01999000	NM_173362	upstream	4557	Hs.399758	RFESD
rs6503349	17	10874193	0.0752	93.33%	0.171900	0.180300	0.02340000	0.04338000	ENST00000386899	upstream	127304	---	---
rs10509984	10	115457298	0.0396	97.54%	0.351000	1.000000	0.02341000	0.06265000	NM_033339	intron	0	Hs.9216	CASP7
rs2419912	5	157777113	0.2826	96.84%	0.237000	0.429200	0.02341000	0.05833000	NM_024007	downstream	281584	Hs.657753	EBF1
rs9288163	2	189721391	0.1873	88.07%	0.094830	0.545300	0.02343000	0.12500000	NM_000393	intron	0	Hs.445827	COL5A2
rs7602328	2	28088688	0.3684	93.33%	0.116100	0.393700	0.02345000	0.08940000	NM_199192	intron	0	Hs.258314	BRE
rs1489635	2	163736991	0.2574	94.74%	0.427200	1.000000	0.02346000	0.11350000	NM_173162	upstream	333717	Hs.657413	KCNH7
rs16952186	15	37812517	0.2182	96.49%	1.000000	0.216200	0.02346000	0.01996000	NM_152597	intron	0	Hs.129598	FSIP1
rs9319810	18	52024294	0.2770	97.54%	0.550900	0.220900	0.02346000	0.03160000	NM_004786	downstream	396759	Hs.114412	TXNL1
rs10506378	12	57068256	0.0253	97.19%	1.000000	1.000000	0.02347000	0.02480000	NM_153377	downstream	483949	Hs.253736	LRIG3
rs6748937	2	184738190	0.2698	92.98%	0.639600	0.719000	0.02349000	0.03902000	NM_194250	upstream	433742	Hs.159528	ZNF804A
rs7129170	11	35711040	0.0996	95.09%	0.316900	0.291800	0.02350000	0.09387000	NM_017583	intron	0	Hs.591987	TRIM44
rs16915547	11	90461508	0.1225	88.77%	0.000034	0.000897	0.02352000	0.03470000	NM_012124	upstream	865681	Hs.22857	CHORDC1
rs6453843	6	76615205	0.0519	94.74%	0.150000	0.178000	0.02353000	0.06290000	NM_004999	intron	0	Hs.149387	MYO6
rs10505998	12	26428161	0.0722	94.74%	0.038020	0.037810	0.02354000	0.07406000	NM_002223	intron	0	Hs.512235	ITPR2
rs10486765	7	15599931	0.4889	95.09%	0.330800	0.233500	0.02355000	0.19370000	NM_005924	downstream	17431	Hs.170355	MEOX2
rs2223995	6	134977457	0.3223	89.82%	0.151500	0.232900	0.02356000	0.00484900	NM_005627	upstream	439777	Hs.510078	SGK1
rs10513169	9	114633691	0.0741	94.74%	0.166400	0.173700	0.02357000	0.04182000	ENST00000277244	intron	0	---	---
rs10500269	19	39174218	0.0522	94.04%	0.026730	0.033750	0.02359000	0.03210000	ENST00000387184	upstream	63848	---	---
rs1073572	17	66467656	0.2133	97.89%	0.720600	0.684200	0.02360000	0.03556000	NM_000891	downstream	779901	Hs.1547	KCNJ2
rs1679384	12	74247599	0.2970	95.09%	0.041790	0.439500	0.02362000	0.04508000	NM_007043	upstream	55940	Hs.645517	KRR1
rs1383673	4	12267660	0.4182	96.49%	0.385300	0.427300	0.02365000	0.03280000	ENST00000387175	downstream	344248	---	---
rs17700588	2	18581216	0.0887	92.98%	0.441200	0.434200	0.02366000	0.01438000	NM_020905	downstream	18254	Hs.288880	RDH14
rs9639563	7	27721012	0.4283	83.16%	0.508500	0.872900	0.02366000	0.04144000	NM_006024	upstream	25321	Hs.34576	TAX1BP1
rs10500638	11	5346395	0.4852	95.09%	0.144400	0.365200	0.02367000	0.03732000	NM_005330	intron	0	Hs.655195	HBE1
rs6424701	1	80639887	0.2286	94.39%	1.000000	0.560000	0.02368000	0.07527000	ENST00000388387	upstream	850175	---	---
rs4964063	12	23085765	0.1167	94.74%	0.550900	1.000000	0.02373000	0.08515000	NM_178010	downstream	490734	Hs.657542	SOX5
rs1401968	3	152993775	0.4297	89.82%	0.055430	0.145900	0.02376000	0.17160000	NM_001086	upstream	20776	Hs.506908	AADAC

rs10508262	10	3741274	0.2151	97.89%	0.477700	0.621400	0.02377000	0.04473000	NM_001300	downstream	66914	Hs.4055	KLF6
rs1604105	5	83657237	0.3132	92.98%	0.475200	1.000000	0.02377000	0.00779000	NM_005711	intron	0	Hs.482730	EDIL3
rs7206384	16	51299759	0.1753	95.09%	0.832900	0.815600	0.02377000	0.02448000	NM_025134	upstream	447580	Hs.59159	CHD9
rs1560469	17	51479991	0.4179	96.14%	0.012900	0.048080	0.02378000	0.03006000	NM_153228	upstream	105850	Hs.673040	ANKFN1
rs2344804	4	158613607	0.4649	95.09%	0.903400	0.760100	0.02378000	0.00984300	NM_000826	downstream	106930	Hs.32763	GRIA2
rs1981261	6	63806154	0.3622	78.95%	0.061720	0.009793	0.02379000	0.00899400	ENST00000356170	upstream	173267	---	---
rs4666764	2	189350464	0.2205	89.12%	0.583200	0.540600	0.02379000	0.04142000	NM_052952	intron	0	Hs.470892	DIRC1
rs285050	13	97560454	0.0827	97.54%	1.000000	0.499000	0.02380000	0.12240000	NM_001001715	upstream	32981	Hs.403917	FARP1
rs1995381	5	75606414	0.1463	86.32%	0.007867	0.090830	0.02381000	0.07868000	NM_014979	intron	0	Hs.663229	SV2C
rs681870	9	29529147	0.1113	86.67%	0.018440	0.098660	0.02382000	0.02042000	NM_152570	upstream	868864	Hs.699432	LINGO2
rs1412116	10	34113154	0.1875	44.91%	0.000069	0.002500	0.02383000	0.16320000	NM_019619	downstream	326949	Hs.131489	PARD3
rs1466863	16	68461639	0.3333	95.79%	0.496000	0.627200	0.02383000	0.15240000	NM_199423	intron	0	Hs.408458	WWP2
rs1386389	4	112172719	0.3244	97.89%	0.101200	0.363400	0.02389000	0.01945000	NM_000325	upstream	409016	Hs.643588	PITX2
rs9312601	4	177604036	0.4025	97.19%	0.261300	0.069490	0.02389000	0.37150000	NM_021928	downstream	116698	Hs.42194	SPCS3
rs250888	5	131043245	0.3280	97.89%	0.175800	0.367700	0.02391000	0.01254000	NM_001008738	intron	0	Hs.591273	FNIP1
rs10503371	8	6376190	0.3818	90.53%	0.597500	0.263700	0.02392000	0.02263000	NM_024596	intron	0	Hs.656769	MCPH1
rs4344939	2	18703181	0.2537	95.44%	0.038200	0.049780	0.02393000	0.05120000	NM_033253	upstream	68862	Hs.120319	NT5C1B
rs4448156	7	18051547	0.1101	97.19%	0.549900	0.743200	0.02394000	0.03456000	NM_175886	upstream	17620	Hs.169284	PRPS1L1
rs10516730	4	86200300	0.1612	75.09%	0.038430	0.054250	0.02395000	0.01894000	ENST00000365031	upstream	40373	---	---
rs10492036	12	124770007	0.1577	84.56%	0.146000	0.314800	0.02396000	0.04310000	NM_052907	downstream	64691	Hs.524838	TMEM132B
rs352716	15	100155950	0.3872	93.33%	0.093000	0.248800	0.02396000	0.00876500	NM_001005326	upstream	7496	Hs.553399	OR4F6
rs10512534	17	65490477	0.0155	90.53%	1.000000	1.000000	0.02398000	0.07744000	NM_002758	downstream	439410	Hs.463978	MAP2K6
rs6140226	20	7174483	0.4111	88.77%	0.698100	0.875200	0.02398000	0.02921000	NM_001200	downstream	465573	Hs.73853	BMP2
rs2216236	12	95615010	0.1105	96.84%	0.549600	0.625100	0.02399000	0.07979000	NM_198520	intron	0	Hs.436197	C12orf63
rs722151	4	159409295	0.2420	87.72%	0.489500	0.160900	0.02401000	0.00526600	NM_018342	downstream	13409	Hs.176227	TMEM144
rs229809	14	82983679	0.0849	95.09%	1.000000	0.509500	0.02403000	0.02280000	ENST00000387648	downstream	140449	---	---
rs2375988	4	36280171	0.1716	95.09%	0.668800	0.130000	0.02403000	0.01723000	ENST00000357504	downstream	258360	---	---
rs1196185	2	182593204	0.2630	94.74%	0.345100	0.527800	0.02404000	0.01191000	ENST00000280295	intron	0	---	---
rs2359189	12	49778776	0.1941	95.79%	0.331300	0.784800	0.02405000	0.03951000	NM_005653	intron	0	Hs.699156	TFCP2
rs851407	2	19307439	0.1115	97.54%	0.759400	0.530600	0.02405000	0.10340000	NM_145260	downstream	107288	Hs.123933	OSR1
rs10505420	8	123532174	0.1454	88.07%	0.071950	0.265600	0.02406000	0.01788000	NM_005328	upstream	809363	Hs.571528	HAS2
rs1597083	3	68067518	0.2004	93.68%	0.000002	0.000015	0.02407000	0.21740000	NM_213609	upstream	70942	Hs.655061	FAM19A1
rs1861245	2	102333338	0.4015	92.63%	0.372400	1.000000	0.02407000	0.12300000	NM_016232	intron	0	Hs.66	IL1RL1
rs756176	4	90218431	0.2072	92.28%	0.186900	0.529500	0.02408000	0.20620000	NM_001015045	upstream	21085	Hs.702033	FAM13A1
rs965010	11	96409418	0.2842	97.54%	1.000000	0.387500	0.02408000	0.05270000	ENST00000362445	upstream	624256	---	---
rs10496903	2	142325253	0.3217	85.61%	0.005124	0.012350	0.02409000	0.01040000	NM_018557	intron	0	Hs.656461	LRP1B
rs1344869	3	21282605	0.2879	81.05%	0.203600	0.277500	0.02411000	0.00672400	ENST00000295509	upstream	139919	---	---
rs1436524	4	87158801	0.1111	97.89%	0.356600	0.528400	0.02411000	0.10150000	NM_138981	intron	0	Hs.125503	MAPK10
rs1436336	3	106156256	0.1171	94.39%	0.382500	1.000000	0.02412000	0.00716000	NM_001627	upstream	412147	Hs.591293	ALCAM
rs1884022	1	185269042	0.3648	94.74%	0.600100	1.000000	0.02418000	0.06460000	NM_024420	downstream	44306	Hs.497200	PLA2G4A
rs8029355	15	48074273	0.1127	96.49%	0.361100	0.532800	0.02418000	0.04274000	NM_024837	intron	0	Hs.511311	ATP8B4
rs6563812	13	39741805	0.4758	94.39%	0.142900	0.217500	0.02423000	0.03148000	NM_002015	downstream	286012	Hs.370666	FOXO1
rs10489312	1	173793149	0.0696	83.16%	0.308900	1.000000	0.02425000	0.03805000	ENST00000367674	intron	0	---	---
rs1882257	3	172933446	0.3628	93.33%	0.427800	1.000000	0.02429000	0.15030000	NM_002662	intron	0	Hs.382865	PLD1
rs823692	6	7435616	0.0627	95.09%	0.609300	1.000000	0.02431000	0.06180000	NM_153005	downstream	72347	Hs.591353	RIOK1
rs492781	1	57522014	0.3966	91.58%	0.796800	1.000000	0.02434000	0.13400000	NM_021080	intron	0	Hs.477370	DAB1
rs9291501	4	27145749	0.3363	97.54%	0.140100	0.213400	0.02435000	0.04119000	ENST00000363691	upstream	311602	---	---
rs3806052	6	70943505	0.3849	92.98%	0.896800	0.441200	0.02436000	0.05407000	NM_001858	intron	0	Hs.444842	COL19A1
rs670044	8	9929939	0.3699	94.39%	1.000000	0.614300	0.02436000	0.01191000	NM_012331	upstream	19249	Hs.490981	MSRA
rs3768416	1	224988063	0.0507	96.84%	0.144700	0.175300	0.02441000	0.00614500	NM_002221	intron	0	Hs.528087	ITPKB
rs1163969	12	17425668	0.3978	97.89%	1.000000	0.449800	0.02442000	0.02066000	ENST00000386797	upstream	309962	---	---
rs1008582	7	33365456	0.1387	83.51%	0.586500	0.220200	0.02445000	0.00257600	NM_001033605	intron	0	Hs.372360	BBS9
rs710070	14	61145062	0.0243	86.67%	1.000000	1.000000	0.02448000	0.15110000	NM_006255	downstream	57619	Hs.333907	PRKCH
rs10505793	12	16215985	0.1426	89.82%	1.000000	1.000000	0.02450000	0.04232000	NM_015954	downstream	134509	Hs.39429	DERA

rs5005274	11	98341301	0.4152	97.19%	0.537700	0.338700	0.02450000	0.00301100	ENST00000388171	upstream	154827	---	---
rs9320736	6	120425841	0.4152	97.19%	0.804500	0.749900	0.02450000	0.01722000	NM_005907	upstream	713216	Hs.102788	MAN1A1
rs10484007	14	88452101	0.1920	96.84%	0.250300	0.278800	0.02453000	0.20550000	NM_198310	downstream	38014	Hs.303055	TTC8
rs4688360	3	63008489	0.1920	96.84%	0.078140	0.130300	0.02453000	0.26290000	NM_183393	upstream	172395	Hs.654933	CADPS
rs7179625	15	93984042	0.2210	96.84%	0.293600	0.112800	0.02453000	0.04217000	ENST00000365127	upstream	105995	---	---
rs4112338	9	117077093	0.0708	84.21%	1.000000	1.000000	0.02454000	0.07664000	NM_017418	intron	0	Hs.148841	01.Dec
rs10498813	6	56792678	0.1277	97.54%	1.000000	0.770500	0.02455000	0.03488000	NM_020388	intron	0	Hs.631992	DST
rs1075013	7	90125440	0.3642	92.98%	0.506800	0.082540	0.02455000	0.05297000	ENST00000306129	intron	0	---	---
rs10492061	12	4861546	0.3333	97.89%	0.282700	0.592800	0.02456000	0.03374000	NM_000217	upstream	29260	Hs.416139	KCNA1
rs6953623	7	31695505	0.1310	95.09%	1.000000	1.000000	0.02456000	0.08125000	NM_006658	intron	0	Hs.227011	C7orf16
rs2120091	4	85756849	0.4101	97.54%	0.536900	0.632100	0.02459000	0.07766000	NM_001263	intron	0	Hs.654899	CDS1
rs720080	6	163442734	0.0159	88.07%	1.000000	1.000000	0.02459000	0.05902000	NM_152410	intron	0	Hs.25791	PACRG
rs10483432	14	32751756	0.4341	95.79%	0.538800	0.360700	0.02464000	0.05627000	NM_022123	intron	0	Hs.659456	NPAS3
rs4671265	2	56028148	0.1957	88.77%	0.688600	1.000000	0.02464000	0.03578000	NM_001039349	upstream	25439	Hs.76224	EFEMP1
rs10483364	14	30265621	0.1550	95.09%	0.352300	0.316200	0.02465000	0.13150000	NM_182835	intron	0	Hs.369168	SCFD1
rs10500382	16	12065713	0.4241	90.18%	0.522900	0.438800	0.02467000	0.08808000	NM_032167	downstream	11071	Hs.458401	RUNDC2A
rs2274185	1	158587804	0.0630	94.74%	1.000000	1.000000	0.02467000	0.21190000	NM_015331	intron	0	Hs.517249	NCSTN
rs1071882	5	178068646	0.2724	94.04%	0.644900	0.724100	0.02468000	0.12740000	NM_005649	downstream	2491	Hs.484324	ZNF354A
rs543740	11	84468782	0.3346	91.23%	0.782000	1.000000	0.02468000	0.15910000	ENST00000376104	intron	0	---	---
rs10503331	8	5528064	0.0279	94.39%	0.181800	0.235800	0.02470000	0.11290000	ENST00000363751	downstream	227782	---	---
rs7741833	6	121042746	0.4604	79.65%	0.007845	0.134300	0.02470000	0.04620000	ENST00000363440	upstream	6721	---	---
rs10515327	5	101991415	0.2732	94.39%	0.127100	0.216300	0.02472000	0.10960000	NM_173488	upstream	128825	Hs.388874	SLCO6A1
rs6560414	9	76660881	0.3802	93.68%	0.155400	0.313100	0.02473000	0.01796000	NM_017662	intron	0	Hs.272225	TRPM6
rs2916707	8	6197122	0.1591	92.63%	0.257100	0.612900	0.02474000	0.05164000	NM_024596	upstream	54408	Hs.656769	MCPH1
rs6891270	5	165966509	0.2063	94.39%	0.457200	0.836700	0.02474000	0.01576000	ENST00000320147	downstream	224322	---	---
rs1911291	10	67976820	0.0571	89.12%	0.186400	1.000000	0.02475000	0.02241000	NM_013266	intron	0	Hs.660362	CTNNA3
rs7680105	4	147727101	0.1300	97.19%	1.000000	0.697700	0.02475000	0.02609000	NM_004575	upstream	52437	Hs.266	POU4F2
rs10508529	10	17504371	0.0278	94.74%	0.181100	0.234600	0.02476000	0.04707000	NM_001004470	intron	0	Hs.677766	ST8SIA6
rs1112401	7	151299255	0.2176	97.54%	0.295600	0.420000	0.02481000	0.03551000	NM_145292	intron	0	Hs.647077	GALNTL5
rs9293488	5	87103437	0.0762	94.39%	0.656200	1.000000	0.02481000	0.01735000	NM_153354	downstream	423344	Hs.379972	TMEM161B
rs9300791	13	102626893	0.3455	96.49%	0.593800	0.426600	0.02483000	0.09291000	NM_000452	upstream	109696	Hs.194783	SLC10A2
rs10490071	2	60522394	0.1685	97.89%	0.086770	0.055910	0.02485000	0.14490000	NM_138553	downstream	9412	---	BCL11A
rs2460401	2	7070780	0.1880	82.11%	0.201800	0.349900	0.02486000	0.04594000	NM_014746	intron	0	Hs.699230	RNF144A
rs1553273	4	101706843	0.1697	97.19%	0.393100	0.634100	0.02487000	0.02240000	NM_016242	upstream	48641	Hs.152913	EMCN
rs1962150	7	115664896	0.1272	97.89%	0.177400	0.246300	0.02487000	0.01833000	NM_152829	intron	0	Hs.592286	TES
rs9319686	18	60628256	0.2090	94.04%	0.136900	0.207100	0.02489000	0.02371000	ENST00000363325	upstream	260533	---	---
rs431370	4	31056923	0.4163	83.86%	1.000000	0.497900	0.02490000	0.09119000	NM_032457	downstream	303354	Hs.570785	PCDH7
rs952251	11	12406813	0.4632	90.53%	0.003918	0.084770	0.02491000	0.02285000	NM_018222	intron	0	Hs.607144	PARVA
rs1029494	7	80774410	0.0591	97.89%	0.608900	1.000000	0.02494000	0.03757000	NM_006379	upstream	387807	Hs.269109	SEMA3C
rs10501922	11	99118908	0.2395	92.28%	0.736500	0.646800	0.02494000	0.01143000	NM_175566	upstream	76565	Hs.656783	CNTN5
rs1897049	15	52255958	0.0591	97.89%	0.608900	1.000000	0.02494000	0.00762300	ENST00000260323	upstream	58563	---	---
rs709243	12	91203275	0.0591	97.89%	1.000000	1.000000	0.02494000	0.08231000	NM_001025232	downstream	134726	Hs.551199	CLLU1OS
rs10511619	9	16414985	0.3519	94.74%	0.061360	0.034440	0.02497000	0.00643500	NM_017637	intron	0	Hs.656581	BNC2
rs2378502	3	190786405	0.4273	96.49%	0.901900	0.544100	0.02501000	0.05663000	NM_003722	upstream	45505	Hs.137569	TP63
rs2033015	2	56556375	0.4140	97.89%	0.621700	0.879900	0.02502000	0.01279000	NM_006296	upstream	1570849	Hs.631890	VRK2
rs332148	10	28911251	0.1059	89.47%	0.175000	0.149100	0.02502000	0.00987200	NM_100486	intron	0	Hs.435610	WAC
rs7475474	10	122419759	0.4140	97.89%	0.805100	1.000000	0.02502000	0.01678000	NM_001012711	downstream	70149	---	C10orf85
rs1916818	7	22863400	0.0269	97.89%	1.000000	1.000000	0.02503000	0.00435400	NM_019059	upstream	34454	Hs.112318	TOMM7
rs3734590	6	7960719	0.1790	95.09%	0.539500	0.248600	0.02503000	0.02864000	NM_022085	intron	0	Hs.150837	TXNDC5
rs2861785	2	79453958	0.1185	94.74%	0.555000	1.000000	0.02504000	0.10840000	NM_138937	upstream	213571	Hs.567312	REG3A
rs9297611	8	121139152	0.0286	91.93%	1.000000	1.000000	0.02505000	0.03619000	NM_022783	downstream	7215	Hs.112981	DEPDC6
rs1503420	11	121071107	0.0441	91.58%	0.078610	0.099800	0.02506000	0.16070000	NM_003105	downstream	65510	Hs.368592	SORL1
rs236877	6	79362203	0.3509	96.49%	0.791300	0.874400	0.02507000	0.04339000	NM_001010844	upstream	271705	Hs.656212	IRAK1BP1
rs362341	14	72711211	0.0333	94.74%	0.028280	0.040480	0.02507000	0.10770000	NM_000021	intron	0	Hs.592324	PSEN1

rs10484417	6	67185952	0.0108	97.19%	0.026980	1.000000	0.02508000	0.10380000	ENST00000365187	downstream	309503	---	---
rs2548261	5	100155146	0.4038	92.98%	0.799700	0.627200	0.02512000	0.06655000	NM_175052	downstream	18110	Hs.308628	ST8SIA4
rs989433	21	38705678	0.2037	94.74%	0.004667	0.000203	0.02515000	0.03009000	NM_004449	intron	0	Hs.473819	ERG
rs10518015	4	67511540	0.1208	94.39%	0.083460	0.213700	0.02516000	0.03449000	ENST00000383902	downstream	466365	---	---
rs2034683	16	16913349	0.1521	84.21%	0.616600	0.786100	0.02516000	0.08566000	ENST00000381510	downstream	184210	---	---
rs3858021	9	10854239	0.2945	96.49%	0.664400	0.169900	0.02517000	0.01431000	ENST00000363183	downstream	1421887	---	---
rs149467	3	3016819	0.1806	92.28%	1.000000	0.644900	0.02518000	0.17150000	NM_175613	intron	0	Hs.298705	CNTN4
rs10499777	7	63618238	0.0562	90.53%	0.181800	1.000000	0.02519000	0.07741000	NM_178558	3UTR	0	Hs.520886	ZNF680
rs2743868	6	15733787	0.4519	83.86%	0.067300	0.415200	0.02522000	0.11740000	NM_183041	intron	0	Hs.571148	DTNBP1
rs7116734	11	120957150	0.3417	97.54%	0.023650	0.037250	0.02524000	0.04893000	NM_003105	intron	0	Hs.368592	SORL1
rs859362	1	173761713	0.1190	94.39%	1.000000	0.374300	0.02524000	0.04588000	ENST00000367674	intron	0	---	---
rs1032701	3	193231892	0.3569	89.47%	0.496400	0.745200	0.02525000	0.07299000	ENST00000364513	upstream	61713	---	---
rs861092	3	196179309	0.1703	97.89%	1.000000	0.761300	0.02527000	0.04840000	NM_152531	downstream	91310	Hs.478741	C3orf21
rs10504321	8	61975455	0.0618	93.68%	1.000000	1.000000	0.02531000	0.03519000	NM_017780	downstream	33438	Hs.20395	CHD7
rs10517254	4	32247093	0.0323	97.89%	1.000000	1.000000	0.02531000	0.00291300	NM_032457	downstream	1493524	Hs.570785	PCDH7
rs10520231	2	79149887	0.0323	97.89%	0.246600	0.295700	0.02531000	0.03186000	NM_006507	downstream	15772	Hs.4158	REG1B
rs3773559	3	55037455	0.0768	93.68%	1.000000	1.000000	0.02534000	0.02746000	NM_018398	intron	0	Hs.656687	CACNA2D3
rs10501851	11	95676492	0.1935	97.89%	0.179300	0.010960	0.02535000	0.02703000	NM_024725	downstream	49097	Hs.525088	CCDC82
rs10488112	7	10095101	0.0759	94.74%	0.655400	0.433500	0.02537000	0.00418500	ENST00000387411	upstream	133848	---	---
rs1392265	3	119283235	0.2555	96.14%	1.000000	0.712700	0.02537000	0.11830000	NM_001015887	downstream	820456	Hs.112873	IGSF11
rs10483412	14	32130473	0.3951	93.68%	0.161100	0.255100	0.02539000	0.01417000	NM_004274	intron	0	Hs.509083	AKAP6
rs873595	3	191000572	0.1816	93.68%	0.038460	0.139200	0.02540000	0.01269000	NM_003722	intron	0	Hs.137569	TP63
rs10515851	5	162584264	0.0594	97.54%	0.060240	0.264800	0.02541000	0.21360000	NM_199246	upstream	212891	Hs.79101	CCNG1
rs10515852	5	162588042	0.0594	97.54%	0.244300	0.264800	0.02541000	0.29870000	NM_199246	upstream	209113	Hs.79101	CCNG1
rs4845116	1	204839376	0.2033	86.32%	0.243300	0.375400	0.02542000	0.01954000	NM_006893	intron	0	Hs.497581	LGTN
rs10518013	4	67282795	0.1389	94.74%	0.317000	0.580000	0.02545000	0.06584000	ENST00000339867	downstream	639958	---	---
rs10495173	1	219889502	0.3694	94.04%	0.151300	0.606800	0.02547000	0.10540000	NM_144729	downstream	51887	Hs.497822	DUSP10
rs2366160	11	113830940	0.3522	96.14%	0.113000	0.387400	0.02549000	0.01655000	NM_015523	downstream	4729	Hs.7527	REXO2
rs1824306	2	71260077	0.1487	97.89%	0.475200	1.000000	0.02550000	0.10580000	NM_005791	downstream	29339	Hs.656208	MPHOSPH10
rs9321616	6	137882761	0.1394	94.39%	0.318800	0.470200	0.02550000	0.01723000	NM_175747	upstream	25681	Hs.195398	OLIG3
rs2270653	4	78160406	0.1033	95.09%	0.750300	0.608900	0.02551000	0.00425900	NM_018243	intron	0	Hs.128199	11.Sep
rs889962	2	21699691	0.0634	94.04%	1.000000	1.000000	0.02551000	0.04029000	ENST00000383859	upstream	299679	---	---
rs4755765	11	33599949	0.3921	84.56%	0.000674	0.023280	0.02556000	0.05141000	ENST00000389726	intron	0	---	---
rs362355	14	72707714	0.0352	94.74%	0.279600	0.338300	0.02558000	0.06387000	NM_000021	intron	0	Hs.592324	PSEN1
rs10514462	16	78146415	0.0344	96.84%	0.274300	0.330200	0.02559000	0.08500000	NM_001031804	downstream	39321	Hs.134859	MAF
rs2306129	8	99174902	0.3885	94.39%	0.523600	0.757400	0.02560000	0.00420800	NM_173549	3UTR	0	Hs.171455	C8orf47
rs2023124	8	123403249	0.1365	95.09%	0.438000	0.134100	0.02562000	0.04122000	NM_005328	upstream	680438	Hs.571528	HAS2
rs723416	21	19428994	0.1365	95.09%	0.438000	0.702300	0.02562000	0.01534000	NM_203430	downstream	276343	---	PPIA
rs10497242	2	164287000	0.0975	97.19%	0.156700	0.274800	0.02564000	0.02835000	NM_018086	intron	0	Hs.593650	FIGN
rs2029250	1	192520743	0.0341	97.89%	0.271700	0.328600	0.02564000	0.10570000	ENST00000385495	downstream	203113	---	---
rs347236	5	72898045	0.2458	83.51%	1.000000	0.633300	0.02564000	0.02655000	NM_032175	intron	0	Hs.406703	UTP15
rs10248833	7	24787486	0.2037	94.74%	1.000000	0.672700	0.02566000	0.11800000	NM_145321	downstream	15205	Hs.520259	OSBPL3
rs758113	17	10839468	0.1004	96.14%	0.742000	1.000000	0.02567000	0.02038000	ENST00000362504	upstream	109041	---	---
rs2613671	8	21083065	0.4375	84.21%	0.432400	0.429800	0.02568000	0.06992000	ENST00000387614	upstream	417187	---	---
rs1400593	12	116535961	0.0614	97.19%	0.269000	0.290900	0.02569000	0.05897000	NM_173598	intron	0	Hs.375836	KSR2
rs10509744	10	102261569	0.2342	94.39%	1.000000	1.000000	0.02570000	0.25260000	NM_015490	intron	0	Hs.18889	SEC31B
rs2955529	12	23900409	0.3510	89.47%	0.001559	0.002983	0.02570000	0.11830000	NM_178010	intron	0	Hs.657542	SOX5
rs5007492	18	21662551	0.3815	94.74%	0.155700	0.619400	0.02570000	0.04900000	NM_005637	downstream	187666	Hs.404263	SS18
rs6795120	3	59414325	0.4301	97.89%	0.903400	0.347200	0.02570000	0.01610000	NM_002012	downstream	298667	Hs.655995	FHIT
rs719125	2	201726105	0.2011	95.09%	0.059320	0.130900	0.02571000	0.04929000	NM_003879	intron	0	Hs.390736	CFLAR
rs3738367	1	222775211	0.2239	90.88%	0.006558	0.067220	0.02572000	0.02666000	NM_025160	upstream	86587	Hs.497873	WDR26
rs10495850	2	35991005	0.0541	94.04%	0.550900	0.206400	0.02575000	0.09629000	NM_016441	upstream	445568	Hs.699247	CRIM1
rs10511388	3	120550403	0.0466	86.67%	0.010330	0.014100	0.02575000	0.07889000	NM_020754	intron	0	Hs.657263	CDGAP
rs2141610	3	190784288	0.2315	94.74%	0.732800	1.000000	0.02575000	0.00830500	NM_198485	downstream	260324	Hs.338851	FAM79B

rs2465762	11	106423462	0.1942	97.54%	1.000000	1.000000	0.02575000	0.00487600	NM_000855	upstream	29081	Hs.654555	GUCY1A2
rs3912622	11	102626683	0.1982	96.49%	0.851300	1.000000	0.02575000	0.10790000	ENST00000334267	intron	0	---	---
rs149352	5	94224367	0.2809	93.68%	0.022230	0.104000	0.02576000	0.05903000	NM_001002796	intron	0	Hs.655087	MCTP1
rs565398	11	91858879	0.2774	92.98%	0.541000	0.721300	0.02576000	0.05556000	ENST00000298047	intron	0	---	---
rs10489834	1	156787658	0.4265	97.89%	0.179500	0.209800	0.02578000	0.10380000	NM_001005189	upstream	3139	Hs.553780	OR6Y1
rs10507046	12	93777538	0.4129	92.63%	0.899000	0.285000	0.02578000	0.01644000	NM_018838	downstream	111702	Hs.506374	NDUFA12
rs1343945	13	30380241	0.4265	97.89%	0.542000	0.531100	0.02578000	0.05183000	NM_032849	intron	0	Hs.646647	C13orf33
rs192166	5	130880424	0.3232	92.28%	0.007530	0.057900	0.02579000	0.01967000	NM_016340	intron	0	Hs.483329	RAPGEF6
rs801739	11	65679165	0.4927	96.14%	0.011300	0.050030	0.02580000	0.07739000	NM_018026	intron	0	Hs.644326	PACS1
rs10514280	5	85744155	0.2108	94.04%	0.855100	1.000000	0.02583000	0.01079000	NM_001867	upstream	205322	Hs.430075	COX7C
rs1955544	14	39844441	0.1960	97.54%	0.125200	0.198200	0.02587000	0.08662000	NM_203301	upstream	873070	Hs.324342	FBXO33
rs9308191	4	146805664	0.2637	95.79%	0.876300	0.523100	0.02587000	0.01177000	NM_172250	downstream	6371	Hs.452864	MMAA
rs10512394	9	111269044	0.1880	82.11%	0.829800	0.537200	0.02590000	0.04522000	NM_002829	intron	0	Hs.436429	PTPN3
rs574522	1	193212122	0.2299	96.14%	0.394500	1.000000	0.02590000	0.02837000	NM_198503	downstream	1249414	Hs.657046	KCNT2
rs6003160	22	42061618	0.3229	95.09%	0.486900	0.855900	0.02590000	0.02054000	NM_173050	intron	0	Hs.133995	SCUBE1
rs669143	11	109238959	0.0443	95.09%	1.000000	1.000000	0.02591000	0.12060000	NM_207645	downstream	437341	Hs.172982	LOC399947
rs6433083	2	169207560	0.2890	92.28%	0.016610	0.008746	0.02595000	0.05649000	NM_203463	intron	0	Hs.506829	LASS6
rs962586	13	96859208	0.1794	87.02%	0.004228	0.001650	0.02596000	0.02739000	NM_207304	downstream	16943	Hs.657347	MBNL2
rs10488165	7	132788184	0.4422	97.19%	0.714700	1.000000	0.02597000	0.07442000	NM_001037126	intron	0	Hs.321273	EXOC4
rs10497288	2	167084851	0.0989	97.54%	1.000000	1.000000	0.02597000	0.02553000	NM_002976	upstream	33124	Hs.695947	SCN7A
rs10503006	18	51720427	0.0426	94.74%	0.386800	0.433500	0.02597000	0.12170000	NM_001039759	downstream	126642	---	FLJ45743
rs292432	11	30692095	0.0444	94.74%	1.000000	1.000000	0.02597000	0.05128000	NM_001584	upstream	126982	Hs.289795	MPPED2
rs7500906	16	48354332	0.4691	96.49%	0.184400	0.230000	0.02598000	0.00644400	NM_015069	intron	0	Hs.530930	ZNF423
rs9318756	13	80700407	0.4057	92.98%	0.610100	0.215400	0.02599000	0.01287000	ENST00000387380	upstream	37476	---	---
rs10498023	2	215369955	0.0612	97.54%	0.070450	0.076000	0.02602000	0.03592000	NM_000465	intron	0	Hs.591642	BARD1
rs1986631	9	72282280	0.4157	87.37%	0.037450	0.138400	0.02602000	0.06802000	NM_001007471	downstream	57508	Hs.47288	TRPM3
rs2079437	7	28886197	0.0424	95.09%	0.385600	0.121800	0.02602000	0.19010000	NM_001011666	downstream	54161	Hs.437075	CREB5
rs1504037	3	6137218	0.0623	95.79%	1.000000	1.000000	0.02603000	0.09464000	NM_181875	upstream	740858	---	GRM7
rs6994971	8	91924984	0.4854	96.14%	0.117400	0.168400	0.02605000	0.09255000	NM_022351	intron	0	Hs.560892	NECAB1
rs2403590	11	20362222	0.2453	93.68%	1.000000	0.822500	0.02611000	0.01624000	NM_006410	downstream	318	Hs.90753	HTATIP2
rs10511980	9	71928159	0.3875	95.09%	0.040120	0.093530	0.02612000	0.05004000	NM_153267	intron	0	Hs.547172	MAMDC2
rs10513938	18	1577735	0.3409	84.91%	0.062090	0.453700	0.02612000	0.01108000	NM_022840	downstream	949795	Hs.126888	METTL4
rs1482581	2	213467362	0.2920	91.93%	0.653900	0.492100	0.02612000	0.06536000	NM_016260	downstream	112227	Hs.604950	IKZF2
rs2257086	6	116925713	0.2588	90.18%	0.253600	0.574400	0.02612000	0.07643000	ENST00000310560	intron	0	---	---
rs10510809	3	59414093	0.1953	97.89%	0.187300	0.389100	0.02613000	0.01832000	NM_002012	downstream	298899	Hs.655995	FHIT
rs1288547	4	186453130	0.4513	97.19%	0.627700	0.442800	0.02613000	0.00493800	NM_031953	intron	0	Hs.369091	SNX25
rs1386037	12	114623756	0.1971	97.89%	1.000000	1.000000	0.02613000	0.02006000	ENST00000386460	upstream	16151	---	---
rs16934682	10	33942758	0.0357	93.33%	0.283300	0.336600	0.02613000	0.03088000	NM_001024629	upstream	279302	Hs.131704	NRP1
rs2085852	2	230630	0.2636	96.49%	0.436600	1.000000	0.02613000	0.06482000	NM_015677	intron	0	Hs.515951	SH3YL1
rs950136	14	29433812	0.1953	97.89%	0.850100	1.000000	0.02613000	0.13270000	NM_002742	intron	0	Hs.508999	PRKD1
rs2871668	3	96407708	0.4431	89.47%	0.253000	0.114800	0.02614000	0.06919000	ENST00000332119	upstream	268150	---	---
rs971434	6	110084001	0.3271	94.39%	0.890400	0.870400	0.02614000	0.01558000	NM_145025	intron	0	Hs.486169	C6orf199
rs994952	6	78319674	0.4649	84.91%	0.052380	0.053990	0.02615000	0.05883000	NM_000863	upstream	89835	Hs.123016	HTR1B
rs1948369	1	101711149	0.3569	96.84%	0.693800	0.389500	0.02616000	0.08061000	NM_001400	downstream	231487	Hs.154210	EDG1
rs1923868	13	103620989	0.1022	96.14%	0.176900	0.153800	0.02617000	0.04103000	NM_000452	upstream	1103792	Hs.194783	SLC10A2
rs7576149	2	20789943	0.1053	93.33%	0.749600	1.000000	0.02620000	0.03289000	NM_021925	intron	0	Hs.187823	C2orf43
rs2174748	3	154521672	0.1275	89.47%	0.575400	0.541000	0.02622000	0.04797000	ENST00000383956	upstream	121176	---	---
rs4131049	10	124981594	0.3736	95.79%	0.440700	0.615000	0.02622000	0.04302000	NM_153442	upstream	434267	Hs.12751	GPR26
rs4665049	2	159817465	0.2918	90.18%	0.009793	0.032080	0.02623000	0.01050000	NM_152528	intron	0	Hs.20848	WDSUB1
rs4075701	2	116146260	0.1978	97.54%	0.573100	0.593900	0.02624000	0.02658000	NM_001004360	intron	0	Hs.591555	DPP10
rs1316575	6	54273259	0.4371	97.54%	0.542000	0.278200	0.02625000	0.00152500	NM_014464	upstream	7903	Hs.127011	TINAG
rs10483246	22	48069909	0.3007	96.84%	0.886300	0.440500	0.02627000	0.02248000	NM_207478	upstream	258424	---	FLJ44385
rs10488082	7	29911394	0.1456	91.58%	0.041460	0.268800	0.02627000	0.08621000	ENST00000242140	downstream	526	---	---
rs1631871	14	86545527	0.0360	92.63%	0.002510	0.004064	0.02627000	0.04460000	NM_001037525	downstream	923586	Hs.513439	GALC

rs2024374	7	7725084	0.1505	97.89%	0.003702	0.003013	0.02628000	0.00751000	NM_002947	upstream	321	Hs.487540	RPA3
rs721424	20	20287310	0.4604	97.54%	0.717400	1.000000	0.02629000	0.00603400	NM_015585	intron	0	Hs.176013	C20orf26
rs10510057	10	121301038	0.2581	97.89%	0.754400	0.830500	0.02630000	0.04889000	NM_002925	upstream	8826	Hs.501200	RGS10
rs1349734	2	129987676	0.2581	97.89%	0.436900	1.000000	0.02630000	0.02756000	ENST00000385692	downstream	86110	---	---
rs1513595	20	11600924	0.3533	96.84%	0.693300	0.603700	0.02630000	0.12740000	ENST00000318298	upstream	63632	---	---
rs9301634	13	89372499	0.2222	97.89%	0.297200	0.812400	0.02630000	0.02062000	ENST00000363863	downstream	85007	---	---
rs6882495	5	57478580	0.2138	94.39%	0.855700	0.838300	0.02633000	0.03768000	NM_006622	downstream	306986	Hs.398157	PLK2
rs2594480	2	79080378	0.2116	93.68%	0.142600	0.126100	0.02634000	0.05555000	NM_198448	upstream	25956	Hs.447084	REG3G
rs10516478	4	102610288	0.0352	94.74%	1.000000	1.000000	0.02636000	0.05341000	NM_000944	upstream	122912	Hs.435512	PPP3CA
rs10485927	7	78502422	0.0717	97.89%	1.000000	1.000000	0.02639000	0.03241000	NM_012301	intron	0	Hs.654788	MAGI2
rs1372705	9	17427735	0.3543	97.54%	0.238300	0.636900	0.02639000	0.18070000	NM_017738	intron	0	Hs.435381	C9orf39
rs6793392	3	43300839	0.4498	97.89%	0.468100	0.881100	0.02642000	0.02153000	NM_017719	upstream	2195	Hs.476052	SNRK
rs10504082	8	50426124	0.3569	96.84%	0.434800	0.428700	0.02643000	0.07067000	NM_018967	upstream	1043202	Hs.584914	SNTG1
rs961904	6	141324292	0.4361	93.33%	0.710000	1.000000	0.02645000	0.14550000	NM_006079	upstream	1586814	Hs.82071	CITED2
rs1397328	2	239233691	0.2220	90.88%	0.029970	0.039780	0.02646000	0.01341000	NM_001040445	downstream	213581	Hs.516788	ASB1
rs232779	1	58899264	0.0907	94.74%	0.251500	0.202100	0.02647000	0.05428000	ENST00000294610	intron	0	---	---
rs9582457	13	100618459	0.2529	91.58%	0.871200	0.576500	0.02647000	0.16180000	NM_052867	intron	0	Hs.525146	NALCN
rs1957491	14	43379465	0.4373	97.89%	0.903200	0.352800	0.02650000	0.06204000	NM_152447	downstream	1935967	Hs.136893	LRFN5
rs7795236	7	108046946	0.3012	90.88%	0.554900	0.605700	0.02653000	0.02418000	NM_012328	downstream	44416	Hs.6790	DNAJB9
rs6941115	6	97336333	0.2890	92.28%	0.293400	0.386200	0.02654000	0.18450000	NM_030784	downstream	16312	Hs.632612	GPR63
rs1921026	12	78188457	0.2815	94.74%	0.132100	0.080100	0.02655000	0.03860000	NM_005639	intron	0	Hs.310545	SYT1
rs10505077	8	106704679	0.0351	95.09%	1.000000	1.000000	0.02656000	0.01629000	NM_012082	intron	0	Hs.431009	ZFPM2
rs10520866	5	20576714	0.0351	95.09%	1.000000	1.000000	0.02656000	0.04007000	NM_004934	upstream	552618	Hs.317632	CDH18
rs3785214	16	7657122	0.4302	92.98%	0.318200	0.541900	0.02656000	0.01611000	NM_018723	intron	0	Hs.459842	A2BP1
rs9321772	6	140904839	0.4873	96.84%	0.401100	0.655100	0.02656000	0.08014000	NM_006079	upstream	1167361	Hs.82071	CITED2
rs10517898	4	167498959	0.0823	87.37%	0.674000	0.437400	0.02657000	0.01712000	NM_016950	downstream	392208	Hs.481133	SPOCK3
rs1067332	2	44480476	0.3605	90.53%	1.000000	0.730100	0.02658000	0.01364000	NM_024766	intron	0	Hs.468349	C2orf34
rs4266352	4	109685796	0.1727	97.54%	0.526800	0.814300	0.02660000	0.13080000	NM_016269	upstream	376769	Hs.555947	LEF1
rs10518726	15	51610704	0.2004	97.19%	0.137500	0.605200	0.02661000	0.03983000	NM_182758	intron	0	Hs.122125	WDR72
rs931217	2	213204695	0.1057	92.98%	0.053650	0.036280	0.02667000	0.15760000	NM_001042599	upstream	93196	Hs.390729	ERBB4
rs7734364	5	142416345	0.4586	97.54%	0.904100	0.654600	0.02668000	0.01686000	NM_015071	intron	0	Hs.654668	ARHGAP26
rs10507727	13	65268708	0.2617	97.19%	0.876600	0.832700	0.02669000	0.04155000	NM_020403	downstream	506264	Hs.654709	PCDH9
rs10494675	1	191417496	0.0399	83.51%	0.003388	0.061810	0.02671000	0.04315000	NM_003783	intron	0	Hs.518834	B3GALT2
rs1105244	2	66165727	0.1075	92.98%	0.200900	0.171000	0.02673000	0.09324000	NM_002398	upstream	350309	Hs.526754	MEIS1
rs2440359	15	50919088	0.1336	97.19%	0.295500	0.155800	0.02674000	0.02509000	NM_004498	upstream	49587	Hs.658573	ONECUT1
rs3798236	6	116416342	0.4601	83.51%	0.151500	0.098870	0.02674000	0.12420000	NM_002031	intron	0	Hs.89426	FRK
rs10490204	2	102422966	0.1654	93.33%	1.000000	0.746900	0.02675000	0.11890000	NM_003853	intron	0	Hs.158315	IL18RAP
rs10490910	10	118066445	0.1407	92.28%	0.019940	0.172600	0.02675000	0.07121000	NM_198515	upstream	7485	Hs.233407	C10orf96
rs6449674	5	63031694	0.4658	97.54%	0.630400	0.647000	0.02675000	0.00436800	NM_000524	downstream	260340	Hs.247940	HTR1A
rs1335989	10	33104406	0.0866	97.19%	0.133000	0.119200	0.02676000	0.04594000	NM_024688	intron	0	Hs.585464	C10orf68
rs6692851	1	34119149	0.1056	94.74%	0.193900	0.166000	0.02676000	0.02203000	NM_052896	intron	0	Hs.656915	CSMD2
rs6982790	8	122777359	0.1056	94.74%	0.749600	0.743800	0.02676000	0.06318000	NM_005328	upstream	54548	Hs.571528	HAS2
rs10509471	10	84937402	0.3668	96.14%	0.896700	0.865500	0.02677000	0.19750000	ENST00000363904	upstream	119595	---	---
rs10494630	1	188370938	0.2975	97.89%	1.000000	0.866100	0.02678000	0.03568000	NM_199051	intron	0	Hs.65765	FAM5C
rs10520186	2	77541306	0.0932	92.28%	0.057880	1.000000	0.02678000	0.04431000	NM_024993	intron	0	Hs.285782	LRRTM4
rs10486616	7	33752103	0.3222	94.74%	1.000000	1.000000	0.02679000	0.00384300	ENST00000311067	downstream	17513	---	---
rs4498221	5	54056959	0.2463	94.04%	0.031610	0.572200	0.02679000	0.13610000	NM_052870	downstream	205533	Hs.432755	SNAG1
rs651661	11	84366176	0.3234	94.39%	0.166600	0.137900	0.02680000	0.11930000	ENST00000376104	intron	0	---	---
rs803262	7	91196798	0.2625	90.88%	0.335200	0.202600	0.02680000	0.04553000	NM_006980	downstream	143159	Hs.532216	MTERF
rs2107472	7	8672612	0.2491	93.68%	0.254600	0.452200	0.02681000	0.03640000	NM_152745	upstream	84551	Hs.487564	NXPH1
rs10490405	2	56565471	0.4928	96.84%	0.717800	0.763300	0.02683000	0.00701700	NM_006296	upstream	1561753	Hs.631890	VRK2
rs9308877	2	104709728	0.3658	95.44%	0.360800	1.000000	0.02684000	0.02071000	ENST00000384520	upstream	6396	---	---
rs10507729	13	65590973	0.1426	92.28%	0.445200	0.243600	0.02687000	0.04522000	NM_020403	downstream	183999	Hs.654709	PCDH9
rs2423454	20	9667984	0.2608	97.54%	0.042140	0.071310	0.02687000	0.01315000	NM_177990	intron	0	Hs.32539	PAK7

rs4498548	8	122751374	0.0860	97.89%	0.704700	0.697500	0.02687000	0.05538000	NM_005328	upstream	28563	Hs.571528	HAS2
rs2798765	6	48609119	0.3077	82.11%	0.020660	0.032340	0.02688000	0.01767000	NM_207499	upstream	464820	---	C6orf138
rs4908004	1	99926262	0.1084	92.28%	0.521700	1.000000	0.02688000	0.05776000	NM_017734	intron	0	Hs.483993	PALMD
rs1364516	7	132017402	0.4819	97.19%	0.117800	0.450800	0.02689000	0.06302000	NM_181775	upstream	33415	Hs.675491	PLXNA4
rs2076756	16	49314382	0.1630	94.74%	0.260500	0.805700	0.02689000	0.02751000	NM_022162	intron	0	Hs.592072	NOD2
rs3891298	3	64219964	0.0719	97.54%	0.156000	0.397700	0.02689000	0.06036000	NM_198859	upstream	33793	Hs.699317	PRICKLE2
rs10498098	2	221337878	0.0845	97.54%	0.116300	1.000000	0.02690000	0.01652000	ENST00000385336	downstream	231306	---	---
rs10516579	4	112902972	0.0655	93.68%	0.090170	1.000000	0.02690000	0.08660000	NM_152400	upstream	383155	Hs.23439	C4orf32
rs1113253	10	57300806	0.0866	97.19%	1.000000	1.000000	0.02690000	0.06163000	NM_001005414	downstream	486805	---	ZWINT
rs1323154	13	42872326	0.4712	97.54%	0.053810	0.222100	0.02693000	0.03377000	NM_017993	intron	0	Hs.128258	ENOX1
rs7512592	1	156756569	0.4819	96.84%	0.116600	0.445400	0.02697000	0.24510000	NM_001005189	downstream	26973	Hs.553780	OR6Y1
rs10517409	4	37038537	0.0737	97.54%	1.000000	1.000000	0.02698000	0.01221000	ENST00000389006	intron	0	---	---
rs10513835	3	190517515	0.1624	95.09%	0.375900	0.459000	0.02699000	0.00989800	NM_198485	intron	0	Hs.338851	FAM79B
rs10501899	11	98419726	0.1757	96.84%	0.034820	0.034940	0.02700000	0.01242000	ENST00000388171	upstream	76402	---	---
rs1464077	1	225520842	0.1071	93.33%	0.749500	0.743100	0.02700000	0.01048000	NM_003607	intron	0	Hs.35433	CDC42BPA
rs2001486	17	9705256	0.4079	93.33%	0.374000	0.143700	0.02701000	0.02665000	NM_004246	intron	0	Hs.248202	GLP2R
rs159863	1	80188802	0.0223	94.39%	0.117800	1.000000	0.02702000	0.33240000	ENST00000294631	upstream	943852	---	---
rs6987004	8	34643545	0.2318	96.14%	0.040540	0.356500	0.02703000	0.09218000	NM_080872	upstream	877907	Hs.238889	UNC5D
rs1013186	9	100924158	0.1870	94.74%	0.316400	0.261800	0.02704000	0.01900000	NM_004612	intron	0	Hs.494622	TGFBR1
rs1367590	7	136630389	0.4946	97.54%	0.093960	0.291500	0.02705000	0.03636000	NM_002825	intron	0	Hs.371249	PTN
rs10496656	2	126830142	0.0735	97.89%	0.648800	0.646300	0.02707000	0.09995000	NM_016815	upstream	334402	Hs.59138	GYPC
rs993735	2	83617367	0.4803	97.89%	0.401600	0.649900	0.02708000	0.03852000	ENST00000362425	upstream	121003	---	---
rs1500150	5	85120228	0.2749	95.09%	0.760800	0.722300	0.02709000	0.16040000	ENST00000333189	downstream	238704	---	---
rs10503152	8	795383	0.0363	91.93%	1.000000	1.000000	0.02711000	0.06544000	NM_207332	upstream	124157	Hs.655310	ERICH1
rs1840574	11	106419826	0.1925	92.98%	0.427400	0.413900	0.02711000	0.00303700	NM_000855	upstream	25445	Hs.654555	GUCY1A2
rs10495174	1	219889526	0.3584	97.89%	0.297700	0.494600	0.02712000	0.11120000	NM_144729	downstream	51863	Hs.497822	DUSP10
rs10516930	4	94776709	0.3157	96.14%	0.162800	0.577300	0.02712000	0.00617500	NM_001510	intron	0	Hs.480281	GRID2
rs6846560	4	131850456	0.4489	92.63%	0.804500	1.000000	0.02713000	0.03932000	ENST00000363026	upstream	203076	---	---
rs10502020	11	103220691	0.4132	92.98%	0.057020	0.195300	0.02715000	0.00619900	NM_033135	downstream	62440	Hs.352298	PDGFD
rs10508992	10	54669439	0.3464	93.68%	0.590500	1.000000	0.02719000	0.07383000	ENST00000387222	upstream	218515	---	---
rs1519918	2	34631981	0.0870	96.84%	1.000000	1.000000	0.02720000	0.06102000	ENST00000322472	upstream	825635	---	---
rs1899728	15	50933835	0.1326	97.89%	0.293400	0.152500	0.02720000	0.02848000	NM_004498	upstream	64334	Hs.658573	ONECUT1
rs3745091	18	5945083	0.1082	94.04%	0.527500	1.000000	0.02720000	0.04252000	ENST00000317931	3UTR	0	---	---
rs10509947	10	113462163	0.1654	94.39%	0.505500	1.000000	0.02721000	0.01885000	NM_020918	downstream	437451	Hs.42586	GPAM
rs10511296	3	112680250	0.1344	97.89%	0.303100	0.165200	0.02722000	0.01167000	NM_005816	upstream	63296	Hs.142023	CD96
rs2830244	21	26769135	0.1344	97.89%	0.438900	0.576700	0.02722000	0.01171000	NM_052954	intron	0	Hs.37445	CYYR1
rs9294266	6	83666784	0.2100	94.39%	0.461500	0.301800	0.02724000	0.00531300	NM_198920	intron	0	Hs.148609	UBE2CBP
rs1011170	6	91386931	0.3339	95.09%	0.891300	0.858400	0.02725000	0.13150000	NM_145333	upstream	33303	Hs.644143	MAP3K7
rs2142333	8	116728632	0.4867	92.28%	0.711300	1.000000	0.02725000	0.16390000	NM_014112	intron	0	Hs.657018	TRPS1
rs7946495	11	7286469	0.2302	97.54%	0.615800	0.814300	0.02725000	0.06561000	NM_175733	intron	0	Hs.177193	SYT9
rs2291959	16	68431493	0.2626	97.54%	0.357200	0.857100	0.02726000	0.10680000	NM_199423	intron	0	Hs.408458	WWP2
rs10484888	6	10945240	0.1070	95.09%	0.099430	1.000000	0.02729000	0.02752000	NM_005906	intron	0	Hs.446125	MAK
rs7849676	9	8015789	0.3046	91.58%	0.883700	0.865600	0.02729000	0.04879000	NM_033428	upstream	225722	Hs.7517	C9orf123
rs10508670	10	24499009	0.2454	95.09%	0.622200	0.851600	0.02731000	0.05960000	ENST00000376462	intron	0	---	---
rs10513441	3	154522958	0.4624	97.89%	0.719500	0.288600	0.02731000	0.10900000	ENST00000383956	upstream	122462	---	---
rs33423	5	71916292	0.2454	95.09%	0.411400	0.349500	0.02731000	0.00745300	NM_152625	upstream	77290	Hs.370303	ZNF366
rs4109855	2	34807591	0.3022	97.54%	1.000000	0.704100	0.02731000	0.02745000	ENST00000384525	upstream	742584	---	---
rs42775	5	71916270	0.2454	95.09%	0.411400	0.349500	0.02731000	0.00703600	NM_152625	upstream	77268	Hs.370303	ZNF366
rs3852286	7	140319903	0.2014	97.54%	0.851700	0.398500	0.02732000	0.05617000	NM_053035	downstream	32533	Hs.416207	MRPS33
rs957634	1	29171596	0.0632	97.19%	1.000000	0.297100	0.02732000	0.01730000	NM_004437	intron	0	Hs.175437	EPB41
rs10518118	4	74970842	0.3199	95.44%	0.577200	0.359000	0.02733000	0.05241000	NM_001511	downstream	2593	Hs.789	CXCL1
rs10491019	10	110097967	0.4096	95.09%	0.707100	0.763100	0.02735000	0.17110000	ENST00000388288	downstream	592854	---	---
rs6842938	4	96111151	0.0646	95.09%	1.000000	1.000000	0.02735000	0.01198000	NM_001203	intron	0	Hs.661426	BMPR1B
rs242326	21	27749561	0.1147	97.89%	0.146500	0.206300	0.02736000	0.00887700	ENST00000382877	upstream	103780	---	---

rs10518978	15	56543465	0.1852	94.74%	0.543500	0.652600	0.02737000	0.02517000	NM_000236	intron	0	Hs.654472	LIPC
rs721114	7	51781201	0.1852	94.74%	0.067300	0.176900	0.02737000	0.07342000	ENST00000388001	downstream	29081	---	---
rs8093632	18	64297451	0.0221	95.09%	1.000000	1.000000	0.02737000	0.03747000	NM_019022	downstream	194456	Hs.440534	TXNDC10
rs4747872	10	10714372	0.0904	95.09%	0.707600	1.000000	0.02738000	0.01974000	NM_001025077	upstream	533084	Hs.309288	CUGBP2
rs943855	9	89460147	0.4964	97.89%	0.472400	0.072420	0.02738000	0.36700000	NM_004938	intron	0	Hs.380277	DAPK1
rs580253	11	104405698	0.2472	94.39%	0.623400	1.000000	0.02740000	0.23110000	NM_033293	CDS	0	Hs.2490	CASP1
rs948435	18	19637770	0.1773	88.07%	0.082460	0.057310	0.02740000	0.02298000	NM_198129	intron	0	Hs.436367	LAMA3
rs2073042	6	4622959	0.2191	93.68%	0.720000	0.685100	0.02743000	0.08442000	NM_170752	upstream	28433	Hs.269092	CDYL
rs9294331	6	86019189	0.2444	94.74%	0.743600	0.850000	0.02743000	0.19970000	ENST00000385105	upstream	35758	---	---
rs1060373	10	62224506	0.1354	97.19%	1.000000	0.234300	0.02747000	0.03247000	NM_033379	3UTR	0	Hs.334562	CDC2
rs10485823	20	1367846	0.0382	91.93%	0.000163	0.000301	0.02748000	0.08018000	NM_182483	downstream	2965	Hs.12865	NSFL1C
rs10507778	13	69480993	0.0413	84.91%	0.335800	0.393100	0.02749000	0.06229000	NM_020866	intron	0	Hs.508201	KLHL1
rs2213951	14	30345865	0.2310	97.19%	0.734800	1.000000	0.02749000	0.08583000	NM_004086	upstream	68031	Hs.21016	COCH
rs973090	1	216228890	0.0823	55.44%	0.000000	0.000000	0.02751000	0.58350000	NM_138796	downstream	117229	Hs.171130	SPATA17
rs10510492	3	19767401	0.2119	94.39%	0.000037	0.000532	0.02752000	0.02040000	NM_144715	downstream	128571	Hs.670883	EFHB
rs10491440	5	58924962	0.1477	92.63%	0.323300	0.299000	0.02754000	0.14350000	ENST00000340635	intron	0	---	---
rs1486767	4	30325563	0.3272	86.32%	0.310700	0.343100	0.02754000	0.06038000	NM_032457	upstream	6580	Hs.570785	PCDH7
rs1416851	10	84325671	0.3692	83.16%	0.328800	0.720400	0.02757000	0.01241000	NM_001010848	intron	0	Hs.125119	NRG3
rs6687999	1	212975718	0.1653	84.91%	0.008426	0.313000	0.02757000	0.04199000	NM_016343	downstream	71183	Hs.497741	CENPF
rs10515373	1	60474312	0.2088	95.79%	1.000000	1.000000	0.02758000	0.09392000	NM_152377	upstream	162282	Hs.47385	C1orf87
rs8111998	19	22533515	0.1196	96.84%	0.247800	0.198700	0.02758000	0.03997000	ENST00000384667	downstream	3506	---	---
rs10492498	13	109648524	0.3180	95.44%	0.001289	0.013970	0.02759000	0.04110000	NM_001845	intron	0	Hs.17441	COL4A1
rs10504845	8	89414303	0.2022	97.19%	0.851900	0.832700	0.02759000	0.04094000	NM_022564	upstream	5411	Hs.546267	MMP16
rs1448907	2	138002461	0.1654	92.28%	0.259900	0.211200	0.02760000	0.11620000	ENST00000272643	intron	0	---	---
rs10501751	11	90475764	0.1078	94.39%	1.000000	1.000000	0.02761000	0.03711000	NM_012124	upstream	879937	Hs.22857	CHORDC1
rs173969	5	40877713	0.3401	94.39%	0.787300	0.747600	0.02761000	0.00990900	NM_032587	intron	0	Hs.200242	CARD6
rs832994	7	136489930	0.0370	94.74%	1.000000	1.000000	0.02761000	0.01381000	NM_002825	downstream	72707	Hs.371249	PTN
rs2019576	11	98988128	0.2907	94.74%	0.883200	0.559300	0.02762000	0.02642000	NM_175566	upstream	207345	Hs.656783	CNTN5
rs4773773	13	93509501	0.3370	94.74%	0.685300	0.749000	0.02762000	0.03963000	NM_005708	intron	0	Hs.444329	GPC6
rs1924605	13	32253337	0.2727	96.49%	0.048530	0.300800	0.02764000	0.17900000	NM_015032	downstream	3180	Hs.699308	PDS5B
rs10504868	8	89993930	0.2058	97.19%	1.000000	0.836700	0.02765000	0.05412000	NM_003821	upstream	845243	Hs.103755	RIPK2
rs1582165	1	246668012	0.0984	87.37%	0.070790	0.704700	0.02766000	0.10780000	NM_001004136	upstream	14710	Hs.554482	OR2T2
rs3996993	6	52833480	0.3669	97.54%	0.796400	0.877000	0.02766000	0.02313000	NM_153699	upstream	14628	Hs.553652	GSTA5
rs10486120	7	11293764	0.0143	97.89%	1.000000	1.000000	0.02770000	0.02071000	NM_014660	downstream	117997	Hs.655688	PHF14
rs7172518	15	44973231	0.3967	95.09%	0.203800	0.444700	0.02772000	0.03305000	ENST00000388020	upstream	173230	---	---
rs10514397	16	74602921	0.4875	97.89%	0.401700	1.000000	0.02773000	0.04987000	ENST00000362429	upstream	150365	---	---
rs1512801	8	5703362	0.0222	94.74%	1.000000	1.000000	0.02773000	0.04039000	ENST00000363751	downstream	52484	---	---
rs2961857	5	165880413	0.2172	93.68%	0.470700	1.000000	0.02773000	0.01842000	ENST00000320147	downstream	138226	---	---
rs7326191	13	48737980	0.2878	95.09%	0.882400	0.845600	0.02773000	0.07998000	NM_030911	intron	0	Hs.388220	CDADC1
rs871976	16	74603719	0.4875	97.89%	0.401700	1.000000	0.02773000	0.04987000	ENST00000362429	upstream	151163	---	---
rs4935636	10	57502313	0.4248	93.33%	0.258500	0.220200	0.02774000	0.08724000	NM_001005414	downstream	284892	---	ZWINT
rs1106684	7	131104205	0.0709	89.12%	0.000496	0.350300	0.02775000	0.06356000	ENST00000305171	upstream	14885	---	---
rs1151380	13	100553186	0.1800	96.49%	1.000000	0.564000	0.02775000	0.03001000	NM_052867	intron	0	Hs.525146	NALCN
rs1918462	3	22132614	0.2677	89.12%	1.000000	0.514000	0.02775000	0.02073000	NM_024697	upstream	364794	Hs.21026	ZNF385D
rs1607429	2	57476744	0.1139	90.88%	0.031630	0.141800	0.02777000	0.00708600	NM_006296	upstream	650480	Hs.631890	VRK2
rs335200	5	122538028	0.0369	95.09%	0.304900	1.000000	0.02778000	0.10610000	ENST00000261364	intron	0	---	---
rs3765023	2	163336511	0.2624	92.28%	0.205700	0.144200	0.02779000	0.19340000	NM_173162	intron	0	Hs.657413	KCNH7
rs554473	6	25164660	0.0145	96.84%	1.000000	1.000000	0.02780000	0.02542000	ENST00000377993	downstream	24614	---	---
rs10488337	7	131128084	0.0764	96.49%	0.662300	0.661400	0.02781000	0.10240000	ENST00000305171	upstream	38764	---	---
rs1147963	10	44292854	0.2932	93.33%	0.038220	0.428000	0.02782000	0.06853000	NM_001033886	upstream	92306	Hs.522891	CXCL12
rs2512186	11	123634557	0.4302	92.98%	0.380100	0.542700	0.02783000	0.04253000	NM_001005198	intron	0	Hs.626840	OR8G5
rs7791639	7	21766724	0.0947	92.63%	0.018260	0.055330	0.02783000	0.02407000	NM_003777	intron	0	Hs.655326	DNAH11
rs314014	5	3116045	0.2158	84.56%	0.001842	0.175200	0.02784000	0.06365000	NM_178569	downstream	307534	Hs.668017	C5orf38
rs7981822	13	30586396	0.2373	89.47%	0.603300	0.237900	0.02785000	0.06300000	NM_006644	downstream	22369	Hs.36927	HSPH1

rs10258427	7	135839268	0.2292	88.77%	0.480200	0.540600	0.02787000	0.05089000	NM_001006629	upstream	364688	Hs.535891	CHRM2
rs542892	11	84468686	0.3483	93.68%	0.686200	0.602300	0.02787000	0.13410000	ENST00000376104	intron	0	---	---
rs2725225	4	89171100	0.4792	92.63%	0.391100	0.223200	0.02792000	0.08522000	NM_000297	intron	0	Hs.181272	PKD2
rs10492403	13	32641090	0.0689	89.12%	0.021450	0.045770	0.02793000	0.03041000	NM_178008	intron	0	---	STARD13
rs1327307	13	26600230	0.1444	94.74%	0.806500	1.000000	0.02797000	0.00623600	NM_182488	intron	0	Hs.42400	USP12
rs10499008	6	97445369	0.2585	92.98%	0.198400	0.195800	0.02799000	0.09508000	NM_014165	3UTR	0	Hs.591333	C6orf66
rs10514861	5	58833672	0.1241	94.74%	0.268600	0.544000	0.02799000	0.05858000	ENST00000340635	intron	0	---	---
rs4770548	13	23487379	0.0165	84.91%	0.057070	1.000000	0.02799000	0.16580000	NM_153023	upstream	145508	Hs.657121	SPATA13
rs722575	5	41033992	0.3082	97.89%	0.327300	1.000000	0.02801000	0.10910000	NM_173489	CDS	0	Hs.97714	FLJ40243
rs1371231	4	178378630	0.3687	97.54%	0.197300	0.215200	0.02804000	0.08159000	NM_018248	upstream	89392	Hs.405467	NEIL3
rs10496717	2	134548190	0.0426	82.46%	0.055760	1.000000	0.02805000	0.03048000	ENST00000385574	upstream	157047	---	---
rs9284859	3	26883268	0.1896	94.39%	1.000000	0.782800	0.02805000	0.18150000	NM_052953	downstream	156002	Hs.517868	LRRC3B
rs10521313	16	53078197	0.3759	97.54%	0.898300	0.759500	0.02808000	0.01059000	NM_024336	upstream	200318	Hs.499205	IRX3
rs442873	15	32431935	0.1124	90.53%	0.219300	1.000000	0.02808000	0.00690900	NM_175741	intron	0	Hs.525769	C15orf55
rs7686717	4	179246639	0.1472	92.98%	0.136500	0.474500	0.02811000	0.35050000	ENST00000362450	downstream	28217	---	---
rs266170	2	107413976	0.2338	97.54%	0.614800	0.647000	0.02812000	0.16800000	NM_032528	upstream	544934	Hs.98265	ST6GAL2
rs10497341	2	169036241	0.1264	94.39%	0.780400	0.770200	0.02813000	0.05035000	NM_203463	intron	0	Hs.506829	LASS6
rs9323037	14	40501442	0.4811	92.63%	0.326800	0.539400	0.02813000	0.01259000	NM_152447	upstream	645081	Hs.136893	LRFN5
rs540128	18	58695331	0.4733	91.93%	0.456900	0.442700	0.02814000	0.07117000	NM_194449	intron	0	Hs.465337	PHLPP
rs536412	11	74393553	0.2462	92.63%	0.047600	0.018760	0.02816000	0.00438100	NM_006656	intron	0	Hs.191074	NEU3
rs7156012	14	82440783	0.4073	81.40%	0.586500	0.329400	0.02816000	0.02608000	ENST00000362427	upstream	143892	---	---
rs10494639	1	188954081	0.4547	92.98%	0.025560	0.041000	0.02817000	0.06117000	NM_199051	upstream	240699	Hs.65765	FAM5C
rs2425094	20	33770388	0.1372	97.19%	0.616100	0.243600	0.02817000	0.01408000	NM_184241	intron	0	---	RBM39
rs6124036	20	58950874	0.2932	93.33%	0.183300	0.169100	0.02817000	0.00594700	NM_001794	upstream	310080	Hs.473231	CDH4
rs1409809	1	182456492	0.3182	96.49%	0.012220	0.197300	0.02819000	0.06701000	ENST00000362970	upstream	48912	---	---
rs714028	22	44303830	0.0977	89.82%	0.717600	1.000000	0.02819000	0.03522000	NM_006486	intron	0	Hs.24601	FBLN1
rs2328412	20	19495270	0.3782	96.49%	0.898000	0.738200	0.02821000	0.06494000	NM_020689	intron	0	Hs.654790	SLC24A3
rs7675107	4	77256806	0.1812	96.84%	0.418600	0.648000	0.02821000	0.01351000	NM_017426	intron	0	Hs.430435	NUP54
rs876050	7	30085935	0.2185	94.74%	0.212200	0.046640	0.02822000	0.03529000	NM_015899	intron	0	Hs.558495	PLEKHA9
rs9320379	6	112291314	0.2185	94.74%	1.000000	1.000000	0.02822000	0.03759000	NM_153048	intron	0	Hs.390567	FYN
rs459530	5	122524475	0.1028	87.02%	0.153600	0.262600	0.02825000	0.11690000	ENST00000261364	intron	0	---	---
rs10518722	15	51573373	0.2058	97.19%	0.267800	0.797900	0.02829000	0.05864000	NM_182758	downstream	21300	Hs.122125	WDR72
rs2167390	2	79455249	0.1421	95.09%	0.452600	0.714900	0.02829000	0.18010000	NM_138937	upstream	214862	Hs.567312	REG3A
rs10510760	3	52625388	0.4403	94.04%	0.385700	0.225800	0.02833000	0.10540000	NM_181042	intron	0	Hs.189920	PBRM1
rs10513239	3	145210016	0.1559	97.89%	0.359100	1.000000	0.02833000	0.03783000	NM_173552	downstream	16123	Hs.288954	C3orf58
rs4842779	12	90484944	0.3340	94.04%	0.783800	0.869400	0.02833000	0.04539000	NM_133507	upstream	384007	Hs.156316	DCN
rs410629	19	52162702	0.4608	94.04%	1.000000	0.644600	0.02834000	0.02310000	NM_004491	intron	0	Hs.509447	GRLF1
rs4132192	18	30000140	0.3869	96.14%	0.127600	0.250400	0.02834000	0.24680000	NM_003787	intron	0	Hs.514795	NOL4
rs233723	12	111515824	0.2717	96.84%	0.094160	0.300800	0.02835000	0.04894000	NM_002834	downstream	83725	Hs.506852	PTPN11
rs10504391	8	66795663	0.0433	97.19%	0.082870	1.000000	0.02837000	0.01418000	NM_002603	intron	0	Hs.584788	PDE7A
rs2417968	12	21263396	0.4678	92.63%	1.000000	0.760300	0.02838000	0.06269000	NM_006446	intron	0	Hs.449738	SLCO1B1
rs1018617	13	104839981	0.2981	92.98%	1.000000	0.863800	0.02840000	0.00954400	NM_172370	upstream	76612	Hs.381382	DAOA
rs10488598	7	136238383	0.2745	82.46%	0.072440	0.038560	0.02840000	0.01310000	NM_001006629	intron	0	Hs.535891	CHRM2
rs1222512	12	89297974	0.4244	95.09%	0.901300	0.430400	0.02842000	0.03146000	ENST00000359455	upstream	558590	---	---
rs2961223	3	105757056	0.1698	91.93%	1.000000	1.000000	0.02843000	0.11680000	ENST00000388649	downstream	255396	---	---
rs1998276	11	34266095	0.3199	91.58%	0.086990	0.095380	0.02847000	0.01834000	NM_145804	intron	0	Hs.23361	ABTB2
rs360376	2	126836257	0.0808	93.33%	0.682100	0.682700	0.02850000	0.11390000	NM_016815	upstream	328287	Hs.59138	GYPC
rs483330	1	49364564	0.3494	94.39%	0.893600	0.606000	0.02850000	0.10030000	ENST00000334103	intron	0	---	---
rs2038621	9	100689774	0.1468	94.39%	0.473400	1.000000	0.02851000	0.00849800	NM_024642	downstream	37590	Hs.47099	GALNT12
rs4146372	12	70417887	0.3000	92.98%	0.884100	1.000000	0.02851000	0.04881000	NM_014999	upstream	17038	Hs.524590	RAB21
rs1003069	9	16630702	0.3687	90.88%	0.595600	0.337300	0.02853000	0.15590000	NM_017637	intron	0	Hs.656581	BNC2
rs721358	2	224075200	0.2688	97.89%	0.878800	1.000000	0.02855000	0.05233000	NM_003469	downstream	94706	Hs.516726	SCG2
rs1892482	1	240840701	0.0556	94.74%	0.573700	1.000000	0.02856000	0.15520000	NM_152666	upstream	86080	Hs.672452	PLD5
rs963850	2	115859998	0.2897	95.09%	0.767600	0.434000	0.02856000	0.20440000	NM_001004360	intron	0	Hs.591555	DPP10

rs10514613	16	84688032	0.2823	95.09%	0.456300	1.000000	0.02861000	0.14640000	ENST00000326395	downstream	60506	---	---
rs1293288	8	11755937	0.4943	92.28%	0.387800	0.066080	0.02861000	0.01246000	NM_147783	intron	0	Hs.520898	CTSB
rs828722	5	162240294	0.1711	93.33%	0.029460	0.060480	0.02861000	0.00957000	NM_199246	upstream	556861	Hs.79101	CCNG1
rs10510620	3	29305290	0.1692	93.33%	0.828200	0.475200	0.02862000	0.13040000	NM_001003792	intron	0	Hs.696468	RBMS3
rs2314739	3	1573861	0.2547	92.98%	0.626800	0.828100	0.02862000	0.03661000	NM_014461	downstream	153583	Hs.387300	CNTN6
rs3769194	2	173691231	0.1774	97.89%	0.215400	0.489500	0.02862000	0.16570000	NM_133646	intron	0	Hs.444451	ZAK
rs4789523	17	73524449	0.3873	96.49%	1.000000	1.000000	0.02862000	0.03237000	NM_018996	upstream	32140	Hs.584945	TNRC6C
rs8005131	14	32660856	0.4981	93.33%	0.463400	0.169400	0.02863000	0.18230000	NM_022123	intron	0	Hs.659456	NPAS3
rs9283795	5	79118103	0.1417	84.21%	0.434300	0.252200	0.02864000	0.00209000	NM_153610	intron	0	Hs.482625	CMYA5
rs9316173	13	45365265	0.1096	91.23%	0.332600	0.614100	0.02864000	0.29870000	NM_015070	downstream	69050	Hs.136102	ZC3H13
rs10496879	2	141835904	0.0299	94.04%	1.000000	1.000000	0.02865000	0.04583000	NM_018557	intron	0	Hs.656461	LRP1B
rs1370361	2	55416524	0.2417	95.09%	0.621200	0.822400	0.02865000	0.03637000	NM_018084	intron	0	Hs.292925	CCDC88A
rs1192372	2	84865057	0.2463	94.74%	0.013950	0.013340	0.02866000	0.14500000	ENST00000389394	intron	0	---	---
rs10261822	7	50282713	0.2099	96.14%	0.016570	0.099250	0.02867000	0.12480000	NM_006060	upstream	32211	Hs.488251	IKZF1
rs26521	5	102510633	0.2862	94.39%	0.552300	0.108300	0.02868000	0.08498000	NM_015216	intron	0	Hs.212046	HISPPD1
rs737982	7	156099985	0.1817	97.54%	0.543500	0.772100	0.02868000	0.01193000	NM_032625	downstream	24981	---	C7orf13
rs921145	8	84487829	0.1187	97.54%	0.562000	1.000000	0.02868000	0.02696000	NM_173848	upstream	1116283	Hs.121663	RALYL
rs9322965	14	20862651	0.1187	97.54%	0.562000	0.661400	0.02868000	0.03927000	NM_020366	CDS	0	Hs.126035	RPGRIP1
rs1539530	1	48747520	0.3242	95.79%	0.130500	1.000000	0.02869000	0.04769000	NM_019073	upstream	37088	Hs.538103	SPATA6
rs2048873	2	113139305	0.2663	91.58%	1.000000	0.833200	0.02869000	0.03062000	NM_005415	downstream	1438	Hs.187946	SLC20A1
rs1391130	12	58270954	0.1954	91.58%	0.693800	0.824100	0.02872000	0.01262000	NM_004731	upstream	5161	Hs.439643	SLC16A7
rs961313	2	170641763	0.3984	89.82%	0.433600	0.741800	0.02874000	0.08603000	NM_172070	intron	0	---	UBR3
rs891848	15	64485226	0.2151	95.44%	0.591900	0.683400	0.02875000	0.05765000	NM_002755	intron	0	Hs.145442	MAP2K1
rs1869273	6	141565264	0.0465	94.39%	0.442600	1.000000	0.02877000	0.00400800	NM_006079	upstream	1827786	Hs.82071	CITED2
rs1608488	7	16936616	0.3269	82.11%	0.236500	0.222200	0.02878000	0.04024000	NM_176813	upstream	48478	Hs.100686	AGR3
rs7938204	11	78059326	0.4442	88.07%	0.015430	0.019810	0.02881000	0.01022000	ENST00000278550	intron	0	---	---
rs10521090	9	110659840	0.2548	92.28%	1.000000	0.574200	0.02882000	0.02139000	NM_006686	upstream	772	Hs.534390	ACTL7B
rs728472	14	79548254	0.1823	97.19%	0.108100	0.260600	0.02884000	0.02463000	NM_138970	downstream	147743	Hs.368307	NRXN3
rs2395182	6	32521295	0.2043	97.89%	0.361400	0.441000	0.02885000	0.04314000	NM_019111	downstream	352	Hs.520048	HLA-DRA
rs7701735	5	85719306	0.2076	97.19%	0.855700	0.798800	0.02885000	0.00756800	NM_001867	upstream	230171	Hs.430075	COX7C
rs10507414	13	33195843	0.0303	92.63%	1.000000	1.000000	0.02889000	0.01221000	NM_181558	upstream	94391	Hs.115474	RFC3
rs9300654	13	100624895	0.2623	92.98%	1.000000	0.360600	0.02889000	0.11170000	NM_052867	intron	0	Hs.525146	NALCN
rs1909192	1	220813609	0.1120	90.88%	0.026210	0.085210	0.02890000	0.47890000	NM_139352	intron	0	Hs.153088	TAF1A
rs541164	18	10031354	0.4739	94.04%	0.713100	0.293700	0.02890000	0.04762000	NM_194434	downstream	81783	Hs.699980	VAPA
rs1368798	15	37015026	0.2762	97.19%	0.880400	1.000000	0.02892000	0.06408000	NM_207444	downstream	235495	Hs.448785	C15orf53
rs2877551	4	71298176	0.4920	87.37%	0.526100	0.758100	0.02894000	0.07098000	NM_021225	upstream	70	Hs.661425	PROL1
rs874205	8	124899398	0.0973	90.18%	0.073630	1.000000	0.02894000	0.05058000	NM_144963	downstream	5138	Hs.459174	FAM91A1
rs1477901	4	18182414	0.3195	97.19%	0.582300	1.000000	0.02896000	0.02033000	NM_153686	upstream	549817	Hs.446201	LCORL
rs1486466	8	91955434	0.4684	94.39%	0.222500	0.228700	0.02896000	0.09470000	NM_022351	intron	0	Hs.560892	NECAB1
rs1588076	12	62209455	0.2402	80.35%	0.203000	0.147400	0.02896000	0.02497000	NM_173812	downstream	29505	Hs.533644	DPY19L2
rs2295884	6	39976014	0.0500	73.68%	0.409000	0.123400	0.02896000	0.19920000	NM_015345	intron	0	Hs.652207	DAAM2
rs10499722	7	53896575	0.1959	94.04%	0.845800	1.000000	0.02897000	0.00761000	NM_001001707	upstream	49465	---	FLJ45974
rs718262	8	103045180	0.3574	92.28%	0.893900	0.600600	0.02899000	0.07376000	NM_032041	intron	0	Hs.492427	NCALD
rs2191508	2	195375221	0.1029	97.19%	1.000000	0.735200	0.02900000	0.04873000	ENST00000364989	upstream	138078	---	---
rs1378753	2	138268400	0.2418	95.79%	0.048070	0.171300	0.02903000	0.01467000	NM_001024075	upstream	169878	Hs.42151	HNMT
rs10487523	7	144482284	0.2091	96.49%	0.361200	0.298700	0.02906000	0.01174000	ENST00000363803	upstream	304174	---	---
rs10488917	4	112142061	0.0461	95.09%	0.440100	1.000000	0.02907000	0.03169000	NM_000325	upstream	378358	Hs.643588	PITX2
rs10506556	12	66195833	0.0846	91.23%	0.701500	1.000000	0.02907000	0.05608000	NM_006482	upstream	133188	Hs.173135	DYRK2
rs10516257	4	12253560	0.4035	90.88%	0.072100	0.145400	0.02908000	0.03447000	ENST00000387175	downstream	330148	---	---
rs6821318	4	20804115	0.4407	94.74%	1.000000	1.000000	0.02910000	0.11210000	NM_001035004	intron	0	Hs.655705	KCNIP4
rs7728402	5	174361854	0.4524	81.05%	0.000000	0.000000	0.02910000	0.16120000	NM_001004348	upstream	6531	---	FLJ16171
rs2377426	9	77736209	0.2712	91.23%	1.000000	0.835600	0.02911000	0.04060000	NM_006200	intron	0	Hs.368542	PCSK5
rs6969997	7	31278995	0.1661	95.09%	0.509800	1.000000	0.02911000	0.12240000	NM_022728	downstream	64612	Hs.45152	NEUROD6
rs10490814	3	45192647	0.1191	97.19%	0.000360	0.001747	0.02913000	0.10520000	NM_178181	upstream	29729	Hs.476093	CDCP1

rs2826129	21	20589180	0.4310	83.86%	0.064780	0.122900	0.02914000	0.01941000	ENST00000387009	downstream	60751	---	---
rs6696791	1	225311107	0.1062	95.79%	0.098340	0.169200	0.02914000	0.01050000	NM_003607	intron	0	Hs.35433	CDC42BPA
rs237112	6	79451719	0.3911	90.18%	0.049170	0.156500	0.02915000	0.03931000	NM_001010844	upstream	182189	Hs.656212	IRAK1BP1
rs1065024	11	15945926	0.2247	93.68%	0.380600	0.147400	0.02916000	0.16210000	NM_033326	downstream	2445	Hs.368226	SOX6
rs2140218	12	70906018	0.0297	94.39%	0.017490	0.042370	0.02916000	0.19500000	NM_001025457	downstream	27542	---	LOC283392
rs2340768	8	105222962	0.1581	88.77%	0.000732	0.002930	0.02916000	0.10840000	NM_014677	intron	0	Hs.655271	RIMS2
rs10496416	2	107699969	0.0331	84.91%	0.000024	0.047160	0.02920000	0.05275000	NM_032528	upstream	830927	Hs.98265	ST6GAL2
rs2145535	6	97141436	0.2546	94.39%	0.422400	0.664400	0.02921000	0.00509500	NM_020482	intron	0	Hs.632608	FHL5
rs866651	3	24237159	0.3201	97.54%	0.492100	0.364300	0.02921000	0.01148000	NM_000461	intron	0	Hs.187861	THRB
rs787116	10	29074879	0.1400	96.49%	0.129300	0.173600	0.02922000	0.11980000	NM_012342	downstream	63005	Hs.533336	BAMBI
rs7243011	18	1600953	0.1328	89.82%	1.000000	1.000000	0.02923000	0.01527000	NM_022840	downstream	926577	Hs.126888	METTL4
rs9314565	8	5217548	0.4963	93.68%	0.902900	0.647600	0.02923000	0.15890000	ENST00000386510	upstream	229911	---	---
rs2146959	6	9061356	0.3266	96.14%	0.215800	0.511100	0.02929000	0.07739000	NM_015948	upstream	680641	Hs.285847	SLC35B3
rs10509321	10	71655739	0.2239	94.04%	0.860500	1.000000	0.02930000	0.10270000	NM_021129	intron	0	Hs.437403	PPA1
rs3798244	6	116383432	0.3261	88.77%	0.775100	0.312900	0.02930000	0.07607000	NM_002031	intron	0	Hs.89426	FRK
rs10520936	5	27619491	0.1047	97.19%	0.517400	0.489600	0.02931000	0.04387000	ENST00000384531	downstream	471953	---	---
rs1991126	2	21676503	0.0944	94.74%	0.486100	0.602900	0.02931000	0.01993000	ENST00000383859	upstream	322867	---	---
rs2963426	5	157914908	0.2963	94.74%	0.558900	0.497200	0.02931000	0.05433000	NM_024007	downstream	143789	Hs.657753	EBF1
rs6696981	1	22575445	0.0944	94.74%	0.071580	0.052350	0.02931000	0.01668000	NM_014870	upstream	75492	Hs.418966	ZBTB40
rs3771712	2	159960814	0.2772	96.84%	0.766100	0.860600	0.02932000	0.08696000	NM_013450	intron	0	Hs.470369	BAZ2B
rs10498260	2	232764865	0.1489	91.93%	0.048890	0.142300	0.02935000	0.05728000	NM_152383	intron	0	Hs.471637	DIS3L2
rs296171	5	152584259	0.1227	95.79%	0.394600	0.224600	0.02936000	0.00961200	NM_000827	upstream	266240	Hs.519693	GRIA1
rs6784979	3	153836590	0.4887	93.33%	0.271400	0.648800	0.02936000	0.03616000	NM_207297	downstream	170332	Hs.478000	MBNL1
rs10488498	7	20934866	0.1385	97.54%	0.319400	0.579500	0.02938000	0.05646000	ENST00000325612	downstream	101166	---	---
rs2025259	14	53668865	0.1385	97.54%	0.203600	0.265400	0.02938000	0.04789000	NM_130851	upstream	175503	Hs.68879	BMP4
rs10516254	4	12243759	0.3609	93.33%	0.790200	0.395100	0.02943000	0.02592000	ENST00000387175	downstream	320347	---	---
rs10516275	4	13831732	0.4833	94.39%	0.142900	0.223600	0.02943000	0.13040000	NM_148894	upstream	593287	Hs.444517	FAM44A
rs10485494	20	5867378	0.0305	91.93%	0.210200	1.000000	0.02945000	0.06617000	NM_015939	intron	0	Hs.128791	TRMT6
rs4673993	2	215920584	0.3074	85.61%	0.369500	0.430200	0.02945000	0.02737000	NM_004044	intron	0	Hs.90280	ATIC
rs10497036	2	149052674	0.0579	90.88%	0.201800	1.000000	0.02947000	0.09138000	NM_018328	downstream	65162	Hs.458312	MBD5
rs748548	12	124387008	0.4204	94.74%	0.133500	0.758800	0.02947000	0.00893800	NM_023928	downstream	193194	Hs.656073	AACS
rs10513996	18	66895350	0.0560	94.04%	0.576600	0.235800	0.02949000	0.04987000	NM_182511	downstream	1459545	Hs.569851	CBLN2
rs16824479	2	184010520	0.0558	75.44%	0.019650	0.157600	0.02949000	0.04412000	NM_001008544	downstream	275870	---	NUP35
rs10505907	12	23806140	0.0554	95.09%	1.000000	1.000000	0.02950000	0.08278000	NM_178010	intron	0	Hs.657542	SOX5
rs10516210	4	10356961	0.0554	95.09%	0.572300	0.233400	0.02950000	0.04487000	NM_052964	upstream	61477	Hs.678910	MIST
rs2129260	8	91956201	0.4684	94.39%	0.222500	0.176800	0.02950000	0.09916000	NM_022351	intron	0	Hs.560892	NECAB1
rs2458437	12	69495876	0.1764	90.53%	0.016310	0.011090	0.02951000	0.16440000	NM_002849	intron	0	Hs.506076	PTPRR
rs10494649	1	189079912	0.4526	88.77%	0.005153	0.013830	0.02952000	0.04268000	NM_199051	upstream	366530	Hs.65765	FAM5C
rs2716454	11	24710115	0.2428	96.84%	0.250400	0.652600	0.02952000	0.00735800	NM_001009909	intron	0	Hs.144138	LUZP2
rs548635	5	131083445	0.2989	95.09%	0.191100	0.248500	0.02954000	0.02027000	NM_001008738	intron	0	Hs.591273	FNIP1
rs1940622	18	5945181	0.2565	95.09%	0.110400	0.026540	0.02955000	0.16800000	ENST00000317931	3UTR	0	---	---
rs1411025	10	110197196	0.0795	92.63%	0.000350	0.000923	0.02961000	0.03889000	ENST00000388288	downstream	493625	---	---
rs10495913	2	44197180	0.2770	97.54%	0.653100	0.726800	0.02963000	0.21690000	NM_133259	upstream	120590	Hs.368084	LRPPRC
rs6924188	6	155249827	0.4777	94.39%	1.000000	0.759800	0.02963000	0.18550000	NM_012454	intron	0	Hs.586279	TIAM2
rs7183692	15	59687544	0.1022	97.89%	0.182800	0.317300	0.02963000	0.16850000	NM_001018088	downstream	244340	Hs.511668	VPS13C
rs2010316	20	17077082	0.3787	83.86%	0.000000	0.000000	0.02964000	0.05097000	ENST00000384329	upstream	39073	---	---
rs720721	12	2538005	0.2722	90.88%	0.004452	0.003174	0.02966000	0.05295000	NM_000719	intron	0	Hs.118262	CACNA1C
rs10509771	10	106145540	0.0520	97.89%	0.535700	1.000000	0.02968000	0.21990000	NM_001008723	intron	0	Hs.253576	CCDC147
rs2367478	3	194498336	0.1093	94.74%	0.751900	1.000000	0.02968000	0.01942000	NM_198505	intron	0	Hs.658032	ATP13A5
rs1997833	20	39123756	0.3187	91.93%	0.775800	0.739700	0.02971000	0.00602800	NM_003286	intron	0	Hs.592136	TOP1
rs922904	4	53575902	0.2457	82.11%	0.294500	0.236200	0.02972000	0.00973100	NM_152540	intron	0	Hs.302287	SCFD2
rs9305269	21	26274819	0.3022	94.04%	0.562500	1.000000	0.02972000	0.00580500	NM_201414	intron	0	Hs.434980	APP
rs10492371	12	27637547	0.0465	94.39%	0.101300	0.148800	0.02974000	0.10360000	NM_177444	intron	0	Hs.172445	PPFIBP1
rs1532964	3	36835547	0.2401	97.89%	1.000000	0.650800	0.02975000	0.10260000	ENST00000389421	downstream	11816	---	---

rs4977880	9	23659526	0.2401	97.89%	0.249400	0.502400	0.02975000	0.03042000	NM_004432	downstream	22458	Hs.166109	ELAVL2
rs10504196	8	56609912	0.1413	96.84%	0.041990	0.258300	0.02977000	0.52030000	NM_052898	downstream	10572	Hs.130197	XKR4
rs1527307	7	141357368	0.3524	95.09%	0.424000	0.268100	0.02977000	0.04987000	NM_004668	intron	0	Hs.122785	MGAM
rs3744583	17	11597190	0.3268	90.18%	0.000595	0.007135	0.02977000	0.03492000	NM_001372	intron	0	Hs.567259	DNAH9
rs10500911	11	21568879	0.2135	96.14%	0.590000	1.000000	0.02979000	0.03986000	NM_006157	downstream	15076	Hs.657172	NELL1
rs10520083	5	129967905	0.4944	94.74%	0.331800	0.365300	0.02979000	0.05476000	NM_005340	downstream	554978	Hs.483305	HINT1
rs10435419	7	133980899	0.0547	96.14%	0.568000	0.600200	0.02983000	0.07082000	NM_199186	upstream	1196	Hs.198365	BPGM
rs10483353	14	30622371	0.0547	96.14%	0.568000	0.600200	0.02983000	0.09733000	NM_007077	intron	0	Hs.293411	AP4S1
rs6560496	9	77875690	0.0543	96.84%	1.000000	1.000000	0.02983000	0.01624000	NM_006200	intron	0	Hs.368542	PCSK5
rs9311101	3	35613665	0.3041	94.04%	0.386100	0.126100	0.02984000	0.05757000	NM_001025069	upstream	45188	Hs.475902	ARPP-21
rs10485661	20	37858285	0.3109	93.68%	0.001620	0.131400	0.02985000	0.14080000	NM_005461	downstream	889617	Hs.702085	MAFB
rs7138789	12	96314350	0.0540	97.54%	0.562500	0.595800	0.02985000	0.03044000	ENST00000384854	upstream	167371	---	---
rs844604	6	147966148	0.3285	96.14%	0.000927	0.008412	0.02985000	0.10150000	NM_001030060	downstream	33298	Hs.567973	SAMD5
rs660717	9	9559081	0.3166	90.88%	0.195800	0.307300	0.02986000	0.02476000	ENST00000363183	downstream	126729	---	---
rs724053	20	16933601	0.1398	97.89%	0.319000	1.000000	0.02988000	0.08291000	ENST00000387043	upstream	21923	---	---
rs1335296	6	141788521	0.0685	94.74%	1.000000	0.363800	0.02990000	0.01428000	NM_006079	upstream	2051043	Hs.82071	CITED2
rs8074693	17	65321997	0.1835	97.54%	1.000000	1.000000	0.02991000	0.20810000	NM_002758	downstream	270930	Hs.463978	MAP2K6
rs2877631	17	50218320	0.2670	92.63%	0.272500	0.469500	0.02994000	0.04754000	NM_005486	upstream	114883	Hs.153504	TOM1L1
rs462296	3	18596143	0.2985	94.04%	0.381600	0.845300	0.02995000	0.02856000	NM_002971	upstream	140935	Hs.517717	SATB1
rs6083460	20	2424631	0.3712	92.63%	0.429600	1.000000	0.02996000	0.03599000	NM_024325	intron	0	Hs.516846	ZNF343
rs6933879	6	22907679	0.1278	94.74%	0.407800	0.101000	0.02997000	0.06438000	ENST00000387548	upstream	108245	---	---
rs1497997	3	112666187	0.1210	88.42%	0.381200	0.343000	0.03002000	0.02451000	NM_005816	upstream	77359	Hs.142023	CD96
rs10509911	10	112009678	0.0162	97.54%	1.000000	1.000000	0.03003000	0.99790000	NM_001008541	intron	0	Hs.501023	MXI1
rs10514850	13	52649537	0.0162	97.54%	0.063640	0.098420	0.03003000	0.99770000	NM_006418	downstream	125350	Hs.559736	OLFM4
rs7552778	1	190709879	0.0162	97.54%	1.000000	1.000000	0.03003000	0.99780000	NM_002922	upstream	101601	Hs.75256	RGS1
rs10131076	14	79844138	0.1245	95.79%	0.779500	0.377100	0.03005000	0.02303000	NM_001007023	upstream	96534	Hs.202354	DIO2
rs2288383	5	168947791	0.0163	96.84%	1.000000	1.000000	0.03005000	0.99790000	NM_017785	intron	0	Hs.368710	CCDC99
rs6818281	4	164709149	0.4717	92.98%	0.901900	1.000000	0.03006000	0.04973000	NM_017923	intron	0	Hs.696248	01.Mar
rs7662165	4	12420925	0.3903	97.54%	0.314100	0.166600	0.03006000	0.03092000	ENST00000387175	downstream	497513	---	---
rs1908638	1	150415882	0.0161	97.89%	0.063420	0.097890	0.03008000	0.99760000	ENST00000388718	intron	0	---	---
rs7286017	22	31091715	0.0161	97.89%	1.000000	1.000000	0.03008000	0.99800000	ENST00000382086	intron	0	---	---
rs967016	2	83548442	0.3907	97.89%	0.381300	0.283900	0.03009000	0.01955000	ENST00000362425	upstream	189928	---	---
rs3816013	5	146761633	0.3975	97.54%	0.133400	0.256600	0.03010000	0.27720000	NM_001387	intron	0	Hs.519659	DPYSL3
rs8091945	18	59680510	0.4109	90.53%	0.701000	0.623700	0.03012000	0.08569000	NM_002575	upstream	25412	Hs.594481	SERPINB2
rs1483822	8	24001297	0.0360	97.54%	0.040900	1.000000	0.03013000	0.12000000	NM_021777	upstream	206268	Hs.174030	ADAM28
rs719395	6	52398886	0.3297	96.84%	0.587200	0.415100	0.03013000	0.02537000	NM_018100	intron	0	Hs.403171	EFHC1
rs10516388	4	21086690	0.0712	91.23%	0.373700	0.376100	0.03014000	0.05409000	NM_025221	intron	0	Hs.655705	KCNIP4
rs4239162	17	43110809	0.4618	87.37%	0.160200	0.038980	0.03014000	0.03736000	NM_002265	intron	0	Hs.532793	KPNB1
rs10506435	12	60725172	0.1122	92.28%	0.029250	0.033050	0.03015000	0.03874000	NM_178539	upstream	21343	Hs.269745	FAM19A2
rs10520111	15	36896223	0.2437	97.19%	0.049080	0.076970	0.03015000	0.13720000	NM_207444	downstream	116692	Hs.448785	C15orf53
rs1337987	10	113528178	0.4272	94.04%	0.803200	0.758100	0.03015000	0.07312000	NM_020918	downstream	371436	Hs.42586	GPAM
rs10508244	10	3143203	0.1236	96.49%	1.000000	0.679600	0.03016000	0.12360000	NM_002627	intron	0	Hs.26010	PFKP
rs4363467	1	17459240	0.1220	87.72%	1.000000	0.367900	0.03018000	0.00644200	NM_016233	intron	0	Hs.149195	PADI3
rs10492507	13	48722291	0.2039	81.75%	0.161800	0.370300	0.03019000	0.08177000	NM_030911	intron	0	Hs.388220	CDADC1
rs2243385	6	117770192	0.1115	94.39%	1.000000	1.000000	0.03019000	0.08216000	NM_002944	intron	0	Hs.1041	ROS1
rs9285165	13	47591998	0.1488	84.91%	0.199900	0.098630	0.03019000	0.04613000	NM_014166	upstream	24757	Hs.181112	MED4
rs10516215	4	10487213	0.2603	93.68%	0.752000	0.673600	0.03021000	0.04954000	NM_052964	upstream	191729	Hs.678910	MIST
rs1079032	18	6554219	0.1474	94.04%	0.812000	0.727100	0.03021000	0.00614500	ENST00000386846	upstream	37320	---	---
rs3764703	20	37077073	0.4076	96.84%	0.708900	0.747600	0.03022000	0.01953000	NM_021931	intron	0	Hs.444520	DHX35
rs10502526	18	24852634	0.2889	94.74%	0.301800	0.159500	0.03023000	0.03521000	NM_001792	upstream	841445	Hs.464829	CDH2
rs3775442	4	90934254	0.1097	94.39%	1.000000	1.000000	0.03023000	0.01964000	NM_000345	intron	0	Hs.271771	SNCA
rs254487	5	52483969	0.1223	97.54%	0.153500	0.225500	0.03025000	0.02444000	NM_004531	upstream	42633	Hs.163645	MOCS2
rs9293254	5	26985148	0.0166	95.09%	0.001280	0.003061	0.03025000	0.99780000	NM_016279	intron	0	Hs.272212	CDH9
rs2393220	10	58501127	0.0171	92.28%	1.000000	1.000000	0.03026000	0.99790000	NM_001005414	upstream	710085	---	ZWINT

rs2397429	6	100263963	0.1860	87.72%	0.206600	0.768300	0.03026000	0.16260000	NM_032503	downstream	210544	Hs.591342	MCHR2
rs274460	12	58238608	0.1828	97.89%	0.546100	0.650800	0.03026000	0.01035000	ENST00000365601	downstream	28233	---	---
rs797002	6	119393692	0.0551	95.44%	1.000000	1.000000	0.03026000	0.09530000	NM_024581	intron	0	Hs.443789	C6orf60
rs1395771	3	97946270	0.2531	85.96%	0.174700	0.117200	0.03031000	0.07496000	ENST00000341636	upstream	32521	---	---
rs2372648	12	98085110	0.3978	97.89%	0.707600	0.746300	0.03031000	0.04648000	NM_020140	intron	0	Hs.506458	ANKS1B
rs10491403	5	146535383	0.1111	94.74%	1.000000	1.000000	0.03032000	0.12780000	NM_145001	upstream	59336	Hs.697853	STK32A
rs2362330	4	71900310	0.0899	97.54%	1.000000	1.000000	0.03032000	0.15920000	NM_002092	downstream	3804	Hs.309763	GRSF1
rs3024896	2	191604961	0.1111	94.74%	0.346200	0.516300	0.03032000	0.05695000	NM_003151	intron	0	Hs.80642	STAT4
rs6514066	20	43934201	0.3574	94.74%	0.791100	0.874700	0.03032000	0.15970000	NM_080752	intron	0	Hs.292135	ZSWIM3
rs10517903	4	167948429	0.2621	94.39%	0.083670	0.717900	0.03033000	0.02969000	NM_016950	intron	0	Hs.481133	SPOCK3
rs10491707	9	2343522	0.1685	93.68%	0.076520	0.109100	0.03034000	0.05217000	NM_003070	downstream	159898	Hs.298990	SMARCA2
rs10518874	15	34446099	0.3648	94.74%	0.148400	0.083640	0.03038000	0.01814000	NM_080650	upstream	820443	Hs.107196	ATPBD4
rs10510864	3	61180878	0.2115	97.89%	1.000000	1.000000	0.03039000	0.03932000	NM_002841	upstream	342124	Hs.654488	PTPRG
rs2622542	8	56479295	0.4163	81.75%	0.500900	0.745000	0.03040000	0.11670000	NM_052898	intron	0	Hs.130197	XKR4
rs10511001	3	70715884	0.2419	97.19%	0.070090	0.072260	0.03041000	0.04237000	NM_001013679	downstream	31111	---	---
rs340881	1	212215651	0.1227	97.19%	0.020420	0.225200	0.03041000	0.04949000	NM_002763	upstream	12832	Hs.585369	PROX1
rs939628	2	78718842	0.3788	92.63%	0.896100	0.875100	0.03044000	0.05173000	ENST00000325094	upstream	225301	---	---
rs10502581	18	27884067	0.0165	95.79%	1.000000	1.000000	0.03045000	0.99770000	NM_017831	intron	0	Hs.633703	RNF125
rs9293370	5	83694245	0.3281	89.82%	0.672300	0.500600	0.03046000	0.00613900	NM_005711	intron	0	Hs.482730	EDIL3
rs3851564	10	126937238	0.1132	92.98%	1.000000	1.000000	0.03047000	0.01573000	ENST00000316750	upstream	31426	---	---
rs1339593	1	191174886	0.1107	95.09%	0.345400	0.196400	0.03053000	0.01138000	NM_015984	downstream	76626	Hs.591458	UCHL5
rs9307014	4	87171711	0.1107	95.09%	0.220000	0.319300	0.03053000	0.11810000	NM_138981	intron	0	Hs.125503	MAPK10
rs2391704	7	28912352	0.0843	91.58%	0.231800	1.000000	0.03054000	0.13170000	NM_014817	downstream	49400	Hs.21572	KIAA0644
rs1424125	16	6002554	0.2520	89.12%	0.098540	0.127100	0.03055000	0.00821400	NM_001013705	upstream	365211	---	LOC440337
rs9319281	13	25680887	0.2738	92.28%	1.000000	0.533700	0.03055000	0.05209000	NM_183045	intron	0	---	RNF6
rs509946	6	52411949	0.3400	87.72%	0.090980	0.028990	0.03056000	0.02827000	NM_018100	intron	0	Hs.403171	EFHC1
rs1352968	4	167586459	0.0484	90.53%	0.108900	0.008503	0.03057000	0.04322000	NM_016950	downstream	304708	Hs.481133	SPOCK3
rs1827358	5	22599756	0.3462	95.79%	0.422000	0.218100	0.03058000	0.04929000	NM_004061	intron	0	Hs.113684	CDH12
rs2150696	9	12454876	0.3811	92.98%	0.896700	0.867200	0.03058000	0.02545000	NM_000550	upstream	228559	Hs.270279	TYRP1
rs2220390	1	152499640	0.3345	96.49%	0.786400	0.745200	0.03058000	0.00489200	NM_014847	intron	0	Hs.490551	UBAP2L
rs2325528	6	94568196	0.3993	97.54%	0.900500	0.517400	0.03060000	0.08712000	NM_004440	upstream	382203	Hs.73962	EPHA7
rs1854227	13	97036437	0.2446	97.54%	0.628900	0.574600	0.03061000	0.09839000	NM_021033	downstream	118194	Hs.508480	RAP2A
rs6859245	5	125204151	0.4963	94.04%	0.271200	0.171400	0.03061000	0.02133000	NM_023927	upstream	582849	Hs.363558	GRAMD3
rs10505299	8	118495921	0.1157	89.47%	0.546900	0.365300	0.03063000	0.02093000	NM_080651	upstream	106290	Hs.492612	MED30
rs10493244	1	58379054	0.1506	94.39%	1.000000	0.318900	0.03064000	0.08327000	NM_021080	intron	0	Hs.477370	DAB1
rs6604659	1	215459562	0.0690	94.04%	0.117600	0.062750	0.03064000	0.01871000	NM_206595	upstream	129992	Hs.444225	ESRRG
rs10502079	11	106224087	0.1756	91.93%	0.001104	0.003497	0.03065000	0.02118000	NM_000855	intron	0	Hs.654555	GUCY1A2
rs995830	3	69684474	0.1648	95.79%	0.024860	0.027700	0.03065000	0.21980000	NM_198178	upstream	211178	Hs.166017	MITF
rs10496837	2	140668940	0.3694	94.04%	0.008453	0.007510	0.03066000	0.04453000	ENST00000385554	upstream	22351	---	---
rs3861571	2	55435740	0.0172	91.58%	1.000000	1.000000	0.03066000	0.99800000	NM_018084	intron	0	Hs.292925	CCDC88A
rs10496121	2	64652930	0.0815	94.74%	0.690800	1.000000	0.03067000	0.02252000	NM_001002243	intron	0	Hs.655167	AFTPH
rs10501019	11	25686846	0.2097	97.89%	0.586800	0.613500	0.03067000	0.06454000	NM_031418	upstream	622701	Hs.91791	TMEM16C
rs724098	15	37560051	0.2097	97.89%	0.364700	0.312100	0.03067000	0.11210000	NM_003246	upstream	100521	Hs.164226	THBS1
rs10499653	7	47112349	0.1421	97.54%	0.621500	0.474800	0.03068000	0.10960000	NM_022748	downstream	168930	Hs.520814	TNS3
rs10506388	12	57021115	0.1387	89.82%	0.114400	0.395500	0.03068000	0.04983000	NM_153377	downstream	531090	Hs.253736	LRIG3
rs1327272	6	51336858	0.1421	97.54%	1.000000	1.000000	0.03068000	0.02776000	ENST00000320956	downstream	45980	---	---
rs10484242	14	43329259	0.1643	97.19%	0.662900	1.000000	0.03071000	0.04344000	NM_152447	downstream	1885761	Hs.136893	LRFN5
rs517425	18	4375309	0.0228	92.28%	0.005217	1.000000	0.03074000	0.03235000	ENST00000384548	upstream	490504	---	---
rs959702	10	2139260	0.2259	94.74%	1.000000	0.552700	0.03074000	0.04399000	NM_018702	upstream	369590	Hs.657984	ADARB2
rs486975	11	93709139	0.3699	94.39%	0.190600	0.209400	0.03075000	0.06747000	NM_016540	downstream	40992	Hs.272385	GPR83
rs7184633	16	81379514	0.4428	95.09%	0.902000	0.875300	0.03075000	0.02545000	NM_001257	upstream	69946	Hs.654386	CDH13
rs1414483	1	76520348	0.1216	89.47%	1.000000	1.000000	0.03076000	0.07896000	NM_152996	intron	0	Hs.337040	ST6GALNAC
rs10483513	14	39556095	0.1057	97.89%	0.526100	0.349400	0.03077000	0.10100000	NM_203301	upstream	584724	Hs.324342	FBXO33
rs1341734	9	15753124	0.3143	85.96%	0.377400	0.080340	0.03077000	0.10660000	NM_173550	intron	0	Hs.17267	C9orf93

rs1483821	8	24001333	0.0358	97.89%	0.040620	1.000000	0.03079000	0.12470000	NM_021777	upstream	206232	Hs.174030	ADAM28
rs2106970	12	111158740	0.1440	87.72%	0.067040	0.475600	0.03080000	0.04486000	ENST00000377563	intron	0	---	---
rs721912	3	172643128	0.2419	97.89%	0.624000	0.077350	0.03080000	0.20240000	NM_015028	intron	0	Hs.34024	TNIK
rs10488991	4	11186234	0.1476	95.09%	0.811600	0.301800	0.03083000	0.05884000	NM_005114	upstream	145747	Hs.507348	HS3ST1
rs7996544	13	45088338	0.1877	97.19%	0.112400	0.404900	0.03083000	0.06148000	NM_152719	upstream	86109	Hs.186363	SPERT
rs10486048	7	14210927	0.2316	85.61%	0.045950	0.023730	0.03085000	0.06788000	NM_145695	intron	0	Hs.567255	DGKB
rs261008	5	169271614	0.3333	96.84%	0.587600	0.872500	0.03086000	0.24840000	NM_004946	intron	0	Hs.586174	DOCK2
rs10483160	22	30407247	0.0168	94.04%	0.065970	0.100100	0.03087000	0.99780000	NM_173566	intron	0	Hs.438906	C22orf30
rs7150974	14	78958125	0.3273	97.54%	0.275500	0.048340	0.03087000	0.07618000	NM_138970	intron	0	Hs.368307	NRXN3
rs10493701	1	81919788	0.3074	94.74%	0.318800	0.240200	0.03091000	0.29210000	ENST00000370721	intron	0	---	---
rs2604532	11	101655698	0.3074	94.74%	0.775000	0.738400	0.03091000	0.04088000	NM_006106	downstream	46342	Hs.503692	YAP1
rs10493001	1	20973503	0.0948	94.39%	1.000000	0.602500	0.03092000	0.05055000	NM_016287	intron	0	Hs.142442	HP1BP3
rs10513954	18	64928523	0.0173	91.23%	0.067960	0.102900	0.03092000	0.99780000	NM_152721	upstream	290748	Hs.569915	DOK6
rs1094199	8	89538530	0.0173	91.23%	0.067960	0.102900	0.03092000	0.99790000	NM_003821	upstream	1300643	Hs.103755	RIPK2
rs722366	6	106080157	0.3388	96.84%	0.590600	1.000000	0.03092000	0.14000000	NM_002726	upstream	122495	Hs.436564	PREP
rs534731	9	119642451	0.3359	90.88%	0.003297	0.013230	0.03093000	0.02976000	NM_138554	downstream	123481	Hs.174312	TLR4
rs907808	15	97097777	0.3093	94.74%	0.568700	0.707100	0.03093000	0.01940000	NM_000875	intron	0	Hs.698330	IGF1R
rs723631	7	86239528	0.2133	97.89%	0.377700	0.324100	0.03096000	0.03535000	NM_000840	intron	0	Hs.590575	GRM3
rs10485011	6	22714921	0.0585	92.98%	1.000000	0.602100	0.03098000	0.00489100	NM_138574	downstream	35047	Hs.629246	HDGFL1
rs10487557	7	155689355	0.0556	82.11%	1.000000	1.000000	0.03099000	0.09129000	NM_032625	downstream	435611	---	C7orf13
rs10514263	5	83426017	0.0647	97.54%	0.614000	1.000000	0.03099000	0.06053000	NM_005711	intron	0	Hs.482730	EDIL3
rs6005907	22	27557971	0.1187	90.18%	0.000000	0.001073	0.03099000	0.10230000	NM_005080	upstream	31411	Hs.437638	XBP1
rs10520018	15	33715161	0.0475	92.28%	1.000000	1.000000	0.03100000	0.00815000	NM_080650	upstream	89505	Hs.107196	ATPBD4
rs738283	22	35167066	0.0291	96.49%	0.201100	1.000000	0.03100000	0.09546000	NM_012473	downstream	25973	Hs.211929	TXN2
rs10506525	12	64069645	0.2007	94.39%	0.252500	0.520800	0.03101000	0.03215000	NM_001031679	intron	0	Hs.339024	MSRB3
rs10501385	11	59981058	0.0167	94.39%	1.000000	1.000000	0.03102000	0.99800000	NM_021950	intron	0	Hs.438040	MS4A1
rs9298792	9	18370943	0.4203	96.84%	0.267800	0.761600	0.03103000	0.05422000	NM_052866	upstream	93156	Hs.522019	ADAMTSL1
rs10495979	2	49725943	0.1649	96.84%	0.826800	0.536200	0.03105000	0.04650000	ENST00000388647	downstream	243567	---	---
rs2645466	17	55207996	0.3217	90.53%	0.391400	0.349400	0.03108000	0.07099000	NM_030938	intron	0	Hs.444569	TMEM49
rs10511434	9	2104024	0.1524	94.39%	0.642400	0.490700	0.03109000	0.02397000	NM_003070	intron	0	Hs.298990	SMARCA2
rs10496310	2	84478534	0.0167	94.74%	1.000000	1.000000	0.03118000	0.99790000	NM_003849	downstream	25660	Hs.270428	SUCLG1
rs10496311	2	84478766	0.0167	94.74%	1.000000	1.000000	0.03118000	0.99790000	NM_003849	downstream	25428	Hs.270428	SUCLG1
rs6555016	5	1892458	0.0360	97.54%	1.000000	1.000000	0.03118000	0.04289000	NM_004553	downstream	9848	Hs.408257	NDUFS6
rs1376757	2	161300495	0.0914	97.89%	0.144300	0.134400	0.03119000	0.10480000	NM_002897	upstream	241944	Hs.470412	RBMS1
rs10483549	14	44403776	0.0574	94.74%	0.598800	1.000000	0.03120000	0.07768000	NM_001017923	upstream	32481	Hs.82098	C14orf28
rs10482914	21	21244794	0.0308	91.23%	0.211700	1.000000	0.03121000	0.01164000	NM_004540	upstream	329973	Hs.473450	NCAM2
rs898165	6	21316408	0.1401	90.18%	0.796200	0.575400	0.03122000	0.05723000	NM_017774	intron	0	Hs.657604	CDKAL1
rs6732705	2	56763256	0.4817	95.79%	0.182500	0.451800	0.03123000	0.03858000	NM_006296	upstream	1363968	Hs.631890	VRK2
rs725235	6	152695457	0.0662	95.44%	0.326000	0.333000	0.03123000	0.08183000	NM_033071	intron	0	Hs.12967	SYNE1
rs1022378	2	21910174	0.3685	94.74%	0.190300	0.042590	0.03125000	0.01266000	ENST00000383859	upstream	89196	---	---
rs9323834	14	88405366	0.1930	95.44%	0.247300	0.273600	0.03125000	0.07920000	NM_198310	intron	0	Hs.303055	TTC8
rs10487088	7	89408405	0.1147	93.33%	0.220900	1.000000	0.03126000	0.13750000	ENST00000317128	upstream	178245	---	---
rs9312259	4	181363535	0.1315	94.74%	0.791300	0.575700	0.03126000	0.06889000	ENST00000385763	downstream	279419	---	---
rs1886356	6	150535548	0.3943	86.32%	0.044840	0.126100	0.03128000	0.14830000	NM_030949	intron	0	Hs.486798	PPP1R14C
rs224938	5	81752932	0.4485	95.44%	0.623800	0.291000	0.03128000	0.02826000	NM_001039779	downstream	34841	---	FLJ41309
rs10500381	16	12065401	0.4309	96.49%	0.538500	0.763600	0.03129000	0.10600000	NM_032167	downstream	10759	Hs.458401	RUNDC2A
rs1490901	4	112377247	0.2870	97.19%	0.378000	0.330500	0.03130000	0.02362000	NM_000325	upstream	613544	Hs.643588	PITX2
rs10485530	20	15508116	0.0967	94.39%	0.722800	1.000000	0.03132000	0.03347000	NM_001033086	intron	0	---	MACROD2
rs10494785	1	197428385	0.4833	94.74%	0.225300	0.758400	0.03132000	0.04118000	ENST00000312868	downstream	242806	---	---
rs10502042	11	104096976	0.4221	96.84%	0.623600	0.172800	0.03132000	0.03742000	ENST00000375726	downstream	164900	---	---
rs1903154	8	21140781	0.0967	94.39%	0.722800	1.000000	0.03132000	0.10380000	ENST00000387614	upstream	359471	---	---
rs438016	20	15040594	0.1864	97.89%	0.233200	0.403000	0.03132000	0.08922000	NM_001033086	intron	0	---	MACROD2
rs1485741	8	25974937	0.0572	95.09%	1.000000	0.603100	0.03134000	0.16220000	ENST00000380737	intron	0	---	---
rs4464906	7	81111692	0.2140	97.54%	0.031300	0.142900	0.03134000	0.01467000	NM_001010934	downstream	54566	Hs.396530	HGF

rs1154961	8	123154424	0.0166	95.09%	1.000000	1.000000	0.03135000	0.99810000	NM_005328	upstream	431613	Hs.571528	HAS2
rs1448730	4	190034153	0.0166	95.09%	1.000000	1.000000	0.03135000	0.99790000	ENST00000321235	downstream	136701	---	---
rs2155786	18	28701558	0.0166	95.09%	1.000000	1.000000	0.03135000	0.99770000	NM_198995	downstream	70029	Hs.115461	C18orf34
rs2788862	4	25978818	0.0166	95.09%	1.000000	1.000000	0.03135000	0.99790000	NM_203284	intron	0	Hs.479396	RBPJ
rs7524644	1	119568206	0.0166	95.09%	1.000000	1.000000	0.03135000	0.99790000	NM_015836	upstream	83389	Hs.523506	WARS2
rs6917477	6	30133963	0.0896	97.89%	0.708800	1.000000	0.03138000	0.06683000	NM_014596	upstream	3052	Hs.57813	ZNRD1
rs988102	18	37687927	0.5000	89.12%	0.060450	0.121000	0.03138000	0.11730000	NM_002647	upstream	101270	Hs.464971	PIK3C3
rs2889921	1	220406261	0.4915	82.11%	0.090220	0.335300	0.03140000	0.02460000	ENST00000355775	downstream	114125	---	---
rs6760096	2	151628484	0.1447	95.79%	1.000000	0.788800	0.03140000	0.05916000	NM_198557	downstream	185672	Hs.302442	RBM43
rs1359932	6	97436898	0.4188	97.19%	0.216600	0.527700	0.03142000	0.15780000	NM_014165	downstream	7012	Hs.591333	C6orf66
rs893884	3	144050273	0.4723	88.77%	0.166100	0.635900	0.03146000	0.17340000	NM_013363	intron	0	Hs.8944	PCOLCE2
rs9300422	13	97021321	0.3141	94.39%	0.888000	0.510500	0.03149000	0.05853000	NM_021033	downstream	103078	Hs.508480	RAP2A
rs1560106	16	12616051	0.2374	83.51%	0.857900	1.000000	0.03150000	0.04315000	NM_018340	downstream	48387	Hs.460002	FLJ11151
rs11076556	16	49712544	0.0927	96.49%	0.714200	1.000000	0.03151000	0.01102000	NM_002968	downstream	15286	Hs.135787	SALL1
rs1029181	6	141790162	0.0650	97.19%	1.000000	0.330200	0.03152000	0.01732000	NM_006079	upstream	2052684	Hs.82071	CITED2
rs10489364	1	167134224	0.4278	97.19%	0.902800	0.639600	0.03153000	0.01937000	NM_001937	upstream	169098	Hs.80552	DPT
rs2419914	5	157777464	0.2906	97.19%	0.307900	0.434000	0.03153000	0.05314000	NM_024007	downstream	281233	Hs.657753	EBF1
rs185357	5	5808783	0.4763	88.77%	0.032180	0.086140	0.03156000	0.05891000	NM_001001702	downstream	556700	Hs.376760	FLJ33360
rs10491832	9	24709895	0.4176	97.89%	0.902700	0.530100	0.03157000	0.02758000	ENST00000380098	upstream	175660	---	---
rs2409411	21	31842275	0.4176	97.89%	1.000000	0.428400	0.03157000	0.01298000	NM_003253	intron	0	Hs.517228	TIAM1
rs3107648	8	104391979	0.1749	92.28%	0.394000	0.370600	0.03157000	0.05737000	NM_003506	intron	0	Hs.591863	FZD6
rs6135343	20	15060292	0.2878	97.54%	0.559800	0.330500	0.03159000	0.06872000	NM_001033086	intron	0	---	MACROD2
rs10493164	1	52491469	0.0361	97.19%	0.299200	1.000000	0.03160000	0.27240000	NM_004799	intron	0	Hs.532345	ZFYVE9
rs8042622	15	52692250	0.1170	92.98%	0.003788	0.180300	0.03160000	0.09402000	ENST00000260323	intron	0	---	---
rs1293135	20	52360876	0.4261	90.18%	0.073620	0.088140	0.03164000	0.10120000	NM_018431	upstream	164712	Hs.656582	DOK5
rs9309437	2	25956101	0.4196	89.47%	0.157400	0.868500	0.03165000	0.00710400	NM_018263	upstream	1262	Hs.700650	ASXL2
rs4897047	6	125894320	0.3264	92.98%	0.484400	0.742100	0.03168000	0.00377700	NM_012259	upstream	218099	Hs.144287	HEY2
rs147959	8	116469762	0.1974	95.09%	0.000444	0.002065	0.03170000	0.00323700	NM_014112	downstream	24209	Hs.657018	TRPS1
rs1709084	19	46294310	0.0970	94.04%	0.085030	1.000000	0.03172000	0.04236000	NM_000766	downstream	371	Hs.567252	CYP2A13
rs1870564	3	53691841	0.2934	90.88%	0.453300	0.692500	0.03172000	0.11940000	NM_000720	intron	0	Hs.476358	CACNA1D
rs719153	18	5945554	0.2667	94.74%	0.085700	0.010870	0.03173000	0.16400000	ENST00000317931	3UTR	0	---	---
rs7748312	6	38002867	0.1186	88.77%	0.012740	0.097970	0.03175000	0.01676000	NM_021943	intron	0	Hs.36959	ZFAND3
rs10495995	2	50102417	0.4191	97.54%	0.389500	0.527700	0.03177000	0.04402000	NM_138735	intron	0	Hs.637685	NRXN1
rs4876877	8	112837498	0.0418	88.07%	1.000000	1.000000	0.03177000	0.03662000	ENST00000364160	downstream	392598	---	---
rs10492821	16	71028325	0.1444	97.19%	0.475000	1.000000	0.03178000	0.16640000	ENST00000378742	downstream	60042	---	---
rs10505908	12	23905336	0.3921	79.65%	0.781000	0.740400	0.03178000	0.08281000	NM_178010	intron	0	Hs.657542	SOX5
rs1503160	3	106287101	0.2695	94.39%	1.000000	0.679700	0.03178000	0.10380000	NM_001627	upstream	281302	Hs.591293	ALCAM
rs1834971	19	33308700	0.3148	94.74%	0.157600	0.463200	0.03178000	0.07045000	ENST00000379373	upstream	479642	---	---
rs10487396	7	117722354	0.0568	95.79%	0.206500	1.000000	0.03182000	0.11220000	NM_019644	downstream	52377	Hs.657737	ANKRD7
rs1560325	5	97053454	0.0992	91.93%	0.486500	0.473400	0.03182000	0.03287000	ENST00000388601	downstream	13241	---	---
rs454574	20	15040864	0.1871	97.54%	0.233800	0.404900	0.03184000	0.09606000	NM_001033086	intron	0	---	MACROD2
rs7308694	12	5686942	0.2631	94.04%	0.000058	0.000355	0.03184000	0.07005000	NM_020373	intron	0	Hs.148970	TMEM16B
rs10502872	18	42364579	0.0970	94.04%	0.486000	0.602500	0.03185000	0.09889000	NM_144612	intron	0	---	LOXHD1
rs10492015	12	111201383	0.1753	95.09%	0.832900	0.770400	0.03186000	0.03136000	NM_173813	upstream	5443	---	C12orf51
rs2285808	12	111228156	0.1753	95.09%	0.832900	0.770400	0.03186000	0.03136000	NM_173813	upstream	32216	---	C12orf51
rs963265	9	72284355	0.4332	91.93%	0.165900	0.205600	0.03186000	0.07260000	NM_001007471	downstream	55433	Hs.47288	TRPM3
rs967079	5	28791750	0.4209	97.54%	0.624000	0.651000	0.03186000	0.12710000	ENST00000387488	downstream	131121	---	---
rs10505178	8	113416432	0.1444	97.19%	0.014840	0.013610	0.03188000	0.12460000	NM_052900	intron	0	Hs.91381	CSMD3
rs681511	1	42073888	0.2168	97.89%	0.859900	0.316600	0.03188000	0.07033000	NM_024503	intron	0	Hs.591503	HIVEP3
rs176435	6	103636845	0.0395	93.33%	1.000000	1.000000	0.03189000	0.03042000	NM_175768	downstream	1012194	Hs.654523	GRIK2
rs943537	9	71930214	0.3929	93.33%	0.073140	0.215500	0.03193000	0.04558000	NM_153267	intron	0	Hs.547172	MAMDC2
rs9307573	4	126773338	0.4436	96.49%	0.902700	0.213000	0.03197000	0.01070000	NM_024582	downstream	140965	Hs.702217	FAT4
rs10493808	1	88396583	0.2657	95.09%	0.353900	0.096150	0.03198000	0.03601000	ENST00000388450	downstream	319516	---	---
rs2280394	16	63539534	0.3165	93.68%	0.395500	0.460300	0.03200000	0.04894000	NM_001797	intron	0	Hs.116471	CDH11

rs10509715	10	99724181	0.0362	96.84%	0.300100	1.000000	0.03204000	0.04398000	NM_018058	intron	0	Hs.500736	CRTAC1
rs1573366	18	61107313	0.3369	97.89%	0.591600	0.381900	0.03204000	0.01274000	ENST00000387762	upstream	186416	---	---
rs2421930	2	117917574	0.0558	97.54%	0.587400	0.232200	0.03207000	0.11120000	NM_006773	upstream	371151	Hs.363492	DDX18
rs205795	20	55806890	0.3770	87.02%	0.498900	0.630000	0.03208000	0.09705000	NM_199171	upstream	86943	Hs.517155	TMEPAI
rs1567967	2	16332551	0.1434	97.89%	0.471900	0.592500	0.03209000	0.05558000	ENST00000388035	downstream	88506	---	---
rs633485	11	69130174	0.0665	97.54%	0.341400	0.352100	0.03209000	0.01541000	NM_053056	upstream	34880	Hs.523852	CCND1
rs964707	7	33202823	0.1148	94.74%	1.000000	0.368600	0.03210000	0.01088000	NM_001033605	intron	0	Hs.372360	BBS9
rs10504901	8	91867078	0.1321	92.98%	0.590200	0.377600	0.03211000	0.04205000	NM_022351	upstream	6213	Hs.560892	NECAB1
rs1529581	3	157989601	0.0690	94.04%	1.000000	1.000000	0.03212000	0.02596000	ENST00000314241	upstream	37169	---	---
rs10507337	13	24143752	0.0668	97.19%	1.000000	1.000000	0.03213000	0.02177000	NM_001676	upstream	8943	Hs.147111	ATP12A
rs10515767	5	157978895	0.0623	90.18%	0.250300	0.276800	0.03213000	0.05267000	NM_024007	downstream	79802	Hs.657753	EBF1
rs1884981	1	34078764	0.4850	81.75%	0.793200	0.071650	0.03213000	0.12500000	NM_052896	intron	0	Hs.656915	CSMD2
rs1386290	3	151354149	0.2041	93.68%	0.571100	0.673300	0.03214000	0.00840900	ENST00000386021	upstream	75841	---	---
rs10504658	8	78793709	0.0824	93.68%	0.087550	0.077020	0.03216000	0.04677000	NM_000318	upstream	718802	Hs.437966	PXMP3
rs1342705	1	145720880	0.1444	97.19%	0.624200	0.593900	0.03216000	0.10840000	NM_181703	upstream	8814	Hs.447968	GJA5
rs4634109	3	64267925	0.0291	96.49%	0.016760	0.042130	0.03216000	0.10580000	NM_198859	upstream	81754	Hs.699317	PRICKLE2
rs995636	20	6956824	0.2202	97.19%	0.381700	0.467300	0.03216000	0.03890000	NM_001200	downstream	247897	Hs.73853	BMP2
rs10488780	11	79490000	0.1938	96.84%	0.248600	0.173500	0.03217000	0.00772600	ENST00000363276	upstream	426652	---	---
rs10516770	4	87391122	0.0673	96.49%	0.346500	0.354900	0.03217000	0.10430000	NM_138981	intron	0	Hs.125503	MAPK10
rs3904729	10	84091786	0.2578	89.82%	1.000000	0.517100	0.03217000	0.15500000	NM_001010848	intron	0	Hs.125119	NRG3
rs163482	3	21538716	0.1927	96.49%	0.845900	1.000000	0.03218000	0.06240000	NM_024697	intron	0	Hs.21026	ZNF385D
rs2303554	2	182501229	0.0225	70.18%	1.000000	1.000000	0.03218000	0.99800000	NM_006751	intron	0	Hs.591602	SSFA2
rs2421542	2	125916248	0.3007	95.09%	0.885500	0.845500	0.03218000	0.00134800	NM_016815	upstream	1248296	Hs.59138	GYPC
rs2458439	12	69495562	0.1927	96.49%	0.444300	0.267400	0.03218000	0.12450000	NM_002849	intron	0	Hs.506076	PTPRR
rs647908	1	214171349	0.1330	93.68%	0.104400	0.035120	0.03218000	0.01524000	NM_206933	intron	0	Hs.655974	USH2A
rs9294558	6	93869536	0.0800	96.49%	0.685800	0.679600	0.03219000	0.02356000	NM_004440	downstream	138328	Hs.73962	EPHA7
rs223128	17	26933541	0.3093	94.74%	0.670400	0.502100	0.03221000	0.17110000	ENST00000362317	downstream	6888	---	---
rs858696	11	129679205	0.1820	91.58%	0.303500	0.485500	0.03222000	0.19180000	NM_014155	intron	0	---	ZBTB44
rs9321886	6	143382283	0.1306	94.04%	0.181100	1.000000	0.03222000	0.06802000	NM_016108	upstream	41423	Hs.567501	AIG1
rs1077572	13	108874772	0.0778	74.39%	0.116500	1.000000	0.03223000	0.03621000	NM_015011	downstream	216416	Hs.656587	MYO16
rs10495131	1	217872810	0.0608	92.28%	0.241700	0.267300	0.03225000	0.04657000	NM_138794	downstream	419980	Hs.657617	LYPLAL1
rs10501292	11	42952963	0.0848	78.60%	0.661700	0.661100	0.03226000	0.05583000	NM_006595	upstream	337146	Hs.435771	API5
rs6556635	5	162178999	0.4963	95.79%	0.147500	0.544100	0.03226000	0.02268000	NM_199246	upstream	618156	Hs.79101	CCNG1
rs689389	6	79348661	0.4564	96.49%	0.543400	0.880800	0.03226000	0.13500000	NM_001010844	upstream	285247	Hs.656212	IRAK1BP1
rs1021484	14	42934100	0.0647	97.54%	1.000000	1.000000	0.03228000	0.11820000	NM_152447	downstream	1490602	Hs.136893	LRFN5
rs10507070	12	94873188	0.0647	97.54%	0.614000	0.608900	0.03228000	0.03611000	NM_152435	intron	0	Hs.424907	AMDHD1
rs7123748	11	99278455	0.0217	97.19%	0.114600	1.000000	0.03229000	0.02744000	NM_175566	intron	0	Hs.656783	CNTN5
rs10502509	18	24245194	0.3504	96.14%	0.895200	0.599300	0.03230000	0.01151000	NM_001792	upstream	234005	Hs.464829	CDH2
rs790951	1	106065063	0.4286	90.88%	1.000000	0.871700	0.03231000	0.14100000	ENST00000388703	downstream	1082666	---	---
rs2981069	8	70100501	0.2461	89.82%	0.614300	0.847300	0.03232000	0.15730000	NM_052958	downstream	207157	Hs.491941	C8orf34
rs1159328	2	221779581	0.0771	97.89%	0.670200	0.463500	0.03234000	0.04574000	NM_004438	downstream	211412	Hs.371218	EPHA4
rs4442930	19	15912902	0.1913	97.19%	0.248600	0.511700	0.03236000	0.11490000	NM_021187	upstream	6576	Hs.187393	CYP4F11
rs4690560	4	178899904	0.4160	91.93%	0.799700	1.000000	0.03236000	0.21200000	ENST00000365495	downstream	160836	---	---
rs3125353	6	58629563	0.4319	97.89%	0.808100	0.549500	0.03237000	0.01363000	NM_206911	upstream	233908	---	GUSBL2
rs302957	8	106567598	0.4072	92.63%	1.000000	0.283100	0.03239000	0.01623000	NM_012082	intron	0	Hs.431009	ZFPM2
rs311326	6	153179314	0.2019	92.98%	0.702200	1.000000	0.03239000	0.00691400	NM_194435	downstream	56721	Hs.53973	VIP
rs10518396	1	87945984	0.0779	96.84%	1.000000	0.467500	0.03240000	0.01875000	ENST00000362448	upstream	254340	---	---
rs4030582	3	144231408	0.2704	94.74%	0.757400	0.300800	0.03240000	0.08501000	ENST00000319822	intron	0	---	---
rs1957210	14	39382051	0.3435	97.54%	0.110200	0.424100	0.03241000	0.04847000	NM_203301	upstream	410680	Hs.324342	FBXO33
rs787111	10	29096634	0.2019	94.74%	0.450600	0.521300	0.03241000	0.15070000	ENST00000364131	upstream	6896	---	---
rs10519382	4	137315609	0.1685	96.84%	0.666000	0.481700	0.03242000	0.03394000	ENST00000384592	upstream	178912	---	---
rs887010	7	66762679	0.1549	74.74%	0.299600	0.240600	0.03242000	0.03488000	ENST00000388509	upstream	260626	---	---
rs249721	5	115050156	0.1500	94.74%	0.089270	0.184400	0.03246000	0.15120000	NM_021649	upstream	60546	Hs.705455	TICAM2
rs935661	2	45908861	0.4164	92.28%	0.799600	0.877700	0.03247000	0.02628000	NM_005400	intron	0	Hs.580351	PRKCE

rs10517276	4	53624201	0.2063	94.39%	0.576500	0.204700	0.03249000	0.00943400	NM_152540	intron	0	Hs.302287	SCFD2
rs1878759	4	73733126	0.4033	85.26%	0.690600	0.613100	0.03249000	0.04394000	NM_014243	upstream	79746	Hs.590919	ADAMTS3
rs2201179	2	140669861	0.4046	91.93%	0.020950	0.191200	0.03249000	0.02919000	ENST00000385554	upstream	21430	---	---
rs1647598	5	154844311	0.3843	94.04%	0.301200	0.756000	0.03251000	0.05165000	ENST00000385835	downstream	75758	---	---
rs4543079	4	47322588	0.2921	97.89%	0.885500	0.396300	0.03251000	0.01374000	NM_006587	intron	0	Hs.518618	CORIN
rs7214679	17	16383189	0.0289	97.19%	0.199800	1.000000	0.03253000	0.10180000	NM_020653	downstream	12247	Hs.99724	ZNF287
rs7089102	10	57516173	0.4923	90.88%	0.535600	0.358200	0.03257000	0.01928000	NM_001005414	downstream	270822	---	ZWINT
rs10501181	11	37604748	0.0766	87.02%	0.041890	0.160400	0.03259000	0.01800000	NM_138787	downstream	967351	Hs.406726	C11orf74
rs1587535	3	158462900	0.3922	94.39%	0.798400	0.877600	0.03259000	0.04510000	NM_024621	intron	0	Hs.658046	VEPH1
rs2570100	4	58766242	0.4544	96.14%	0.544400	0.352500	0.03259000	0.22900000	ENST00000387903	upstream	712946	---	---
rs10492822	16	71028485	0.1452	97.89%	0.473700	1.000000	0.03262000	0.11210000	ENST00000378742	downstream	59882	---	---
rs10507236	12	110952822	0.1571	91.58%	0.240700	1.000000	0.03262000	0.05572000	NM_024953	intron	0	Hs.530941	C12orf30
rs10490956	10	11168479	0.1715	96.14%	0.204500	0.342200	0.03263000	0.22260000	NM_001025077	upstream	78977	Hs.309288	CUGBP2
rs10516940	4	94813222	0.3423	91.23%	0.680200	0.372400	0.03263000	0.01419000	NM_001510	intron	0	Hs.480281	GRID2
rs10491158	17	52099116	0.1693	88.07%	0.376300	0.747100	0.03268000	0.09210000	NM_005450	downstream	71574	Hs.248201	NOG
rs1385986	4	72111975	0.0389	94.74%	0.332600	1.000000	0.03268000	0.44790000	NM_000788	intron	0	Hs.709	DCK
rs10514164	5	79000485	0.1766	94.39%	0.010120	0.032210	0.03269000	0.04366000	NM_173797	CDS	0	Hs.418198	PAPD4
rs7738188	6	162733654	0.4802	88.77%	0.078100	0.057280	0.03269000	0.02109000	NM_013988	intron	0	Hs.132954	PARK2
rs10506615	12	69590946	0.0215	97.89%	1.000000	1.000000	0.03272000	0.03734000	NM_002849	intron	0	Hs.506076	PTPRR
rs10513244	3	145877784	0.0215	97.89%	1.000000	1.000000	0.03272000	0.10280000	ENST00000355888	downstream	230408	---	---
rs2200946	3	21698622	0.0595	94.39%	0.055560	0.266100	0.03272000	0.03222000	NM_024697	intron	0	Hs.21026	ZNF385D
rs2132528	8	50768208	0.1328	95.09%	0.439500	1.000000	0.03273000	0.03344000	NM_018967	upstream	701118	Hs.584914	SNTG1
rs7819004	8	50698414	0.1328	95.09%	0.439500	1.000000	0.03273000	0.03344000	NM_018967	upstream	770912	Hs.584914	SNTG1
rs1026909	4	65011960	0.0794	97.19%	1.000000	0.381300	0.03275000	0.03962000	NM_001010874	upstream	54191	Hs.227752	SRD5A2L2
rs10494469	1	165417387	0.1233	79.65%	0.756500	1.000000	0.03276000	0.00414000	NM_002697	upstream	39372	Hs.493649	POU2F1
rs10488509	7	91455027	0.2572	96.84%	0.155100	0.270100	0.03277000	0.07162000	NM_147166	intron	0	Hs.651221	AKAP9
rs9283944	8	107403285	0.2639	94.39%	0.004538	0.003338	0.03277000	0.17710000	NM_181354	upstream	335985	Hs.148778	OXR1
rs4902965	14	71529448	0.0985	92.63%	0.088600	0.601600	0.03278000	0.07527000	NM_004296	intron	0	Hs.509872	RGS6
rs10520496	4	182751386	0.4019	92.98%	0.702300	0.877700	0.03279000	0.02032000	ENST00000378957	downstream	70238	---	---
rs10503275	8	4601576	0.0376	93.33%	1.000000	1.000000	0.03280000	0.03664000	ENST00000383934	downstream	371633	---	---
rs10484944	6	80104580	0.0734	90.88%	0.634000	0.632200	0.03283000	0.02074000	NM_181714	downstream	146847	Hs.21945	LCA5
rs4764768	12	99828039	0.2731	95.09%	0.761900	0.723900	0.03284000	0.01150000	NM_178826	intron	0	Hs.58785	TMEM16D
rs1387837	10	19043099	0.4301	97.89%	0.114400	0.099540	0.03285000	0.04379000	NM_178815	downstream	36153	Hs.25362	ARL5B
rs1399899	3	162929198	0.4301	97.89%	0.181700	0.001605	0.03285000	0.03431000	ENST00000327928	downstream	224774	---	---
rs4873328	8	50269412	0.4742	88.42%	0.800400	0.337500	0.03285000	0.14000000	NM_001007176	downstream	118698	Hs.49890	C8orf22
rs6668120	1	177740186	0.3484	97.19%	0.236700	0.749900	0.03286000	0.04039000	NM_182766	intron	0	Hs.658505	C1orf125
rs872037	2	41494861	0.2333	94.74%	0.866400	0.327900	0.03287000	0.00814400	NM_021097	upstream	960673	Hs.468274	SLC8A1
rs10484586	6	143364121	0.0678	95.79%	0.620200	0.616100	0.03288000	0.10390000	NM_016108	upstream	59595	Hs.567501	AIG1
rs10518800	15	53251181	0.0678	95.79%	1.000000	1.000000	0.03288000	0.03244000	NM_016304	downstream	9632	Hs.274772	C15orf15
rs10495650	2	16406714	0.1530	94.04%	0.812600	0.297900	0.03289000	0.04279000	ENST00000388035	downstream	162669	---	---
rs7594267	2	23286410	0.3594	89.82%	0.892000	0.871700	0.03289000	0.00561000	ENST00000288548	upstream	175665	---	---
rs1450194	9	117771594	0.1537	76.49%	0.186500	0.258700	0.03290000	0.08586000	NM_021208	upstream	44287	---	C9orf27
rs10502754	18	37054962	0.0720	92.63%	1.000000	1.000000	0.03291000	0.06792000	NM_002647	upstream	734235	Hs.464971	PIK3C3
rs1401608	8	139451116	0.2271	95.79%	0.492700	0.336400	0.03291000	0.00467700	NM_015912	upstream	1687	Hs.126024	FAM135B
rs1954669	14	27134852	0.0597	94.04%	0.608800	1.000000	0.03291000	0.07213000	NM_006491	upstream	998052	Hs.31588	NOVA1
rs10483102	22	17794222	0.0287	97.89%	1.000000	1.000000	0.03292000	0.02144000	NM_003325	intron	0	Hs.474206	HIRA
rs2291500	3	56633354	0.1567	94.04%	0.109600	0.124800	0.03292000	0.03975000	NM_015224	intron	0	Hs.116877	C3orf63
rs2736553	11	5341005	0.3173	95.09%	0.888500	0.870000	0.03294000	0.01647000	NM_005330	intron	0	Hs.655195	HBE1
rs10515853	5	162588458	0.0558	97.54%	0.200700	0.233400	0.03295000	0.36090000	NM_199246	upstream	208697	Hs.79101	CCNG1
rs1196184	2	182593441	0.0590	95.09%	0.609100	1.000000	0.03295000	0.01735000	ENST00000280295	intron	0	---	---
rs1571686	21	31018283	0.0590	95.09%	0.230900	0.264800	0.03295000	0.01996000	NM_181617	downstream	22857	Hs.553699	KRTAP21-2
rs6133822	20	10107081	0.4691	96.49%	0.467400	0.878100	0.03295000	0.16120000	NM_130811	upstream	40396	Hs.167317	SNAP25
rs7791296	7	54188384	0.1537	94.74%	0.238800	0.204700	0.03295000	0.06507000	NM_001001707	upstream	341274	---	FLJ45974
rs167602	11	5518171	0.0290	96.84%	1.000000	1.000000	0.03299000	0.02035000	NM_001005289	downstream	4196	Hs.554540	OR52H1

rs2166363	5	3694988	0.4316	92.28%	0.257800	0.541200	0.03299000	0.09265000	NM_024337	downstream	40471	Hs.424156	IRX1
rs7582749	2	37452841	0.3640	87.72%	0.000202	0.019050	0.03300000	0.08072000	NM_012413	intron	0	Hs.79033	QPCT
rs10517356	4	56213050	0.3810	94.39%	0.606900	1.000000	0.03303000	0.10190000	NM_006681	upstream	15828	Hs.418367	NMU
rs1759092	19	39368378	0.4603	97.19%	0.185400	0.229900	0.03303000	0.09377000	NM_015578	intron	0	Hs.407368	LSM14A
rs10518388	4	122499785	0.0833	94.74%	0.097360	0.152400	0.03306000	0.08553000	NM_198179	intron	0	Hs.368977	GPR103
rs16847672	4	73324748	0.2069	91.58%	0.036260	0.178500	0.03307000	0.07259000	NM_014243	downstream	40803	Hs.590919	ADAMTS3
rs2743870	6	15732741	0.1093	97.89%	0.755200	0.512600	0.03307000	0.08032000	NM_183041	intron	0	Hs.571148	DTNBP1
rs959655	18	48132862	0.1093	97.89%	0.008699	0.089390	0.03307000	0.01979000	NM_005215	intron	0	Hs.579550	DCC
rs10482947	21	24659428	0.1254	97.89%	0.783000	0.550100	0.03309000	0.05049000	ENST00000355181	upstream	123713	---	---
rs16921658	8	56422954	0.0390	94.39%	0.052620	1.000000	0.03309000	0.10580000	NM_052898	intron	0	Hs.130197	XKR4
rs2513600	11	105805209	0.1406	89.82%	0.000213	0.000815	0.03311000	0.03398000	NM_000855	downstream	257911	Hs.654555	GUCY1A2
rs10490353	2	45270098	0.1556	94.74%	0.487200	0.502100	0.03312000	0.02349000	ENST00000378479	intron	0	---	---
rs10079088	5	102648573	0.1260	86.32%	0.000000	0.001505	0.03314000	0.05619000	NM_033211	downstream	6313	Hs.482976	C5orf30
rs2714610	7	66778682	0.1000	94.74%	0.087720	0.080790	0.03314000	0.00470400	ENST00000388509	upstream	276629	---	---
rs7870306	9	29308870	0.1379	91.58%	0.600600	0.776300	0.03316000	0.07806000	NM_152570	upstream	648587	Hs.699432	LINGO2
rs10485014	6	22779332	0.0728	91.58%	1.000000	0.366000	0.03318000	0.02924000	NM_138574	downstream	99458	Hs.629246	HDGFL1
rs10503899	8	32066776	0.4151	92.98%	0.447400	0.359700	0.03319000	0.02258000	NM_013962	intron	0	Hs.453951	NRG1
rs8025054	15	84616500	0.3047	96.14%	0.886800	1.000000	0.03320000	0.07871000	NM_152336	intron	0	Hs.679833	AGBL1
rs9320422	6	113304686	0.1282	97.19%	0.787400	1.000000	0.03321000	0.02039000	ENST00000364516	upstream	94702	---	---
rs7099339	10	125040124	0.4373	92.28%	0.259300	0.170400	0.03322000	0.11670000	NM_153442	upstream	375737	Hs.12751	GPR26
rs7068381	10	107980672	0.4611	90.18%	0.061150	0.019510	0.03323000	0.00540100	NM_001013031	downstream	346297	Hs.591915	SORCS1
rs846452	6	79348887	0.4516	97.89%	0.629300	1.000000	0.03323000	0.11750000	NM_001010844	upstream	285021	Hs.656212	IRAK1BP1
rs9301680	13	89949096	0.4783	88.77%	0.314000	0.750200	0.03324000	0.05387000	ENST00000385123	downstream	267564	---	---
rs3172355	3	56631087	0.1803	94.39%	0.406800	0.253300	0.03327000	0.02534000	NM_001012506	downstream	211	Hs.476399	CCDC66
rs2000349	14	97785329	0.2264	96.84%	0.605500	1.000000	0.03328000	0.10060000	ENST00000387922	downstream	102128	---	---
rs9309989	3	85882197	0.1226	90.18%	0.077960	0.672900	0.03330000	0.09983000	NM_153184	intron	0	Hs.164578	CADM2
rs2565210	15	52085319	0.0560	97.19%	0.201800	1.000000	0.03333000	0.08087000	ENST00000380705	upstream	89218	---	---
rs4264258	13	100889192	0.2011	95.09%	0.850600	0.789500	0.03333000	0.03516000	NM_004791	upstream	13665	Hs.696554	ITGBL1
rs10502198	11	114240545	0.0531	85.96%	1.000000	1.000000	0.03334000	0.03139000	NM_182495	downstream	157050	---	FAM55B
rs2030879	12	73656959	0.0218	96.49%	0.115400	1.000000	0.03335000	0.09930000	NM_153748	downstream	63204	Hs.591041	KCNC2
rs9297675	8	124872878	0.1882	89.47%	0.220600	0.380600	0.03336000	0.03450000	NM_144963	intron	0	Hs.459174	FAM91A1
rs4799	2	10850700	0.4801	70.53%	0.572900	0.303200	0.03338000	0.01459000	NM_005742	CDS	0	Hs.212102	PDIA6
rs591364	18	67338997	0.2230	97.54%	0.389400	0.430700	0.03341000	0.01414000	NM_182511	downstream	1015898	Hs.569851	CBLN2
rs1018283	4	73291711	0.4496	97.54%	0.544400	1.000000	0.03343000	0.01220000	NM_053036	downstream	59069	Hs.99231	NPFFR2
rs25810	5	129015788	0.3259	94.74%	0.491900	0.148900	0.03343000	0.04169000	NM_133638	intron	0	Hs.23751	ADAMTS19
rs10513443	3	154487130	0.0595	94.39%	0.233500	1.000000	0.03344000	0.08780000	ENST00000383956	upstream	86634	---	---
rs1350283	8	95291352	0.3588	89.47%	0.000001	0.000067	0.03344000	0.02618000	NM_004063	upstream	1366	Hs.591853	CDH17
rs687862	18	67338741	0.2204	97.89%	0.294600	0.315600	0.03344000	0.01957000	NM_182511	downstream	1016154	Hs.569851	CBLN2
rs10508061	13	100757140	0.2490	91.58%	0.245100	0.261000	0.03346000	0.08122000	NM_052867	intron	0	Hs.525146	NALCN
rs1403839	7	145122893	0.1654	89.12%	1.000000	0.616600	0.03346000	0.01326000	ENST00000385572	upstream	202461	---	---
rs439222	5	125229474	0.4801	96.84%	1.000000	0.294100	0.03346000	0.02192000	NM_023927	upstream	557526	Hs.363558	GRAMD3
rs7856322	9	9821142	0.1362	94.04%	0.296400	0.050670	0.03346000	0.06423000	ENST00000363183	downstream	388790	---	---
rs9301021	13	104774248	0.3556	97.19%	0.191500	0.058000	0.03347000	0.05614000	NM_172370	upstream	142345	Hs.381382	DAOA
rs945642	1	102236102	0.2601	87.02%	0.004574	0.003907	0.03348000	0.01216000	NM_058170	upstream	928	Hs.484475	OLFM3
rs1532379	7	97626429	0.1942	97.54%	0.338500	0.190000	0.03350000	0.00582300	NM_014916	intron	0	Hs.444179	LMTK2
rs2063182	12	73370892	0.1713	89.12%	1.000000	0.756200	0.03350000	0.15610000	ENST00000344556	upstream	26710	---	---
rs2172712	1	163354116	0.4173	93.33%	0.899700	0.358700	0.03350000	0.18420000	NM_001033507	downstream	83618	Hs.667312	LMX1A
rs3800756	7	133753838	0.3513	97.89%	0.793000	0.388200	0.03350000	0.06736000	NM_001628	downstream	23811	Hs.521212	AKR1B1
rs4944529	11	85006039	0.2398	94.39%	0.132900	0.361200	0.03350000	0.22550000	NM_018480	upstream	11257	Hs.525063	TMEM126B
rs964572	3	145162615	0.1028	88.77%	0.032880	0.019660	0.03350000	0.03767000	NM_173552	upstream	10988	Hs.288954	C3orf58
rs10494509	1	176267683	0.1136	84.91%	1.000000	1.000000	0.03353000	0.01751000	ENST00000308284	intron	0	---	---
rs1395874	4	58641115	0.2817	94.04%	0.548600	1.000000	0.03353000	0.07886000	ENST00000387903	upstream	587819	---	---
rs1423260	5	39937278	0.2309	96.49%	0.172400	0.247700	0.03353000	0.04562000	NM_001343	upstream	476575	Hs.481980	DAB2
rs10491962	12	2313149	0.0396	92.98%	1.000000	1.000000	0.03354000	0.13530000	NM_000719	intron	0	Hs.118262	CACNA1C

rs17145129	5	118668070	0.0235	89.47%	0.124000	1.000000	0.03355000	0.01995000	NM_005509	downstream	55349	Hs.181042	DMXL1
rs4834724	4	120080425	0.1148	94.74%	1.000000	1.000000	0.03355000	0.04131000	NM_133477	intron	0	Hs.655519	SYNPO2
rs1848284	15	47903387	0.3410	91.58%	0.129600	0.150600	0.03356000	0.02421000	NM_024837	downstream	34340	Hs.511311	ATP8B4
rs5542	2	40195164	0.4657	97.19%	0.054390	0.097690	0.03356000	0.13330000	NM_021097	3UTR	0	Hs.468274	SLC8A1
rs3011384	10	122583101	0.1685	97.89%	1.000000	0.537600	0.03358000	0.04562000	ENST00000369065	intron	0	---	---
rs764133	18	63441694	0.2950	97.54%	1.000000	0.609700	0.03358000	0.05389000	NM_032160	upstream	106747	Hs.124673	DSEL
rs1410054	10	112764145	0.1494	95.09%	0.472400	0.790000	0.03361000	0.07490000	NM_007373	downstream	730	Hs.104315	SHOC2
rs10497118	2	153432089	0.1889	91.93%	0.311500	0.163400	0.03365000	0.03145000	NM_152522	downstream	106152	Hs.516468	ARL6IP6
rs10511231	3	106125687	0.1935	97.89%	0.084660	0.028070	0.03365000	0.17340000	NM_001627	upstream	442716	Hs.591293	ALCAM
rs10490812	3	45152010	0.1240	89.12%	0.777000	0.373800	0.03366000	0.01088000	NM_178181	intron	0	Hs.476093	CDCP1
rs601057	7	42941352	0.1982	79.65%	0.019440	0.141300	0.03366000	0.07510000	NM_031903	intron	0	Hs.50252	MRPL32
rs1577302	10	19690726	0.0871	92.63%	0.001283	0.000793	0.03368000	0.23710000	ENST00000377265	upstream	136696	---	---
rs3787168	20	33696692	0.1580	94.39%	1.000000	1.000000	0.03370000	0.03041000	NM_152931	intron	0	Hs.246413	CPNE1
rs10489972	2	101887046	0.0535	85.26%	0.506400	0.546400	0.03371000	0.02899000	NM_145687	downstream	9463	Hs.431550	MAP4K4
rs10514482	16	78709428	0.0701	95.09%	1.000000	1.000000	0.03373000	0.04755000	NM_130897	upstream	422927	Hs.98849	DYNLRB2
rs1527672	7	94401948	0.4578	78.95%	0.346700	0.333200	0.03373000	0.13880000	NM_017650	intron	0	Hs.21816	PPP1R9A
rs7828353	8	52779963	0.0701	95.09%	1.000000	1.000000	0.03373000	0.03304000	NM_052937	upstream	112735	Hs.308480	PCMTD1
rs1378647	7	136283723	0.4731	97.89%	0.149400	0.761700	0.03377000	0.04633000	NM_001006629	intron	0	Hs.535891	CHRM2
rs3211828	7	80117539	0.1038	92.98%	0.175400	0.325000	0.03378000	0.00843700	NM_000072	intron	0	Hs.120949	CD36
rs4728499	7	81610505	0.2563	97.89%	0.083360	0.283100	0.03378000	0.27680000	NM_000722	intron	0	Hs.282151	CACNA2D1
rs1380424	4	36489912	0.3352	93.68%	1.000000	0.857900	0.03379000	0.00743700	ENST00000357504	downstream	468101	---	---
rs1494409	1	163354456	0.4139	93.68%	0.900100	0.167500	0.03379000	0.18410000	NM_001033507	downstream	85279	Hs.667312	LMX1A
rs9306903	2	16977976	0.2256	97.19%	0.305400	0.634100	0.03379000	0.05988000	ENST00000386331	downstream	78220	---	---
rs1032077	3	177508198	0.2116	93.68%	0.198000	0.039420	0.03380000	0.03105000	ENST00000366444	downstream	279800	---	---
rs1029039	9	77843658	0.3686	82.81%	1.000000	1.000000	0.03382000	0.03758000	NM_006200	intron	0	Hs.368542	PCSK5
rs10506073	12	31499882	0.1175	94.04%	0.551500	0.535400	0.03383000	0.01517000	NM_144973	intron	0	Hs.118166	MGC24039
rs10484262	6	10089479	0.0849	95.09%	1.000000	1.000000	0.03385000	0.02141000	NM_153003	intron	0	Hs.532138	OFCC1
rs1947432	2	39080567	0.0852	94.74%	0.704100	0.697700	0.03385000	0.00466300	NM_005633	intron	0	Hs.654397	SOS1
rs2939213	11	28944086	0.0683	97.54%	0.367100	0.362100	0.03385000	0.03954000	NM_152636	downstream	632567	Hs.243326	METT5D1
rs7945783	11	28793038	0.0683	97.54%	0.367100	0.362100	0.03385000	0.03566000	NM_152636	downstream	481519	Hs.243326	METT5D1
rs1445698	5	85718380	0.2238	97.19%	0.605700	0.814300	0.03387000	0.01139000	NM_001867	upstream	231097	Hs.430075	COX7C
rs10507946	13	84451033	0.0450	97.54%	1.000000	1.000000	0.03388000	0.05363000	ENST00000377082	upstream	145472	---	---
rs1807159	19	22570023	0.1828	94.04%	0.540800	0.578400	0.03388000	0.12340000	ENST00000387067	upstream	6124	---	---
rs653166	1	192497072	0.0450	97.54%	0.431400	0.465400	0.03388000	0.12840000	ENST00000385495	downstream	226784	---	---
rs10511577	9	13144803	0.2399	95.09%	0.868400	0.823200	0.03389000	0.03993000	NM_003829	intron	0	Hs.169378	MPDZ
rs1067637	12	62815387	0.4821	97.89%	1.000000	0.654700	0.03389000	0.13650000	NM_020762	intron	0	Hs.450763	SRGAP1
rs1564692	4	127403558	0.2399	95.09%	0.738800	0.260300	0.03389000	0.15980000	NM_024582	downstream	771185	Hs.702217	FAT4
rs847328	14	71900759	0.1935	87.02%	0.224900	0.374400	0.03391000	0.02196000	NM_004296	intron	0	Hs.509872	RGS6
rs918961	2	60074049	0.2741	94.74%	0.021340	0.049090	0.03391000	0.08181000	ENST00000386566	downstream	298559	---	---
rs306079	5	110683306	0.4785	97.89%	1.000000	0.762900	0.03394000	0.02281000	NM_001744	intron	0	Hs.591269	CAMK4
rs2189464	7	8600283	0.3296	94.74%	0.129600	0.141700	0.03397000	0.05549000	NM_152745	upstream	156880	Hs.487564	NXPH1
rs10487882	7	9816524	0.1480	97.19%	1.000000	0.313400	0.03398000	0.04200000	ENST00000361014	downstream	82084	---	---
rs1401879	12	88053742	0.0393	93.68%	0.335700	1.000000	0.03401000	0.07268000	ENST00000364267	downstream	146389	---	---
rs10484737	6	124724705	0.2590	97.54%	0.438400	0.274400	0.03402000	0.01370000	NM_001040214	intron	0	Hs.656604	NKAIN2
rs10495648	2	15977810	0.3633	97.54%	0.194200	0.273500	0.03405000	0.04525000	ENST00000388030	downstream	16465	---	---
rs10498431	14	51213444	0.4113	92.98%	0.446800	0.415900	0.03405000	0.05358000	NM_152330	intron	0	Hs.434914	FRMD6
rs4637381	4	154889706	0.3333	61.05%	0.000001	0.000033	0.03405000	0.06419000	NM_173662	intron	0	Hs.388364	RNF175
rs10499273	6	153591089	0.1953	97.89%	0.850100	0.592500	0.03407000	0.04118000	ENST00000312401	downstream	54026	---	---
rs10501321	11	47251202	0.2395	91.58%	0.017870	0.049470	0.03408000	0.04469000	NM_130476	intron	0	Hs.82548	MADD
rs3759213	12	97797721	0.1984	88.42%	0.000038	0.000249	0.03408000	0.03131000	NM_020140	intron	0	Hs.506458	ANKS1B
rs1941619	18	20848396	0.3240	93.68%	0.025940	0.019160	0.03409000	0.17670000	NM_021624	downstream	536829	Hs.287388	HRH4
rs2940341	7	112996798	0.1615	91.23%	0.356100	0.184900	0.03410000	0.21090000	NM_002711	downstream	304824	Hs.458309	PPP1R3A
rs10491367	5	54239231	0.2085	95.09%	0.267500	0.212000	0.03412000	0.01237000	NM_007036	downstream	70218	Hs.129944	ESM1
rs7736638	5	124570049	0.0474	96.14%	0.463300	0.503100	0.03412000	0.07502000	NM_020747	upstream	461345	Hs.266616	ZNF608

rs7961701	12	79937739	0.1027	90.53%	0.009340	0.022670	0.03412000	0.02235000	NM_024560	upstream	58201	Hs.259559	ACSS3
rs195476	6	116279962	0.4856	97.54%	0.402900	0.135900	0.03413000	0.01328000	NM_002031	downstream	89424	Hs.89426	FRK
rs3829918	10	3198140	0.3745	96.49%	0.370100	0.440400	0.03413000	0.04814000	NM_014889	intron	0	Hs.528300	PITRM1
rs10508208	10	900983	0.1906	92.98%	0.323400	0.266300	0.03414000	0.12160000	NM_015155	intron	0	Hs.705392	LARP5
rs2300881	2	37390926	0.3646	97.19%	0.244000	0.735000	0.03414000	0.08396000	NM_005813	intron	0	Hs.696257	PRKD3
rs697491	6	166361018	0.3705	88.07%	1.000000	0.860400	0.03414000	0.14260000	NM_001013720	downstream	37926	---	LOC441177
rs1929488	9	103205819	0.0830	95.09%	0.702700	0.692700	0.03415000	0.08284000	NM_197977	intron	0	Hs.50123	ZNF189
rs1929491	9	103206021	0.0830	95.09%	0.702700	0.692700	0.03415000	0.08284000	NM_197977	intron	0	Hs.50123	ZNF189
rs1322178	6	106738473	0.1162	95.09%	0.550600	0.536700	0.03417000	0.12790000	NM_004849	downstream	571	Hs.486063	ATG5
rs10512754	5	41039556	0.3736	95.79%	0.198700	0.531900	0.03418000	0.10570000	NM_173489	intron	0	Hs.97714	FLJ40243
rs2413338	22	33993523	0.4157	93.68%	1.000000	0.761500	0.03419000	0.09407000	NM_001003681	intron	0	Hs.588815	HMG2L1
rs423463	6	89955427	0.2654	74.04%	0.289000	0.817200	0.03419000	0.08986000	NM_002042	intron	0	Hs.437745	GABRR1
rs10488914	4	112031737	0.0824	95.79%	0.406700	1.000000	0.03421000	0.04360000	NM_000325	upstream	268034	Hs.643588	PITX2
rs224924	5	81756475	0.4415	92.98%	0.318500	0.638600	0.03421000	0.19980000	NM_001039779	downstream	38384	---	FLJ41309
rs2366979	5	38414969	0.0467	78.95%	0.072780	1.000000	0.03421000	0.07774000	NM_182799	intron	0	Hs.20103	EGFLAM
rs10484706	6	56155275	0.0448	97.89%	1.000000	1.000000	0.03422000	0.04235000	NM_030820	intron	0	Hs.47629	COL21A1
rs10497731	2	193021180	0.0815	96.84%	1.000000	0.501000	0.03422000	0.02734000	NM_016192	upstream	253293	Hs.144513	TMEFF2
rs885631	6	56209793	0.0448	97.89%	1.000000	1.000000	0.03422000	0.04235000	NM_030820	intron	0	Hs.47629	COL21A1
rs10488554	7	93020251	0.2452	91.58%	0.179200	0.061120	0.03424000	0.10940000	NM_001742	intron	0	Hs.489127	CALCR
rs10518716	15	65281877	0.3040	97.54%	0.888300	0.614400	0.03424000	0.02981000	NM_024666	3UTR	0	Hs.254642	FLJ11506
rs10504433	8	70021434	0.1347	95.09%	0.037980	0.154100	0.03428000	0.11200000	NM_052958	downstream	128090	Hs.491941	C8orf34
rs7739686	6	23669660	0.2132	92.98%	0.715200	0.129100	0.03428000	0.08295000	ENST00000330282	upstream	293201	---	---
rs914840	13	65255649	0.2299	96.14%	0.305500	0.813800	0.03428000	0.02089000	ENST00000321014	downstream	4416	---	---
rs6688573	1	106110661	0.2572	97.54%	1.000000	1.000000	0.03432000	0.08644000	ENST00000388703	downstream	1037068	---	---
rs1469063	5	164791994	0.2768	95.09%	0.000808	0.004419	0.03434000	0.24790000	ENST00000385732	upstream	177030	---	---
rs10490478	2	207636308	0.0688	96.84%	0.370700	1.000000	0.03441000	0.02441000	NM_003709	downstream	17015	Hs.471221	KLF7
rs10512393	9	111192977	0.4330	91.58%	0.023320	0.120400	0.03441000	0.15850000	NM_002829	intron	0	Hs.436429	PTPN3
rs4753569	11	93653841	0.3755	95.79%	0.605700	0.756200	0.03443000	0.04328000	NM_015368	downstream	99060	Hs.591976	PANX1
rs565685	6	129021415	0.2711	78.95%	0.735800	0.173500	0.03444000	0.13510000	ENST00000385937	upstream	40062	---	---
rs1589764	7	10362495	0.3032	89.12%	0.181000	0.426600	0.03445000	0.09933000	ENST00000387411	downstream	133332	---	---
rs10492296	12	85656971	0.0451	97.19%	0.432600	0.465400	0.03448000	0.05298000	NM_013244	upstream	689325	Hs.662231	MGAT4C
rs3136409	8	95516438	0.1370	94.74%	0.797000	0.702800	0.03448000	0.05812000	NM_012415	intron	0	Hs.30561	RAD54B
rs331622	13	109088827	0.0451	97.19%	0.432600	0.465400	0.03448000	0.02681000	NM_003749	downstream	115358	Hs.442344	IRS2
rs1333667	6	10296494	0.0293	95.79%	1.000000	1.000000	0.03449000	0.04032000	NM_153003	upstream	127586	Hs.532138	OFCC1
rs229460	21	20973470	0.2435	94.39%	0.408300	0.849100	0.03449000	0.11010000	NM_004540	upstream	601297	Hs.473450	NCAM2
rs2417970	12	21304001	0.2030	95.09%	0.850800	0.830200	0.03449000	0.03678000	NM_005075	downstream	15489	Hs.46440	SLCO1A2
rs285402	8	87110843	0.2225	79.65%	0.000392	0.016080	0.03449000	0.14170000	NM_033126	downstream	18964	Hs.680136	PSKH2
rs9317039	13	58484740	0.2299	96.14%	1.000000	1.000000	0.03449000	0.03592000	ENST00000364727	upstream	467464	---	---
rs2343116	4	158032128	0.1357	94.39%	0.797000	0.576400	0.03450000	0.01208000	NM_016205	intron	0	Hs.570855	PDGFC
rs10488769	11	110026148	0.2500	92.63%	0.870000	1.000000	0.03453000	0.04555000	NM_020809	intron	0	Hs.6136	ARHGAP20
rs275358	3	81551603	0.3664	91.93%	0.597100	0.734300	0.03453000	0.07914000	NM_000158	downstream	69939	Hs.436062	GBE1
rs857107	1	56995187	0.2273	96.49%	0.734100	0.843700	0.03457000	0.28560000	NM_001004303	intron	0	Hs.437655	C1orf168
rs2579884	8	35460189	0.1470	97.89%	0.339200	1.000000	0.03458000	0.05507000	ENST00000380061	intron	0	---	---
rs4237426	10	12790628	0.4771	91.93%	0.013030	0.125500	0.03458000	0.01706000	NM_153498	intron	0	Hs.659517	CAMK1D
rs7732805	5	28814401	0.1470	97.89%	0.339200	1.000000	0.03458000	0.11290000	ENST00000333399	upstream	147063	---	---
rs9309182	2	50530852	0.2887	83.86%	0.027580	0.527500	0.03458000	0.01597000	NM_138735	upstream	102482	Hs.637685	NRXN1
rs9285493	6	141022454	0.4431	86.32%	0.518400	0.336100	0.03459000	0.04062000	NM_006079	upstream	1284976	Hs.82071	CITED2
rs2289561	11	20076082	0.1240	91.93%	1.000000	0.766900	0.03461000	0.02798000	NM_145117	intron	0	---	NAV2
rs7003388	8	89459527	0.1487	97.89%	0.092770	0.188400	0.03463000	0.02832000	NM_022564	upstream	50635	Hs.546267	MMP16
rs337331	7	125733626	0.3651	97.54%	0.897000	0.498300	0.03464000	0.02713000	NM_000845	downstream	132783	Hs.449625	GRM8
rs10517390	4	36490528	0.3321	95.09%	1.000000	0.858400	0.03469000	0.00953000	ENST00000357504	downstream	468717	---	---
rs10511071	3	85882767	0.1375	94.39%	1.000000	0.703200	0.03470000	0.08975000	NM_153184	intron	0	Hs.164578	CADM2
rs2372665	3	81129513	0.2301	96.84%	0.172100	0.017660	0.03471000	0.03486000	NM_000158	downstream	492029	Hs.436062	GBE1
rs4885322	13	75069332	0.3040	97.54%	0.255900	1.000000	0.03471000	0.11040000	NM_006002	intron	0	Hs.162241	UCHL3

rs561454	4	74985590	0.4151	95.09%	0.901000	1.000000	0.03473000	0.05389000	NM_001511	downstream	17341	Hs.789	CXCL1
rs10519822	5	124925188	0.0498	91.58%	0.480500	0.516100	0.03474000	0.14390000	NM_023927	upstream	861812	Hs.363558	GRAMD3
rs2132966	11	20367216	0.2490	92.28%	0.620400	1.000000	0.03474000	0.02366000	NM_005788	intron	0	Hs.152337	PRMT3
rs7527085	1	98025993	0.1475	97.54%	0.089470	0.060190	0.03474000	0.03828000	NM_000110	intron	0	Hs.335034	DPYD
rs1921650	2	224062927	0.1991	79.30%	0.037930	0.036260	0.03479000	0.01905000	NM_003469	downstream	106979	Hs.516726	SCG2
rs1329710	6	45077103	0.4549	93.33%	0.324700	0.225900	0.03480000	0.01720000	NM_181356	intron	0	Hs.368325	SUPT3H
rs10493152	1	50901365	0.1027	90.53%	0.738200	1.000000	0.03483000	0.12760000	NM_007051	intron	0	Hs.530402	FAF1
rs293966	11	26536069	0.0572	95.09%	0.208900	0.240700	0.03483000	0.13050000	NM_031418	intron	0	Hs.91791	TMEM16C
rs3929856	13	18867056	0.2500	89.82%	0.617300	1.000000	0.03483000	0.03414000	NM_199254	downstream	28146	Hs.377488	TPTE2
rs7817589	8	82316787	0.1347	85.96%	0.022880	0.108700	0.03483000	0.02772000	NM_001444	upstream	38539	Hs.408061	FABP5
rs797054	3	30776179	0.4238	94.39%	0.319000	0.289100	0.03483000	0.01726000	NM_207359	intron	0	Hs.657052	GADL1
rs10487625	7	143768517	0.4363	93.68%	0.384700	0.116100	0.03484000	0.01556000	ENST00000385576	upstream	11201	---	---
rs10501896	11	98406293	0.1727	97.54%	0.056880	0.063070	0.03484000	0.01475000	ENST00000388171	upstream	89835	---	---
rs9291745	5	61327157	0.3435	91.93%	0.013690	0.019100	0.03484000	0.01260000	NM_004520	upstream	310678	Hs.558351	KIF2A
rs574545	6	94894667	0.4401	84.91%	0.036830	0.020930	0.03485000	0.10610000	ENST00000364082	downstream	249001	---	---
rs10489265	1	171502688	0.2753	93.68%	0.542800	0.531800	0.03486000	0.00797100	NM_003326	upstream	59594	Hs.181097	TNFSF4
rs413206	5	35405210	0.1448	88.42%	0.316200	0.574600	0.03486000	0.01622000	NM_000949	upstream	138876	Hs.368587	PRLR
rs10514646	2	84922143	0.1808	95.09%	0.014070	0.010560	0.03487000	0.08723000	ENST00000335459	intron	0	---	---
rs10511622	9	16586071	0.1111	97.89%	0.221300	0.367000	0.03488000	0.00933500	NM_017637	intron	0	Hs.656581	BNC2
rs1513215	3	39561067	0.0215	81.75%	1.000000	1.000000	0.03489000	0.99780000	NM_182935	downstream	28571	Hs.121333	MOBP
rs1566358	1	211729368	0.4086	94.04%	0.899900	1.000000	0.03489000	0.05084000	ENST00000331583	upstream	60326	---	---
rs3002309	1	212611255	0.2879	92.63%	0.548300	0.233000	0.03490000	0.01389000	NM_005401	intron	0	Hs.696573	PTPN14
rs9315112	13	30594502	0.2264	89.12%	0.074670	0.084130	0.03491000	0.11100000	NM_006644	downstream	14263	Hs.36927	HSPH1
rs10492213	12	105819971	0.1386	93.68%	0.797100	0.260300	0.03493000	0.10220000	NM_018157	downstream	14781	Hs.131306	RIC8B
rs2048988	2	170860831	0.2312	88.77%	0.012260	0.331800	0.03494000	0.06773000	NM_138995	intron	0	Hs.671900	MYO3B
rs10500912	11	21569166	0.0597	94.04%	1.000000	0.270200	0.03495000	0.04478000	NM_006157	downstream	15363	Hs.657172	NELL1
rs10517551	4	40538045	0.0967	96.14%	0.152100	0.709800	0.03496000	0.06093000	NM_173075	intron	0	Hs.479602	APBB2
rs709274	2	239996908	0.2248	97.54%	0.493000	0.551500	0.03496000	0.19940000	NM_006037	upstream	9328	Hs.20516	HDAC4
rs737939	22	28645416	0.4380	93.33%	0.619800	0.760700	0.03497000	0.04137000	NM_021090	intron	0	Hs.474536	MTMR3
rs10483865	14	74901236	0.2309	96.49%	1.000000	0.644200	0.03499000	0.02676000	NM_130469	upstream	63577	Hs.699420	JDP2
rs10515610	5	147733282	0.2663	96.84%	0.645700	0.858200	0.03499000	0.01794000	NM_205836	upstream	10457	Hs.483772	FBXO38
rs10520842	5	16679717	0.0730	93.68%	1.000000	1.000000	0.03500000	0.07838000	NM_012334	downstream	38696	Hs.481720	MYO10
rs1916503	10	57119488	0.3620	97.89%	0.603900	0.735500	0.03502000	0.04839000	NM_001005414	downstream	667717	---	ZWINT
rs6735587	2	125754892	0.3094	97.54%	0.778800	0.505500	0.03502000	0.08674000	NM_016815	upstream	1409652	Hs.59138	GYPC
rs10494545	1	180467282	0.2481	94.04%	0.189200	0.378900	0.03504000	0.02875000	NM_001009992	upstream	169812	Hs.684328	ZNF648
rs11066232	12	111203549	0.1715	97.19%	0.674600	1.000000	0.03505000	0.03541000	NM_173813	upstream	7609	---	C12orf51
rs17066851	8	3504744	0.0321	92.98%	1.000000	1.000000	0.03505000	0.01228000	NM_033225	upstream	250208	Hs.571466	CSMD1
rs10490954	10	11178054	0.3585	92.98%	0.183700	0.169700	0.03507000	0.07373000	NM_001025077	upstream	69402	Hs.309288	CUGBP2
rs10498638	14	93004188	0.2111	94.74%	0.360000	0.803300	0.03508000	0.03354000	NM_020818	intron	0	Hs.126561	KIAA1409
rs957438	11	84279025	0.3065	97.89%	0.887900	0.867800	0.03508000	0.09833000	ENST00000376104	intron	0	---	---
rs864391	3	45422021	0.4007	95.44%	0.448400	0.442900	0.03511000	0.28460000	NM_015340	intron	0	Hs.526975	LARS2
rs4632028	13	52504516	0.1764	96.49%	0.534500	1.000000	0.03512000	0.10280000	NM_006418	intron	0	Hs.559736	OLFM4
rs278411	3	74330763	0.2529	92.28%	1.000000	0.268900	0.03514000	0.06292000	NM_020872	downstream	63649	Hs.12723	CNTN3
rs2043556	10	52729412	0.4419	93.68%	0.171800	0.041380	0.03516000	0.13470000	NM_006258	intron	0	Hs.654556	PRKG1
rs3823572	7	133331141	0.3175	96.14%	0.332600	0.409300	0.03520000	0.09319000	NM_001037126	intron	0	Hs.321273	EXOC4
rs678134	2	220288792	0.4167	94.74%	0.531900	0.650900	0.03522000	0.02463000	ENST00000355668	upstream	14840	---	---
rs409620	2	40231848	0.3705	97.54%	0.028370	0.064080	0.03524000	0.28430000	NM_021097	intron	0	Hs.468274	SLC8A1
rs967895	2	111517873	0.3705	97.54%	0.519900	0.866700	0.03524000	0.00736900	NM_018308	intron	0	---	ACOXL
rs1979546	3	151327247	0.2124	93.33%	0.464400	0.405100	0.03525000	0.01969000	ENST00000386021	upstream	48939	---	---
rs1839800	11	4397360	0.2381	95.79%	0.316500	0.113000	0.03530000	0.05074000	NM_003141	upstream	25858	Hs.532357	TRIM21
rs10510820	3	59929425	0.1434	90.53%	0.199600	0.090750	0.03531000	0.03306000	NM_002012	intron	0	Hs.655995	FHIT
rs2246981	2	29792132	0.2103	88.42%	1.000000	0.605200	0.03534000	0.07020000	NM_004304	intron	0	Hs.654469	ALK
rs10515619	5	148155297	0.0979	66.32%	0.019760	0.024360	0.03535000	0.07829000	NM_000024	upstream	29704	Hs.591251	ADRB2
rs181586	10	119258198	0.3821	80.35%	0.124300	0.308600	0.03539000	0.08053000	NM_004098	upstream	33748	Hs.202095	EMX2

rs10521042	16	6339838	0.0953	97.54%	1.000000	0.476000	0.03540000	0.03790000	NM_001013705	upstream	27927	---	LOC440337
rs9315113	13	30619220	0.3321	94.04%	0.681000	0.856100	0.03545000	0.03577000	NM_006644	intron	0	Hs.36927	HSPH1
rs715285	5	131513282	0.3774	91.58%	0.895100	1.000000	0.03547000	0.03554000	NM_001017973	downstream	42920	Hs.519568	P4HA2
rs1981360	18	29496909	0.3966	93.33%	0.020980	0.116900	0.03549000	0.44000000	NM_198995	upstream	222226	Hs.115461	C18orf34
rs10489015	4	26982600	0.0989	95.79%	0.159200	0.717600	0.03551000	0.03256000	ENST00000363691	upstream	148453	---	---
rs10495064	1	215896812	0.1320	87.72%	0.780500	0.696400	0.03553000	0.04572000	NM_138796	intron	0	Hs.171130	SPATA17
rs210236	10	42332699	0.1751	97.19%	0.532500	0.551300	0.03554000	0.18000000	ENST00000374537	downstream	2386	---	---
rs2038479	1	170205913	0.1899	83.16%	0.032000	0.099720	0.03555000	0.22200000	NM_015569	intron	0	Hs.654775	DNM3
rs980222	13	100888302	0.2011	96.84%	0.851100	0.606400	0.03557000	0.04444000	NM_004791	upstream	14555	Hs.696554	ITGBL1
rs10501590	11	84620725	0.2719	96.14%	0.092270	0.535800	0.03558000	0.10940000	ENST00000376104	intron	0	---	---
rs2383713	9	26763231	0.0529	89.47%	0.516300	0.554100	0.03558000	0.10990000	NM_024828	downstream	67452	Hs.178357	C9orf82
rs693819	1	28265882	0.0223	78.60%	1.000000	1.000000	0.03561000	0.01899000	NM_001990	intron	0	Hs.185774	EYA3
rs561637	11	89992350	0.3968	88.42%	0.359900	0.340100	0.03564000	0.03603000	NM_012124	upstream	396523	Hs.22857	CHORDC1
rs722341	11	17429722	0.1015	93.33%	0.740100	0.720700	0.03566000	0.02775000	NM_000352	intron	0	Hs.54470	ABCC8
rs2315894	13	54866889	0.2940	93.68%	0.769400	0.845200	0.03567000	0.03659000	ENST00000258651	upstream	953438	---	---
rs3897767	5	144661751	0.1462	91.23%	0.130000	0.474600	0.03567000	0.09318000	NM_138492	downstream	457024	Hs.314261	PRELID2
rs2867698	4	81220964	0.0887	92.98%	1.000000	1.000000	0.03568000	0.06084000	NM_058172	upstream	8223	Hs.162963	ANTXR2
rs1456017	2	46761131	0.2460	66.32%	0.078080	0.115800	0.03569000	0.02246000	NM_144949	upstream	18464	Hs.468426	SOCS5
rs10517686	4	159262079	0.0407	90.53%	0.056800	0.104100	0.03570000	0.06783000	NM_016613	downstream	5930	Hs.567498	C4orf18
rs9318757	13	80711441	0.3547	90.53%	0.221900	0.509700	0.03571000	0.01635000	ENST00000387380	upstream	26442	---	---
rs995653	9	106840914	0.3235	89.47%	0.021540	0.064090	0.03571000	0.11880000	NM_005502	upstream	110575	Hs.429294	ABCA1
rs1834476	7	49802856	0.0650	86.32%	1.000000	1.000000	0.03572000	0.03025000	NM_198570	intron	0	Hs.677488	VWC2
rs10513311	9	119648541	0.3850	96.14%	0.375000	0.098310	0.03574000	0.02000000	NM_138554	downstream	129571	Hs.174312	TLR4
rs141014	1	55071727	0.1996	97.54%	0.708600	0.400000	0.03576000	0.09015000	NM_152607	intron	0	---	C1orf177
rs188541	5	37097018	0.3725	89.47%	1.000000	0.384900	0.03576000	0.00564500	NM_015384	intron	0	Hs.481927	NIPBL
rs3777425	6	116385510	0.3395	95.09%	1.000000	0.198300	0.03576000	0.09653000	NM_002031	intron	0	Hs.89426	FRK
rs6847164	4	120754182	0.0950	97.89%	0.025320	0.014800	0.03576000	0.06738000	NM_033437	intron	0	Hs.647971	PDE5A
rs10512626	3	125802903	0.1827	95.09%	1.000000	0.647700	0.03578000	0.06627000	NM_007064	intron	0	Hs.8004	KALRN
rs10516386	4	21088565	0.0668	86.67%	0.080770	0.320700	0.03578000	0.06831000	NM_025221	intron	0	Hs.655705	KCNIP4
rs732289	3	152119035	0.2966	94.04%	0.190300	0.336900	0.03578000	0.01397000	NM_052995	downstream	7605	Hs.380222	CLRN1
rs1797626	3	114308943	0.3433	94.04%	0.225300	0.482400	0.03582000	0.00977200	NM_033254	upstream	105122	Hs.591318	BOC
rs4131756	12	24204836	0.2233	72.28%	0.313200	0.823400	0.03582000	0.05719000	NM_144667	downstream	422984	Hs.350668	FLJ32894
rs556968	11	105339334	0.3470	94.04%	0.500100	0.223900	0.03582000	0.08483000	NM_000829	intron	0	Hs.503743	GRIA4
rs1357194	6	115019312	0.1309	96.49%	1.000000	0.227600	0.03586000	0.04301000	ENST00000386065	downstream	275452	---	---
rs4903076	14	39382590	0.3223	95.79%	0.071580	0.323300	0.03586000	0.04113000	NM_203301	upstream	411219	Hs.324342	FBXO33
rs10506292	12	49031020	0.2519	94.04%	0.517900	1.000000	0.03587000	0.03491000	ENST00000380174	downstream	2674	---	---
rs1252041	9	119484618	0.1204	94.74%	0.777900	0.382100	0.03587000	0.02521000	NM_138554	upstream	21853	Hs.174312	TLR4
rs10487275	7	107197515	0.3515	93.33%	0.282100	0.488100	0.03588000	0.31710000	NM_000111	intron	0	Hs.1650	SLC26A3
rs10493632	1	80227457	0.0195	89.82%	0.085660	1.000000	0.03593000	0.09245000	ENST00000294631	upstream	982507	---	---
rs664815	1	225358116	0.3457	94.39%	0.344800	0.751100	0.03594000	0.16120000	NM_003607	intron	0	Hs.35433	CDC42BPA
rs1468786	7	12050906	0.4547	92.98%	1.000000	0.761700	0.03595000	0.07221000	NM_018374	upstream	170047	Hs.396358	TMEM106B
rs10488272	7	83672266	0.0957	97.19%	1.000000	1.000000	0.03596000	0.05776000	NM_006080	upstream	10421	Hs.252451	SEMA3A
rs10507887	13	77885957	0.1757	96.84%	0.147200	0.770200	0.03597000	0.08495000	NM_006237	downstream	185271	Hs.654522	POU4F1
rs3868879	3	114254158	0.2126	86.67%	0.034760	0.008483	0.03597000	0.04965000	NM_033254	upstream	159907	Hs.591318	BOC
rs1870016	4	31206602	0.0480	95.09%	1.000000	1.000000	0.03598000	0.04053000	NM_032457	downstream	453033	Hs.570785	PCDH7
rs1877269	3	170109714	0.4683	94.04%	0.027010	0.021020	0.03598000	0.07333000	NM_005241	downstream	175530	Hs.656395	EVI1
rs2199076	12	73809319	0.2088	87.37%	0.248500	0.604600	0.03598000	0.18770000	NM_153748	intron	0	Hs.591041	KCNC2
rs702458	7	124994203	0.0480	95.09%	0.467100	1.000000	0.03598000	0.01337000	NM_001042594	upstream	637093	Hs.31968	POT1
rs2089190	5	171972970	0.4850	93.33%	0.461200	1.000000	0.03599000	0.02571000	ENST00000355361	upstream	3929	---	---
rs637074	12	51395424	0.4408	85.96%	0.001784	0.071760	0.03600000	0.06104000	NM_175078	upstream	11910	Hs.334989	KRT77
rs10496583	2	122835383	0.3566	92.98%	0.351500	0.384300	0.03602000	0.12450000	NM_004622	downstream	595970	Hs.75066	TSN
rs2347713	7	134065068	0.3794	90.18%	0.691100	0.523100	0.03603000	0.01689000	NM_033140	upstream	49643	Hs.490203	CALD1
rs4376787	1	96172071	0.4517	94.39%	0.048910	0.120400	0.03605000	0.04900000	ENST00000363930	upstream	291974	---	---
rs10496245	2	80940209	0.4596	91.23%	0.169600	0.337400	0.03607000	0.02576000	NM_004389	downstream	211701	Hs.167368	CTNNA2

rs5543	2	40195109	0.2381	95.79%	0.617300	0.496600	0.03607000	0.06921000	NM_021097	3UTR	0	Hs.468274	SLC8A1
rs10515366	5	106068927	0.1308	97.89%	1.000000	1.000000	0.03609000	0.03986000	NM_001962	downstream	671562	Hs.658451	EFNA5
rs200145	6	143385463	0.1308	97.89%	0.796100	1.000000	0.03609000	0.05972000	NM_016108	upstream	38243	Hs.567501	AIG1
rs942835	1	120064541	0.1308	97.89%	0.440100	0.573700	0.03609000	0.09387000	NM_006623	intron	0	Hs.487296	PHGDH
rs1358091	2	184735915	0.2664	90.88%	1.000000	1.000000	0.03610000	0.02510000	NM_194250	upstream	436017	Hs.159528	ZNF804A
rs3119882	13	60263985	0.4979	84.91%	0.608500	0.424400	0.03611000	0.13600000	ENST00000384127	downstream	2191	---	---
rs2236158	20	17882961	0.2481	94.74%	0.744000	0.452000	0.03612000	0.13690000	NM_014426	intron	0	Hs.316890	SNX5
rs1701586	18	59645607	0.1136	95.79%	0.003057	0.093670	0.03614000	0.48710000	NM_001040147	downstream	22024	Hs.138202	SERPINB7
rs9292166	5	57867126	0.1377	92.98%	0.300300	0.040380	0.03614000	0.06355000	NM_152687	downstream	39184	Hs.547697	C5orf29
rs2339686	10	52436044	0.3165	97.54%	1.000000	0.855300	0.03615000	0.10770000	ENST00000373985	intron	0	---	---
rs10494190	1	117194463	0.1318	97.19%	0.797500	0.575700	0.03618000	0.01800000	NM_020440	upstream	59739	Hs.418093	PTGFRN
rs1378147	18	67302947	0.3728	97.89%	0.160100	0.121200	0.03618000	0.01561000	NM_182511	downstream	1051948	Hs.569851	CBLN2
rs1338787	13	54688732	0.2820	93.33%	0.047950	0.003660	0.03619000	0.07075000	ENST00000258651	upstream	775281	---	---
rs1572239	6	155658406	0.3740	83.51%	0.003527	0.004681	0.03620000	0.09460000	NM_016020	intron	0	Hs.279908	TFB1M
rs278465	3	74300577	0.2500	94.74%	0.516000	0.076730	0.03620000	0.06279000	NM_020872	downstream	93835	Hs.12723	CNTN3
rs1918891	2	228870980	0.2338	97.54%	0.507700	1.000000	0.03621000	0.14450000	NM_030623	upstream	116394	Hs.436306	SPHKAP
rs7005262	8	14903851	0.4426	94.74%	0.217900	0.755700	0.03621000	0.26940000	NM_139167	intron	0	Hs.676196	SGCZ
rs10493115	1	43479870	0.1903	94.04%	0.425700	0.788700	0.03622000	0.03226000	ENST00000315972	CDS	0	---	---
rs725040	13	91066847	0.3763	97.89%	0.898900	0.618600	0.03622000	0.06759000	NM_004466	intron	0	Hs.655675	GPC5
rs10483251	14	20741117	0.4480	94.39%	1.000000	0.276800	0.03624000	0.12070000	NM_004500	downstream	7440	Hs.508848	HNRNPC
rs10515460	5	132718332	0.1345	96.49%	1.000000	0.776500	0.03624000	0.02875000	NM_015082	intron	0	Hs.483390	FSTL4
rs10520213	4	173158174	0.0229	76.49%	1.000000	1.000000	0.03624000	0.07298000	NM_001034845	intron	0	Hs.386236	GALNT17
rs10505781	12	14104370	0.1412	91.93%	0.317700	0.170200	0.03625000	0.04629000	NM_000834	upstream	80051	Hs.654430	GRIN2B
rs9307048	4	89368594	0.1935	91.58%	0.016730	0.013470	0.03625000	0.36400000	ENST00000362456	downstream	1589	---	---
rs2060	1	215560722	0.3521	93.68%	0.286100	0.484400	0.03626000	0.05544000	NM_018040	downstream	109735	Hs.420757	GPATCH2
rs2321996	13	58577337	0.2197	92.63%	0.594900	1.000000	0.03627000	0.04008000	ENST00000364727	upstream	374867	---	---
rs10504009	8	37458281	0.1842	93.33%	0.838600	0.819400	0.03628000	0.13730000	NM_021631	downstream	592295	Hs.651853	FKSG2
rs295603	11	55187106	0.1152	85.26%	0.000000	0.000001	0.03629000	0.11190000	NM_001004704	intron	0	Hs.706616	OR4C6
rs10517612	4	156145501	0.0188	93.33%	1.000000	1.000000	0.03632000	0.02266000	NM_144979	downstream	176087	Hs.133095	RBM46
rs1945873	11	91315531	0.3212	96.14%	0.677500	0.713800	0.03632000	0.15260000	NM_012124	upstream	1719704	Hs.22857	CHORDC1
rs10483454	14	35046027	0.1636	94.39%	0.659500	1.000000	0.03633000	0.04386000	NM_032594	upstream	26972	Hs.62813	INSM2
rs3798393	6	117838661	0.3577	86.32%	0.001438	0.021960	0.03633000	0.01864000	NM_002944	intron	0	Hs.1041	ROS1
rs2712767	18	1815170	0.4000	89.47%	0.241500	0.114800	0.03635000	0.01582000	NM_022840	downstream	712360	Hs.126888	METTL4
rs347311	1	160570531	0.3136	97.89%	0.331100	0.355000	0.03636000	0.05849000	NM_014697	intron	0	Hs.655000	NOS1AP
rs10489252	1	170014768	0.1275	89.47%	0.092830	0.129900	0.03641000	0.04511000	NM_001007239	upstream	2691	Hs.494705	KIAA0859
rs1390593	8	50759834	0.1336	97.19%	0.438300	1.000000	0.03643000	0.03101000	NM_018967	upstream	709492	Hs.584914	SNTG1
rs978178	1	49962719	0.3534	93.33%	0.502000	0.485000	0.03645000	0.10220000	ENST00000334103	intron	0	---	---
rs1628472	3	117717496	0.2688	97.89%	0.285200	0.155400	0.03646000	0.04955000	NM_002338	upstream	70928	Hs.657246	LSAMP
rs2078542	2	148122694	0.2688	97.89%	0.365100	0.155400	0.03646000	0.00325300	NM_001616	upstream	196373	Hs.470174	ACVR2A
rs10518871	15	54805516	0.1077	91.23%	0.193900	1.000000	0.03647000	0.02712000	NM_001002843	intron	0	Hs.511477	ZNF280D
rs2884516	4	150122279	0.1389	94.74%	0.069640	0.258300	0.03647000	0.18130000	ENST00000365396	downstream	9894	---	---
rs10502606	18	28561872	0.2948	94.04%	0.659100	0.167300	0.03649000	0.20000000	NM_020805	intron	0	Hs.446164	KLHL14
rs710588	3	191301129	0.2566	93.68%	0.151300	0.670400	0.03649000	0.02600000	NM_018192	intron	0	Hs.374191	LEPREL1
rs10492214	12	105818888	0.1445	92.28%	0.082100	0.584100	0.03651000	0.06494000	NM_018157	downstream	13698	Hs.131306	RIC8B
rs10508751	10	30294004	0.0189	92.98%	0.082830	0.008646	0.03651000	0.03044000	NM_021738	upstream	330097	Hs.499209	SVIL
rs10514474	16	78548724	0.0187	94.04%	1.000000	1.000000	0.03651000	0.01201000	NM_001031804	upstream	356612	Hs.134859	MAF
rs495860	6	125438744	0.0187	94.04%	0.081920	1.000000	0.03651000	0.10230000	NM_152553	intron	0	Hs.368639	RNF217
rs2459442	10	37332756	0.3781	97.89%	0.373300	0.759500	0.03654000	0.00888900	NM_052997	upstream	122035	Hs.373787	ANKRD30A
rs1558699	7	77930588	0.4944	93.68%	0.625500	0.451200	0.03656000	0.12100000	NM_012301	intron	0	Hs.654788	MAGI2
rs3757798	7	117659225	0.0618	93.68%	0.259600	1.000000	0.03656000	0.13910000	NM_019644	intron	0	Hs.657737	ANKRD7
rs6686086	1	191902023	0.4134	89.12%	1.000000	0.532300	0.03657000	0.04862000	NM_024529	downstream	414344	Hs.576497	CDC73
rs6984320	8	77352569	0.1313	97.54%	0.440200	0.772100	0.03657000	0.02079000	NM_024721	upstream	426266	Hs.458973	ZFHX4
rs10520506	4	182863700	0.2472	95.09%	0.871000	1.000000	0.03661000	0.38340000	ENST00000378957	downstream	182552	---	---
rs2075397	12	113053753	0.2670	97.89%	0.759800	0.413200	0.03661000	0.06907000	NM_016196	upstream	165259	Hs.7482	RBM19

rs2383143	9	20639540	0.2127	94.04%	0.855100	0.206100	0.03661000	0.12100000	NM_017794	upstream	8768	Hs.408652	KIAA1797
rs10506589	12	69013265	0.1203	93.33%	1.000000	0.668400	0.03662000	0.10360000	NM_014515	intron	0	Hs.133350	CNOT2
rs2829746	21	25719960	0.0332	89.82%	1.000000	1.000000	0.03663000	0.03987000	ENST00000332587	downstream	4125	---	---
rs6574548	14	79690049	0.1129	97.89%	0.367900	0.528400	0.03663000	0.04564000	NM_001007023	downstream	46546	Hs.202354	DIO2
rs4061659	17	1944194	0.3822	96.84%	0.251300	0.409300	0.03664000	0.07737000	NM_017575	intron	0	Hs.448342	SMG6
rs10514925	17	42799867	0.0874	94.39%	0.436200	0.568700	0.03666000	0.07775000	NM_152347	intron	0	Hs.463303	C17orf57
rs628926	9	119641627	0.3120	82.11%	0.000007	0.000028	0.03666000	0.03637000	NM_138554	downstream	122657	Hs.174312	TLR4
rs7094169	10	12127660	0.4918	85.96%	0.249800	0.264700	0.03667000	0.03143000	NM_015542	upstream	2821	Hs.370689	UPF2
rs10251209	7	49584973	0.3030	81.05%	0.121100	0.204500	0.03670000	0.06502000	NM_198570	upstream	198825	Hs.677488	VWC2
rs10517210	4	30253178	0.1429	93.33%	0.801200	1.000000	0.03670000	0.08007000	NM_032457	upstream	78965	Hs.570785	PCDH7
rs10488127	7	10291095	0.0185	94.74%	1.000000	1.000000	0.03671000	0.03752000	ENST00000387411	downstream	61932	---	---
rs4140470	20	7155479	0.4557	95.09%	0.625300	0.367800	0.03672000	0.03390000	NM_001200	downstream	446552	Hs.73853	BMP2
rs10489160	1	22445846	0.1788	96.14%	0.213900	0.816300	0.03673000	0.12400000	NM_030761	upstream	103808	Hs.25766	WNT4
rs720313	11	119425320	0.1788	96.14%	0.679500	0.485600	0.03673000	0.13120000	NM_012101	downstream	61885	Hs.504115	TRIM29
rs723363	4	111943950	0.3172	97.89%	0.169100	0.510700	0.03673000	0.01708000	NM_000325	upstream	180247	Hs.643588	PITX2
rs6560488	9	77845747	0.4721	94.39%	0.625500	0.764300	0.03674000	0.05747000	NM_006200	intron	0	Hs.368542	PCSK5
rs8018273	14	86867956	0.4906	93.68%	1.000000	0.222900	0.03674000	0.05428000	NM_001037525	downstream	601157	Hs.513439	GALC
rs3804338	6	106879466	0.2500	94.04%	0.102000	0.265600	0.03675000	0.04542000	NM_004849	intron	0	Hs.486063	ATG5
rs10518812	15	41440229	0.2054	84.56%	0.435000	0.496200	0.03676000	0.02436000	NM_152455	downstream	279	Hs.418287	ZSCAN29
rs1992331	8	62438596	0.4868	92.98%	0.269600	0.540800	0.03676000	0.00117600	NM_173519	intron	0	Hs.654963	RLBP1L1
rs1287293	1	236434231	0.1159	96.84%	0.771300	0.749400	0.03677000	0.02045000	ENST00000385340	downstream	121379	---	---
rs955779	10	53613893	0.2706	97.89%	0.649500	1.000000	0.03678000	0.04373000	NM_006258	intron	0	Hs.654556	PRKG1
rs788063	10	29301503	0.1022	94.39%	0.171300	0.145300	0.03679000	0.08271000	NM_032517	upstream	316493	Hs.558572	LYZL1
rs604844	6	154496665	0.2235	92.63%	0.859000	0.312800	0.03680000	0.05533000	NM_001008503	intron	0	Hs.2353	OPRM1
rs2072464	2	20303107	0.2885	79.65%	0.106000	0.195800	0.03681000	0.30460000	NM_015317	downstream	8826	Hs.467824	PUM2
rs4072139	10	62327362	0.3721	91.93%	0.354500	0.866200	0.03683000	0.12190000	NM_001032380	intron	0	Hs.148670	RHOBTB1
rs541763	1	120046930	0.3569	94.39%	0.791500	0.731400	0.03684000	0.02729000	NM_006623	upstream	9103	Hs.487296	PHGDH
rs1436136	4	113282975	0.3607	91.93%	1.000000	0.871800	0.03685000	0.11090000	NM_152400	upstream	3152	Hs.23439	C4orf32
rs957574	21	22054437	0.4613	95.09%	0.141900	0.100600	0.03686000	0.22190000	ENST00000358903	downstream	115964	---	---
rs10507889	13	77917117	0.2683	90.88%	0.272400	0.297700	0.03688000	0.02499000	NM_006237	downstream	154111	Hs.654522	POU4F1
rs10515283	5	98121571	0.1255	90.88%	0.258800	0.213200	0.03688000	0.06257000	NM_173670	upstream	11329	Hs.526902	RGMB
rs10486010	7	97316254	0.1545	96.49%	0.817800	1.000000	0.03689000	0.11150000	NM_183356	downstream	3125	Hs.489207	ASNS
rs7829511	8	84033179	0.3863	97.19%	0.526500	0.622900	0.03689000	0.02442000	NM_152837	upstream	1116189	Hs.492121	SNX16
rs10490861	3	7004628	0.1686	91.58%	0.006596	0.052310	0.03690000	0.19590000	NM_181874	intron	0	Hs.660131	GRM7
rs10504743	8	82951580	0.1199	95.09%	0.392400	1.000000	0.03690000	0.10010000	NM_152837	upstream	34590	Hs.492121	SNX16
rs10510576	3	26450054	0.1756	97.89%	0.678700	0.166300	0.03690000	0.05535000	NM_052953	upstream	189247	Hs.517868	LRRC3B
rs10512009	9	72991086	0.3835	97.89%	0.043490	0.220800	0.03690000	0.05705000	NM_206945	intron	0	Hs.47288	TRPM3
rs7685129	4	17912310	0.2106	95.79%	0.717500	0.679700	0.03690000	0.03740000	NM_153686	upstream	279713	Hs.446201	LCORL
rs10518335	4	120741620	0.1222	93.33%	0.249000	0.132200	0.03691000	0.06124000	NM_033437	intron	0	Hs.647971	PDE5A
rs10520248	4	174086924	0.0682	84.91%	1.000000	1.000000	0.03691000	0.09151000	NM_001034845	intron	0	Hs.386236	GALNT17
rs6962216	7	144488779	0.2044	96.14%	0.711200	0.673300	0.03692000	0.01571000	ENST00000363803	upstream	310669	---	---
rs10498452	14	53304673	0.1033	95.09%	1.000000	1.000000	0.03693000	0.03256000	NM_130851	downstream	181534	Hs.68879	BMP4
rs914959	1	97413151	0.3577	93.68%	0.045120	0.119400	0.03694000	0.06054000	NM_000110	intron	0	Hs.335034	DPYD
rs315499	17	27045434	0.3136	82.81%	0.048480	0.031910	0.03696000	0.08394000	ENST00000365156	upstream	19451	---	---
rs10501535	11	82011466	0.1155	97.19%	0.145900	0.206300	0.03698000	0.04702000	ENST00000357859	upstream	66783	---	---
rs1403439	3	34878928	0.4668	95.09%	0.331100	0.296400	0.03698000	0.30960000	NM_001025069	upstream	779925	Hs.475902	ARPP-21
rs10491916	9	9875311	0.0379	97.19%	1.000000	1.000000	0.03699000	0.11310000	ENST00000363183	downstream	442959	---	---
rs10516901	4	92899736	0.1699	90.88%	0.508000	0.806400	0.03703000	0.08859000	NM_207491	downstream	160505	Hs.654735	MGC48628
rs357749	2	71225997	0.3500	94.74%	0.688000	0.635100	0.03705000	0.10410000	NM_005791	intron	0	Hs.656208	MPHOSPH10
rs9790802	4	27145627	0.3327	96.49%	0.135800	0.157400	0.03705000	0.05086000	ENST00000363691	upstream	311480	---	---
rs1351232	3	144051683	0.4593	94.74%	0.328000	0.877800	0.03706000	0.24580000	NM_013363	intron	0	Hs.8944	PCOLCE2
rs7911323	10	122047679	0.1558	96.84%	0.820900	1.000000	0.03706000	0.29690000	ENST00000330207	downstream	56738	---	---
rs841122	5	87090681	0.4539	95.09%	0.327100	0.093530	0.03706000	0.02092000	NM_153354	downstream	436100	Hs.379972	TMEM161B
rs10516102	5	173018153	0.0451	85.61%	0.075030	0.132600	0.03709000	0.01728000	NM_138369	upstream	41891	Hs.425091	FAM44B

rs10504762	8	83590018	0.1340	87.72%	0.001244	0.034040	0.03710000	0.03102000	NM_152837	upstream	673028	Hs.492121	SNX16
rs10513637	3	22177217	0.2183	94.04%	0.150300	0.157100	0.03714000	0.07698000	NM_024697	upstream	409397	Hs.21026	ZNF385D
rs10501430	11	78052837	0.4742	95.09%	0.051520	0.097430	0.03717000	0.01350000	ENST00000278550	intron	0	---	---
rs26992	5	65459341	0.2716	97.54%	1.000000	0.837900	0.03717000	0.01682000	NM_001006600	downstream	46735	Hs.591774	ERBB2IP
rs744318	15	55669181	0.3438	95.44%	0.423500	1.000000	0.03717000	0.06630000	NM_001018091	upstream	2225	Hs.437256	Gcom1
rs9316974	13	57658355	0.3284	95.09%	0.784300	1.000000	0.03718000	0.06439000	ENST00000365552	downstream	194757	---	---
rs10502228	11	117618793	0.2782	96.49%	0.550500	0.841900	0.03719000	0.03357000	NM_198275	intron	0	Hs.15396	MPZL3
rs10502887	18	43433881	0.3668	85.61%	0.053430	0.048680	0.03720000	0.16700000	ENST00000328279	downstream	22660	---	---
rs7657136	4	182522209	0.3249	97.19%	0.217000	0.326200	0.03720000	0.12540000	ENST00000378957	upstream	158598	---	---
rs722726	12	113065706	0.3833	90.18%	0.000203	0.006393	0.03721000	0.04837000	NM_016196	upstream	177212	Hs.7482	RBM19
rs9308786	2	4607030	0.1423	93.68%	0.800800	0.276800	0.03724000	0.03565000	ENST00000387792	downstream	245436	---	---
rs2832296	21	29663325	0.2860	87.72%	0.008636	0.048270	0.03728000	0.02521000	NM_001011545	downstream	17404	Hs.154276	BACH1
rs2837780	21	40972828	0.4594	95.09%	0.066160	0.021760	0.03732000	0.12480000	NM_001389	intron	0	Hs.397800	DSCAM
rs997385	1	42094639	0.2199	93.33%	1.000000	0.413600	0.03732000	0.05612000	NM_024503	intron	0	Hs.591503	HIVEP3
rs1022311	2	180264129	0.1563	95.44%	0.493500	0.497000	0.03733000	0.04644000	NM_152520	intron	0	Hs.655005	ZNF385B
rs10494115	1	43479622	0.1877	94.39%	0.424100	0.782800	0.03733000	0.03884000	ENST00000372493	intron	0	---	---
rs2060415	8	109077209	0.4907	94.74%	0.808400	1.000000	0.03733000	0.07121000	NM_178565	intron	0	Hs.444834	RSPO2
rs293376	15	87436272	0.4907	94.74%	0.808400	0.763800	0.03733000	0.04778000	ENST00000352732	intron	0	---	---
rs245914	7	29184684	0.1458	92.63%	1.000000	0.283200	0.03734000	0.06437000	NM_004067	upstream	16167	Hs.654611	CHN2
rs10505141	8	111223962	0.0738	95.09%	0.644600	1.000000	0.03738000	0.14220000	NM_014379	upstream	167827	Hs.13285	KCNV1
rs10504205	8	57655013	0.4667	94.74%	0.027570	0.071540	0.03739000	0.13300000	NM_006211	upstream	133870	Hs.339831	PENK
rs10500925	11	22371525	0.2068	97.54%	0.855800	0.802800	0.03741000	0.03870000	NM_020346	downstream	13906	Hs.242821	SLC17A6
rs1764142	6	956570	0.1896	94.39%	0.844100	0.423100	0.03741000	0.19030000	NM_018303	upstream	318461	Hs.484412	EXOC2
rs2782451	13	68802710	0.4870	94.39%	0.273200	0.069330	0.03742000	0.04526000	NM_020866	downstream	370016	Hs.508201	KLHL1
rs10501004	11	25304107	0.0192	91.58%	1.000000	1.000000	0.03743000	0.14430000	NM_001009909	downstream	247327	Hs.144138	LUZP2
rs6083461	20	2424778	0.3688	92.28%	0.234700	0.733100	0.03744000	0.04761000	NM_024325	intron	0	Hs.516846	ZNF343
rs1543468	1	232415048	0.4333	89.47%	0.202700	0.749500	0.03745000	0.02288000	NM_173508	intron	0	Hs.158748	SLC35F3
rs601828	11	30017096	0.0057	91.93%	1.000000	1.000000	0.03746000	0.99880000	NM_002233	upstream	22032	Hs.592002	KCNA4
rs10507198	12	104625308	0.2154	93.68%	0.046130	0.132600	0.03747000	0.21450000	NM_207376	downstream	335883	---	OCC-1
rs3844166	1	77643728	0.0827	97.54%	1.000000	0.692700	0.03747000	0.07328000	NM_012093	intron	0	Hs.559718	AK5
rs10504842	8	89484318	0.2025	97.89%	0.194300	0.289200	0.03749000	0.06136000	NM_003821	upstream	1354855	Hs.103755	RIPK2
rs10512513	17	61965600	0.4981	94.74%	0.903100	0.651000	0.03749000	0.06048000	NM_002737	intron	0	Hs.531704	PRKCA
rs10514564	16	81298757	0.4981	94.74%	0.714900	0.450800	0.03749000	0.05274000	NM_001257	upstream	150703	Hs.654386	CDH13
rs34879254	6	46586309	0.2025	97.89%	0.194300	0.137800	0.03749000	0.32070000	NM_016593	downstream	39096	Hs.387367	CYP39A1
rs229336	2	149729617	0.1878	85.96%	0.003119	0.007863	0.03750000	0.07012000	NM_177964	intron	0	Hs.357567	LOC130576
rs850434	6	97342285	0.0960	87.72%	0.258300	1.000000	0.03750000	0.28940000	NM_030784	downstream	10360	Hs.632612	GPR63
rs10505183	8	113329508	0.1421	95.09%	0.040920	0.030470	0.03751000	0.12180000	NM_052900	intron	0	Hs.91381	CSMD3
rs7312252	12	49030438	0.2528	94.39%	0.522900	1.000000	0.03753000	0.03103000	ENST00000380174	downstream	3256	---	---
rs9301029	13	104896251	0.1807	96.14%	1.000000	0.770800	0.03755000	0.07456000	NM_172370	upstream	20342	Hs.381382	DAOA
rs10493379	1	65818515	0.3604	92.98%	1.000000	0.873600	0.03756000	0.17990000	NM_001003680	intron	0	Hs.705413	LEPR
rs1488939	4	56213901	0.3871	97.89%	0.129800	0.255800	0.03757000	0.24520000	NM_006681	upstream	16679	Hs.418367	NMU
rs1927964	9	89587150	0.2175	94.39%	0.375800	0.806100	0.03757000	0.00148500	NM_001023564	intron	0	Hs.690450	CTSL3
rs240165	6	101149947	0.0376	97.89%	1.000000	1.000000	0.03757000	0.07411000	NM_006828	intron	0	Hs.486031	ASCC3
rs4129606	7	70598829	0.0376	97.89%	0.323500	0.362100	0.03757000	0.05477000	NM_022479	intron	0	Hs.488591	WBSCR17
rs9287518	2	139879348	0.1227	94.39%	0.777600	1.000000	0.03757000	0.04052000	ENST00000384174	upstream	106116	---	---
rs10508408	10	10672427	0.2201	94.04%	0.722400	1.000000	0.03758000	0.05957000	NM_001025077	upstream	575029	Hs.309288	CUGBP2
rs7698530	4	73977981	0.2509	93.68%	0.050080	0.089440	0.03758000	0.24440000	NM_014243	upstream	324601	Hs.590919	ADAMTS3
rs10510793	3	57448790	0.0676	85.61%	0.002108	0.003379	0.03760000	0.16350000	ENST00000389536	intron	0	---	---
rs10513719	3	174992823	0.2831	95.44%	1.000000	0.226100	0.03760000	0.04826000	NM_014932	intron	0	Hs.478289	NLGN1
rs10520475	4	182468476	0.0858	96.14%	0.121300	0.102700	0.03760000	0.13710000	ENST00000378957	upstream	212331	---	---
rs210056	6	90729099	0.1173	97.19%	0.556600	0.660000	0.03760000	0.09992000	NM_021813	intron	0	Hs.269764	BACH2
rs2189813	7	86190735	0.1799	97.54%	0.839400	0.573700	0.03762000	0.12900000	NM_000840	intron	0	Hs.590575	GRM3
rs10509897	10	111221178	0.0243	93.68%	0.138900	0.184100	0.03763000	0.04786000	NM_020383	downstream	393336	Hs.390623	XPNPEP1
rs1390808	8	115601897	0.1056	94.74%	1.000000	0.352100	0.03764000	0.22100000	NM_014112	downstream	892074	Hs.657018	TRPS1

rs1283726	8	108545032	0.0486	97.54%	1.000000	1.000000	0.03767000	0.02462000	NM_001146	intron	0	Hs.369675	ANGPT1
rs740600	10	118641872	0.3187	91.93%	0.887600	0.741300	0.03767000	0.06729000	NM_018330	intron	0	Hs.501140	KIAA1598
rs10495045	1	215484461	0.2970	95.09%	0.771400	0.248500	0.03768000	0.05641000	NM_206595	upstream	154891	Hs.444225	ESRRG
rs1160595	2	50988845	0.2383	97.19%	0.623400	0.503900	0.03770000	0.06086000	ENST00000378259	upstream	80340	---	---
rs1342744	6	16160498	0.3561	95.09%	0.894700	1.000000	0.03770000	0.01485000	NM_013262	upstream	76798	Hs.484738	MYLIP
rs1566584	1	42570352	0.0609	95.09%	1.000000	1.000000	0.03771000	0.01966000	NM_014947	intron	0	Hs.26023	FOXJ3
rs10500747	11	11728465	0.3760	90.53%	0.145400	0.169600	0.03773000	0.09977000	NM_017944	upstream	91081	Hs.577256	USP47
rs1805731	12	8986493	0.3989	97.19%	0.008283	0.091300	0.03775000	0.05217000	NM_002355	intron	0	Hs.134084	M6PR
rs10517265	4	32784855	0.0714	95.79%	0.376500	0.366900	0.03777000	0.03614000	NM_032457	downstream	2031286	Hs.570785	PCDH7
rs10517335	4	34549602	0.0842	97.89%	0.424900	1.000000	0.03777000	0.17890000	NM_032457	downstream	3796033	Hs.570785	PCDH7
rs9294019	6	77463618	0.1724	91.58%	0.831000	0.365400	0.03777000	0.03045000	NM_000863	downstream	765023	Hs.123016	HTR1B
rs4808957	19	19428248	0.4182	96.49%	0.901300	0.287500	0.03778000	0.13790000	NM_017660	intron	0	Hs.696033	GATAD2A
rs10513965	18	65357521	0.3297	96.84%	0.277500	0.334100	0.03779000	0.12100000	NM_152721	intron	0	Hs.569915	DOK6
rs781382	14	51980688	0.1588	96.14%	0.500400	0.743300	0.03779000	0.26060000	NM_020784	intron	0	Hs.532609	TXNDC16
rs2122599	8	111137054	0.0845	97.54%	0.426000	0.407700	0.03780000	0.08332000	NM_014379	upstream	80919	Hs.13285	KCNV1
rs778432	1	56439397	0.1642	94.04%	1.000000	0.804000	0.03780000	0.06599000	NM_177414	downstream	293130	Hs.405156	PPAP2B
rs10514519	16	80532566	0.0851	96.84%	0.704200	0.223100	0.03782000	0.03421000	NM_002661	intron	0	Hs.413111	PLCG2
rs545833	1	166956564	0.3662	94.39%	0.036430	0.061740	0.03783000	0.04190000	NM_001937	intron	0	Hs.80552	DPT
rs6573512	14	62925328	0.2306	90.53%	0.158500	0.157400	0.03783000	0.07038000	NM_006246	intron	0	Hs.334868	PPP2R5E
rs967287	2	156852400	0.3333	96.84%	0.277900	0.746200	0.03783000	0.01133000	NM_173173	downstream	36794	Hs.563344	NR4A2
rs10497987	2	213555755	0.1349	97.54%	0.797500	0.701900	0.03784000	0.01578000	NM_016260	downstream	23834	Hs.604950	IKZF2
rs1400060	12	71961188	0.1576	96.84%	0.501100	0.803800	0.03784000	0.07923000	NM_013381	downstream	615500	Hs.199814	TRHDE
rs3761867	13	30613555	0.4118	95.44%	0.455600	0.441400	0.03784000	0.05253000	NM_006644	intron	0	Hs.36927	HSPH1
rs2066987	5	76892373	0.1208	94.39%	1.000000	1.000000	0.03785000	0.08920000	NM_018268	upstream	68285	Hs.482573	WDR41
rs2420178	1	68944457	0.2204	94.74%	0.160100	0.106500	0.03785000	0.04720000	NM_017779	upstream	209071	Hs.445098	DEPDC1
rs10488618	7	37979723	0.1041	94.39%	0.505700	1.000000	0.03786000	0.08216000	NM_017549	downstream	21658	Hs.563491	EPDR1
rs10494396	1	161895132	0.0749	93.68%	1.000000	1.000000	0.03786000	0.03769000	ENST00000385703	downstream	149146	---	---
rs10496819	2	139878846	0.1218	95.09%	0.777700	1.000000	0.03786000	0.04724000	ENST00000384174	upstream	106618	---	---
rs199055	6	23598096	0.0749	93.68%	0.170500	1.000000	0.03786000	0.02956000	ENST00000330282	upstream	364765	---	---
rs10504790	8	85087422	0.2565	94.39%	0.874300	1.000000	0.03787000	0.12880000	NM_173848	upstream	516690	Hs.121663	RALYL
rs644708	4	135906943	0.2190	84.91%	0.706600	1.000000	0.03788000	0.12900000	ENST00000384459	downstream	549082	---	---
rs10507672	13	61194641	0.0741	94.74%	0.166400	0.433500	0.03789000	0.03273000	ENST00000387130	upstream	64430	---	---
rs1822891	11	26982453	0.2701	91.58%	0.270900	0.297000	0.03791000	0.12560000	NM_203371	downstream	7247	Hs.705612	FIBIN
rs10484759	6	127078735	0.2177	95.09%	0.720900	0.840900	0.03792000	0.02318000	NM_032784	upstream	402707	Hs.135254	RSPO3
rs3733045	3	52618347	0.4981	92.98%	0.805900	0.758100	0.03794000	0.20290000	NM_181042	intron	0	Hs.189920	PBRM1
rs10513310	9	119644581	0.3979	82.46%	0.000216	0.000056	0.03798000	0.05102000	NM_138554	downstream	125611	Hs.174312	TLR4
rs10515255	5	96547331	0.0830	97.19%	0.103300	0.152400	0.03799000	0.03722000	NM_018343	upstream	2611	Hs.27021	RIOK2
rs9309572	2	81323050	0.2743	90.18%	0.002884	0.211800	0.03799000	0.07128000	NM_004389	downstream	594542	Hs.167368	CTNNA2
rs10512302	9	104487695	0.0591	97.89%	0.608900	0.602600	0.03800000	0.16360000	NM_001340	upstream	309735	Hs.3232	CYLC2
rs10512303	9	104487817	0.0591	97.89%	0.608900	0.602600	0.03800000	0.16360000	NM_001340	upstream	309613	Hs.3232	CYLC2
rs722183	11	7732438	0.0591	97.89%	0.608900	0.602600	0.03800000	0.10400000	ENST00000379801	downstream	7475	---	---
rs10503669	8	19891970	0.0712	96.14%	1.000000	1.000000	0.03803000	0.05370000	NM_000237	downstream	24058	Hs.180878	LPL
rs1427430	3	69838257	0.0487	97.19%	1.000000	1.000000	0.03803000	0.10400000	NM_198178	upstream	57395	Hs.166017	MITF
rs2806904	13	45383927	0.3311	80.00%	0.294900	0.475100	0.03803000	0.05319000	NM_015070	downstream	50388	Hs.136102	ZC3H13
rs10505054	8	105192600	0.1807	96.14%	0.041910	0.038480	0.03804000	0.13740000	NM_014677	intron	0	Hs.655271	RIMS2
rs33436	19	35635517	0.4377	95.79%	0.538800	0.764500	0.03807000	0.01282000	NM_014717	intron	0	Hs.378901	ZNF536
rs7305447	12	17097477	0.2491	95.09%	0.103500	0.271600	0.03807000	0.10230000	NM_001001395	upstream	445186	Hs.504908	LMO3
rs10508993	10	54712286	0.3358	96.14%	0.278900	1.000000	0.03809000	0.15010000	ENST00000387222	upstream	175668	---	---
rs1441185	1	162052120	0.4275	96.84%	1.000000	1.000000	0.03810000	0.08310000	ENST00000385701	upstream	107411	---	---
rs2378282	1	217892389	0.0573	97.89%	0.220800	0.227400	0.03810000	0.05693000	NM_138794	downstream	439559	Hs.657617	LYPLAL1
rs2378283	1	217892524	0.0573	97.89%	0.220800	0.227400	0.03810000	0.05693000	NM_138794	downstream	439694	Hs.657617	LYPLAL1
rs3802026	7	90310999	0.0573	97.89%	1.000000	0.603300	0.03810000	0.09135000	NM_012395	intron	0	Hs.430742	PFTK1
rs6483496	11	95675971	0.1813	95.79%	0.414600	0.140100	0.03813000	0.05225000	NM_024725	downstream	49618	Hs.525088	CCDC82
rs7224319	17	36410913	0.1813	95.79%	1.000000	0.380100	0.03813000	0.37220000	NM_031959	upstream	1282	Hs.307026	KRTAP3-2

rs6547232	2	79452642	0.1182	96.49%	0.778800	1.000000	0.03814000	0.16720000	NM_138937	upstream	212255	Hs.567312	REG3A
rs10491913	9	9896906	0.1531	90.53%	0.151900	0.487400	0.03816000	0.07365000	ENST00000363183	downstream	464554	---	---
rs10489579	1	183410344	0.3351	96.84%	0.223700	0.288200	0.03817000	0.02510000	NM_017673	CDS	0	Hs.134183	C1orf26
rs10498273	14	20214639	0.0964	87.37%	0.483100	0.601700	0.03817000	0.05629000	ENST00000386662	downstream	379	---	---
rs6474715	9	12471436	0.3500	87.72%	0.490300	0.857600	0.03820000	0.00334400	NM_000550	upstream	211999	Hs.270279	TYRP1
rs10493176	1	55311140	0.0618	93.68%	0.066640	0.068190	0.03821000	0.07134000	ENST00000294383	intron	0	---	---
rs7300261	12	19760199	0.1873	93.68%	0.228400	0.008859	0.03821000	0.00978200	NM_153207	downstream	195775	Hs.126497	AEBP2
rs4747989	10	12630181	0.2115	88.77%	0.088280	0.141300	0.03822000	0.03598000	NM_153498	intron	0	Hs.659517	CAMK1D
rs963165	8	135009559	0.3750	94.04%	0.897000	0.535200	0.03823000	0.02314000	NM_020863	downstream	549658	Hs.446172	ZFAT1
rs1496555	1	2224111	0.0907	67.72%	0.656600	1.000000	0.03827000	0.12840000	NM_003036	intron	0	Hs.705384	SKI
rs10485102	6	92385814	0.0419	71.23%	1.000000	1.000000	0.03828000	0.07530000	NM_145333	upstream	1032186	Hs.644143	MAP3K7
rs2895570	11	94102741	0.3285	97.19%	0.173500	0.591900	0.03828000	0.05214000	NM_130847	upstream	38444	Hs.503594	AMOTL1
rs479316	8	103015890	0.3388	96.84%	0.419600	0.292600	0.03828000	0.08320000	NM_032041	intron	0	Hs.492427	NCALD
rs4662872	2	129468207	0.1011	95.44%	1.000000	0.605300	0.03830000	0.19810000	ENST00000375987	downstream	248224	---	---
rs746365	5	2059779	0.2214	95.09%	1.000000	0.629800	0.03830000	0.13800000	NM_016358	upstream	123899	Hs.196927	IRX4
rs10516087	5	171081602	0.2781	62.46%	0.261300	0.666100	0.03831000	0.02132000	NM_003862	downstream	264835	Hs.87191	FGF18
rs10515080	5	67678969	0.0057	92.28%	1.000000	1.000000	0.03832000	0.99900000	NM_181524	downstream	45566	Hs.132225	PIK3R1
rs1146408	1	81354461	0.1354	97.19%	0.305900	0.465200	0.03832000	0.11080000	ENST00000388387	upstream	135601	---	---
rs10505953	12	24975016	0.1360	87.72%	0.001669	0.007055	0.03833000	0.29790000	NM_005504	upstream	28924	Hs.438993	BCAT1
rs10504945	8	96434663	0.0426	90.53%	0.373300	0.416600	0.03834000	0.04074000	NM_177965	upstream	84069	Hs.548157	C8orf37
rs3911569	11	94735683	0.0314	95.09%	0.228100	1.000000	0.03836000	0.09354000	NM_144665	upstream	131789	Hs.659934	SESN3
rs10506555	12	66195617	0.2613	93.33%	0.151300	0.268000	0.03837000	0.07335000	NM_006482	upstream	133404	Hs.173135	DYRK2
rs6716843	2	16301741	0.0899	93.68%	0.454300	1.000000	0.03837000	0.07333000	ENST00000388035	downstream	57696	---	---
rs10512184	9	89183421	0.0484	97.89%	0.125200	0.173500	0.03840000	0.00165900	NM_004938	upstream	118542	Hs.380277	DAPK1
rs6595716	5	125871789	0.3244	97.89%	0.132800	0.589000	0.03840000	0.02450000	NM_023927	downstream	13844	Hs.363558	GRAMD3
rs2045704	11	26375998	0.0236	96.84%	1.000000	1.000000	0.03841000	0.06811000	NM_031418	intron	0	Hs.91791	TMEM16C
rs2827016	21	22054808	0.2575	94.04%	1.000000	0.671900	0.03841000	0.09860000	ENST00000358903	downstream	116335	---	---
rs2211984	21	16923481	0.1450	91.93%	0.619200	1.000000	0.03842000	0.11820000	NM_001005734	downstream	22068	Hs.473394	C21orf34
rs10490518	2	31164857	0.3798	91.93%	0.018000	0.233400	0.03844000	0.09433000	NM_024572	intron	0	Hs.468058	GALNT14
rs10509012	10	55796840	0.1056	94.74%	0.749600	1.000000	0.03845000	0.01356000	NM_033056	intron	0	Hs.672170	PCDH15
rs10490121	2	48985285	0.0743	94.39%	0.645900	0.435500	0.03846000	0.03349000	NM_181446	downstream	57871	Hs.1428	FSHR
rs10492606	13	31281225	0.1798	88.77%	0.052220	0.072280	0.03846000	0.18050000	NM_130806	downstream	6216	Hs.680763	RXFP2
rs584197	6	124842312	0.2464	96.14%	0.514400	0.452200	0.03846000	0.00570700	NM_001040214	intron	0	Hs.656604	NKAIN2
rs10509345	10	75031309	0.0233	97.89%	1.000000	1.000000	0.03847000	0.99770000	NM_021245	downstream	30109	Hs.238756	MYOZ1
rs2462465	10	57668426	0.1350	96.14%	0.603300	1.000000	0.03847000	0.10710000	NM_001005414	downstream	118569	---	ZWINT
rs10491791	9	81286014	0.1236	95.09%	0.152000	0.692700	0.03848000	0.03404000	NM_007005	upstream	90684	Hs.444213	TLE4
rs7599374	2	167634200	0.1236	95.09%	0.267000	1.000000	0.03848000	0.06469000	ENST00000328983	intron	0	---	---
rs10491915	9	9875376	0.0394	97.89%	1.000000	1.000000	0.03850000	0.11680000	ENST00000363183	downstream	443024	---	---
rs6703370	1	192552316	0.0394	97.89%	0.349900	0.395800	0.03850000	0.09976000	ENST00000385495	downstream	171540	---	---
rs1194670	10	53818050	0.4199	89.82%	0.444600	0.520100	0.03853000	0.08539000	NM_012242	downstream	70627	Hs.40499	DKK1
rs10490937	10	42406998	0.0104	84.21%	1.000000	1.000000	0.03858000	0.25100000	NM_006955	3UTR	0	Hs.499453	ZNF33B
rs10517371	4	36008688	0.2283	92.98%	0.000190	0.056060	0.03858000	0.03906000	ENST00000357504	intron	0	---	---
rs9312226	4	169008205	0.0310	96.14%	0.225800	0.270200	0.03862000	0.11200000	NM_007193	upstream	242105	Hs.188401	ANXA10
rs10487175	7	103360382	0.0968	88.77%	0.485200	0.703000	0.03865000	0.07400000	NM_173054	intron	0	Hs.655654	RELN
rs360377	2	126836315	0.0760	92.28%	0.650300	0.643600	0.03865000	0.12230000	NM_016815	upstream	328229	Hs.59138	GYPC
rs10491094	17	10151479	0.1102	89.12%	1.000000	0.610000	0.03866000	0.00280700	NM_003802	intron	0	Hs.440895	MYH13
rs10510757	3	51385104	0.2446	96.84%	1.000000	1.000000	0.03868000	0.13260000	NM_004947	intron	0	Hs.476284	DOCK3
rs2209511	13	18600686	0.3364	96.49%	0.420700	0.859800	0.03868000	0.08776000	ENST00000343741	downstream	8873	---	---
rs4402440	13	80698201	0.4023	91.58%	1.000000	0.534000	0.03871000	0.01552000	ENST00000387380	upstream	39682	---	---
rs3011399	10	122491477	0.1642	94.04%	0.381000	0.329300	0.03872000	0.05352000	ENST00000369065	intron	0	---	---
rs10485909	7	78077684	0.0397	97.19%	0.352000	1.000000	0.03873000	0.07080000	NM_012301	intron	0	Hs.654788	MAGI2
rs354167	1	49443637	0.3357	97.19%	1.000000	1.000000	0.03874000	0.11130000	ENST00000371836	intron	0	---	---
rs10484894	6	33590032	0.0512	75.44%	0.432100	0.127700	0.03876000	0.02702000	NM_152735	downstream	56733	Hs.591805	ZBTB9
rs9290776	3	185080692	0.4683	94.04%	0.049410	0.222000	0.03876000	0.12240000	NM_001037639	intron	0	Hs.478469	PARL

rs7966096	12	13268178	0.2153	96.14%	0.859700	1.000000	0.03878000	0.02606000	NM_001423	downstream	7207	Hs.705477	EMP1
rs10513597	3	164180162	0.0717	97.89%	0.376600	0.367000	0.03879000	0.00850000	ENST00000365009	downstream	490525	---	---
rs915877	21	44327272	0.0717	97.89%	1.000000	1.000000	0.03879000	0.01672000	NM_003274	CDS	0	Hs.126221	TMEM1
rs10505965	12	25614865	0.0532	92.28%	1.000000	1.000000	0.03880000	0.02818000	NM_152590	upstream	17420	Hs.44647	IFLTD1
rs2287686	5	115859229	0.2293	93.33%	1.000000	1.000000	0.03880000	0.01965000	NM_020796	intron	0	Hs.156967	SEMA6A
rs7770496	6	156756476	0.3067	94.39%	0.886000	1.000000	0.03880000	0.00891500	ENST00000385709	downstream	14829	---	---
rs224940	5	81752818	0.4485	95.44%	0.461900	0.361900	0.03881000	0.03953000	NM_001039779	downstream	34727	---	FLJ41309
rs1021261	11	24698333	0.3699	94.39%	0.600800	0.876300	0.03882000	0.15080000	NM_001009909	intron	0	Hs.144138	LUZP2
rs10518529	4	129279663	0.2410	97.54%	0.745000	0.704100	0.03882000	0.26830000	NM_032239	intron	0	Hs.657067	LARP2
rs7698500	4	73279543	0.2106	89.12%	0.184300	0.439800	0.03882000	0.08904000	NM_053036	downstream	46901	Hs.99231	NPFFR2
rs10506985	12	89236173	0.2076	97.19%	0.143900	0.400300	0.03883000	0.06326000	ENST00000388111	downstream	591243	---	---
rs678038	9	22697594	0.2428	96.84%	0.412000	1.000000	0.03885000	0.10560000	NM_022160	downstream	255122	Hs.371976	DMRTA1
rs6724282	2	24847176	0.0520	94.39%	0.523300	0.204200	0.03886000	0.08853000	NM_147233	downstream	101	Hs.699183	NCOA1
rs3213732	2	102364711	0.4291	96.49%	0.084340	0.129000	0.03888000	0.31340000	NM_003855	intron	0	Hs.469521	IL18R1
rs590599	6	153161845	0.1382	96.49%	0.798700	0.260800	0.03888000	0.01668000	NM_194435	downstream	39252	Hs.53973	VIP
rs10505613	8	134855058	0.1464	92.28%	0.043940	0.073670	0.03889000	0.07002000	NM_020863	downstream	704159	Hs.446172	ZFAT1
rs10514753	3	111616274	0.0399	96.84%	0.353100	1.000000	0.03889000	0.22240000	ENST00000388702	downstream	137134	---	---
rs2173482	1	211733512	0.4083	97.54%	0.457200	1.000000	0.03889000	0.02842000	ENST00000331583	upstream	64470	---	---
rs10489332	1	173217691	0.0699	97.89%	0.034130	0.028870	0.03890000	0.27660000	ENST00000347255	intron	0	---	---
rs3758697	11	100215104	0.0730	96.14%	0.642600	0.636000	0.03891000	0.02301000	ENST00000363177	upstream	467	---	---
rs4570109	8	82168194	0.4104	97.89%	0.710500	0.634400	0.03891000	0.01689000	NM_018440	intron	0	Hs.266175	PAG1
rs3772812	3	125966523	0.2778	97.89%	0.025940	0.013960	0.03892000	0.02731000	NM_002213	intron	0	Hs.536663	ITGB5
rs10516467	4	101820125	0.3370	96.84%	0.345700	0.201400	0.03893000	0.07668000	NM_016242	upstream	161923	Hs.152913	EMCN
rs1360841	6	79939428	0.0625	92.63%	1.000000	1.000000	0.03893000	0.01924000	NM_138730	downstream	28257	Hs.77558	HMGN3
rs3902839	1	216355561	0.3041	85.96%	0.000001	0.000002	0.03893000	0.15640000	NM_138796	downstream	243900	Hs.171130	SPATA17
rs2199219	4	62866363	0.1523	89.82%	0.053510	0.420800	0.03896000	0.05632000	ENST00000273844	upstream	212001	---	---
rs4695256	4	47309657	0.3309	97.54%	1.000000	0.331400	0.03896000	0.03220000	NM_006587	intron	0	Hs.518618	CORIN
rs7328333	13	80236724	0.1810	97.89%	0.423000	0.499700	0.03897000	0.17850000	ENST00000365528	upstream	395054	---	---
rs2204134	7	54586758	0.2850	75.09%	0.000009	0.000096	0.03898000	0.13290000	NM_182546	3UTR	0	Hs.335933	VSTM2A
rs7649500	3	146739486	0.1679	94.04%	0.380200	0.535700	0.03898000	0.08856000	ENST00000356397	upstream	525562	---	---
rs2291386	6	159104495	0.0315	94.74%	0.228800	1.000000	0.03899000	0.04231000	NM_001009991	CDS	0	Hs.436977	SYTL3
rs710518	3	190242432	0.0315	94.74%	1.000000	1.000000	0.03899000	0.07391000	NM_005578	downstream	167471	Hs.444362	LPP
rs6814821	4	170394672	0.0813	88.42%	0.214900	0.017540	0.03900000	0.05408000	NM_020870	intron	0	Hs.301804	SH3RF1
rs1955557	14	39820852	0.2097	97.89%	0.006579	0.060560	0.03902000	0.17260000	NM_203301	upstream	849481	Hs.324342	FBXO33
rs7592580	2	27974694	0.3327	97.54%	0.787000	0.859100	0.03905000	0.05798000	NM_199192	intron	0	Hs.258314	BRE
rs2120524	6	81802454	0.4346	91.23%	0.376600	0.874500	0.03908000	0.05008000	NM_000056	downstream	689764	Hs.654441	BCKDHB
rs382379	4	2776707	0.4234	96.14%	1.000000	0.878700	0.03908000	0.08792000	ENST00000356331	intron	0	---	---
rs1430235	2	221931811	0.3078	94.04%	0.251800	0.257400	0.03909000	0.04760000	NM_004438	downstream	59182	Hs.371218	EPHA4
rs191692	2	60315082	0.3189	92.98%	0.671700	0.712700	0.03910000	0.00657700	ENST00000363937	upstream	150162	---	---
rs3924594	12	73969063	0.3189	92.98%	0.322300	0.460900	0.03910000	0.02638000	NM_032606	intron	0	Hs.407154	CAPS2
rs10493096	1	42097709	0.2241	94.74%	0.726200	0.112800	0.03911000	0.07192000	NM_024503	intron	0	Hs.591503	HIVEP3
rs6728771	2	201700652	0.2281	92.28%	0.603300	0.544900	0.03911000	0.05833000	NM_003879	intron	0	Hs.390736	CFLAR
rs3731348	7	92091169	0.0412	93.68%	1.000000	1.000000	0.03912000	0.10230000	NM_001259	intron	0	Hs.119882	CDK6
rs952793	20	6918831	0.1178	96.84%	0.778000	0.662700	0.03912000	0.00825700	NM_001200	downstream	209904	Hs.73853	BMP2
rs1413213	1	240872523	0.4137	97.54%	0.712100	0.880100	0.03913000	0.01591000	NM_152666	upstream	117902	Hs.672452	PLD5
rs7797684	7	3940681	0.0056	94.74%	1.000000	1.000000	0.03914000	0.99900000	NM_152744	intron	0	Hs.655699	SDK1
rs10500108	7	124779737	0.1926	85.61%	0.060800	0.165400	0.03915000	0.15010000	NM_001042594	upstream	422627	Hs.31968	POT1
rs1433114	19	34206067	0.1606	96.14%	1.000000	1.000000	0.03915000	0.27370000	NM_006003	downstream	183940	Hs.170107	UQCRFS1
rs7148692	14	39577732	0.0644	92.63%	0.609800	0.605500	0.03915000	0.02522000	NM_203301	upstream	606361	Hs.324342	FBXO33
rs1007212	15	97101441	0.4023	93.33%	0.371800	0.199700	0.03916000	0.02396000	NM_000875	intron	0	Hs.698330	IGF1R
rs9298952	9	3358781	0.0637	93.68%	0.609600	0.605300	0.03916000	0.04859000	NM_134428	intron	0	Hs.136829	RFX3
rs576266	11	115993343	0.1372	97.19%	0.442100	0.260000	0.03918000	0.03754000	NM_032725	downstream	130753	Hs.437341	BUD13
rs10501480	11	80209157	0.2611	94.74%	0.114000	0.202100	0.03919000	0.10010000	ENST00000363276	downstream	292399	---	---
rs762147	21	38160480	0.1966	93.68%	0.559900	0.519700	0.03919000	0.05944000	NM_002240	upstream	136918	Hs.658533	KCNJ6

rs4698818	4	111390394	0.4221	96.84%	0.266300	0.293200	0.03920000	0.03438000	NM_024090	upstream	51174	Hs.412939	ELOVL6
rs10515324	5	101649839	0.1439	95.09%	0.805900	1.000000	0.03921000	0.02282000	NM_180991	intron	0	Hs.127648	SLCO4C1
rs10506524	12	64070379	0.0517	95.09%	1.000000	1.000000	0.03924000	0.05682000	NM_001031679	intron	0	Hs.339024	MSRB3
rs10510528	3	22455267	0.0059	89.47%	0.005894	1.000000	0.03926000	0.99870000	ENST00000255320	intron	0	---	---
rs10499349	7	9212088	0.4536	83.16%	0.793200	0.750900	0.03927000	0.05875000	NM_152745	downstream	454164	Hs.487564	NXPH1
rs10514542	16	81115185	0.1594	96.84%	0.653100	0.616900	0.03928000	0.01651000	NM_001257	upstream	334275	Hs.654386	CDH13
rs2389016	1	80571917	0.2251	95.09%	1.000000	0.429500	0.03929000	0.08207000	ENST00000388387	upstream	918145	---	---
rs6748166	2	170498527	0.4087	92.28%	0.445000	1.000000	0.03930000	0.11590000	NM_172070	intron	0	---	UBR3
rs9305856	21	18529414	0.0904	95.09%	0.250800	0.233500	0.03931000	0.01967000	NM_024944	upstream	9607	Hs.283725	CHODL
rs10507470	13	37828100	0.0246	92.63%	1.000000	1.000000	0.03932000	0.09120000	NM_016617	intron	0	Hs.643655	UFM1
rs1882027	1	30885819	0.1279	91.93%	0.158500	0.094170	0.03932000	0.12040000	NM_002379	downstream	72764	Hs.150366	MATN1
rs204434	22	33124787	0.4309	96.49%	0.221000	0.115500	0.03932000	0.02630000	NM_133642	upstream	478461	Hs.474667	LARGE
rs10504736	8	82269581	0.2259	94.74%	0.035930	0.342200	0.03933000	0.05586000	NM_018440	upstream	82723	Hs.266175	PAG1
rs605781	21	43558435	0.1193	85.26%	0.010380	0.004398	0.03934000	0.06765000	NM_000394	downstream	92453	Hs.184085	CRYAA
rs7231714	18	59725580	0.3839	93.68%	0.897100	0.506500	0.03934000	0.10090000	NM_002575	downstream	3480	Hs.594481	SERPINB2
rs10493133	1	48813681	0.1349	97.54%	0.797500	1.000000	0.03935000	0.04119000	ENST00000371839	intron	0	---	---
rs9315797	13	40453352	0.1349	97.54%	0.065520	0.047700	0.03935000	0.04754000	NM_172373	intron	0	Hs.135646	ELF1
rs10493895	1	98043814	0.1588	97.19%	0.073330	0.132800	0.03937000	0.03654000	NM_000110	intron	0	Hs.335034	DPYD
rs10495723	2	21910766	0.3722	94.74%	0.241700	0.061120	0.03937000	0.01624000	ENST00000383859	upstream	88604	---	---
rs6857229	4	76710592	0.1588	97.19%	0.367700	0.615700	0.03937000	0.04338000	NM_178497	downstream	487	Hs.24510	C4orf26
rs10484177	14	43966684	0.4505	95.79%	0.393000	0.088070	0.03938000	0.10830000	NM_032135	downstream	76779	Hs.307086	FSCB
rs10501935	11	99317183	0.1437	94.04%	0.004330	0.018000	0.03938000	0.01454000	NM_175566	intron	0	Hs.656783	CNTN5
rs605954	11	74258861	0.1992	93.33%	0.338800	0.426000	0.03938000	0.00403900	ENST00000321448	intron	0	---	---
rs10488398	7	135214873	0.0410	94.04%	0.361800	0.401300	0.03939000	0.04120000	NM_145808	downstream	47178	Hs.654894	MTPN
rs10518349	1	71024526	0.0907	94.74%	0.466700	1.000000	0.03939000	0.05245000	NM_198719	downstream	184526	Hs.445000	PTGER3
rs10508433	10	11909527	0.1203	83.16%	0.544800	0.740700	0.03940000	0.02831000	NM_153256	intron	0	Hs.435775	C10orf47
rs10515938	2	171098475	0.1828	97.89%	0.426900	1.000000	0.03942000	0.02181000	NM_138995	intron	0	Hs.671900	MYO3B
rs941725	14	102181576	0.0911	94.39%	0.143300	1.000000	0.03943000	0.08293000	NM_015156	intron	0	Hs.510521	RCOR1
rs902523	5	77251993	0.1612	95.79%	0.500500	0.803000	0.03944000	0.00445600	NM_003664	downstream	80112	Hs.532091	AP3B1
rs1983085	3	15504547	0.3116	94.04%	0.776600	0.867800	0.03945000	0.27530000	NM_005677	intron	0	Hs.146735	COLQ
rs4263839	9	116606261	0.2264	92.98%	0.077410	0.231100	0.03945000	0.13970000	NM_005118	intron	0	Hs.241382	TNFSF15
rs10508263	10	3775605	0.0616	94.04%	0.258200	0.265000	0.03946000	0.15590000	NM_001300	downstream	32583	Hs.4055	KLF6
rs1951537	14	28630572	0.1019	94.74%	0.745400	0.720700	0.03946000	0.08638000	ENST00000386746	upstream	109205	---	---
rs9304469	18	20423422	0.0659	90.53%	0.084600	0.086370	0.03946000	0.01905000	NM_021624	downstream	111855	Hs.287388	HRH4
rs10505062	8	105663612	0.0627	95.09%	0.075020	0.077070	0.03947000	0.04005000	NM_013437	intron	0	Hs.654804	LRP12
rs3917289	2	102148343	0.0639	93.33%	0.285400	0.295800	0.03949000	0.02259000	NM_000877	intron	0	Hs.701982	IL1R1
rs1501845	5	61012367	0.1250	94.04%	0.778600	0.222100	0.03950000	0.01820000	NM_173667	upstream	6099	Hs.683866	FLJ37543
rs4980171	10	124733190	0.4209	97.54%	0.109300	0.343000	0.03951000	0.02839000	NM_153336	intron	0	Hs.281004	PSTK
rs342239	7	106124138	0.3704	94.74%	0.009284	0.240200	0.03952000	0.12160000	NM_002649	upstream	168839	Hs.32942	PIK3CG
rs756452	5	85703889	0.2130	97.19%	0.719900	1.000000	0.03952000	0.01111000	NM_001867	upstream	245588	Hs.430075	COX7C
rs10483708	14	58478842	0.2274	93.33%	0.079540	0.339000	0.03953000	0.01330000	NM_014992	upstream	246310	Hs.654934	DAAM1
rs10489580	1	183408955	0.3412	97.19%	0.109800	0.218400	0.03953000	0.01758000	NM_017673	intron	0	Hs.134183	C1orf26
rs735390	12	87899538	0.0637	93.68%	0.609600	1.000000	0.03953000	0.01769000	ENST00000388095	downstream	114892	---	---
rs9302327	15	27944249	0.2659	93.68%	1.000000	0.855500	0.03953000	0.01140000	NM_003257	upstream	42239	Hs.510833	TJP1
rs28373914	13	32099966	0.3412	96.14%	0.139300	0.052330	0.03954000	0.06027000	NM_015032	intron	0	Hs.699308	PDS5B
rs726863	1	150666985	0.3394	97.19%	0.284100	0.078160	0.03955000	0.12100000	NM_016190	upstream	13622	Hs.242057	CRNN
rs10491874	9	1251602	0.0056	94.39%	1.000000	1.000000	0.03957000	0.99860000	NM_181872	downstream	204050	Hs.59506	DMRT2
rs2120021	15	96719695	0.2464	96.84%	1.000000	0.661600	0.03957000	0.00798900	NM_182562	downstream	80688	Hs.668070	FLJ39743
rs6869856	5	166017651	0.3302	75.44%	0.090510	0.279400	0.03958000	0.08707000	ENST00000384798	downstream	205147	---	---
rs1880014	8	123672654	0.4366	96.84%	0.327200	0.232300	0.03959000	0.02528000	NM_005328	upstream	949843	Hs.571528	HAS2
rs1918715	8	117404580	0.0305	97.89%	1.000000	1.000000	0.03961000	0.07206000	NM_003756	downstream	321673	Hs.492599	EIF3H
rs212390	6	159409555	0.1293	90.88%	0.401900	1.000000	0.03962000	0.07527000	NM_138810	upstream	23383	Hs.529984	TAGAP
rs4891538	18	70088232	0.1731	91.23%	0.029180	0.010680	0.03962000	0.10840000	NM_001914	intron	0	Hs.465413	CYB5A
rs10504128	8	52911112	0.2678	93.68%	0.029040	0.069080	0.03963000	0.00580300	ENST00000360540	intron	0	---	---

rs953528	17	23092917	0.4487	82.11%	0.003808	0.002045	0.03963000	0.21230000	NM_153292	downstream	15005	---	NOS2A
rs17060071	13	38976326	0.0316	94.39%	0.022520	0.058970	0.03964000	0.01018000	NM_005780	intron	0	Hs.507798	LHFP
rs2450287	8	50992349	0.0874	94.39%	0.704100	0.549600	0.03965000	0.06270000	NM_018967	upstream	476977	Hs.584914	SNTG1
rs528633	6	117755033	0.1096	91.23%	1.000000	1.000000	0.03966000	0.17110000	NM_002944	intron	0	Hs.1041	ROS1
rs6487673	12	28329674	0.0409	94.39%	0.360700	0.399500	0.03970000	0.02378000	NM_018318	intron	0	Hs.653125	CCDC91
rs7336772	13	101917288	0.3792	94.39%	0.028170	0.404500	0.03970000	0.04111000	NM_175929	upstream	65132	Hs.696392	FGF14
rs485865	7	71371987	0.1116	88.07%	0.099010	0.342400	0.03972000	0.07108000	NM_001017440	intron	0	Hs.592750	CALN1
rs597741	18	53355474	0.3796	67.02%	0.218400	0.098560	0.03974000	0.02393000	NM_000140	downstream	11061	Hs.465221	FECH
rs10515496	5	137072425	0.1155	88.07%	1.000000	0.741400	0.03975000	0.02163000	NM_017415	intron	0	Hs.655084	KLHL3
rs1125930	13	60210088	0.0962	91.23%	1.000000	1.000000	0.03977000	0.10800000	ENST00000384127	upstream	51607	---	---
rs10512781	5	41270400	0.3369	97.89%	0.690000	0.336800	0.03978000	0.08609000	NM_000065	intron	0	Hs.481992	C6
rs1073277	4	36680445	0.1222	93.33%	0.777400	0.534300	0.03978000	0.03163000	ENST00000389006	upstream	354001	---	---
rs2061459	4	66751596	0.4601	96.84%	0.545500	0.879600	0.03978000	0.03276000	ENST00000339867	downstream	108759	---	---
rs10493731	1	83043983	0.1444	94.74%	0.806500	0.724800	0.03979000	0.03159000	NM_012302	downstream	813288	Hs.24212	LPHN2
rs3736583	10	124732782	0.4211	97.89%	0.111300	0.347200	0.03981000	0.02830000	NM_153336	CDS	0	Hs.281004	PSTK
rs4270120	15	43723526	0.4211	97.89%	0.066080	0.059870	0.03981000	0.07333000	NM_021199	intron	0	Hs.511251	SQRDL
rs679783	1	49322083	0.3345	97.54%	0.892800	0.723900	0.03982000	0.11220000	ENST00000371836	intron	0	---	---
rs693014	1	65525828	0.0771	65.96%	0.295600	1.000000	0.03982000	0.29190000	NM_014787	intron	0	Hs.647643	DNAJC6
rs1999334	21	45494885	0.3191	90.18%	0.153800	0.056990	0.03983000	0.03010000	ENST00000364254	downstream	6152	---	---
rs2402298	7	117521094	0.0489	96.84%	0.487400	1.000000	0.03983000	0.19870000	NM_016200	upstream	90361	Hs.657737	LSM8
rs2133516	4	30822103	0.0996	84.56%	1.000000	1.000000	0.03984000	0.03885000	NM_032457	downstream	68534	Hs.570785	PCDH7
rs2827114	21	22205437	0.2100	70.18%	0.000204	0.000645	0.03984000	0.07616000	ENST00000333482	upstream	198221	---	---
rs10515507	5	138694271	0.2832	97.89%	1.000000	0.728100	0.03986000	0.11460000	NM_018834	downstream	244	Hs.268939	MATR3
rs726206	12	16541935	0.4247	97.89%	0.270300	0.654000	0.03986000	0.12310000	NM_001001395	downstream	50639	Hs.504908	LMO3
rs10513200	3	144611488	0.2196	95.09%	0.594600	1.000000	0.03987000	0.20850000	NM_173653	intron	0	Hs.302257	SLC9A9
rs666214	2	45741435	0.1558	91.23%	0.015310	0.187200	0.03989000	0.04854000	NM_005400	intron	0	Hs.580351	PRKCE
rs10487416	7	119528279	0.0179	97.89%	1.000000	1.000000	0.03991000	0.18940000	ENST00000350695	upstream	94888	---	---
rs825064	12	100541701	0.0714	85.96%	0.110700	0.361800	0.03991000	0.09460000	NM_206821	intron	0	Hs.654589	MYBPC1
rs985788	2	167205127	0.0179	97.89%	1.000000	1.000000	0.03991000	0.08815000	NM_002976	upstream	153400	Hs.695947	SCN7A
rs10484629	6	133159563	0.0733	81.40%	1.000000	1.000000	0.03992000	0.05036000	NM_052831	intron	0	Hs.347144	C6orf192
rs1356300	4	153604919	0.3895	93.68%	0.248400	0.410400	0.03992000	0.00853300	NM_033632	intron	0	Hs.696040	FBXW7
rs1543673	9	28417231	0.4618	96.49%	0.808200	0.654600	0.03992000	0.01730000	NM_152570	intron	0	Hs.699432	LINGO2
rs10513328	9	120113841	0.4075	92.98%	1.000000	0.646000	0.03994000	0.08117000	NM_014618	downstream	854888	Hs.532316	DBC1
rs1732474	12	126013813	0.0193	90.88%	0.084700	1.000000	0.03996000	0.07089000	ENST00000364482	upstream	49896	---	---
rs4128982	8	131966264	0.2035	90.53%	0.568800	0.382000	0.03997000	0.01052000	NM_001115	intron	0	Hs.591859	ADCY8
rs10520462	4	182262097	0.1468	94.39%	0.811700	1.000000	0.03998000	0.02878000	ENST00000378957	upstream	418710	---	---
rs10491652	9	5099531	0.0055	95.09%	1.000000	1.000000	0.03999000	0.99870000	NM_004972	intron	0	Hs.656213	JAK2
rs10500155	7	145063916	0.0782	91.93%	1.000000	1.000000	0.03999000	0.02162000	ENST00000385572	upstream	261438	---	---
rs10501112	11	29165022	0.0055	95.09%	1.000000	1.000000	0.03999000	0.99880000	NM_152636	downstream	853503	Hs.243326	METT5D1
rs10502102	11	107935884	0.0055	95.09%	1.000000	1.000000	0.03999000	0.99880000	NM_015065	intron	0	Hs.269591	EXPH5
rs10504320	8	62090667	0.0055	95.09%	1.000000	1.000000	0.03999000	0.99880000	NM_017780	downstream	148650	Hs.20395	CHD7
rs10506370	12	57217919	0.0055	95.09%	1.000000	1.000000	0.03999000	0.99880000	NM_153377	downstream	334286	Hs.253736	LRIG3
rs10506774	12	77107229	0.0055	95.09%	1.000000	1.000000	0.03999000	0.99870000	NM_014903	intron	0	Hs.655301	NAV3
rs10508510	10	16422484	0.0055	95.09%	1.000000	1.000000	0.03999000	0.99880000	ENST00000386700	downstream	58176	---	---
rs10514239	5	81714745	0.0055	95.09%	1.000000	1.000000	0.03999000	0.99870000	NM_001039779	5UTR	0	---	FLJ41309
rs10517726	4	161971354	0.0055	95.09%	1.000000	1.000000	0.03999000	0.99900000	NM_020116	downstream	553147	Hs.591707	FSTL5
rs1372717	2	106162372	0.0055	95.09%	1.000000	1.000000	0.03999000	0.99860000	NM_025076	intron	0	Hs.469561	UXS1
rs1550600	4	78545318	0.0055	95.09%	1.000000	1.000000	0.03999000	0.99880000	NM_006419	upstream	200680	Hs.100431	CXCL13
rs1837872	4	77429961	0.0055	95.09%	1.000000	1.000000	0.03999000	0.99880000	NM_003943	upstream	16242	Hs.109590	STBD1
rs3797364	5	118877864	0.0055	95.09%	1.000000	1.000000	0.03999000	0.99880000	NM_000414	intron	0	Hs.406861	HSD17B4
rs6029893	20	40018906	0.3460	96.84%	1.000000	0.861600	0.03999000	0.06153000	NM_007050	downstream	115900	Hs.526879	PTPRT
rs10497111	2	153147664	0.1269	92.63%	0.047500	0.092520	0.04000000	0.05029000	NM_052905	intron	0	Hs.654630	FMNL2
rs10520091	15	35805776	0.0078	67.37%	0.007833	1.000000	0.04001000	0.99880000	NM_152453	upstream	208973	Hs.179646	TMCO5
rs1553622	1	207530483	0.2267	86.67%	0.370400	0.410200	0.04003000	0.04252000	NM_025179	upstream	1046195	Hs.497626	PLXNA2

rs3884586	1	115938348	0.0634	94.04%	0.609500	0.604700	0.04003000	0.02320000	NM_002506	upstream	255968	Hs.2561	NGFB
rs1112687	1	49841291	0.3381	97.54%	0.788600	0.725800	0.04004000	0.14580000	ENST00000371836	intron	0	---	---
rs2375675	1	65325781	0.1004	97.89%	0.172000	0.143000	0.04005000	0.09395000	ENST00000362265	upstream	28998	---	---
rs10236906	7	18706195	0.0942	91.23%	0.142600	0.603100	0.04007000	0.07958000	NM_178425	intron	0	Hs.196054	HDAC9
rs1417315	13	58408332	0.3256	75.44%	0.043450	0.145800	0.04007000	0.03515000	ENST00000365514	upstream	408446	---	---
rs1446499	2	47000878	0.1191	97.19%	0.392900	0.675000	0.04007000	0.09179000	NM_139279	upstream	4425	Hs.293689	MCFD2
rs8177130	7	142269523	0.0306	97.54%	1.000000	1.000000	0.04007000	0.08412000	NM_004445	upstream	1106	Hs.380089	EPHB6
rs10509707	10	98039194	0.1380	97.89%	0.622500	1.000000	0.04008000	0.17880000	NM_001017520	upstream	14881	Hs.534206	DNTT
rs10233491	7	25433046	0.2317	90.88%	0.000744	0.002228	0.04011000	0.22900000	ENST00000365363	downstream	162021	---	---
rs10235122	7	21192425	0.0260	87.72%	0.147900	0.189300	0.04011000	0.05549000	ENST00000387535	downstream	29256	---	---
rs2008612	8	101236871	0.4363	90.88%	0.801100	0.635800	0.04011000	0.09934000	NM_005034	downstream	1465	Hs.351475	POLR2K
rs4889002	16	78219413	0.4176	97.89%	0.139100	0.879900	0.04011000	0.05233000	NM_001031804	upstream	27301	Hs.134859	MAF
rs10493728	1	82967419	0.1588	97.19%	0.652400	0.530600	0.04013000	0.08941000	NM_012302	downstream	736724	Hs.24212	LPHN2
rs1055252	13	25516922	0.2330	92.63%	0.604800	0.357400	0.04013000	0.18660000	NM_001007538	3UTR	0	Hs.433791	TMEM46
rs10485929	7	78524904	0.2115	97.89%	0.590500	0.803600	0.04014000	0.05738000	NM_012301	intron	0	Hs.654788	MAGI2
rs10484527	6	166964675	0.0059	89.82%	1.000000	1.000000	0.04016000	0.99870000	ENST00000366866	intron	0	---	---
rs539937	4	16801724	0.4881	88.42%	0.131900	0.058630	0.04019000	0.09904000	NM_001290	upstream	292299	Hs.23748	LDB2
rs10504840	8	89496115	0.1929	93.68%	0.049690	0.114700	0.04023000	0.07808000	NM_003821	upstream	1343058	Hs.103755	RIPK2
rs1943714	11	84236319	0.3202	93.68%	0.888300	1.000000	0.04023000	0.08517000	ENST00000376104	intron	0	---	---
rs1392326	3	169050871	0.3387	97.89%	0.062120	0.079220	0.04024000	0.02750000	NM_005025	downstream	24829	Hs.478153	SERPINI1
rs2615295	15	42199890	0.1011	97.19%	0.748200	1.000000	0.04024000	0.06276000	NM_032892	intron	0	Hs.578544	FRMD5
rs2078426	15	52740396	0.4863	89.47%	0.380300	0.353700	0.04025000	0.05688000	NM_016304	downstream	520417	Hs.274772	C15orf15
rs1831298	1	90272407	0.2657	95.09%	0.640000	0.718800	0.04026000	0.02577000	NM_181781	downstream	5725	---	ZNF326
rs1978797	16	61814767	0.0308	96.84%	1.000000	1.000000	0.04029000	0.08435000	NM_001797	downstream	1723419	Hs.116471	CDH11
rs794129	6	164626098	0.0441	91.58%	0.397500	0.441400	0.04029000	0.03894000	NM_144980	downstream	987058	Hs.144734	C6orf118
rs9638846	7	27720217	0.3829	94.39%	0.439000	0.757400	0.04031000	0.03386000	NM_006024	upstream	26116	Hs.34576	TAX1BP1
rs10517426	4	58541512	0.0686	89.47%	0.100700	0.341300	0.04033000	0.03015000	ENST00000387903	upstream	488216	---	---
rs1985065	4	165421869	0.3866	94.39%	0.520100	0.537900	0.04033000	0.00806900	NM_012403	upstream	83556	Hs.661161	ANP32C
rs10504873	8	90138115	0.0630	94.74%	1.000000	0.303000	0.04034000	0.17470000	NM_003821	upstream	701058	Hs.103755	RIPK2
rs10510108	10	124086150	0.0756	95.09%	0.378900	0.368600	0.04034000	0.09543000	NM_144587	CDS	0	Hs.422466	BTBD16
rs10514974	5	63879671	0.3302	91.93%	0.676700	0.854800	0.04034000	0.03578000	NM_001029875	intron	0	Hs.657133	RGS7BP
rs6967565	7	8096253	0.3868	92.98%	0.604200	0.248900	0.04037000	0.01814000	NM_138426	downstream	2760	Hs.131673	GLCCI1
rs728539	7	30614576	0.1400	96.49%	0.448800	0.259900	0.04037000	0.24530000	NM_002047	intron	0	Hs.404321	GARS
rs2184719	10	56193061	0.1022	97.89%	1.000000	0.732700	0.04038000	0.01780000	NM_033056	intron	0	Hs.672170	PCDH15
rs251506	5	52549638	0.0958	91.58%	0.270400	1.000000	0.04039000	0.02596000	NM_004531	upstream	108302	Hs.163645	MOCS2
rs10505180	8	113392265	0.1458	95.09%	0.014320	0.013240	0.04040000	0.13200000	NM_052900	intron	0	Hs.91381	CSMD3
rs1436302	2	125890401	0.2933	89.12%	0.069010	0.070800	0.04040000	0.00095670	NM_016815	upstream	1274143	Hs.59138	GYPC
rs7146962	14	53215067	0.3190	77.54%	0.440500	0.856000	0.04041000	0.01664000	NM_130851	downstream	271140	Hs.68879	BMP4
rs349235	4	60516353	0.4317	97.54%	0.272300	0.349900	0.04042000	0.03864000	ENST00000364905	downstream	133873	---	---
rs10486577	7	28100688	0.0056	94.74%	1.000000	1.000000	0.04043000	0.99880000	NM_175061	intron	0	Hs.368944	JAZF1
rs10496331	2	98708387	0.0056	94.74%	0.005566	1.000000	0.04043000	0.99850000	NM_012214	intron	0	Hs.177576	MGAT4A
rs10501107	11	28934521	0.0056	94.74%	1.000000	1.000000	0.04043000	0.99880000	NM_152636	downstream	623002	Hs.243326	METT5D1
rs10504907	8	92502885	0.0056	94.74%	1.000000	1.000000	0.04043000	0.99880000	NM_134266	downstream	26763	Hs.354013	SLC26A7
rs10515483	5	136168921	0.0056	94.74%	1.000000	1.000000	0.04043000	0.99870000	NM_020389	upstream	447947	Hs.591263	TRPC7
rs2410039	8	13111212	0.0056	94.74%	1.000000	1.000000	0.04043000	0.99880000	NM_182643	intron	0	Hs.134296	DLC1
rs352035	4	75005492	0.0056	94.74%	1.000000	1.000000	0.04043000	0.99880000	NM_002619	downstream	60168	Hs.81564	PF4
rs811888	3	119475535	0.0056	94.74%	1.000000	1.000000	0.04043000	0.99870000	NM_001015887	downstream	628156	Hs.112873	IGSF11
rs2134095	1	163644176	0.3509	96.49%	1.000000	0.387300	0.04044000	0.20530000	NM_006917	CDS	0	Hs.26550	RXRG
rs9285134	13	38982118	0.2140	97.54%	1.000000	0.415200	0.04045000	0.01360000	NM_005780	intron	0	Hs.507798	LHFP
rs9316657	13	53230159	0.3910	93.33%	0.796700	0.250400	0.04045000	0.05320000	ENST00000387037	downstream	499651	---	---
rs1008427	3	171733956	0.0056	93.68%	1.000000	1.000000	0.04047000	0.99860000	NM_020949	intron	0	Hs.674783	SLC7A14
rs2054069	8	126708685	0.0056	93.68%	1.000000	1.000000	0.04047000	0.99880000	NM_025195	downstream	188861	Hs.444947	TRIB1
rs1400061	12	71960825	0.1607	97.19%	0.501800	0.803600	0.04048000	0.08137000	NM_013381	downstream	615137	Hs.199814	TRHDE
rs1731545	12	66295979	0.4355	97.89%	0.903200	0.437100	0.04050000	0.27550000	NM_006482	upstream	33042	Hs.173135	DYRK2

rs10515291	5	99258961	0.0057	92.63%	1.000000	1.000000	0.04051000	0.99860000	ENST00000362508	upstream	126446	---	---
rs1473141	10	120133148	0.2797	82.81%	0.146700	0.134300	0.04051000	0.00341900	NM_022063	upstream	41326	Hs.372309	C10orf84
rs9311887	3	64329981	0.1090	93.33%	1.000000	1.000000	0.04051000	0.16690000	NM_198859	upstream	143810	Hs.699317	PRICKLE2
rs10489715	1	184039665	0.0243	94.04%	1.000000	1.000000	0.04052000	0.10350000	NM_031935	intron	0	Hs.58877	HMCN1
rs1407179	6	117831192	0.4727	96.49%	0.184200	0.651900	0.04052000	0.03479000	NM_002944	intron	0	Hs.1041	ROS1
rs3126042	6	155017159	0.1704	94.74%	1.000000	1.000000	0.04052000	0.04730000	NM_014892	upstream	78992	Hs.591329	RBM16
rs7831445	8	15625490	0.0243	94.04%	1.000000	1.000000	0.04052000	0.18950000	NM_178234	intron	0	Hs.591845	TUSC3
rs10510164	3	315536	0.1870	89.12%	0.006091	0.000690	0.04053000	0.28680000	NM_006614	intron	0	Hs.148909	CHL1
rs2302373	10	115333277	0.2455	97.89%	0.034780	0.058770	0.04053000	0.03312000	NM_004132	intron	0	Hs.422542	HABP2
rs10513381	3	152119387	0.0365	81.75%	0.029590	0.040140	0.04056000	0.09950000	NM_052995	downstream	7253	Hs.380222	CLRN1
rs1692523	18	24499034	0.4564	96.49%	0.113900	0.282800	0.04056000	0.01971000	NM_001792	upstream	487845	Hs.464829	CDH2
rs4762169	12	95958668	0.4760	87.72%	0.075400	0.107400	0.04056000	0.06295000	ENST00000365498	upstream	61130	---	---
rs1407467	9	7958906	0.4065	91.93%	0.522000	1.000000	0.04058000	0.38690000	NM_033428	upstream	168839	Hs.7517	C9orf123
rs1066067	12	76443469	0.3671	88.42%	0.222200	0.119400	0.04060000	0.02210000	ENST00000378652	upstream	250225	---	---
rs1276672	5	157980115	0.0925	92.98%	0.709100	1.000000	0.04060000	0.05970000	NM_024007	downstream	78582	Hs.657753	EBF1
rs285321	3	36225045	0.1907	94.74%	0.428600	0.822500	0.04060000	0.12550000	NM_003149	upstream	172056	Hs.56045	STAC
rs6827691	4	102641036	0.0434	92.98%	1.000000	1.000000	0.04060000	0.08396000	NM_000944	upstream	153660	Hs.435512	PPP3CA
rs6677149	1	111195236	0.4477	97.19%	0.182800	0.073420	0.04061000	0.04364000	ENST00000361218	upstream	3208	---	---
rs7423586	2	195751198	0.0792	90.88%	0.000277	0.479800	0.04061000	0.21830000	ENST00000365336	upstream	335809	---	---
rs10498812	6	56792169	0.1205	97.54%	1.000000	1.000000	0.04062000	0.03754000	NM_020388	intron	0	Hs.631992	DST
rs2858087	4	3238690	0.4424	97.54%	0.808000	0.549700	0.04065000	0.08793000	NM_001012982	downstream	3206	Hs.442291	LOC345222
rs10492741	13	44426505	0.0060	88.42%	0.005964	1.000000	0.04068000	0.99870000	NM_012345	intron	0	Hs.525006	NUFIP1
rs1365225	15	24495712	0.3455	96.49%	0.061620	0.053570	0.04068000	0.08763000	NM_021912	intron	0	Hs.302352	GABRB3
rs8018626	14	78130702	0.0424	95.09%	1.000000	1.000000	0.04068000	0.03342000	ENST00000330071	intron	0	---	---
rs314564	13	88696334	0.0863	97.54%	1.000000	0.564300	0.04069000	0.04689000	NM_015567	downstream	1566462	Hs.591208	SLITRK5
rs4972613	2	174456595	0.3893	85.61%	0.003094	0.057410	0.04070000	0.13180000	NM_001017371	downstream	24910	Hs.531587	SP3
rs10499687	7	49804234	0.0873	96.49%	0.443600	1.000000	0.04071000	0.01384000	NM_198570	intron	0	Hs.677488	VWC2
rs7753540	6	153629421	0.0439	91.93%	0.396300	0.437400	0.04071000	0.09230000	ENST00000312401	downstream	15694	---	---
rs9294633	6	65883178	0.1974	95.09%	0.122300	0.301800	0.04071000	0.09135000	ENST00000370616	upstream	58839	---	---
rs10490430	2	16977626	0.2177	87.02%	0.024410	0.119300	0.04072000	0.07750000	ENST00000386331	downstream	77870	---	---
rs2034900	5	78351720	0.2133	97.89%	0.596300	0.837500	0.04073000	0.01477000	NM_013391	intron	0	Hs.655653	DMGDH
rs2170116	8	16157007	0.1187	97.54%	0.245500	0.347900	0.04077000	0.37180000	NM_138716	upstream	62412	Hs.147635	MSR1
rs719993	4	15076133	0.2122	97.54%	0.858000	0.623000	0.04077000	0.01998000	NM_031911	downstream	19248	Hs.153714	C1QTNF7
rs756451	5	85703846	0.2122	97.54%	0.720000	1.000000	0.04077000	0.01298000	NM_001867	upstream	245631	Hs.430075	COX7C
rs1889900	9	26779975	0.0781	94.39%	1.000000	1.000000	0.04078000	0.08145000	NM_024828	downstream	50708	Hs.178357	C9orf82
rs1102025	1	157150039	0.1625	97.19%	0.186300	0.536700	0.04079000	0.02624000	NM_198930	upstream	17935	Hs.380250	PYHIN1
rs10510027	10	118859597	0.2885	97.89%	0.384800	0.863100	0.04082000	0.04431000	NM_199131	downstream	18425	Hs.681703	VAX1
rs1316576	6	54273276	0.3158	93.33%	0.022550	0.090720	0.04085000	0.00259600	NM_014464	upstream	7886	Hs.127011	TINAG
rs10493894	1	98038255	0.1554	88.07%	0.631000	0.419300	0.04086000	0.02208000	NM_000110	intron	0	Hs.335034	DPYD
rs6503350	17	10874259	0.0784	94.04%	0.209500	0.203600	0.04086000	0.05991000	ENST00000386899	upstream	127238	---	---
rs6798938	3	114700896	0.1697	95.09%	0.385800	0.479300	0.04087000	0.00911000	NM_144718	intron	0	Hs.477144	CCDC52
rs10495541	2	6684559	0.0973	77.54%	0.039020	0.574700	0.04088000	0.04089000	NM_001001695	downstream	102224	---	FLJ42418
rs1564559	4	86778363	0.1082	94.04%	0.527500	0.493000	0.04088000	0.18670000	NM_001025616	intron	0	Hs.444229	ARHGAP24
rs10507494	13	41146016	0.0056	94.39%	1.000000	1.000000	0.04089000	0.99900000	NM_015058	intron	0	Hs.368282	KIAA0564
rs10518122	4	75542740	0.0056	94.39%	1.000000	1.000000	0.04089000	0.99870000	NM_001657	upstream	157123	Hs.270833	AREG
rs10489854	1	157515483	0.0658	93.33%	0.090990	0.335000	0.04090000	0.06437000	ENST00000368119	CDS	0	---	---
rs1184776	12	79798196	0.4271	84.21%	0.005601	0.002551	0.04090000	0.01867000	NM_004664	intron	0	Hs.144333	LIN7A
rs1536447	10	97250554	0.0737	97.54%	0.649500	1.000000	0.04090000	0.06734000	NM_001034957	intron	0	Hs.696027	SORBS1
rs2770	6	31429786	0.2182	96.49%	0.002403	0.011850	0.04092000	0.09901000	NM_005514	3UTR	0	Hs.77961	HLA-B
rs231683	17	3155100	0.4618	96.49%	0.808200	0.282200	0.04094000	0.02451000	NM_002551	intron	0	Hs.647414	OR3A2
rs10486429	7	24710003	0.0432	93.33%	1.000000	1.000000	0.04095000	0.04886000	NM_004403	intron	0	Hs.520708	DFNA5
rs10498141	2	223251126	0.1062	95.79%	0.521600	0.489600	0.04095000	0.03351000	NM_058165	intron	0	Hs.344090	MOGAT1
rs2394325	10	68582152	0.0775	95.09%	1.000000	1.000000	0.04095000	0.00828000	NM_013266	intron	0	Hs.660362	CTNNA3
rs413764	4	167456005	0.0759	94.74%	0.655400	0.647400	0.04096000	0.07044000	NM_016950	downstream	435162	Hs.481133	SPOCK3

rs494338	8	20867328	0.4526	81.40%	0.033630	0.028580	0.04096000	0.10360000	ENST00000387608	upstream	350611	---	---
rs743136	1	184259951	0.4657	97.19%	0.810200	1.000000	0.04098000	0.13200000	NM_031935	intron	0	Hs.58877	HMCN1
rs2065042	20	52882032	0.4640	97.54%	0.717900	0.551900	0.04103000	0.03855000	NM_018431	downstream	180915	Hs.656582	DOK5
rs7540176	1	42095740	0.2034	92.28%	1.000000	0.517100	0.04103000	0.09776000	NM_024503	intron	0	Hs.591503	HIVEP3
rs4781461	16	13401842	0.1877	97.19%	0.234400	0.186100	0.04106000	0.05517000	NM_005236	upstream	519682	Hs.696757	ERCC4
rs966058	13	75052056	0.3866	94.39%	0.367800	0.744600	0.04106000	0.14810000	NM_006002	intron	0	Hs.162241	UCHL3
rs10511072	3	85884225	0.1312	92.28%	1.000000	0.697400	0.04107000	0.13130000	NM_153184	intron	0	Hs.164578	CADM2
rs1073231	3	176610769	0.1033	96.84%	1.000000	0.609600	0.04107000	0.06254000	NM_207015	intron	0	Hs.660010	NAALADL2
rs10494154	1	113665954	0.0778	94.74%	0.206200	0.467500	0.04109000	0.10750000	NM_020965	upstream	68940	---	MAGI3
rs7959125	12	79999295	0.1269	94.04%	0.591300	0.770800	0.04109000	0.04565000	NM_024560	intron	0	Hs.259559	ACSS3
rs1029707	7	108028327	0.3893	95.09%	0.701400	0.538200	0.04110000	0.04160000	NM_012328	downstream	25797	Hs.6790	DNAJB9
rs10490057	2	59518115	0.0405	77.89%	1.000000	1.000000	0.04110000	0.02847000	ENST00000365640	downstream	115190	---	---
rs10492316	12	99417212	0.0660	92.98%	0.312100	0.336600	0.04112000	0.04308000	NM_005123	intron	0	Hs.282735	NR1H4
rs362876	6	146715429	0.0059	89.12%	0.005917	1.000000	0.04112000	0.99850000	NM_000838	intron	0	Hs.32945	GRM1
rs3942865	6	55363787	0.3081	95.09%	0.392000	0.612900	0.04114000	0.31380000	NM_207410	intron	0	Hs.526967	GFRAL
rs1040903	6	111576846	0.4535	90.53%	0.317300	0.157400	0.04117000	0.06634000	NM_018593	intron	0	Hs.591327	SLC16A10
rs1874744	3	112666598	0.1113	92.98%	0.544600	0.506900	0.04117000	0.03252000	NM_005816	upstream	76948	Hs.142023	CD96
rs10489855	1	157515811	0.0653	94.04%	0.089360	0.333400	0.04118000	0.04586000	ENST00000368119	CDS	0	---	---
rs205953	6	118592435	0.0241	94.74%	1.000000	1.000000	0.04121000	0.04983000	NM_001029858	intron	0	Hs.654841	SLC35F1
rs1949271	13	60478385	0.3959	94.39%	0.612500	1.000000	0.04122000	0.08261000	NM_022843	downstream	403434	Hs.391781	PCDH20
rs10491297	5	141868222	0.0866	97.19%	0.441100	0.566500	0.04124000	0.02514000	NM_033137	downstream	85085	Hs.483635	FGF1
rs10506595	12	69191621	0.1292	93.68%	0.784100	0.555400	0.04124000	0.05191000	NM_002837	downstream	9610	Hs.434375	PTPRB
rs1427478	7	33987672	0.3844	78.95%	0.575000	0.866000	0.04124000	0.07114000	NM_133468	intron	0	Hs.660998	BMPER
rs327973	9	106996520	0.1630	96.84%	0.266800	0.049700	0.04124000	0.19490000	NM_080546	upstream	50204	Hs.573495	SLC44A1
rs1406436	18	25176153	0.4748	97.54%	0.030090	0.068940	0.04126000	0.05115000	ENST00000384802	downstream	145663	---	---
rs2589765	8	35483073	0.0765	94.04%	0.188700	0.443400	0.04126000	0.10720000	ENST00000380061	intron	0	---	---
rs868973	18	45909391	0.0740	97.19%	1.000000	0.431600	0.04126000	0.23390000	ENST00000285039	intron	0	---	---
rs2715158	7	82307542	0.3477	97.89%	0.290500	0.488800	0.04127000	0.11160000	ENST00000389477	intron	0	---	---
rs4650672	1	183171589	0.3953	88.77%	0.792500	0.352800	0.04127000	0.06781000	NM_052966	intron	0	Hs.518662	FAM129A
rs173671	5	12217918	0.2695	94.39%	0.090060	0.070210	0.04128000	0.05180000	NM_001332	upstream	260808	Hs.314543	CTNND2
rs9293886	6	63339439	0.3680	69.12%	0.003596	0.061360	0.04128000	0.00321900	NM_152688	upstream	285348	Hs.519794	KHDRBS2
rs308690	3	162965339	0.4855	96.49%	0.717900	0.221300	0.04129000	0.03479000	ENST00000327928	downstream	260915	---	---
rs1073251	2	148179531	0.2045	92.63%	0.850100	0.605000	0.04131000	0.02613000	NM_001616	upstream	139536	Hs.470174	ACVR2A
rs1434406	4	158651695	0.1418	96.49%	0.456700	0.715800	0.04131000	0.02410000	NM_000826	downstream	145018	Hs.32763	GRIA2
rs1957480	14	43467640	0.3185	94.74%	0.779700	0.856700	0.04131000	0.09128000	NM_152447	downstream	2024142	Hs.136893	LRFN5
rs2141767	3	177333015	0.3459	97.89%	0.791700	1.000000	0.04135000	0.06004000	ENST00000366444	downstream	104617	---	---
rs10516763	4	87171775	0.1093	94.74%	0.221400	0.316200	0.04138000	0.11810000	NM_138981	intron	0	Hs.125503	MAPK10
rs2395692	6	38232057	0.1097	94.39%	1.000000	0.742400	0.04139000	0.01978000	NM_021943	downstream	1682	Hs.36959	ZFAND3
rs6602102	10	16446830	0.0057	92.98%	1.000000	1.000000	0.04140000	0.99900000	ENST00000386700	downstream	33830	---	---
rs10493182	1	56108530	0.0923	95.09%	0.482800	0.229400	0.04144000	0.05899000	NM_207465	downstream	138150	---	---
rs10496576	2	122681870	0.0923	95.09%	0.482800	1.000000	0.04144000	0.12000000	NM_004622	downstream	442457	Hs.75066	TSN
rs10515993	18	60222306	0.0923	95.09%	0.482800	1.000000	0.04144000	0.20920000	ENST00000387753	upstream	34393	---	---
rs1368909	2	80082251	0.4532	97.54%	0.228900	0.371000	0.04146000	0.06347000	NM_004389	intron	0	Hs.167368	CTNNA2
rs531981	1	238012046	0.2491	97.89%	0.148800	0.382300	0.04146000	0.14660000	NM_000740	intron	0	Hs.7138	CHRM3
rs590246	7	42934785	0.2491	97.89%	0.630700	0.831000	0.04146000	0.04277000	NM_002787	intron	0	Hs.333786	PSMA2
rs283623	5	73234405	0.3720	89.12%	0.286000	0.493100	0.04149000	0.01697000	ENST00000287898	intron	0	---	---
rs10498411	14	48158503	0.0344	91.93%	1.000000	1.000000	0.04150000	0.05674000	ENST00000384636	upstream	278218	---	---
rs10492932	1	5153405	0.1845	88.42%	0.532000	0.813400	0.04151000	0.06185000	NM_001042478	downstream	415691	Hs.25924	AJAP1
rs10494720	1	193309148	0.4747	97.19%	0.279000	0.881100	0.04151000	0.03115000	NM_198503	downstream	1152388	Hs.657046	KCNT2
rs2068071	6	21184770	0.4427	97.89%	0.546300	0.134400	0.04151000	0.01294000	NM_017774	intron	0	Hs.657604	CDKAL1
rs968136	15	87870512	0.4427	97.89%	0.466300	0.294700	0.04151000	0.31820000	NM_016321	upstream	29694	Hs.459284	RHCG
rs4074170	17	25405267	0.4946	97.54%	0.632500	1.000000	0.04152000	0.10680000	NM_001033562	CDS	0	---	EFCAB5
rs4332647	13	58055006	0.0792	92.98%	0.670900	1.000000	0.04152000	0.25910000	ENST00000365514	upstream	55120	---	---
rs8192981	9	13671648	0.2184	97.19%	0.860000	0.628600	0.04152000	0.00548000	NM_003829	upstream	431291	Hs.169378	MPDZ

rs10494721	1	193309689	0.3569	96.84%	0.693800	0.611600	0.04153000	0.03541000	NM_198503	downstream	1151847	Hs.657046	KCNT2
rs2883953	8	114570395	0.3870	94.74%	0.123500	0.440100	0.04153000	0.06115000	NM_052900	upstream	51977	Hs.91381	CSMD3
rs2245747	10	42236621	0.2000	94.74%	0.702900	1.000000	0.04154000	0.03360000	ENST00000345581	intron	0	---	---
rs10499210	6	139626307	0.0240	95.09%	1.000000	1.000000	0.04158000	0.07023000	NM_153235	intron	0	Hs.535820	TXLNB
rs1372218	5	112020387	0.4873	96.49%	0.070040	0.447800	0.04158000	0.20870000	NM_000038	upstream	81096	Hs.158932	APC
rs37684	5	59769122	0.4117	93.33%	0.526500	0.630200	0.04158000	0.02134000	ENST00000356568	downstream	6426	---	---
rs1342947	1	156726338	0.4711	97.19%	0.227700	0.445400	0.04159000	0.25680000	NM_001004472	downstream	9039	Hs.704755	OR10R2
rs1384747	8	90213747	0.3525	97.54%	0.792700	0.393200	0.04161000	0.07575000	NM_003821	upstream	625426	Hs.103755	RIPK2
rs7861646	9	23722000	0.3525	97.54%	0.694800	0.231800	0.04161000	0.07536000	NM_004432	intron	0	Hs.166109	ELAVL2
rs728611	1	19821094	0.1474	94.04%	0.624400	0.593000	0.04162000	0.06212000	NM_001032363	intron	0	Hs.466662	C1orf151
rs10508855	10	37110805	0.1493	94.04%	0.148300	0.483700	0.04163000	0.17830000	NM_052997	upstream	343986	Hs.373787	ANKRD30A
rs3849400	2	22766039	0.3962	92.98%	0.898500	0.875800	0.04163000	0.02127000	ENST00000388128	upstream	171892	---	---
rs10484899	6	108661772	0.1074	94.74%	1.000000	1.000000	0.04164000	0.22940000	NM_003795	intron	0	Hs.12102	SNX3
rs273884	1	97045901	0.3923	86.32%	0.001292	0.000181	0.04164000	0.07643000	NM_021190	intron	0	Hs.591430	PTBP2
rs7176275	15	91700530	0.1074	94.74%	0.208200	0.166000	0.04164000	0.14220000	ENST00000386183	downstream	63984	---	---
rs10519336	5	112508003	0.2041	93.68%	0.087370	0.071400	0.04165000	0.01457000	NM_002387	intron	0	Hs.593171	MCC
rs10489340	1	207864691	0.0340	92.98%	1.000000	1.000000	0.04166000	0.07212000	NM_001017402	intron	0	Hs.497636	LAMB3
rs10486584	7	28443038	0.3755	94.39%	0.795200	0.865500	0.04167000	0.23240000	NM_001011666	intron	0	Hs.437075	CREB5
rs858792	1	165025361	0.0888	84.91%	0.412100	0.539100	0.04167000	0.03749000	NM_017542	upstream	49987	Hs.432752	POGK
rs229750	14	44328959	0.0336	94.04%	0.255600	0.300000	0.04171000	0.06058000	NM_001017923	upstream	107298	Hs.82098	C14orf28
rs10495927	2	46206003	0.2268	94.39%	0.727600	0.314200	0.04172000	0.02039000	NM_005400	intron	0	Hs.580351	PRKCE
rs10514242	5	81994082	0.2960	97.19%	0.002378	0.000641	0.04172000	0.22700000	NM_001039779	downstream	275991	---	FLJ41309
rs153235	5	80463678	0.2960	97.19%	0.193700	0.495600	0.04172000	0.07331000	NM_006909	intron	0	Hs.162129	RASGRF2
rs2103084	3	192816407	0.2268	94.39%	0.863000	0.546400	0.04172000	0.01229000	ENST00000364990	upstream	25212	---	---
rs2956147	15	31046945	0.2960	97.19%	0.470600	0.495600	0.04172000	0.13470000	ENST00000334528	intron	0	---	---
rs4657796	1	193319560	0.4748	97.54%	0.335600	0.880800	0.04172000	0.02982000	NM_198503	downstream	1141976	Hs.657046	KCNT2
rs10513138	5	13454407	0.0061	86.67%	1.000000	1.000000	0.04173000	0.99870000	NM_001369	downstream	289030	Hs.212360	DNAH5
rs947268	1	120178000	0.1920	87.72%	0.023600	0.169100	0.04174000	0.16570000	NM_032044	upstream	22194	Hs.660883	REG4
rs10507902	13	79513112	0.0272	90.18%	1.000000	1.000000	0.04175000	0.02841000	NM_005842	downstream	295000	Hs.18676	SPRY2
rs10489716	1	184040194	0.0258	95.09%	1.000000	1.000000	0.04177000	0.07474000	NM_031935	intron	0	Hs.58877	HMCN1
rs10507898	13	78805175	0.0258	95.09%	1.000000	1.000000	0.04177000	0.02075000	NM_022118	intron	0	Hs.558528	RBM26
rs2053848	13	38142418	0.3403	92.28%	0.583000	0.721800	0.04178000	0.03157000	NM_207361	upstream	17064	Hs.253994	FREM2
rs1396571	12	126165117	0.1882	97.89%	1.000000	0.786600	0.04180000	0.07067000	ENST00000386224	downstream	51289	---	---
rs9323656	14	77394323	0.0327	96.49%	1.000000	1.000000	0.04180000	0.06578000	NM_020421	intron	0	Hs.413208	ADCK1
rs10484057	14	98010218	0.0324	97.54%	1.000000	1.000000	0.04181000	0.01648000	NM_182560	upstream	237485	Hs.448754	C14orf177
rs10490396	2	56317650	0.0056	93.68%	0.005629	1.000000	0.04181000	0.99860000	ENST00000233161	intron	0	---	---
rs947861	11	85005633	0.2452	91.58%	0.018430	0.039290	0.04182000	0.23070000	NM_018480	upstream	11663	Hs.525063	TMEM126B
rs544080	13	95474210	0.4695	97.89%	0.810000	0.881100	0.04183000	0.06535000	NM_020121	intron	0	Hs.193226	UGCGL2
rs887695	2	191201411	0.1488	84.91%	0.798300	1.000000	0.04184000	0.02812000	NM_005966	upstream	20682	Hs.570078	NAB1
rs309762	4	177727556	0.1189	85.61%	1.000000	0.740600	0.04188000	0.03804000	NM_005429	downstream	114129	Hs.435215	VEGFC
rs1982939	12	129433601	0.0926	94.74%	1.000000	0.602900	0.04189000	0.04874000	NM_004764	downstream	10776	Hs.405659	PIWIL1
rs7158689	14	87575945	0.0926	94.74%	0.484400	0.231400	0.04189000	0.01618000	NM_003608	downstream	27781	Hs.513440	GPR65
rs10485214	6	153740608	0.1619	97.54%	0.383000	0.536700	0.04190000	0.03539000	ENST00000364238	upstream	42659	---	---
rs6795968	3	23392031	0.3502	97.19%	0.008368	0.055240	0.04190000	0.01174000	NM_152653	intron	0	Hs.475688	UBE2E2
rs10505852	12	19787991	0.0242	94.39%	1.000000	1.000000	0.04192000	0.17200000	NM_000921	upstream	625495	Hs.591150	PDE3A
rs10485262	6	154181892	0.1423	96.14%	1.000000	1.000000	0.04193000	0.02540000	NM_001008505	upstream	220244	Hs.2353	OPRM1
rs7155413	14	61016413	0.0057	91.58%	0.005758	1.000000	0.04193000	0.99840000	NM_006255	intron	0	Hs.333907	PRKCH
rs354532	6	111655506	0.4570	97.89%	0.398300	0.646200	0.04194000	0.05465000	NM_018593	downstream	3668	Hs.591327	SLC16A10
rs4144592	9	121018601	0.4570	97.89%	0.229000	0.361000	0.04194000	0.07896000	NM_014618	intron	0	Hs.532316	DBC1
rs7979386	12	30966129	0.3327	92.28%	0.210500	0.740300	0.04195000	0.04905000	ENST00000261177	upstream	32064	---	---
rs10518772	15	52655153	0.0262	93.68%	1.000000	1.000000	0.04199000	0.09026000	ENST00000260323	intron	0	---	---
rs10517855	4	63802754	0.0434	92.98%	1.000000	1.000000	0.04200000	0.05960000	NM_001010874	downstream	1025631	Hs.227752	SRD5A2L2
rs2080326	2	202056548	0.4729	97.19%	0.717700	1.000000	0.04201000	0.09606000	NM_018571	downstream	2853	Hs.652338	ALS2CR2
rs977274	8	95348936	0.4049	86.67%	0.693000	0.867300	0.04202000	0.03668000	NM_181702	upstream	5203	Hs.654463	GEM

rs1951549	14	43389373	0.4427	91.93%	0.707500	0.341900	0.04204000	0.09141000	NM_152447	downstream	1945875	Hs.136893	LRFN5
rs10489774	1	56290538	0.4916	83.86%	0.069710	0.340500	0.04205000	0.05000000	NM_207465	downstream	320158	---	---
rs1174600	12	57021979	0.4267	93.33%	0.210100	0.346000	0.04205000	0.07405000	NM_153377	downstream	530226	Hs.253736	LRIG3
rs10485147	6	84180069	0.4910	97.54%	0.718800	0.366500	0.04207000	0.07796000	NM_002395	intron	0	Hs.21160	ME1
rs950487	1	156726754	0.4713	97.89%	0.186100	0.362700	0.04207000	0.25610000	NM_001004472	downstream	9455	Hs.704755	OR10R2
rs10509024	10	56567832	0.0323	97.89%	1.000000	1.000000	0.04210000	0.01116000	NM_033056	upstream	336775	Hs.672170	PCDH15
rs2414367	15	52790015	0.0323	97.89%	0.026580	1.000000	0.04210000	0.21940000	NM_016304	downstream	470798	Hs.274772	C15orf15
rs7668692	4	65933645	0.0187	94.04%	1.000000	1.000000	0.04210000	0.03740000	NM_182472	intron	0	Hs.654492	EPHA5
rs9320607	6	117963564	0.0323	97.89%	0.246600	1.000000	0.04210000	0.03260000	NM_173674	intron	0	Hs.658304	DCBLD1
rs10515799	5	158919215	0.1493	94.04%	0.811200	0.789700	0.04211000	0.17150000	NM_002187	upstream	229156	Hs.674	IL12B
rs1612279	4	161243608	0.3610	97.19%	0.514600	0.529600	0.04215000	0.01590000	ENST00000385897	downstream	462028	---	---
rs10515961	18	49185236	0.3296	93.68%	0.053250	0.188300	0.04217000	0.09758000	NM_005215	intron	0	Hs.579550	DCC
rs9297381	8	107403507	0.2582	95.79%	0.080940	0.095380	0.04219000	0.17760000	NM_181354	upstream	335763	Hs.148778	OXR1
rs1323802	9	9791153	0.1403	88.77%	0.117900	0.051440	0.04221000	0.11240000	ENST00000363183	downstream	358801	---	---
rs1916961	2	189300129	0.0894	96.14%	0.248800	0.432200	0.04221000	0.07904000	NM_052952	downstream	6628	Hs.470892	DIRC1
rs7122992	11	33993259	0.0653	94.04%	0.612200	1.000000	0.04221000	0.01204000	NM_203364	upstream	37285	Hs.471818	CAPRIN1
rs10487525	7	144463196	0.2080	91.93%	0.710100	0.671700	0.04222000	0.03341000	ENST00000363803	upstream	285086	---	---
rs1482650	3	43459673	0.2360	93.68%	0.397700	0.357200	0.04222000	0.07649000	NM_018075	intron	0	Hs.656657	TMEM16K
rs2890898	9	9964221	0.1996	94.04%	0.849900	0.662900	0.04222000	0.21150000	ENST00000363183	downstream	531869	---	---
rs7341478	7	81592385	0.2263	96.14%	1.000000	0.843100	0.04223000	0.02723000	NM_000722	intron	0	Hs.282151	CACNA2D1
rs31564	5	135258152	0.3868	92.98%	0.436700	0.271600	0.04224000	0.04094000	NM_000590	intron	0	Hs.960	IL9
rs1047022	7	26546774	0.1403	97.54%	0.802700	1.000000	0.04225000	0.05382000	NM_003930	downstream	126441	Hs.200770	SKAP2
rs4294760	15	51573583	0.2168	97.89%	0.293900	0.807900	0.04225000	0.06247000	NM_182758	downstream	21090	Hs.122125	WDR72
rs4538888	8	9254784	0.1403	97.54%	0.453500	0.420600	0.04225000	0.01834000	ENST00000382558	upstream	6976	---	---
rs6721063	2	151628774	0.1403	97.54%	0.621000	0.579500	0.04225000	0.07083000	NM_198557	downstream	185382	Hs.302442	RBM43
rs702974	16	78204225	0.2168	97.89%	0.483800	0.628900	0.04225000	0.05022000	NM_001031804	upstream	12113	Hs.134859	MAF
rs7513705	1	93438937	0.4676	97.54%	0.022230	0.071540	0.04226000	0.02529000	NM_206886	intron	0	Hs.310637	CCDC18
rs4886239	13	60017463	0.4808	91.23%	0.083630	0.122200	0.04228000	0.01992000	NM_030794	intron	0	Hs.525061	TDRD3
rs529579	19	39360134	0.3750	95.44%	1.000000	0.737300	0.04228000	0.15440000	NM_015578	intron	0	Hs.407368	LSM14A
rs1863440	8	81222113	0.3647	89.47%	0.224000	0.607300	0.04229000	0.07137000	NM_005079	intron	0	Hs.368433	TPD52
rs6712682	2	151632773	0.1416	97.89%	0.621500	0.421100	0.04231000	0.06253000	NM_198557	downstream	181383	Hs.302442	RBM43
rs1196155	2	182629517	0.2563	97.19%	0.755000	1.000000	0.04235000	0.01636000	ENST00000280295	intron	0	---	---
rs2911524	13	69326151	0.2563	97.19%	1.000000	1.000000	0.04235000	0.09972000	NM_020866	intron	0	Hs.508201	KLHL1
rs10492626	13	93941954	0.2168	89.82%	0.356500	0.802100	0.04236000	0.06828000	NM_001922	upstream	12030	Hs.301865	DCT
rs1343982	1	65817839	0.3305	83.86%	0.056530	0.117100	0.04237000	0.12780000	NM_001003680	intron	0	Hs.705413	LEPR
rs341148	6	158759986	0.1563	89.82%	0.231000	0.036920	0.04238000	0.05097000	NM_001007466	intron	0	Hs.486993	TULP4
rs1651215	4	161233738	0.3587	96.84%	0.600700	0.636100	0.04240000	0.01737000	ENST00000385897	downstream	471898	---	---
rs2903581	16	14247571	0.0338	93.33%	0.257300	0.300000	0.04241000	0.11060000	NM_014048	intron	0	Hs.592047	MKL2
rs1156605	3	25998305	0.3000	89.47%	0.766000	1.000000	0.04242000	0.08624000	NM_017897	downstream	187277	Hs.55781	OXSM
rs1444504	8	93348728	0.4874	97.19%	0.030240	0.050620	0.04242000	0.03507000	ENST00000363950	upstream	99774	---	---
rs1504910	3	59585967	0.1631	97.89%	0.270900	0.458100	0.04245000	0.10310000	NM_002012	downstream	127025	Hs.655995	FHIT
rs9309326	2	60478443	0.2731	95.09%	0.362500	1.000000	0.04245000	0.02964000	NM_018014	downstream	53363	Hs.370549	BCL11A
rs1514540	13	60483549	0.4030	94.04%	0.611500	0.871200	0.04246000	0.06848000	NM_022843	downstream	398270	Hs.391781	PCDH20
rs10499942	7	97621592	0.1934	96.14%	0.330700	0.124100	0.04248000	0.00647700	NM_014916	intron	0	Hs.444179	LMTK2
rs1188306	8	13110780	0.2397	93.68%	0.738600	0.111800	0.04248000	0.08920000	NM_182643	intron	0	Hs.134296	DLC1
rs716077	8	131194895	0.2333	94.74%	1.000000	1.000000	0.04248000	0.23480000	NM_018482	intron	0	Hs.655552	DDEF1
rs10504003	8	37247698	0.3359	90.88%	0.036140	0.359300	0.04249000	0.34210000	NM_021631	downstream	381712	Hs.651853	FKSG2
rs10516511	4	106040880	0.4942	60.35%	0.000000	0.000000	0.04250000	0.08162000	NM_025212	upstream	405380	Hs.12248	CXXC4
rs1322960	13	52650341	0.4005	68.77%	0.766300	0.716900	0.04250000	0.02007000	NM_006418	downstream	126154	Hs.559736	OLFM4
rs10487920	7	146089012	0.1421	97.54%	0.012250	0.029620	0.04252000	0.10200000	ENST00000307013	intron	0	---	---
rs1846466	3	1167829	0.1421	97.54%	0.806300	1.000000	0.04252000	0.01433000	NM_014461	intron	0	Hs.387300	CNTN6
rs6541279	1	229820086	0.4885	91.23%	0.025930	0.046570	0.04252000	0.03478000	ENST00000366656	intron	0	---	---
rs425567	21	17150110	0.3107	95.44%	0.573500	0.313600	0.04255000	0.34300000	ENST00000365203	downstream	136763	---	---
rs10517469	4	37773873	0.0938	78.60%	0.110400	0.399600	0.04256000	0.09223000	NM_015173	intron	0	Hs.176503	TBC1D1

rs473968	11	84688230	0.2600	96.49%	0.275600	0.831100	0.04257000	0.07469000	NM_001364	upstream	982394	Hs.654862	DLG2
rs6737341	2	119040468	0.0098	89.47%	1.000000	1.000000	0.04257000	0.15320000	NM_001426	downstream	275768	Hs.271977	EN1
rs9317825	13	69043126	0.2229	87.37%	1.000000	0.680600	0.04257000	0.20300000	NM_020866	downstream	129600	Hs.508201	KLHL1
rs10496818	2	139849720	0.1369	88.42%	1.000000	1.000000	0.04259000	0.10700000	ENST00000384174	upstream	135744	---	---
rs2961370	12	19431361	0.3043	96.84%	0.016020	0.003882	0.04259000	0.01005000	NM_019012	downstream	10763	Hs.188614	PLEKHA5
rs10486009	7	97316047	0.1643	97.19%	1.000000	0.630300	0.04260000	0.08492000	NM_183356	downstream	3332	Hs.489207	ASNS
rs546848	6	81285137	0.4245	92.98%	0.380500	0.645500	0.04265000	0.03305000	NM_000056	downstream	172447	Hs.654441	BCKDHB
rs475348	9	103219299	0.4874	97.54%	1.000000	1.000000	0.04266000	0.11960000	NM_000035	downstream	3382	Hs.530274	ALDOB
rs10505504	8	128769526	0.4198	94.04%	0.617500	0.170900	0.04267000	0.02725000	NM_002467	upstream	47972	Hs.202453	MYC
rs7563206	2	215898899	0.4033	94.39%	0.447000	0.282600	0.04270000	0.06242000	NM_004044	intron	0	Hs.90280	ATIC
rs10494700	1	192400158	0.1825	92.28%	0.541800	0.355500	0.04272000	0.03938000	ENST00000385495	downstream	323698	---	---
rs2076635	6	52523998	0.1754	94.04%	1.000000	1.000000	0.04274000	0.16150000	NM_012288	intron	0	Hs.520182	TRAM2
rs10497658	2	185354265	0.0255	96.14%	1.000000	1.000000	0.04275000	0.01549000	NM_194250	intron	0	Hs.159528	ZNF804A
rs10516882	4	92127599	0.1832	91.93%	0.149000	0.259400	0.04275000	0.01897000	NM_207491	intron	0	Hs.654735	MGC48628
rs9287868	2	167135645	0.0255	96.14%	0.000268	0.028210	0.04275000	0.08514000	NM_002976	upstream	83918	Hs.695947	SCN7A
rs10484168	14	96633495	0.0330	95.79%	0.027700	0.059640	0.04276000	0.16940000	NM_003384	downstream	215792	Hs.422662	VRK1
rs1178102	7	18704391	0.3259	94.74%	0.405300	0.623700	0.04278000	0.04337000	NM_178425	intron	0	Hs.196054	HDAC9
rs7187550	16	53230696	0.2204	97.89%	0.161500	0.095650	0.04278000	0.03752000	NM_005853	upstream	291916	Hs.435730	IRX5
rs10244401	7	24409901	0.3628	97.19%	0.193500	0.498300	0.04281000	0.03168000	NM_000905	downstream	111899	Hs.1832	NPY
rs10490029	2	210650144	0.3290	94.39%	0.410500	0.513200	0.04281000	0.04300000	NM_152519	intron	0	Hs.591638	FLJ23861
rs4359155	10	61484423	0.3664	97.19%	0.197900	1.000000	0.04282000	0.04968000	NM_001149	intron	0	Hs.499725	ANK3
rs2116648	7	42499900	0.1000	87.72%	0.721100	0.601200	0.04285000	0.23130000	NM_000168	upstream	270480	Hs.21509	GLI3
rs2256552	21	35028656	0.2332	94.04%	0.865000	0.558300	0.04285000	0.04942000	NM_053277	downstream	16267	Hs.702188	CLIC6
rs1998277	11	34265813	0.2681	87.02%	0.034680	0.085500	0.04286000	0.01063000	NM_145804	intron	0	Hs.23361	ABTB2
rs4739323	8	36663871	0.1741	94.74%	0.523600	1.000000	0.04286000	0.05402000	ENST00000363115	upstream	77250	---	---
rs4743380	9	101847100	0.3095	95.79%	0.574300	0.866100	0.04290000	0.06412000	NM_015051	intron	0	Hs.591899	TXNDC4
rs217692	14	61473002	0.0888	96.84%	0.144700	0.227100	0.04291000	0.02021000	NM_031914	upstream	59489	Hs.404139	SYT16
rs32768	5	35407927	0.1767	93.33%	1.000000	1.000000	0.04292000	0.02957000	NM_000949	upstream	141593	Hs.368587	PRLR
rs10490202	2	102527264	0.2776	85.96%	0.873900	0.463600	0.04293000	0.10560000	NM_001011552	downstream	10405	Hs.447686	SLC9A4
rs503314	2	70528257	0.3713	95.44%	0.697800	0.874100	0.04294000	0.03091000	NM_003236	3UTR	0	Hs.170009	TGFA
rs10250146	7	46981249	0.2815	83.51%	0.003900	0.082720	0.04296000	0.21690000	NM_022748	downstream	300030	Hs.520814	TNS3
rs469783	5	96147280	0.4339	84.91%	0.793400	1.000000	0.04296000	0.15500000	NM_001040458	CDS	0	Hs.436186	ERAP1
rs899467	5	117546401	0.1647	88.42%	0.166100	1.000000	0.04296000	0.18360000	ENST00000329863	upstream	403787	---	---
rs2014195	3	7014450	0.2807	94.39%	0.650100	0.431800	0.04297000	0.04258000	NM_181874	intron	0	Hs.660131	GRM7
rs17757084	16	78159145	0.0395	80.00%	0.002678	0.003923	0.04298000	0.13210000	NM_001031804	downstream	26591	Hs.134859	MAF
rs3856451	2	8704019	0.4978	78.60%	0.230800	0.744100	0.04299000	0.07871000	NM_002166	upstream	32772	Hs.180919	ID2
rs10500766	11	12876485	0.1155	92.63%	1.000000	0.651000	0.04300000	0.01809000	NM_021961	intron	0	Hs.655331	TEAD1
rs2856206	17	15421834	0.2059	83.51%	0.162400	0.413800	0.04300000	0.06285000	ENST00000379628	intron	0	---	---
rs626804	9	9529114	0.1364	88.77%	0.430200	0.564000	0.04301000	0.03935000	ENST00000363183	downstream	96762	---	---
rs2024741	6	107285089	0.4808	91.58%	0.710200	0.354100	0.04302000	0.07952000	NM_018292	downstream	62104	Hs.406917	QRSL1
rs10484261	6	10091850	0.0878	97.89%	1.000000	1.000000	0.04309000	0.03940000	NM_153003	intron	0	Hs.532138	OFCC1
rs10487060	7	86428289	0.0878	97.89%	0.453900	1.000000	0.04309000	0.09693000	NM_152748	upstream	19665	Hs.208093	KIAA1324L
rs2514322	8	99304253	0.0878	97.89%	0.245800	0.115300	0.04309000	0.02420000	NM_024759	intron	0	Hs.309489	NPAL2
rs3118225	1	49237254	0.4126	86.32%	0.087300	0.130700	0.04309000	0.18500000	ENST00000371836	intron	0	---	---
rs997427	5	5220587	0.4093	94.74%	0.078990	0.067340	0.04309000	0.00833400	NM_139056	intron	0	Hs.661915	ADAMTS16
rs10507108	12	98119420	0.1524	94.39%	1.000000	0.794700	0.04310000	0.08082000	NM_020140	intron	0	Hs.506458	ANKS1B
rs1156110	5	12518018	0.2741	94.74%	0.091630	0.048650	0.04311000	0.08760000	NM_001369	downstream	1227021	Hs.212360	DNAH5
rs4904705	14	90094687	0.3664	81.40%	0.887500	0.736700	0.04312000	0.19900000	NM_001010854	intron	0	Hs.655697	TTC7B
rs7620700	3	114753680	0.0894	96.14%	0.049640	0.121200	0.04312000	0.38010000	NM_017699	intron	0	Hs.591291	SIDT1
rs10483668	14	56052341	0.0252	97.54%	1.000000	1.000000	0.04314000	0.04596000	NM_017799	upstream	63923	Hs.497253	C14orf101
rs2269197	5	109064976	0.0252	97.54%	1.000000	1.000000	0.04314000	0.00595100	NM_002372	intron	0	Hs.432822	MAN2A1
rs2613163	18	63652390	0.1500	94.74%	0.633800	0.743800	0.04314000	0.13460000	NM_032160	upstream	317443	Hs.124673	DSEL
rs3901894	13	95204126	0.4113	92.98%	0.799800	1.000000	0.04314000	0.07606000	NM_006260	intron	0	Hs.656476	DNAJC3
rs9287104	1	197898148	0.1578	92.28%	0.247500	0.120600	0.04314000	0.12180000	ENST00000385491	downstream	81203	---	---

rs358523	5	63253368	0.1245	95.79%	0.779500	1.000000	0.04315000	0.02366000	NM_000524	downstream	38666	Hs.247940	HTR1A
rs10499890	7	85032278	0.1111	94.74%	0.549700	0.366700	0.04316000	0.12440000	NM_000840	upstream	1078882	Hs.590575	GRM3
rs10510777	3	55212778	0.4167	94.74%	0.133200	0.345500	0.04317000	0.03247000	NM_018398	downstream	129156	Hs.656687	CACNA2D3
rs4851004	2	102375969	0.4321	92.98%	0.017540	0.045780	0.04318000	0.36690000	NM_003855	intron	0	Hs.469521	IL18R1
rs9315305	13	34170349	0.2481	91.93%	0.618800	0.848500	0.04318000	0.13200000	NM_015678	upstream	244107	Hs.491172	NBEA
rs10510706	3	40075261	0.2636	96.49%	0.217400	0.206400	0.04321000	0.22610000	NM_015460	intron	0	Hs.594535	MYRIP
rs7596444	2	133474230	0.3981	94.74%	0.611500	0.439900	0.04322000	0.09768000	NM_207481	intron	0	Hs.537329	NAP5
rs2824432	21	17963050	0.1920	96.84%	0.171500	0.268200	0.04323000	0.01341000	NM_001012707	upstream	37000	---	C21orf114
rs229271	14	30058777	0.2985	90.53%	0.235500	0.861400	0.04326000	0.08286000	NM_017769	upstream	39351	Hs.509008	KIAA1333
rs7300179	12	71809958	0.2260	87.72%	0.017660	0.045330	0.04326000	0.04257000	NM_013381	downstream	464270	Hs.199814	TRHDE
rs6915203	6	147045441	0.2079	93.68%	0.096310	0.297300	0.04327000	0.13710000	ENST00000326929	intron	0	---	---
rs10497216	2	161296725	0.0903	97.19%	0.143800	0.136500	0.04330000	0.12510000	NM_002897	upstream	238174	Hs.470412	RBMS1
rs1872394	3	7655566	0.2572	97.54%	0.271700	0.525000	0.04333000	0.10540000	NM_181875	intron	0	---	GRM7
rs795446	3	30776092	0.4219	94.39%	0.382000	0.362600	0.04333000	0.01137000	NM_207359	intron	0	Hs.657052	GADL1
rs10516063	5	168711427	0.0368	66.67%	0.219900	0.272300	0.04334000	0.20290000	NM_003062	upstream	51136	Hs.604116	SLIT3
rs763720	10	124252434	0.1716	94.04%	0.522200	0.627000	0.04335000	0.04683000	NM_002775	intron	0	Hs.501280	HTRA1
rs1990140	7	41042219	0.4182	94.39%	0.380100	0.205400	0.04338000	0.04882000	NM_002192	downstream	652907	Hs.583348	INHBA
rs6738407	2	196851876	0.1753	95.09%	0.832900	1.000000	0.04338000	0.22910000	NM_020760	intron	0	Hs.654742	HECW2
rs984040	6	126062970	0.4451	92.63%	0.533300	0.761200	0.04338000	0.03425000	NM_012259	upstream	49449	Hs.144287	HEY2
rs10507742	13	66646843	0.1321	92.98%	0.789400	0.127400	0.04342000	0.09167000	NM_020403	intron	0	Hs.654709	PCDH9
rs10492347	12	89527027	0.0896	97.89%	0.144000	0.232800	0.04345000	0.01093000	ENST00000359455	upstream	329537	---	---
rs3849439	3	96323133	0.0759	94.74%	0.185700	0.443400	0.04345000	0.08657000	ENST00000332119	upstream	183575	---	---
rs6727029	2	75625412	0.0896	97.89%	0.708800	0.698700	0.04345000	0.01368000	NM_032181	intron	0	Hs.302346	TMEM166
rs845006	21	32953727	0.0436	76.49%	0.336200	0.392000	0.04347000	0.11310000	NM_203446	intron	0	Hs.473632	SYNJ1
rs2043555	10	52729605	0.4436	93.33%	0.171900	0.041770	0.04348000	0.14220000	NM_006258	intron	0	Hs.654556	PRKG1
rs10486336	7	19172961	0.2828	93.68%	0.228200	0.112800	0.04351000	0.33980000	NM_152898	upstream	21451	Hs.592168	FERD3L
rs10518439	4	125837632	0.0909	96.49%	0.057450	0.600200	0.04351000	0.04108000	NM_020337	intron	0	Hs.480694	ANKRD50
rs9302644	16	71629089	0.1942	97.54%	0.338500	0.054740	0.04352000	0.11480000	NM_006885	intron	0	Hs.652666	ZFHX3
rs10495071	1	215958401	0.1259	94.74%	0.003656	0.090900	0.04353000	0.04712000	NM_138796	intron	0	Hs.171130	SPATA17
rs1072746	5	43646445	0.4151	95.09%	0.044910	0.049570	0.04353000	0.03189000	NM_182977	intron	0	Hs.482043	NNT
rs434193	4	86472513	0.1925	88.42%	0.153900	0.361200	0.04353000	0.05080000	NM_001042669	upstream	597937	Hs.444229	ARHGAP24
rs1143770	11	121522808	0.3509	92.98%	0.893500	0.224000	0.04354000	0.09085000	ENST00000362105	upstream	285	---	---
rs7025261	9	120112360	0.2519	90.53%	0.408600	1.000000	0.04354000	0.34690000	NM_014618	downstream	856369	Hs.532316	DBC1
rs6709784	2	167495240	0.2618	96.49%	0.211500	0.464100	0.04356000	0.09328000	ENST00000328983	intron	0	---	---
rs1899358	6	165511172	0.2095	84.56%	0.176800	0.193100	0.04357000	0.03589000	NM_144980	downstream	101984	Hs.144734	C6orf118
rs1509478	18	59315112	0.4671	90.53%	1.000000	0.752400	0.04358000	0.01732000	NM_002639	intron	0	Hs.55279	SERPINB5
rs666762	10	113634574	0.0524	93.68%	0.526100	0.547300	0.04359000	0.02809000	NM_020918	downstream	265040	Hs.42586	GPAM
rs718702	12	57021546	0.1591	92.63%	0.495000	1.000000	0.04359000	0.04423000	NM_153377	downstream	530659	Hs.253736	LRIG3
rs1985652	1	115587931	0.4620	92.28%	0.106300	0.129000	0.04360000	0.02800000	NM_002506	downstream	42129	Hs.2561	NGFB
rs10483931	14	79217351	0.0251	97.89%	0.153900	1.000000	0.04361000	0.05112000	NM_138970	intron	0	Hs.368307	NRXN3
rs2983499	6	165953840	0.0251	97.89%	1.000000	1.000000	0.04361000	0.07072000	NM_006661	intron	0	Hs.584856	PDE10A
rs1006212	2	224338947	0.1897	88.77%	0.837800	0.573900	0.04362000	0.23160000	NM_001039569	intron	0	Hs.632555	AP1S3
rs10503806	8	26938920	0.0564	90.18%	0.566900	1.000000	0.04362000	0.05585000	NM_033304	upstream	160317	Hs.654357	ADRA1A
rs8182037	15	73921656	0.2425	93.33%	0.407900	1.000000	0.04363000	0.08078000	NM_173469	upstream	1199	Hs.23033	UBE2Q2
rs997229	6	101143434	0.3858	89.12%	0.895000	1.000000	0.04363000	0.00538900	NM_006828	intron	0	Hs.486031	ASCC3
rs1439162	8	35599896	0.0509	96.49%	0.145500	0.155300	0.04365000	0.05685000	NM_080872	intron	0	Hs.238889	UNC5D
rs4271550	15	43723558	0.4182	94.39%	0.012480	0.026280	0.04366000	0.08153000	NM_021199	intron	0	Hs.511251	SQRDL
rs10508935	10	52536509	0.2815	94.74%	0.132100	0.045680	0.04367000	0.34210000	NM_006258	intron	0	Hs.654556	PRKG1
rs722367	3	4730956	0.1259	96.14%	0.022340	0.069920	0.04367000	0.05527000	NM_002222	intron	0	Hs.699169	ITPR1
rs279091	5	100099358	0.1278	94.74%	0.407800	0.401200	0.04370000	0.09086000	NM_175052	downstream	73898	Hs.308628	ST8SIA4
rs726039	13	103947454	0.0259	94.74%	1.000000	1.000000	0.04370000	0.01637000	NM_172370	upstream	969139	Hs.381382	DAOA
rs10490256	2	40636555	0.1105	93.68%	0.340600	0.498400	0.04371000	0.03422000	NM_021097	upstream	102367	Hs.468274	SLC8A1
rs9318007	13	70773135	0.3745	96.49%	0.370100	0.875800	0.04371000	0.18420000	NM_004392	downstream	136964	Hs.129452	DACH1
rs7541392	1	201006518	0.1574	82.46%	0.005023	0.005056	0.04373000	0.29350000	NM_006618	intron	0	Hs.443650	JARID1B

rs10488170	7	132976542	0.4185	94.74%	0.900500	0.430800	0.04374000	0.06298000	NM_001037126	intron	0	Hs.321273	EXOC4
rs10488821	11	30458751	0.3087	97.19%	0.778800	0.579400	0.04375000	0.01580000	NM_001584	intron	0	Hs.289795	MPPED2
rs4551866	13	25045654	0.1861	63.16%	1.000000	0.791300	0.04376000	0.09634000	NM_016529	intron	0	Hs.444957	ATP8A2
rs606443	12	119395013	0.4844	90.18%	0.060860	0.120300	0.04376000	0.05461000	NM_003769	upstream	3072	Hs.700688	SFRS9
rs1014095	7	148094793	0.2091	92.28%	0.455800	0.671100	0.04380000	0.03248000	NM_003592	intron	0	Hs.146806	CUL1
rs6909559	6	149293111	0.3047	97.89%	0.396800	0.319100	0.04381000	0.22990000	NM_005715	intron	0	Hs.657370	UST
rs6940841	6	149291239	0.3047	97.89%	0.396800	0.319100	0.04381000	0.22990000	NM_005715	intron	0	Hs.657370	UST
rs9320161	6	106957981	0.3849	88.42%	0.002156	0.003637	0.04381000	0.02096000	NM_004849	upstream	77622	Hs.486063	ATG5
rs1110183	9	38446365	0.2004	88.42%	0.323500	0.129200	0.04382000	0.02313000	NM_001007563	upstream	31921	Hs.349705	IGFBPL1
rs7245218	18	49529525	0.4924	92.63%	0.461800	0.447000	0.04384000	0.15020000	NM_005215	downstream	218504	Hs.579550	DCC
rs220799	6	165723490	0.3339	95.09%	0.587200	0.723900	0.04385000	0.04963000	NM_006661	intron	0	Hs.584856	PDE10A
rs10509713	10	99105699	0.0513	95.79%	1.000000	1.000000	0.04386000	0.13550000	NM_015179	downstream	749	Hs.434251	RRP12
rs54566	14	79705600	0.1062	95.79%	0.521600	0.739300	0.04386000	0.05076000	NM_001007023	downstream	30995	Hs.202354	DIO2
rs10483156	22	28599773	0.3232	63.51%	0.003536	0.043590	0.04387000	0.01181000	NM_021090	upstream	9426	Hs.474536	MTMR3
rs10501820	11	94513456	0.0414	97.54%	1.000000	1.000000	0.04387000	0.03037000	NM_144665	downstream	32325	Hs.659934	SESN3
rs7020632	9	31605237	0.4342	85.26%	0.001668	0.003832	0.04388000	0.17940000	ENST00000360120	downstream	360979	---	---
rs10493788	1	86254922	0.1143	90.53%	0.000008	0.000068	0.04389000	0.24620000	NM_152890	intron	0	Hs.659516	COL24A1
rs139954	22	35179861	0.0418	96.49%	0.381100	1.000000	0.04391000	0.02316000	ENST00000381877	intron	0	---	---
rs10516565	4	112377171	0.3755	97.19%	0.307500	0.280000	0.04394000	0.04225000	NM_000325	upstream	613468	Hs.643588	PITX2
rs1847118	4	40185760	0.4291	94.04%	0.708600	0.875700	0.04395000	0.12840000	NM_019027	intron	0	Hs.518727	RBM47
rs949636	10	23741106	0.2048	95.09%	0.134900	0.398500	0.04396000	0.03483000	ENST00000386792	upstream	12572	---	---
rs7910052	10	19739010	0.3787	95.44%	0.441000	0.616600	0.04399000	0.12020000	ENST00000377265	upstream	88412	---	---
rs9357639	6	50038173	0.3023	90.53%	1.000000	0.242700	0.04399000	0.17180000	NM_001037499	intron	0	Hs.381372	DEFB114
rs1003986	3	85953053	0.2444	94.04%	0.408000	0.186900	0.04400000	0.22150000	NM_153184	intron	0	Hs.164578	CADM2
rs10510800	3	58930912	0.0055	95.09%	1.000000	1.000000	0.04400000	0.99870000	NM_198463	intron	0	Hs.368434	C3orf67
rs362620	20	10262383	0.1863	92.28%	0.306600	0.257900	0.04400000	0.19740000	NM_130811	downstream	26318	Hs.167317	SNAP25
rs966287	18	27580619	0.2673	96.49%	0.285200	0.371600	0.04400000	0.05455000	ENST00000269205	downstream	13110	---	---
rs10513506	9	130838960	0.2439	86.32%	0.391600	0.494900	0.04401000	0.05179000	ENST00000277475	intron	0	---	---
rs950214	10	64337410	0.2230	97.54%	0.728800	1.000000	0.04404000	0.04191000	NM_000399	upstream	91277	Hs.1395	EGR2
rs952435	19	5622821	0.1909	89.12%	1.000000	0.498700	0.04405000	0.05107000	NM_002967	downstream	3333	Hs.23978	SAFB
rs1974584	4	10166959	0.3383	94.39%	0.342100	0.144500	0.04406000	0.06728000	NM_052964	intron	0	Hs.678910	MIST
rs2619541	6	15731919	0.1065	97.19%	0.528500	0.490700	0.04406000	0.11450000	NM_183041	intron	0	Hs.571148	DTNBP1
rs321989	7	78133650	0.1032	88.42%	1.000000	1.000000	0.04407000	0.07481000	NM_012301	intron	0	Hs.654788	MAGI2
rs9320189	6	107285235	0.4773	92.63%	0.901800	0.441400	0.04407000	0.09027000	NM_018292	downstream	62250	Hs.406917	QRSL1
rs10508931	10	52023961	0.1081	95.79%	0.337000	0.315400	0.04408000	0.15540000	NM_147156	intron	0	Hs.654698	SGMS1
rs6935311	6	134952731	0.3023	90.53%	0.767700	1.000000	0.04408000	0.02714000	NM_005627	upstream	415051	Hs.510078	SGK1
rs7699779	4	79736581	0.0060	88.07%	0.005988	1.000000	0.04410000	0.99870000	NM_005139	intron	0	Hs.480042	ANXA3
rs10509242	10	67348809	0.0502	97.89%	0.509800	0.529800	0.04412000	0.04094000	NM_013266	downstream	916	Hs.660362	CTNNA3
rs1960118	4	42378409	0.0502	97.89%	0.509800	0.529800	0.04412000	0.23190000	NM_006095	upstream	24750	Hs.435052	ATP8A1
rs61875	6	86912251	0.0502	97.89%	1.000000	1.000000	0.04412000	0.02272000	ENST00000257777	downstream	68433	---	---
rs9311896	3	64514815	0.0502	97.89%	1.000000	1.000000	0.04412000	0.03905000	NM_182920	intron	0	Hs.656071	ADAMTS9
rs10487933	7	6033769	0.2093	94.74%	0.460700	0.614600	0.04416000	0.00810200	NM_014413	intron	0	Hs.520205	EIF2AK1
rs2866382	4	102609326	0.0380	87.72%	1.000000	1.000000	0.04417000	0.05952000	NM_000944	upstream	121950	Hs.435512	PPP3CA
rs4131769	12	49238289	0.3098	96.84%	0.888400	1.000000	0.04417000	0.07126000	NM_173602	intron	0	Hs.505516	DIP2B
rs7083302	10	19162022	0.4951	71.93%	0.068940	0.172800	0.04417000	0.16300000	NM_178815	downstream	155076	Hs.25362	ARL5B
rs2466830	17	28226096	0.3668	90.88%	1.000000	0.388500	0.04421000	0.10370000	NM_015194	intron	0	Hs.658000	MYO1D
rs1538352	1	109106525	0.1032	88.42%	0.033470	0.076810	0.04422000	0.15460000	NM_007269	intron	0	Hs.530436	STXBP3
rs10495659	2	17167437	0.2072	92.28%	1.000000	0.603900	0.04423000	0.36770000	ENST00000388051	downstream	242	---	---
rs10508187	13	107191904	0.0511	96.14%	1.000000	1.000000	0.04424000	0.10430000	ENST00000375915	intron	0	---	---
rs1038062	8	4489894	0.2143	90.88%	0.196200	0.141800	0.04430000	0.13460000	ENST00000383934	downstream	483315	---	---
rs586284	9	27142977	0.2407	94.04%	0.503600	0.259100	0.04431000	0.05713000	NM_000459	intron	0	Hs.89640	TEK
rs7258628	19	35860761	0.0944	94.74%	0.071580	0.010100	0.04431000	0.29460000	NM_014717	downstream	119956	Hs.378901	ZNF536
rs934837	3	62688009	0.0566	89.82%	0.568400	0.244600	0.04431000	0.05325000	NM_183393	intron	0	Hs.654933	CADPS
rs10493406	1	66730967	0.1467	96.84%	0.811700	0.481500	0.04432000	0.03142000	NM_032291	upstream	41586	Hs.132121	SGIP1

rs724177	10	14691056	0.2608	97.54%	0.276500	0.100100	0.04432000	0.04944000	NM_031453	intron	0	Hs.446315	FAM107B
rs1375199	2	33674902	0.2011	95.09%	1.000000	0.829400	0.04433000	0.09814000	NM_015475	intron	0	Hs.468140	FAM98A
rs10494399	1	162081957	0.0415	97.19%	0.000481	0.121800	0.04435000	0.02057000	ENST00000385701	upstream	77574	---	---
rs7046513	9	104439252	0.2417	95.09%	0.319200	0.254400	0.04436000	0.05965000	ENST00000374800	intron	0	---	---
rs10487637	7	143932280	0.0505	97.19%	0.512400	0.532000	0.04438000	0.12930000	NM_022445	intron	0	Hs.660232	TPK1
rs10505445	8	125662775	0.4738	93.68%	0.539600	0.650900	0.04440000	0.02544000	NM_014751	intron	0	Hs.336994	MTSS1
rs10505850	12	19802620	0.3370	94.74%	0.223300	0.035180	0.04440000	0.04722000	NM_000921	upstream	610866	Hs.591150	PDE3A
rs2830399	21	26986248	0.1000	91.23%	0.029040	0.018520	0.04440000	0.13570000	NM_052954	upstream	118796	Hs.37445	CYYR1
rs10490538	2	191298311	0.2399	95.09%	0.738800	0.850200	0.04441000	0.04520000	NM_005966	downstream	32577	Hs.570078	NAB1
rs10518420	1	73633131	0.0420	96.14%	0.382200	1.000000	0.04441000	0.14210000	ENST00000388295	upstream	203003	---	---
rs3217824	12	4261593	0.0055	95.79%	0.005505	1.000000	0.04441000	0.99920000	NM_001759	intron	0	Hs.376071	CCND2
rs722359	6	117899571	0.1964	96.49%	0.568700	0.519700	0.04443000	0.02486000	NM_173674	upstream	10942	Hs.658304	DCBLD1
rs7719358	5	136366264	0.1667	87.37%	0.647000	1.000000	0.04443000	0.04740000	NM_004598	intron	0	Hs.654695	SPOCK1
rs555906	11	97458358	0.3528	92.98%	1.000000	0.729900	0.04444000	0.06236000	ENST00000384812	downstream	169259	---	---
rs10500142	7	144262265	0.1273	96.49%	0.784400	0.770400	0.04445000	0.08733000	ENST00000363803	upstream	84155	---	---
rs277886	16	25070005	0.3728	97.89%	0.608400	0.317700	0.04447000	0.00418500	NM_001032391	intron	0	Hs.337730	LCMT1
rs618701	1	63489839	0.4403	94.04%	0.804700	0.649700	0.04448000	0.04485000	NM_012183	upstream	71461	Hs.546573	FOXD3
rs10517333	4	34525359	0.1734	96.14%	0.406600	0.551300	0.04449000	0.09206000	NM_032457	downstream	3771790	Hs.570785	PCDH7
rs571361	1	81899114	0.4317	95.09%	0.387900	0.291000	0.04449000	0.25670000	ENST00000370721	intron	0	---	---
rs1258470	11	34685841	0.3700	97.19%	0.897500	0.876300	0.04452000	0.13940000	NM_012153	downstream	46184	Hs.653859	EHF
rs1565924	4	11120550	0.3822	96.84%	0.702300	0.870100	0.04453000	0.12660000	NM_005114	upstream	80063	Hs.507348	HS3ST1
rs10506295	12	49371738	0.3105	97.19%	0.778800	1.000000	0.04454000	0.04669000	NM_173602	intron	0	Hs.505516	DIP2B
rs1372469	4	46100211	0.0535	95.09%	1.000000	1.000000	0.04454000	0.00618300	NM_000807	upstream	13509	Hs.116250	GABRA2
rs9296984	6	15697285	0.0989	92.28%	0.296200	0.265100	0.04454000	0.13380000	NM_183041	intron	0	Hs.571148	DTNBP1
rs951954	2	109816794	0.0256	95.79%	1.000000	1.000000	0.04455000	0.01371000	NM_023016	downstream	82942	Hs.355455	ANKRD57
rs2027148	13	79302300	0.2000	85.96%	0.228600	0.495700	0.04457000	0.08820000	NM_019080	downstream	276078	Hs.525093	NDFIP2
rs10508760	10	31603466	0.0485	68.77%	0.000000	0.000000	0.04459000	0.29990000	NM_030751	upstream	44666	Hs.124503	ZEB1
rs7147624	14	64935378	0.1798	93.68%	0.836700	0.815000	0.04459000	0.03122000	NM_178157	upstream	13910	Hs.654961	FUT8
rs7336352	13	43013099	0.4607	93.68%	0.805300	0.760000	0.04459000	0.08604000	NM_017993	intron	0	Hs.128258	ENOX1
rs2349576	5	152618231	0.1142	93.68%	1.000000	1.000000	0.04460000	0.02008000	NM_000827	upstream	232268	Hs.519693	GRIA1
rs150951	14	69605628	0.0523	97.19%	0.538400	0.564300	0.04464000	0.03097000	NM_182936	intron	0	Hs.337696	SLC8A3
rs4799483	18	34403672	0.0523	97.19%	0.538400	0.564300	0.04464000	0.01754000	NM_020180	upstream	1003731	Hs.435976	BRUNOL4
rs2791790	6	102471860	0.0537	94.74%	0.548000	0.570900	0.04466000	0.00924000	NM_175768	intron	0	Hs.654523	GRIK2
rs9294097	6	78877951	0.4513	93.68%	0.387600	1.000000	0.04471000	0.05044000	NM_001010844	upstream	755957	Hs.656212	IRAK1BP1
rs10503432	8	12970627	0.0054	97.54%	1.000000	1.000000	0.04472000	0.99870000	NM_024767	downstream	145830	Hs.134296	DLC1
rs10504753	8	83410374	0.0054	97.54%	1.000000	1.000000	0.04472000	0.99880000	NM_152837	upstream	493384	Hs.492121	SNX16
rs1335310	10	2074135	0.4614	77.19%	0.001088	0.001555	0.04472000	0.03315000	NM_018702	upstream	304465	Hs.657984	ADARB2
rs352132	18	48085095	0.0569	89.47%	0.569900	0.591600	0.04473000	0.00635200	NM_005215	upstream	36061	Hs.579550	DCC
rs2246086	6	102472246	0.0520	97.89%	0.535700	0.562100	0.04474000	0.00826400	NM_175768	intron	0	Hs.654523	GRIK2
rs2791791	6	102472131	0.0520	97.89%	0.535700	0.562100	0.04474000	0.00826400	NM_175768	intron	0	Hs.654523	GRIK2
rs10485734	20	9573787	0.1942	97.54%	0.443500	0.420500	0.04475000	0.01577000	NM_177990	intron	0	Hs.32539	PAK7
rs10493184	1	56101529	0.1205	78.60%	0.749800	1.000000	0.04477000	0.02906000	NM_207465	downstream	131149	---	---
rs724858	4	6722860	0.4425	70.18%	0.000002	0.001621	0.04477000	0.08490000	NM_033296	downstream	27503	Hs.705528	MRFAP1
rs2038478	1	116536020	0.3027	91.58%	0.883700	0.567300	0.04479000	0.03816000	NM_152367	downstream	56636	Hs.376194	C1orf161
rs847331	14	71898828	0.1892	88.07%	0.147200	0.769800	0.04480000	0.06844000	NM_004296	intron	0	Hs.509872	RGS6
rs9284682	4	147294757	0.0167	73.33%	0.049750	1.000000	0.04480000	0.11900000	NM_007080	upstream	21574	Hs.190520	LSM6
rs10502904	18	45704096	0.0527	96.49%	0.030490	1.000000	0.04481000	0.16540000	ENST00000285039	intron	0	---	---
rs3117638	9	27168697	0.1000	91.23%	0.728600	1.000000	0.04485000	0.01731000	NM_000459	intron	0	Hs.89640	TEK
rs2197815	4	111786326	0.0636	96.49%	1.000000	1.000000	0.04487000	0.04570000	NM_000325	upstream	22623	Hs.643588	PITX2
rs998206	3	81804701	0.1201	89.12%	0.144200	0.372600	0.04487000	0.03050000	NM_000158	intron	0	Hs.436062	GBE1
rs1476946	9	112904570	0.3439	94.39%	0.225000	0.424300	0.04489000	0.42380000	NM_205859	downstream	225014	Hs.381312	OR2K2
rs10490697	2	105924977	0.1079	97.54%	0.340400	0.499200	0.04490000	0.01877000	NM_001004722	downstream	47840	Hs.705602	NCK2
rs1381100	20	58273271	0.2854	94.04%	0.368600	0.727800	0.04490000	0.04824000	NM_173644	downstream	191868	Hs.335319	C20orf197
rs2200014	8	79118949	0.4391	95.09%	0.387800	0.440400	0.04490000	0.11410000	NM_000318	upstream	1044042	Hs.437966	PXMP3

rs1477545	4	67736481	0.0796	94.74%	0.227800	0.501000	0.04491000	0.07785000	ENST00000383902	downstream	241424	---	---
rs1913419	4	12530692	0.3145	89.82%	0.309900	0.174700	0.04491000	0.14440000	ENST00000382444	upstream	413444	---	---
rs10494257	1	145721193	0.1462	97.19%	0.332700	0.289500	0.04493000	0.14420000	NM_181703	upstream	9127	Hs.447968	GJA5
rs10509960	10	114065482	0.1462	97.19%	0.811900	0.789300	0.04493000	0.28880000	NM_058222	downstream	12422	Hs.348615	TECTB
rs10511549	9	10538235	0.2398	94.39%	0.030240	0.166900	0.04494000	0.31530000	ENST00000363183	downstream	1105883	---	---
rs1871071	10	54669520	0.3358	95.09%	0.892200	0.601400	0.04495000	0.15120000	ENST00000387222	upstream	218434	---	---
rs9311976	3	7097638	0.3358	95.09%	0.222500	0.076290	0.04495000	0.12480000	NM_181874	intron	0	Hs.660131	GRM7
rs3767237	1	20972278	0.0755	97.54%	0.193900	0.173700	0.04497000	0.07072000	NM_016287	intron	0	Hs.142442	HP1BP3
rs4783361	16	22128305	0.1727	87.37%	0.376200	0.625300	0.04499000	0.05643000	NM_013302	intron	0	Hs.498892	EEF2K
rs10511297	3	112685490	0.1061	97.54%	0.527300	0.488600	0.04501000	0.05473000	NM_005816	upstream	58056	Hs.142023	CD96
rs1115558	7	68123432	0.1254	97.89%	0.591200	0.698300	0.04501000	0.02683000	ENST00000388535	upstream	41870	---	---
rs1414798	9	26799022	0.1254	97.89%	0.783000	1.000000	0.04501000	0.20350000	NM_024828	downstream	31661	Hs.178357	C9orf82
rs1829515	3	185087417	0.1697	97.19%	0.285200	0.239300	0.04502000	0.14240000	NM_001037639	upstream	2047	Hs.478469	PARL
rs10492673	13	87464048	0.3112	97.54%	0.050250	0.461000	0.04508000	0.08955000	NM_015567	downstream	334176	Hs.591208	SLITRK5
rs10517861	4	64167920	0.1911	90.88%	0.840700	0.653000	0.04508000	0.08627000	NM_001010874	downstream	660465	Hs.227752	SRD5A2L2
rs1963296	17	13110425	0.3112	97.54%	0.779900	1.000000	0.04508000	0.04962000	NM_006042	downstream	229306	Hs.462270	HS3ST3A1
rs2022212	6	69588678	0.3112	97.54%	0.888900	0.712400	0.04508000	0.04321000	NM_001704	intron	0	Hs.13261	BAI3
rs492681	18	9853662	0.1069	96.84%	1.000000	1.000000	0.04510000	0.23890000	NM_006868	downstream	1115	Hs.99528	RAB31
rs10500261	19	37719854	0.4943	91.93%	1.000000	0.355600	0.04511000	0.02496000	NM_004708	upstream	44091	Hs.443831	PDCD5
rs10496788	2	138225602	0.0646	95.09%	0.086990	0.082720	0.04512000	0.08030000	NM_001024075	upstream	212676	Hs.42151	HNMT
rs10491560	9	96884334	0.2000	96.49%	0.259000	0.392800	0.04514000	0.13340000	NM_032823	intron	0	Hs.434253	C9orf3
rs1349061	12	84753974	0.1338	94.39%	1.000000	0.578400	0.04515000	0.31080000	NM_005447	upstream	30101	Hs.527881	PAMCI
rs1501208	3	136386356	0.0356	93.68%	1.000000	1.000000	0.04515000	0.14450000	ENST00000360935	intron	0	---	---
rs10506068	12	30521940	0.1160	92.28%	0.762100	1.000000	0.04516000	0.06178000	NM_006390	downstream	151250	Hs.505136	IPO8
rs1869070	2	105982008	0.3426	94.74%	0.588600	1.000000	0.04518000	0.00585600	NM_032411	upstream	66537	Hs.43125	C2orf40
rs10482862	21	15245706	0.1211	89.82%	0.230900	0.339300	0.04520000	0.20730000	NM_003489	downstream	13202	Hs.155017	NRIP1
rs183436	21	42550900	0.3402	93.33%	0.784700	0.626200	0.04520000	0.36150000	NM_207630	intron	0	---	ABCG1
rs10503431	8	12970291	0.0054	97.19%	1.000000	1.000000	0.04521000	0.99870000	NM_024767	downstream	146166	Hs.134296	DLC1
rs10517532	4	61913826	0.0054	97.19%	1.000000	1.000000	0.04521000	0.99870000	ENST00000381217	downstream	61856	---	---
rs2417869	9	110430205	0.3407	94.74%	0.892300	0.379300	0.04524000	0.05888000	NM_006686	downstream	226487	Hs.534390	ACTL7B
rs1235012	8	4294524	0.0316	83.16%	1.000000	1.000000	0.04527000	0.06393000	ENST00000383934	downstream	678685	---	---
rs17045807	2	54385935	0.1680	88.77%	0.000000	0.000034	0.04528000	0.10590000	NM_138448	downstream	158	Hs.516173	ACYP2
rs10508374	10	9028079	0.0753	97.89%	0.380900	0.368500	0.04532000	0.12980000	NM_001013656	upstream	461012	---	LOC389936
rs10509872	10	110232026	0.0753	97.89%	1.000000	0.368500	0.04532000	0.14420000	ENST00000388288	downstream	458795	---	---
rs6578840	11	7245750	0.3810	88.42%	0.690300	0.488700	0.04533000	0.01541000	NM_175733	intron	0	Hs.177193	SYT9
rs10504857	8	89651741	0.1491	96.49%	0.344800	0.425100	0.04534000	0.06581000	NM_003821	upstream	1187432	Hs.103755	RIPK2
rs1392062	3	65671865	0.2671	97.19%	0.758600	0.591900	0.04534000	0.05547000	NM_001033057	intron	0	Hs.651939	MAGI1
rs665554	11	105339382	0.3593	92.28%	0.789300	0.391100	0.04534000	0.09031000	NM_000829	intron	0	Hs.503743	GRIA4
rs9315759	13	39616680	0.0769	95.79%	1.000000	1.000000	0.04534000	0.01289000	ENST00000384727	upstream	82284	---	---
rs10494635	1	188759982	0.1328	95.09%	0.594400	1.000000	0.04535000	0.00514200	NM_199051	upstream	46600	Hs.65765	FAM5C
rs7559023	2	40008348	0.1328	95.09%	0.439500	0.260600	0.04535000	0.10240000	NM_025264	upstream	148437	Hs.468254	THUMPD2
rs9313981	5	163193993	0.0414	97.54%	1.000000	1.000000	0.04535000	0.12390000	ENST00000385742	upstream	38244	---	---
rs10518389	4	122531523	0.3831	97.54%	0.527000	0.745900	0.04539000	0.05819000	NM_198179	upstream	10271	Hs.368977	GPR103
rs556585	1	232415025	0.1335	82.81%	1.000000	0.696800	0.04539000	0.06235000	NM_173508	intron	0	Hs.158748	SLC35F3
rs10519143	15	45842859	0.1703	80.35%	0.000010	0.000126	0.04540000	0.13340000	NM_024966	intron	0	Hs.511265	SEMA6D
rs10503123	18	64625503	0.3011	92.63%	0.559300	0.172600	0.04544000	0.05297000	NM_024781	intron	0	Hs.280781	CCDC102B
rs10514820	9	8005209	0.1585	92.98%	0.819200	0.522200	0.04548000	0.07050000	NM_033428	upstream	215142	Hs.7517	C9orf123
rs4652412	1	177778277	0.4244	95.09%	0.025010	0.290000	0.04548000	0.02412000	NM_182766	intron	0	Hs.658505	C1orf125
rs10499928	7	96222714	0.4407	94.74%	0.538000	0.755700	0.04549000	0.08115000	NM_006304	upstream	45575	Hs.489201	SHFM1
rs1961969	7	18060812	0.2119	94.39%	0.467500	0.099250	0.04551000	0.02682000	NM_175886	upstream	26885	Hs.169284	PRPS1L1
rs10506534	12	63662905	0.1470	97.89%	1.000000	1.000000	0.04552000	0.05532000	NM_007191	downstream	67769	Hs.284122	WIF1
rs661240	11	81554995	0.3147	97.54%	0.889900	0.618700	0.04552000	0.04465000	ENST00000357859	upstream	523254	---	---
rs4896783	6	125539447	0.0394	84.56%	0.043500	1.000000	0.04553000	0.05809000	NM_001003397	intron	0	Hs.591347	TPD52L1
rs1486897	3	114698508	0.1641	90.88%	0.173900	0.317100	0.04554000	0.01382000	NM_144718	intron	0	Hs.477144	CCDC52

rs10513530	3	160147221	0.2577	91.23%	0.000035	0.000021	0.04555000	0.04599000	NM_014575	upstream	327083	Hs.134665	SCHIP1
rs1067335	2	44479462	0.3457	94.39%	0.789200	0.602300	0.04557000	0.01945000	NM_024766	intron	0	Hs.468349	C2orf34
rs10486757	7	50661352	0.0799	94.39%	0.229500	0.132900	0.04558000	0.45230000	NM_001001555	intron	0	Hs.164060	GRB10
rs10520025	5	128486733	0.2653	97.19%	0.216800	0.472300	0.04559000	0.07075000	NM_016048	downstream	9121	Hs.483296	ISOC1
rs2508371	11	100773657	0.1475	97.54%	1.000000	1.000000	0.04559000	0.00417300	NM_004621	downstream	53920	Hs.159003	TRPC6
rs4954657	2	139878418	0.1315	94.74%	0.791300	1.000000	0.04559000	0.13820000	ENST00000384174	upstream	107046	---	---
rs10484903	6	108467752	0.0054	97.89%	1.000000	1.000000	0.04560000	0.99900000	NM_014028	downstream	1554	Hs.226780	OSTM1
rs10494905	1	206988952	0.0054	97.89%	1.000000	1.000000	0.04560000	0.99870000	NM_025179	upstream	504664	Hs.497626	PLXNA2
rs10498506	14	62154183	0.0054	97.89%	1.000000	1.000000	0.04560000	0.99900000	NM_172375	downstream	88869	Hs.27043	KCNH5
rs10499591	7	36524263	0.0054	97.89%	1.000000	1.000000	0.04560000	0.99870000	NM_001637	intron	0	Hs.488007	AOAH
rs10500487	16	62617865	0.0054	97.89%	0.005386	1.000000	0.04560000	0.99860000	NM_001797	downstream	920321	Hs.116471	CDH11
rs10504750	8	83361452	0.0054	97.89%	1.000000	1.000000	0.04560000	0.99880000	NM_152837	upstream	444462	Hs.492121	SNX16
rs1393230	5	9677151	0.0054	97.89%	1.000000	1.000000	0.04560000	0.99870000	NM_019599	downstream	4964	Hs.567492	TAS2R1
rs2361493	6	110477273	0.0054	97.89%	1.000000	1.000000	0.04560000	0.99890000	NM_001024936	downstream	51109	Hs.75850	WASF1
rs3886641	7	30634762	0.0054	97.89%	1.000000	1.000000	0.04560000	0.99880000	NM_002047	CDS	0	Hs.404321	GARS
rs7120189	11	28648363	0.0054	97.89%	1.000000	1.000000	0.04560000	0.99880000	NM_152636	downstream	336844	Hs.243326	METT5D1
rs7576	10	85902586	0.1828	94.04%	0.540800	1.000000	0.04560000	0.04304000	NM_014394	3UTR	0	Hs.352656	GHITM
rs1851243	3	118236015	0.3891	96.49%	1.000000	0.438700	0.04561000	0.02865000	ENST00000363502	upstream	69252	---	---
rs10504289	8	60315628	0.2284	97.54%	0.865200	0.845600	0.04562000	0.02351000	ENST00000384126	upstream	103014	---	---
rs10507441	13	36231087	0.0551	92.28%	0.558000	0.577700	0.04563000	0.11070000	NM_203451	downstream	61446	Hs.422375	LOC400120
rs1257409	14	98681324	0.0132	92.98%	1.000000	1.000000	0.04563000	0.09418000	NM_022898	downstream	24053	Hs.699440	BCL11B
rs10506433	12	60733039	0.1048	87.02%	0.161600	0.014480	0.04566000	0.04238000	NM_178539	upstream	29210	Hs.269745	FAM19A2
rs9319349	13	18801580	0.4136	95.44%	0.453000	0.093570	0.04566000	0.02780000	ENST00000305173	upstream	52930	---	---
rs10485057	6	154454948	0.0772	95.44%	0.061360	0.180300	0.04567000	0.14660000	NM_001008505	intron	0	Hs.2353	OPRM1
rs2060915	4	40956257	0.1573	93.68%	0.490800	0.301800	0.04567000	0.06878000	NM_004181	intron	0	Hs.518731	UCHL1
rs10520817	5	15612551	0.0694	88.42%	0.103500	0.330500	0.04568000	0.15380000	NM_012304	upstream	56540	Hs.433057	FBXL7
rs6424623	1	79258910	0.0799	94.39%	0.679200	0.117000	0.04568000	0.02035000	ENST00000370739	upstream	13960	---	---
rs1327559	10	97158130	0.0354	94.04%	0.281400	1.000000	0.04569000	0.04902000	NM_001034957	intron	0	Hs.696027	SORBS1
rs10505829	12	18731968	0.0351	95.09%	1.000000	1.000000	0.04571000	0.04228000	NM_033123	intron	0	Hs.97542	PLCZ1
rs2272433	6	139293010	0.0351	95.09%	1.000000	1.000000	0.04571000	0.04373000	NM_031922	intron	0	Hs.334603	REPS1
rs1834160	18	49560946	0.0771	97.89%	1.000000	1.000000	0.04573000	0.10040000	NM_005215	downstream	249925	Hs.579550	DCC
rs7325791	13	103774402	0.0094	92.98%	0.018850	1.000000	0.04573000	0.22700000	NM_172370	upstream	1142191	Hs.381382	DAOA
rs690600	15	51796631	0.2670	97.89%	0.759800	0.472300	0.04576000	0.00809800	NM_182758	intron	0	Hs.122125	WDR72
rs725421	12	86220791	0.2274	97.19%	1.000000	0.692700	0.04580000	0.11090000	NM_013244	upstream	1253145	Hs.662231	MGAT4C
rs9312239	4	180199353	0.4226	79.30%	0.000071	0.024230	0.04580000	0.10270000	ENST00000364857	downstream	634395	---	---
rs1916184	8	47842969	0.1515	84.56%	0.010110	0.009701	0.04581000	0.07206000	ENST00000388124	downstream	15714	---	---
rs996655	12	98858278	0.3835	97.89%	0.130100	0.250400	0.04582000	0.20590000	NM_152788	intron	0	Hs.506458	ANKS1B
rs10510871	3	62065420	0.0773	97.54%	0.386500	1.000000	0.04584000	0.02384000	NM_002841	intron	0	Hs.654488	PTPRG
rs2383183	9	21177700	0.0539	94.39%	0.549400	0.568700	0.04584000	0.01922000	NM_021068	upstream	30	Hs.1510	IFNA4
rs6823638	4	20857093	0.2016	87.02%	0.553400	0.415900	0.04584000	0.19950000	NM_001035004	intron	0	Hs.655705	KCNIP4
rs1917213	7	42544397	0.0578	88.07%	1.000000	1.000000	0.04585000	0.20210000	NM_000168	upstream	314977	Hs.21509	GLI3
rs1358554	4	92512505	0.3513	94.39%	0.503200	0.523600	0.04588000	0.02794000	NM_207491	intron	0	Hs.654735	MGC48628
rs2640629	12	56351715	0.3413	95.09%	0.080590	0.037290	0.04589000	0.26840000	NM_001017958	upstream	22497	Hs.527861	OS9
rs2631271	4	103133419	0.1617	93.33%	0.364000	0.337700	0.04591000	0.09787000	NM_017935	intron	0	Hs.480400	BANK1
rs1351649	11	7703698	0.3370	94.74%	0.275200	0.418200	0.04593000	0.18090000	NM_198185	upstream	19181	Hs.532475	OVCH2
rs1519320	4	187177130	0.2154	93.68%	0.018650	0.212100	0.04593000	0.19940000	ENST00000363627	downstream	7479	---	---
rs6880876	5	28317298	0.2729	95.79%	0.092860	0.298900	0.04593000	0.21190000	ENST00000387488	upstream	343223	---	---
rs2350786	7	136327110	0.4375	87.02%	0.367800	0.417000	0.04594000	0.15000000	NM_001006629	intron	0	Hs.535891	CHRM2
rs2909958	5	112195840	0.3242	95.79%	0.268400	0.510300	0.04594000	0.23460000	NM_000038	intron	0	Hs.158932	APC
rs10511548	9	10504108	0.1854	84.21%	0.674400	1.000000	0.04597000	0.17080000	ENST00000363183	downstream	1071756	---	---
rs10493499	1	72728578	0.2103	95.09%	0.855200	0.836300	0.04598000	0.04460000	NM_173808	upstream	207573	Hs.146542	NEGR1
rs1919224	4	92505468	0.4249	95.79%	0.385900	0.430600	0.04598000	0.18810000	NM_207491	intron	0	Hs.654735	MGC48628
rs4545290	1	72728411	0.2103	95.09%	0.855200	0.836300	0.04598000	0.04460000	NM_173808	upstream	207406	Hs.146542	NEGR1
rs3738516	1	43212788	0.1984	88.42%	1.000000	0.822800	0.04599000	0.09189000	NM_006516	upstream	15700	Hs.473721	SLC2A1

rs2249862	21	21528981	0.4721	94.39%	0.328900	0.762100	0.04600000	0.02173000	NM_004540	upstream	45786	Hs.473450	NCAM2
rs10486245	7	8676842	0.0352	94.74%	1.000000	1.000000	0.04601000	0.03832000	NM_152745	upstream	80321	Hs.487564	NXPH1
rs2048178	2	41414979	0.2489	83.16%	0.014280	0.420700	0.04602000	0.02679000	NM_021097	upstream	880791	Hs.468274	SLC8A1
rs1016315	10	131742295	0.1152	94.39%	0.368800	0.535100	0.04603000	0.16140000	NM_001005463	upstream	90200	Hs.699395	EBF3
rs2880170	4	60264032	0.2319	96.84%	0.400300	1.000000	0.04603000	0.01984000	ENST00000364806	upstream	117456	---	---
rs2095447	6	84169475	0.4944	93.33%	0.902400	0.444300	0.04605000	0.05139000	NM_002395	intron	0	Hs.21160	ME1
rs10516658	4	82899808	0.0627	97.89%	0.610600	0.604300	0.04606000	0.15230000	NM_152545	upstream	287723	Hs.591696	RASGEF1B
rs10516659	4	82899710	0.0627	97.89%	0.610600	0.604300	0.04606000	0.23690000	NM_152545	upstream	287625	Hs.591696	RASGEF1B
rs1996553	15	68011638	0.2652	97.89%	0.538400	0.148900	0.04607000	0.16250000	NM_005078	downstream	117835	Hs.287362	TLE3
rs10485363	6	13245751	0.1592	93.68%	0.356600	0.611000	0.04608000	0.03191000	NM_030948	intron	0	Hs.436996	PHACTR1
rs1475398	1	65755845	0.3487	95.09%	0.893700	1.000000	0.04609000	0.11900000	NM_001003680	intron	0	Hs.705413	LEPR
rs4466461	9	120353919	0.4244	95.09%	0.046420	0.285100	0.04609000	0.03594000	NM_014618	downstream	614810	Hs.532316	DBC1
rs10483358	14	30456745	0.0054	97.54%	1.000000	1.000000	0.04610000	0.99880000	NM_014574	intron	0	Hs.401843	STRN3
rs10505525	8	129519439	0.0054	97.54%	0.005405	1.000000	0.04610000	0.99870000	ENST00000364912	downstream	217180	---	---
rs10518067	4	70694528	0.0054	97.54%	1.000000	1.000000	0.04610000	0.99870000	NM_014465	upstream	33509	Hs.129742	SULT1B1
rs2964302	5	165911438	0.1964	88.42%	1.000000	0.655500	0.04610000	0.03567000	ENST00000320147	downstream	169251	---	---
rs10502607	18	28562048	0.2973	92.63%	0.184600	0.489300	0.04613000	0.23120000	NM_020805	intron	0	Hs.446164	KLHL14
rs346510	4	87055405	0.3799	97.89%	0.030460	0.323600	0.04615000	0.21260000	NM_001025616	intron	0	Hs.444229	ARHGAP24
rs10516583	4	113244713	0.1347	95.09%	1.000000	0.702300	0.04619000	0.15700000	NM_152400	upstream	41414	Hs.23439	C4orf32
rs1504795	8	91935906	0.4532	93.68%	0.083770	0.094990	0.04619000	0.13640000	NM_022351	intron	0	Hs.560892	NECAB1
rs6461856	7	3133925	0.1727	97.54%	0.020330	0.074500	0.04619000	0.03711000	NM_032415	upstream	83900	Hs.648101	CARD11
rs1011844	8	83545626	0.1872	79.65%	0.662400	1.000000	0.04620000	0.03989000	NM_152837	upstream	628636	Hs.492121	SNX16
rs10498074	2	220842523	0.0568	92.63%	0.195800	0.209500	0.04620000	0.05971000	ENST00000355668	upstream	568571	---	---
rs10503629	8	18601009	0.0055	96.49%	1.000000	1.000000	0.04620000	0.99870000	NM_206909	intron	0	Hs.434255	PSD3
rs10496986	2	146210691	0.4449	85.96%	0.156100	0.038590	0.04623000	0.09793000	NM_014795	upstream	1216305	Hs.34871	ZEB2
rs757918	2	101979731	0.4064	88.07%	0.695400	0.751000	0.04623000	0.05969000	NM_173343	intron	0	Hs.25333	IL1R2
rs7816623	8	105733450	0.2239	90.88%	0.475300	0.141200	0.04623000	0.04385000	NM_013437	upstream	63123	Hs.654804	LRP12
rs313265	2	125818067	0.1580	94.39%	0.820900	0.610200	0.04624000	0.14640000	NM_016815	upstream	1346477	Hs.59138	GYPC
rs2040644	12	46021747	0.0558	94.39%	0.575100	0.598000	0.04625000	0.07430000	NM_138371	downstream	105041	Hs.560100	FAM113B
rs10491865	9	1312136	0.2717	96.84%	0.447000	0.838300	0.04626000	0.15240000	NM_181872	downstream	264584	Hs.59506	DMRT2
rs2026978	9	31605043	0.4722	94.74%	1.000000	0.760900	0.04626000	0.08861000	ENST00000360120	downstream	360785	---	---
rs2331107	5	33004477	0.0556	94.74%	1.000000	1.000000	0.04626000	0.06060000	NM_024563	downstream	176901	Hs.13528	C5orf23
rs1002508	12	129318585	0.0554	95.09%	0.572300	0.595800	0.04627000	0.02035000	NM_007197	downstream	102348	Hs.31664	FZD10
rs4449188	2	99915769	0.0573	91.93%	0.041850	0.045990	0.04627000	0.20900000	NM_001025108	intron	0	Hs.444414	AFF3
rs881958	6	156774970	0.0988	90.53%	0.084250	1.000000	0.04627000	0.14390000	ENST00000385709	downstream	33323	---	---
rs10512902	5	6956471	0.3545	77.19%	0.076240	0.160100	0.04630000	0.06608000	ENST00000363373	upstream	54896	---	---
rs2241992	2	106765797	0.1574	94.74%	0.820300	0.455200	0.04632000	0.06776000	NM_032528	downstream	18695	Hs.98265	ST6GAL2
rs6140677	20	8597582	0.0664	95.09%	0.327600	1.000000	0.04632000	0.03352000	NM_182734	intron	0	Hs.431173	PLCB1
rs207295	4	18710314	0.0266	92.28%	1.000000	1.000000	0.04633000	0.01330000	ENST00000360734	upstream	16568	---	---
rs10499207	6	139328550	0.0353	94.39%	1.000000	1.000000	0.04634000	0.04794000	NM_031922	intron	0	Hs.334603	REPS1
rs10504470	8	71253000	0.1561	94.39%	0.352400	0.606400	0.04636000	0.04182000	NM_006540	intron	0	Hs.446678	NCOA2
rs705197	1	34617423	0.1673	89.12%	0.039310	0.747100	0.04636000	0.12330000	NM_032884	downstream	160104	Hs.194610	C1orf94
rs10501182	11	37605068	0.1127	85.61%	0.203800	0.493500	0.04638000	0.02493000	NM_138787	downstream	967671	Hs.406726	C11orf74
rs4113916	5	130089917	0.1573	93.68%	0.252000	0.203700	0.04638000	0.11650000	NM_005340	downstream	432966	Hs.483305	HINT1
rs10509130	10	61791118	0.2547	92.98%	0.626800	0.576000	0.04641000	0.04795000	NM_020987	intron	0	Hs.499725	ANK3
rs10503352	8	5939750	0.0643	95.44%	1.000000	1.000000	0.04644000	0.10540000	NM_024596	upstream	311780	Hs.656769	MCPH1
rs1394605	5	155692365	0.0643	95.44%	1.000000	1.000000	0.04644000	0.05024000	NM_172244	intron	0	Hs.591727	SGCD
rs354877	4	68150615	0.1268	96.84%	0.410500	1.000000	0.04645000	0.07982000	NM_012108	intron	0	Hs.435579	STAP1
rs1220849	4	70849631	0.4778	94.74%	0.544600	0.363900	0.04646000	0.10170000	NM_001025104	downstream	2754	Hs.3155	CSN1S1
rs949586	1	37555489	0.4778	94.74%	0.807200	0.762200	0.04646000	0.01778000	NM_025079	upstream	157251	Hs.656294	ZC3H12A
rs25999	5	106832906	0.3058	91.23%	0.188100	0.862800	0.04651000	0.06632000	NM_001962	intron	0	Hs.658451	EFNA5
rs2106092	1	157099068	0.1851	91.93%	1.000000	0.256900	0.04652000	0.04220000	NM_002432	downstream	13150	Hs.153837	MNDA
rs1122080	5	157948481	0.2018	96.49%	0.023530	0.009119	0.04653000	0.13070000	NM_024007	downstream	110216	Hs.657753	EBF1
rs6840951	4	14053172	0.1863	92.28%	0.226900	0.384600	0.04657000	0.03355000	NM_148894	upstream	814727	Hs.444517	FAM44A

rs9297775	8	129805894	0.2907	94.74%	0.554800	1.000000	0.04657000	0.03445000	ENST00000384930	downstream	95328	---	---
rs1280099	4	187775324	0.4888	94.39%	0.272100	0.128700	0.04658000	0.13850000	NM_005245	CDS	0	Hs.481371	FAT
rs2346442	7	133356447	0.3219	97.54%	0.582600	0.869700	0.04658000	0.14220000	NM_001037126	intron	0	Hs.321273	EXOC4
rs10490900	10	29609281	0.0060	88.42%	1.000000	1.000000	0.04659000	0.99870000	NM_032517	upstream	8715	Hs.558572	LYZL1
rs10512175	9	88142133	0.0541	94.04%	0.550900	0.568700	0.04659000	0.12710000	NM_024617	intron	0	Hs.655162	ZCCHC6
rs10484822	6	139973840	0.0054	97.19%	1.000000	1.000000	0.04660000	0.99870000	NM_006079	upstream	236362	Hs.82071	CITED2
rs355686	4	78724396	0.1892	90.88%	1.000000	0.397400	0.04660000	0.01152000	NM_006419	upstream	21602	Hs.100431	CXCL13
rs2243737	20	50943913	0.3895	90.53%	0.795000	0.178700	0.04661000	0.00144000	NM_173485	upstream	78440	Hs.473117	TSHZ2
rs313877	11	102830195	0.1325	94.04%	0.281000	0.135900	0.04661000	0.05094000	ENST00000334267	intron	0	---	---
rs2526471	17	20092265	0.3383	94.39%	0.497600	0.048860	0.04662000	0.06597000	NM_001033553	intron	0	Hs.431045	SPECC1
rs2097229	7	141736663	0.3521	93.68%	0.043560	0.300200	0.04663000	0.13710000	ENST00000356506	intron	0	---	---
rs1113765	7	55856828	0.2932	93.33%	0.767400	0.727700	0.04664000	0.01068000	NM_207366	intron	0	Hs.453629	14.Sep
rs10488628	7	128155128	0.4833	94.74%	0.028900	0.009583	0.04665000	0.10200000	NM_032599	intron	0	Hs.131098	FAM71F1
rs10518077	4	71252668	0.0535	95.09%	1.000000	1.000000	0.04666000	0.16660000	NM_012390	upstream	8414	Hs.701334	SMR3A
rs925986	2	151321815	0.0535	95.09%	0.546600	1.000000	0.04666000	0.04435000	NM_005168	upstream	269389	Hs.6838	RND3
rs10488625	7	135928298	0.2595	92.63%	0.633500	0.267900	0.04667000	0.03341000	NM_001006629	upstream	275658	Hs.535891	CHRM2
rs998247	14	64616052	0.2595	92.63%	0.748400	0.853200	0.04667000	0.05924000	NM_197957	intron	0	Hs.285354	MAX
rs723364	4	111943920	0.1989	97.89%	0.134800	0.793000	0.04668000	0.03156000	NM_000325	upstream	180217	Hs.643588	PITX2
rs250141	16	78218998	0.4094	96.84%	0.061550	0.451600	0.04669000	0.04995000	NM_001031804	upstream	26886	Hs.134859	MAF
rs1053054	8	75056579	0.2767	88.77%	1.000000	0.366000	0.04670000	0.05029000	NM_001040613	3UTR	0	Hs.106650	TMEM70
rs11066209	12	111161662	0.1733	97.19%	0.527300	1.000000	0.04670000	0.05331000	ENST00000377563	intron	0	---	---
rs10500329	16	5834783	0.4779	95.09%	0.330600	0.450800	0.04672000	0.08661000	NM_001013705	upstream	532982	---	LOC440337
rs9307376	4	113089585	0.3917	89.12%	0.293600	0.864900	0.04675000	0.06906000	NM_152400	upstream	196542	Hs.23439	C4orf32
rs4364933	1	200942166	0.2815	89.12%	0.437200	0.475200	0.04680000	0.05878000	NM_177402	intron	0	Hs.585015	SYT2
rs2422207	1	170914146	0.2771	81.05%	0.870900	0.255600	0.04683000	0.00683900	NM_000639	downstream	11509	Hs.2007	FASLG
rs1943788	18	69169964	0.4164	96.49%	0.081860	0.208400	0.04685000	0.20370000	NM_138966	upstream	486050	Hs.465407	NETO1
rs3736585	10	124732576	0.4164	96.49%	0.136000	0.345500	0.04685000	0.03576000	NM_153336	intron	0	Hs.281004	PSTK
rs4775711	15	45859424	0.4244	95.09%	0.709900	0.645500	0.04685000	0.11370000	NM_024966	downstream	9388	Hs.511265	SEMA6D
rs7084992	10	58087356	0.2036	96.49%	0.060950	0.087910	0.04685000	0.33750000	NM_001005414	upstream	296323	---	ZWINT
rs4520564	11	100808606	0.0443	95.09%	0.412900	1.000000	0.04686000	0.01564000	NM_004621	downstream	18971	Hs.159003	TRPC6
rs10517830	4	63388819	0.0677	93.33%	0.617000	0.610900	0.04687000	0.02472000	NM_001010874	downstream	1439566	Hs.227752	SRD5A2L2
rs2135615	4	178551536	0.2399	95.79%	0.318000	0.171300	0.04688000	0.04539000	NM_018248	downstream	30542	Hs.405467	NEIL3
rs9321445	6	134794917	0.2100	94.39%	0.712800	0.452600	0.04688000	0.02554000	NM_005627	upstream	257237	Hs.510078	SGK1
rs3786335	18	59712501	0.3774	90.18%	1.000000	0.394300	0.04692000	0.07913000	NM_002575	intron	0	Hs.594481	SERPINB2
rs3845680	2	25952517	0.4328	94.04%	0.215400	1.000000	0.04695000	0.00984800	NM_018263	intron	0	Hs.700650	ASXL2
rs10485835	7	141093287	0.3520	68.77%	0.018480	0.063180	0.04696000	0.04302000	NM_003143	intron	0	Hs.490394	SSBP1
rs443537	3	8797352	0.2126	75.09%	0.006897	0.020240	0.04696000	0.04160000	NM_000916	upstream	11052	Hs.2820	OXTR
rs6133598	20	8597730	0.0566	92.98%	0.580900	1.000000	0.04696000	0.02698000	NM_182734	intron	0	Hs.431173	PLCB1
rs10514260	5	83007677	0.4588	93.68%	1.000000	0.878400	0.04697000	0.04488000	NM_001884	intron	0	Hs.591758	HAPLN1
rs215702	7	32366183	0.3030	92.63%	0.465800	0.442100	0.04698000	0.07124000	NM_005020	upstream	288667	Hs.655694	PDE1C
rs2385525	8	50768163	0.1357	94.39%	0.437200	1.000000	0.04698000	0.05407000	NM_018967	upstream	701163	Hs.584914	SNTG1
rs598810	7	42940865	0.1357	94.39%	0.117600	0.470200	0.04698000	0.03812000	NM_031903	intron	0	Hs.50252	MRPL32
rs1863246	12	9410439	0.0094	93.33%	1.000000	1.000000	0.04700000	0.11600000	ENST00000334227	downstream	31061	---	---
rs10521198	17	12550196	0.0917	97.54%	0.264700	1.000000	0.04703000	0.14810000	NM_153604	intron	0	Hs.567641	MYOCD
rs10500910	11	21561727	0.0667	94.74%	1.000000	0.365500	0.04704000	0.05725000	NM_006157	downstream	7924	Hs.657172	NELL1
rs767278	3	64954723	0.0667	94.74%	0.329300	1.000000	0.04704000	0.00635000	NM_182920	upstream	306318	Hs.656071	ADAMTS9
rs851408	2	19307216	0.1286	96.84%	0.787900	0.553900	0.04704000	0.23630000	NM_145260	downstream	107511	Hs.123933	OSR1
rs1358987	11	35998190	0.0812	95.09%	0.233300	0.382100	0.04710000	0.02287000	NM_174902	intron	0	Hs.700909	LDLRAD3
rs10484708	6	56209427	0.0787	82.46%	1.000000	1.000000	0.04711000	0.05167000	NM_030820	intron	0	Hs.47629	COL21A1
rs2369955	3	134176278	0.1610	93.68%	1.000000	0.329200	0.04711000	0.04176000	NM_023943	upstream	63658	Hs.191616	TMEM108
rs2823503	21	16124683	0.1610	93.68%	0.822400	0.206100	0.04711000	0.01955000	NM_013396	intron	0	Hs.473370	USP25
rs2647468	1	218235256	0.2537	94.04%	0.749300	0.577000	0.04712000	0.09284000	NM_004446	intron	0	Hs.497788	EPRS
rs840611	2	187906691	0.2996	93.68%	0.019940	0.039770	0.04712000	0.03065000	NM_005795	downstream	11386	Hs.470882	CALCRL
rs7679666	4	84925488	0.4478	94.04%	1.000000	0.875500	0.04714000	0.00827100	ENST00000364763	upstream	149015	---	---

rs10496660	2	127629514	0.3989	97.19%	0.045980	0.015070	0.04715000	0.12570000	NM_001001665	downstream	30948	Hs.407639	CYP27C1
rs542610	13	27133069	0.2070	95.79%	0.459300	1.000000	0.04715000	0.27730000	NM_152705	intron	0	Hs.507584	POLR1D
rs10509002	10	55338443	0.0746	87.02%	0.000077	0.000541	0.04717000	0.14680000	NM_033056	intron	0	Hs.672170	PCDH15
rs8076457	17	8884654	0.2014	97.54%	1.000000	0.205800	0.04717000	0.00479400	NM_004822	intron	0	Hs.660885	NTN1
rs10510190	3	2393162	0.0896	88.07%	0.120400	0.193700	0.04718000	0.09054000	NM_175613	upstream	194929	Hs.298705	CNTN4
rs10520854	5	18025013	0.2454	95.09%	0.190400	0.185700	0.04719000	0.03119000	ENST00000332101	upstream	336617	---	---
rs1430234	2	221931940	0.3052	93.68%	0.313200	0.258400	0.04720000	0.04881000	NM_004438	downstream	59053	Hs.371218	EPHA4
rs1819633	6	141354060	0.4625	93.68%	0.221300	0.273600	0.04720000	0.25550000	NM_006079	upstream	1616582	Hs.82071	CITED2
rs2247034	13	77849881	0.2706	97.89%	0.879600	0.211000	0.04722000	0.06423000	NM_006237	downstream	221347	Hs.654522	POU4F1
rs1498841	3	69663376	0.2556	93.33%	0.146800	0.026220	0.04723000	0.06085000	NM_198178	upstream	232276	Hs.166017	MITF
rs9283716	5	56022507	0.0055	95.79%	0.005505	1.000000	0.04723000	0.99870000	ENST00000381279	upstream	84691	---	---
rs2135760	6	54740495	0.3861	83.16%	0.001568	0.001615	0.04727000	0.06081000	NM_014464	downstream	377625	Hs.127011	TINAG
rs1383102	12	74303228	0.1593	94.74%	0.647700	1.000000	0.04730000	0.03208000	NM_007043	upstream	111569	Hs.645517	KRR1
rs6916797	6	51774782	0.2973	92.63%	0.557200	0.556200	0.04730000	0.04212000	NM_170724	intron	0	Hs.662050	PKHD1
rs1972855	3	61077762	0.3267	97.19%	0.341500	0.513000	0.04732000	0.03143000	NM_002841	upstream	445240	Hs.654488	PTPRG
rs10499053	6	110047191	0.3303	97.19%	0.787200	0.747600	0.04733000	0.03312000	ENST00000368948	intron	0	---	---
rs571693	9	14100242	0.2040	97.19%	0.853500	1.000000	0.04734000	0.11140000	NM_005596	intron	0	Hs.644095	NFIB
rs1073599	7	22855042	0.3074	90.18%	0.018770	0.017430	0.04736000	0.02706000	NM_019059	upstream	26096	Hs.112318	TOMM7
rs9302260	15	69961665	0.3304	80.70%	0.139300	0.560200	0.04736000	0.00910600	NM_006901	intron	0	Hs.546268	MYO9A
rs2790070	1	193811183	0.1393	91.93%	0.304700	0.705900	0.04737000	0.04727000	NM_198503	downstream	650353	Hs.657046	KCNT2
rs9325124	5	148229011	0.4029	97.54%	0.215400	0.872900	0.04737000	0.05172000	NM_000024	downstream	40564	Hs.591251	ADRB2
rs10511424	3	125711810	0.0667	94.74%	1.000000	1.000000	0.04738000	0.04923000	NM_003947	intron	0	Hs.8004	KALRN
rs2393150	10	58350942	0.0728	89.12%	0.628500	1.000000	0.04741000	0.11060000	NM_001005414	upstream	559902	---	ZWINT
rs10490161	2	51160894	0.1550	84.91%	0.136400	0.152900	0.04743000	0.01805000	ENST00000378257	upstream	51985	---	---
rs10486430	7	24710178	0.0444	94.74%	1.000000	1.000000	0.04744000	0.06855000	NM_004403	intron	0	Hs.520708	DFNA5
rs1399428	9	119700389	0.3309	95.44%	0.339600	0.098360	0.04744000	0.01716000	NM_138554	downstream	181419	Hs.174312	TLR4
rs10504382	8	66158149	0.1738	97.89%	0.143800	0.349500	0.04747000	0.06174000	NM_004820	upstream	284247	Hs.667720	CYP7B1
rs3005894	1	56557350	0.1751	97.19%	0.062530	0.351600	0.04747000	0.07632000	NM_177414	downstream	175177	Hs.405156	PPAP2B
rs2614059	8	28212815	0.1162	95.09%	0.145900	1.000000	0.04751000	0.43710000	NM_006228	upstream	17753	Hs.88218	PNOC
rs6734835	2	228923891	0.0695	93.33%	0.119600	1.000000	0.04752000	0.03780000	NM_030623	upstream	169305	Hs.436306	SPHKAP
rs10497940	2	211685573	0.2444	94.04%	1.000000	1.000000	0.04754000	0.09109000	NM_001042599	downstream	271012	Hs.390729	ERBB4
rs2104527	10	120684972	0.1727	97.54%	1.000000	1.000000	0.04757000	0.24890000	NM_001009553	upstream	94246	---	NANOS1
rs10483984	14	85223780	0.2802	95.79%	0.229800	0.313800	0.04759000	0.34340000	NM_013231	downstream	59758	Hs.533710	FLRT2
rs10512557	17	66450255	0.2183	94.04%	0.595300	0.683400	0.04761000	0.05179000	NM_000891	downstream	762500	Hs.1547	KCNJ2
rs7962399	12	113228631	0.3087	92.63%	0.013750	0.173400	0.04761000	0.04067000	NM_181486	downstream	47488	Hs.381715	TBX5
rs10492448	13	74546514	0.0093	94.04%	1.000000	1.000000	0.04762000	0.02641000	ENST00000384085	upstream	35211	---	---
rs4129191	2	44059455	0.4777	86.67%	0.074330	0.108600	0.04762000	0.06497000	NM_133259	intron	0	Hs.368084	LRPPRC
rs10491299	5	136367514	0.1125	95.09%	0.758200	0.742200	0.04763000	0.08488000	NM_004598	intron	0	Hs.654695	SPOCK1
rs10505035	8	103844351	0.3694	94.04%	0.599700	0.755200	0.04763000	0.01564000	NM_207505	upstream	43875	---	FLJ45248
rs1950703	14	32116139	0.1125	95.09%	0.354700	0.191700	0.04763000	0.05004000	NM_004274	CDS	0	Hs.509083	AKAP6
rs9320807	6	121694486	0.4094	96.84%	0.619800	1.000000	0.04763000	0.02210000	NM_152730	intron	0	Hs.121396	C6orf170
rs1037291	5	3097895	0.1845	95.09%	0.690300	0.781000	0.04767000	0.02483000	NM_178569	downstream	289384	Hs.668017	C5orf38
rs260818	11	103425315	0.3619	94.04%	0.792700	0.735500	0.04767000	0.24320000	NM_033135	intron	0	Hs.352298	PDGFD
rs6992591	8	116437619	0.1760	87.72%	0.186900	0.810400	0.04768000	0.02899000	NM_014112	downstream	56352	Hs.657018	TRPS1
rs7813351	8	2940819	0.4032	97.89%	0.456100	0.634100	0.04769000	0.16500000	NM_033225	intron	0	Hs.571466	CSMD1
rs10489269	1	171426686	0.0685	94.74%	1.000000	1.000000	0.04770000	0.19650000	NM_003326	intron	0	Hs.181097	TNFSF4
rs720092	11	22434196	0.2356	97.54%	0.867800	0.822700	0.04770000	0.05906000	NM_020346	downstream	76577	Hs.242821	SLC17A6
rs199241	6	143384561	0.1295	97.54%	0.791500	1.000000	0.04772000	0.07415000	NM_016108	upstream	39145	Hs.567501	AIG1
rs2823976	21	16979504	0.2500	94.74%	1.000000	0.707100	0.04773000	0.12320000	NM_001005734	downstream	78091	Hs.473394	C21orf34
rs4605250	18	36919202	0.1300	97.19%	0.281200	0.136900	0.04773000	0.27250000	NM_002647	upstream	869995	Hs.464971	PIK3C3
rs1816804	15	43723047	0.4182	96.49%	0.048460	0.040270	0.04774000	0.11100000	NM_021199	intron	0	Hs.511251	SQRDL
rs717224	6	25689859	0.0055	95.44%	1.000000	1.000000	0.04775000	0.99870000	ENST00000329474	intron	0	---	---
rs9300106	11	10756011	0.2615	91.93%	0.632200	0.712000	0.04775000	0.11360000	NM_014633	intron	0	Hs.173288	CTR9
rs1344421	1	61603645	0.2183	94.04%	0.472700	0.462500	0.04776000	0.21830000	NM_005595	intron	0	Hs.191911	NFIA

rs2497350	10	94511239	0.1290	97.89%	0.790900	0.560600	0.04776000	0.11060000	NM_001013848	upstream	73208	Hs.655657	EXOC6
rs2250879	8	14713113	0.3366	89.12%	0.888800	0.367400	0.04778000	0.08566000	NM_139167	intron	0	Hs.676196	SGCZ
rs17622602	7	53590451	0.0100	87.72%	1.000000	1.000000	0.04779000	0.10220000	ENST00000388026	upstream	64826	---	---
rs10516729	4	85950660	0.1300	97.19%	0.792100	0.227100	0.04780000	0.02522000	NM_178585	intron	0	Hs.480116	WDFY3
rs10517684	4	159128895	0.3367	88.07%	0.323500	0.181900	0.04780000	0.05237000	NM_016613	downstream	139114	Hs.567498	C4orf18
rs2018521	21	37439134	0.4194	95.79%	0.620500	0.445200	0.04780000	0.07018000	NM_001001894	intron	0	Hs.368214	TTC3
rs9319581	16	81539927	0.2800	96.49%	0.549400	0.548000	0.04782000	0.05136000	NM_001257	intron	0	Hs.654386	CDH13
rs7750805	6	57405705	0.1667	92.63%	1.000000	0.757800	0.04783000	0.12610000	NM_000947	intron	0	Hs.654580	PRIM2
rs10485899	7	77943497	0.1023	92.63%	1.000000	0.610000	0.04784000	0.01577000	NM_012301	intron	0	Hs.654788	MAGI2
rs1150648	6	19038539	0.3733	77.54%	0.003910	0.067670	0.04786000	0.14720000	ENST00000385041	downstream	358449	---	---
rs10507950	13	84880981	0.1522	96.84%	0.644500	0.048380	0.04788000	0.26650000	ENST00000384285	downstream	175903	---	---
rs2958187	18	51311341	0.3797	93.33%	0.696400	1.000000	0.04789000	0.01290000	NM_003199	intron	0	Hs.644653	TCF4
rs513998	20	9958393	0.1745	97.54%	1.000000	1.000000	0.04793000	0.14360000	NM_198798	upstream	5304	Hs.70903	ANKRD5
rs10501877	11	97474361	0.2108	94.04%	0.855100	0.206200	0.04794000	0.11330000	ENST00000384812	downstream	185262	---	---
rs1270028	10	65149421	0.0592	91.93%	1.000000	1.000000	0.04794000	0.13820000	NM_001001330	downstream	94534	Hs.499833	REEP3
rs4412638	1	50386263	0.2537	94.74%	0.076810	0.050250	0.04794000	0.22720000	NM_021952	intron	0	Hs.213050	ELAVL4
rs6794619	3	71122585	0.1167	94.74%	0.228000	0.662700	0.04794000	0.04258000	NM_032682	intron	0	Hs.431498	FOXP1
rs9306417	22	24629346	0.2537	94.74%	0.748900	0.518800	0.04794000	0.07979000	NM_032608	intron	0	Hs.417959	MYO18B
rs10497612	2	183225351	0.0688	94.39%	0.621700	0.616100	0.04797000	0.01826000	NM_018981	upstream	63891	Hs.516632	DNAJC10
rs10519039	15	57780050	0.0477	77.19%	0.394000	0.446300	0.04797000	0.03273000	NM_004330	upstream	11240	Hs.646490	BNIP2
rs4515397	6	96711946	0.4152	97.19%	0.620500	0.174400	0.04797000	0.16030000	NM_006581	intron	0	Hs.49117	FUT9
rs10494517	1	177770933	0.4068	97.89%	0.106300	0.449200	0.04798000	0.02800000	NM_182766	intron	0	Hs.658505	C1orf125
rs3922569	4	63297437	0.1056	88.07%	0.744500	0.299900	0.04801000	0.15940000	ENST00000314406	upstream	68534	---	---
rs9294830	6	63030582	0.0939	97.19%	0.277800	0.244700	0.04801000	0.11070000	NM_152688	intron	0	Hs.519794	KHDRBS2
rs987865	11	22871411	0.1522	96.84%	1.000000	0.605600	0.04802000	0.08371000	ENST00000354193	upstream	63454	---	---
rs4476231	17	50912628	0.2798	88.42%	0.209900	0.142600	0.04803000	0.06492000	NM_012329	upstream	58288	Hs.463483	MMD
rs2078535	7	22983055	0.3345	96.49%	0.278200	0.258400	0.04804000	0.11240000	NM_032581	intron	0	Hs.85603	FAM126A
rs788911	4	73632087	0.3226	97.89%	0.784900	0.516700	0.04805000	0.03922000	NM_014243	intron	0	Hs.590919	ADAMTS3
rs10509474	10	85038324	0.0446	94.39%	1.000000	1.000000	0.04806000	0.05077000	ENST00000363904	upstream	220517	---	---
rs10510847	3	60467192	0.3662	94.39%	0.895400	0.614300	0.04806000	0.18580000	NM_002012	intron	0	Hs.655995	FHIT
rs1861968	7	154855160	0.2798	97.19%	0.370100	0.294400	0.04806000	0.02182000	ENST00000389257	intron	0	---	---
rs1373475	4	36607819	0.0548	76.84%	0.001731	0.001972	0.04807000	0.04074000	ENST00000389006	upstream	426627	---	---
rs728123	4	105784667	0.0593	88.77%	1.000000	1.000000	0.04807000	0.02946000	NM_025212	upstream	149167	Hs.12248	CXXC4
rs1159488	13	77726771	0.2770	97.54%	0.880900	0.221400	0.04809000	0.04258000	ENST00000363816	downstream	100594	---	---
rs10510900	3	63584070	0.4173	97.54%	0.064470	0.041890	0.04810000	0.32710000	NM_144642	downstream	6434	Hs.648668	SYNPR
rs10484576	6	106817124	0.2808	96.84%	0.135200	0.316000	0.04811000	0.07181000	NM_004849	intron	0	Hs.486063	ATG5
rs1571590	9	100923629	0.1859	94.39%	0.311200	0.170100	0.04811000	0.03929000	NM_004612	intron	0	Hs.494622	TGFBR1
rs7423932	2	59803822	0.0856	92.28%	0.000967	0.161400	0.04812000	0.25330000	ENST00000386566	downstream	28332	---	---
rs300064	5	37061628	0.4273	96.49%	0.219900	0.752600	0.04813000	0.00802200	NM_015384	intron	0	Hs.481927	NIPBL
rs10504412	8	69048188	0.3759	93.33%	1.000000	0.504800	0.04814000	0.13190000	NM_025170	intron	0	Hs.591867	DEPDC2
rs10504614	8	77323739	0.1304	96.84%	0.592700	0.770500	0.04817000	0.02411000	ENST00000383892	downstream	16597	---	---
rs771320	2	7021617	0.2821	95.79%	0.764800	1.000000	0.04817000	0.04114000	NM_014746	intron	0	Hs.699230	RNF144A
rs10484161	14	82974558	0.0809	95.44%	0.688700	0.505200	0.04821000	0.05483000	ENST00000387648	downstream	149570	---	---
rs7024617	9	101179261	0.0791	97.54%	1.000000	1.000000	0.04823000	0.03538000	NM_173199	upstream	444697	Hs.279522	NR4A3
rs10505672	8	138343044	0.1386	93.68%	0.609900	0.399200	0.04824000	0.03474000	NM_015912	downstream	870664	Hs.126024	FAM135B
rs10521263	16	50870133	0.1511	97.54%	0.098850	0.067260	0.04825000	0.18380000	ENST00000219746	downstream	159293	---	---
rs1955709	14	39097616	0.1511	97.54%	0.479900	0.793500	0.04825000	0.05879000	NM_203301	upstream	126245	Hs.324342	FBXO33
rs1323147	13	42935235	0.3225	91.93%	0.065440	0.031140	0.04826000	0.16710000	NM_017993	intron	0	Hs.128258	ENOX1
rs7200614	16	50794864	0.1756	97.89%	0.036020	0.246300	0.04827000	0.11500000	ENST00000388816	downstream	235777	---	---
rs459743	5	122524830	0.1038	91.23%	0.496600	1.000000	0.04829000	0.21030000	ENST00000261364	intron	0	---	---
rs10512881	3	134177352	0.1604	94.04%	0.822600	0.537500	0.04834000	0.04730000	NM_023943	upstream	62584	Hs.191616	TMEM108
rs1538001	13	33683068	0.3000	94.74%	0.564000	0.341000	0.04834000	0.02273000	NM_015678	upstream	731388	Hs.491172	NBEA
rs9311151	3	37082134	0.0096	91.58%	1.000000	1.000000	0.04834000	0.10360000	NM_017724	intron	0	Hs.475319	LRRFIP2
rs1374491	4	130188071	0.1939	92.28%	0.048310	0.503600	0.04835000	0.16350000	NM_144643	intron	0	Hs.654690	SCLT1

rs329167	5	125161941	0.2261	91.58%	0.157200	0.155200	0.04836000	0.02283000	NM_023927	upstream	625059	Hs.363558	GRAMD3
rs4057888	11	123766132	0.2760	97.89%	0.232900	0.156400	0.04839000	0.01876000	NM_001005467	downstream	5384	Hs.690456	OR8B3
rs6072572	20	39998232	0.2380	87.72%	0.008343	0.008037	0.04839000	0.10950000	NM_007050	downstream	136574	Hs.526879	PTPRT
rs10483622	14	53206281	0.1148	94.74%	1.000000	1.000000	0.04840000	0.11480000	NM_130851	downstream	279926	Hs.68879	BMP4
rs1845389	8	119869566	0.4209	97.54%	0.806400	0.653900	0.04841000	0.13160000	NM_002546	downstream	135412	Hs.81791	TNFRSF11B
rs685286	11	102347662	0.2130	94.74%	0.017570	0.096090	0.04841000	0.05550000	NM_002427	upstream	15990	Hs.2936	MMP13
rs3950256	2	217519913	0.4275	96.84%	0.110500	0.876200	0.04842000	0.22010000	NM_003284	upstream	86886	Hs.3017	TNP1
rs6129969	20	39967987	0.1381	94.04%	0.608800	1.000000	0.04847000	0.04634000	NM_007050	downstream	166819	Hs.526879	PTPRT
rs17105166	5	146047763	0.1171	94.39%	0.382500	0.203600	0.04848000	0.01434000	NM_181678	intron	0	Hs.655213	PPP2R2B
rs1424038	16	61521954	0.2778	97.89%	0.765400	0.691100	0.04851000	0.04817000	NM_001797	downstream	2016232	Hs.116471	CDH11
rs4102214	11	62969296	0.2318	91.58%	0.298700	0.815000	0.04851000	0.15050000	NM_054108	downstream	18090	Hs.410316	HRASLS5
rs4310018	5	152266397	0.2818	96.49%	0.882100	0.430400	0.04852000	0.15490000	NM_000827	upstream	584102	Hs.519693	GRIA1
rs1334339	1	88182865	0.3243	90.88%	0.064640	0.130100	0.04854000	0.05705000	ENST00000362448	upstream	491221	---	---
rs1893241	18	21752217	0.2575	94.04%	0.749000	0.522100	0.04855000	0.01974000	NM_005637	downstream	98000	Hs.404263	SS18
rs10502516	18	25176204	0.2929	94.04%	0.556400	0.483000	0.04856000	0.06502000	ENST00000384802	downstream	145714	---	---
rs2973519	5	11131958	0.3864	92.63%	0.697400	0.868700	0.04857000	0.08794000	NM_001332	intron	0	Hs.314543	CTNND2
rs10510947	3	65804384	0.4188	97.19%	0.902300	0.752000	0.04859000	0.04733000	NM_001033057	intron	0	Hs.651939	MAGI1
rs1925757	13	45084977	0.2191	82.46%	0.848500	1.000000	0.04861000	0.13020000	ENST00000298738	intron	0	---	---
rs4073846	1	46235468	0.4907	94.74%	0.466800	0.647900	0.04861000	0.18100000	NM_015112	intron	0	Hs.319481	MAST2
rs1863702	2	49714260	0.0939	97.19%	0.718500	0.465200	0.04862000	0.06830000	ENST00000388647	downstream	255250	---	---
rs9322105	6	148065767	0.0939	97.19%	0.718500	0.465200	0.04862000	0.06462000	NM_001030060	downstream	132917	Hs.567973	SAMD5
rs716180	7	134087371	0.2703	90.88%	0.207800	0.145900	0.04864000	0.05985000	NM_033140	upstream	27340	Hs.490203	CALD1
rs1498843	3	69663591	0.2593	94.04%	0.205000	0.043700	0.04865000	0.06822000	NM_198178	upstream	232061	Hs.166017	MITF
rs2710831	4	169645396	0.2489	77.54%	0.374500	0.468000	0.04865000	0.11420000	NM_016081	upstream	9396	Hs.151220	PALLD
rs3845641	2	141563449	0.3635	95.09%	0.792900	0.274400	0.04867000	0.03809000	NM_018557	intron	0	Hs.656461	LRP1B
rs10484010	14	88368041	0.1529	97.54%	0.486400	0.441000	0.04868000	0.10380000	NM_198310	intron	0	Hs.303055	TTC8
rs945089	1	230886240	0.4039	89.47%	0.365700	0.426700	0.04868000	0.03004000	ENST00000366656	intron	0	---	---
rs10506098	12	33034204	0.3630	94.74%	1.000000	1.000000	0.04870000	0.03288000	NM_004572	upstream	93211	Hs.164384	PKP2
rs706358	1	56466767	0.4889	94.74%	0.807500	0.443100	0.04871000	0.03015000	NM_177414	downstream	265760	Hs.405156	PPAP2B
rs10512873	3	133842102	0.0448	94.04%	0.416500	1.000000	0.04872000	0.06963000	NM_032169	intron	0	Hs.441378	ACAD11
rs2320127	16	63340114	0.3206	91.93%	0.395600	0.581500	0.04872000	0.14360000	NM_001797	downstream	198072	Hs.116471	CDH11
rs9318422	13	75813311	0.1324	83.51%	0.149500	0.686800	0.04872000	0.02969000	NM_138444	downstream	538991	Hs.693617	KCTD12
rs1986413	11	94077583	0.3273	97.54%	0.275500	0.719700	0.04873000	0.08079000	NM_130847	upstream	63602	Hs.503594	AMOTL1
rs891552	11	94101220	0.3273	97.54%	0.275500	0.719700	0.04873000	0.07942000	NM_130847	upstream	39965	Hs.503594	AMOTL1
rs960297	6	120942274	0.4263	97.54%	0.461700	0.365800	0.04873000	0.07807000	ENST00000384130	upstream	53029	---	---
rs1900451	10	55347706	0.2055	96.49%	1.000000	1.000000	0.04875000	0.10350000	NM_033056	intron	0	Hs.672170	PCDH15
rs340199	4	86569707	0.2383	97.19%	0.412400	0.440900	0.04875000	0.04723000	NM_001042669	upstream	500743	Hs.444229	ARHGAP24
rs10490596	2	207887764	0.3280	97.89%	0.058010	0.103900	0.04876000	0.14820000	NM_003709	upstream	149087	Hs.471221	KLF7
rs10510083	10	122674515	0.1642	92.98%	0.656700	1.000000	0.04876000	0.07794000	ENST00000369065	intron	0	---	---
rs10510885	3	62637152	0.1123	96.84%	0.359900	0.198800	0.04877000	0.11540000	NM_183393	intron	0	Hs.654933	CADPS
rs4800933	18	51580839	0.3019	94.74%	0.385600	0.735400	0.04879000	0.06867000	NM_003199	upstream	173981	Hs.644653	TCF4
rs1503086	3	106285667	0.2528	95.09%	1.000000	0.385200	0.04880000	0.09698000	NM_001627	upstream	282736	Hs.591293	ALCAM
rs9297181	8	29996147	0.1523	97.89%	0.485400	1.000000	0.04880000	0.01088000	ENST00000387787	downstream	24726	---	---
rs2014366	2	235393894	0.0139	88.42%	1.000000	1.000000	0.04881000	0.05740000	NM_014521	upstream	158174	Hs.516777	SH3BP4
rs10483763	14	62636534	0.4314	97.19%	0.393400	0.651000	0.04883000	0.09797000	NM_172375	upstream	54825	Hs.27043	KCNH5
rs10495539	2	6706226	0.0540	97.54%	1.000000	1.000000	0.04884000	0.01863000	NM_001001695	downstream	80557	---	FLJ42418
rs10506863	12	80977957	0.0540	97.54%	0.562500	1.000000	0.04884000	0.06075000	NM_014167	downstream	292798	Hs.582627	CCDC59
rs10494435	1	163442188	0.2584	94.39%	0.525100	0.831100	0.04888000	0.22270000	NM_001033507	intron	0	Hs.667312	LMX1A
rs10502126	11	110423186	0.0093	94.39%	1.000000	1.000000	0.04889000	0.09352000	NM_198498	upstream	208731	Hs.298685	C11orf53
rs10517988	4	67184679	0.1265	59.65%	0.000000	0.000231	0.04889000	0.30450000	ENST00000339867	downstream	541842	---	---
rs10494674	1	191359364	0.3011	94.39%	0.563100	0.608300	0.04890000	0.05372000	NM_024529	intron	0	Hs.576497	CDC73
rs1456991	14	97558657	0.0280	94.04%	1.000000	1.000000	0.04892000	0.01783000	ENST00000380268	upstream	259736	---	---
rs1907091	4	158822236	0.2088	95.79%	0.713800	1.000000	0.04892000	0.07678000	NM_000826	downstream	315559	Hs.32763	GRIA2
rs10505201	8	114295819	0.1410	93.33%	0.072390	0.174100	0.04894000	0.05754000	NM_052900	intron	0	Hs.91381	CSMD3

rs10482977	21	27014402	0.1111	97.89%	0.126000	0.089390	0.04895000	0.14070000	NM_006988	downstream	116077	Hs.643357	ADAMTS1
rs2149814	9	10885645	0.1111	97.89%	0.221300	0.047270	0.04895000	0.01492000	ENST00000364783	downstream	1404625	---	---
rs1568050	12	60077380	0.0095	92.28%	1.000000	1.000000	0.04898000	0.06472000	NM_178539	downstream	310942	Hs.269745	FAM19A2
rs7754169	6	91370279	0.3759	94.74%	0.439600	0.323200	0.04899000	0.17760000	NM_145333	upstream	16651	Hs.644143	MAP3K7
rs7515996	1	192591449	0.0953	97.54%	0.724100	1.000000	0.04900000	0.03199000	ENST00000385495	downstream	132407	---	---
rs9293956	6	63510535	0.3708	95.09%	0.696100	0.532400	0.04904000	0.01910000	ENST00000384941	upstream	59403	---	---
rs10491406	5	162909423	0.0935	97.54%	0.486600	0.701900	0.04906000	0.00877000	NM_182796	downstream	30519	Hs.696057	MAT2B
rs17088462	4	58190120	0.1031	91.93%	0.495400	1.000000	0.04907000	0.05377000	ENST00000387903	upstream	136824	---	---
rs1321464	20	52465664	0.1605	95.09%	0.651500	1.000000	0.04910000	0.12490000	NM_018431	upstream	59924	Hs.656582	DOK5
rs7608543	2	36416477	0.3787	94.04%	0.603800	0.349600	0.04912000	0.03656000	NM_016441	upstream	20096	Hs.699247	CRIM1
rs10485562	20	16688575	0.1733	52.63%	1.000000	0.417100	0.04913000	0.01302000	NM_020157	downstream	7766	Hs.41119	OTOR
rs1121946	8	129234380	0.1917	93.33%	0.845500	0.582200	0.04917000	0.02909000	ENST00000364912	upstream	67552	---	---
rs2374544	12	104659538	0.3007	95.09%	0.471700	0.036260	0.04918000	0.05916000	NM_014840	downstream	321717	Hs.524692	NUAK1
rs10498999	6	96776326	0.4209	97.54%	0.538300	0.174100	0.04919000	0.09248000	NM_006581	downstream	15849	Hs.49117	FUT9
rs7713115	5	86607880	0.4739	87.37%	0.056460	0.110800	0.04921000	0.08650000	NM_022650	intron	0	Hs.695926	RASA1
rs10511610	9	15262462	0.0685	76.84%	1.000000	1.000000	0.04922000	0.06289000	NM_152574	intron	0	Hs.563630	C9orf52
rs10506656	12	71247495	0.2220	94.04%	0.077450	0.001088	0.04923000	0.04309000	NM_013381	intron	0	Hs.199814	TRHDE
rs4688284	3	62062420	0.0789	97.89%	0.393100	1.000000	0.04923000	0.04448000	NM_002841	intron	0	Hs.654488	PTPRG
rs17622017	1	192607831	0.0731	91.23%	0.143000	0.410700	0.04924000	0.13650000	ENST00000385495	downstream	116025	---	---
rs2214586	7	11591954	0.1007	94.04%	0.738500	1.000000	0.04927000	0.15470000	ENST00000262042	intron	0	---	---
rs248257	5	179341142	0.3628	75.44%	0.001867	0.002112	0.04928000	0.05596000	NM_018434	intron	0	Hs.484363	RNF130
rs1903991	10	53570878	0.2076	97.19%	0.714800	0.455200	0.04929000	0.16860000	NM_006258	intron	0	Hs.654556	PRKG1
rs7149815	14	81417277	0.0806	95.79%	0.396800	0.505200	0.04929000	0.07754000	NM_005065	upstream	347391	Hs.181300	SEL1L
rs1434611	4	162937760	0.3102	93.33%	0.318700	0.500900	0.04930000	0.08808000	NM_020116	intron	0	Hs.591707	FSTL5
rs669233	11	93669262	0.4293	96.84%	0.390400	0.762100	0.04930000	0.05227000	NM_015368	downstream	114481	Hs.591976	PANX1
rs1922296	2	102189311	0.2401	97.89%	0.744200	1.000000	0.04932000	0.02976000	NM_003854	intron	0	Hs.659863	IL1RL2
rs2642002	9	582718	0.2401	97.89%	0.001653	0.003097	0.04932000	0.19790000	NM_015158	intron	0	Hs.306764	ANKRD15
rs4977879	9	23659371	0.2401	97.89%	0.249400	0.501700	0.04932000	0.04689000	NM_004432	downstream	22438	Hs.166109	ELAVL2
rs10509698	10	97782342	0.0542	97.19%	1.000000	1.000000	0.04933000	0.10240000	NM_001001732	3UTR	0	Hs.652240	CC2D2B
rs6715726	2	195874055	0.1534	97.19%	0.247800	0.084870	0.04933000	0.17270000	ENST00000365336	upstream	212952	---	---
rs10493922	1	100692376	0.1370	94.74%	0.065220	0.048850	0.04934000	0.10280000	NM_033313	intron	0	Hs.127411	CDC14A
rs4597125	12	74694839	0.0942	96.84%	0.486300	0.602500	0.04934000	0.18690000	NM_007350	downstream	15744	Hs.602085	PHLDA1
rs817429	1	85408319	0.1370	94.74%	0.037940	0.084040	0.04934000	0.02718000	ENST00000341460	intron	0	---	---
rs1984067	9	104452452	0.1577	84.56%	0.146000	0.055990	0.04936000	0.14910000	ENST00000374800	intron	0	---	---
rs9294148	6	80265632	0.1904	83.86%	0.089800	0.039650	0.04936000	0.01584000	NM_181714	intron	0	Hs.21945	LCA5
rs10508279	10	4290734	0.1617	94.39%	0.653000	0.803100	0.04937000	0.34840000	NM_001300	upstream	473267	Hs.4055	KLF6
rs2291498	3	56633911	0.1617	94.39%	0.178700	0.134600	0.04937000	0.04565000	NM_015224	CDS	0	Hs.116877	C3orf63
rs1487377	18	20137550	0.1636	94.39%	1.000000	1.000000	0.04938000	0.10200000	NM_080597	intron	0	Hs.370725	OSBPL1A
rs200790	12	97791214	0.2381	81.05%	0.017210	0.138100	0.04938000	0.16330000	NM_020140	intron	0	Hs.506458	ANKS1B
rs10496823	2	140035465	0.0996	93.33%	0.731900	0.711300	0.04940000	0.04828000	ENST00000384174	downstream	49870	---	---
rs1562533	3	105328962	0.0449	93.68%	0.417700	1.000000	0.04943000	0.18960000	ENST00000388643	downstream	33252	---	---
rs1825478	12	26529224	0.2326	90.53%	0.002622	0.026590	0.04945000	0.17190000	NM_002223	intron	0	Hs.512235	ITPR2
rs10483361	14	30343066	0.1429	93.33%	0.317100	1.000000	0.04947000	0.14960000	NM_182835	downstream	68297	Hs.369168	SCFD1
rs8046846	16	78392110	0.0455	92.63%	0.011150	0.152100	0.04947000	0.30900000	NM_001031804	upstream	199998	Hs.134859	MAF
rs1491520	3	193243145	0.0964	96.49%	0.294400	1.000000	0.04948000	0.02929000	ENST00000364513	upstream	50460	---	---
rs7731154	5	162178752	0.4963	95.44%	0.395700	0.880300	0.04948000	0.04568000	NM_199246	upstream	618403	Hs.79101	CCNG1
rs7963098	12	51406976	0.2061	97.89%	0.463200	0.533400	0.04949000	0.18720000	NM_175078	upstream	23462	Hs.334989	KRT77
rs10488979	4	13984477	0.0092	95.09%	1.000000	1.000000	0.04951000	0.06345000	NM_148894	upstream	746032	Hs.444517	FAM44A
rs10502125	11	110422773	0.0092	95.09%	1.000000	1.000000	0.04951000	0.09463000	NM_198498	upstream	209144	Hs.298685	C11orf53
rs10508156	13	104676816	0.0092	95.09%	1.000000	1.000000	0.04951000	0.12390000	NM_172370	upstream	239777	Hs.381382	DAOA
rs1524476	7	115436045	0.0092	95.09%	1.000000	1.000000	0.04951000	0.07315000	NM_001018058	intron	0	Hs.125962	TFEC
rs6996396	8	33097918	0.0092	95.09%	1.000000	1.000000	0.04951000	0.05936000	ENST00000387861	upstream	103601	---	---
rs10489358	1	166921497	0.2196	95.09%	0.158600	0.333700	0.04952000	0.03162000	NM_001937	downstream	9824	Hs.80552	DPT
rs10496840	2	140767078	0.3037	84.91%	0.047750	0.109400	0.04952000	0.04512000	NM_018557	intron	0	Hs.656461	LRP1B

rs10492156	12	14710038	0.0109	80.70%	0.021710	1.000000	0.04953000	0.14910000	NM_004963	intron	0	Hs.524278	GUCY2C
rs912171	9	646765	0.1198	92.28%	0.232900	0.334500	0.04953000	0.04004000	NM_015158	intron	0	Hs.306764	ANKRD15
rs10495459	1	238382591	0.4435	80.70%	0.349400	0.738000	0.04954000	0.21820000	NM_020066	intron	0	---	FMN2
rs1366669	8	15513049	0.2076	97.19%	0.584900	0.146700	0.04954000	0.01504000	NM_178234	intron	0	Hs.591845	TUSC3
rs1635516	1	240131955	0.2076	97.19%	0.584900	0.836300	0.04954000	0.08641000	NM_003686	downstream	12091	Hs.498248	EXO1
rs530723	9	109779657	0.3460	96.84%	1.000000	0.751500	0.04955000	0.05976000	ENST00000297812	upstream	142726	---	---
rs10503965	8	34642766	0.2363	89.82%	0.117800	0.354000	0.04956000	0.20860000	NM_080872	upstream	878686	Hs.238889	UNC5D
rs10490242	2	50168999	0.2814	97.89%	0.377100	0.599600	0.04957000	0.18940000	NM_138735	intron	0	Hs.637685	NRXN1
rs2421802	2	126387856	0.1034	91.58%	0.744500	0.721700	0.04957000	0.05183000	NM_016815	upstream	776688	Hs.59138	GYPC
rs10490987	10	117679868	0.2086	97.54%	0.585200	0.836700	0.04958000	0.06200000	NM_207303	intron	0	Hs.501127	ATRNL1
rs1536170	1	65551917	0.2086	97.54%	0.279000	0.210500	0.04958000	0.02964000	NM_014787	intron	0	Hs.647643	DNAJC6
rs2041739	2	102360765	0.4265	97.89%	0.086300	0.099900	0.04958000	0.37890000	NM_003855	intron	0	Hs.469521	IL18R1
rs802424	7	86132630	0.2410	97.54%	0.745000	0.377100	0.04960000	0.21120000	NM_000840	intron	0	Hs.590575	GRM3
rs521693	6	93930690	0.2593	94.74%	0.266000	0.203300	0.04962000	0.22530000	NM_004440	downstream	77174	Hs.73962	EPHA7
rs10484368	6	18471817	0.1280	89.12%	0.019520	0.027350	0.04963000	0.03099000	NM_182757	upstream	23756	Hs.148741	RNF144B
rs12644496	4	152324798	0.1221	90.53%	1.000000	1.000000	0.04965000	0.03634000	NM_001009555	intron	0	Hs.567725	SH3D19
rs258759	5	142536786	0.1825	96.14%	0.687400	0.494500	0.04965000	0.09363000	NM_015071	intron	0	Hs.654668	ARHGAP26
rs10494395	1	161894333	0.0717	92.98%	1.000000	1.000000	0.04966000	0.05108000	ENST00000385703	downstream	148347	---	---
rs962222	1	92743541	0.1423	93.68%	1.000000	0.788700	0.04966000	0.01397000	NM_005665	downstream	3300	Hs.656836	EVI5
rs10514137	18	70522812	0.1350	96.14%	0.191100	0.702800	0.04967000	0.21590000	NM_017757	intron	0	Hs.536490	ZNF407
rs2484749	1	39531542	0.2101	96.84%	0.585400	1.000000	0.04967000	0.06927000	NM_012090	intron	0	Hs.580782	MACF1
rs2862078	3	154695022	0.4690	96.14%	0.395700	0.361000	0.04967000	0.07046000	ENST00000383016	intron	0	---	---
rs10505294	8	118613249	0.2222	94.74%	1.000000	1.000000	0.04968000	0.02211000	NM_080651	intron	0	Hs.492612	MED30
rs3211830	7	80117654	0.1352	85.61%	0.782900	1.000000	0.04968000	0.02052000	NM_000072	intron	0	Hs.120949	CD36
rs6938648	6	3954503	0.2326	80.70%	0.042640	0.287000	0.04968000	0.34010000	ENST00000366365	downstream	4485	---	---
rs10511668	9	19012470	0.4386	97.19%	0.626500	1.000000	0.04970000	0.05012000	NM_153707	intron	0	Hs.98943	C9orf138
rs9321092	6	127856574	0.2613	93.33%	0.528900	0.853600	0.04971000	0.15290000	NM_001012279	intron	0	Hs.319247	C6orf174
rs10520985	5	31560322	0.4564	96.49%	0.903300	0.548700	0.04972000	0.06233000	NM_013235	intron	0	Hs.97997	RNASEN
rs9287517	2	139874529	0.1199	93.68%	1.000000	1.000000	0.04972000	0.05781000	ENST00000384174	upstream	110935	---	---
rs10520007	5	128180413	0.0858	94.04%	0.423400	0.566500	0.04973000	0.09651000	NM_014031	upstream	148307	Hs.49765	SLC27A6
rs9378878	6	4397665	0.0709	94.04%	0.629100	0.401300	0.04973000	0.05053000	NM_001011540	upstream	158180	---	KU-MEL-3
rs927180	6	63572152	0.4451	89.47%	0.001546	0.006259	0.04974000	0.06758000	ENST00000384941	upstream	121020	---	---
rs972246	7	8551314	0.2346	91.23%	0.225500	0.102300	0.04974000	0.12810000	NM_152745	upstream	205849	Hs.487564	NXPH1
rs10509128	10	61721033	0.2930	95.79%	0.381000	0.732000	0.04976000	0.03793000	NM_020987	intron	0	Hs.499725	ANK3
rs10509573	10	91179201	0.1184	93.33%	0.552200	0.205300	0.04977000	0.12420000	NM_213606	downstream	1552	Hs.530338	SLC16A12
rs9326370	11	65748584	0.4779	87.37%	0.022200	0.076600	0.04977000	0.04082000	NM_018026	intron	0	Hs.644326	PACS1
rs997934	10	1785194	0.2741	90.88%	0.160700	0.682900	0.04977000	0.13000000	NM_018702	upstream	15524	Hs.657984	ADARB2
rs10506025	12	27617637	0.3170	92.98%	0.776400	0.616600	0.04978000	0.09424000	NM_177444	intron	0	Hs.172445	PPFIBP1
rs2291385	6	159104665	0.0279	94.39%	0.181800	1.000000	0.04978000	0.07624000	NM_001009991	intron	0	Hs.436977	SYTL3
rs1605714	5	98064521	0.0285	92.28%	0.013890	1.000000	0.04979000	0.07146000	NM_173670	upstream	68379	Hs.526902	RGMB
rs1828674	12	129035565	0.1137	97.19%	0.063940	0.647400	0.04979000	0.25930000	NM_133448	upstream	81728	Hs.507268	TMEM132D
rs9323293	14	56024440	0.1557	95.79%	0.353900	0.316600	0.04979000	0.16010000	NM_017799	upstream	91824	Hs.497253	C14orf101
rs10518078	4	71838557	0.0701	95.09%	0.378100	1.000000	0.04980000	0.09024000	NM_014961	intron	0	Hs.7972	RUFY3
rs2823527	21	16270439	0.0701	95.09%	0.378100	1.000000	0.04980000	0.13880000	NM_013396	downstream	96191	Hs.473370	USP25
rs10514020	18	67537751	0.0978	95.09%	0.728400	1.000000	0.04981000	0.05875000	NM_182511	downstream	817144	Hs.569851	CBLN2
rs7751261	6	5486393	0.1818	96.49%	0.108400	0.146400	0.04981000	0.03544000	NM_006567	intron	0	Hs.484547	FARS2
rs10491068	10	97946892	0.0538	97.89%	1.000000	1.000000	0.04982000	0.10190000	NM_013314	intron	0	Hs.665244	BLNK
rs1547167	13	42144097	0.0099	88.77%	0.019740	1.000000	0.04982000	0.20020000	NM_033012	downstream	64789	Hs.333791	TNFSF11
rs1676965	8	4560250	0.0538	97.89%	1.000000	1.000000	0.04982000	0.01937000	ENST00000383934	downstream	412959	---	---
rs4456365	13	96616515	0.0538	97.89%	0.561100	1.000000	0.04982000	0.12220000	NM_207304	upstream	56094	Hs.657347	MBNL2
rs2421528	5	159948254	0.3745	93.68%	0.361600	0.611400	0.04984000	0.17570000	ENST00000327245	intron	0	---	---
rs10512057	9	78790963	0.4319	97.89%	0.627100	0.454500	0.04985000	0.09614000	NM_001013735	upstream	33428	Hs.553843	FOXB2
rs6472776	8	74530495	0.1540	96.84%	0.018230	0.085400	0.04985000	0.16370000	ENST00000343268	intron	0	---	---
rs10484764	6	131628795	0.0728	89.12%	1.000000	1.000000	0.04986000	0.02530000	NM_016377	intron	0	Hs.486483	AKAP7

rs1420984	16	49966235	0.0957	97.19%	0.724700	1.000000	0.04986000	0.00308600	NM_002968	upstream	223582	Hs.135787	SALL1
rs2700169	12	70301317	0.4083	84.21%	0.790200	1.000000	0.04988000	0.08678000	NM_144982	intron	0	Hs.527874	CCDC131
rs6557512	6	157242759	0.2239	94.04%	0.379500	0.814800	0.04988000	0.20300000	NM_175863	intron	0	Hs.291587	ARID1B
rs6848573	4	182523529	0.2528	95.09%	0.259400	0.078620	0.04988000	0.22260000	ENST00000378957	upstream	157278	---	---
rs10500902	11	21242404	0.0800	96.49%	0.080110	0.503100	0.04989000	0.07811000	NM_006157	intron	0	Hs.657172	NELL1
rs10514623	2	108329924	0.0800	96.49%	0.019810	0.020150	0.04989000	0.04070000	NM_006588	upstream	30929	Hs.312644	SULT1C4
rs4129740	8	121902146	0.3406	96.84%	0.893400	0.860600	0.04989000	0.13080000	NM_021021	upstream	8882	Hs.655236	SNTB1
rs10511162	3	98853627	0.0578	94.04%	0.601700	1.000000	0.04990000	0.16370000	ENST00000389672	intron	0	---	---
rs1911361	10	67976917	0.0578	94.04%	0.212500	1.000000	0.04990000	0.03979000	NM_013266	intron	0	Hs.660362	CTNNA3
rs10501020	11	25686881	0.2079	97.89%	0.717200	0.613500	0.04992000	0.07155000	NM_031418	upstream	622666	Hs.91791	TMEM16C
rs2388557	10	3181527	0.3745	95.09%	0.796200	0.758100	0.04992000	0.04888000	NM_014889	intron	0	Hs.528300	PITRM1
rs2778961	10	20603224	0.2832	97.89%	0.055020	0.171200	0.04992000	0.09928000	NM_032812	intron	0	Hs.658134	PLXDC2
rs7549499	1	83790546	0.2832	97.89%	1.000000	1.000000	0.04992000	0.02485000	NM_024686	downstream	312753	Hs.445826	TTLL7
rs7672957	4	12269913	0.4072	92.63%	0.373700	0.421900	0.04992000	0.05919000	ENST00000387175	downstream	346501	---	---
rs10507674	13	61332602	0.0549	95.79%	0.185600	0.022710	0.04994000	0.01950000	ENST00000387130	upstream	202391	---	---
rs1041044	10	10644387	0.3081	95.09%	0.671300	0.866700	0.04996000	0.07709000	NM_001025077	upstream	603069	Hs.309288	CUGBP2
rs9325098	5	147807631	0.2659	93.68%	0.875900	1.000000	0.04996000	0.02464000	NM_001040174	downstream	3241	Hs.483773	HTR4
