# Supplementary material for: Identification of Diabetic Retinopathy Genes through a Genome-Wide Association Study among Mexican-Americans from Starr County, Texas
Source: J Ophthalmol. 2010 Sep 2;2010:861291. doi: 10.1155/2010/861291 (PMC2939442; doi:10.1155/2010/861291)
Supplement: Supplementary file 4 [file 861291.f4.pdf]

Supplementary Table 2. Imputation quality of SNP markers used in MACH

chr	# of SNPs in original Affymetrix 100K set	# of SNPs used for imputation from Affymetrix 100K set after QC	# of SNPs available from HapMap3 MXL	# of SNPs imputed from HapMap III MXL	MACH estimated per allele imputation error rate	# of imputed SNPs with MAF>1%	MACH imputation r^2>=0.5	%
**1**	9,165	8,403	116,415	111,422	8.54%	96,923	35,010	36.12%
**2**	10,309	9,545	116,430	110,965	8.03%	97,475	39,557	40.58%
**3**	7,798	7,256	96,537	92,265	8.36%	81,578	30,324	37.17%
**4**	8,537	7,795	85,772	81,382	8.50%	71,673	30,032	41.90%
**5**	8,329	7,769	87,919	83,421	8.40%	74,099	31,645	42.71%
**6**	8,040	7,565	91,357	87,239	8.22%	78,260	32,845	41.97%
**7**	7,023	6,589	75,320	71,610	8.18%	64,135	24,313	37.91%
**8**	6,946	6,411	75,272	71,782	7.99%	63,568	25,086	39.46%
**9**	4,768	4,429	63,612	61,032	9.22%	54,610	17,868	32.72%
**10**	5,656	5,306	73,832	70,715	8.56%	63,642	22,616	35.54%
**11**	5,338	4,942	70,973	68,112	8.77%	59,716	21,920	36.71%
**12**	5,245	4,909	68,525	65,644	9.11%	58,484	20,000	34.20%
**13**	5,199	4,818	51,915	49,233	8.79%	43,721	17,418	39.84%
**14**	3,997	3,726	45,474	43,275	8.95%	38,672	14,908	38.55%
**15**	3,022	2,761	42,353	40,598	9.53%	35,629	11,304	31.73%
**16**	2,372	2,142	44,648	43,283	10.35%	37,650	8,317	22.09%
**17**	1,945	1,824	38,401	37,229	10.35%	32,254	7,670	23.78%
**18**	3,552	3,305	40,824	38,965	9.00%	34,679	11,339	32.70%
**19**	686	636	26,238	25,833	13.22%	22,608	2,595	11.48%
**20**	2,086	1,951	36,258	34,998	10.05%	31,254	8,101	25.92%
**21**	1,895	1,801	19,306	18,332	9.27%	16,532	5,395	32.63%
**22**	758	689	20,085	19,655	11.98%	17,265	2,747	15.91%
**Total**	112,666	104,572	1,387,466	1,326,990	8.70%	1,174,427	421,010	35.85%

* r^2: Estimated squared correlation between imputed and true genotypes.
