# Supplementary material for: Identification of Diabetic Retinopathy Genes through a Genome-Wide Association Study among Mexican-Americans from Starr County, Texas
Source: J Ophthalmol. 2010 Sep 2;2010:861291. doi: 10.1155/2010/861291 (PMC2939442; doi:10.1155/2010/861291)
Supplement: Supplementary file 5 [file 861291.f5.pdf]

Official symbol	Location	GeneID	Official full name
ACE	17q23.3	1636	angiotensin I converting enzyme 1
ADAMTS5	21q21.3	11096	ADAM metallopeptidase with thrombospondin type 1 motif 5
ADD1	4p16.3	118	adducin 1
ADRA2C	4p16	152	adrenergic, alpha-2C-, receptor
ADRB1	10q24-q26	153	adrenergic, beta-1-, receptor
ADRB3	8p12-p11.2	155	adrenergic, beta-3-, receptor
AGT	1q42-q43	183	angiotensinogen
AGTR1	3q21-q25	185	angiotensin II receptor, type 1
AKR1B1	7q35	231	aldo-keto reductase family 1, member B1 (aldose reductase)
AKR1B10	7q33	57016	aldo-keto reductase family 1, member B10 (aldose reductase)
ALDH2	12q24.2	217	aldehyde dehydrogenase 2 family (mitochondrial)
ANGPT1	8q22.3-q23	284	angiopoietin 1
ANGPT2	8p23.1	285	angiopoietin 2
ANKRD50	4q28.1	57182	ankyrin repeat domain 50
APOB	2p24-p23	338	apolipoprotein B
APOE	19q13.2	348	apolipoprotein E
ARF6	14q21.3	382	ADP-ribosylation factor 6
ARL6	3q11.2	84100	ADP-ribosylation factor-like 6
ARRB2	17p13	409	arrestin, beta 2
ASIP	20q11.2-q12	434	agouti signaling protein, nonagouti homolog (mouse)
ATP1A1	1p21	476	ATPase, Na+/K+ transporting, alpha 1 polypeptide
BAX	19q13.3-q13.4	581	BCL2-associated X protein
BBS2	16q21	583	Bardet-Biedl syndrome 2
CACNA1E	1q25-q31	777	calcium channel, voltage-dependent, R type, alpha 1E subunit
CAPN10	2q37.3	11132	calpain 10
CAPN3	15q15.1-q21.1	825	calpain 3, (p94)
CASP3	4q34	836	caspase 3, apoptosis-related cysteine peptidase
CASP9	1p36.3-p36.1	842	caspase 9, apoptosis-related cysteine peptidase
CAT	11p13	847	catalase
CCL2	17q11.2-q12	6347	chemokine (C-C motif) ligand 2
CCL3	17q11-q21	6348	chemokine (C-C motif) ligand 3
CCL5	17q11.2-q12	6352	chemokine (C-C motif) ligand 5
CCR5	3p21.31	1234	chemokine (C-C motif) receptor 5

CD4	12pter-p12	920	CD4 molecule
CD8A	2p12	925	CD8a molecule
CDC42BPA	1q42.11	8476	CDC42 binding protein kinase alpha
CDKN2A	9p21	1029	cyclin-dependent kinase inhibitor 2A
CDKN2B	9p21	1030	cyclin-dependent kinase inhibitor 2B
CFH	1q32	3075	complement factor H
CHD6	20q12	84181	chromodomain helicase DNA binding protein 6
COL4A1	13q34	1282	collagen, type IV, alpha 1
COMT	22q11.21-q11.23	1312	catechol-O-methyltransferase
CRH	8q13	1392	corticotropin releasing hormone
CRX	19q13.3	1406	cone-rod homeobox
CXCL10	4q21	3627	chemokine (C-X-C motif) ligand 10
CXCL12	10q11.1	6387	chemokine (C-X-C motif) ligand 12
CXCL5	4q12-q13	6374	chemokine (C-X-C motif) ligand 5
CYP11B2	8q21-q22	1585	cytochrome P450, family 11, subfamily B, polypeptide 2
DDIT4	10pter-q26.12	54541	DNA-damage-inducible transcript 4
DGKA	12q13.3	1606	diacylglycerol kinase, alpha 80kDa
DRD1	5q35.1	1812	dopamine receptor D1
DRD2	11q23	1813	dopamine receptor D2
DRD3	3q13.3	1814	dopamine receptor D3
DRD4	11p15.5	1815	dopamine receptor D4
E2F1	20q11.2	1869	E2F transcription factor 1
EDN1	6p24.1	1906	endothelin 1
EDN2	1p34	1907	endothelin 2
EDN3	20q13.2-q13.3	1908	endothelin 3
EDNRA	4q31.23	1909	endothelin receptor type A
EDNRB	13q22	1910	endothelin receptor type B
EFNB2	13q33	1948	ephrin-B2
ENPP1	6q22-q23	5176	ectonucleotide pyrophosphatase/phosphodiesterase 1
EPHB4	7q22	2050	EPH receptor B4
EPO	7q22	2056	erythropoietin
EPOR	19p13.3-13.2	2057	erythropoietin receptor
FGF2	4q26-q27	2247	fibroblast growth factor 2 (basic)
FIZ1	19q13.42	84922	FLT3-interacting zinc finger 1

FLT1	13q12	2321	fms-related tyrosine kinase 1
GABBR1	6p21.31	2550	gamma-aminobutyric acid (GABA) B receptor, 1
GAD1	2q31	2571	glutamate decarboxylase 1 (brain, 67kDa)
GAPDHS	19q13.12	26330	glyceraldehyde-3-phosphate dehydrogenase, spermatogenic
GCG	2q36-q37	2641	glucagon
GCGR	17q25	2642	glucagon receptor
GCK	7p15.3-p15.1	2645	glucokinase (hexokinase 4)
GFAP	17q21	2670	glial fibrillary acidic protein
GHRL	3p26-p25	51738	ghrelin/obestatin prepropeptide
GJA1	6q21-q23.2	2697	gap junction protein, alpha 1, 43kDa
GLP1R	6p21	2740	glucagon-like peptide 1 receptor
GMNN	6p22.2	51053	geminin, DNA replication inhibitor
GNAS	20q13.3	2778	GNAS complex locus
GNB3	12p13	2784	guanine nucleotide binding protein (G protein), beta polypeptide 3
GP1BA	17pter-p12	2811	glycoprotein Ib (platelet), alpha polypeptide
GPX4	19p13.3	2879	glutathione peroxidase 4
GRIK1	21q22.11	2897	glutamate receptor, ionotropic, kainate 1
GRIN1	9q34.3	2902	glutamate receptor, ionotropic, N-methyl D-aspartate 1
GSK3A	19q13.2	2931	glycogen synthase kinase 3 alpha
GSK3B	3q13.3	2932	glycogen synthase kinase 3 beta
HIF1A	14q21-q24	3091	hypoxia-inducible factor 1, alpha subunit
HLA-B	6p21.3	3106	major histocompatibility complex, class I, B
HLA-C	6p21.3	3107	major histocompatibility complex, class I, C
HLA-DQA1	6p21.3	3117	major histocompatibility complex, class II, DQ alpha 1
HNF4A	20q12-q13.1	3172	hepatocyte nuclear factor 4, alpha
HNT	11q25	50863	neurotrimin
HP	16q22.1	3240	haptoglobin
HSPG2	1p36.1-p34	3339	heparan sulfate proteoglycan 2
HTR1A	5q11.2-q13	3350	5-hydroxytryptamine (serotonin) receptor 1A
HTR1B	6q13	3351	5-hydroxytryptamine (serotonin) receptor 1B
HTR3B	11q23.1	9177	5-hydroxytryptamine (serotonin) receptor 3B
ICAM1	19p13.3-p13.2	3383	intercellular adhesion molecule 1
IGF1R	15q26.3	3480	insulin-like growth factor 1 receptor
IGHM	14q32.33	3507	immunoglobulin heavy constant mu

IL1A	2q14	3552	interleukin 1, alpha
IL2	4q26-q27	3558	interleukin 2
IL6	7p21	3569	interleukin 6 (interferon, beta 2)
IL8RB	2q35	3579	interleukin 8 receptor, beta
IMPG2	3q12.2-q12.3	50939	interphotoreceptor matrix proteoglycan 2
ING4	12p13.31	51147	inhibitor of growth family, member 4
INS	11p15.5	3630	insulin
INSR	19p13.3-p13.2	3643	insulin receptor
IRS1	2q36	3667	insulin receptor substrate 1
IRS2	13q34	8660	insulin receptor substrate 2
ITGA2	5q23-q31	3673	integrin, alpha 2
ITGB2	21q22.3	3689	integrin, beta 2
ITGB5	3q21.2	3693	integrin, beta 5
KCNJ11	11p15.1	3767	potassium inwardly-rectifying channel, subfamily J, member 11
KCNS1	20q12	3787	potassium voltage-gated channel, delayed-rectifier, subfamily S, member 1
KDR	4q11-q12	3791	kinase insert domain receptor
KHDRBS3	8q24.2	10656	KH domain containing, RNA binding, signal transduction associated 3
KLK1	19q13.3	3816	kallikrein 1
LEP	7q31.3	3952	leptin
LEPR	1p31	3953	leptin receptor
LGALS3	14q21-q22	3958	lectin, galactoside-binding, soluble, 3
LPL	8p22	4023	lipoprotein lipase
LTA	6p21.3	4049	lymphotoxin alpha (TNF superfamily, member 1)
MAP2K2	19p13.3	5605	mitogen-activated protein kinase kinase 2
MAPK3	16p11.2	5595	mitogen-activated protein kinase 3
MEF2A	15q26	4205	myocyte enhancer factor 2A
MEF2C	5q14	4208	myocyte enhancer factor 2C
MMP3	11q22.3	4314	matrix metallopeptidase 3
MPO	17q23.1	4353	myeloperoxidase
NFKB1	4q24	4790	nuclear factor of kappa light polypeptide gene enhancer in B-cells 1
NLGN1	3q26.31	22871	neuroligin 1
NOS3	7q36	4846	nitric oxide synthase 3
NOX3	6q25.1-q26	50508	NADPH oxidase 3
NOX4	11q14.2-q21	50507	NADPH oxidase 4

NOX5	15q23	79400	NADPH oxidase, EF-hand calcium binding domain 5
NOXA1	9q34.3	10811	NADPH oxidase activator 1
NPY	7p15.1	4852	neuropeptide Y
NR2E1	6q21	7101	nuclear receptor subfamily 2, group E, member 1
NR2E3	15q22.32	10002	nuclear receptor subfamily 2, group E, member 3
NR2F2	15q26	7026	nuclear receptor subfamily 2, group F, member 2
NXPH1	7p22	30010	neurexophilin 1
OLR1	12p13.2-p12.3	4973	oxidized low density lipoprotein receptor 1
OTX2	14q21-q22	5015	orthodenticle homeobox 2
PDGFA	7p22	5154	platelet-derived growth factor alpha polypeptide
PDGFB	22q13.1	5155	platelet-derived growth factor beta polypeptide
PECAM1	17q23	5175	platelet/endothelial cell adhesion molecule
PIGF	2p21-p16	5281	phosphatidylinositol glycan anchor biosynthesis, class F
PLA2G4A	1q25	5321	phospholipase A2, group IVA
PNMT	17q21-q22	5409	phenylethanolamine N-methyltransferase
PON1	7q21.3	5444	paraoxonase 1
PPARG	3p25	5468	peroxisome proliferator-activated receptor gamma
PPARGC1A	4p15.1	10891	peroxisome proliferator-activated receptor gamma, coactivator 1 alpha
PRKCB1	16p11.2	5579	protein kinase C, beta 1
PRKCE	2p21	5581	protein kinase C, epsilon
PROS1	3q11.2	5627	protein S (alpha)
PRPF31	19q13.42	26121	PRP31 pre-mRNA processing factor 31 homolog
PTGES	9q34.3	9536	prostaglandin E synthase
PTGIS	20q13.13	5740	prostaglandin I2 (prostacyclin) synthase
PTGS1	9q32-q33.3	5742	prostaglandin-endoperoxide synthase 1
PTGS2	1q25.2-q25.3	5743	prostaglandin-endoperoxide synthase 2
PTK2	8q24-qter	5747	PTK2 protein tyrosine kinase 2
PXN	12q24.31	5829	paxillin
RAGE	14q32	5891	renal tumor antigen
RALGPS2	1q25.2	55103	Ral GEF with PH domain and SH3 binding motif 2
RAPGEF1	9q34.3	2889	Rap guanine nucleotide exchange factor (GEF) 1
REN	1q32	5972	renin
RGMA	15q26.1	56963	RGM domain family, member A
ROBO2	3p12.3	6092	roundabout, axon guidance receptor, homolog 2

RRAD	16q22	6236	Ras-related associated with diabetes
RXRA	9q34.3	6256	retinoid X receptor, alpha
SCNN1A	12p13	6337	sodium channel, nonvoltage-gated 1 alpha
SELE	1q22-q25	6401	selectin E
SELP	1q22-q25	6403	selectin P
SERPINE1	7q21.3-q22	5054	serpin peptidase inhibitor, clade E member 1
SERPINF1	17p13.1	5176	serpin peptidase inhibitor, clade F member 1
SGK1	6q23	6446	serum/glucocorticoid regulated kinase 1
SLC1A2	11p13-p12	6506	solute carrier family 1 member 2
SLC1A3	5p13	6507	solute carrier family 1 member 3
SLC24A3	20p13	57419	solute carrier family 24 member 3
SLC2A1	1p35-p31.3	6513	solute carrier family 2 member 1
SLC2A11	22q11.2	66035	solute carrier family 2 member 11
SLC2A2	3q26.1-q26.2	6514	solute carrier family 2 member 2
SLC2A4	17p13	6517	solute carrier family 2 member 4
SLC6A3	5p15.3	6531	solute carrier family 6 member 3
SLC6A6	3p25-p24	6533	solute carrier family 6 member 6
SMAD3	15q22.33	4088	SMAD family member 3
SNX16	8q21.13	64089	sorting nexin 16
SOD1	21q22.1	6647	superoxide dismutase 1, soluble
SOD2	6q25.3	6648	superoxide dismutast 2, mitochondrial
SORD	15q15.3	6652	sorbitol dehydrogenase
STAT3	17q21.31	6774	signal transducer and activator of transcription 3
SUMO4	6q25	387082	SMT3 suppressor of mif two 3 homolog 4
TAS2R1	5p15	50834	taste receptor, type 2, member 1
TF	3q22.1	7018	transferrin
TFRC	3q29	7037	transferrin receptor (p90, CD71)
TGM2	20q12	7052	transglutaminase 2
TGM3	20q11.2	7053	transglutaminase 3
TNF	6p21.3	7124	tumor necrosis factor
TPH2	12q21.1	121278	tryptophan hydroxylase 2
TULP3	12p13.3	7289	tubby like protein 3
UBE2I	16p13.3	7329	ubiquitin-conjugating enzyme E2I
VCAM1	1p32-p31	7412	vascular cell adhesion molecule 1

VEGFA	6p12	7422	vascular endothelial growth factor A
VEGFB	11q13	7423	vascular endothelial growth factor B
VPS45	1q21.2	11311	vacuolar protein sorting 45 homolog
WNK1	12p13.3	65125	WNK lysine deficient protein kinase 1
WNT5B	12p13.3	81029	wingless-type MMTV integration site family, member 5B
